# A Theoretical Analysis of Interaction Energies and Intermolecular Interactions between Amphotericin B and Potential Bioconjugates Used in the Modification of Nanocarriers for Drug Delivery

**DOI:** 10.3390/molecules28062674

**Published:** 2023-03-15

**Authors:** Jennifer Cuellar, Lorena Parada-Díaz, Jorge Garza, Sol M. Mejía

**Affiliations:** 1 Línea de Investigación en Química Computacional, Grupo de Investigación GIFUJ, Departamento de Química, Facultad de Ciencias, Pontificia Universidad Javeriana, Bogota 110231, Colombia; 2 Departamento de Química, Universidad Autonoma Metropolitana-Iztapalapa, San Rafael Atlixco 186, Col. Vicentina, Iztapalapa, Ciudad de Mexico 09340, Mexico

**Keywords:** amphotericin B, bioconjugates, weak interactions, DFT, NBO, NCI

## Abstract

Amphotericin B (AmB) is an antibiotic with a wide spectrum of action and low multidrug resistance, although it exhibits self-aggregation, low specificity, and solubility in aqueous media. An alternative for its oral administration is its encapsulation in polymers modified with bioconjugates. The aim of the present computational research is to determine the affinity between AmB and six bioconjugates to define which one could be more suitable. The CAM-B3LYP-D3/6-31+G(d,p) method was used for all computational calculations. The dimerization enthalpy of the most stable and abundant systems at pH = 7 allows obtaining this affinity order: AmB_1,2-distearoyl-sn-glycerol-3-phosphorylethanolamine (DSPE) > AmB_γ-cyclodextrin > AmB_DSPEc > AmB_retinol > AmB_cholesterol > AmB_dodecanol, where DSPEc is a DSPE analog. Quantum theory of atoms in molecules, the non-covalent interactions index, and natural bond orbital analysis revealed the highest abundance of noncovalent interactions for AmB-DSPE (51), about twice the number of interactions of the other dimers. Depending on the interactions’ strength and abundance of the AmB-DSPE dimer, these are classified as strong: O-H---O (2), N-H---O (3) and weak: C-H---O (25), H---H (18), C-H---C (3). Although the C-H---O hydrogen bond is weak, the number of interactions involved in all dimers cannot be underestimated. Thus, non-covalent interactions drive the stabilization of copolymers, and from our analysis, the most promising candidates for encapsulating are DSPE and γ-cyclodextrin.

## 1. Introduction

Amphotericin B (AmB) is a polyene and macrolide antibiotic used for its ability to overcome multidrug resistance and a wide spectrum of action in the treatment of serious and life-threatening fungal infections [1]. At the present, the formulations commonly supplied correspond to AmB-deoxycholate (Fungizone^®^, Barcelona, Spain), liposomal AmB (AmBisome^®^, Northbrook, IL, USA), and fluconazole used in therapy of Candida sp and Cryptococcus, pathogens with a significant incidence in invasive fungal disease [2,3].

Specifically, this macrolide (C_47_H_73_NO_17_) is made up of a hydrophobic section with seven carbon atoms linked by conjugated double bonds, methyl groups, an ester group, a hydrophilic section composed mainly of hydroxyl groups, a carboxyl group, and a mycosamine [4]. In this sense, the molecule presents an amphipathic behavior due to hydrophobic and hydrophilic groups and simultaneously an amphoteric behavior at physiological pH, given by the carboxyl group that acts as a proton donor (acid) and the amino group (mycosamine) as a proton acceptor (base) [5].

Due to the presence of hydrophobic groups, the AmB exhibits low solubility in an aqueous medium, and consequently this antibiotic is frequently formulated in a colloidal solution whose administration in most cases is intravenous given its low oral bioavailability rate (0.3%), which involves complications due to the fragmentation in specific receptors [6,7]. The AmB’s mechanism of action results from its affinity for ergosterol, the principal sterol of fungal cells. In essence, AmB interacts with the cytoplasmic membrane of fungal cells, destabilizing the phospholipids, thus creating channels that disrupt the ionic environment, causing cell lysis [8,9]. However, despite its remarkable effectiveness in combating fungal diseases, the challenges that AmB faces for its use in the human body are still diverse. Thus, it is necessary to investigate and develop methods to reduce its toxicity and its affinity for the sterols present in human cells, such as cholesterol [10]. It is essential to reduce the AmB side effects that mainly affect kidneys, liver, and blood, whose symptoms can range from headaches and nausea to anemia and cardiac arrhythmia [11].

On the other hand, the AmB tends to self-aggregate; that is, it forms dimers and trimers when it encounters an aqueous medium. This feature reduces the efficacy and specificity of AmB towards ergosterol in fungal cells and increases its hydrophobic urge for insertion into unspecified membranes (ergosterol-cholesterol). This effect causes an increase in toxicity and, therefore, causes the complications described above [12]. Due to this, it has been found that the best way to administer AmB is in monomeric form. However, the environment composition prevents AmB from being distributed in this way [13].

Naturally, different alternatives have been explored to reduce the biotoxicity and increase the bioavailability of AmB through encapsulation with less toxic substances such as liposomes and polymeric micelles, since they are versatile carriers of hydrophobic guests through the lipophilic membrane that prevent self-aggregation; subsequently, AmB prefers to form interactions with this type of compound instead to form aggregates [14]. Among the main advantages found in this method are greater solubility without interfering with the efficacy in the treatment of fungal infections, minimization of side effects, and protection during the time of circulation in the body [12,15,16]. However, and in cases such as micelles, a low load capacity caused by weak host-guest interactions has been perceived, limiting their encapsulation capacity [17]. For this reason, Abdellatif et al. proposed the incorporation of hydrophobic compounds (partners conjugates) into the copolymer core to increase the guest affinity [18].

The host-guest interactions of conjugated polymers are stronger than those of nonconjugated polymers [19]. In this sense, the analysis criteria for the selection of aggregates are defined by the nature and magnitude of the interactions generated with AmB. Thus, and following these characteristics, examples of compounds that have aroused great interest are cholesterol, retinol, γ-cyclodextrin (γ-CD), dodecanol, and phosphorylethanolamine phospholipid (DSPE). For instance, Jia et al., in comparative studies of β-CD and γ-CD, found that van der Waals forces and electrostatic interactions are the main forces promoting inclusion in active sites [1,20].

In particular, the DSPE has shown favorable results in the disaggregation and delivery of AmB; in studies with micelles, it was shown that the maximum tolerable amount of administration of a conventional compound, such as Fungizone^®^, was lower compared to aggregated nanoparticles, being 3.0 mg for Fungizone^®^ and 10 mg for DSPE. This allows a more efficient formulation where organic solvents or complex techniques are not involved [14,21]. A similar case occurs with compounds such as retinol and cholesterol because they increase the controlled and sustained supply of active ingredients, achieving a drug content of 10.21% for retinol and 5.88% for cholesterol with lower release rates, significantly improving the accumulation [7,18].

Although there are experimental reports on this topic, the behavior of AmB when interacting with bioconjugates is still unclear, so a detailed computational analysis of these interactions is critical to understanding the nature of these interactions, which reduce the self-aggregation of this antibiotic [17].

Accordingly, the present theoretical study explores and analysis the interactions of AmB with six bioconjugates: cholesterol (Chol), retinol (Ret), γ-CD (Cyc), dodecanol (Dod), DSPE, and a DSPE analog (DSPEc), which contains carbon chains shorter than those presented in DSPE. Thus, this article, through computational simulations, describes and identifies the most promising bioconjugate to encapsulate the AmB. It is expected that the present results will be used later in experimental research, facilitating the selection of bioconjugates according to their performance in modifying the encapsulating material, thus providing a more accurate approach to overcome the limitations of the drug. The detailed abbreviations and definitions used in the paper are listed in Table 1.

## 2. Results and Discussion

### 2.1. Potential Energy Surface Exploration

**Figure 1:**
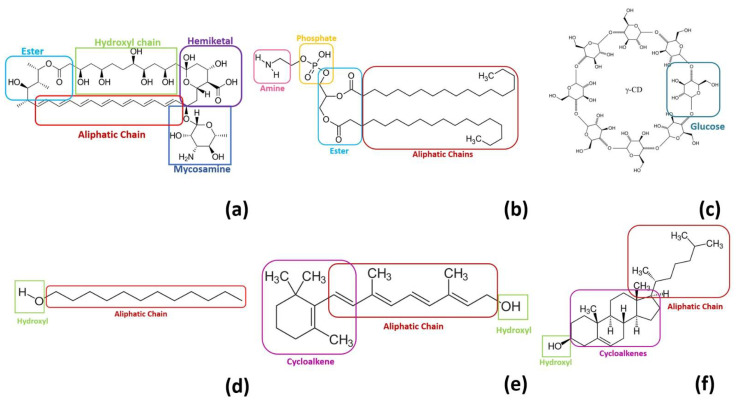
Functional group classification of the monomers. (**a**) amphotericin B, (**b**) DSPE, (**c**) γ-cyclodextrin, (**d**) dodecanol, (**e**) retinol, (**f**) cholesterol.

The potential energy surface (PES) exploration of five types of dimers, AmB-DSPE, AmB-Cyc, AmB-Chol, AmB-Ret and AmB-Dod, was performed by a stochastic method. The hybrid SnippetKick algorithm was used [22], where a maximum of 100 structures were obtained for each dimer, which were classified into categories according to the different orientations of the functional groups in both molecules, the host (amphotericin B) and the guest (biomolecule or bioconjugate). Figure 1 highlights the most important functional groups for each monomer. For example, for AmB there are five types of functional groups: ester, hydroxyl chain, hemiketal, aliphatic chain, and a mycosamine. The number of subgroups varies depending on the number of functional groups present in each molecule and their coupling. The dimers with the lowest dimerization energy of each subgroup were finally the starting structures for the reoptimization and frequencies calculations with DFT, obtaining 12 dimers of AmB-Dod, 7 of AmB-Chol, 10 of AmB-DSPE, seven of AmB-Ret and seven of AmB-Cyc.

Furthermore, from the 10 AmB-DSPE dimers, 10 AmB-DSPEc dimers were built and seven optimized. Additionally, using electrostatic potential maps, four more AmB-Dod dimers and nine more AmB-Chol dimers were proposed. Therefore, the total number of proposed dimers was 70: AmB-DSPE (10), AmB-Cyc (7), AmB-DSPEc (7), AmB-Chol (16), AmB-Ret (14) and AmB-Dod (16).

### 2.2. Geometries and Thermodynamic Properties

Once the 70 AmB-bioconjugates dimers were optimized and confirmed as stable systems (zero imaginary frequencies), different thermodynamic properties were calculated. These values are reported in Table S1 (see Supporting Information (SI)). As expected, the dimerization process was exothermic (ΔE < 0, ΔH < 0), in all cases it was spontaneous (ΔG < 0), and implies a loss of degrees of freedom compared to having the isolated monomers (ΔS < 0). The dimers of each type were arranged from the most stable to the least stable according to the ΔH value and were named AmB-bioconjugateX where 0 < X ≤ 17, since 17 stable dimers were obtained for the AmB-CholX system.

**Figure 2:**
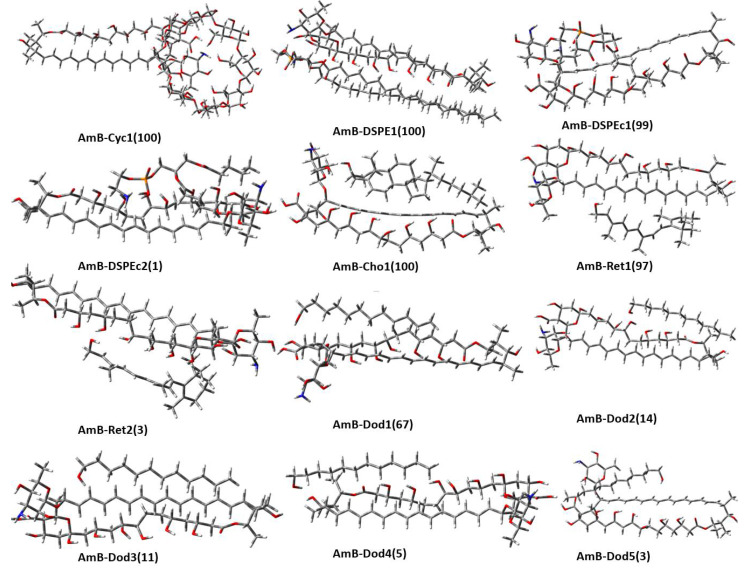
Optimized geometries of the most abundant AmB-bioconjugate dimers. Isomeric abundances in parentheses.

According to the abundance percentage for each type of dimer, those with abundances greater than or equal to 1% were determined as representative dimers (see Figure 2). Five AmB-Dod dimers with abundances between 3% and 67% were found, two AmB-Ret dimers with abundances of 97% and 3%, respectively, for the AmB-DSPEc system an abundance of 99% and 1% was calculated for its second most stable dimer. For the rest of the systems, only one dimer is representative, with an abundance of 100%. 

**Figure 3:**
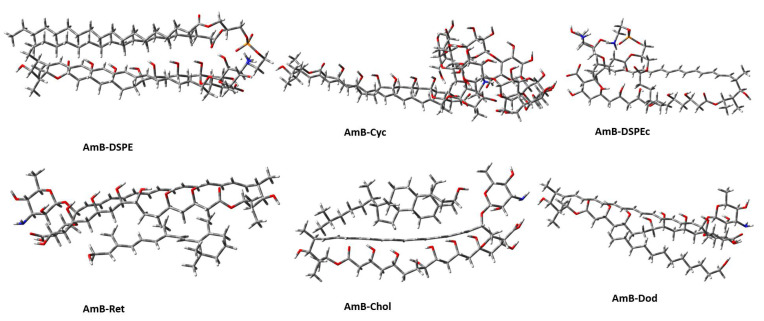
Optimized geometries of each AmB-bioconjugate dimers including protonation and deprotonation in basic and acid groups, respectively.

The most stable and abundant dimer of each system was selected to include protonation and deprotonation in basic and acid groups, respectively. At pH = 7, AmB is protonated at the amine of the mycosamine group and deprotonated at the carboxylic acid of the hemiketal group. In DSPE and DSPEc, protonation in the amine group and deprotonation in the phosphate group were observed. Thus, AmB, DSPE and DSPEc monomers were reoptimized, and all dimers were reoptimized maintaining protonated and deprotonated groups during the geometry optimization. The optimized geometries can be seen in Figure 3, and the thermodynamic values of dimerization are found in Table 2. Dimers were organized from most to lest stable according to ΔH. 

From Table 2, the dimerization was a thermodynamically favorable process (exothermic (ΔE and ΔH < 0) and spontaneous (ΔG < 0)), with a loss of freedom degrees compared to free monomers (ΔS < 0). Based on the dimerization enthalpy, the stabilization order is AmB-DSPE > AmB-Cyc > AmB-DSPEc > AmB-Ret > AmB-Chol > AmB-Dod. The AmB-DSPE dimer is the most stable with a value of −141.46 kcal/mol, while the AmB-Dod dimer is the less stable with a dimerization enthalpy of −26.11 kcal/mol. Comparing the AmB-DSPEc dimer with AmB-DSPE, the difference of 63.72 kcal/mol is observed. This shows that the DSPEc bioconjugate does not provide a valid approximation to the interaction behavior of DSPE with AmB. The modification in DSPEc affects the abundance of possible weak interactions, limiting the groups of AmB that can interact with this fragment compared to DSPE (see Figure 3).

The dimerization energy follows the same order (AmB-DSPE > AmB-Cyc > AmB-DSPEc > AmB-Ret > AmB-Chol > AmB-Dod), evidenced according to the dimerization enthalpy, thus the dimer with the most negative value was AmB-DSPE with −110.66 kcal/mol, and the least negative was AmB-Dod with −16.01 kcal/mol. The comparison between ΔE and ΔH found differences between −10 and −31 kcal/mol, so the effect of the temperature predicts that dimerization processes are considerably more favorable (more exothermic) at 298.15 K.

The Gibbs free energy of dimerization follows the same order as the dimerization enthalpy, so the dimerization process occurs more spontaneously in AmB-DSPE with a value of −101.65 kcal/mol, followed by the AmB-Cyc dimer with a value of −69.69 kcal/mol. The less spontaneous process is presented by the dimerization of dodecanol with the AmB (ΔG = −9.50 kcal/mol). 

Finally, the dimerization entropy predicts the dimerization between AmB and DSPE as the process with the highest entropy loss (−133.53 cal/molK), followed by AmB-DSPEc (−89.84 cal/molK). It agrees with the observation that the aliphatic chains of DSPE interact with most of the AmB functional groups, restricting the freedom degrees of movement in the dimer in both fragments. Although the AmB-Cyc dimer is more stable than AmB-DSPEc, but it ranks after this dimer in terms of entropy and has a less negative entropy change. In turn, γ-cyclodextrin does not have a good coupling with AmB compared to DSPE because it only interacts with mycosamine, the carboxylic acid of the hemiketal and part of the hydroxyl chain (see Figure 1 and Figure 3). Thus, this suggests that the entropy change can be interpreted in terms of a higher or lower coupling between the monomers that form the dimer; therefore, the abundance and/or strength of the intermolecular interactions can explain the geometric preferences and the different thermodynamics of the dimers.

The geometries resulting from the dimerization process are essential because they can influence the distribution of AmB in the human body, either positively or negatively. For example, the dimers that interact with the mycosamine group of AmB may present a disadvantage compared to the dimers that do not interact because, as has been found in previous studies such as that proposed by Wilcock et al., the mycosamine is the most important functional group of AmB because it provides the antifungal activity [23]. Thus, if degradation of the bioconjugate is not possible during the AmB administration, dimers that do not interact with mycosamine can still interact with ergosterol from the fungal cells, otherwise dimers such as AmB-DSPE, AmB-Ret, AmB-Chol, and AmB-Dod would have an advantage over AmB-Cyc and AmB- DSPEc.

For the analysis of dimers’ geometries, the preferred orientation reported in this study for dimerization between AmB and γ-cyclodextrin led to an inclusion complex between the mycosamine of AmB with the primary face of γ-cyclodextrin. Seven stable AmB-Cyc dimers were obtained, although the two most stable form inclusion complexes, both of which bound to the primary face of γ-cyclodextrin. It is possible to observe for this system that most of AmB functional groups are free except for the mycosamine that was inside the inclusion complex, the hemiketal group, and part of the hydroxyl chain, that, although they were not inside of inclusion complex, also interact with γ-cyclodextrin. For the AmB-DSPE dimer, it can be seen that DSPE interacts with most of the AmB functional groups except for mycosamine. The AmB-DSPEc dimers follow the same trend as the AmB-DSPE dimer, but two differences can be seen: the first is that due to the cutting of the aliphatic chains, the interactions with AmB functional groups are less and it is possible to see interaction with the DSPEc and the mycosamine group of the AmB. The rest of the systems have preferences for nonpolar functional groups of AmB (see Figure 2).

### 2.3. Non-Covalent Interactions Analysis from Quantum Chemistry Tools

#### 2.3.1. Quantum Theory of Atoms in Molecules Analysis

**Figure 4:**
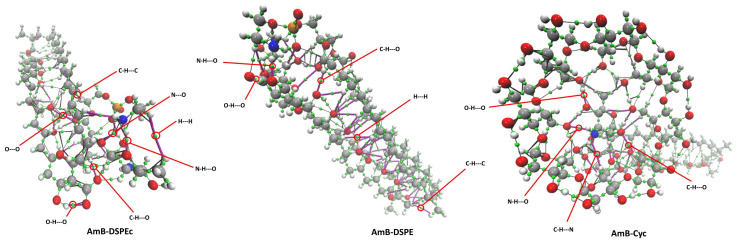
Molecular graphs highlighting the different types of intermolecular interactions. Small green spheres: bond critical points. Purple lines: bond path associated with intermolecular interactions. Black lines: bonds paths associated with intramolecular interactions.

The formation of intermolecular interactions and their analysis in terms of strength and abundance were performed using quantum theory of atoms in molecules (QTAIM) and non-covalent interactions index (NCI) analyses. According to molecular graphs, there are eight types of interactions involved in the dimers considered in this work O-H---O, C-H---O, H---H, C-H---C, C-H---N, O---O, N-H---O, N---O. Figure 4 shows the molecular graphs of AmB-DSPE, AmB-DSPEc and AmB-Cyc, highlighting the bond critical points (BCP) associated with all different types of interactions that can be observed. The six molecular graphs of the most stable dimer of each type can be seen in Figure S1 in the ESI. In general, the interaction between the aliphatic zones of the monomers is observed, prevailing the C-H---C, C-H---O, H---H and O-H---O interactions.

Based on the QTAIM, the nature of the interactions was evaluated through the values of five topological parameters evaluated at the BCPs: electron density ρ(r_CP_), Laplacian of the electron density ∇^2^ρ(r_CP_), electron energy density H(r_CP_), and the relation between the Virial field lV(r_CP_)l and the kinetic energy G(r_CP_) (lV(rcp)l/G(rcp)). Average values and ranges of these topological parameters can be seen in Table 3 and Table S2, respectively, along with the bond lengths and the abundance of each interaction type.

Table 3 confirms well-established observations; for example, strong hydrogen bonds (HBs) such as O-H---H and O-H---N exhibit shorter bond lengths and consequently the highest values of ρ(r_CP_) [24,25]. However, the number of weak non-covalent interactions such as C-H---O and H---H is relevant and, in some cases, they are responsible for the stabilization of some dimers. The Espinosa-Molins-Lecomte approach was used to estimate the strength of each non-covalent interaction. The interaction energy (IE) from this approach is reported in Table 3. From this table, there is a nice match between IE and ρ(r_CP_), which is good, since many times ρ(rCP) has been used to indicate the strength of a non-covalent contacts. We stress this point since the Espinosa-Molins-Lecomte approach was designed for HBs. However, we observe that even cases such as H---H and O---O follow this approach. By looking only HBs, the role of the C-H---O contacts is clear; they present a small IE, but they are by far who predominate in the stabilization of the dimers considered in this article. This conclusion is crucial to understanding the forces responsible for the formation of dimers between AmB and the six systems proposed in this article. 

Note that the AmB-DSPE dimer is the most stable dimer. However, its interactions may be less strong than those in dimers such as AmB-Chol and AmB-Dod; therefore, not only the strength of the intermolecular interactions is decisive for the stabilization of the dimers, but also their abundance. This is also observed in the AmB-Dod dimer; despite having one of the strongest interactions (ρ(r_CP_) for C-H---C = 3.06 × 10^−2^ a.u), it is ranked as the least stable because it has the lowest number of interactions of all the dimers studied.

On the other hand, it has been reported that H---H interactions are usually longer and weaker than C-H---O interactions in other types of clusters, such as those observed between ethanol or methanol molecules or clusters of those alcohols with water molecules [26]. For the (methanol)_5_-water system, the difference between the topological parameters shows that C-H---O has a magnitude greater than H---H interactions, indicating that interactions such as C-H---O are of great importance in the formation of these clusters [27]. However, in the present study, several H---H interactions are as short and strong as some C-H---O interactions. For a complete overview, see Table S2 (ranges data) and Table 3 (average values).

It Iils noteworthy that the AmB-DSPE dimer exhibits the two strongest interactions (O-H---O and N-H---O), although interactions such as C-H---O, H---H and C-H---C in strength can be overcome by the same type of interactions in other dimers, their abundance is higher for this dimer. The IE confirms this conclusion, and it shows the relevance of non-conventional contacts, which are often overlooked.

Regarding the nature of the interactions, according to the Laplacian value, these show a closed-shell behavior since all of these interactions have positive values [28], indicating non-covalent interactions. The HBs can be classified considering the values of ∇^2^ρ(r_CP_) and those of H(r_CP_), following the criteria established by Rozas et al. [29] where the HBs are strong when H(r_CP_) < 0 and ∇^2^ρ(r_CP_) > 0, very strong when H(r_CP_) < 0 and ∇^2^ρ(r_CP_) < 0, and weak when H(rc) > 0 and ∇^2^ρ(r_CP_) > 0. It is verified that the interactions with the highest Laplacians in all the dimers of this research belong to the strong category. These results can be supported by the lV(r_CP_)l/G(r_CP_) parameter, which shows that the strongest HBs are in the range of 1 and 2; thus, these have both behaviors, closed-shell and open-shell. For the rest of the interactions, the values are in the range of 0 and 1, so those are exclusively closed shell. In addition, IE confirms these arguments as we have discussed above.

#### 2.3.2. Non-Covalent Interactions Index Analysis

For a complementary analysis of the interactions strength and nature, the non-covalent interactions index (NCI) was chosen. NCI allows describing non-covalent interactions through a 3D representation of the reduced density gradient [30].

**Figure 5:**
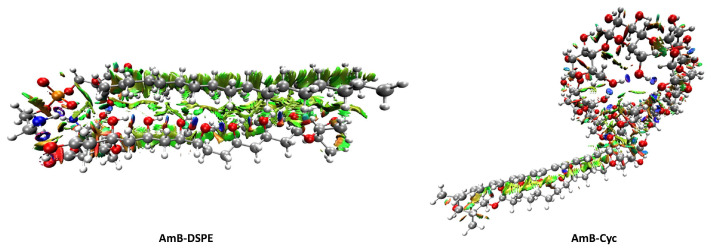
3D NCI plots revealing non-covalent interactions in real space. Blue surfaces: strong attractive interactions, green surfaces: weak attractive interactions, red surfaces: strong repulsive interactions.

Figure 5 shows the 3D isosurfaces of AmB-DSPE and AmB-Cyc, and Figure S1 shows the 3D NCI plots for each dimer. All systems exhibit both strong attractive interactions (blue surfaces) and weak attractive interactions (green surfaces). A higher abundance of strong interactions (blue surfaces) in AmB-Cyc stands out. The predominant interactions in all dimers have a weak character since most of the isosurfaces are green, so dispersion zones are relevant for the dimers’ stabilization. No attractive interactions were found within cycles and as part of intramolecular interactions.

Another point that is important to highlight is that this analysis allowed us to observe that monomers such as AmB, γ-cyclodextrin and cholesterol are stabilized by intramolecular interactions that can limit their flexibility when forming dimers. For example, the γ-cyclodextrin has a considerable number of intramolecular interactions that only allow it to interact with the mycosamine, the carboxylic acid of hemiketal, and part of the hydroxyl chain of AmB. In contrast, DSPE can interact with most AmB functional groups because its aliphatic chains do not have intramolecular interactions, which gives it great flexibility for dimer formation. Regarding the above, see the 3D plots of the monomers in Figure S1.

It can be inferred that this greater flexibility of the DSPE and its large size contribute at the experimental level to the fact that the encapsulating material modified with DSPE presents better encapsulation percentages than Fungizone^®^ [31,32]. Note that the dodecanol monomer is limited in its affinity for AmB due to its smaller size, which only allows it to interact with one or a maximum of two AmB functional groups. This may suggest that the encapsulation capacity of these systems is somewhat lower than DSPE-modified systems [33,34].

#### 2.3.3. NBO Analysis

According to the results obtained for the thermodynamic properties, the NBO analysis was performed for the most stable dimer of each type with and without the protonation and deprotonation process. This analysis allows quantitatively estimating the second-order stabilization energy E_i→j_^(2)^ for each of the possible donor (i)-acceptor (j) interactions according to the Lewis structure for Lewis-type NBOs (occupied) and non-Lewis NBOs (empty) [35]. For the present study, we report this property only for intermolecular interactions with a value of E_i→j_^(2)^ > 1 kcal/mol. Results can be found in Table S3 of the ESI. In accordance with the results obtained with NBO, the C-H---O, O-H---O, C-H---C and N-H---O type interactions contribute to the strength of the dimers through the second-order interaction energies while the other interactions identified with the QTAIM analysis (O---N, O---O, H---H, C-H---N) do not present values of E_i→j_^(2)^ > 1 kcal/mol, reinforcing the observation that the importance of these weak interactions lies in their abundance as a group rather than in their individual strength, as recently seen in other research explaining properties such as the boiling point and stabilization of systems such as heptane, based on the abundance of weak H---H interactions [36,37]. On the other hand, the number of significant interactions per dimer varied between two and 15, as follows: AmB-Cyc (15), AmB-DSPE (13), AmB-DSPEc (9), AmB-Chol (2), AmB-Ret (5) and AmB-Dod (2). Helga et al. [38] affirm by means of FT-IR analysis that for the AmB-Cyc dimer, hydrophilic and van der Waals interactions are responsible for maintaining equilibrium in the complex, instead of ionic or covalent bonds.

Considering for each type of interaction with the highest value of E_i→j_^(2)^, the highest stabilization of the N-O type occurred in the AmB-DSPE dimer in the lone pair orbital located at O_132_ of the AmB hemiketal with the σ* orbital of the H_267_-O_265_ bond of the DSPE phosphate group with a value equal to 31.37 kcal/mol. O-H---O type interactions present the second highest value = 27.28 kcal/mol for AmB-Dod between the O_172_ donor of the hemiketal of AmB and the H_39_-O_38_ acceptor of the hydroxyl of the bioconjugate. In the case of C-H---O interactions, the AmB-Cyc dimer presented the highest stabilization between the lone pair O_74_ of the glucose of the γ-cyclodextrin and the H_178_-C_177_ of the mycosamine present in AmB with a value of 4.85 kcal/mol. Finally, the AmB-DSPEc dimer presents the C-H---C type interaction with greater second order stabilization between the aliphatic chain of DSPEc and the mycosamine of AmB with a value of 1.45 kcal/mol. It should be noted that weak C-H---C type interactions were only present in AmB-DSPEc and AmB-Ret dimers.

## 3. Computational Procedure

**Figure 6:**
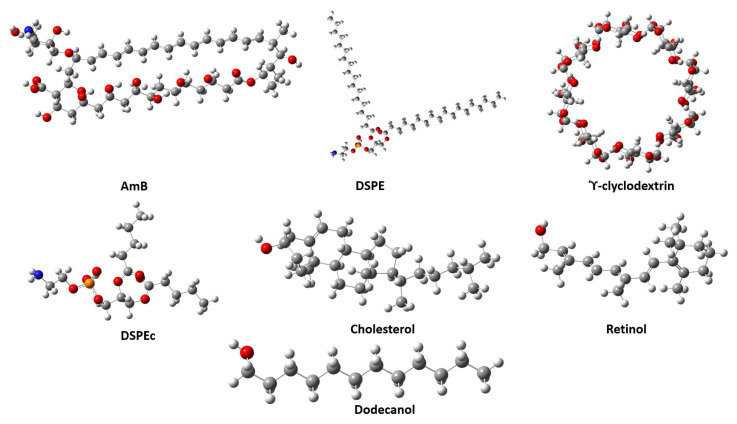
Optimized geometries of the monomers. DSPEc is a homologous structure of DSPE where its aliphatic chains are shorter than those of DSPE.

The starting structures for AmB, cholesterol (Chol), retinol (Ret), γ-cyclodextrin (Cyc), dodecanol (Dod), and DSPE were obtained from PubChem [31] and Chemspider [32]. All these structures underwent conformational analysis using molecular dynamics with the universal force field (UFF) in the Avogadro software (V1.2.0, Marcus D Hanwell et al. Lawrence, KS, USA) [33] and from snapshots, some structures were selected to be optimized with the CAM-B3LYP-D3/6-31G(d,p) electronic structure method. The selection of this method is based on previous reports related to systems such as those considered in this paper [34,39]. The lowest energy monomeric structures are shown in Figure 6.

AmB-Chol, AmB-Ret, AmB-Cyc, AmB-Dod, and AmB-DSPE starting dimers were obtained through the SnippetKick approach [22]. This approach generated 3000 structures for each dimer using UFF, where each monomer can rotate or translate within a cubic box with a side length of 1.5 times the size of the largest dimer (a monomer in front of the other monomer in a horizontal line). All these structures were optimized by the PM6-DH2 semiempirical method [40] as implemented in MOPAC2016 [41]. From here, some of the 100 structures with the lowest energy were reoptimized with the CAM-B3LYP-D3/6-31G(d,p) method (see Section 2.1 for more details). Some additional dimer structures were proposed based on the results of the electrostatic potential maps of the different monomers that connect the acceptor with the donor sites. Additionally, to evaluate the effect of the length of the aliphatic chains of DSPE in the formation of dimers with AmB, a sixth type of dimer was considered: AmB-DSPEc, where DSPEc means a short DSPE, a homologues molecule of DSPE. That is, the aliphatic chains of 17 carbon atoms of the DSPE molecule in the AmB-DSPE dimers were shortened to 4 carbon atoms and those geometries were reoptimized. Gaussian09 program (Gaussian 09, Revision E. 01, M. J. Frisch. et al., Wallingford CT, USA) [42] was used to all optimization and frequencies calculations.

The dimerization thermodynamic properties considered are: ΔE (0 K): energy, ΔH (298.15 K): enthalpy, ΔG (298.15 K): Gibbs free energy, ΔS (298.15 K): entropy in cal/(mol × K). This was achieved by means of the supermolecule approach [43] (see Equation (1)).

(1) $$\Delta X=X_{dimer}-\left(\sum X_{monomer}\right)$$

where X represents E, H, G or S. In the case of ΔE, the Counterpoise correction [44] was included to estimate the error due to base superposition error and the zero-point energy correction (ZPE). Additionally, the percentage of isomeric abundance ($\%X_{i}$) was calculated based on the Boltzmann distribution, see Equation (2).

(2) $$\%X_{t}=100\times \frac{e^{\frac{E_{j}}{kT}}}{\sum e^{-\frac{\Delta E_{j}}{kT}}}$$

where *k* is the Boltzmann constant = 1.38 × 10^−2^ J/K, T is the temperature (in our case, 298.15 K), and ΔE is the energy difference between the lowest energy conformer and the analyzed conformer.

Considering that the compounds must act at physiological pH [45], the changes in the molecular structures of the different monomers were analysed at pH = 7 using the Marvin Sketch program (V21.17.0, ChemAxon, Montreal, Canada) [46]. Then, AmB, DSPE and the most stable dimers of each type were reoptimized, taking care that protonation and deprotonation are maintained in the converged structures.

To characterize the weak interactions involved in the dimers considered in this work, the GPUAM (Graphics processing units for atoms and molecules. V1.0, R. Hernández-Esparza et al. Mexico City, Mexico) software [47] was used, where Bader’s Quantum Theory of Atoms in Molecules (QTAIM) is implemented [48]. Note that the GPUAM program performs the topological electron density calculations using GPUs. The non-covalent interactions index (NCI) was also used, which, through 2D and 3D representations, allows predicting the types of interactions, strength, and their location within the chemical species. This analysis was carried out using the Multiwfn software (V3.8, Tian Lu et al. Beijing, China) [49] and the molecular visualization program, VMD (V1.9.4, Humphrey, W et al. IL, USA) [50]. Additionally, the analysis of natural bonding orbitals was performed using the NBO (V3.1, E. D. Glendening, et al., Wallingford CT, USA) in Gaussian 09.

## 4. Conclusions

The present research focused on the characterization of intermolecular interactions between amphotericin B (AmB) and a series of six bioconjugates (γ-cyclodextrin = Cyc, Retinol = Ret, Cholesterol = Chol, dodecanol = Dod, phosphorylethanolamine phospholipid = DSPE and a homologous molecule of DSPE = DSPEc (with a shorter aliphatic chain)). Bioconjugates were chosen as potential nanoparticle modifiers that can encapsulate and release AmB in a controlled manner. The formation of all dimers considered in this article represents an exothermic and spontaneous process.

The highest affinity for AmB was presented by DSPE, followed by γ-cyclodextrin. Therefore, these two bioconjugates are proposed among the six studied as the most promising compounds to modify the encapsulating material of AmB, seeking increase the encapsulation percentage. The affinity order is as follows: AmB-DSPE > AmB-Cyc > AmB-DSPEc > AmB-Ret > AmB-Chol > AmB-Dod.

The truncation of the aliphatic chain’ of DSPE does not provide a good model since the number of interactions of a dispersive nature formed in the corresponding dimer is considerably reduced. NCI 3D plots demonstrated that such interactions cover large zones and are relevant for the stabilization.

The higher affinity for AmB that the DSPE bioconjugate exhibits is explained by the high abundance of intermolecular interactions when it interacts with amphotericin, showing excellent coupling when surrounding AmB with almost its entire structure. It is worth noting that besides conventional hydrogen bonds (O-H---O and N-H---O), non-conventional hydrogen bonds (C-H---O, C-H---C, and C-H---N) as well as O---O, H---H and O---N interactions contribute to the stabilization of the dimers considered in our study.

## Figures and Tables

**Table 1:** List of abbreviations and acronyms used in the paper.

Abbreviation	Definition	Abbreviation	Definition
AmB	Amphotericin B	ZPE	Zero point energy
DSPE	1,2-distearoyl-sn-glycerol-3-phosphorylethanolamine	UFF	Universal force field
Cyc	Cyclodextrin	HBs	Hydrogen bond
β-Cyc	Beta-cyclodextrin	BCP	Bond critical points
γ-Cyc	alpha-cyclodextrin	ρ(r_CP_)	Electron density
DSPEc	Homologous DSPE with a shorter aliphatic chain	∇^2^ρ(r_CP_)	Laplacian of the electron density
Ret	Retinol	H(r_CP_)	Total energy density
Chol	Cholesterol	(lV(r_CP_)l/G(r_CP_))	Relation between the Virial field and kinetic energy density
Dod	Dodecanol	IE	Interaction energy
AmB-DSPE	Dimer formed between Amphotericin B and 1,2-distearoyl-sn-glycerol-3-phosphorylethanolamine	$E_{i\to j}^{\left(2\right)}$	Donor–acceptor second-order interaction energies
AmB_γ-cyclodextrin or AmB-Cyc	Dimer formed between Amphotericin B and alpha-cyclodextrin	ΔE	Dimerization energy
AmB_DSPEc	Dimer formed between Amphotericin B and DSPE homologous with a shorter aliphatic chain	ΔH	Dimerization enthalpy
AmB_retinol or AmB-Ret	Dimer formed between Amphotericin B and retinol	ΔG	Dimerization Gibbs free energy
AmB_cholesterol or AmB-Chol	Dimer formed between Amphotericin B and cholesterol	ΔS	Dimerization entropy
AmB_dodecanol or AmB-Dod	Dimer formed between Amphotericin B and dodecanol	SI	Supporting Information
DFT	Density Functional Theory	PES	Potential Energy Surface
NCI	Non-Covalent interactions Index	QTAIM	Quantum theory of atoms in molecules

**Table 2:** Thermodynamic parameters predicted by the CAM-B3LYP-D3/6-31G(d,p) method of each AmB-bioconjugate dimer including protonated and deprotonated groups. ΔE, ΔH, and ΔG in kcal/mol. ΔS in cal/molK.

Dimers	ΔE	ΔH	ΔG	ΔS
AmB-DSPE	−10.66	−141.46	−101.65	−133.53
AmB-Cyc	−64.79	−95.30	−69.69	−85.88
AmB-DSPEc	−56.01	−77.74	−50.95	−89.84
AmB-Ret	−22.32	−34.86	−16.09	−62.94
AmB-Cho	−17.82	−31.51	−12.28	−64.49
AmB-Dod	−16.01	−26.11	−9.50	−55.69

**Table 3:** Abundance, average of length, and topological parameters at bond critical points (CP) for intermolecular interactions of each AmB-bioconjugate dimer, including protonation and deprotonation in basic and acid groups. Data predicted by the CAM-B3LYP-D3/6-31G(d,p) method.

IT ^a^	Length ^b^ [Å]	ρ(r_CP_) ^c^ 10^−2^	∇^2^ρ(r_CP_) ^d^ 10^−2^	H(r_CP_) ^e^ 10^−3^	lV(r_CP_)l/ G(r_CP_)) ^f^	IE ^g^ 10^−2^ [kcal/mol]	NI ^h^
AmB-DSPE							
O-H---O	1.70	4.88	13.33	−4.2	1.08	−2.09	2
C-H---O	2.68	0.75	2.71	0.86	0.82	−0.25	25
H---H	2.51	0.48	1.71	1.00	0.69	−0.11	18
C-H---C	3.04	0.43	1.51	0.88	0.71	−0.10	3
N-H---O	1.89	3.68	10.71	−1.06	1.01	−1.44	3
AmB-Cyc							
O-H---O	1.89	4.16	7.87	−10.64	1.11	−2.05	6
C-H---O	2.63	0.85	2.99	0.75	0.83	−0.30	1
H---H	2.06	1.01	3.66	1.6	0.79	−0.30	12
N-H---O	2.01	3.07	8.81	−0.90	1.01	−1.19	1
C-H---N	3.39	0.19	6.90	0.50	0.63	−0.04	1
AmB-DSPEc							
O-H---O	1.75	3.99	11.94	−1.03	1.03	−1.60	3
C-H---O	2.54	0.91	3.13	0.81	0.87	−0.31	11
H---H	2.43	0.56	2.09	1.10	0.70	−0.15	8
C-H---C	3.13	0.45	1.55	0.80	0.72	−0.12	2
O---O	3.10	0.66	2.74	0.90	0.86	−0.26	1
N-H---O	1.88	3.01	8.66	−0.80	1.04	−1.17	1
N---O	2.77	1.47	6.04	1.80	0.86	−0.58	1
AmB-Ret							
O-H---O	1.98	2.55	6.21	−1.80	1.10	−0.96	1
C-H---O	2.39	1.29	3.95	0.26	0.96	−0.47	5
H---H	2.39	0.58	2.07	1.14	0.70	−0.14	10
C-H---C	2.97	0.53	1.68	0.83	0.75	−0.13	11
AmB-Chol							
O-H---O	1.96	2.52	7.11	−1.00	1.05	−0.99	1
C-H---O	2.82	0.59	2.25	9.29	0.77	−0.19	7
H---H	2.32	0.64	2.30	12.20	0.72	−0.17	13
C-H---C	2.95	0.59	1.80	9.00	0.74	−0.13	5
AmB-Dod							
O-H---O	1.75	4.23	11.43	−1.80	1.06	−1.61	1
C-H---O	2.73	0.72	2.61	0.80	0.80	−0.24	4
H---H	2.47	0.49	1.69	0.95	0.70	−0.12	13
C-H---C	3.06	0.49	1.62	0.82	0.73	−0.12	5

^a^ Interaction type, ^b^ Euclidean distance between the attractors, ^c^ electron density, ^d^ electron density Laplacian, ^e^ total energy density, ^f^ virial field/kinetic energy, ^g^ interaction energy, ^h^ number of interactions.

## Data Availability

Not applicable.

## Supplementary Materials

The following supporting information can be downloaded at: https://www.mdpi.com/article/10.3390/molecules28062674/s1. Table S1. Thermodynamic parameters of the AmB-bioconjugates dimers. CAM-B3LYP-D3/6-31+G(d,p). Figure S1. Plots revealing noncovalent interactions at the monomers and the most stable dimer of each type including the pH effect. Monomer: 3D-NCI plots. Dimers: molecular graphs (left) where purple lines: bond path associated with intermolecular interactions. Black lines: bons paths associated with intramolecular interactions. 3D-NCI plots (right). Table S2. Length and topological parameters (ranges) evaluated at bond critical points (CP) for intermolecular interactions for the most stable dimer of each type including the pH effect. CAM-B3LYP-D3/6-31+G(d,p). Table S3. NBO Donor—acceptor second-order interaction energies $E_{i\to j}^{\left(2\right)}$ in kcal/mol for each interaction type of AmB-bioconjugate dimers including the pH effect. CAM-B3LYP-D3/6-31+G(d,p). Coordinates of the structures worked on in this paper.
