# Supplementary material for: A Theoretical Analysis of Interaction Energies and Intermolecular Interactions between Amphotericin B and Potential Bioconjugates Used in the Modification of Nanocarriers for Drug Delivery
Source: Molecules. 2023 Mar 15;28(6):2674. doi: 10.3390/molecules28062674 (PMC10055876; doi:10.3390/molecules28062674)
Supplement: Supplementary file 1 [file molecules-28-02674-s001.zip › molecules-2132970-supplementary.pdf]

A Theoretical Analysis of Interactions Energies and Intermolecular Interactions between Amphotericin B and Potential Bioconjugates Used in the Modification of Nanocarriers for Drug Delivery

Jennifer Cuellar [1,†] Lorena Parada-Díaz [1,†] Jorge Garza [2] and Sol M. Mejía [1,*]

[1] Línea de Investigación en Química Computacional, Grupo de Investigación GIFUJ, Departamento de Química, Facultad de Ciencias, Pontificia Universidad Javeriana, Bogotá 110231, Colombia

[2] Departamento de Química, Universidad Autónoma Metropolitana-Iztapalapa. San Rafael Atlixco 186. Col. Vicentina, Iztapalapa, Ciudad de Mexico 09340, México

* Corresponding author email: sol.mejia@javeriana.edu.co

† These authors contributed equally to this work.

Electronic Supporting Information

Table S1. *Thermodynamic parameters of the AmB-bioconjugates dimers. CAM-B3LYP-D3/6-31+G(d,p)*

Dimers	%X	ΔE[a,b]	ΔH[b]	ΔG[b]	ΔS[c]
**AmB-Cyc**					
**AmB-Cyc1**	100.0	-83.05	-83.80	-62.57	-71.20
**AmB-Cyc2**	0.0	-75.15	-75.71	-54.95	-69.64
**AmB-Cyc3**	0.0	-49.51	-49.61	-31.17	-61.85
**AmB-Cyc4**	0.0	-27.79	-27.63	-13.75	-46.55
**AmB-Cyc5**	0.0	-27.77	-27.35	-12.37	-50.25
**AmB-Cyc6**	0.0	-25.66	-25.87	-12.24	-50.33
**AmB-Cyc7**	0.0	-22.18	-22.65	-9.47	-37.18
**AmB-DSPE**					
**AmB-DSPE1**	100.00	-81.72	-84.30	-47.02	-125.04
**AmB-DSPE2**	0.00	-65.47	-66.84	-39.93	-90.24
**AmB-DSPE3**	0.00	-65.45	-66.90	-37.35	-99.10
**AmB-DSPE4**	0.00	-65.16	-66.47	-40.94	-85.61
**AmB-DSPE5**	0.00	-55.15	-55.96	-33.60	-75.00
**AmB-DSPE6**	0.00	-48.18	-49.34	-24.00	-85.02
**AmB-DSPE7**	0.00	-29.15	-29.07	-15.63	-45.06
**AmB-DSPE8**	0.00	-27.00	-27.01	-7.17	-66.57
**AmB-DSPE9**	0.00	-16.61	-18.87	-3.52	-75.09
**AmB-DSPE10**	0.00	-11.32	-13.80	-8.24	-73.94
**AmB-DSPEc**					

**AmB-DSPE1c**	98.70	-54.55	-55.67	-32.20	-78.71
**AmB-DSPE2c**	1.23	-51.95	-52.85	-30.17	-76.06
**AmB-DSPE3c**	0.07	-50.23	-50.92	-29.98	-70.23
**AmB-DSPE4c**	0.00	-47.70	-48.40	-27.99	-68.44
**AmB-DSPE5c**	0.00	-44.19	-45.40	-21.56	-79.94
**AmB-DSPE6c**	0.00	-43.59	-44.53	-23.77	-69.61
**AmB-DSPE7c**	0.00	-43.59	-44.53	-23.77	-69.61
**AmB-Cho**					
**AmB-Chol1**	99.91	-39.27	-39.37	-20.76	-62.43
**AmB-Chol2**	0.08	-35.08	-34.95	-17.59	-58.23
**AmB-Chol3**	0.00	-32.05	-31.64	-15.62	-53.73
**AmB-Chol4**	0.00	-31.28	-30.75	-16.25	-48.65
**AmB-Chol5**	0.00	-28.40	-29.23	-8.78	-68.61
**AmB-Chol6**	0.00	-26.91	-27.55	-8.58	-63.62
**AmB-Chol7**	0.00	-22.86	-22.44	-7.88	-48.83
**AmB-Chol8**	0.00	-20.76	-21.21	-3.59	-59.09
**AmB-Chol9**	0.00	-19.51	-18.85	-6.90	-40.08
**AmB-Chol10**	0.00	-15.79	-16.81	2.58	-65.04
**AmB-Chol11**	0.00	-11.27	-10.65	-0.30	-34.71
**AmB-Chol12**	0.00	-6.77	-9.44	-2.56	-37.83
**AmB-Chol13**	0.00	-4.44	-7.95	-1.30	-32.37
**AmB-Chol14**	0.00	-4.00	-6.84	-0.92	-30.40
**AmB-Chol15**	0.00	-2.99	-3.94	-0.45	-28.90
**AmB-Chol16**	0.00	-1.93	-2.07	-0.98	-26.76
**AmB-Ret**					
**AmB-Ret1**	97.35	-33.51	-33.41	-15.88	-58.78
**AmB-Ret2**	2.60	-31.36	-31.14	-14.90	-54.45
**AmB-Ret3**	0.04	-28.88	-28.64	-13.73	-50.03
**AmB-Ret4**	0.01	-28.18	-28.13	-11.54	-55.64
**AmB-Ret5**	0.00	-27.46	-27.27	-11.26	-53.69
**AmB-Ret6**	0.00	-27.17	-26.99	-11.12	-53.23
**AmB-Ret7**	0.00	-26.04	-25.90	-10.01	-53.31
**AmB-Ret8**	0.00	-22.44	-22.18	-6.72	-51.85
**AmB-Ret9**	0.00	-20.60	-20.16	-7.76	-41.60
**AmB-Ret10**	0.00	-20.19	-19.94	-5.99	-46.79
**AmB-Ret11**	0.00	-19.76	-19.38	-5.65	-46.08
**AmB-Ret12**	0.00	-18.18	-17.60	-5.11	-41.89
**AmB-Ret13**	0.00	-17.60	-75.63	-3.01	-48.11
**AmB-Ret14**	0.00	-15.22	-1453	-1.89	-42.41
**AmB-Dod**					
**AmB-Dod1**	67.35	-26.71	-26.78	-11.01	-52.90

**AmB-Dod2**	13.86	-25.77	-25.99	-8.65	-58.17
**AmB-Dod3**	10.88	-25.63	-25.56	-9.43	-54.10
**AmB-Dod4**	4.90	-25.15	-25.27	-9.10	-54.22
**AmB-Dod5**	3.00	-24.86	-24.87	-8.39	-55.29
**AmB-Dod6**	0.02	-21.76	-22.85	-3.51	-64.88
**AmB-Dod7**	0.00	-19.92	-19.96	-3.87	-53.96
**AmB-Dod8**	0.00	-18.36	-18.20	-3.39	-49.68
**AmB-Dod9**	0.00	-17.88	-19.63	3.89	-78.91
**AmB-Dod10**	0.00	-17.26	-17.40	-1.82	-52.26
**AmB-Dod11**	0.00	-15.80	-15.53	-1.71	-46.38
**AmB-Dod12**	0.00	-12.32	-13.68	6.63	-68.14
**AmB-Dod13**	0.00	-9.62	-10.70	9.19	-66.73
**AmB-Dod14**	0.00	-7.14	-10.32	-7.99	-22.36
**AmB-Dod15**	0.00	-4.55	-6.31	-3.30	-13.76
**AmB-Dod16**	0.00	-4.14	-5.96	-1.01	-13.96

[a]Dimerization energy including ZPE and Counterpoise correction, [a b] in kcal/mol. [c] in cal/molK

Monomers

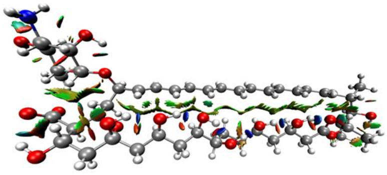

AmB

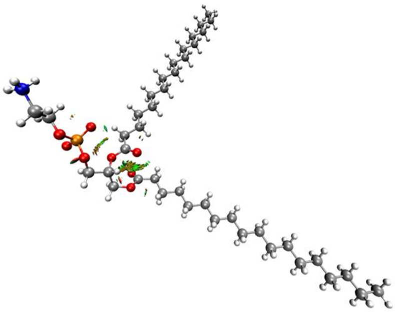

DSPE

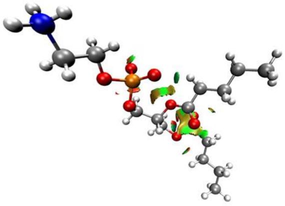

DSPEc

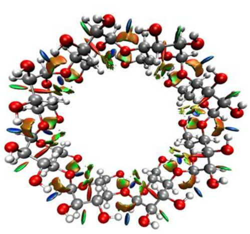

γ-Cyclodextrin

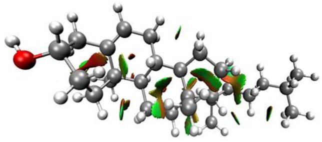

Cholesterol

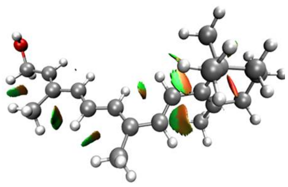

Retinol

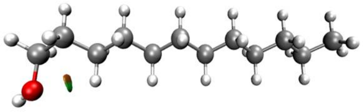

Dodecanol

Dimers

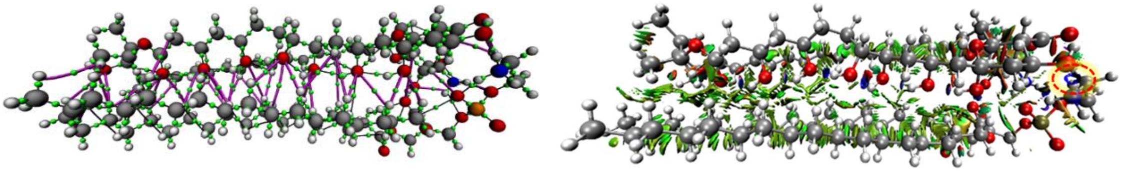

AmB-DSPE

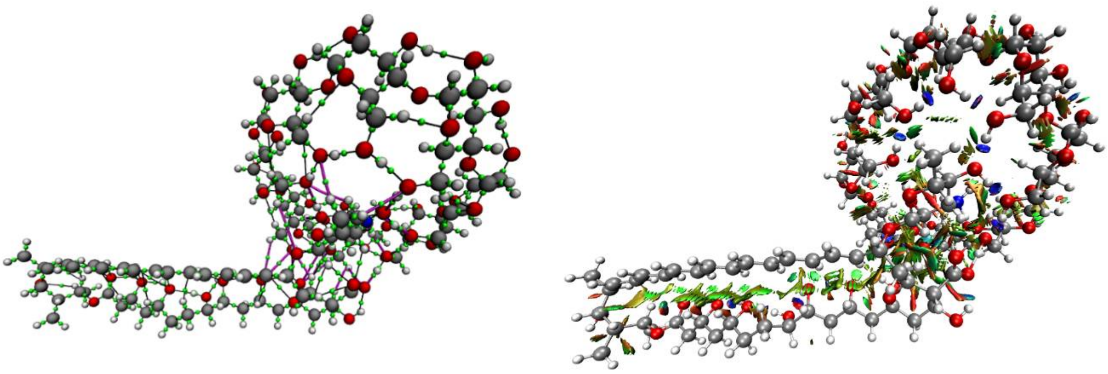

AmB-Cyc

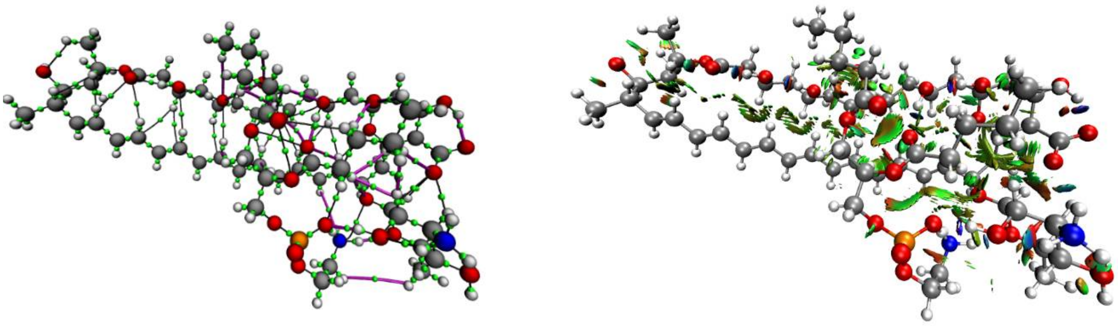

AmB-DSPEc

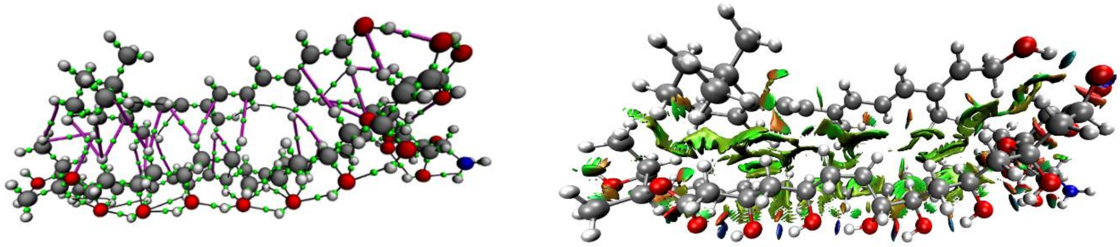

AmB-Ret

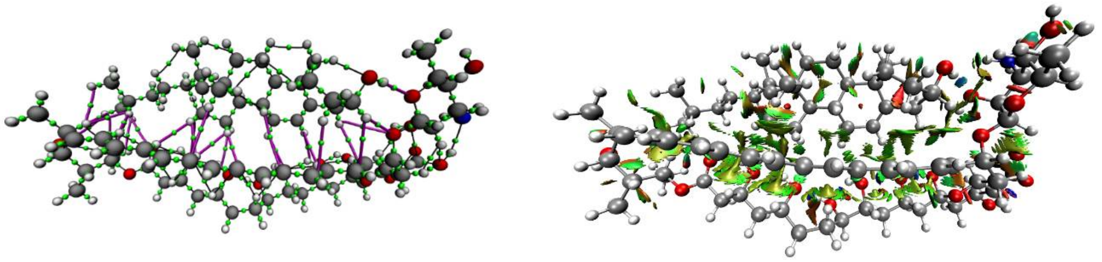

AmB-Cho

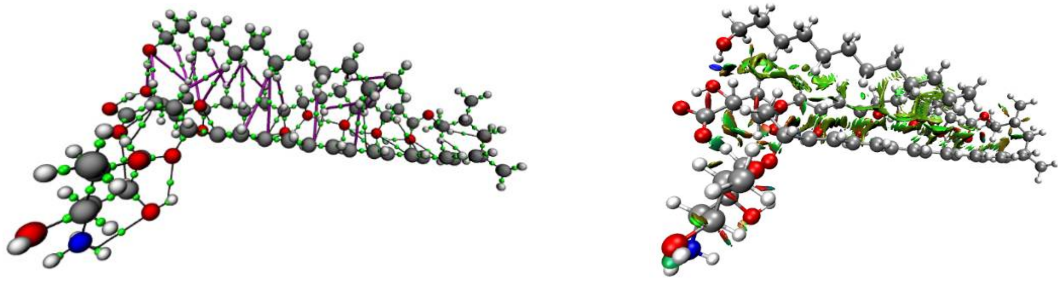

AmB-Dod

Figure S1. *Plots revealing noncovalent interactions at the monomers and the most stable dimer of each type including the pH effect. Monomer: 3D-NCI plots. Dimers: molecular graphs (left) where purple lines: bond path associated with intermolecular interactions. Black lines: bons paths associated with intramolecular interactions. 3D-NCI plots (right)*

Table S2. *Length and topological parameters (ranges) evaluated at bond critical points (CP) for intermolecular interactions for the most stable dimer of each type including the pH effect. CAM-B3LYP-D3/6-31+G(d,p)*

**Interaction type**	**Length[a] [Å]**	**$\rho(r_{CP})$[b] 10^{-2}**	**$\nabla^2\rho(r_{CP})$[c] 10^{-2}**	**$H(r_{CP})$[e] 10^{-4}**	**$\lvert V(r_{CP})\rvert/G(r_{CP})$[f]**	**IE[g] 10^{-2} [kcal/mol]**
**AmB-DSPE**						
O-H---O	1.58 - 1.81	3.28 - 6.47	10.40 - 16.30	-87.00 - 3.00	0.99 - 1.18	-1.26- -2.91
C-H---O	2.21 - 3.32	0.14 - 1.65	0.65 - 5.32	3.00 - 17.00	0.55 - 0.98	-0.01- -0.63
H---H	2.14 - 2.97	0.16 - 0.86	0.49 - 2.96	3.00 - 15.00	0.63 - 0.78	-0.03- -0.24
C-H---C	2.95 - 3.10	0.38 - 0.52	1.34 - 1.82	8.00 - 10.00	0.70 - 0.71	-0.09- -0.13
N-H---O	1.69 - 2.24	1.73 - 5.11	7.15 - 12.90	-38.00 - 17.00	0.89 - 1.11	-0.72- -2.00
**AmB-Cyc**						
O-H---O	1.34 - 2.55	0.72 - 11.70	1.47 - 12.40	-743.00 - 12.00	0.82 - 1.95	-0.65- -7.61
C-H---O	2.09 - 3.18	0.21 - 2.08	0.89 - 6.20	-5.00 - 12.00	0.62 - 1.03	-0.05- -0.82
H---H	2.06	1.01	3.66	16.00	0.79	-0.30
N-H---O	1.72 - 2.30	1.44 - 4.70	5.38 - 12.20	-28.00 - 10.00	0.92 - 1.08	-0.57- -1.81
C-H---N	3.39	0.19	0.69	5.00	0.63	-0.04
**AmB-DSPEc**						
O-H---O	1.64 - 1.82	3.13 - 5.12	10.40 - 15.00	-24.00 - 11.00	0.96 - 1.06	-1.21- -2.11
C-H---O	2.35 - 2.96	0.47 - 1.31	2.04 - 4.77	2.00 - 12.00	0.69 - 0.97	-0.14- -0.48

H---H	2.03 - 3.31	0.08 - 1.04	0.27 - 3.48	2.00 - 18.00	0.64 - 0.82	-0.06- -0.24
C-H---C	2.62 - 3.63	0.13 - 0.78	0.420 - 2.67	3.00 - 13.00	0.68 - 0.76	-0.03- -0.21
O---O	3.10	0.66	2.74	9.00	0.86	-0.26
N-H---O	1.88	3.01	8.66	8.00	1.03	-1.17
N---O	2.77	1.47	6.04	18.00	0.86	-0.57
**AmB-Ret**						
O-H---O	1.98	2.55	6.21	-18.00	1.10	-0.95
C-H---O	2.24 - 2.62	0.78 - 1.68	2.62 - 5.19	-1.00 - 7.00	0.88 - 1.01	-0.25- -0.62
H---H	2.09 - 2.68	0.31 - 1.08	1.10 - 3.81	7.00 - 17.00	0.66 - 0.78	-0.07- -0.31
C-H---C	2.67 - 3.37	0.24 - 0.80	0.75 - 2.57	4.00 - 14.00	0.69 - 0.79	-0.05- -0.18
**AmB-Col**						
O-H---O	1.96	2.52	7.11	-10.00	1.05	-0.98
C-H---O	2.56 - 3.16	0.24 - 0.94	1.00 - 3.19	7.00 - 13.00	0.63 - 0.89	-0.05 - -0.33
H---H	2.04 - 2.61	0.41 - 0.94	1.38 - 3.53	8.00 - 16.00	0.67 - 0.79	-0.09- -0.29
C-H---C	2.73 - 3.51	0.18 - 0.80	0.56 - 2.56	3.00 - 14.00	0.69 - 0.78	-0.03- -0.20
**AmB-Dod**						
O-H---O	1.75	4.23	11.43	18.00	1.06	-1.61
C-H---O	2.31 - 3.03	0.34 - 1.39	1.40 - 4.46	4.00 - 11.00	0.69 - 0.96	-0.09- -0.51
H---H	2.16 - 2.79	0.30 - 0.80	1.03 - 2.98	6.00 - 16.00	0.68 - 0.78	-0.06- -0.21
C-H---C	2.73 - 3.47	0.24 - 0.81	0.77 - 2.55	4.00 - 14.00	0.70 - 0.78	-0.05- -0.21

[a] Euclidian distance between the attractors, [b] Electron density, [c] Electron density laplacian, [d] Bond ellipticity,[e] Total energy density, [f] Virial Field/ Kinetic energy density [g] Interaction energy.

Table S3. *NBO Donor - acceptor second-order interaction energies* $E^{(2)}_{i\rightarrow j}$ *in kcal/mol for each interaction type of AmB-bioconjugate dimers including the pH effect. CAM-B3LYP-D3/6-31+G(d,p)*

	Amb-Dod			AmB-Ret			AmB-Cho			AmB_DSPEc			AmB-Cyc			AmB_DSPE		
**Type**	**Donor**	**Acceptor**	$E^{(2)}_{i\rightarrow j}$	**Donor**	**Acceptor**	$E^{(2)}_{i\rightarrow j}$	**Donor**	**Acceptor**	$E^{(2)}_{i\rightarrow j}$	**Donor**	**Acceptor**	$E^{(2)}_{i\rightarrow j}$	**Donor**	**Acceptor**	$E^{(2)}_{i\rightarrow j}$	**Donor**	**Acceptor**	$E^{(2)}_{i\rightarrow j}$
C-H---O	LP (O_{38})	H_{131}-C_{130})	2.27	LP (O_{50})	BD*(H_{139}- C_{137})		LP (O_{28})	BD*(H_{78} -C_{77})	2.40	LP (O_{118})	BD*(H_{148}-C_{146})	2.22	LP (O_{74})	BD* (H_{178}-C_{177})	4.85	LP (O_{114})	BD*(H_{203}-C_{201})	2.30
				LP (O_{167})	BD* (H_{46}-C_{43})	2.06				LP(O_{114})	BD*(H_{172}-C_{170})	1.65	BD* (O_{53} - H_{63})	BD* (C_{259} - C_{263})	2.32	LP (O_{117})	BD*(H_{263}-C_{262})	1.99
				LP (O_{167})	BD* (H_{49}-C_{47})	1.73				LP (O_{114})	BD*(H_{166}-C_{164})	1.47	LP (O_{137})	BD* (H_{180}-C_{179})	2.29	LP (O_{122})	BD*(H_{220}-C_{219})	1.85
										LP (O_{122})	BD*(H_{142}-C_{140})	1.17	LP (O_{11})	BD* (H_{181}-C_{179})	1.39	LP (O_{108})	BD*(H_{195}-C_{194})	1.79
																LP (O_{128})	BD*(H_{154}-C_{152})	1.39
																BD (C_{95} - O_{117})	BD*(H_{263}- C_{262})	1.23
																LP (O_{120})	BD*(H_{171}-C_{170})	1.22
																LP (O_{126})	BD*(H_{160}-C_{158})	1.13
O-H---O	LP (O_{172})	BD*(H_{39} - O_{38}) O-H—O	27.18	LP (O_{184})	BD* (H_{51}-O_{50})	13.20	LP (O_{182})	BD* (H_{74}-O_{28})	11.30	LP (O_{178})	BD* (H_{109}-O_{108})	28.08	LP (O_{278})	BD* (H_{21}- O_{11})	25.70	LP (O_{199})	BD*(H_{109}-O_{108})	5.09
										LP (O_{174})	BD* (H_{119}-O_{118})	14.26	LP (O_{32})	BD* (H_{279}-O_{278})	23.38			
													LP (O_{116})	BD* (H_{277}-O_{276})	22.93			
													LP (O_{286})	BD* (H_{63}-O_{53})	15.15			
													LP (O_{281})	BD* (H_{105}- O_{95})	12.46			
													BD (C_{263} - O_{286})	BD* (H_{63}- O_{53})	4.98			
													LP (O_{95})	BD* (H_{283}-O_{282})	3.92			
													BD* (O_{53} - H_{63})	BD* (C_{263} - O_{286})	2.95			
C-H---C				BD (C_{80}-C_{82})	BD* (H_{33}-C_{32})	1.34				BD (C_{167} - H_{169})	BD* (H_{10}-C_{9})	1.45						
N-H--O										LP (O_{112})	BD*(H_{186}-N_{185})	10.33	LP (O_{32})	BD* (H_{304}-N_{303})	1.09	LP (O_{132})	BD*(H_{267}-N_{265})	31.37
										BD* (N_{185} - H_{192})	BD* (H_{109}-O_{108})	1.66				LP (O_{256})	BD*(H_{135}-N_{134})	30.55
													LP (O_{32})	BD* (H_{304}-N_{303})	2.40	BD (O_{132} - H_{133})	BD*(H_{267}-N_{265})	3.43
																LP (O_{255})	BD*(H_{271}-N_{134})	1.21
													LP (O_{53})	BD* (H_{306}-N_{303})	25.10			

Molecular structures with CAM-B3LYP/ 6-31G(d,p)

AmB-Cyc

AmB-Cyc1

0 1

C	-9.52017500	5.02018900	-1.94736600
C	-10.84501200	4.60196400	-1.32282600
C	-10.79224900	3.17137300	-0.82275400
C	-10.25495800	2.24911100	-1.90911800
C	-8.90360900	2.77187400	-2.38776900
C	-8.23503300	1.95601200	-3.47468500
O	-8.56396000	5.13078300	-0.93767500
O	-11.15466600	5.49821400	-0.27231300
O	-12.09986500	2.84825100	-0.42410500
O	-9.12149200	4.08532300	-2.91645900
O	-6.85982000	2.27808900	-3.58097600
H	-10.95682700	2.23400500	-2.75302500
H	-9.63618000	5.97232700	-2.47521600
H	-11.61223800	4.65033100	-2.10792900
H	-10.08969700	3.12534200	0.02374800
H	-8.21712800	2.82332800	-1.53455600
H	-8.37016500	0.89088800	-3.25986600
H	-8.72123700	2.17575800	-4.43089600
H	-11.90755900	5.10918200	0.19479900
H	-12.14586400	1.89907500	-0.20021400

H	-6.34285600	1.59498100	-3.09557300
C	-10.83479800	-0.09911800	-1.94013800
C	-11.65020100	-0.82869500	-0.87649400
C	-10.73481100	-1.51720400	0.11318400
C	-9.78110000	-2.41406100	-0.65185300
C	-9.00233800	-1.61505200	-1.69525000
C	-8.07436300	-2.47058700	-2.55945600
O	-10.14035500	0.95461800	-1.33818800
O	-12.51043600	0.08406000	-0.22246300
O	-11.56451400	-2.23537600	0.99209000
O	-9.95357400	-0.99526400	-2.57207100
O	-6.71985200	-2.17982100	-2.25908000
H	-10.37143400	-3.17511200	-1.17958300
H	-11.49298500	0.27406100	-2.73081400
H	-12.24188600	-1.60160500	-1.38578200
H	-10.14739000	-0.75334500	0.64384500
H	-8.39664500	-0.84593700	-1.20208800
H	-8.26510500	-3.53505800	-2.39360900
H	-8.28810000	-2.23852200	-3.60844400
H	-12.82459400	-0.37078600	0.57261400
H	-11.01550300	-2.78991400	1.57872000
H	-6.20768900	-2.99882500	-2.10010800
C	-8.92956600	-4.43106400	0.33801500
C	-9.04308300	-4.89409700	1.79056200
C	-7.77338900	-4.60765600	2.56602000
C	-6.58930900	-5.18156400	1.80881000

C	-6.56221600	-4.58767100	0.40541200
C	-5.39364900	-5.06136600	-0.43378900
O	-8.92262500	-3.03289500	0.28924900
O	-10.16328800	-4.27099200	2.38969500
O	-7.92992300	-5.17814100	3.84035500
O	-7.77424000	-4.95793200	-0.26248400
O	-4.96328500	-4.04807800	-1.34345900
H	-6.69700700	-6.27199800	1.73713300
H	-9.77073400	-4.82806300	-0.23847500
H	-9.18277600	-5.98394900	1.77270400
H	-7.63827400	-3.51676000	2.62796500
H	-6.51083400	-3.49619500	0.49138400
H	-4.56211700	-5.36120900	0.20807200
H	-5.70514000	-5.92497600	-1.02628400
H	-10.08095400	-4.42493300	3.34161000
H	-7.08752100	-5.09892100	4.32882600
H	-4.68431300	-3.23689000	-0.80278700
C	-4.59581900	-5.88888300	2.96593500
C	-4.20435300	-5.61190700	4.41056500
C	-3.28876200	-4.40819100	4.49646300
C	-2.12360300	-4.59087900	3.53681200
C	-2.62835000	-4.83978600	2.11827300
C	-1.54362900	-5.07468400	1.06247700
O	-5.42246200	-4.84282000	2.52713500
O	-5.38046300	-5.42678200	5.17950100
O	-2.88019900	-4.30916500	5.83820400

O	-3.45714300	-6.01679300	2.15901900
O	-1.37832200	-3.93060600	0.26221400
H	-1.53772800	-5.46270500	3.85887700
H	-5.12399400	-6.84514400	2.89492300
H	-3.64685100	-6.48441800	4.77676100
H	-3.85039100	-3.51552100	4.18137700
H	-3.22272900	-3.98127900	1.78590200
H	-0.59425100	-5.37249400	1.51973100
H	-1.88418600	-5.90927000	0.43797500
H	-5.09320100	-5.05227800	6.02408200
H	-2.08636600	-3.74290700	5.89306700
H	-0.48224600	-3.55616300	0.35771400
C	0.04283900	-3.54775800	3.78847500
C	0.46591900	-2.72723600	5.00466100
C	0.25973700	-1.25124700	4.74429500
C	0.94508900	-0.85892000	3.45255800
C	0.50426000	-1.74963500	2.29754200
C	1.29887600	-1.49866100	1.03678300
O	-1.34212900	-3.41655300	3.62327600
O	-0.27914100	-3.15361100	6.13365900
O	0.77774200	-0.54758400	5.85033700
O	0.73493100	-3.12103300	2.65133400
O	0.76810800	-2.25642100	-0.02825700
H	2.03130300	-0.94839900	3.59482900
H	0.31387500	-4.59874000	3.93152300
H	1.53709600	-2.90266600	5.17060100

H	-0.81795200	-1.06771600	4.62375400
H	-0.55810600	-1.58699700	2.08372100
H	1.27658900	-0.43389600	0.79510200
H	2.33771300	-1.81123700	1.19875600
H	-0.11658400	-2.49701700	6.82567800
H	0.96002600	0.36867300	5.57294200
H	0.32933800	-1.65304400	-0.65917100
C	1.53672800	1.35346800	2.67947300
C	1.70512700	2.54346400	3.62237500
C	0.46497000	3.41035100	3.60737200
C	0.09666500	3.73286300	2.16898100
C	-0.13684300	2.44009600	1.39045100
C	-0.59271900	2.61017600	-0.05682900
O	0.58779500	0.49390300	3.23868000
O	1.97842900	2.05395400	4.92392300
O	0.76106300	4.56539900	4.35794400
O	1.13613700	1.77167500	1.39912100
O	-1.99310900	2.35471800	-0.10694400
H	0.93967000	4.24554600	1.68811400
H	2.49333000	0.83972300	2.54167100
H	2.54484600	3.15108400	3.25802100
H	-0.36511100	2.84447100	4.05548000
H	-0.88194100	1.81332700	1.89048300
H	-0.37456700	3.62151800	-0.41666600
H	-0.05129400	1.88674000	-0.67805300
H	1.91904300	2.82196600	5.50959600

H	-0.01739300	5.15201000	4.35775500
H	-2.42992000	2.92740700	-0.76765000
C	-0.80772400	5.89080600	1.75101600
C	-1.59786700	6.85945500	2.62725700
C	-3.08721000	6.69718600	2.40252700
C	-3.36820000	6.76046100	0.91069000
C	-2.56369200	5.68132700	0.19472400
C	-2.81742000	5.54346800	-1.30178200
O	-1.02627800	4.58980300	2.20987900
O	-1.23914900	6.64411600	3.97894700
O	-3.73558000	7.72572000	3.11339000
O	-1.18184100	6.01276500	0.40202900
O	-3.41587900	4.27870200	-1.53972900
H	-3.05047500	7.73828100	0.52557900
H	0.25625300	6.14218500	1.79501600
H	-1.32414500	7.87810100	2.31841800
H	-3.39154900	5.70480100	2.76685600
H	-2.77205600	4.70681200	0.64565900
H	-3.46942100	6.34289200	-1.66576400
H	-1.85306700	5.62071700	-1.81826100
H	-1.88792000	7.11843300	4.51617100
H	-4.68597000	7.69969100	2.90012900
H	-4.14546200	4.36868400	-2.19565100
C	-5.43929900	7.62412500	0.07143100
C	-6.77488900	7.86286200	0.76643200
C	-7.68752800	6.66837800	0.58297400

C	-7.76875400	6.30112300	-0.88892700
C	-6.37465100	6.07535900	-1.46650400
C	-6.32865700	5.73090500	-2.95718100
O	-4.76525800	6.60708000	0.74813800
O	-6.52860400	8.13171200	2.13377000
O	-8.94739100	7.01340100	1.10836700
O	-5.63771300	7.29377000	-1.28065200
O	-5.49241600	4.61739600	-3.22701000
H	-8.26131700	7.11932700	-1.43164900
H	-4.84923000	8.54608000	0.07032000
H	-7.25000400	8.72841400	0.28442900
H	-7.25015100	5.81278000	1.11892200
H	-5.88887700	5.26355200	-0.91436600
H	-7.32965200	5.52664300	-3.34118000
H	-5.92657600	6.60085700	-3.48471600
H	-7.38437700	8.08985000	2.58163300
H	-9.60258700	6.36373500	0.79510800
H	-6.04985500	3.81440300	-3.33953800
C	-3.78858500	-1.14186800	-1.49497000
H	-3.96140700	-1.85612900	-2.31223200
C	-4.16342300	0.23852800	-2.03773100
H	-3.99437400	1.01126300	-1.27939900
C	-3.26092900	0.53730700	-3.25044800
H	-3.43520300	-0.25978200	-3.99312900
C	-1.54197300	-0.81794700	-2.41698400
H	-1.81737500	-1.51783500	-3.20910900

C	-2.29842900	-1.20880500	-1.16058800
H	-2.05473900	-2.23916300	-0.89325200
C	-3.48630000	1.88418200	-3.89644200
H	-3.25477900	2.68588000	-3.19525700
H	-4.51570800	1.98710900	-4.24209600
H	-2.82271700	1.98627400	-4.75774300
C	0.69331100	-1.20061700	-3.27256000
H	0.57025000	-0.42409000	-4.03699300
C	2.07350100	-1.14513600	-2.69673200
H	2.29177300	-1.87194500	-1.91918500
C	3.00698700	-0.26034100	-3.05861800
H	2.79803700	0.47317200	-3.83694200
C	4.30940000	-0.21976500	-2.42444900
H	4.47610300	-0.94060100	-1.62755600
C	5.29805700	0.64298000	-2.72463500
H	5.15483100	1.37673400	-3.51735700
C	6.55816100	0.64340000	-2.02042300
H	6.65840500	-0.10447300	-1.23596300
C	7.59169400	1.47285000	-2.26753100
H	7.50838100	2.22240000	-3.05391300
C	8.82734700	1.41900500	-1.52625600
H	8.90330500	0.65417000	-0.75345300
C	9.88591600	2.22926200	-1.73147700
H	9.83458300	2.98792200	-2.51174300
C	11.09816500	2.16292600	-0.95426600
H	11.13307700	1.41818500	-0.15924600

C	12.17509000	2.95082600	-1.14792900
H	12.16020600	3.68355100	-1.95405000
C	13.36534400	2.88629900	-0.33495900
H	13.34249700	2.20065100	0.51033200
C	14.49015400	3.59100000	-0.55600400
H	14.54422500	4.26173000	-1.41276300
C	0.33900400	-2.56730200	-3.88598200
H	-0.62092300	-2.50738500	-4.39640200
H	1.09100400	-2.79652300	-4.64676700
C	15.67039300	3.46981600	0.27753000
H	15.54751500	2.88013000	1.18387200
C	16.87451600	3.97253500	-0.01581600
H	17.00099100	4.54916700	-0.93364900
C	18.11235400	3.76630900	0.80984500
H	17.83121800	3.30502400	1.76345800
C	19.09402900	2.80440800	0.11244100
H	19.48995200	3.32685600	-0.77433900
C	18.46796000	1.49267100	-0.39682900
H	17.67790000	1.79077700	-1.09292800
C	17.75851500	0.69303500	0.70122400
H	17.13837200	1.35666100	1.29724800
C	15.58171100	-0.26466800	0.37606400
C	14.77617500	-1.23857700	-0.44642200
H	15.26195600	-1.38669000	-1.41268000
H	14.78635200	-2.19838500	0.08315200
C	13.32439900	-0.78659800	-0.62193500

H	13.30863900	0.17989800	-1.14572000
C	12.52662200	-1.80645300	-1.42145100
H	12.98986800	-1.91896000	-2.40754600
H	12.58293400	-2.77748200	-0.91497400
C	11.05465300	-1.44538900	-1.60625800
H	10.97561900	-0.43609500	-2.03284100
C	10.35830000	-2.43126600	-2.53861000
H	10.62000700	-3.45175600	-2.23260200
H	10.77800800	-2.28780900	-3.53900100
C	8.82896700	-2.27721500	-2.57739200
H	8.53695800	-1.27953300	-2.24096100
H	8.47176300	-2.36617500	-3.60795600
C	8.08377500	-3.32941100	-1.75823300
H	8.27100000	-4.31260700	-2.21235900
C	6.55491500	-3.07672400	-1.75244800
H	6.27430700	-2.48433300	-2.63121400
C	5.74159900	-4.36595600	-1.73820400
H	6.00459300	-4.94955200	-0.84757500
H	6.01612800	-4.96166900	-2.61665400
C	4.23545500	-4.11563800	-1.76202500
H	4.00023600	-3.46531700	-2.61643200
C	3.45206800	-5.41163300	-1.92812200
H	3.71253100	-6.10886800	-1.12622000
H	3.73778200	-5.86479500	-2.88253200
C	1.93481500	-5.22550000	-1.90064900
C	1.16782200	-6.45318700	-2.37356100

H	1.34548800	-7.29666200	-1.70257100
H	1.50701900	-6.72875900	-3.37639700
C	-0.31540500	-6.13991600	-2.40082800
H	-0.63024000	-5.91106500	-1.38028600
C	-0.61205700	-4.90235900	-3.26434500
H	-0.44814100	-5.16611900	-4.31619200
C	0.32341700	-3.72498700	-2.88616600
H	0.00927700	-3.38037300	-1.90005000
C	-2.06192000	-4.46421700	-3.12237600
C	18.63682000	-0.13658700	1.61518900
H	18.03026400	-0.60498100	2.39366400
H	19.37958000	0.51361500	2.07942900
H	19.15187500	-0.92350000	1.05999900
C	19.48275400	0.65395200	-1.17204200
H	20.34052100	0.37390100	-0.55304900
H	19.85741500	1.20531500	-2.03931100
H	19.02495900	-0.26989300	-1.53262500
C	18.80752900	5.09648100	1.11354400
H	19.08123900	5.60940900	0.18482200
H	19.71713100	4.92741700	1.69140800
H	18.14547200	5.75763600	1.67725800
O	-1.96683700	-0.42412500	-0.03631500
H	-2.04743100	0.53246900	-0.23934400
O	-5.54969700	0.28198500	-2.36754200
H	-5.89520800	-0.61672800	-2.55264200
O	-1.89807500	0.49222300	-2.82091300

O	-0.17552400	-0.86840500	-2.16037100
O	1.47341400	-4.93109300	-0.61531700
H	1.93939500	-4.13178800	-0.31452000
O	1.67243600	-4.16388900	-2.81235700
O	-2.54199500	-3.49442200	-3.68032500
O	-2.78867200	-5.21849800	-2.29999200
H	-3.64651300	-4.74626800	-2.11441100
O	3.80524300	-3.47491500	-0.56076200
H	4.52439500	-2.87881300	-0.28863600
O	6.24271300	-2.27716500	-0.61025400
H	6.94496900	-2.52947100	0.02470100
O	8.50084900	-3.39201500	-0.40171900
H	9.21249000	-2.73394500	-0.25103100
O	10.36476000	-1.45712900	-0.35330700
H	10.97917800	-1.12034900	0.32412100
O	12.68999800	-0.64341700	0.64629900
H	13.29291400	-0.12795900	1.21130600
O	15.10585100	0.38636400	1.29276000
O	16.85182500	-0.21695600	0.00240200
O	20.14958900	2.57047300	1.03996800
H	20.90978400	2.22241600	0.56189900
O	-0.99537700	-7.27965300	-2.89793500
H	-1.92804100	-7.14404600	-2.68869500
N	-4.54661100	-1.59787000	-0.32636400
H	-5.49320300	-1.22808400	-0.35652500
H	-4.07939300	-1.24568100	0.50728000

AmB-Cyc2

0 1

C	-9.90649500	-0.51710400	-1.50897700
C	-10.74213300	-1.01477200	-0.33525700
C	-9.90686000	-1.90434100	0.55627600
C	-9.27044800	-2.99998600	-0.28037500
C	-8.46426500	-2.40095900	-1.43281200
C	-7.83775000	-3.42674300	-2.37630200
O	-8.89940300	0.32466700	-1.02037600
O	-11.25887900	0.09586600	0.37619700
O	-10.75933500	-2.42159400	1.55062600
O	-9.36271300	-1.59690400	-2.21616400
O	-6.48008400	-3.11757100	-2.65373800
H	-10.06428600	-3.64376300	-0.68319000
H	-10.54705300	0.01864800	-2.21596800
H	-11.56451800	-1.61609000	-0.74600400
H	-9.09879900	-1.30031500	0.99398900
H	-7.66459700	-1.76817600	-1.03425100
H	-7.90213500	-4.43395900	-1.95681300
H	-8.39368700	-3.40356300	-3.31674800
H	-11.55602300	-0.23741400	1.23391000
H	-10.29211200	-3.13514600	2.02262300
H	-5.90267400	-3.61391800	-2.03202500

C	-8.53913100	-5.12549300	0.60536000
C	-8.64720700	-5.62840500	2.04273200
C	-7.34495000	-5.40016700	2.77790500
C	-6.16827900	-5.95630800	1.98763000
C	-6.16509400	-5.32549600	0.58891000
C	-5.07091500	-5.75713300	-0.37686100
O	-8.45680300	-3.73417600	0.61891200
O	-9.72383900	-4.96336000	2.67753500
O	-7.45581500	-6.01092400	4.04231900
O	-7.41497300	-5.68587000	-0.02184100
O	-4.71366400	-4.70491400	-1.27371900
H	-6.26700900	-7.04665600	1.89747100
H	-9.40563800	-5.45710400	0.02403400
H	-8.83325200	-6.71047200	2.00457400
H	-7.19200300	-4.31479100	2.87510800
H	-6.10534300	-4.23659200	0.70020600
H	-4.19261200	-6.09771700	0.16904900
H	-5.45067900	-6.58799400	-0.97665000
H	-9.64639600	-5.15999100	3.62140200
H	-6.58258800	-5.96655100	4.46818600
H	-4.00665300	-4.15929200	-0.84853600
C	-4.01586000	-6.56409700	2.90335100
C	-3.39309200	-6.44930200	4.29566200
C	-2.54292400	-5.19480600	4.44994300
C	-1.63152600	-5.02034100	3.24571600
C	-2.48996800	-5.02919500	1.99166100

C	-1.82717500	-4.67653000	0.67071700
O	-5.04603100	-5.62854900	2.78683700
O	-4.43185700	-6.50394300	5.25307400
O	-1.81637100	-5.32577200	5.64953300
O	-3.03814700	-6.35802000	1.91803500
O	-2.58062300	-3.64568500	0.03985300
H	-0.92114200	-5.85632500	3.20675000
H	-4.40109400	-7.57788900	2.74822500
H	-2.71504100	-7.30728200	4.40800600
H	-3.21494100	-4.32338400	4.48619900
H	-3.30154900	-4.30832600	2.12151200
H	-0.80007000	-4.34855600	0.79552800
H	-1.81144200	-5.56454600	0.02962300
H	-4.02261400	-6.33262000	6.11176800
H	-1.07189700	-4.69866900	5.62132200
H	-2.01715700	-2.85397600	-0.09009500
C	0.39623300	-3.74969100	3.03973700
C	1.22440400	-3.18292100	4.18493000
C	0.94857600	-1.70608800	4.37473200
C	1.07502900	-0.96777200	3.04761100
C	0.13777800	-1.60551900	2.02586500
C	0.16604600	-0.97647600	0.64583900
O	-0.94300500	-3.80131200	3.44264700
O	0.93655300	-3.91176900	5.36309000
O	1.86590900	-1.25690900	5.34300400
O	0.55725400	-2.97040100	1.88376400

O	-1.00941200	-1.28914200	-0.08366000
H	2.10384400	-1.05306100	2.67439100
H	0.75289000	-4.74992600	2.77473600
H	2.28412200	-3.29156700	3.91397000
H	-0.08893300	-1.58756300	4.72096700
H	-0.89287500	-1.57070400	2.39658300
H	0.27182600	0.10698700	0.75355000
H	1.04363900	-1.34979900	0.10659000
H	1.34165900	-3.40669700	6.08265800
H	1.65668900	-0.33279100	5.57879300
H	-1.50525100	-0.45346900	-0.21216600
C	1.80677700	1.30291100	3.28893000
C	1.66281200	2.22915500	4.49246000
C	0.44182800	3.10757500	4.33602800
C	0.52728400	3.83905500	3.00744200
C	0.66034100	2.83475600	1.86317200
C	0.82352300	3.45326900	0.47995900
O	0.74382100	0.39195000	3.27379200
O	1.59068200	1.47286600	5.68608700
O	0.43259300	3.98218500	5.43954000
O	1.84083400	2.05847700	2.10573800
O	-0.43875000	3.55483400	-0.15134800
H	1.42495800	4.47130200	3.00594400
H	2.76367300	0.77499100	3.33528800
H	2.54905600	2.87818400	4.51267700
H	-0.45309500	2.46854800	4.32231000

H	-0.22138600	2.18261500	1.84138500
H	1.29228400	4.44122300	0.54936100
H	1.48718000	2.79808500	-0.09692200
H	1.23305500	2.06905500	6.35957500
H	-0.35105200	4.56206100	5.38523000
H	-0.53612100	4.45945800	-0.53339600
C	-0.46418400	6.02776100	2.95704200
C	-1.55342500	6.61388800	3.85253400
C	-2.90971200	6.40931200	3.21514600
C	-2.91872200	6.96400900	1.79924300
C	-1.76493500	6.35562500	1.00638000
C	-1.62595100	6.88409100	-0.40367600
O	-0.63258900	4.64004600	2.88109400
O	-1.50027000	6.00682300	5.13054600
O	-3.86682000	7.02452900	4.04494300
O	-0.53257400	6.61716300	1.68681400
O	-0.85684300	6.00127500	-1.20473200
H	-2.84373300	8.05929300	1.82389600
H	0.52374900	6.27507800	3.35726700
H	-1.36422700	7.69268300	3.93763600
H	-3.08781200	5.32682400	3.13937200
H	-1.93353100	5.27989000	0.95059900
H	-2.62560900	7.02231400	-0.82546000
H	-1.13173700	7.86210800	-0.36406300
H	-2.35241000	6.18962200	5.55137800
H	-4.75224100	6.86477700	3.66710000

H	-1.44818500	5.65202400	-1.91318800
C	-5.10213500	7.48563500	0.83040800
C	-6.45617500	6.98823100	1.32429900
C	-6.73843500	5.61798900	0.72146300
C	-6.64344300	5.70241300	-0.79142800
C	-5.31717800	6.33793800	-1.21904900
C	-5.25978800	6.60317800	-2.71354200
O	-4.15077900	6.55352200	1.23189700
O	-6.44292200	6.91293000	2.73751000
O	-7.97974100	5.13399900	1.18885600
O	-5.14457500	7.60123100	-0.57481600
O	-5.03054500	5.40394000	-3.45349100
H	-7.47782900	6.29844400	-1.17808000
H	-4.87696200	8.48527600	1.21557000
H	-7.22409000	7.69823400	0.98703000
H	-5.95950200	4.93518800	1.07524600
H	-4.50407400	5.65207500	-0.94910500
H	-6.17795100	7.09387700	-3.06081100
H	-4.42350400	7.27495700	-2.92094600
H	-7.18862200	6.34490600	2.98115100
H	-8.62994400	5.21255500	0.46744000
H	-5.45463900	4.66297500	-2.99545800
C	-7.60521500	4.06828700	-2.34637300
C	-9.05334500	3.90596300	-1.88486600
C	-9.23785200	2.66906200	-1.03962400
C	-8.65670300	1.48165600	-1.78668900

C	-7.17710000	1.72894300	-2.06354500
C	-6.44364800	0.58277200	-2.75219100
O	-6.69720300	4.37172200	-1.30693400
O	-9.50737700	5.05945300	-1.18464600
O	-10.62594100	2.55351000	-0.82580000
O	-7.13463900	2.88860000	-2.93116500
O	-5.91035500	-0.23688200	-1.73113900
H	-9.14991700	1.39444500	-2.76385200
H	-7.55801700	4.83838700	-3.12653000
H	-9.65196100	3.77835900	-2.79700700
H	-8.69411800	2.79819200	-0.09265500
H	-6.64262700	1.94823400	-1.13384100
H	-7.13465000	0.02073200	-3.39532100
H	-5.64975400	1.00903200	-3.38080700
H	-10.40257100	4.82451300	-0.89267300
H	-10.80105600	1.78788200	-0.24504500
H	-5.50852100	-1.03208500	-2.12260000
C	-3.41156400	2.86155400	-1.64083400
H	-4.41679000	2.87137800	-2.07822700
C	-2.44214900	3.40741600	-2.69633400
H	-1.41575500	3.18324500	-2.39689700
C	-2.76543500	2.71092800	-4.02023700
H	-3.71883600	3.12870700	-4.37249700
C	-2.51268000	0.70321600	-2.67489700
H	-2.87870100	-0.31920300	-2.71962700
C	-3.04197400	1.37784800	-1.38422600

H	-3.93839900	0.83700300	-1.07467000
C	-1.71118900	2.96168700	-5.08158700
H	-0.71750300	2.69631600	-4.71629100
H	-1.70232900	4.02270900	-5.33908400
H	-1.92848100	2.37696600	-5.97812300
C	-0.34595700	0.08463900	-3.64122800
H	-0.20705000	0.79883700	-4.45951300
C	0.97559900	-0.16689000	-2.98087500
H	1.01291900	-0.98026500	-2.26397000
C	2.05019600	0.60332900	-3.17711900
H	2.00092800	1.42846100	-3.88774600
C	3.29938800	0.41080700	-2.46854100
H	3.34135900	-0.43686200	-1.78858500
C	4.38400800	1.19986000	-2.59093000
H	4.35199700	2.05704700	-3.26298800
C	5.60936400	0.95333000	-1.87001800
H	5.61816800	0.06542600	-1.24126200
C	6.72603700	1.70477100	-1.94795200
H	6.73820100	2.59529300	-2.57551000
C	7.93485500	1.37319800	-1.23542300
H	7.92171100	0.45237100	-0.65234600
C	9.07222200	2.09762400	-1.26077000
H	9.10911300	3.01438300	-1.84821600
C	10.26057900	1.73065400	-0.53222700
H	10.21466100	0.81116100	0.05127000
C	11.40890500	2.43722600	-0.53255400

H	11.47343400	3.34749900	-1.12767600
C	12.57712300	2.05725300	0.22401300
H	12.48353100	1.17554100	0.85587000
C	13.76085100	2.69613600	0.18831200
H	13.88220300	3.56567400	-0.45670600
C	-1.00846200	-1.15519500	-4.24956700
H	-2.00638400	-0.87731300	-4.59853500
H	-0.43356900	-1.42650900	-5.14050000
C	14.91756700	2.25351300	0.94197000
H	14.73924700	1.44328500	1.64623400
C	16.16070800	2.72193300	0.78730400
H	16.34156100	3.52311400	0.06870700
C	17.37090200	2.19013200	1.50052200
H	17.04609100	1.48979300	2.27870500
C	18.28370900	1.40659900	0.53656600
H	18.73229900	2.13756500	-0.15673500
C	17.56364200	0.36218300	-0.33615100
H	16.80460100	0.91559700	-0.89754100
C	16.78612300	-0.67463200	0.48132400
H	16.21945400	-0.17624400	1.26275400
C	14.54136300	-1.31956900	-0.06499200
C	13.65903300	-1.94448400	-1.11640700
H	14.12631000	-1.83222000	-2.09656800
H	13.60061800	-3.01664100	-0.89528300
C	12.24416600	-1.36015100	-1.10827400
H	12.29788900	-0.28464300	-1.32947200

C	11.36138100	-2.04361100	-2.14294700
H	11.80369000	-1.89614700	-3.13411700
H	11.34766000	-3.12163100	-1.94209900
C	9.92068100	-1.53884000	-2.16460000
H	9.91732200	-0.44625200	-2.27853900
C	9.13043300	-2.15629500	-3.31392300
H	9.30483600	-3.23927900	-3.32184200
H	9.54609700	-1.76542200	-4.24763000
C	7.62126500	-1.87071200	-3.24146500
H	7.43034500	-0.99968100	-2.61043500
H	7.23878400	-1.61591200	-4.23458000
C	6.79142500	-3.04862300	-2.73324700
H	6.83703900	-3.85558600	-3.47750200
C	5.31128900	-2.64656500	-2.52785400
H	5.05685000	-1.81199400	-3.19047600
C	4.33503700	-3.79476500	-2.76271700
H	4.56484400	-4.60712200	-2.06250800
H	4.47949100	-4.18304000	-3.77732900
C	2.87751400	-3.36900500	-2.60562900
H	2.66579700	-2.55240400	-3.30819100
C	1.91250900	-4.51379700	-2.89734400
H	2.14247400	-5.36476100	-2.24850100
H	2.04798300	-4.82828200	-3.93628800
C	0.44590300	-4.14136200	-2.67983600
C	-0.53314600	-5.22107800	-3.13259700
H	-0.40395900	-6.12168800	-2.52780300

H	-0.34431700	-5.47335000	-4.18038900
C	-1.95461600	-4.71207800	-2.97291000
H	-2.10634500	-4.45292700	-1.92534400
C	-2.13358800	-3.40104400	-3.78271800
H	-2.04802900	-3.63897800	-4.84728000
C	-1.05908900	-2.38927100	-3.35750100
H	-1.23555700	-2.10651600	-2.31577100
C	-3.52223300	-2.89495300	-3.49348300
C	17.58990500	-1.80978700	1.08197900
H	16.94219500	-2.44747300	1.68795100
H	18.37663600	-1.39296900	1.71229300
H	18.04725100	-2.42388000	0.30320800
C	18.51709800	-0.27757500	-1.34380700
H	19.34527600	-0.79590100	-0.85120200
H	18.94051800	0.47958100	-2.01013400
H	17.99219600	-1.01169900	-1.95918000
C	18.16214000	3.31498100	2.17379900
H	18.48001400	4.05857300	1.43457400
H	19.05233800	2.91495600	2.66115500
H	17.54796000	3.82618100	2.91851400
O	-2.11563100	1.24406400	-0.31286300
H	-1.34924600	1.81960400	-0.49677400
O	-2.48282000	4.80912800	-2.89742900
H	-3.39053600	5.06617900	-3.16781700
O	-3.02996400	1.31369200	-3.83663500
O	-1.11534600	0.73873200	-2.61371200

O	0.18127500	-3.90079800	-1.32572300
H	0.93977300	-3.41303600	-0.95817100
O	0.23549300	-2.97901100	-3.46871000
O	-3.79225200	-2.20688500	-2.51646100
O	-4.44223000	-3.33903100	-4.33311600
H	-5.33345800	-3.15343800	-3.93799900
O	2.62819300	-2.89579000	-1.27623500
H	3.46977100	-2.55148900	-0.92195500
O	5.18072600	-2.15589900	-1.19011400
H	5.86404000	-2.66829800	-0.70992800
O	7.25359000	-3.57374600	-1.49591400
H	8.02399400	-3.04780100	-1.19046500
O	9.24744700	-1.86432600	-0.94502000
H	9.89458800	-1.78053900	-0.22084800
O	11.63517400	-1.54730600	0.16650600
H	12.28254100	-1.26404100	0.83696400
O	14.12124300	-0.93754900	1.01592900
O	15.81039500	-1.25599600	-0.44020200
O	19.30439100	0.82254600	1.34065100
H	20.04269000	0.57712400	0.77291900
O	-2.86146900	-5.71554800	-3.37027400
H	-3.69199100	-5.53069800	-2.90548200
N	-3.46441400	3.67211900	-0.43536800
H	-2.64252200	3.47541900	0.13055900
H	-4.26634300	3.38346300	0.11780600

AmB-Cyc3

0 1

C	1.66518900	9.98791900	0.03644500
C	2.88438400	9.83604700	-0.86185800
C	3.56369900	8.50034100	-0.64891500
C	3.89235700	8.32620100	0.82476600
C	2.62196800	8.47019400	1.66533400
C	2.83304300	8.47665300	3.17911700
O	0.65563100	9.10504000	-0.35583300
O	2.50903500	9.99634900	-2.21614700
O	4.70296200	8.52139600	-1.47041900
O	2.04656000	9.76552500	1.37617600
O	3.22517100	7.23194600	3.68916200
H	4.60556000	9.10703700	1.12361500
H	1.30269000	11.02062300	0.00481100
H	3.59670600	10.62208100	-0.57627600
H	2.86966400	7.69699000	-0.93896300
H	1.90763300	7.68183100	1.40652300
H	3.54228300	9.28148500	3.42909800
H	1.87443700	8.72860300	3.63989400
H	3.25882400	9.66883800	-2.73520700
H	5.29680500	7.77987800	-1.23929400
H	4.15896600	7.07566900	3.48170000
C	5.75451400	6.94555300	1.54469800

C	6.69503000	6.18408400	0.62361300
C	6.25698800	4.74355200	0.47383600
C	6.10832100	4.09788700	1.84267600
C	5.17978500	4.92685400	2.73250600
C	5.10521500	4.47831300	4.19177700
O	4.48814100	7.04419600	0.96091200
O	6.74961700	6.82044100	-0.64005200
O	7.23517200	4.13218600	-0.32741300
O	5.68780100	6.28007300	2.78595200
O	4.41665600	3.26990900	4.36512000
H	7.09861600	4.02791100	2.31382300
H	6.15670200	7.94056000	1.76241900
H	7.68902300	6.19149800	1.09060500
H	5.26849200	4.72600200	-0.00934000
H	4.16937700	4.93016400	2.31176500
H	6.12636900	4.44913000	4.60401900
H	4.55132400	5.24508500	4.73951800
H	7.15798400	6.17328200	-1.23511900
H	7.10689900	3.16175000	-0.34146900
H	4.96541500	2.53075800	4.06064700
C	6.25881300	1.67719000	2.08714500
C	6.50097400	0.72080100	0.93544400
C	5.17334200	0.23490800	0.37955500
C	4.40413500	-0.41469800	1.52258000
C	4.21271900	0.56592300	2.68324600
C	3.59567100	-0.01922300	3.95289400

O	5.57508100	2.80380900	1.61722800
O	7.30207200	1.37781000	-0.02413600
O	5.42891900	-0.67961600	-0.66333800
O	5.51515300	1.03602600	3.09747100
O	2.21980900	-0.26299200	3.84223100
H	4.98530500	-1.27984500	1.87064700
H	7.21169500	1.96170200	2.54587200
H	7.03090100	-0.15260100	1.32552000
H	4.59769000	1.08938700	0.00079000
H	3.60274700	1.41467100	2.35805500
H	4.16451900	-0.91681200	4.24315500
H	3.73055400	0.72399600	4.74328200
H	7.70575700	0.66713100	-0.55944300
H	4.67882900	-1.30017900	-0.78198700
H	2.07907800	-1.07115700	3.32489100
C	2.78787200	-2.17987000	1.20190800
C	2.53636800	-2.87376100	-0.13033500
C	1.25753500	-2.38793100	-0.78117800
C	0.10768300	-2.45524400	0.21741000
C	0.44427400	-1.66885200	1.48283100
C	-0.55330600	-1.80617500	2.63677000
O	3.15857600	-0.84933400	0.98805900
O	3.66699300	-2.67226900	-0.94653400
O	0.99015200	-3.23673300	-1.89746100
O	1.64645200	-2.25350300	2.01982200
O	-1.87345600	-1.46505700	2.30362800

H	-0.04115000	-3.50186900	0.51087700
H	3.57239700	-2.72097000	1.73609100
H	2.40654300	-3.93523200	0.08160500
H	1.38421400	-1.35246700	-1.11790000
H	0.61451500	-0.61428000	1.24117500
H	-0.47600700	-2.83294800	3.01211300
H	-0.22786200	-1.13196600	3.43334400
H	3.76206700	-3.43740800	-1.54287400
H	0.18504500	-2.94119500	-2.38106700
H	-2.25212900	-2.18600900	1.78230700
C	-2.03860800	-2.91049000	-0.72949200
C	-2.50347900	-2.79831900	-2.17497000
C	-3.24853200	-1.50641400	-2.41893200
C	-4.37860100	-1.36581700	-1.41521200
C	-3.84227800	-1.48448300	0.01063700
C	-4.90863400	-1.48929700	1.10482600
O	-1.05073000	-1.96777900	-0.45051700
O	-1.39064000	-2.89405400	-3.04567500
O	-3.69975000	-1.56403000	-3.74985100
O	-3.16037600	-2.75425900	0.12262700
O	-5.44811200	-0.21915500	1.35055800
H	-5.11590800	-2.16091500	-1.59460000
H	-1.67266700	-3.91978100	-0.53951700
H	-3.19170300	-3.63314600	-2.35895900
H	-2.55532900	-0.66601200	-2.26398800
H	-3.13740000	-0.67450600	0.22018800

H	-5.68311900	-2.22560800	0.84409300
H	-4.43577300	-1.82936400	2.02892900
H	-1.70006000	-2.62256500	-3.92127500
H	-4.38659600	-0.88532400	-3.89360800
H	-6.08979800	-0.00112300	0.65825500
C	-6.33574800	0.02544700	-1.82430400
C	-6.63158400	0.69591900	-3.15682900
C	-6.10972100	2.11713700	-3.16151000
C	-6.65034400	2.88476900	-1.96503900
C	-6.35652900	2.13568100	-0.66345600
C	-7.01669700	2.71026400	0.58868700
O	-4.95685000	-0.08977700	-1.65716200
O	-6.04385100	-0.05218700	-4.20531700
O	-6.50981100	2.66755700	-4.39123100
O	-6.90146200	0.80314300	-0.79059900
O	-6.47124800	3.94036800	0.98427300
H	-7.73789700	2.99436800	-2.07748300
H	-6.82236700	-0.95401700	-1.76275800
H	-7.72222900	0.72885700	-3.28095200
H	-5.01409500	2.08586900	-3.06933400
H	-5.27592100	2.08009200	-0.50096800
H	-8.10423500	2.76204000	0.42216600
H	-6.84120600	2.00213300	1.40261600
H	-6.07548000	0.52447000	-4.98345300
H	-6.30921100	3.62373600	-4.40501300
H	-6.77818500	4.64086500	0.38951200

C	-6.82102400	5.30557100	-2.03644700
C	-6.40730400	6.18740400	-3.20479300
C	-5.01749300	6.74906200	-2.99297100
C	-4.95653300	7.47398500	-1.65773000
C	-5.38541700	6.53604900	-0.52672500
C	-5.53945200	7.19146500	0.84510300
O	-6.02381200	4.16183900	-1.99332100
O	-6.46948300	5.45184900	-4.41158600
O	-4.78292300	7.59399800	-4.09057400
O	-6.70957200	6.04636600	-0.84058300
O	-4.32451500	7.63107700	1.38945800
H	-5.64927200	8.32642200	-1.68840600
H	-7.87936200	5.03657600	-2.11860700
H	-7.10995400	7.03079500	-3.24408200
H	-4.29928400	5.91624000	-2.96098100
H	-4.68840100	5.69562700	-0.44957700
H	-6.28355000	7.99915600	0.76178900
H	-5.94618700	6.43677200	1.52306600
H	-5.98090500	5.97903400	-5.06161100
H	-3.95299700	8.09225600	-3.95633500
H	-4.04311700	8.43848000	0.93412700
C	-3.41369600	9.31222400	-1.35112500
C	-2.38289200	9.80626100	-2.35609000
C	-1.00147900	9.27406900	-2.03866100
C	-0.64055500	9.63384000	-0.60653700
C	-1.69880800	9.08024300	0.35043500

C	-1.54851100	9.50204300	1.81178500
O	-3.62137600	7.93928000	-1.49751900
O	-2.76952700	9.44256700	-3.66669400
O	-0.14877400	9.85983100	-2.98882800
O	-2.97753600	9.62757300	-0.04682400
O	-0.41563900	8.95918900	2.43299700
H	-0.62223800	10.72848000	-0.51193100
H	-4.35452000	9.85695400	-1.48152100
H	-2.34530000	10.90078700	-2.26890700
H	-1.01165800	8.17696700	-2.12422400
H	-1.72849800	7.98737500	0.29044200
H	-1.57198700	10.60219900	1.85834400
H	-2.42471800	9.13103200	2.34966100
H	-1.98224500	9.57247600	-4.21643500
H	0.78440300	9.70411200	-2.74370100
H	0.37281700	9.43361900	2.12982100
C	10.08825200	-1.63550700	-0.01713300
H	10.74943400	-2.48421100	0.19512300
C	9.88392000	-0.88355200	1.30059300
H	9.25923600	0.00146100	1.10382400
C	9.17152700	-1.76818000	2.32146800
H	9.83962500	-2.61883700	2.53646500
C	8.14281400	-3.01446100	0.59137900
H	8.86280800	-3.82138400	0.80779700
C	8.75712800	-2.18394200	-0.53431800
H	8.93650600	-2.86255000	-1.37593400

C	8.83603000	-1.04582000	3.60753300
H	8.09701500	-0.26122300	3.42442000
H	9.74006400	-0.59483500	4.01980200
H	8.41196800	-1.73963800	4.33638300
C	6.62630500	-4.81639600	0.81965600
H	6.99210200	-4.80298900	1.85407100
C	5.15037800	-5.03725500	0.83025900
H	4.67599400	-5.01824800	-0.14402900
C	4.43073800	-5.33726600	1.91877000
H	4.89891700	-5.33753800	2.90266500
C	3.00788100	-5.60872800	1.82586200
H	2.63045500	-5.76891000	0.81935500
C	2.09295400	-5.58010000	2.81322200
H	2.38806900	-5.41984900	3.84908400
C	0.68588300	-5.62475700	2.48125500
H	0.47797400	-5.70853900	1.41768100
C	-0.35885100	-5.45751200	3.31447200
H	-0.20367800	-5.36915700	4.38889900
C	-1.70112900	-5.26877900	2.81058700
H	-1.82091100	-5.25725900	1.72644000
C	-2.78843700	-5.02660100	3.56909100
H	-2.69567900	-5.04497400	4.65425400
C	-4.08017500	-4.68486000	3.02461100
H	-4.13539000	-4.53096100	1.94779400
C	-5.18717700	-4.49466400	3.76985100
H	-5.13448700	-4.64662100	4.84719700

C	-6.45145200	-4.06233100	3.22634800
H	-6.46736000	-3.81973800	2.16684000
C	-7.59034500	-3.92635600	3.92881600
H	-7.60638800	-4.18924600	4.98570500
C	7.35404700	-5.93304900	0.04385200
H	8.41604200	-5.88175000	0.28855500
H	6.98588900	-6.90503200	0.38600000
C	-8.82524000	-3.45484100	3.33163900
H	-8.73361400	-3.04562800	2.32772700
C	-10.03237500	-3.52056800	3.90287900
H	-10.12490600	-3.94443000	4.90401900
C	-11.31388200	-3.08087100	3.25165700
H	-11.07516500	-2.47432500	2.37065700
C	-12.13801600	-4.28985100	2.76712800
H	-12.45834800	-4.84653500	3.66335000
C	-11.36922500	-5.28935400	1.88163500
H	-10.51230100	-5.61821900	2.47707100
C	-10.77025900	-4.64595600	0.62582800
H	-10.26888900	-3.71883200	0.88985500
C	-8.51406000	-5.10401000	-0.05657400
C	-7.56598600	-6.16841400	-0.55201100
H	-7.94661900	-7.15312400	-0.27541800
H	-7.55669100	-6.10657600	-1.64650800
C	-6.13934800	-5.96067200	-0.03525900
H	-6.14092000	-6.04356800	1.06217200
C	-5.18304900	-6.99160800	-0.61951100

H	-5.54389000	-7.99439100	-0.36619400
H	-5.19500000	-6.89678500	-1.71166000
C	-3.73913000	-6.85102600	-0.14048400
H	-3.71174400	-6.90902100	0.95843900
C	-2.84758700	-7.94562000	-0.72193600
H	-3.05649900	-8.03031600	-1.79523400
H	-3.14825000	-8.89512600	-0.26891000
C	-1.34233800	-7.70082300	-0.50786500
H	-1.18545300	-6.97730700	0.29636300
H	-0.85636700	-8.62808600	-0.18923700
C	-0.61106100	-7.21212600	-1.76076200
H	-0.62339400	-8.02784000	-2.50165900
C	0.85099500	-6.86521200	-1.44876100
H	1.28274000	-7.64860500	-0.81347500
C	1.70338400	-6.72499200	-2.70822200
H	1.19278500	-6.06779800	-3.42182000
H	1.77703700	-7.70543100	-3.19065800
C	3.10830900	-6.17566200	-2.42462700
H	3.37649100	-6.37807100	-1.38248300
C	4.15281000	-6.82578600	-3.32179100
H	3.85916000	-6.70905600	-4.36994900
H	4.16523000	-7.89555400	-3.09434300
C	5.57682200	-6.28485900	-3.18525800
C	6.56250900	-7.14197000	-3.96631300
H	6.34081900	-7.08694500	-5.03395000
H	6.46556400	-8.18345200	-3.64686700

C	7.99888700	-6.71558800	-3.73433600
H	8.14397100	-5.70691700	-4.14838100
C	8.28277800	-6.65826300	-2.21013300
H	8.28902000	-7.69520000	-1.85359000
C	7.19994400	-5.85081600	-1.47181900
H	7.26795500	-4.80671600	-1.78813800
C	9.65283800	-6.07344000	-1.97406900
C	-11.71464700	-4.40084900	-0.53309200
H	-11.18812300	-3.89275400	-1.34440400
H	-12.54034400	-3.77539800	-0.19181600
H	-12.11677700	-5.33985000	-0.91938000
C	-12.21951900	-6.51771500	1.56171600
H	-13.13614600	-6.25183100	1.02662000
H	-12.50366800	-7.03980400	2.47985300
H	-11.66601300	-7.21913900	0.93335600
C	-12.14801600	-2.22096700	4.20516000
H	-12.39651500	-2.78230400	5.11282900
H	-13.07957900	-1.91395700	3.72859600
H	-11.59106400	-1.33061300	4.50556000
O	7.97073800	-1.08984100	-0.95002000
H	7.00132600	-1.26182100	-0.89196800
O	11.13362000	-0.50586700	1.83089500
H	11.63640000	-0.19921900	1.05899100
O	7.94791900	-2.25413000	1.76792800
O	6.91195600	-3.54094800	0.21815000
O	5.68713200	-4.97721700	-3.67145600

H	4.87599300	-4.51065400	-3.41338700
O	5.90567600	-6.35311800	-1.79876200
O	9.91730000	-5.03407400	-1.41592600
O	10.63542100	-6.84260700	-2.50215500
H	11.47160700	-6.37775000	-2.33403900
O	3.17226800	-4.75118800	-2.63106100
H	2.29888300	-4.32041700	-2.52891500
O	0.90114300	-5.67898300	-0.68076900
H	0.58859600	-4.94257900	-1.23863100
O	-1.19675800	-6.06718500	-2.35211700
H	-1.96470600	-5.79430600	-1.81826600
O	-3.19079700	-5.59667200	-0.53677300
H	-3.86706800	-4.90238700	-0.41459800
O	-5.66090500	-4.67112400	-0.40736700
H	-6.37229000	-4.03723500	-0.20309700
O	-8.18061200	-3.93827700	0.10239300
O	-9.73302500	-5.56883900	0.15911500
O	-13.28027300	-3.75901100	2.10107700
H	-13.95565800	-4.44378800	2.05192100
O	8.79979300	-7.66721300	-4.39851600
H	9.72420800	-7.49850400	-4.17745000
N	10.79606800	-0.74531400	-0.93081400
H	10.13007100	-0.08636200	-1.32655300
H	11.18539100	-1.26909500	-1.70809500

AmB-Cyc4

0 1

C	0.63754700	3.28284400	0.29802600
C	0.65022400	4.32308800	-0.81245000
C	2.05096100	4.85667900	-1.02155100
C	2.59622300	5.40369800	0.28769700
C	2.52145200	4.34587700	1.39023600
C	2.82347600	4.85705000	2.79900600
O	1.38245300	2.16659100	-0.08157000
O	0.14495300	3.76006400	-2.00430500
O	1.94175000	5.84221900	-2.01767100
O	1.15936100	3.86928300	1.47367900
O	4.15124200	5.27394500	2.96938400
H	1.99201100	6.27241300	0.58400100
H	-0.39068400	2.99766500	0.54324400
H	0.01207600	5.15633700	-0.48705100
H	2.69963300	4.02762000	-1.33998100
H	3.19067000	3.51314000	1.15176600
H	2.09736600	5.64914100	3.03985000
H	2.65259300	4.03082700	3.49347100
H	0.41654900	4.36003900	-2.71489400
H	2.81173700	6.26285500	-2.16111000
H	4.28577900	6.11799500	2.51365500
C	4.27434500	7.14918800	0.19817700
C	4.99952100	7.65799000	-1.03903600

C	6.34971700	6.99028000	-1.18738000
C	7.15749800	7.18480800	0.08578900
C	6.37738500	6.65796000	1.29212500
C	6.99836700	6.95370900	2.65672900
O	3.93889900	5.80454900	0.03982700
O	4.20583400	7.44077000	-2.18907900
O	6.94474700	7.58499800	-2.31282600
O	5.10147200	7.33732700	1.32699200
O	8.18825300	6.25006400	2.88899100
H	7.34367300	8.25883900	0.22473500
H	3.37960600	7.75134700	0.38690100
H	5.16880800	8.73427200	-0.89850300
H	6.19709900	5.91043100	-1.33228900
H	6.21933900	5.57912800	1.19581000
H	7.12439700	8.04357700	2.75343700
H	6.28035400	6.63587100	3.41650800
H	4.80221400	7.54875100	-2.94482300
H	7.88047700	7.31210400	-2.37757300
H	8.90560500	6.65408100	2.37849000
C	9.57970700	7.21792200	0.02103600
C	10.44801800	7.01576000	-1.21236800
C	10.95820000	5.59228900	-1.30041700
C	11.66406700	5.22582500	-0.00505900
C	10.71883200	5.43023200	1.18071200
C	11.33617300	5.22030200	2.56278700
O	8.38999000	6.49877800	-0.10126600

O	9.71809500	7.36036800	-2.37374700
O	11.80551100	5.56306600	-2.42069500
O	10.30304300	6.81502100	1.16476500
O	11.59987200	3.87241900	2.84521800
H	12.53534700	5.88321200	0.12089300
H	9.37255500	8.28328800	0.16566800
H	11.31975200	7.67590700	-1.10822300
H	10.10100100	4.91342200	-1.42393400
H	9.84353000	4.78011800	1.08409900
H	12.23076100	5.85656700	2.65228600
H	10.60893300	5.56969900	3.29973400
H	10.23225100	7.01830000	-3.11998200
H	12.36368100	4.76203700	-2.39652600
H	12.40359200	3.59654700	2.37976200
C	13.43123900	3.57022100	0.10249200
C	14.03994600	2.87257900	-1.10472900
C	13.43851600	1.49792300	-1.30755300
C	13.55367600	0.69277400	-0.02296000
C	12.90076100	1.45305900	1.13313500
C	13.06130000	0.82134100	2.51500400
O	12.09079900	3.87610800	-0.14072200
O	13.85975400	3.67370700	-2.25656200
O	14.14597000	0.93213400	-2.38106600
O	13.56228900	2.73414500	1.23170700
O	12.31340200	-0.35505300	2.67085300
H	14.61757500	0.54529500	0.20833200

H	13.99659800	4.47712700	0.34058600
H	15.11147300	2.74211900	-0.90157300
H	12.36891300	1.60935700	-1.54143800
H	11.83476500	1.60113800	0.93301300
H	14.13420300	0.67186000	2.71285200
H	12.68956500	1.54277400	3.24723700
H	14.08444100	3.10579800	-3.00864900
H	13.96563700	-0.02658800	-2.43240600
H	12.76339600	-1.08472400	2.21943000
C	13.66030000	-1.72944000	-0.03671800
C	13.67535300	-2.59410100	-1.28786100
C	12.28694000	-3.10576500	-1.60265500
C	11.71405900	-3.82781000	-0.39353900
C	11.74421300	-2.92065300	0.83790200
C	11.38955000	-3.60347400	2.15883000
O	12.93129900	-0.56148900	-0.26923000
O	14.19261800	-1.85414000	-2.37659300
O	12.43579000	-3.94318300	-2.72090100
O	13.10422700	-2.47017100	1.02733000
O	10.05845400	-4.04145400	2.22249000
H	12.32391200	-4.71959800	-0.19274900
H	14.68289100	-1.49107600	0.27365200
H	14.31950300	-3.45970500	-1.08223200
H	11.63604400	-2.24647600	-1.82228200
H	11.08678500	-2.05916900	0.68320300
H	12.11062600	-4.41781400	2.32920800

H	11.52834200	-2.86799300	2.95547500
H	13.96865400	-2.36736300	-3.16771800
H	11.58113800	-4.36621800	-2.93509500
H	9.95293800	-4.83120500	1.67175400
C	10.06899200	-5.56744000	-0.76061400
C	9.39631500	-5.91852200	-2.07937600
C	8.05858500	-5.22201000	-2.19015100
C	7.19303600	-5.57644900	-0.99199400
C	7.91838800	-5.25343200	0.31503400
C	7.24815400	-5.79889600	1.57648800
O	10.38694200	-4.20891900	-0.73342500
O	10.23231200	-5.56308200	-3.16301600
O	7.50224500	-5.64808700	-3.40854400
O	9.20674400	-5.90887700	0.30275900
O	5.97298500	-5.26508000	1.81581500
H	6.97053900	-6.65227100	-1.02282600
H	10.96650700	-6.17784900	-0.61720500
H	9.22168400	-7.00303100	-2.08052300
H	8.22566900	-4.13520200	-2.17848800
H	8.05485800	-4.17106300	0.40531400
H	7.23718000	-6.89754800	1.50581200
H	7.87781300	-5.52769000	2.42747000
H	9.65636800	-5.55621900	-3.94265900
H	6.62890600	-5.22804600	-3.53675200
H	5.34980500	-5.61888800	1.16472300
C	4.79742800	-5.51001600	-1.32452600

C	4.03866000	-4.89256300	-2.48878400
C	3.57802700	-3.48302000	-2.16796800
C	2.76355000	-3.51781800	-0.88802000
C	3.57545500	-4.15247300	0.24428300
C	2.79789600	-4.38135000	1.53711100
O	5.99255200	-4.82366500	-1.11285000
O	4.84792300	-4.89993100	-3.64829100
O	2.85499500	-3.05384200	-3.29213300
O	3.98349300	-5.47347000	-0.16818400
O	2.62081700	-3.19419800	2.29916600
H	1.86443500	-4.12314600	-1.06437000
H	4.98693100	-6.57117200	-1.51495100
H	3.14253600	-5.50616300	-2.65435700
H	4.46085100	-2.84941300	-1.99673100
H	4.45963300	-3.54462700	0.46207300
H	1.83051600	-4.84816200	1.31898700
H	3.38500700	-5.05574800	2.16223600
H	4.41523800	-4.30377800	-4.27765300
H	2.13748200	-2.44052300	-3.02825500
H	2.20351300	-2.50085900	1.75537000
C	1.04771400	-1.96017700	-0.23155100
C	0.33689800	-1.16511200	-1.32192100
C	0.82758900	0.26590200	-1.38023000
C	0.79719300	0.87250500	0.02402100
C	1.53065700	0.02103500	1.05802500
C	1.33096900	0.44133600	2.51398300

O	2.39577000	-2.18279200	-0.56678600
O	0.51387900	-1.81006500	-2.56247700
O	-0.02518200	0.97069900	-2.23688100
O	0.98468500	-1.31074400	1.01235700
O	2.00751100	1.61818400	2.85596000
H	-0.25638000	0.96074400	0.32493400
H	0.54704100	-2.91480300	-0.06912900
H	-0.73041800	-1.12881200	-1.07394400
H	1.87133100	0.26986700	-1.73080300
H	2.60100300	-0.00307100	0.82578400
H	0.24859600	0.50030500	2.71143700
H	1.72194800	-0.36379200	3.14282700
H	-0.18503300	-2.49168800	-2.61469400
H	0.21525300	1.91500200	-2.22388800
H	1.52798900	2.37736900	2.48938800
C	-2.27958300	-6.08981200	-2.26779900
H	-1.32405800	-6.13831400	-2.80185800
C	-3.18523800	-5.15616100	-3.06819800
H	-4.09682300	-4.95710200	-2.48332800
C	-2.44276200	-3.84829700	-3.32604500
H	-1.73964100	-4.05305600	-4.13951800
C	-2.00602200	-3.95950300	-0.92366500
H	-1.27337100	-3.57512600	-0.22030000
C	-2.03821800	-5.50702200	-0.85886500
H	-1.06662600	-5.84707000	-0.48504000
C	-3.34385800	-2.69015700	-3.69758300

H	-4.07989400	-2.50672900	-2.91206100
H	-3.87490800	-2.93424800	-4.62088200
H	-2.75491900	-1.78316300	-3.85765700
C	-3.44472500	-2.14127100	-0.17246700
H	-3.06001200	-1.48837700	-0.96610500
C	-4.92750500	-1.98327000	-0.06412800
H	-5.42636800	-2.69182200	0.59167900
C	-5.64858100	-1.08310300	-0.73945500
H	-5.15750500	-0.36660800	-1.39764000
C	-7.09472500	-1.04341000	-0.65292900
H	-7.55692700	-1.80381600	-0.02869900
C	-7.89694600	-0.17945000	-1.30176000
H	-7.46492900	0.59284300	-1.93744500
C	-9.33564400	-0.25067700	-1.20111500
H	-9.72325900	-1.04657700	-0.56755500
C	-10.20538600	0.57361600	-1.81795500
H	-9.83337900	1.37799900	-2.45167800
C	-11.63465600	0.43360100	-1.68620500
H	-11.99133700	-0.37020000	-1.04232100
C	-12.54656600	1.22268800	-2.28983000
H	-12.20921000	2.04252400	-2.92308800
C	-13.96926000	1.02735800	-2.16258800
H	-14.29712000	0.18683900	-1.55128400
C	-14.90488700	1.80393600	-2.74498200
H	-14.59112800	2.65991700	-3.34142900
C	-16.32235000	1.55970900	-2.63029000

H	-16.62039500	0.66222200	-2.09112800
C	-17.28735600	2.35838800	-3.12169000
H	-17.01380000	3.27609800	-3.64105100
C	-2.67246700	-1.85094500	1.12516400
H	-1.60086500	-1.83093200	0.92128800
H	-2.94834300	-0.84746400	1.46401200
C	-18.69953300	2.07503400	-2.95358900
H	-18.93545800	1.10009000	-2.53198300
C	-19.69468300	2.92775600	-3.21992800
H	-19.45765200	3.91221300	-3.62681000
C	-21.14787000	2.65563800	-2.95335200
H	-21.26885800	1.59929900	-2.68700300
C	-21.65970600	3.48605800	-1.75998300
H	-21.67374900	4.54176300	-2.07832500
C	-20.78018800	3.42170800	-0.49766800
H	-19.79085600	3.77346800	-0.80607100
C	-20.56109200	1.99420900	0.01564600
H	-20.30999000	1.33894700	-0.81383300
C	-18.39453700	1.19668900	0.67100500
C	-17.24922100	1.39204800	1.63241700
H	-17.25634300	2.41918100	2.00195500
H	-17.42536600	0.72845200	2.48701300
C	-15.89882800	1.04052300	1.00350800
H	-15.72048700	1.69978700	0.14197600
C	-14.76677700	1.20533500	2.00701900
H	-14.74536400	2.24734500	2.34377500

H	-14.97338300	0.57734300	2.88198800
C	-13.38929800	0.83437900	1.46323600
H	-13.19851100	1.39219900	0.53598400
C	-12.29165900	1.16184100	2.47033700
H	-12.58977200	0.78052700	3.45478700
H	-12.24342400	2.25143700	2.55923800
C	-10.91209500	0.59932100	2.09077800
H	-10.86325800	0.40120000	1.01717000
H	-10.13692400	1.34401200	2.29664800
C	-10.52656200	-0.66312600	2.86008500
H	-10.43231700	-0.40064900	3.92315200
C	-9.16819800	-1.23690300	2.37635300
H	-8.59842200	-0.45264200	1.86409400
C	-8.32379800	-1.80390300	3.51168100
H	-8.88330600	-2.59960300	4.01839100
H	-8.14203700	-1.00922100	4.24460900
C	-6.98135000	-2.34676200	3.03004100
H	-6.46845300	-1.56425500	2.45409600
C	-6.08115700	-2.75024600	4.18886500
H	-6.59229800	-3.48612500	4.81726700
H	-5.88417500	-1.86214800	4.79589800
C	-4.74600100	-3.35742400	3.75257200
C	-3.77346800	-3.48536700	4.91950500
H	-4.12934100	-4.24579900	5.61766800
H	-3.72576300	-2.52952900	5.44892300
C	-2.35929400	-3.82818900	4.47870200

H	-2.34657400	-4.84760700	4.06387300
C	-1.90457300	-2.86030900	3.35662700
H	-1.80254800	-1.86508300	3.80311400
C	-2.96115900	-2.83747700	2.24785500
H	-3.04035200	-3.84918000	1.83900400
C	-0.55339100	-3.29796300	2.85072600
C	-21.68532400	1.37980600	0.82425600
H	-21.43928600	0.34705000	1.08181900
H	-22.60018400	1.39427200	0.23030900
H	-21.85461000	1.93405000	1.74995000
C	-21.29547500	4.36397600	0.58893100
H	-22.31362800	4.11095200	0.89950200
H	-21.29775600	5.39855900	0.23376600
H	-20.66117800	4.31424700	1.47679500
C	-22.00375400	2.93359000	-4.19229800
H	-21.89025800	3.97485100	-4.51399200
H	-23.05770200	2.75629800	-3.97416700
H	-21.69947900	2.29264500	-5.02283900
O	-3.01233600	-5.95751700	0.05543800
H	-3.66660000	-5.24631900	0.13470200
O	-3.50516900	-5.72663600	-4.31228100
H	-3.60108900	-6.67423400	-4.10856900
O	-1.59927000	-3.50024300	-2.19513100
O	-3.28796400	-3.51159800	-0.59291300
O	-4.91274200	-4.62534200	3.18491300
H	-5.69899400	-4.56524400	2.61370800

O	-4.20936400	-2.44386000	2.80116400
O	-0.36192900	-3.86034700	1.78644600
O	0.42117600	-3.05020500	3.72177300
H	1.30681000	-3.25744700	3.29848400
O	-7.16234900	-3.49022700	2.19054800
H	-7.98868500	-3.35315000	1.69234200
O	-9.43136800	-2.24837700	1.40336000
H	-10.32920300	-2.55606700	1.65086000
O	-11.48333100	-1.70690300	2.75550300
H	-12.23509700	-1.40408500	2.20172200
O	-13.31640100	-0.56327000	1.16755300
H	-14.18643100	-0.83766800	0.82379600
O	-15.89031100	-0.31603700	0.56619900
H	-16.71265100	-0.45973400	0.06422300
O	-18.40607500	0.32273500	-0.18173200
O	-19.37845000	2.05992300	0.87368200
O	-22.99064900	3.04518400	-1.50899600
H	-23.44501900	3.71413600	-0.98585800
O	-1.55196300	-3.73265800	5.63093500
H	-0.62469200	-3.70883700	5.35839400
N	-2.87219400	-7.42090700	-2.27155900
H	-3.50817100	-7.47859400	-1.47837800
H	-2.16394900	-8.13265500	-2.12877300

AmB-Cyc5

0 1

C	-7.37396300	2.37920500	-6.50590600
C	-8.82558600	1.94436000	-6.64378500
C	-9.05784000	0.62184800	-5.94609100
C	-8.08555900	-0.41668000	-6.48193000
C	-6.64500200	0.07697800	-6.33159200
C	-5.58123100	-0.78760600	-7.00695900
O	-7.08560100	2.66688100	-5.17187500
O	-9.68238200	2.94138800	-6.12188100
O	-10.40223000	0.29308000	-6.19035100
O	-6.53664300	1.35523400	-6.99869300
O	-5.41758900	-2.04258800	-6.40402700
H	-8.29773500	-0.58276900	-7.54725500
H	-7.17759700	3.24937200	-7.14071500
H	-9.02433700	1.80262900	-7.71479200
H	-8.86038600	0.74914700	-4.87136500
H	-6.39639800	0.19049600	-5.27174400
H	-5.82198400	-0.86081200	-8.07921800
H	-4.62894100	-0.25951500	-6.91738500
H	-10.54272900	2.51284500	-6.00135500
H	-10.58223500	-0.61658000	-5.88287000
H	-6.16398700	-2.61377800	-6.63812200
C	-8.67066600	-2.76995200	-6.44138600
C	-9.93676700	-3.37297500	-5.85119200
C	-9.69133200	-3.89047000	-4.44951900

C	-8.51909400	-4.85833500	-4.45999400
C	-7.28282100	-4.18477500	-5.05977900
C	-6.07225000	-5.09333600	-5.27018900
O	-8.32057100	-1.61185800	-5.74651500
O	-10.97445600	-2.41160400	-5.85593400
O	-10.89793200	-4.48943000	-4.04958200
O	-7.63674200	-3.72960700	-6.38571700
O	-5.45082600	-5.46694700	-4.07035000
H	-8.78422500	-5.72698800	-5.07855600
H	-8.81426100	-2.55390700	-7.50515800
H	-10.21552500	-4.22847600	-6.48119000
H	-9.42601800	-3.04283800	-3.80004400
H	-6.98004300	-3.33180700	-4.44429800
H	-6.38086500	-5.95939200	-5.87680500
H	-5.33997000	-4.52792400	-5.85149700
H	-11.66672800	-2.76859900	-5.28004100
H	-10.75143800	-5.03172800	-3.25067800
H	-5.97806300	-6.15148000	-3.63208600
C	-8.29424800	-6.63080600	-2.82041700
C	-9.31229600	-6.95652700	-1.73719700
C	-8.91598800	-6.35084500	-0.40650400
C	-7.49904400	-6.77432200	-0.05548500
C	-6.54171500	-6.38214600	-1.18250800
C	-5.09549000	-6.84583300	-1.01628600
O	-8.31848200	-5.26610400	-3.11206200
O	-10.59130800	-6.50184500	-2.13520400

O	-9.87490000	-6.80170500	0.51603400
O	-7.01334300	-7.03268100	-2.38416600
O	-4.40358900	-6.12786700	-0.02998200
H	-7.47499500	-7.86639200	0.06267800
H	-8.49782900	-7.22101000	-3.71993400
H	-9.32314600	-8.04820700	-1.61668500
H	-8.92813300	-5.25418100	-0.49784200
H	-6.54628100	-5.29660600	-1.32293900
H	-5.08686500	-7.93236900	-0.83663200
H	-4.58615900	-6.66590900	-1.96643600
H	-11.14910800	-6.55887100	-1.34538400
H	-9.54149300	-6.68722900	1.42678100
H	-4.65814600	-6.45399900	0.84610400
C	-6.68946600	-6.93180400	2.22352400
C	-7.56685200	-6.77509300	3.45617500
C	-7.46277800	-5.37575600	4.02318600
C	-6.00445000	-5.02739000	4.27733200
C	-5.18782100	-5.20410600	2.99558400
C	-3.67631100	-5.04533900	3.15150600
O	-7.18119200	-6.14556000	1.17951100
O	-8.90777500	-7.08274700	3.12681700
O	-8.23927200	-5.38435700	5.19383000
O	-5.36710800	-6.56709100	2.55055100
O	-3.29169500	-3.73467600	3.46982800
H	-5.60680300	-5.70472400	5.04565700
H	-6.63962400	-7.98376900	1.92403300

H	-7.19572300	-7.47601400	4.21599500
H	-7.85488300	-4.66405600	3.28110100
H	-5.54508500	-4.51250200	2.22583500
H	-3.32197600	-5.78164200	3.88904400
H	-3.22012000	-5.29669400	2.19029100
H	-9.43725900	-6.77455400	3.87758900
H	-8.11884700	-4.54546600	5.67991200
H	-3.45332300	-3.57424900	4.41065100
C	-5.43926800	-3.43226100	6.01078000
C	-6.42335100	-2.62462400	6.84424100
C	-6.62340000	-1.25553500	6.23429300
C	-5.28409200	-0.55045500	6.09471300
C	-4.29397700	-1.40990500	5.30809200
C	-2.84535700	-0.92894300	5.36586700
O	-5.97929200	-3.68591800	4.74968800
O	-7.65647900	-3.30848500	6.94103700
O	-7.50736400	-0.57348700	7.09180400
O	-4.22621400	-2.71840800	5.91500600
O	-2.66261500	0.38606100	4.87864300
H	-4.87589500	-0.36299200	7.09785700
H	-5.18678900	-4.36811400	6.51951200
H	-5.98368000	-2.49932300	7.84312900
H	-7.04980900	-1.37715600	5.22888300
H	-4.62565400	-1.50090800	4.26882700
H	-2.49479400	-1.02839400	6.40229600
H	-2.23188200	-1.58068300	4.74132600

H	-8.29495000	-2.64411200	7.24257500
H	-7.78507800	0.26252500	6.67023400
H	-3.03305200	1.01615600	5.51225900
C	-5.50552000	1.84622200	6.18833000
C	-6.72560500	2.69945000	5.86367400
C	-6.68111600	3.16829000	4.42536300
C	-5.38411100	3.91229000	4.19072200
C	-4.16841500	3.07909900	4.60020200
C	-2.87312600	3.88374200	4.66597200
O	-5.51547300	0.68197300	5.42125000
O	-7.90522600	1.96048400	6.11290700
O	-7.80871400	3.98652900	4.22571000
O	-4.32927300	2.59379000	5.94531600
O	-2.24190900	4.11524800	3.41956600
H	-5.41307500	4.84048900	4.77723700
H	-5.48574700	1.61010500	7.25638200
H	-6.68987300	3.58301800	6.51522100
H	-6.70355000	2.28969600	3.76514700
H	-4.05061400	2.23693100	3.90941000
H	-3.05888200	4.83185500	5.18736600
H	-2.15741200	3.31509900	5.26422900
H	-8.62789500	2.43664600	5.67981700
H	-7.60382400	4.65762800	3.54808300
H	-2.83844100	4.34266900	2.67755800
C	-4.83500800	5.44921700	2.40423600
C	-5.93856600	6.31305100	1.81856000

C	-6.40451100	5.79970500	0.47373600
C	-5.21243700	5.56965000	-0.44466900
C	-4.22900800	4.60597700	0.22365000
C	-2.93021900	4.34101700	-0.53264600
O	-5.34714800	4.20737400	2.80442600
O	-7.01266100	6.35037900	2.74350400
O	-7.30294900	6.76627800	-0.00368800
O	-3.81345200	5.23395300	1.46503200
O	-3.13250700	3.63932800	-1.73453900
H	-4.69795300	6.52246500	-0.62869300
H	-4.36222300	5.96488600	3.24701200
H	-5.53050500	7.32111100	1.66697300
H	-6.89839500	4.82633100	0.62074000
H	-4.73049000	3.65706200	0.43943600
H	-2.42552600	5.30299200	-0.68150500
H	-2.29888000	3.71373100	0.10398900
H	-7.73377200	6.80655600	2.28489800
H	-7.51362800	6.59227700	-0.94226800
H	-3.43866400	4.26269500	-2.40851800
C	-5.47032900	5.72247300	-2.85204100
C	-6.77299100	6.00217800	-3.58747600
C	-7.41992000	4.70758600	-4.02789400
C	-6.44401600	3.89977700	-4.86767500
C	-5.13532800	3.67780300	-4.10799500
C	-3.99546200	3.08906500	-4.93944500
O	-5.73750000	5.04848800	-1.65779100

O	-7.65074500	6.73801000	-2.75710800
O	-8.57527000	5.07698300	-4.73887600
O	-4.62720800	4.96392800	-3.68981600
O	-4.25409300	1.79435700	-5.41253200
H	-6.23115200	4.45360600	-5.79263800
H	-4.93027200	6.65447300	-2.65750100
H	-6.52666900	6.58714200	-4.48385600
H	-7.66641000	4.11632900	-3.13392200
H	-5.31472000	3.04817300	-3.23081700
H	-3.76581900	3.79645300	-5.75133700
H	-3.11570000	3.02826900	-4.29407800
H	-8.52217600	6.66542100	-3.17525500
H	-9.02501400	4.27637100	-5.07284400
H	-4.91312400	1.83552800	-6.12071000
C	-0.30828100	5.95380100	1.53496300
H	-1.01122200	5.15272800	1.29019800
C	-0.15673800	6.83510400	0.29629100
H	0.51621200	7.66981900	0.55208700
C	0.48521800	6.06427200	-0.85542600
H	-0.19343500	5.24949500	-1.15175500
C	1.52244800	4.56975600	0.65496700
H	0.75225700	3.84770700	0.35099100
C	1.03586100	5.31624300	1.89017300
H	0.91125000	4.60473500	2.71407300
C	0.78499000	6.94216200	-2.04890000
H	1.49578300	7.72376200	-1.76684700

H	-0.13496500	7.41447200	-2.39866500
H	1.22087900	6.35260300	-2.85793200
C	3.11508500	2.91419100	0.01965700
H	3.03036200	3.28991100	-1.00709100
C	4.54183100	2.58753400	0.33036600
H	4.74932000	2.26529700	1.34790200
C	5.52894900	2.61221400	-0.56980900
H	5.32142900	2.93154700	-1.59082300
C	6.89150000	2.22795800	-0.25869500
H	7.07712800	1.88220300	0.75600600
C	7.91804700	2.27264400	-1.12921500
H	7.74215000	2.61847500	-2.14756900
C	9.26278400	1.88823500	-0.77396500
H	9.39942300	1.51932900	0.24057200
C	10.32766200	1.93691500	-1.59993600
H	10.20406500	2.29598200	-2.62120200
C	11.65041300	1.53483700	-1.19147700
H	11.75502300	1.15153800	-0.17654300
C	12.74873100	1.58655200	-1.97314200
H	12.65942700	1.95154700	-2.99575300
C	14.06177300	1.19941800	-1.52230600
H	14.14489500	0.85240200	-0.49244200
C	15.17568600	1.23695700	-2.28148300
H	15.10199400	1.56133300	-3.31889300
C	16.48389700	0.87537000	-1.79236800
H	16.55504800	0.61494300	-0.73759000

C	17.60240000	0.82350200	-2.53897300
H	17.55012600	1.05399000	-3.60241800
C	2.20183300	1.69307000	0.20612400
H	1.18657700	1.93142400	-0.11349600
H	2.55286600	0.88119500	-0.43615300
C	18.88920600	0.43109500	-1.99826000
H	18.92667000	0.31054300	-0.91738300
C	19.98200500	0.16355800	-2.72151900
H	19.94060300	0.26740600	-3.80705000
C	21.28464100	-0.32347400	-2.15336200
H	21.24330100	-0.25377900	-1.06023800
C	21.52885700	-1.80416700	-2.50422700
H	21.72094000	-1.85671300	-3.58892800
C	20.34025000	-2.74230800	-2.22500200
H	19.50543200	-2.34671300	-2.81152400
C	19.86616700	-2.70065700	-0.76828300
H	19.79745000	-1.66961800	-0.43290000
C	17.54616400	-2.53715200	-0.18203500
C	16.19724300	-3.20255500	-0.29079700
H	16.18613400	-3.86079600	-1.16158200
H	16.06876800	-3.82659900	0.60128900
C	15.05504400	-2.18537100	-0.35142700
H	15.18588400	-1.55073800	-1.23934100
C	13.70237500	-2.87889400	-0.42479000
H	13.67373600	-3.49680600	-1.32874900
H	13.59651300	-3.54724500	0.43825100

C	12.51200100	-1.92264500	-0.44092900
H	12.64551100	-1.18434500	-1.24321400
C	11.20226400	-2.66937100	-0.67256100
H	11.17348600	-3.54688500	-0.01471600
H	11.21794700	-3.04890000	-1.69879800
C	9.95134700	-1.80523800	-0.44315100
H	10.20191900	-0.74434000	-0.51758900
H	9.21318700	-1.99867000	-1.22748200
C	9.25860800	-2.06630000	0.89320000
H	8.84851300	-3.08561800	0.87828500
C	8.09807700	-1.07122300	1.13290200
H	7.71202300	-0.71091200	0.17238000
C	6.95167300	-1.66932800	1.94251700
H	7.33074800	-1.99389300	2.91913200
H	6.57653900	-2.55716200	1.42055600
C	5.80106800	-0.68516900	2.12939200
H	5.46928200	-0.33876800	1.14142900
C	4.60351900	-1.30912200	2.83792300
H	4.90575600	-1.67367100	3.82451700
H	4.25043800	-2.16011800	2.24832600
C	3.44647800	-0.32561500	3.02465500
C	2.16715600	-0.97219500	3.55053500
H	2.32724100	-1.34948500	4.56297000
H	1.88940500	-1.81102600	2.90502600
C	1.04415300	0.05161500	3.56166700
H	1.33649300	0.86534400	4.23380100

C	0.85674200	0.64066000	2.15567300
H	0.51798900	-0.14423300	1.47211500
C	2.20465600	1.19825900	1.65018200
H	2.49032000	2.01793600	2.31877300
C	-0.18712900	1.73305300	2.13356800
C	20.66848100	-3.50612200	0.23295500
H	20.27306900	-3.35174400	1.23953700
H	21.70842600	-3.17821600	0.20062900
H	20.62547000	-4.57356400	0.00623000
C	20.62346700	-4.16269600	-2.71095200
H	21.49400400	-4.59932000	-2.21217000
H	20.81123000	-4.17209700	-3.78853600
H	19.77106000	-4.81610800	-2.51194300
C	22.45916800	0.53127600	-2.63804500
H	22.52052400	0.51824100	-3.73192800
H	23.39918500	0.14826300	-2.23835400
H	22.33564400	1.56997900	-2.32332600
O	1.96052800	6.30779100	2.29722900
H	2.51317500	6.50007400	1.52694800
O	-1.42399100	7.32672500	-0.09615100
H	-1.87714400	7.47723500	0.75190500
O	1.71933000	5.50473100	-0.40220200
O	2.73166900	3.96150000	0.93008900
O	3.78189700	0.70198300	3.91335800
H	4.70271900	0.95268700	3.71694800
O	3.19376100	0.18191700	1.71759700

O	-0.77401800	2.10706400	1.13509100
O	-0.37503000	2.30601700	3.32157300
H	-1.07306900	3.00590700	3.21982200
O	6.22303200	0.44825500	2.89374800
H	7.16250700	0.60670100	2.68801200
O	8.63709500	0.07085400	1.80360300
H	9.36921700	-0.31579500	2.32653600
O	10.12935000	-1.96683900	2.01180300
H	11.02584300	-1.71843100	1.70028100
O	12.40648100	-1.22088500	0.80095300
H	13.31169900	-1.02999300	1.10731500
O	15.05655400	-1.36666400	0.81522700
H	15.97027500	-1.05562500	0.94435300
O	17.72459600	-1.48469500	0.41060200
O	18.50323200	-3.23047700	-0.78134500
O	22.69941200	-2.19480400	-1.79237000
H	23.05079200	-2.99765800	-2.19191200
O	-0.13690900	-0.57236700	4.02151300
H	-0.78218000	0.11843600	4.24137300
N	-0.91352600	6.76219700	2.58738600
H	-0.17990500	7.33317900	3.00295500
H	-1.26790800	6.14138300	3.30868200

AmB-Cyc6

0 1

C	12.94772800	-4.89184900	-2.97921000
C	13.31099200	-3.44012500	-2.61206600
C	13.21461800	-2.58326100	-3.89524000
C	11.53749500	-4.97493300	-3.60264700
C	11.79085200	-2.66321700	-4.48181800
C	11.66782300	-2.11344900	-5.91832600
O	10.54614200	-4.60778300	-2.63490300
O	12.97064300	-5.74225000	-1.82946300
O	14.65696100	-3.53879600	-2.15633000
O	11.41719200	-4.06073900	-4.68361700
O	11.38699700	-0.72325300	-5.88382100
H	13.98779800	-2.89276100	-4.63446100
H	11.32179300	-5.95902500	-4.07645900
H	13.70165100	-5.30987100	-3.69569300
H	12.67665300	-3.02873400	-1.79413000
H	11.04829800	-2.18835100	-3.79911600
H	12.56563900	-2.31456300	-6.52642100
H	10.77848100	-2.54824700	-6.42686700
H	13.76066000	-5.50461200	-1.27099500
H	15.04527100	-2.61449200	-2.03628000
H	12.16675900	-0.22572200	-5.51492700
C	14.51776900	-0.54560100	-4.12873300
C	15.51357300	0.01715100	-3.09142800
C	14.85539100	1.10474200	-2.22058400
C	14.32185600	2.21491800	-3.15530100

C	13.31654500	1.62625100	-4.16540100
C	12.96696800	2.57039100	-5.33453300
O	13.44677900	-1.22227100	-3.46033600
O	15.97707300	-1.07827900	-2.29653200
O	15.92893200	1.58233100	-1.41509400
O	13.93375900	0.51088000	-4.87863200
O	11.85666700	3.38265900	-4.98744400
H	15.16279000	2.73981900	-3.66231600
H	14.99993600	-1.17690800	-4.90805000
H	16.41268600	0.43237200	-3.61591500
H	14.05275900	0.70266000	-1.56102400
H	12.39814700	1.25867000	-3.65078100
H	13.82219800	3.19289800	-5.64626100
H	12.60201500	1.98782200	-6.20984600
H	16.41379500	-0.71662500	-1.47766800
H	15.63608500	2.40611200	-0.91102100
H	12.10590300	4.00615300	-4.25184400
C	14.03019100	4.49157500	-2.35705500
C	14.38219400	5.00842400	-0.94448200
C	13.13565100	5.06667400	-0.03940600
C	12.07815800	5.95524900	-0.73451000
C	11.72302800	5.36858300	-2.11525800
C	10.88720000	6.30627800	-3.01008200
O	13.60712100	3.12568700	-2.28563300
O	15.37850000	4.13659700	-0.40263300
O	13.61730500	5.67278400	1.15623900

O	12.94517200	5.23088600	-2.90336600
O	9.50393600	6.09206400	-2.77665500
H	12.43590800	7.00700700	-0.80993400
H	14.84430600	4.64736800	-3.09971900
H	14.85265600	6.02280500	-1.01903100
H	12.73101100	4.05748900	0.20247800
H	11.24238600	4.36723000	-2.01771400
H	11.14600600	7.36886800	-2.86880300
H	11.01628900	6.03890400	-4.08295300
H	15.44427100	4.29609300	0.57797700
H	12.83863900	5.94428700	1.73931600
H	9.26329100	6.38344200	-1.85485600
C	10.36881300	7.14266700	0.51867700
C	10.28250100	7.20828300	2.05846500
C	9.27747200	6.17736600	2.60762900
C	7.90334000	6.43514900	1.94664700
C	8.02223600	6.34001700	0.41187200
C	6.80127400	6.89159400	-0.35387800
O	10.90727900	5.87875100	0.11281900
O	11.59757600	6.97698800	2.57275400
O	9.23949700	6.46934200	4.00164800
O	9.07288100	7.24087000	-0.05502000
O	5.81861500	5.87839600	-0.49820800
H	7.48965600	7.41662400	2.27169700
H	10.91417500	8.00218700	0.06880000
H	9.99003100	8.24132000	2.37921100

H	9.61270500	5.12568200	2.45927400
H	8.27032900	5.30161500	0.09168500
H	6.37068800	7.78580000	0.12746500
H	7.07852700	7.13996600	-1.40270400
H	11.52981500	6.79438900	3.54979500
H	8.52809200	5.91106500	4.44727000
H	5.45115500	5.63073000	0.39379200
C	5.86654600	5.76456300	3.08640700
C	5.77095800	5.05866100	4.45695700
C	5.54896500	3.54105800	4.29756200
C	4.26677100	3.33552200	3.45823300
C	4.44590800	3.99386100	2.07529600
C	3.15286200	4.07810800	1.23879600
O	7.05166700	5.35279100	2.39756300
O	6.97471700	5.33331300	5.17768900
O	5.38005900	3.08915000	5.63668900
O	4.76321500	5.40720700	2.26138100
O	2.97662300	2.88120600	0.49623600
H	3.37509700	3.73292100	3.99394800
H	5.78000700	6.87261300	3.15551300
H	4.93599300	5.50904300	5.05442200
H	6.42474600	3.02107600	3.84693800
H	5.26964600	3.51090600	1.50011100
H	2.26310800	4.28771100	1.85564700
H	3.24632500	4.86156500	0.45370600
H	7.05518800	4.68481300	5.92886000

H	5.02469200	2.14310000	5.63862400
H	2.81377400	2.11678100	1.11230600
C	2.90763300	1.33921600	3.71510000
C	3.16690500	0.22264500	4.74574800
C	3.95716100	-0.93806800	4.11602900
C	3.14899900	-1.47860800	2.91436600
C	2.86381700	-0.35489600	1.89716500
C	1.75877600	-0.69868700	0.87645000
O	4.14821500	1.90441600	3.27440600
O	3.86664700	0.79638700	5.85178100
O	4.02064000	-1.90098700	5.16381400
O	2.27020300	0.79935100	2.56485000
O	2.30318800	-1.39524000	-0.23268000
H	2.20741400	-1.97944600	3.26697900
H	2.18888700	2.11264900	4.06737900
H	2.17888500	-0.15550500	5.15397700
H	4.98971300	-0.64677000	3.81581500
H	3.80312600	-0.02966700	1.39112800
H	0.93380700	-1.27579900	1.32749000
H	1.34649800	0.23079900	0.42283300
H	4.24292300	0.05840800	6.40790300
H	4.45368900	-2.74549900	4.82653300
H	2.70571000	-2.25708800	0.06139400
C	3.51906900	-3.76194800	2.15868600
C	4.52391500	-4.77395400	2.75317500
C	5.83141200	-4.81058200	1.93687600

C	5.46357900	-5.15697800	0.47510700
C	4.50712600	-4.08755900	-0.08998300
C	3.88239200	-4.44769700	-1.45255800
O	4.02987000	-2.43246300	2.27090400
O	4.75882100	-4.40269300	4.11352500
O	6.58332900	-5.85298500	2.54801000
O	3.32635700	-4.01312900	0.77033200
O	4.70498100	-3.96514200	-2.50323100
H	5.02509000	-6.17882100	0.41163800
H	2.49438000	-3.86107200	2.58030500
H	4.06127100	-5.79432200	2.78068100
H	6.41012800	-3.86099300	2.00393800
H	4.99208900	-3.08380100	-0.12198800
H	3.70364000	-5.52997400	-1.56492200
H	2.92410500	-3.90078900	-1.59930800
H	5.59598300	-4.84213100	4.42759900
H	7.33942500	-6.13351300	1.93714900
H	5.57737900	-4.44783000	-2.50778000
C	6.99784300	-6.25047400	-1.05683300
C	8.34548800	-6.87099700	-0.63030500
C	9.52314500	-5.92423700	-0.93288400
C	9.49323800	-5.58211800	-2.44085100
C	8.14855100	-4.92085300	-2.80617000
C	7.89100600	-4.80084700	-4.32234200
O	6.71207300	-5.09863900	-0.25099900
O	8.25772800	-7.16154100	0.76871900

O	10.66661400	-6.70653800	-0.60332200
O	7.05404200	-5.80091600	-2.40201500
O	8.44132700	-3.58786700	-4.81084000
H	9.68747600	-6.49149000	-3.05394400
H	6.15881100	-6.98150600	-1.06494200
H	8.49561900	-7.85095900	-1.15259500
H	9.51262700	-5.00579700	-0.30306300
H	8.02839600	-3.93846000	-2.29408100
H	8.28386300	-5.66281500	-4.88732200
H	6.80192800	-4.69664400	-4.52636000
H	9.17233200	-7.36904100	1.10701600
H	11.50507800	-6.22705800	-0.89823300
H	9.43508700	-3.61729000	-4.75394200
C	-20.57690100	5.41698400	-0.83308700
H	-21.43060400	5.26420500	-1.54516600
C	-19.61286700	6.46952200	-1.43867700
H	-18.88332200	6.83046800	-0.68091300
C	-18.89918000	5.92717100	-2.70115900
H	-19.63483500	5.68219100	-3.49830800
C	-19.03306100	3.64797900	-1.85498100
H	-19.63216600	3.25024900	-2.70065700
C	-19.85390900	4.06038300	-0.61083700
H	-20.58255600	3.25944900	-0.34407900
C	-17.80821600	6.85794800	-3.19638200
H	-16.96751100	6.90216600	-2.49133900
H	-18.20477700	7.87750900	-3.31660200

H	-17.40486400	6.52640500	-4.15976000
C	-17.09601500	2.21540500	-2.28276800
H	-16.66822800	3.10830700	-2.79588000
C	-16.11743200	1.52815400	-1.37104700
H	-16.55448000	0.74372600	-0.74579300
C	-14.81707900	1.84680000	-1.33408000
H	-14.40688800	2.64793000	-1.95484800
C	-13.86570200	1.14567000	-0.47348600
H	-14.26704700	0.31304200	0.11840100
C	-12.56524600	1.48128100	-0.39254500
H	-12.15445900	2.31941000	-0.95901800
C	-11.63196100	0.74200800	0.45296000
H	-12.05302600	-0.13141500	0.97543600
C	-10.33754300	1.08095900	0.59405900
H	-9.92139300	1.95829400	0.09441600
C	-9.41742200	0.30438300	1.41926100
H	-9.80840300	-0.63991800	1.82649600
C	-8.15341200	0.69088400	1.67409100
H	-7.75370200	1.62749300	1.28068700
C	-7.25107000	-0.11113200	2.49434000
H	-7.66999600	-1.04338200	2.89229700
C	-5.98218000	0.25131800	2.76058300
H	-5.56583300	1.18495700	2.37606500
C	-5.09170400	-0.57371700	3.56947300
H	-5.53545700	-1.47399600	4.01296100
C	-3.79328300	-0.28708300	3.77701700

H	-3.32654700	0.60839700	3.36541500
C	-17.72718800	1.25604300	-3.30377600
H	-18.37645700	1.83287900	-3.99624200
H	-16.92132100	0.81877400	-3.93366500
C	-2.93807600	-1.18460900	4.55259700
H	-3.46979300	-1.97708300	5.09953400
C	-1.60124700	-1.10220200	4.58312900
H	-1.05292600	-0.31939000	4.06287000
C	-0.75671300	-2.09335200	5.33948900
H	-1.40160700	-2.73928400	5.99591000
C	-0.01988700	-3.03840400	4.35230400
H	0.82311600	-2.50329400	3.84238300
C	-0.94493100	-3.68794300	3.29284300
H	-1.50692600	-2.85633600	2.78172000
C	-2.00891000	-4.58647700	3.95396700
H	-2.48738800	-4.06380100	4.81816800
C	-4.29931300	-4.29903200	3.12064700
C	-5.18542200	-4.62503300	1.95534900
H	-4.60198200	-4.64343200	1.01015300
H	-5.56300700	-5.66689600	2.08338700
C	-6.37199200	-3.64567100	1.85510200
H	-6.01043600	-2.59484000	1.75579400
C	-7.33040900	-4.04656900	0.73043900
H	-6.76483900	-4.24767400	-0.19947800
H	-7.82244000	-5.00741300	1.00295700
C	-8.41356700	-2.98797300	0.46579800

H	-7.97615900	-1.96965900	0.33520800
C	-9.27356900	-3.38860500	-0.74635500
H	-9.56964700	-4.45504700	-0.65618600
H	-8.65351800	-3.32520900	-1.66146600
C	-10.52328800	-2.51447500	-0.90395900
H	-10.44503400	-1.61194500	-0.25521900
H	-10.58927200	-2.12555500	-1.93784900
C	-11.80304100	-3.28352100	-0.55724600
H	-11.92532200	-4.18202300	-1.20364200
C	-13.06989400	-2.37516400	-0.60377000
H	-12.90579800	-1.43972400	-1.18510800
C	-14.28848900	-3.15514300	-1.11424100
H	-14.45279900	-4.04521500	-0.46875900
H	-14.09236100	-3.54561700	-2.12964400
C	-15.55884600	-2.29277100	-1.11055100
H	-15.39088400	-1.29845400	-1.59060900
C	-16.74900000	-3.02083100	-1.75216900
H	-17.00797100	-3.91440200	-1.14465600
H	-16.46692800	-3.38344400	-2.75874000
C	-17.99185200	-2.12745600	-1.84887200
C	-19.16307800	-2.74888900	-2.61486500
H	-19.58973400	-3.59879900	-2.04242500
H	-18.81732200	-3.17849800	-3.57786000
C	-20.26292000	-1.72013900	-2.88388700
H	-20.85367800	-1.49503300	-1.96818000
C	-19.69625400	-0.40980500	-3.50383800

H	-19.30270200	-0.67188200	-4.52630900
C	-18.51466800	0.12343400	-2.64266600
H	-18.87034200	0.39167800	-1.61856300
C	-20.75501600	0.63674800	-3.67836400
C	-1.59406600	-5.99338000	4.32478600
H	-2.41622400	-6.53931700	4.80592700
H	-0.75513700	-5.96367800	5.03912700
H	-1.28691800	-6.58424200	3.45374100
C	-0.13908700	-4.41631100	2.21880300
H	0.50915700	-5.19503800	2.63200600
H	0.47362100	-3.72706700	1.62644600
H	-0.82013600	-4.91526700	1.51098200
C	0.25028400	-1.38104800	6.24419000
H	0.92605100	-0.71380600	5.68595000
H	0.87745500	-2.11725400	6.77092700
H	-0.26029500	-0.77496900	7.00161400
O	-19.03415000	4.19502300	0.55249000
H	-18.07993700	4.04683200	0.31748300
O	-20.36922600	7.58891300	-1.92284700
H	-20.99067800	7.89453000	-1.21784000
O	-18.18917400	4.71509300	-2.33153300
O	-18.11942300	2.68520300	-1.34491100
O	-18.50187000	-1.73860600	-0.59274700
H	-17.76798500	-1.54014600	0.06614800
O	-17.53685200	-0.93355400	-2.52476600
O	-20.65635600	1.83015500	-3.85664400

O	-22.02340900	0.08919300	-3.64131800
H	-22.74082600	0.76474100	-3.78119100
O	-15.97612200	-2.04639500	0.24866000
H	-15.19633400	-1.75249500	0.80327900
O	-13.33763700	-1.89058900	0.72235900
H	-13.11219200	-2.61856900	1.38669700
O	-11.77670100	-3.81333500	0.77713700
H	-10.95591500	-3.48783400	1.27375800
O	-9.30480600	-2.85699900	1.59600100
H	-8.89604500	-3.23136800	2.42571500
O	-7.17271100	-3.72967900	3.04793200
H	-6.63111500	-3.52406600	3.85845300
O	-4.62008900	-3.72142900	4.14312100
O	-3.04218100	-4.75992500	2.89364600
O	0.54883200	-4.03926100	5.21775800
H	1.13870800	-4.63475200	4.72017800
O	-21.09758800	-2.36501900	-3.85457100
H	-21.95429100	-1.88662800	-3.95474700
N	-21.11661000	5.98303700	0.43042300
H	-20.37913800	6.00443800	1.14074600
H	-21.85289400	5.38733500	0.80132700

AmB-Cyc7

0 1

C	4.39130700	-7.17157100	-1.49367800
C	4.40764100	-6.07402100	-2.61253600
C	2.97513300	-5.75906800	-3.11458600
C	2.25308900	-7.08117400	-3.50096100
C	2.37010200	-8.14239500	-2.35603700
C	1.82373600	-9.55218600	-2.76914600
O	3.76405300	-6.67319700	-0.29547100
O	5.03862900	-4.89777400	-2.09437000
O	3.10539300	-4.85410600	-4.21397700
O	3.74161100	-8.36051200	-1.96152400
O	0.41558400	-9.70640300	-2.60124400
H	2.70847300	-7.47787100	-4.41462600
H	5.42896500	-7.47524100	-1.29339000
H	4.98666400	-6.47923100	-3.45301200
H	2.41007200	-5.30506000	-2.28863500
H	1.76790400	-7.78385700	-1.51501700
H	2.15160200	-9.77783800	-3.79158600
H	2.30589800	-10.27891300	-2.10846700
H	4.94385700	-4.22340900	-2.80820200
H	2.27284400	-4.95722300	-4.74508700
H	-0.00520000	-9.15056300	-3.30539300
C	0.26398300	-7.24301300	-4.87242800
C	-0.13810000	-6.05201200	-5.80131100
C	-1.26181500	-5.19913600	-5.15228800
C	-2.40446100	-6.11399300	-4.62206600
C	-1.83959100	-7.28386600	-3.74870800

C	-2.94355000	-8.29954100	-3.29370100
O	0.88181800	-6.70442800	-3.70152200
O	1.05277600	-5.27426900	-6.00600800
O	-1.70951200	-4.26511300	-6.13712100
O	-0.85794500	-8.05102300	-4.48655700
O	-3.63766600	-7.90554800	-2.11163600
H	-2.95097300	-6.53127800	-5.47476800
H	0.93600800	-7.91477900	-5.42600300
H	-0.50602800	-6.45680300	-6.75273900
H	-0.83472300	-4.68474700	-4.27985700
H	-1.38135600	-6.84993700	-2.85210400
H	-3.62023700	-8.49292900	-4.13524800
H	-2.43518300	-9.24128200	-3.06601000
H	0.73696100	-4.47811800	-6.49890900
H	-2.62919400	-4.00733600	-5.86396400
H	-4.23252200	-7.15947000	-2.38023900
C	-4.63416600	-5.37134400	-4.03795300
C	-5.17147300	-4.02120000	-4.61564400
C	-5.11377200	-2.88015700	-3.56252100
C	-5.75372900	-3.38365200	-2.23254500
C	-5.06787900	-4.71745800	-1.79211300
C	-5.63158500	-5.24525300	-0.43807000
O	-3.22495600	-5.24228900	-3.82862400
O	-4.36168500	-3.70628000	-5.76008300
O	-5.77538300	-1.76076700	-4.14315100
O	-5.25859700	-5.74068300	-2.79854800

O	-4.87581500	-4.62946100	0.60731900
H	-6.81748700	-3.58023800	-2.40006200
H	-4.87430500	-6.18032600	-4.74304900
H	-6.21791700	-4.16432900	-4.91404600
H	-4.05709400	-2.66395200	-3.34941000
H	-3.99755700	-4.52838700	-1.65035600
H	-6.70175400	-5.02166500	-0.37295100
H	-5.52354500	-6.33798500	-0.42094500
H	-4.64267400	-2.78921800	-5.99805800
H	-5.90069700	-1.10079100	-3.40234200
H	-5.48867400	-4.54924600	1.38012200
C	-6.74363900	-1.75680200	-0.75222500
C	-6.60588200	-0.20320600	-0.93555600
C	-5.59183000	0.41064000	0.07848100
C	-5.81950100	-0.16649800	1.52118200
C	-5.92037800	-1.72426100	1.46496000
C	-6.23396900	-2.32542400	2.86922600
O	-5.54454000	-2.37712800	-1.22909400
O	-6.24179800	0.00361000	-2.29982100
O	-5.76368900	1.83308200	0.03524900
O	-7.00010600	-2.13340600	0.60468700
O	-5.86960200	-3.71294300	2.96876400
H	-6.76005100	0.22948300	1.91814900
H	-7.62593200	-2.08779700	-1.31855700
H	-7.58806200	0.24195600	-0.72148100
H	-4.57693400	0.13076400	-0.23078400

H	-4.96424100	-2.12340100	1.10633100
H	-5.73543000	-1.72651900	3.63671300
H	-7.31362900	-2.25767200	3.03364600
H	-5.79453300	0.89162900	-2.40013300
H	-4.94985800	2.20997200	0.46771400
H	-4.92729200	-3.69905500	3.26793800
C	-4.89601000	1.36262900	3.13880600
C	-3.75522700	2.38750200	2.81545200
C	-2.37472500	1.90527900	3.34184300
C	-2.50399200	1.46198300	4.82966600
C	-3.69785300	0.46276900	4.99443600
C	-3.95835700	0.05171400	6.48224400
O	-4.70644700	0.16215700	2.37468700
O	-3.70961800	2.57242100	1.39470200
O	-1.46174600	2.98811900	3.14855800
O	-4.92811000	1.06235400	4.54133000
O	-3.07343400	-0.95311200	6.97614200
H	-2.69380200	2.34719600	5.44552000
H	-5.86369000	1.83986300	2.92229500
H	-4.00667200	3.33126400	3.31586000
H	-2.07431200	1.02114000	2.76234900
H	-3.46578800	-0.44100200	4.41856000
H	-3.95667000	0.95373000	7.10623100
H	-4.97043400	-0.36238100	6.51958100
H	-3.04001400	3.27999600	1.24630000
H	-0.71647900	2.83287500	3.78458000

H	-2.21146100	-0.49229400	7.13330000
C	-0.65632600	1.30536800	6.38178200
C	0.72494800	1.95416900	6.04774800
C	1.73403200	0.88826500	5.54488300
C	1.78426700	-0.29743800	6.55424100
C	0.34111100	-0.82906100	6.85406400
C	0.31525600	-1.91150000	7.98711600
O	-1.25555300	0.83138100	5.17343100
O	0.49429100	2.96057900	5.05189700
O	2.98297500	1.56182100	5.38401600
O	-0.53423800	0.22435500	7.31957500
O	0.71573700	-3.21273000	7.56362300
H	2.24130600	0.04987300	7.48674000
H	-1.28350400	2.05422800	6.88712400
H	1.12498200	2.40458200	6.96540000
H	1.36793200	0.48693800	4.58950700
H	-0.05450100	-1.27346700	5.93260500
H	0.90760600	-1.54589400	8.83510800
H	-0.72158800	-1.99048500	8.32700100
H	1.40248100	3.23057300	4.77111900
H	3.67825000	0.85361500	5.36856700
H	1.69854500	-3.16757800	7.45436200
C	3.65928900	-1.82505700	6.70260200
C	4.99242400	-1.56478700	5.92977500
C	5.04623700	-2.40450900	4.62638700
C	4.74769800	-3.89683200	4.95522500

C	3.42675900	-4.03193700	5.78584100
C	3.16633900	-5.49519900	6.28445300
O	2.56620300	-1.32977900	5.92499900
O	5.05697200	-0.15979200	5.64723900
O	6.33834300	-2.19548900	4.05413100
O	3.46204100	-3.21965800	6.98322000
O	2.59529900	-6.35312600	5.29951600
H	5.58244200	-4.30606600	5.53399000
H	3.72030400	-1.33743100	7.68663500
H	5.82828000	-1.86627600	6.57430000
H	4.24920000	-2.05339700	3.95612200
H	2.59059200	-3.72625200	5.14532000
H	4.09820500	-5.89647800	6.70190600
H	2.44682200	-5.42705600	7.10567200
H	5.82551000	-0.07345800	5.03247700
H	6.48380500	-2.95027900	3.42551700
H	3.32928100	-6.56446400	4.66934600
C	5.38076200	-5.74024500	3.51658800
C	6.34280100	-5.54112100	2.30266800
C	5.57028600	-5.45845000	0.95629200
C	4.63293800	-6.70942300	0.85635300
C	3.71867700	-6.72614300	2.12791500
C	2.69628300	-7.90241900	2.09061600
O	4.60333600	-4.55228500	3.68298800
O	7.08210000	-4.33273100	2.53005800
O	6.58054000	-5.35006000	-0.04399700

O	4.50534400	-6.86411900	3.33187000
O	1.60403600	-7.60835900	1.21730000
H	5.24377800	-7.61942700	0.85101400
H	5.97711400	-5.97406500	4.41052900
H	7.02088800	-6.40308200	2.25624500
H	4.92817100	-4.56814600	0.97765300
H	3.15253300	-5.78752400	2.15736000
H	3.21790600	-8.82453800	1.80445300
H	2.29047200	-8.04718100	3.09575100
H	7.52447000	-4.16861400	1.66143600
H	6.09539900	-5.08363600	-0.86813300
H	2.06076100	-7.30248800	0.39536500
C	-9.36014300	4.03800600	2.48029400
H	-10.09118700	4.75580100	2.10287700
C	-8.66743500	4.65516200	3.72587600
H	-7.97040800	3.90916100	4.13077200
C	-7.84145100	5.90896500	3.30472300
H	-8.53118200	6.64373400	2.86732000
C	-7.42025500	5.03269700	1.14717700
H	-8.04790500	5.81035300	0.67510500
C	-8.30755600	3.75288800	1.36794100
H	-8.83688300	3.57652500	0.42081200
C	-7.12807900	6.55069500	4.51336000
H	-6.42083000	5.85056000	4.94676900
H	-7.85932700	6.82304400	5.26773300
H	-6.59006500	7.44099400	4.20248700

C	-5.76993300	5.72644900	-0.44539900
H	-5.88437400	6.67295600	0.09390000
C	-4.28087100	5.41670700	-0.59463700
H	-4.05580500	4.51331900	-1.14810400
C	-3.30139800	6.13368000	-0.08102300
H	-3.50813200	7.03788200	0.47989500
C	-1.87923100	5.73803000	-0.21361100
H	-1.70066400	4.83059200	-0.77901600
C	-0.84127600	6.37336900	0.30829900
H	-0.97274100	7.28441000	0.88042000
C	0.53568300	5.85647800	0.15862100
H	0.61403000	4.92987600	-0.40029100
C	1.63470000	6.41226700	0.64808400
H	1.58814900	7.34170000	1.20302200
C	2.96702000	5.79678800	0.47716200
H	2.96924600	4.83578800	-0.02582300
C	4.11970100	6.30988200	0.88298800
H	4.15210800	7.27128000	1.38177600
C	5.40842800	5.61657100	0.67838300
H	5.33723700	4.62702700	0.24028100
C	6.60467500	6.10394000	0.97362000
H	6.70563700	7.09367600	1.40345800
C	7.85367800	5.35392900	0.72143200
H	7.72251400	4.33994000	0.36024800
C	9.07927700	5.82817400	0.88069900
H	9.23505900	6.84248800	1.22989000

C	-6.49520600	5.83221000	-1.82408100
H	-7.51144400	6.17789900	-1.66011200
H	-5.98741100	6.56111800	-2.45115300
C	10.29291700	5.03688200	0.56536200
H	10.11319500	4.00155500	0.29897000
C	11.52294000	5.50867700	0.56014300
H	11.71000100	6.54331300	0.82653000
C	12.75561800	4.68340700	0.20113200
H	12.44021800	3.66897800	-0.03661400
C	13.50495700	5.25951300	-1.04688000
H	13.95049600	6.21828900	-0.74961000
C	12.56579400	5.57759800	-2.26290400
H	11.81834400	6.27806000	-1.89228900
C	11.76630400	4.34336000	-2.78503200
H	11.27297400	3.86132500	-1.93777100
C	9.54027100	4.21707600	-3.61287700
C	8.47066200	4.88412800	-4.50854600
H	8.68919100	5.94498700	-4.59844300
H	8.51719200	4.43981600	-5.50179700
C	7.06400000	4.65967700	-3.88342900
H	7.05315200	5.15660000	-2.90250800
C	5.92702800	5.26863700	-4.74439000
H	6.05841200	6.34625700	-4.79747100
H	5.97585800	4.86864400	-5.75475800
C	4.53983800	4.93676100	-4.12470400
H	4.55290500	5.26649400	-3.07558400

C	3.38768200	5.67944500	-4.85007200
H	3.31384300	5.31072300	-5.87140400
H	3.65182700	6.73322100	-4.90233600
C	2.01450100	5.56062700	-4.14303500
H	2.13670000	5.73216600	-3.07502000
H	1.37673100	6.35534700	-4.52338600
C	1.25558800	4.21748400	-4.36479500
H	1.16155500	4.05762400	-5.44716000
C	-0.18581800	4.29354600	-3.72754200
H	-0.44736000	5.33810800	-3.52713900
C	-1.28735800	3.69353100	-4.64787300
H	-1.00450800	2.69078600	-4.96129700
H	-1.39315700	4.31079300	-5.53645100
C	-2.63273500	3.63733000	-3.87445800
H	-2.86035400	4.65283000	-3.52212100
C	-3.82354500	3.16786800	-4.74871300
H	-3.61399500	2.18739200	-5.17017400
H	-3.97588900	3.86773600	-5.56558900
C	-5.13418800	3.07822100	-3.90083000
C	-6.33316000	2.64737200	-4.79999200
H	-6.20371000	1.61172400	-5.10340500
H	-6.33289700	3.26394600	-5.69508600
C	-7.69746000	2.80902200	-4.08996800
H	-7.76271300	2.06970300	-3.28197800
C	-7.79322900	4.22979600	-3.44450700
H	-7.80317000	4.97969100	-4.23686100

C	-6.52631200	4.46038600	-2.55420900
H	-6.50022300	3.66720800	-1.79768700
C	-9.11688400	4.39371400	-2.65012100
C	12.60857600	3.27966700	-3.52800300
H	11.99621900	2.41044800	-3.74669500
H	13.44021500	2.97674000	-2.90056000
H	12.99408700	3.67446500	-4.46174400
C	13.33425200	6.29298100	-3.39786500
H	14.13449500	5.67781900	-3.79550400
H	13.76702900	7.21878400	-3.03079300
H	12.65730300	6.53245100	-4.21154900
C	13.73297200	4.60861400	1.40005100
H	14.04897800	5.60308600	1.70093200
H	14.61256800	4.03423700	1.12702400
H	13.25391200	4.12997700	2.24875600
O	-7.55607400	2.60759200	1.75293700
H	-6.93614600	2.41478800	0.99409100
O	-9.63474800	5.04155100	4.70787400
H	-10.23326700	4.25309800	4.74199700
O	-6.82709700	5.54481900	2.35110400
O	-6.34216700	4.62897100	0.30362900
O	-4.97550500	2.15171200	-2.82637900
H	-4.02626600	2.25244200	-2.54440300
O	-5.34739600	4.40472000	-3.37773800
O	-9.46422600	5.34826600	-1.97977800
O	-9.96341500	3.29339100	-2.79143900

H	-10.77752600	3.52850200	-2.28200800
O	-2.53468600	2.74225500	-2.76023100
H	-1.62704700	2.91834400	-2.39002200
O	-0.16257300	3.58984500	-2.47543500
H	0.66972000	3.05292800	-2.57617200
O	1.85031800	3.08990900	-3.72873600
H	2.83681800	3.15965500	-3.87590700
O	4.28862800	3.52825000	-4.19167200
H	5.13526700	3.10732800	-3.89355500
O	6.79007900	3.26186600	-3.72128700
H	7.58042400	2.91206800	-3.23820900
O	9.32077700	3.22960000	-2.92405000
O	10.74692200	4.87924500	-3.66841200
O	14.53934900	4.31378500	-1.36250700
H	15.14357900	4.80124000	-1.97568400
O	-8.71427000	2.58288900	-5.07262000
H	-9.54545500	2.56936500	-4.53731900
N	-10.09655200	2.82619400	2.92990600
H	-9.38532100	2.08302300	3.03156200
H	-10.68767000	2.50932300	2.14409200

AmB-DSPE

AmB-DSPE1

0 1

C	-10.55684100	3.77738100	0.23695300
H	-11.02910500	3.57178400	-0.74070500
C	-10.08563500	5.22148500	0.20005100
H	-9.57838100	5.43821400	1.14697200
C	-9.10150100	5.41684300	-0.96365700
H	-9.66191800	5.22656300	-1.89511300
C	-8.41337700	3.15269800	-0.84080200
H	-8.93065900	2.90660300	-1.78043700
C	-9.33265300	2.87916100	0.33828500
H	-9.64211700	1.82986300	0.27909900
C	-8.51407000	6.81162400	-1.00125300
H	-7.96298400	7.00991700	-0.07763900
H	-9.30520900	7.55618600	-1.11748500
H	-7.82196100	6.90863000	-1.83995000
C	-6.38925300	2.29191400	-1.77154600
H	-6.31422300	3.28801100	-2.22284400
C	-5.05124500	1.88640000	-1.23489000
H	-4.99043700	0.90487300	-0.77291900
C	-3.96428300	2.65972300	-1.31866900
H	-4.04496000	3.65254700	-1.76079400
C	-2.65147200	2.24811300	-0.86207600
H	-2.56176600	1.22320700	-0.50863200
C	-1.55961600	3.03730200	-0.87041900
H	-1.64879400	4.06692200	-1.21634900
C	-0.25369600	2.57691900	-0.46350900
H	-0.18728500	1.53156300	-0.17441600

C	0.86900200	3.32459100	-0.46376000
H	0.82034200	4.37356100	-0.75476400
C	2.16651000	2.78521200	-0.13540900
H	2.20420500	1.73091000	0.13153700
C	3.32308300	3.47855400	-0.17079400
H	3.30066000	4.53659900	-0.43054700
C	4.61444900	2.89346500	0.09621700
H	4.63300900	1.83111500	0.33143300
C	5.77805600	3.57302200	0.04653400
H	5.75792900	4.63581900	-0.19371000
C	7.07354100	2.98087300	0.28125700
H	7.09081200	1.92238000	0.53304100
C	8.23747100	3.65137300	0.18980300
H	8.21949300	4.71051500	-0.06629800
C	-6.94003300	1.30666300	-2.81157700
H	-7.94828100	1.62144100	-3.08408900
H	-6.32507800	1.35929700	-3.71514900
C	9.53795100	3.04190900	0.38490900
H	9.54063400	1.99121500	0.66514700
C	10.70419400	3.67463400	0.20845900
H	10.69825500	4.72724000	-0.08064200
C	12.05441600	3.03488000	0.35239700
H	11.92140400	2.02251400	0.74141100
C	12.76683900	2.91113200	-1.00773000
H	13.04078900	3.92852900	-1.33211000
C	11.90654400	2.30055200	-2.12860000

H	10.99425700	2.90271100	-2.16967600
C	11.44618400	0.87781600	-1.79365700
H	11.16706500	0.82064500	-0.74471100
C	9.16594700	0.16531500	-1.93801700
C	7.97880600	-0.04022700	-2.84230500
H	8.12626900	0.50704500	-3.77467900
H	7.93462700	-1.11006400	-3.07738000
C	6.66354000	0.37030200	-2.17007900
H	6.70183300	1.44400400	-1.93688200
C	5.47655300	0.08959700	-3.07735300
H	5.61011700	0.64368300	-4.01257600
H	5.46461700	-0.97939000	-3.32213400
C	4.12303000	0.46403400	-2.47794200
H	4.14497600	1.51341900	-2.15448800
C	3.01367100	0.28744700	-3.50745300
H	3.10860100	-0.70880900	-3.95734900
H	3.19827100	1.00980300	-4.30857200
C	1.59352400	0.47383200	-2.95235200
H	1.61123400	1.06735800	-2.03585000
H	0.98954400	1.04051200	-3.66759000
C	0.85919700	-0.83647000	-2.69088800
H	0.77598500	-1.37796400	-3.64412500
C	-0.57064900	-0.59707800	-2.14479800
H	-0.90590000	0.40965200	-2.41907800
C	-1.57691400	-1.61537600	-2.66638400
H	-1.27559000	-2.61916000	-2.34206300

H	-1.55337900	-1.60180600	-3.76213800
C	-3.00156700	-1.32366600	-2.20839500
H	-3.27378300	-0.30671200	-2.52277500
C	-3.99901600	-2.29450800	-2.82870300
H	-3.74584000	-3.31780700	-2.53521200
H	-3.92611300	-2.21963900	-3.91799400
C	-5.44413700	-2.02915400	-2.41531700
C	-6.45865200	-2.88544200	-3.16000400
H	-6.29366100	-3.94418400	-2.94570800
H	-6.35095200	-2.72660400	-4.23643200
C	-7.86081300	-2.49916000	-2.72837700
H	-7.96286700	-2.71341800	-1.66185200
C	-8.10385500	-0.98630600	-2.92381800
H	-8.18794900	-0.80328300	-4.00416900
C	-6.94736400	-0.14652800	-2.33794000
H	-7.02802100	-0.18723500	-1.24784800
C	-9.40085400	-0.55313900	-2.26268500
C	12.40894600	-0.24328600	-2.12388400
H	11.98726000	-1.19772200	-1.80868800
H	13.34712200	-0.07802600	-1.59284200
H	12.60779300	-0.29194400	-3.19646600
C	12.59756700	2.40046800	-3.48684800
H	13.56212700	1.88364200	-3.49425800
H	12.77369200	3.44648200	-3.75301500
H	11.98144600	1.95369700	-4.27089600
C	12.93581200	3.80232300	1.34125500

H	13.06769800	4.84173000	1.02072000
H	13.92038700	3.33699300	1.41039200
H	12.47904700	3.81399600	2.33368100
O	-8.64997600	3.15434900	1.54614600
H	-7.71707400	2.92672500	1.41113000
O	-11.23317200	6.04575900	0.02041700
H	-11.04735900	6.90966100	0.40148400
O	-8.01155800	4.51156900	-0.86145500
O	-7.28316300	2.37126700	-0.65111800
O	-5.64677400	-2.27759800	-1.05625800
H	-4.98419600	-1.77070400	-0.55569200
O	-5.68638400	-0.67003300	-2.74459700
O	-9.57007500	0.46499600	-1.64243800
O	-3.11232800	-1.40649000	-0.78503900
H	-2.25343100	-1.14307700	-0.40817700
O	-0.52639400	-0.63111700	-0.71720800
H	0.23353400	-1.21794900	-0.52748900
O	1.52568000	-1.67812400	-1.76345100
H	2.39211500	-1.28631500	-1.52053200
O	3.81482700	-0.35007000	-1.34232000
H	4.64329200	-0.52818400	-0.86286900
O	6.45817900	-0.36338500	-0.96749000
H	7.30545500	-0.39272200	-0.48866900
O	9.13652100	-0.10621800	-0.74650200
O	10.22466200	0.64788800	-2.56723700
O	13.94897900	2.14885700	-0.77092700

H	14.56826200	2.30084700	-1.49273400
O	-8.77056600	-3.29748500	-3.48596900
H	-9.63341700	-2.86710500	-3.40694900
N	-11.46677100	3.55511500	1.35158000
H	-11.95571500	2.67516900	1.21485500
H	-12.16782600	4.29022200	1.33646700
C	14.61552500	-0.82078800	2.25902000
H	14.85946000	-0.88702800	3.32645900
H	14.49603800	-1.85373300	1.90909800
C	13.29614100	-0.07805700	2.08050900
H	13.07494900	-0.01611500	1.00690300
H	13.41742800	0.96122100	2.41288800
C	12.11048500	-0.70808200	2.80633300
H	12.28118200	-0.68058800	3.89012600
H	12.03576900	-1.76841400	2.53504600
C	10.79737700	-0.01199300	2.46629900
H	10.86591000	1.05066400	2.73605200
H	10.65647000	-0.04051000	1.37828200
C	9.55810800	-0.61531800	3.11996200
H	9.63623000	-0.54617000	4.21250000
H	9.50572200	-1.68510900	2.88008000
C	8.28284400	0.06991100	2.63962200
H	8.33707800	1.14373500	2.86301700
H	8.25193800	-0.00421700	1.54656700
C	6.98991000	-0.49784500	3.21333900
H	6.97190900	-0.35514500	4.30136100

H	6.95795300	-1.58210000	3.04522000
C	5.75600300	0.14525100	2.58737700
H	5.81866000	1.23623800	2.69462800
H	5.76708300	-0.04854200	1.50654900
C	4.43512100	-0.33915300	3.17290200
H	4.40630200	-0.10178700	4.24426300
H	4.37817400	-1.43271900	3.10054200
C	3.22126800	0.27748700	2.48483400
H	3.32847000	1.36981300	2.46736100
H	3.20336900	-0.03879100	1.43233500
C	1.89991000	-0.08120200	3.15541200
H	1.93229900	0.25137200	4.20129500
H	1.78631900	-1.17236300	3.18687600
C	0.68453900	0.53522600	2.47044500
H	0.58290600	0.11903000	1.46070500
H	0.84562100	1.61231400	2.33525900
C	-0.60987800	0.31507700	3.24537900
H	-0.49846900	0.74161300	4.25108900
H	-0.77125900	-0.76101900	3.39033000
C	-1.84314700	0.91973900	2.57982600
H	-2.00086100	0.43823900	1.60651300
H	-1.66673300	1.98118700	2.36359800
C	-3.09130800	0.75245100	3.43900800
H	-2.91980100	1.19497900	4.42740700
H	-3.26977100	-0.31119600	3.61687700
C	-4.34885600	1.35942000	2.82904500

H	-4.48222700	1.04225900	1.78743800
H	-4.31972400	2.45267500	2.80780500
C	-5.59750100	0.90533700	3.53560100
O	-5.66918600	-0.04726600	4.27663800
C	15.75983200	-0.15059900	1.50441600
H	16.69496900	-0.70836800	1.60554400
H	15.52465900	-0.06592400	0.43872700
H	15.93101500	0.86486500	1.87535300
C	-7.96036800	1.16974800	3.57553800
H	-7.88592300	0.59100900	4.49733500
H	-8.57515000	2.05773500	3.70843800
C	-8.52142200	0.31996100	2.44694300
H	-8.23882000	0.76158100	1.49462300
C	-10.02599200	0.18194500	2.50639700
H	-10.34433600	-0.41751400	3.36368700
H	-10.47786900	1.17480300	2.54614100
O	-6.66342700	1.66110700	3.19886000
C	-6.88351900	-1.23256700	1.74285700
C	-6.27970600	-2.59415300	1.93395100
H	-6.81880500	-3.14110900	2.70779200
H	-6.41246000	-3.11042200	0.97969700
C	-4.78326800	-2.42796500	2.24673400
H	-4.65096800	-2.31892400	3.32729100
H	-4.45949900	-1.48024400	1.80769900
C	-3.85202400	-3.50010300	1.69114500
H	-4.09132200	-4.49044700	2.09795300

H	-3.98963000	-3.55504200	0.60614800
C	-2.40335500	-3.12591700	2.00334600
H	-2.19846400	-3.29638900	3.06855900
H	-2.29212800	-2.04382300	1.85399200
C	-1.34193400	-3.81868900	1.15685100
H	-1.55080600	-3.61135100	0.09974700
H	-1.39731200	-4.90773500	1.28040100
C	0.05670500	-3.31953200	1.50703900
H	0.05468200	-2.22248300	1.45081200
H	0.27394400	-3.55376900	2.55792300
C	1.17937000	-3.85793300	0.62577200
H	0.96025500	-3.64415300	-0.42576400
H	1.23634800	-4.94989700	0.72228900
C	2.52543700	-3.23794700	0.98879800
H	2.43635400	-2.14494000	0.94485000
H	2.76016000	-3.47615700	2.03543000
C	3.68873500	-3.67062200	0.10171500
H	3.53564100	-3.28351300	-0.91311100
H	3.70030000	-4.76502800	0.01509100
C	5.03393300	-3.19782700	0.64545700
H	5.00638300	-2.11177300	0.79955200
H	5.18471300	-3.63238100	1.64295800
C	6.22568000	-3.54467600	-0.23951800
H	6.19261200	-4.61124900	-0.49810600
H	6.14696300	-2.98884500	-1.18120900
C	7.56401700	-3.23001000	0.42137400

H	7.59037800	-2.17413400	0.71588200
H	7.64690300	-3.79894200	1.35695500
C	8.76970900	-3.53743200	-0.45889000
H	8.74146200	-4.59397100	-0.75657400
H	8.69572400	-2.95664100	-1.38853400
C	10.10231800	-3.22866500	0.21530400
H	10.14512700	-3.73420000	1.18921100
H	10.15114000	-2.15357700	0.42287800
C	11.31292500	-3.64074200	-0.61665000
H	11.29733300	-4.72603700	-0.78104300
H	11.23583600	-3.19046000	-1.61611400
C	12.64122800	-3.24079200	0.01953300
H	12.73370000	-3.73104600	0.99644200
H	12.62996200	-2.16499400	0.22562300
C	13.85213400	-3.57674800	-0.84328500
H	14.78517100	-3.27796200	-0.35699500
H	13.90852200	-4.65068300	-1.04658800
H	13.79995500	-3.06122100	-1.80797700
O	-7.96546400	-1.00507200	2.50566100
O	-6.40982500	-0.39129800	1.00594300
O	-10.50684900	-0.42256000	1.29053700
O	-11.71346300	-2.10302500	-0.02778500
O	-10.90033800	-2.77781200	2.34667900
O	-9.25463500	-2.40844100	0.34389400
C	-12.30402900	-3.39747400	-0.23744500
H	-12.26762600	-3.96060700	0.69989900

H	-13.35210600	-3.22626200	-0.49532100
C	-11.61918600	-4.15326000	-1.36986200
H	-11.67055600	-3.54916900	-2.27725100
H	-12.19258600	-5.06933900	-1.56194300
N	-10.20194100	-4.43001800	-1.08936300
H	-10.09060800	-5.30041900	-0.57854500
H	-9.66337500	-4.48557100	-1.95475400
P	-10.56183700	-2.00516000	1.13888100
H	-9.49161500	-3.22615600	-0.22084200
O	-10.40333000	-1.45053100	-2.41543900
H	-11.07811900	-1.27963200	-1.72598900

AmB-DSPE2

0 1

C	9.49754100	4.38392500	-0.09686400
H	10.13517400	3.49159100	-0.07163500
C	9.66943300	5.08796100	1.25048400
H	9.05627300	6.00407400	1.23952500
C	9.16495100	4.20582300	2.38593500
H	9.75500100	3.27785400	2.40461600
C	7.62230000	3.13073600	0.93808400
H	8.24201000	2.22412300	1.00848500
C	8.04552900	3.94634000	-0.28321200
H	7.95538100	3.30677700	-1.17305000

C	9.22493900	4.88812200	3.73222800
H	8.60133300	5.78753300	3.72466400
H	10.25358900	5.18159300	3.94645200
H	8.86607600	4.21967000	4.51748200
C	5.58459400	2.27513400	1.93681600
H	5.58027400	3.03089900	2.72862500
C	4.18415400	2.03785500	1.46819300
H	4.04989500	1.25278000	0.73103700
C	3.13109500	2.75973200	1.86801100
H	3.27049800	3.57447500	2.57797700
C	1.79099300	2.52313700	1.37047800
H	1.67397500	1.67868800	0.69991900
C	0.69606700	3.26804300	1.61914100
H	0.75117800	4.14802600	2.25849400
C	-0.57402800	2.89922500	1.03506600
H	-0.54196200	2.00550900	0.41448900
C	-1.76600700	3.50147800	1.21992500
H	-1.84869300	4.39225300	1.84144200
C	-2.97738300	2.94524500	0.66431300
H	-2.85872100	2.03939400	0.06940600
C	-4.22755200	3.41033700	0.86030800
H	-4.37773800	4.31805300	1.44370700
C	-5.40280600	2.73700800	0.36247200
H	-5.23628500	1.79653900	-0.16267100
C	-6.66763800	3.16754300	0.54081500
H	-6.83851600	4.10477300	1.06963100

C	-7.83390300	2.44538300	0.09073600
H	-7.65313300	1.49594500	-0.41199700
C	-9.10357400	2.84655200	0.28842600
H	-9.28822900	3.78980200	0.80187400
C	6.29874900	1.04232400	2.49279900
H	7.28128200	1.35014400	2.85167700
H	5.73953900	0.68421600	3.36223500
C	-10.26021800	2.05932900	-0.09046200
H	-10.06006200	1.12791500	-0.61374900
C	-11.52460500	2.36079900	0.22442200
H	-11.73480400	3.28830800	0.76048900
C	-12.70896000	1.48330500	-0.06350800
H	-12.36187400	0.59222200	-0.59798700
C	-13.36405900	1.02689400	1.25387300
H	-13.74522900	1.93398300	1.75112100
C	-12.40557000	0.33736900	2.24134600
H	-11.50955700	0.96336300	2.27724000
C	-11.93348200	-1.03769100	1.74122200
H	-11.77510500	-1.00443800	0.66496600
C	-9.54743200	-1.19863500	1.60954500
C	-8.27587900	-1.47725800	2.36823400
H	-8.43243000	-1.28019200	3.43045400
H	-8.06203700	-2.54660000	2.25504300
C	-7.08698900	-0.67683800	1.82561700
H	-7.28911600	0.39730300	1.94884000
C	-5.80537400	-1.04775000	2.55485600

H	-5.93548900	-0.84802200	3.62386800
H	-5.63982400	-2.12535400	2.43632500
C	-4.55478200	-0.31593800	2.07002800
H	-4.71523400	0.76922000	2.13547600
C	-3.35561900	-0.69668000	2.93187900
H	-3.34154500	-1.78799200	3.04771400
H	-3.53263200	-0.28352900	3.93001700
C	-1.99393500	-0.22022000	2.40259900
H	-2.11434300	0.63451400	1.73475000
H	-1.38308100	0.13129200	3.23957300
C	-1.19411500	-1.30750800	1.68862900
H	-1.05606800	-2.14407600	2.39156700
C	0.19482800	-0.80764100	1.25390500
H	0.57961800	-0.11418800	2.01192500
C	1.19478000	-1.94222300	1.07625200
H	0.85092500	-2.61760000	0.28585800
H	1.23054800	-2.51627400	2.01009800
C	2.60080600	-1.44827400	0.74903700
H	2.82451500	-0.57222500	1.36942100
C	3.65854400	-2.50694400	1.05098300
H	3.48884900	-3.38783200	0.42362700
H	3.56751400	-2.81150500	2.09676800
C	5.08315400	-2.01431900	0.80883000
C	6.15315600	-3.00876600	1.24026500
H	6.09342600	-3.91092000	0.62807700
H	6.00217400	-3.28478700	2.28815300

C	7.51863500	-2.37650300	1.06639300
H	7.64314000	-2.13320200	0.00911200
C	7.60904500	-1.07698400	1.87553600
H	7.53719000	-1.31900800	2.94180000
C	6.42923400	-0.12977000	1.52420200
H	6.56273300	0.22881200	0.49475100
C	8.93523600	-0.37318400	1.69309800
C	-12.80799400	-2.22317700	2.09258400
H	-12.38459400	-3.13771600	1.67283500
H	-13.80001900	-2.07156600	1.66721900
H	-12.89261500	-2.35184800	3.17326100
C	-12.99274100	0.28458500	3.65104000
H	-13.94891400	-0.24800000	3.67982200
H	-13.16149500	1.29465300	4.03381900
H	-12.31407000	-0.22607600	4.33859300
C	-13.73577700	2.20031900	-0.94558500
H	-14.09415300	3.11341800	-0.45739800
H	-14.59504800	1.55611000	-1.13661200
H	-13.28572300	2.48546200	-1.89893000
O	7.27257200	5.11532300	-0.41884500
H	6.34579600	4.87732800	-0.60567400
O	11.02772200	5.38367000	1.46488400
H	11.34146800	5.69772800	0.60094600
O	7.79393500	3.87869200	2.12804800
O	6.27489000	2.82563600	0.79492400
O	5.29827500	-1.72917700	-0.54221100

H	4.43713600	-1.41495600	-0.89139600
O	5.20620000	-0.84075800	1.61606600
O	9.35028600	0.54037600	2.36857600
O	2.73577000	-1.09831900	-0.63288600
H	2.43686500	-0.18702700	-0.79385800
O	0.06036900	-0.06028900	0.05498200
H	-0.65030600	-0.52429000	-0.42167500
O	-1.82709600	-1.78935500	0.51438600
H	-2.75613400	-1.48083100	0.49536300
O	-4.25639800	-0.64279300	0.71062100
H	-5.09958700	-0.77568000	0.24071400
O	-6.88053900	-0.96036600	0.44609300
H	-7.74567100	-0.90002200	0.00301200
O	-9.56039500	-0.94211100	0.41628200
O	-10.63278300	-1.29021200	2.36299900
O	-14.45854800	0.18114400	0.90692400
H	-15.04498600	0.09916100	1.66686800
O	8.49965500	-3.31209000	1.48350300
H	9.34072100	-3.02807100	1.10554200
N	10.00672700	5.27630400	-1.13092000
H	9.28078300	5.95746500	-1.34314000
H	10.19191900	4.76740900	-1.98920300
C	-18.53962500	-2.26989800	-0.30149600
H	-18.34721200	-1.36105600	0.28285200
H	-18.82195400	-1.92839800	-1.30494300
C	-17.25385200	-3.08711500	-0.39381300

H	-17.44288800	-3.99525100	-0.98159800
H	-16.97253400	-3.43277800	0.61058900
C	-16.08967600	-2.31516300	-1.00654200
H	-15.89899500	-1.40663900	-0.42187100
H	-16.36872600	-1.97415900	-2.01236100
C	-14.79929700	-3.12588900	-1.08991400
H	-14.54278900	-3.49999700	-0.08985100
H	-14.95897300	-4.01625900	-1.71220400
C	-13.62853400	-2.31549500	-1.63686900
H	-13.52733700	-1.40468300	-1.03456900
H	-13.86181500	-1.98016700	-2.65598600
C	-12.30273200	-3.07054500	-1.64239800
H	-12.06099800	-3.38071600	-0.61691500
H	-12.40489100	-3.99840100	-2.21999100
C	-11.14809300	-2.24229100	-2.19668800
H	-11.10933800	-1.28235100	-1.66709700
H	-11.34232500	-2.00334000	-3.25035800
C	-9.78447100	-2.91288800	-2.06265600
H	-9.61069000	-3.15019000	-1.00589400
H	-9.77909700	-3.86886700	-2.60219800
C	-8.65075400	-2.02449100	-2.56159500
H	-8.73908000	-1.04731200	-2.06840200
H	-8.78161500	-1.82640800	-3.63328700
C	-7.24874600	-2.57926200	-2.31652400
H	-7.14429100	-2.86094100	-1.26173300
H	-7.10180900	-3.49646100	-2.90074500

C	-6.17409000	-1.55582000	-2.66516400
H	-6.33609700	-0.65955400	-2.05171000
H	-6.30771300	-1.23018000	-3.70513000
C	-4.73061100	-2.01832400	-2.47802000
H	-4.50277700	-2.81981000	-3.19196300
H	-4.59786100	-2.44979100	-1.47735900
C	-3.76902000	-0.84913000	-2.67070600
H	-3.88988500	-0.15522300	-1.82856600
H	-4.07453400	-0.29009800	-3.56534600
C	-2.28791900	-1.19408200	-2.80748900
H	-2.13648700	-1.83559100	-3.68503000
H	-1.94738200	-1.76548900	-1.93646000
C	-1.48853200	0.09711900	-2.95835900
H	-1.61372200	0.70867100	-2.05883200
H	-1.93367600	0.68604600	-3.76867200
C	0.00459400	-0.06204500	-3.23989600
H	0.19342600	-0.73470100	-4.08037900
H	0.53425000	-0.47737300	-2.37469700
C	0.67595100	1.24735300	-3.57057000
O	1.65299000	1.38472500	-4.26507300
C	-19.69601800	-3.04559600	0.32090700
H	-20.60514100	-2.44062200	0.37646900
H	-19.92682100	-3.94155100	-0.26399500
H	-19.44923000	-3.37168800	1.33648800
C	0.52365100	3.59536000	-3.18635400
H	1.07089700	3.65645900	-4.12943400

H	-0.36313700	4.23270900	-3.22957400
C	1.42251400	4.02850900	-2.04096200
H	1.11358800	3.53644700	-1.11774300
C	1.40269900	5.53706000	-1.86519000
H	1.76619600	6.03052100	-2.77175400
H	0.38426900	5.86612100	-1.64871400
O	0.02633600	2.27719800	-2.98405400
C	3.28100800	2.51629600	-1.94936200
C	4.75357700	2.43643200	-2.20719200
H	5.01475400	3.17086500	-2.97252500
H	5.22084700	2.76704200	-1.27337200
C	5.22368300	1.03260400	-2.55878200
H	4.77840300	0.71910100	-3.50970500
H	4.85646100	0.34242700	-1.79841700
C	6.74453600	0.93727400	-2.62331000
H	7.13633100	1.67659800	-3.33582600
H	7.15181700	1.21331900	-1.64073300
C	7.24065600	-0.45701200	-2.99159100
H	6.86893400	-0.72481800	-3.98948100
H	6.79604700	-1.16960100	-2.29174800
C	8.76367700	-0.57687200	-2.97191300
H	9.13050900	-0.28369600	-1.97817600
H	9.19047600	0.14157000	-3.68326400
C	9.28087900	-1.98208000	-3.29384800
H	8.89804500	-2.29735000	-4.27327000
H	10.37394800	-1.95436000	-3.38840900

C	8.90040400	-3.01817900	-2.23932300
H	7.80920200	-3.09268100	-2.16817900
H	9.24337200	-2.65888100	-1.26159700
C	9.47320300	-4.41126000	-2.47543500
H	9.22257500	-4.75018200	-3.48915900
H	10.57004600	-4.36913900	-2.43253000
C	8.95590300	-5.42492300	-1.45646900
H	7.87142900	-5.53805500	-1.58844700
H	9.08416100	-5.02626700	-0.44185100
C	9.62104900	-6.79320500	-1.56021100
H	9.49501700	-7.18573700	-2.57815300
H	10.70364500	-6.68341700	-1.41095700
C	9.07403400	-7.80390500	-0.55649200
H	9.19010200	-7.40394100	0.45932100
H	7.99343800	-7.92056000	-0.71256100
C	9.74682200	-9.17052200	-0.64297900
H	9.63220600	-9.56864900	-1.65996500
H	10.82721100	-9.05418800	-0.48422500
C	9.19596200	-10.18060500	0.35925200
H	9.30862700	-9.78128400	1.37588200
H	8.11605300	-10.29851100	0.19918700
C	9.87044900	-11.54674200	0.27597000
H	10.95032000	-11.42872400	0.43669500
H	9.75824700	-11.94564800	-0.74095100
C	9.31885700	-12.55678000	1.27772500
H	9.43030500	-12.15814100	2.29495400

H	8.23903000	-12.67593200	1.11694000
C	9.99372900	-13.92333300	1.19586400
H	11.07224300	-13.80282900	1.35691500
H	9.88163300	-14.32029700	0.17926000
C	9.43379000	-14.92404100	2.20167000
H	9.93142200	-15.89468500	2.12531800
H	9.56167700	-14.56309600	3.22721200
H	8.36287600	-15.08444100	2.04111500
O	2.78849500	3.70017200	-2.32553600
O	2.60138400	1.64886100	-1.43529700
O	2.19618500	5.92941100	-0.74814600
O	3.92742000	7.36177600	0.16188100
O	4.19349300	6.51319000	-2.31150700
O	4.52351200	4.96741400	-0.27537200
C	5.24499300	7.67749100	0.63341900
H	6.01617400	7.26324600	-0.02430000
H	5.33609700	8.76606000	0.62358000
C	5.41117000	7.15167800	2.04809400
H	4.57155200	7.52058200	2.64260700
H	6.33279100	7.56699700	2.47659300
N	5.40011400	5.68182500	2.07508500
H	6.33688900	5.29650300	1.93845000
H	5.07422900	5.34197400	2.97304800
P	3.76698200	6.19707900	-0.93961900
H	4.71419200	5.11169000	0.72786200
O	9.65875500	-0.84290100	0.64990500

H	10.47262400	-0.31446400	0.61741200

AmB-DSPE3

0 1

C	-12.52622000	5.11758800	2.19728500
H	-13.32048800	4.37979400	2.02332700
C	-12.71920000	6.22529100	1.16088600
H	-11.95374000	6.99748200	1.33566300
C	-12.51030500	5.68233300	-0.24743000
H	-13.24816500	4.88657400	-0.43680300
C	-11.01179800	4.03984000	0.53891200
H	-11.75264000	3.25805400	0.29648400
C	-11.17657800	4.43064700	2.00641400
H	-11.13278800	3.50745400	2.60768400
C	-12.62264700	6.74669400	-1.31341700
H	-11.85309500	7.50826000	-1.16103600
H	-13.60368200	7.22055000	-1.25461300
H	-12.49010800	6.30719600	-2.30407100
C	-9.20119100	3.36192500	-0.93038100
H	-9.02262900	4.34217600	-1.38394100
C	-7.91076300	2.63131100	-0.73192700
H	-7.97405900	1.67921100	-0.21420900
C	-6.72109300	3.06903400	-1.15223500
H	-6.64810400	4.02332800	-1.67387100

C	-5.48996100	2.33235000	-0.93804600
H	-5.57821100	1.37861300	-0.42266600
C	-4.27789200	2.76420400	-1.33001700
H	-4.19734200	3.71726700	-1.85289200
C	-3.04990100	2.04273800	-1.09339400
H	-3.12717800	1.09092600	-0.56880100
C	-1.84291100	2.48494800	-1.49926000
H	-1.78230200	3.43098900	-2.03689000
C	-0.60196200	1.79142300	-1.26729900
H	-0.64852100	0.85120700	-0.71879300
C	0.59449400	2.23618800	-1.70427500
H	0.63729900	3.15958900	-2.28092200
C	1.84219900	1.57103400	-1.43305700
H	1.80200200	0.68917700	-0.79608400
C	3.04100700	1.96962700	-1.90386200
H	3.09920700	2.83065800	-2.56851200
C	4.27456100	1.31705200	-1.54223600
H	4.20538500	0.53357300	-0.79069300
C	5.48910800	1.59832200	-2.04688600
H	5.59637600	2.35125000	-2.82639200
C	-10.20433100	2.60320300	-1.80650200
H	-11.10035400	3.21277200	-1.92420900
H	-9.76942800	2.47530300	-2.80134700
C	6.67992800	0.90309700	-1.59968600
H	6.55378700	0.30210300	-0.70167200
C	7.86927100	0.91337900	-2.20932900

H	7.99833400	1.50069200	-3.11947100
C	9.06318600	0.13059000	-1.74228800
H	8.88532900	-0.20137800	-0.71377000
C	9.29274500	-1.13446000	-2.59209000
H	9.63389200	-0.80203500	-3.58644100
C	8.05125000	-2.01295300	-2.83792700
H	7.32219800	-1.36245900	-3.33143100
C	7.35587800	-2.49964400	-1.55971200
H	7.19158300	-1.66448500	-0.88399200
C	4.95362500	-2.56050900	-1.35307900
C	3.68184800	-3.07605400	-1.97662000
H	3.88004900	-3.37615100	-3.00712200
H	3.34716700	-3.96093300	-1.42285900
C	2.56663100	-2.03062600	-1.91460700
H	2.91525100	-1.09748600	-2.38092500
C	1.31695500	-2.50851500	-2.63857600
H	1.55524900	-2.60398700	-3.70335700
H	1.02989700	-3.49613500	-2.26233500
C	0.13089000	-1.56493100	-2.46295200
H	0.46760900	-0.52438400	-2.55986300
C	-0.96583000	-1.80822000	-3.49347200
H	-1.16732900	-2.88306700	-3.55367000
H	-0.56976200	-1.50147000	-4.46598600
C	-2.26206400	-1.04673900	-3.17027100
H	-2.05698000	-0.25516800	-2.44560800
H	-2.65535700	-0.54736500	-4.06094300

C	-3.36524200	-1.95045300	-2.62005300
H	-3.78047300	-2.54639700	-3.44081300
C	-4.51335500	-1.14034400	-1.96388400
H	-4.50107200	-0.11088800	-2.33811300
C	-5.88058800	-1.76280200	-2.21620300
H	-5.90823500	-2.77299300	-1.79739000
H	-6.03138000	-1.85047700	-3.29868400
C	-7.04144100	-0.95109900	-1.64920500
H	-7.08895000	0.02489800	-2.14825600
C	-8.36970500	-1.68042600	-1.84994500
H	-8.28911300	-2.68443000	-1.42547000
H	-8.56036200	-1.77320600	-2.92315800
C	-9.55304800	-0.96107900	-1.21073200
C	-10.89551800	-1.62312400	-1.50336900
H	-10.94573200	-2.59948500	-1.01628700
H	-10.99202100	-1.77649600	-2.58200500
C	-12.04522100	-0.75158100	-1.02738500
H	-11.98367900	-0.65627900	0.06394400
C	-11.91936800	0.65217800	-1.63694500
H	-11.96248100	0.58140900	-2.72819300
C	-10.53599900	1.22544800	-1.25399800
H	-10.46787700	1.24454700	-0.15920200
C	-13.04000700	1.57688200	-1.23386400
C	8.02056300	-3.63543200	-0.80938700
H	7.45600100	-3.86272100	0.09708600
H	9.03495100	-3.34224100	-0.53550800

H	8.06638900	-4.53814000	-1.42210900
C	8.37525200	-3.16228700	-3.79259400
H	9.15192300	-3.82025700	-3.39099200
H	8.72283200	-2.77819600	-4.75594800
H	7.49022800	-3.77562800	-3.97304100
C	10.32180900	1.00215500	-1.75292100
H	10.51546900	1.38356800	-2.76175100
H	11.19193600	0.43107200	-1.43083800
H	10.20100500	1.86077300	-1.08942400
O	-10.16723300	5.33193700	2.39863000
H	-9.36372300	5.04929800	1.93802200
O	-14.02170400	6.75025100	1.26786600
H	-14.17007700	6.82988000	2.22377600
O	-11.19330400	5.13290400	-0.32125000
O	-9.70982000	3.56727900	0.40656800
O	-9.40837400	-0.88729200	0.19403600
H	-8.45052700	-0.70254400	0.33157800
O	-9.54447600	0.34636100	-1.76229400
O	-13.37192400	2.57622100	-1.82429300
O	-6.87455500	-0.69563500	-0.24433100
H	-5.96179800	-0.92984400	0.01114100
O	-4.25836600	-1.04281800	-0.55671000
H	-3.89023400	-1.90645900	-0.28664200
O	-2.86568700	-2.88689500	-1.67506600
H	-2.07309200	-2.49682300	-1.25531500
O	-0.43322900	-1.73154500	-1.15301400

H	0.31486300	-1.74940100	-0.53471100
O	2.21895300	-1.75378200	-0.55627000
H	3.05214700	-1.59253100	-0.08093600
O	4.96405500	-1.84139000	-0.36424000
O	6.03884900	-2.96210100	-1.99771300
O	10.34128300	-1.85426000	-1.94817100
H	10.71203600	-2.49105100	-2.56822000
O	-13.25510400	-1.38114000	-1.40566000
H	-13.96062000	-0.96592500	-0.89487400
N	-12.73844700	5.70239300	3.51713600
H	-11.89530100	6.20913800	3.77770500
H	-12.87509500	4.97942500	4.21634200
C	18.84982900	2.95779000	-0.35830500
H	18.74230600	3.49241400	0.59397200
H	19.52487600	2.11617000	-0.15970900
C	17.48525900	2.42418600	-0.78374500
H	17.58409000	1.87629900	-1.73014600
H	16.82142700	3.27235700	-0.99393900
C	16.82918500	1.52594000	0.26025500
H	16.80744500	2.05188900	1.22406400
H	17.44531800	0.63177000	0.42052700
C	15.40920000	1.11323300	-0.11378100
H	14.81815100	2.01581400	-0.32089600
H	15.42599100	0.54426900	-1.05253500
C	14.70318900	0.29921500	0.96624600
H	14.73393200	0.85601600	1.91246500

H	15.25090500	-0.63510100	1.14554500
C	13.25121100	-0.00773200	0.61900800
H	12.72533000	0.93929500	0.44023700
H	13.20469500	-0.55757100	-0.33032300
C	12.49933000	-0.79241300	1.69098500
H	12.66062600	-0.32316900	2.67053200
H	12.91217600	-1.80684500	1.76661800
C	11.00441600	-0.85482700	1.40063300
H	10.60743600	0.16861300	1.40766700
H	10.84698800	-1.23217700	0.38387000
C	10.18810700	-1.70068300	2.37327500
H	10.48725000	-1.48942800	3.40793800
H	10.40268300	-2.76424800	2.20458000
C	8.69349000	-1.44031400	2.21067800
H	8.45615700	-0.43987500	2.59458100
H	8.45384700	-1.40888100	1.13951600
C	7.78150200	-2.47282900	2.86060200
H	8.05422600	-2.61256500	3.91367000
H	7.95589200	-3.44288100	2.37700700
C	6.30272600	-2.09308700	2.73700500
H	6.15825700	-1.48199100	1.83863100
H	6.00502100	-1.46147300	3.58320400
C	5.37008900	-3.29300400	2.62187400
H	5.55581800	-4.00673600	3.43426500
H	5.61307400	-3.82268500	1.69138400
C	3.89472900	-2.90074500	2.59681400

H	3.79026500	-1.94441300	2.07033300
H	3.53715600	-2.72077700	3.61851500
C	3.01946900	-3.93800500	1.90268800
H	3.18011300	-4.92885500	2.34291000
H	3.32302000	-4.01392500	0.85582400
C	1.54330900	-3.57890900	1.95839900
H	1.41300900	-2.51540500	1.73632300
H	1.11700600	-3.73606900	2.95250100
C	0.69676800	-4.30273800	0.94663500
O	1.08068300	-4.79502700	-0.09075900
C	19.47112400	3.88648200	-1.39630800
H	20.44832900	4.25636800	-1.07427800
H	19.60717500	3.37253900	-2.35321000
H	18.82824200	4.75416000	-1.57639600
C	-1.54637400	-4.87032000	0.43413200
H	-1.48149100	-4.39180200	-0.54306700
H	-1.34087800	-5.93800100	0.31121700
C	-2.89608400	-4.65739800	1.09004900
H	-2.89438800	-5.09344600	2.09120600
C	-4.03525800	-5.25811600	0.29824200
H	-4.23193900	-4.66826000	-0.59987300
H	-3.78921300	-6.28142500	0.00145300
O	-0.58911700	-4.29696300	1.32850900
C	-3.20560300	-2.73273200	2.48669000
C	-2.90664900	-1.25689800	2.47525500
H	-3.47400000	-0.75025500	1.69113500

H	-3.19024000	-0.85130100	3.44788200
C	-1.39765100	-1.12095200	2.22817700
H	-1.18470100	-1.52753200	1.23601700
H	-0.87710100	-1.77174900	2.94105300
C	-0.80309900	0.28076100	2.32972700
H	-1.23857300	0.93886200	1.56831800
H	-1.03844300	0.72781000	3.30321100
C	0.71031700	0.17759300	2.15124900
H	0.91188600	-0.34778100	1.20851200
H	1.10305800	-0.47617800	2.94194900
C	1.52498500	1.46601800	2.14617900
H	1.30686100	2.06179300	3.04102600
H	1.24405400	2.08130700	1.28361000
C	3.01588800	1.13064000	2.09309000
H	3.30267300	0.63045800	3.02790100
H	3.17691700	0.38336700	1.30390600
C	3.95526500	2.30166300	1.83489500
H	3.77538000	3.09805800	2.56830600
H	3.73061900	2.73015700	0.85113500
C	5.42190200	1.87566400	1.88847100
H	5.67852800	1.59038900	2.91751400
H	5.55318100	0.96534500	1.28914300
C	6.39419200	2.94016900	1.39254400
H	6.20022800	3.88666900	1.91432600
H	6.20080600	3.12987500	0.32963700
C	7.85863100	2.55486900	1.58438300

H	8.06341200	2.44184400	2.65765700
H	8.03971100	1.56896100	1.13649200
C	8.83329400	3.56438200	0.98293800
H	8.74478900	3.53618400	-0.11121100
H	8.54069900	4.57922600	1.28300800
C	10.28425200	3.32658400	1.39368800
H	10.37200800	3.44805700	2.48122800
H	10.56020200	2.28540000	1.18644400
C	11.27631700	4.25369600	0.69716900
H	11.26498800	4.05079600	-0.38225600
H	10.94472800	5.29419900	0.81154900
C	12.70307300	4.12270400	1.22409200
H	13.02471500	3.07505200	1.15848300
H	12.71644800	4.37490500	2.29258000
C	13.70487600	5.00412400	0.48356100
H	13.72510000	4.71747300	-0.57672900
H	13.36096500	6.04678200	0.50803800
C	15.11926100	4.92583300	1.05187800
H	15.45822800	3.88434800	1.02707100
H	15.09995100	5.21277700	2.11044600
C	16.11369000	5.80594900	0.30132800
H	17.12660000	5.70058100	0.70043100
H	16.14702500	5.54402300	-0.76120800
H	15.83454500	6.86214100	0.36903800
O	-3.10617900	-3.24441200	1.22422400
O	-3.38101300	-3.40775700	3.46503200

O	-5.18162000	-5.33263500	1.14728500
O	-7.50966900	-5.22833500	1.79676000
O	-6.86281700	-4.31327000	-0.54316000
O	-6.19382600	-3.06087100	1.62857500
C	-8.90180400	-4.92098900	1.70152300
H	-9.14191800	-4.57897000	0.68805800
H	-9.43944100	-5.85407300	1.88406500
C	-9.29593600	-3.88705200	2.74181600
H	-8.93667200	-4.22988800	3.71555600
H	-10.39285200	-3.83658100	2.78561400
N	-8.68688400	-2.58433200	2.44605400
H	-9.14797200	-2.14788900	1.64325100
H	-8.80144900	-1.95264800	3.23289800
P	-6.46225300	-4.42725000	0.87812300
H	-7.08412200	-2.73344000	2.00841300
O	-13.65613000	1.20635000	-0.08354000
H	-14.33859500	1.87731500	0.08208500

AmB-DSPE4

0 1

C	10.15246400	3.66444200	-1.90940900
H	10.96444000	3.05029800	-1.50124500
C	10.03114400	4.89128500	-1.00738800
H	9.23855400	5.54121300	-1.41016100

C	9.61384400	4.47234100	0.39680100
H	10.38013500	3.79426100	0.80435200
C	8.46999600	2.58213800	-0.42849700
H	9.21749900	1.93357500	0.05035700
C	8.86507300	2.84650700	-1.87983700
H	9.02580800	1.87904000	-2.37536000
C	9.41410000	5.64614100	1.32679300
H	8.61819000	6.29119900	0.94472200
H	10.33693000	6.22509400	1.38826300
H	9.13435100	5.29969100	2.32396500
C	6.45477700	1.96489500	0.76290900
H	6.25679500	3.00600600	1.03336500
C	5.16216800	1.28714800	0.42359100
H	5.19258500	0.22005900	0.22090800
C	4.00300600	1.94992900	0.34951200
H	3.99077700	3.01903500	0.56261000
C	2.73102500	1.34508700	0.00315300
H	2.72913100	0.27947000	-0.21190400
C	1.56872700	2.02369400	-0.04598400
H	1.56996500	3.08917300	0.18427500
C	0.29131100	1.41643000	-0.33835900
H	0.29957000	0.35599800	-0.58212100
C	-0.89037300	2.05800700	-0.23116500
H	-0.90197800	3.10967300	0.05474800
C	-2.16492500	1.39883400	-0.38635200
H	-2.14509300	0.36566900	-0.72748300

C	-3.35349000	1.96104500	-0.08007200
H	-3.38428300	2.99375000	0.26633900
C	-4.61143700	1.25982400	-0.16258900
H	-4.56911900	0.22253400	-0.49498700
C	-5.81148900	1.80030900	0.13514500
H	-5.85500800	2.83831300	0.46346800
C	-7.06699000	1.10072400	0.00379500
H	-7.02254800	0.05765900	-0.30747900
C	-8.27941000	1.64683700	0.22149800
H	-8.35104600	2.68166500	0.55461600
C	7.24598600	1.33191300	1.91186200
H	8.15813300	1.90924500	2.05492700
H	6.65789300	1.42473600	2.82972600
C	-9.51718500	0.91877200	0.02006300
H	-9.40948800	-0.08371800	-0.38851100
C	-10.74192300	1.36719900	0.32152900
H	-10.85499200	2.36565800	0.74726800
C	-12.00267000	0.56861800	0.14348000
H	-11.76423800	-0.34075800	-0.41937800
C	-12.59122200	0.11372500	1.49413500
H	-13.02044600	1.00495300	1.98238600
C	-11.57177600	-0.48188000	2.47724500
H	-10.80618100	0.28775400	2.61330600
C	-10.82280100	-1.69834900	1.91348700
H	-10.59634700	-1.53863000	0.86199600
C	-8.43111300	-1.81179000	1.89957000

C	-7.22932600	-1.46616100	2.73700800
H	-7.22757100	-0.36964800	2.77328500
H	-7.37144800	-1.82597400	3.75812200
C	-5.90448000	-1.97572100	2.18300700
H	-5.90546300	-1.84323800	1.09254000
C	-4.73686800	-1.19764700	2.76797900
H	-4.90222500	-0.13320600	2.57383500
H	-4.71218400	-1.34504800	3.85425200
C	-3.37993200	-1.58177400	2.18555800
H	-3.42974500	-1.51214000	1.08723200
C	-2.29901900	-0.63724400	2.68747700
H	-2.19095000	-0.77968800	3.77037700
H	-2.67170200	0.37991500	2.53456900
C	-0.94540700	-0.73588800	1.98410200
H	-1.09628200	-0.90256200	0.91644300
H	-0.44910800	0.23380800	2.07660500
C	0.03563200	-1.79030100	2.48429600
H	0.15153500	-1.68979900	3.57672700
C	1.40544700	-1.55015400	1.85572700
H	1.67400200	-0.50195800	2.02687000
C	2.52305500	-2.42978400	2.40844500
H	2.34277700	-3.47350200	2.13022800
H	2.51347000	-2.37258500	3.50237900
C	3.88475600	-1.96668400	1.89907200
H	4.00532900	-0.90187000	2.13839300
C	5.04971500	-2.72060300	2.52097500

H	4.96246600	-3.78911100	2.30182600
H	5.01165000	-2.58813400	3.60584300
C	6.41241300	-2.24401000	2.01382000
C	7.57227400	-2.83763500	2.79726700
H	7.62438100	-3.91649500	2.63808200
H	7.41637000	-2.65331000	3.86415000
C	8.88799300	-2.20528100	2.38498000
H	9.09372900	-2.47515000	1.34123000
C	8.80620200	-0.66971300	2.47926400
H	8.68651100	-0.39945400	3.53559500
C	7.56086300	-0.14636900	1.71764800
H	7.69290200	-0.36775700	0.65128100
C	10.08334600	0.00596000	2.02039600
C	-11.49774600	-3.04589100	2.06727500
H	-10.89502900	-3.81653300	1.58183500
H	-12.48169800	-3.00804700	1.59611300
H	-11.61428100	-3.31419200	3.11936600
C	-12.19942100	-0.75314400	3.84313400
H	-13.01995700	-1.47498700	3.78270900
H	-12.59278100	0.16995000	4.27889500
H	-11.45702000	-1.16134700	4.53273200
C	-13.05592000	1.35184800	-0.64557300
H	-13.31417800	2.28266200	-0.12827400
H	-13.96527700	0.75853300	-0.75290800
H	-12.68337500	1.61580400	-1.63821100
O	7.85863100	3.58218100	-2.54148300

H	7.01839500	3.23738200	-2.20558800
O	11.26983600	5.55988300	-0.94645400
H	11.59573300	5.53961700	-1.86057400
O	8.36918600	3.77844800	0.30273900
O	7.21504700	1.96359000	-0.46603500
O	6.60191800	-2.56726500	0.66348600
H	5.76237300	-2.36245500	0.21379100
O	6.41332600	-0.83439800	2.19869800
O	10.29669400	1.19646000	2.01108300
O	3.96990700	-2.12296400	0.47665300
H	3.07592800	-1.95215800	0.12253300
O	1.32780000	-1.68530200	0.42917100
H	0.83404100	-2.50049000	0.22664100
O	-0.35036300	-3.11400300	2.15223300
H	-1.30142000	-3.20626100	2.37429500
O	-3.02115900	-2.91655300	2.54910800
H	-3.82560700	-3.46268500	2.45476100
O	-5.71964300	-3.34690500	2.50286700
H	-6.20020800	-3.89793600	1.84919900
O	-8.40413100	-2.04829700	0.70920100
O	-9.55353600	-1.72711800	2.62002300
O	-13.63138300	-0.80620200	1.17519200
H	-14.18414400	-0.92636700	1.95497800
O	9.87944000	-2.72704800	3.24877700
H	10.73393200	-2.50466800	2.86047900
N	10.55931400	4.12095200	-3.23516500

H	9.73609900	4.48200100	-3.71220600
H	10.90497500	3.34512200	-3.79129900
C	19.06243600	3.13498500	1.65902000
H	19.13699300	2.34590600	2.41765300
H	19.63696100	2.77968900	0.79449300
C	17.60067400	3.30516600	1.25423500
H	17.52420400	4.09467400	0.49493700
H	17.02416600	3.66104700	2.11835400
C	16.96773900	2.02495400	0.71726700
H	17.04420100	1.23571300	1.47708000
H	17.54425000	1.66934200	-0.14705800
C	15.50665900	2.19761200	0.31335700
H	14.92966200	2.55432400	1.17650600
H	15.42917800	2.98627100	-0.44617000
C	14.87209500	0.91811500	-0.22350800
H	14.94927600	0.12746200	0.53558400
H	15.44500700	0.56041300	-1.08885900
C	13.41136300	1.09608300	-0.62398300
H	12.83999900	1.47575000	0.23661400
H	13.33554500	1.88155500	-1.38726800
C	12.75277200	-0.17842900	-1.14982800
H	12.81541400	-0.97129400	-0.39027800
H	13.31741700	-0.56192800	-2.00810800
C	11.29506700	0.04391600	-1.54203400
H	10.80140300	0.62009100	-0.74984200
H	11.25871600	0.68186600	-2.43492200

C	10.48819600	-1.22752800	-1.78465800
H	10.49746200	-1.83129300	-0.86853500
H	10.96578000	-1.83661200	-2.56257000
C	9.04596200	-0.91678700	-2.17261500
H	8.62310000	-0.21185600	-1.44549000
H	9.03448300	-0.38905800	-3.13606800
C	8.12555900	-2.12901200	-2.24719200
H	8.14942800	-2.66185100	-1.29014700
H	8.48234100	-2.82901500	-3.01402100
C	6.68525900	-1.71898000	-2.53800700
H	6.63262800	-1.20385300	-3.50645100
H	6.37805300	-0.97713600	-1.78901500
C	5.69635300	-2.87932600	-2.52117500
H	5.78836800	-3.41729200	-1.57063800
H	5.95626100	-3.60542400	-3.30201900
C	4.25231700	-2.41986000	-2.70428300
H	4.10200200	-2.06208000	-3.73088100
H	4.06611000	-1.56104000	-2.04856500
C	3.24263000	-3.51349700	-2.37994700
H	3.37018100	-3.81045800	-1.33360000
H	3.43141800	-4.40121900	-2.99387800
C	1.79511100	-3.05310900	-2.60165200
H	1.62948300	-2.83601800	-3.65944000
H	1.58942200	-2.15295400	-2.01713900
C	0.84598600	-4.14645800	-2.20659800
O	0.45629400	-5.03285700	-2.92155100

C	19.68372300	4.42111400	2.19416100
H	20.72974300	4.27628900	2.47769200
H	19.64839400	5.21629600	1.44273000
H	19.14492700	4.77946500	3.07704400
C	-0.39509800	-5.01603000	-0.35620500
H	-0.33133900	-5.93029600	-0.94926700
H	-0.08085900	-5.18504400	0.67364800
C	-1.80656500	-4.44752600	-0.36451800
H	-1.86901200	-3.59403800	0.30826400
C	-2.80658400	-5.50314000	0.04002200
H	-2.97199400	-6.20919700	-0.77680900
H	-2.44336400	-6.04213300	0.91800800
O	0.51023200	-4.04731300	-0.88907300
C	-2.05124700	-2.72315000	-1.98831700
C	-2.10267800	-2.50038500	-3.47822400
H	-1.13527800	-2.84084200	-3.86536500
H	-2.84427700	-3.18590600	-3.89976600
C	-2.39170200	-1.04966600	-3.84452400
H	-2.15890700	-0.88535900	-4.90155100
H	-1.73237000	-0.39642800	-3.26433000
C	-3.84721300	-0.69242400	-3.56874300
H	-4.48872700	-1.29620300	-4.22601100
H	-4.08371900	-0.99738500	-2.54314600
C	-4.20350100	0.78041000	-3.73468100
H	-3.86368000	1.14540400	-4.71216100
H	-3.66842300	1.36647500	-2.97848300

C	-5.70668500	1.00102700	-3.58966800
H	-6.05582100	0.46101200	-2.70003500
H	-6.22734300	0.54579400	-4.44343100
C	-6.13883200	2.45392800	-3.43421800
H	-5.66250800	2.85757600	-2.53319600
H	-5.77295900	3.05528200	-4.27575700
C	-7.65710100	2.58040100	-3.30608000
H	-8.02635100	1.74687700	-2.69653000
H	-8.12636300	2.46869800	-4.29215200
C	-8.12402200	3.87491000	-2.65018300
H	-7.64030300	3.95711800	-1.66798000
H	-7.78688700	4.74305600	-3.23117500
C	-9.63777500	3.92149600	-2.46324400
H	-9.96372200	2.98184500	-2.00043600
H	-10.12992900	3.96648400	-3.44367700
C	-10.11694300	5.08135700	-1.59644200
H	-9.60819200	5.03388500	-0.62345500
H	-9.81750400	6.03666400	-2.04695200
C	-11.62564800	5.06671800	-1.37150400
H	-12.13991500	5.14942900	-2.33802600
H	-11.91362700	4.09023500	-0.96179600
C	-12.11999800	6.16650300	-0.43766000
H	-11.59262900	6.09045400	0.52283200
H	-11.85278800	7.14753900	-0.85176800
C	-13.62480900	6.11197200	-0.19082400
H	-14.15343700	6.18896200	-1.15001000

H	-13.88850500	5.12756400	0.21969000
C	-14.12636700	7.20162700	0.75185700
H	-13.86569500	8.18556500	0.34017500
H	-13.59572900	7.12512100	1.71025400
C	-15.63032900	7.14102300	1.00145000
H	-16.16139700	7.21799700	0.04333600
H	-15.89143300	6.15666400	1.41276900
C	-16.13329800	8.23027700	1.94490800
H	-15.87240900	9.21276600	1.53243000
H	-15.60166700	8.15235800	2.90144800
C	-17.63756700	8.15872700	2.18682400
H	-17.97514300	8.94740400	2.86464900
H	-18.19155600	8.26520800	1.24873000
H	-17.91920800	7.19693600	2.62731900
O	-2.12014900	-4.03235600	-1.69978000
O	-1.91023200	-1.85302300	-1.15854800
O	-4.04029100	-4.90300800	0.43718400
O	-5.12989400	-3.31931200	-1.19430500
O	-6.58108000	-4.75586100	0.35675500
O	-5.21385900	-5.83323500	-1.58226800
C	-6.29973700	-2.64942800	-1.70094200
H	-7.10841200	-2.72041600	-0.96920900
H	-6.02071000	-1.60153900	-1.80707000
C	-6.70986600	-3.18919000	-3.05860300
H	-5.85288700	-3.14181300	-3.73530000
H	-7.49133500	-2.52948700	-3.45914700

N	-7.14369800	-4.59367900	-2.97268700
H	-7.93726700	-4.67732300	-2.33962900
H	-7.44471500	-4.93119800	-3.88187200
P	-5.33780900	-4.73822600	-0.45256000
H	-5.90159800	-5.57000500	-2.27515000
O	11.03221500	-0.85983100	1.59452300
H	11.79397800	-0.32552100	1.31620900

AmB-DSPE5

0 1

C	5.97553500	1.78255100	-3.06338400
H	6.79297800	1.10286200	-2.79036600
C	6.25740100	3.10999200	-2.36143700
H	5.45869000	3.82249200	-2.62285300
C	6.22011500	2.91965000	-0.84989900
H	6.96713900	2.15403000	-0.57812200
C	4.62880600	1.19331600	-1.04252500
H	5.37053900	0.46183500	-0.67116700
C	4.66416500	1.17811100	-2.57088300
H	4.59724900	0.12810800	-2.88695100
C	6.50016700	4.19156800	-0.08504200
H	5.78211300	4.96460800	-0.36291600
H	7.50294200	4.54693500	-0.32696000
H	6.42657200	4.01299300	0.98999400

C	3.00618400	0.79459500	0.71260600
H	3.17803800	1.78818000	1.13895300
C	1.55017500	0.47156200	0.72759500
H	1.26080100	-0.37817200	0.12064400
C	0.61718200	1.17885300	1.37148800
H	0.89930400	2.04821000	1.96493000
C	-0.78933300	0.85121800	1.26640600
H	-1.04195700	0.00639200	0.63177300
C	-1.79886700	1.52611400	1.84796800
H	-1.59238900	2.37719900	2.49647300
C	-3.17466100	1.16060100	1.61296400
H	-3.33363100	0.33930000	0.91645100
C	-4.24564000	1.76589600	2.16590300
H	-4.10595900	2.59341800	2.86072700
C	-5.59380800	1.36910200	1.85443600
H	-5.70149700	0.54081800	1.15410500
C	-6.71098900	1.95194300	2.34193100
H	-6.62807900	2.78092000	3.04360500
C	-8.03013100	1.54569700	1.92959800
H	-8.07308300	0.70004500	1.24580000
C	-9.19383300	2.12522200	2.28978600
H	-9.18792000	2.95185300	2.99903600
C	-10.46721900	1.71218200	1.75013600
H	-10.43618000	0.96249900	0.96092300
C	-11.67104900	2.17228400	2.13624700
H	-11.73905600	2.89636300	2.94709100

C	3.88562000	-0.21270600	1.46391900
H	4.88873300	0.20875900	1.56393000
H	3.49448700	-0.32881100	2.47867100
C	-12.90696800	1.71110300	1.53248700
H	-12.79098100	1.11672400	0.62842200
C	-14.13396900	1.91885900	2.02130900
H	-14.25134200	2.49614700	2.93963900
C	-15.39801700	1.37192900	1.41812800
H	-15.19026000	1.04429200	0.39313500
C	-15.90372600	0.14270700	2.19903900
H	-16.18418400	0.49071900	3.20711400
C	-14.86562600	-0.97903100	2.39652600
H	-14.01797300	-0.51526200	2.90981600
C	-14.29751700	-1.52274400	1.07979900
H	-14.00890700	-0.69829200	0.43297500
C	-11.93701100	-1.90745300	0.83216900
C	-10.76700200	-2.73464900	1.29905900
H	-11.00295000	-3.19432600	2.26019200
H	-10.62308600	-3.53796100	0.56725800
C	-9.47653000	-1.91301700	1.38528000
H	-9.62465300	-1.09590100	2.10791900
C	-8.31325700	-2.77605800	1.84442800
H	-8.54143500	-3.15611900	2.84635400
H	-8.21357200	-3.63960500	1.17642400
C	-6.96962800	-2.05287700	1.88245700
H	-7.09209200	-1.07905200	2.37758600

C	-5.93577700	-2.86441100	2.65961000
H	-6.00633900	-3.91301300	2.34665000
H	-6.21866400	-2.83128500	3.71617100
C	-4.49340300	-2.36907900	2.46920300
H	-4.48764800	-1.34297800	2.09338100
H	-3.97043000	-2.34790600	3.43008700
C	-3.67741200	-3.25442400	1.52829000
H	-3.52845300	-4.22652400	2.01941300
C	-2.29250600	-2.64445400	1.21962000
H	-1.95750000	-2.04477600	2.07644400
C	-1.24476600	-3.72105100	0.94814400
H	-1.60094500	-4.39762200	0.16382900
H	-1.12995000	-4.31259800	1.86499500
C	0.11148500	-3.15954500	0.52114600
H	0.28622400	-2.20271500	1.02737600
C	1.27073300	-4.08841000	0.87296200
H	1.12539400	-5.05589000	0.38120800
H	1.28123000	-4.25471200	1.95345200
C	2.63738700	-3.54595800	0.44924200
C	3.78944300	-4.45484800	0.87435900
H	3.72556600	-5.40141800	0.33375000
H	3.72263500	-4.66096300	1.94688200
C	5.11648300	-3.78532600	0.57336000
H	5.17638900	-3.61165100	-0.50477000
C	5.16890200	-2.42899500	1.28474800
H	5.08179900	-2.57587100	2.36487800

C	3.96559700	-1.58272600	0.80750000
H	4.04013600	-1.46619300	-0.27890000
C	6.46921800	-1.70390900	1.06257100
C	-15.16949400	-2.49280400	0.30888600
H	-14.68235600	-2.77030300	-0.62875200
H	-16.12618100	-2.01666600	0.09138400
H	-15.34918600	-3.40334600	0.88441000
C	-15.41073400	-2.08613500	3.29786700
H	-16.30931200	-2.54963400	2.87948300
H	-15.66376300	-1.68993800	4.28539400
H	-14.66861000	-2.87614700	3.43202800
C	-16.48749600	2.44616600	1.36007300
H	-16.72102100	2.81253900	2.36602600
H	-17.40187300	2.04257200	0.92446000
H	-16.15473100	3.29879800	0.76352300
O	3.60309200	1.92606600	-3.12361600
H	2.81743900	1.74804100	-2.58720400
O	7.52612300	3.58581800	-2.74290300
H	7.57015000	3.40702900	-3.69636900
O	4.91790700	2.45520000	-0.49316200
O	3.33414100	0.83486600	-0.69004100
O	2.72116500	-3.39001800	-0.93783400
H	1.81889300	-3.12310900	-1.22379400
O	2.76870300	-2.28214300	1.11032900
O	7.03805300	-1.00835800	1.86607600
O	0.14757900	-2.96248500	-0.89483800

H	-0.19149800	-2.07285600	-1.11908700
O	-2.42373600	-1.74613800	0.13036900
H	-3.18419400	-2.07676200	-0.38291600
O	-4.32022800	-3.50198000	0.28777200
H	-5.11179900	-2.93161000	0.21098600
O	-6.45156700	-1.84010700	0.57254300
H	-7.14883500	-1.54641100	-0.03641200
O	-9.14422600	-1.35770700	0.11659300
H	-9.97766000	-1.03615400	-0.27245400
O	-11.83330100	-1.04336800	-0.02642000
O	-13.06805800	-2.23155700	1.43779900
O	-17.06371400	-0.31969500	1.51387500
H	-17.56569600	-0.89022700	2.10558400
O	6.16353800	-4.64403200	0.99588100
H	6.96761000	-4.37155900	0.53767100
N	6.04394900	2.00796300	-4.50286600
H	5.16411600	2.42325600	-4.80107400
H	6.13786500	1.12868100	-5.00125000
C	15.81067700	-10.60556700	4.79520300
H	15.07217700	-10.75086300	5.59339400
H	15.76622100	-11.50820100	4.17316700
C	15.40862600	-9.39688300	3.95431400
H	16.14717800	-9.25001900	3.15489900
H	15.45266900	-8.49254900	4.57582200
C	14.01728500	-9.52094000	3.34056300
H	13.27948500	-9.66838400	4.14034600

H	13.97388700	-10.42514000	2.71897100
C	13.61560500	-8.31184300	2.50092200
H	13.65882000	-7.40777400	3.12259100
H	14.35320500	-8.16406900	1.70097800
C	12.22398200	-8.43609600	1.88769000
H	11.48664900	-8.58468700	2.68758400
H	12.18075600	-9.33959600	1.26511200
C	11.82137200	-7.22614700	1.04980700
H	11.86495700	-6.32297800	1.67281100
H	12.55817500	-7.07722100	0.24928400
C	10.42897800	-7.35012100	0.43846200
H	9.69237500	-7.49953400	1.23864400
H	10.38486400	-8.25233000	-0.18581300
C	10.02435000	-6.13924100	-0.39682500
H	10.07319500	-5.23718500	0.22858700
H	10.75736600	-5.98869000	-1.20043500
C	8.62771400	-6.26045700	-0.99863700
H	7.89558700	-6.41592000	-0.19534800
H	8.57803500	-7.15647900	-1.63066600
C	8.21168100	-5.04196000	-1.81817500
H	8.26881100	-4.14160000	-1.18755700
H	8.93316400	-4.87370700	-2.62808800
C	6.80124300	-5.15557200	-2.39107200
H	6.11934900	-5.44004100	-1.58033500
H	6.76277700	-5.97450900	-3.12048800
C	6.30217700	-3.85953700	-3.02403300

H	6.94046000	-3.58699200	-3.87463700
H	6.41981200	-3.05255100	-2.29114100
C	4.83779500	-3.91372300	-3.46126700
H	4.25375400	-4.46449200	-2.71547100
H	4.74211600	-4.46216500	-4.40696700
C	4.22083900	-2.52471300	-3.58107600
H	4.82173500	-1.89549800	-4.25274300
H	4.26368300	-2.06202500	-2.58895600
C	2.76845700	-2.53818700	-4.04384600
H	2.22208600	-3.31511100	-3.50369500
H	2.71526400	-2.80314500	-5.10650300
C	2.08275900	-1.19255100	-3.80463600
H	2.61813700	-0.35959900	-4.26749000
H	2.05126700	-0.98563700	-2.72676000
C	0.65984000	-1.11481500	-4.27887300
O	0.14691100	-0.15078400	-4.79670900
C	17.20293900	-10.46990100	5.40327700
H	17.46955500	-11.34594700	6.00080600
H	17.96236500	-10.35410800	4.62339300
H	17.26334200	-9.59137600	6.05356100
C	-1.41145000	-2.28356900	-4.30266100
H	-1.63233100	-1.62996500	-5.14944600
H	-1.63676400	-3.32120600	-4.56032300
C	-2.24380800	-1.86422700	-3.10295400
H	-1.85158200	-2.31582600	-2.19269100
C	-3.70367600	-2.24600800	-3.28737000

H	-4.07723500	-1.88449000	-4.24863500
H	-3.81413800	-3.32990600	-3.22573600
O	-0.01436100	-2.25674700	-4.01028300
C	-1.27017300	0.13990200	-2.26129000
C	-1.34945700	1.63438000	-2.22164600
H	-1.97360500	1.98722900	-3.04426000
H	-1.86554800	1.87960100	-1.28487000
C	0.05799100	2.23311500	-2.22852600
H	0.48723600	2.11086400	-3.23026300
H	0.67103100	1.64253800	-1.54293300
C	0.12332200	3.68964500	-1.78913400
H	-0.41645800	4.33313000	-2.49576300
H	-0.38762200	3.79273800	-0.82235500
C	1.56722300	4.16229400	-1.64408000
H	2.09109800	4.05780100	-2.60231300
H	2.09449000	3.49379500	-0.95098700
C	1.70000100	5.59748500	-1.14813400
H	1.11305900	5.72186700	-0.22795600
H	1.26236400	6.28635200	-1.88281100
C	3.14882600	5.98558700	-0.87374600
H	3.55940300	5.29833700	-0.12388700
H	3.74492100	5.82566800	-1.78242500
C	3.32362800	7.42513200	-0.40152500
H	2.70697300	7.59350100	0.49152200
H	2.93793500	8.10963600	-1.16873200
C	4.77361800	7.78227500	-0.08721500

H	5.14902000	7.11383500	0.69870500
H	5.39571000	7.58682600	-0.97082100
C	4.96320400	9.23073900	0.35242600
H	4.33982800	9.42745400	1.23487200
H	4.59300500	9.90088000	-0.43476900
C	6.41416800	9.57904100	0.67106200
H	6.78372300	8.90845000	1.45809800
H	7.03675800	9.38029600	-0.21120000
C	6.60783500	11.02742700	1.10991600
H	6.23778600	11.69788300	0.32289800
H	5.98580400	11.22581300	1.99289200
C	8.05938500	11.37476700	1.42696000
H	8.42958700	10.70416100	2.21369000
H	8.68108600	11.17634100	0.54392700
C	8.25375800	12.82312900	1.86580500
H	7.88296700	13.49366900	1.07923600
H	7.63254300	13.02135300	2.74934300
C	9.70559300	13.17050600	2.18166400
H	10.32655500	12.97233000	1.29799300
H	10.07643400	12.49973300	2.96799300
C	9.90032000	14.61870900	2.62064300
H	9.52947500	15.29007900	1.83461200
H	9.27988800	14.81742400	3.50478700
C	11.35247700	14.96700200	2.93620300
H	11.97096700	14.76826900	2.05214400
H	11.72162400	14.29561400	3.72142600

C	11.53437500	16.41680400	3.37386800
H	12.58103600	16.64361200	3.59442900
H	11.20061400	17.10743200	2.59284000
H	10.94947700	16.63145300	4.27398000
O	-2.23558300	-0.43246300	-2.97393500
O	-0.39430000	-0.48879100	-1.69453900
O	-4.48603000	-1.69141100	-2.22460500
O	-6.75424800	-0.66124900	-2.07898500
O	-5.11734500	0.25620800	-3.84149300
O	-4.72144800	0.59881300	-1.26752100
C	-7.78897600	0.18620500	-2.59572100
H	-7.47634900	0.58660400	-3.56438800
H	-8.66643200	-0.44770800	-2.74404900
C	-8.12771500	1.28850700	-1.60682400
H	-8.46168100	0.81640300	-0.68577100
H	-8.97079600	1.86933400	-2.00527900
N	-6.96346400	2.12748700	-1.28547100
H	-6.79783600	2.81353400	-2.01710200
H	-7.12535500	2.63111000	-0.41761300
P	-5.22319300	-0.28905700	-2.47522600
H	-5.45887300	1.26833200	-1.10524100
O	6.95613700	-1.87119100	-0.19440500
H	7.77249800	-1.34781400	-0.24310400

AmB-DSPE6

0 1

C	17.57677100	-5.49773100	-1.94692900
H	18.32922600	-4.74540400	-1.68862200
C	17.64645900	-5.74773100	-3.44816000
H	16.89508800	-6.49986500	-3.71999300
C	17.33846300	-4.43657300	-4.20293400
H	18.15895100	-3.73928400	-3.96226300
C	16.05938700	-3.61269900	-2.41696800
H	16.88051100	-2.92901800	-2.15291700
C	16.21441400	-4.91447200	-1.64906300
H	16.11566100	-4.69926900	-0.59105300
C	17.26089800	-4.63662000	-5.70154800
H	16.45983200	-5.34008700	-5.94482500
H	18.20838600	-5.01916300	-6.08969100
H	17.04578800	-3.68836900	-6.19703600
C	14.84161600	-1.80021100	-1.39938500
H	15.29220300	-1.05521600	-2.07041300
C	13.40721100	-1.45075000	-1.15531800
H	12.85789300	-2.10255300	-0.48100200
C	12.79738400	-0.39396100	-1.70014300
H	13.35028700	0.26012300	-2.37411600
C	11.40696500	-0.06773200	-1.45286600
H	10.87334600	-0.71574000	-0.76190100
C	10.74683500	0.95644000	-2.02587900
H	11.27039700	1.60894500	-2.72436700

C	9.35358100	1.23012100	-1.76618900
H	8.86910100	0.57885600	-1.04162300
C	8.63796800	2.22053100	-2.33602600
H	9.11101700	2.88228400	-3.06100700
C	7.24685500	2.44979200	-2.03378700
H	6.79513500	1.80524000	-1.27936300
C	6.48059700	3.40229200	-2.60437500
H	6.91672500	4.06604700	-3.34997700
C	5.08593300	3.58227600	-2.28977900
H	4.66469900	2.90126100	-1.55188600
C	4.28084600	4.51791600	-2.83326000
H	4.69342000	5.21991900	-3.55703200
C	2.88138700	4.64449600	-2.50258700
H	2.47318300	3.89644900	-1.82411200
C	2.06894100	5.62383600	-2.94280200
H	2.46447500	6.40513500	-3.59080800
C	15.67564900	-1.83551500	-0.10428500
H	16.71696100	-2.05844200	-0.33656000
H	15.66235000	-0.83091500	0.32864200
C	0.67719600	5.71071900	-2.54571000
H	0.29957200	4.85399400	-1.99495900
C	-0.13777200	6.75341200	-2.73563800
H	0.22229400	7.63698000	-3.26436800
C	-1.55162300	6.79683300	-2.23071200
H	-1.79182000	5.81164200	-1.81351400
C	-1.74206100	7.82123300	-1.09440800

H	-1.69439300	8.82552800	-1.54600300
C	-0.68385100	7.78157800	0.02475000
H	0.28714400	7.83001000	-0.47729900
C	-0.68783700	6.44892300	0.78293600
H	-0.82490300	5.65071200	0.06031700
C	1.31856900	5.17023600	1.01532900
C	2.59055700	5.04361200	1.80735100
H	2.95455600	6.04328000	2.05417300
H	2.31833200	4.55270800	2.74885400
C	3.68483500	4.23879600	1.10929700
H	3.86623300	4.66461900	0.11606900
C	4.98172300	4.28892300	1.91748300
H	5.28645500	5.33378000	2.04453200
H	4.79518900	3.87543100	2.91649800
C	6.13325300	3.52099900	1.27494200
H	6.30286500	3.90628300	0.25955400
C	7.42568200	3.66366500	2.07129800
H	7.21324300	3.46915300	3.12969800
H	7.74344600	4.70861200	2.00184500
C	8.54697700	2.73156800	1.58283700
H	8.35769800	2.41816600	0.55310100
H	9.50128600	3.26709500	1.56630600
C	8.74688700	1.49410600	2.45628700
H	9.12530600	1.81829900	3.43573400
C	9.77203900	0.51802900	1.82903300
H	10.45681800	1.06666800	1.17208500

C	10.57896600	-0.25184000	2.86877800
H	9.89203700	-0.82553800	3.50265800
H	11.10244500	0.46450400	3.51223800
C	11.60682500	-1.18589200	2.23680200
H	12.26443700	-0.59970100	1.58073400
C	12.47208400	-1.87986700	3.28174700
H	11.83658600	-2.44377900	3.97154600
H	13.00823700	-1.11782800	3.85464100
C	13.49020400	-2.84845700	2.67909700
C	14.48021300	-3.38895700	3.70628400
H	13.95951900	-4.01909900	4.43027900
H	14.94210900	-2.55336600	4.24079000
C	15.56235700	-4.19630900	3.01361300
H	15.09124200	-5.05545700	2.52320400
C	16.25007300	-3.34851200	1.93554800
H	16.73611800	-2.48820600	2.40732200
C	15.16786600	-2.80120200	0.96213000
H	14.66606200	-3.65450400	0.49090200
C	17.33240100	-4.11772500	1.19580500
C	-1.68802300	6.30104400	1.91442400
H	-1.62154100	5.29286600	2.32850600
H	-2.69791700	6.47609000	1.54133900
H	-1.47961100	7.01288300	2.71585300
C	-0.79709400	8.99165800	0.94975100
H	-1.77766900	9.05014800	1.43345500
H	-0.64040300	9.92080700	0.39485000

H	-0.04736100	8.94146000	1.74284300
C	-2.54414200	7.08177400	-3.36234800
H	-2.31760000	8.04070500	-3.84102600
H	-3.56406700	7.12522000	-2.97675500
H	-2.48677200	6.30527800	-4.12839900
O	15.22816300	-5.85478200	-2.03018800
H	14.38299200	-5.38468500	-2.02935900
O	18.96239700	-6.19916800	-3.73350700
H	18.95862200	-6.63839400	-4.59033400
O	16.09556300	-3.86608000	-3.80088100
O	14.81923600	-3.06747200	-2.08790900
O	12.86800400	-3.94891300	2.07960300
H	12.07342500	-3.60671800	1.63043800
O	14.20574800	-2.07724100	1.71558500
O	18.16880400	-3.58657100	0.49329300
O	17.23035900	-5.44479600	1.32551400
O	10.96552200	-2.19607900	1.45242600
H	10.13808300	-1.81443300	1.10392800
O	9.05091400	-0.39109500	0.99357500
H	8.17731300	-0.43664500	1.43622700
O	7.55388200	0.75558400	2.67482100
H	6.81382000	1.18765200	2.19317600
O	5.81871000	2.12840100	1.17439300
H	4.84531700	2.07003400	1.08032600
O	3.30337500	2.87613600	0.90066700
H	2.54639200	2.64331700	1.48790300

O	0.94406300	4.37327400	0.18322200
O	0.62975800	6.26404300	1.36601900
O	-3.05894800	7.58620200	-0.59202700
H	-3.32337300	8.33368000	-0.04442200
O	16.48879300	-4.62874600	3.99361900
H	17.01428000	-5.32566400	3.58041400
N	17.83096900	-6.65407400	-1.08569800
H	17.07070200	-7.32185300	-1.20029600
H	17.69783500	-5.91529200	0.56650100
H	18.68926500	-7.10652100	-1.38916100
C	-24.79356500	-4.79234900	-1.99114900
H	-24.72163700	-4.95442300	-3.07364100
H	-24.37540800	-5.69049000	-1.52136300
C	-23.94252200	-3.58502100	-1.60741100
H	-24.05184900	-3.39188800	-0.53166800
H	-24.32902700	-2.69048700	-2.11337400
C	-22.46402200	-3.76044400	-1.93860300
H	-22.35046300	-3.94985200	-3.01401500
H	-22.08555400	-4.65788000	-1.43219700
C	-21.60721500	-2.56228200	-1.54238400
H	-21.97341000	-1.66469100	-2.05764900
H	-21.73346700	-2.36577400	-0.46916600
C	-20.12511600	-2.75418800	-1.84771300
H	-19.99591900	-2.94403900	-2.92125600
H	-19.76430100	-3.65559000	-1.33572300
C	-19.26377700	-1.56415900	-1.43723000

H	-19.62432400	-0.66031600	-1.94557400
H	-19.39294500	-1.37685500	-0.36271300
C	-17.78156400	-1.75863200	-1.74047600
H	-17.65037700	-1.93554700	-2.81597400
H	-17.42527200	-2.66750800	-1.23883700
C	-16.91750900	-0.57651100	-1.31317400
H	-17.27879400	0.33589600	-1.80544500
H	-17.04331900	-0.40669900	-0.23536200
C	-15.43632300	-0.76642400	-1.62446000
H	-15.30816100	-0.91937800	-2.70395900
H	-15.07964200	-1.68653800	-1.14410800
C	-14.57048500	0.40520500	-1.17265000
H	-14.93635500	1.32973300	-1.63816600
H	-14.68908900	0.54656200	-0.08992300
C	-13.09110200	0.22674300	-1.49924500
H	-12.96927000	0.11170400	-2.58410100
H	-12.73056300	-0.70912100	-1.05326900
C	-12.22465500	1.38330200	-1.01125100
H	-12.33109000	1.48029300	0.07763800
H	-12.60011700	2.32407400	-1.43461400
C	-10.74850200	1.22655100	-1.36256000
H	-10.63717200	1.16676000	-2.45271800
H	-10.37869000	0.27120000	-0.96781600
C	-9.88262400	2.36185600	-0.82574800
H	-9.97466300	2.39901300	0.26831700
H	-10.27063900	3.32130500	-1.19227800

C	-8.41333400	2.22939300	-1.21066100
H	-8.30258700	2.23843100	-2.29913300
H	-8.02811100	1.25706400	-0.88209100
C	-7.55543500	3.33601600	-0.61287000
H	-7.60027600	3.33675200	0.48111100
H	-7.91785300	4.32353800	-0.92365200
C	-6.10986800	3.23856700	-1.02534900
O	-5.68336100	2.55166300	-1.91835100
C	-26.25615300	-4.64215800	-1.58692200
H	-26.84691700	-5.51420200	-1.88006800
H	-26.35144000	-4.52418400	-0.50260100
H	-26.70625200	-3.76066700	-2.05456700
C	-3.93310700	4.00356700	-0.47089900
H	-3.71946400	3.61502700	-1.46946000
H	-3.56774000	5.02938700	-0.38160600
C	-3.29747100	3.12518600	0.59432300
H	-3.48687000	3.53094800	1.59078100
C	-1.79810400	2.98002000	0.38270800
H	-1.59590500	2.07214600	-0.18782700
H	-1.39040800	3.82649000	-0.16975200
O	-5.34224900	4.05090200	-0.26004100
C	-4.00777900	1.03860200	1.54150200
C	-4.90278200	-0.13665200	1.28153400
H	-4.82175400	-0.41576400	0.22820700
H	-4.56538500	-0.96819700	1.90319500
C	-6.35923400	0.22339500	1.61179100

H	-6.63188300	1.13832100	1.07461700
H	-6.44119100	0.44707500	2.68128500
C	-7.32326400	-0.89532000	1.23283400
H	-7.25811200	-1.07193100	0.15129300
H	-7.00541000	-1.82882400	1.71437900
C	-8.77171400	-0.60060400	1.61138200
H	-9.08526100	0.34532700	1.15196900
H	-8.84261700	-0.45105400	2.69634300
C	-9.72698000	-1.71172600	1.18661100
H	-9.39689900	-2.66036900	1.62973800
H	-9.66036100	-1.84858800	0.09894900
C	-11.18035300	-1.45566900	1.57275500
H	-11.25005300	-1.31130700	2.65872000
H	-11.51901900	-0.51564600	1.11888500
C	-12.11246700	-2.58756600	1.15120700
H	-11.77154500	-3.52534400	1.60915500
H	-12.03238200	-2.73719400	0.06606600
C	-13.57362100	-2.34954800	1.51888900
H	-13.65605300	-2.18665000	2.60139700
H	-13.92217100	-1.42294600	1.04527800
C	-14.48598700	-3.50190400	1.10972400
H	-14.13960900	-4.42588100	1.59105600
H	-14.39116400	-3.67284000	0.02891500
C	-15.95391600	-3.27252800	1.45545700
H	-16.05045700	-3.08934400	2.53357900
H	-16.30561400	-2.35850800	0.96012400

C	-16.85193100	-4.44046300	1.05888900
H	-16.74607800	-4.62988800	-0.01780500
H	-16.50225700	-5.35261000	1.56001000
C	-18.32451400	-4.21628300	1.38804300
H	-18.43118400	-4.01495900	2.46198900
H	-18.67819100	-3.31329300	0.87413100
C	-19.21246700	-5.39641200	1.00502500
H	-19.09924800	-5.60233800	-0.06788400
H	-18.86046300	-6.29822300	1.52294100
C	-20.68808700	-5.17457400	1.32219000
H	-21.04233600	-4.28114100	0.79218400
H	-20.80169300	-4.95666200	2.39221100
C	-21.57023500	-6.36338000	0.95295700
H	-21.44827900	-6.58850500	-0.11530200
H	-21.22219800	-7.25578200	1.48980500
C	-23.04940100	-6.13722000	1.25243400
H	-23.39502400	-5.25267600	0.70481700
H	-23.17117400	-5.90168000	2.31684800
C	-23.91973300	-7.33603600	0.89007900
H	-24.97752800	-7.14022900	1.08603400
H	-23.81923500	-7.58872700	-0.17041200
H	-23.63011600	-8.22077100	1.46577800
O	-3.95882300	1.84921100	0.48452100
O	-3.44605300	1.26780700	2.59547600
O	-1.13801800	2.95010300	1.64493800
O	-0.18886500	0.68365200	1.05877600

O	-0.88417200	1.25203600	3.44038500
H	-1.85682600	1.22325200	3.30270300
O	1.23036100	2.30184800	2.49008100
C	1.00490800	0.31111000	0.32634400
H	1.86867200	0.38260400	0.99130900
H	0.87055600	-0.73175600	0.04075500
C	1.19961200	1.20138400	-0.89066800
H	1.06649700	2.24689900	-0.58842600
H	0.43112100	0.96494300	-1.63409000
N	2.51587500	0.91337400	-1.44525900
H	2.60004400	1.28077900	-2.38661100
H	3.20779800	1.39570100	-0.87868800
P	-0.13604400	1.82065500	2.17585900

AmB-DSPE7

0 1

C	4.38343300	2.08353600	-5.20660100
H	4.22594900	2.84317800	-4.43660500
C	4.01121800	2.70030800	-6.55753600
H	4.19488100	1.96666400	-7.35174800
C	2.51774500	3.09149100	-6.56218400
H	2.41133900	3.92039900	-5.84179800
C	2.01099000	1.54288000	-4.85290400
H	2.02337500	2.39447000	-4.15806000

C	3.40198800	0.94149300	-4.96058500
H	3.64794800	0.41615800	-4.04555000
C	2.04677300	3.53326500	-7.93018500
H	2.12234700	2.70101300	-8.63514500
H	2.66422600	4.35693400	-8.29159600
H	1.00440300	3.85313700	-7.88515400
C	0.38007500	0.82839000	-3.23890300
H	-0.21421500	1.74552700	-3.35901100
C	-0.53384500	-0.34281200	-3.06237400
H	-0.05648900	-1.31136500	-2.93842400
C	-1.86573400	-0.24704600	-3.01898400
H	-2.34165800	0.72567700	-3.14203100
C	-2.73578900	-1.38927800	-2.82127100
H	-2.24799600	-2.35025100	-2.67608500
C	-4.08111600	-1.33477200	-2.81598100
H	-4.58002900	-0.37728600	-2.96442400
C	-4.90938400	-2.50167500	-2.62764400
H	-4.37990600	-3.43615700	-2.45373900
C	-6.25752100	-2.50632200	-2.63353100
H	-6.80144200	-1.57718900	-2.80120800
C	-7.03339700	-3.70391300	-2.42563800
H	-6.47803800	-4.61999800	-2.22516100
C	-8.38070000	-3.76280200	-2.44508300
H	-8.95233100	-2.85376400	-2.62916200
C	-9.12404800	-4.98252700	-2.25078800
H	-8.54476100	-5.88947600	-2.07865000

C	-10.46946300	-5.06957900	-2.26557700
H	-11.05947800	-4.16617100	-2.41617000
C	-11.18859500	-6.30906000	-2.09740800
H	-10.58866200	-7.21318200	-2.00871700
C	-12.52766400	-6.42109200	-2.02648200
H	-13.14550800	-5.52583200	-2.08677000
C	1.32292300	1.02269400	-2.03597300
H	1.92207200	1.92145900	-2.17554200
H	0.70923600	1.19261500	-1.14678200
C	-13.20099200	-7.68958300	-1.82717300
H	-12.56556400	-8.57175700	-1.87269900
C	-14.49957100	-7.83512200	-1.54227200
H	-15.13305200	-6.94870700	-1.47965100
C	-15.16845700	-9.14666000	-1.24427700
H	-14.47152500	-9.96127900	-1.47220700
C	-15.52720700	-9.26085900	0.25030400
H	-16.33705900	-8.53907600	0.44851100
C	-14.38394900	-8.91284900	1.22164100
H	-14.08890000	-7.88737200	0.97895200
C	-13.12514800	-9.75896900	1.00166800
H	-12.89869300	-9.81316800	-0.05954100
C	-10.93257000	-8.78701800	0.92674200
C	-9.90365600	-8.00654200	1.70548100
H	-10.39962000	-7.43757800	2.49408300
H	-9.23882800	-8.73381800	2.18594900
C	-9.06869600	-7.09188900	0.80602000

H	-9.73256200	-6.36441000	0.31749700
C	-8.01112200	-6.34800800	1.60873200
H	-8.51149800	-5.74147200	2.37124300
H	-7.37849400	-7.07851600	2.12713500
C	-7.11292700	-5.44302700	0.76892600
H	-7.73475500	-4.76090900	0.17325400
C	-6.17928500	-4.61732200	1.64790200
H	-5.71327900	-5.27900000	2.38847500
H	-6.79663600	-3.90781600	2.20726500
C	-5.09145800	-3.86966300	0.85952800
H	-5.38857500	-3.75628600	-0.18577400
H	-4.97424100	-2.85535000	1.25325700
C	-3.71651100	-4.53224900	0.92436600
H	-3.35517000	-4.47865400	1.96076100
C	-2.69585800	-3.81170500	0.00924500
H	-3.00699400	-2.77267000	-0.14878700
C	-1.27524100	-3.83174700	0.56378500
H	-0.95013000	-4.87249400	0.68230900
H	-1.27654800	-3.36993400	1.55779800
C	-0.29264000	-3.07735600	-0.32656900
H	-0.65452100	-2.04917800	-0.46222400
C	1.10486000	-3.01382700	0.27888600
H	1.48011100	-4.02771200	0.44870100
H	1.04657600	-2.50198800	1.24379100
C	2.11063500	-2.27876900	-0.60732300
C	3.45761700	-2.04575100	0.07401400

H	3.95905700	-3.00084800	0.24275000
H	3.29976100	-1.55771500	1.04055500
C	4.32542900	-1.16179500	-0.80250100
H	4.49199900	-1.67922700	-1.75280800
C	3.60543000	0.15916600	-1.09784000
H	3.40700200	0.68888500	-0.15976900
C	2.23243700	-0.17080900	-1.75436200
H	2.42302600	-0.71979100	-2.68433500
C	4.42328600	1.08825600	-1.97247500
C	-13.13101700	-11.15476400	1.59057300
H	-12.21165200	-11.67894600	1.31967500
H	-13.98757900	-11.70311800	1.19615000
H	-13.20099100	-11.12302200	2.67998600
C	-14.85978600	-8.93293100	2.67321700
H	-15.23687200	-9.91746700	2.96600200
H	-15.66123200	-8.20459900	2.82721000
H	-14.04153300	-8.68149500	3.35173100
C	-16.42220100	-9.34140400	-2.10161900
H	-17.13719900	-8.52910500	-1.92986000
H	-16.91040100	-10.28472600	-1.85309200
H	-16.16677200	-9.34202200	-3.16366900
O	3.46844400	-0.01383000	-6.00314900
H	2.72458000	0.17039600	-6.59482600
O	4.73597300	3.89473900	-6.84617800
H	5.68627400	3.74362800	-6.67639500
O	1.67166700	2.01525700	-6.15110300

O	1.09701900	0.58022800	-4.46441800
O	2.36629300	-2.97366000	-1.79287000
H	1.51371000	-3.33791100	-2.09407100
O	1.50663400	-1.01386300	-0.87220100
O	4.10516400	2.25126200	-2.16239900
O	5.45374000	0.51301000	-2.57799400
O	-0.18795600	-3.69705800	-1.61149700
H	-1.04910900	-4.10938100	-1.81016200
O	-2.73400200	-4.44409200	-1.27212200
H	-2.98848100	-5.36359700	-1.04842600
O	-3.72706100	-5.89972700	0.53968400
H	-4.64032600	-6.15811200	0.29005300
O	-6.30773200	-6.21340000	-0.12796400
H	-6.84677500	-6.96536100	-0.43472500
O	-8.39348700	-7.85438600	-0.19103100
H	-9.05088300	-8.45496300	-0.58572400
O	-10.74898700	-9.17551000	-0.21616300
O	-12.03337400	-9.01408000	1.62795100
O	-16.01088000	-10.58653900	0.44426100
H	-16.50934800	-10.61666900	1.26782300
O	5.56877300	-0.94232500	-0.14829300
H	6.19678100	-0.70781400	-0.84290900
N	5.74984200	1.59032800	-5.02860500
H	5.92824800	0.82717700	-5.67730000
H	5.72857600	1.03232600	-3.41843500
H	6.43759000	2.33307400	-5.15786000

C	-11.69090500	18.49119200	7.09516400
H	-12.44018100	17.81571700	6.66393200
H	-11.53059400	18.14828200	8.12489300
C	-10.38670400	18.35770600	6.31357100
H	-9.63561900	19.03334800	6.74417900
H	-10.54557700	18.70080000	5.28250300
C	-9.83479400	16.93540700	6.29357000
H	-10.58624800	16.26042000	5.86283400
H	-9.67664900	16.59280100	7.32479100
C	-8.53076800	16.80246300	5.51249100
H	-8.68853100	17.14532300	4.48133500
H	-7.77901800	17.47688600	5.94345900
C	-7.97982100	15.37971200	5.49239500
H	-8.73147600	14.70547000	5.06089800
H	-7.82277700	15.03664700	6.52360600
C	-6.67531800	15.24613800	4.71228100
H	-6.83191700	15.58980100	3.68121000
H	-5.92328400	15.91957000	5.14431100
C	-6.12585100	13.82282300	4.69181300
H	-6.87777400	13.14971700	4.25901200
H	-5.97038600	13.47888200	5.72296800
C	-4.82059600	13.68818500	3.91314400
H	-4.97544700	14.03298500	2.88218800
H	-4.06812700	14.36013200	4.34672100
C	-4.27330600	12.26404200	3.89215700
H	-5.02562400	11.59253800	3.45757600

H	-4.11994400	11.91887300	4.92320300
C	-2.96707900	12.12796900	3.11537500
H	-3.11964500	12.47424100	2.08457000
H	-2.21402000	12.79793600	3.55098000
C	-2.42274500	10.70271400	3.09375300
H	-3.17556600	10.03327200	2.65695000
H	-2.27192100	10.35596200	4.12459700
C	-1.11537400	10.56486800	2.31921800
H	-0.36156200	11.23234300	2.75733600
H	-1.26522600	10.91294600	1.28861100
C	-0.57521200	9.13813500	2.29706900
H	-1.32818200	8.47094900	1.85753700
H	-0.42675200	8.78915100	3.32728300
C	0.73329200	8.99808600	1.52499300
H	1.48794900	9.66292700	1.96594600
H	0.58630600	9.34882700	0.49471300
C	1.26201000	7.56781800	1.50631500
H	0.51671800	6.89763200	1.06504100
H	1.41289000	7.20922400	2.52990200
C	2.56773900	7.43622200	0.73585900
H	3.35063500	8.07287900	1.16359200
H	2.45472700	7.76768600	-0.30279900
C	3.08839200	6.02237900	0.71363300
O	2.56714300	5.07060900	1.24059200
C	-12.23275600	19.91694000	7.10796300
H	-13.16619200	19.98887600	7.67280400

H	-11.51375500	20.60608700	7.56230600
H	-12.42981000	20.27114700	6.09109100
C	4.80979300	4.62867800	-0.07448200
H	5.14105800	4.30122200	0.91258000
H	4.07724600	3.91675000	-0.45996400
C	6.00118800	4.73265800	-1.01518600
H	6.70894300	5.47904000	-0.64646900
C	5.55378700	5.05936700	-2.41676100
H	5.10946500	4.16248200	-2.84925300
H	4.82020800	5.86672300	-2.39290000
O	4.23797000	5.93927400	0.01575000
C	7.40777300	3.09148300	-0.02267200
C	7.74215300	1.62973700	-0.10644800
H	6.79259600	1.09975700	0.03047500
H	8.06199400	1.40488800	-1.12955200
C	8.75058500	1.15994600	0.92957700
H	8.41233100	1.47713000	1.92122500
H	9.71410000	1.65557700	0.76544200
C	8.92237200	-0.35645200	0.89414300
H	7.94268800	-0.83438700	1.02417600
H	9.27973700	-0.65958700	-0.09990100
C	9.88810000	-0.87703300	1.95368000
H	9.53130000	-0.57462400	2.94689300
H	10.86723200	-0.39706300	1.82415000
C	10.05778100	-2.39287100	1.91800600
H	10.41800400	-2.69486400	0.92557400

H	9.07656700	-2.86951400	2.04108900
C	11.01370900	-2.92047900	2.98369600
H	11.99489200	-2.44285700	2.86095000
H	10.65290300	-2.61902000	3.97599400
C	11.18221200	-4.43653600	2.94743600
H	11.54428200	-4.73801700	1.95558200
H	10.20063800	-4.91350000	3.06832400
C	12.13529900	-4.96595900	4.01488100
H	13.11707200	-4.48912800	3.89413100
H	11.77301800	-4.66451300	5.00667500
C	12.30282800	-6.48213000	3.97845200
H	12.66586500	-6.78356200	2.98694500
H	11.32085800	-6.95868000	4.09818700
C	13.25443200	-7.01237100	5.04683400
H	14.23655400	-6.53603000	4.92717000
H	12.89130100	-6.71091700	6.03831600
C	13.42127100	-8.52861700	5.01028600
H	12.43901300	-9.00479400	5.12926900
H	13.78492300	-8.83003900	4.01900400
C	14.37191700	-9.05936400	6.07927200
H	15.35426600	-8.58333000	5.96032900
H	14.00820500	-8.75792600	7.07054300
C	14.53830000	-10.57566100	6.04259800
H	13.55585500	-11.05157000	6.16109300
H	14.90232500	-10.87703500	5.05144400
C	15.48837100	-11.10678500	7.11195100

H	15.12422500	-10.80529700	8.10307000
H	16.47082300	-10.63082300	6.99340600
C	15.65463600	-12.62301900	7.07533100
H	14.67234500	-13.09960600	7.19379700
H	16.01913500	-12.92514400	6.08441300
C	16.60459200	-13.15523900	8.14489000
H	16.23918600	-12.85282700	9.13416100
H	17.58539200	-12.67842500	8.02529500
C	16.76254000	-14.67162400	8.09812400
H	17.44657600	-15.03025100	8.87210500
H	15.79983000	-15.17133000	8.24587000
H	17.15550400	-14.99566500	7.12915000
O	6.62869300	3.44723000	-1.06771900
O	7.72185500	3.85054300	0.86197000
O	6.69222500	5.49837100	-3.18285100
O	7.55322500	6.67963600	-5.19114400
O	5.16977200	5.89120600	-5.15095400
H	4.78685600	5.25752400	-5.81560300
O	7.05224000	4.15913300	-5.39329100
C	8.98733200	6.52821900	-5.23586100
H	9.24649800	5.56531000	-5.68160100
H	9.33901600	7.32577000	-5.89393200
C	9.60036800	6.66418500	-3.85037600
H	9.29504000	5.81031500	-3.23963800
H	9.18303600	7.56123200	-3.36879200
N	11.05068800	6.65234900	-3.98810600

H	11.38907100	7.55908100	-4.29371900
H	11.49393200	6.45612500	-3.09835900
P	6.64016000	5.45411100	-4.77306700

AmB-DSPE8

0 1

C	6.75694100	-5.37116800	-0.22825900
H	7.36381900	-4.67942400	0.37341300
C	7.11355300	-5.10016000	-1.69012900
H	6.54794000	-5.79753200	-2.32221600
C	6.72764500	-3.67221700	-2.07576500
H	7.26725100	-2.97011300	-1.42116200
C	4.96078600	-3.64807000	-0.52386400
H	5.49588100	-2.88416400	0.06426300
C	5.28927000	-5.03913500	0.01783400
H	5.08901700	-5.05081800	1.09887800
C	7.01426200	-3.33663600	-3.52233400
H	6.47321700	-4.01840600	-4.18511500
H	8.08559300	-3.41042900	-3.72506800
H	6.69570500	-2.31568500	-3.74019500
C	2.94364800	-2.46763300	-1.19183000
H	3.00098400	-2.75574900	-2.24653200
C	1.51668000	-2.45886300	-0.73927900
H	1.33896000	-2.21002800	0.30379500

C	0.48047300	-2.71906900	-1.54262600
H	0.65695200	-2.96304300	-2.59013300
C	-0.89630700	-2.70098700	-1.08803300
H	-1.05758700	-2.45505600	-0.04115000
C	-1.96448400	-2.97229200	-1.86194200
H	-1.81658200	-3.22082200	-2.91272300
C	-3.31911700	-2.95202300	-1.36210900
H	-3.43472000	-2.68733700	-0.31304700
C	-4.41881000	-3.21441600	-2.09635200
H	-4.31437900	-3.47597000	-3.14897800
C	-5.75604000	-3.16432400	-1.55762100
H	-5.85767100	-2.87787200	-0.51083100
C	-6.87397500	-3.43155500	-2.26281700
H	-6.78406600	-3.70915500	-3.31258300
C	-8.20385100	-3.39027400	-1.70805500
H	-8.29541300	-3.11604300	-0.65741800
C	-9.32275600	-3.66567700	-2.40788000
H	-9.23592500	-3.92263600	-3.46315600
C	-10.65058600	-3.64944700	-1.84414400
H	-10.72989600	-3.44336500	-0.77811400
C	-11.77948500	-3.85409800	-2.54724200
H	-11.71491800	-4.03762300	-3.61924300
C	3.64641500	-1.11741100	-1.03340400
H	4.66969500	-1.21576000	-1.39600800
H	3.14626500	-0.39003200	-1.67916900
C	-13.10286300	-3.80041300	-1.95840400

H	-13.13571800	-3.71027800	-0.87441200
C	-14.24656100	-3.79591500	-2.65182900
H	-14.20682900	-3.86986900	-3.73995200
C	-15.61496400	-3.64211900	-2.05287900
H	-15.53661200	-3.70954900	-0.96180600
C	-16.21004000	-2.25890400	-2.38191100
H	-16.42755300	-2.24467400	-3.46294800
C	-15.27229200	-1.06769100	-2.10924100
H	-14.38188300	-1.24426400	-2.72058100
C	-14.76894200	-1.01647100	-0.66318900
H	-14.44575200	-2.00503800	-0.35014800
C	-12.46827400	-0.61833900	-0.11336700
C	-11.31580600	0.34494300	-0.24049200
H	-11.48437700	1.00238500	-1.09527000
H	-11.31092700	0.96538700	0.66239900
C	-9.96913400	-0.37350700	-0.36039100
H	-9.96845800	-0.98655000	-1.27284700
C	-8.81935700	0.62204900	-0.42299800
H	-8.96017500	1.25949100	-1.30238500
H	-8.85251000	1.26615200	0.46286700
C	-7.44080400	-0.02859200	-0.50293800
H	-7.42237500	-0.74103700	-1.33941100
C	-6.34263800	1.00514900	-0.71706900
H	-6.43979100	1.78783800	0.04620700
H	-6.52896100	1.48483500	-1.68274000
C	-4.92236500	0.41492800	-0.69227100

H	-4.94710000	-0.65369700	-0.92188600
H	-4.31681500	0.87731100	-1.47815300
C	-4.17513600	0.63692200	0.61886800
H	-4.05819200	1.71988500	0.76433300
C	-2.76066500	-0.01102100	0.60002100
H	-2.47800400	-0.25336400	-0.43101700
C	-1.70060100	0.89620000	1.20954500
H	-1.97764800	1.12502600	2.24515400
H	-1.68758800	1.84165800	0.65540000
C	-0.30251000	0.29081100	1.18853600
H	-0.04164300	0.01102500	0.15916800
C	0.73785200	1.28194500	1.69623600
H	0.45331300	1.64685600	2.68776300
H	0.76261100	2.13679700	1.01577700
C	2.14202300	0.69402700	1.79538400
C	3.21172000	1.74383500	2.08747400
H	3.04528400	2.18589700	3.07221000
H	3.15887100	2.53706400	1.33640300
C	4.57969100	1.09306800	2.04555300
H	4.62389100	0.34187000	2.83911600
C	4.79034200	0.39881900	0.69500700
H	4.82673900	1.15813300	-0.09248900
C	3.62105900	-0.56618100	0.38540600
H	3.64557600	-1.38550700	1.11534000
C	6.09706100	-0.34725600	0.63930800
C	-15.71942900	-0.44587800	0.36824200

H	-15.28707400	-0.53582900	1.36612500
H	-16.65749800	-1.00150500	0.33135700
H	-15.92453300	0.60944800	0.17476800
C	-15.89808500	0.25062100	-2.56083100
H	-16.83286000	0.46070000	-2.03252700
H	-16.11263500	0.22962600	-3.63326300
H	-15.22023700	1.08548400	-2.36945500
C	-16.56059800	-4.74808600	-2.52982600
H	-16.64458700	-4.73969400	-3.62224500
H	-17.55672700	-4.60389000	-2.10928700
H	-16.18714300	-5.73022600	-2.23131000
O	4.50731200	-6.00649000	-0.65050300
H	3.62538500	-5.61611100	-0.73606500
O	8.51601900	-5.31102800	-1.81252300
H	8.72888400	-5.45842600	-2.73970500
O	5.32502100	-3.52499000	-1.87764400
O	3.57834000	-3.50178700	-0.40901600
O	2.22911000	-0.27781500	2.79696700
H	1.41505700	-0.81046300	2.73355800
O	2.39555600	0.13412500	0.51005500
O	6.71236000	-0.60589200	-0.37209900
O	-0.24648300	-0.88023300	2.00810300
H	-1.12531500	-1.30093800	1.97588700
O	-2.81448300	-1.24910400	1.30862900
H	-3.59791500	-1.13877400	1.88804100
O	-4.85419800	0.12435800	1.75620300

H	-5.72855100	-0.23458700	1.48836800
O	-7.14229800	-0.74128100	0.70114600
H	-7.96534600	-1.15643600	1.01705600
O	-9.74770200	-1.22211100	0.76494100
H	-10.57495600	-1.71389200	0.92214900
O	-12.38135300	-1.68402400	0.47988500
O	-13.57226100	-0.17398100	-0.69134300
O	-17.42724200	-2.16435400	-1.64876500
H	-17.96567500	-1.46354400	-2.03169000
O	5.56981700	2.09294000	2.25234400
H	6.37091500	1.64669900	2.55279700
N	7.03934700	-6.72637800	0.21147600
H	6.32273500	-7.34208900	-0.16324200
H	7.92955200	-7.01433100	-0.18206400
C	10.11257600	-14.68435400	-0.77460800
H	9.56184200	-14.51569000	-1.70838800
H	11.17209900	-14.74146700	-1.05370500
C	9.90219800	-13.49139100	0.15393100
H	10.45217300	-13.65924700	1.08955200
H	8.84189900	-13.43256800	0.43363700
C	10.33797600	-12.16281900	-0.45673600
H	9.78860100	-11.99633300	-1.39295800
H	11.39861500	-12.22175500	-0.73528800
C	10.12531900	-10.97063500	0.47145800
H	9.06460700	-10.91002600	0.74842000
H	10.67265600	-11.13763400	1.40855300

C	10.56292400	-9.64198400	-0.13760900
H	10.01938400	-9.47835800	-1.07814700
H	11.62538400	-9.69848000	-0.40982900
C	10.33833200	-8.44927400	0.78673400
H	9.27539400	-8.38976300	1.05513900
H	10.87584500	-8.61306900	1.73001500
C	10.77763500	-7.11976300	0.17849500
H	10.27748600	-6.97370500	-0.78769600
H	11.85470700	-7.15107000	-0.03420200
C	10.45784300	-5.92403400	1.06929700
H	9.37543800	-5.89990400	1.24499300
H	10.91802100	-6.06381200	2.05658300
C	10.90342000	-4.58620400	0.48306000
H	10.59750400	-4.53684000	-0.56786500
H	11.99874700	-4.51361300	0.50418900
C	10.28209800	-3.40620000	1.22010500
H	9.19410000	-3.45736400	1.07265200
H	10.43987600	-3.51303300	2.30146000
C	10.77219300	-2.02818200	0.77507900
H	10.88226800	-2.00564300	-0.31621200
H	11.76719200	-1.82952100	1.19180900
C	9.78479700	-0.94606100	1.19966900
H	9.54129800	-1.08924900	2.26398000
H	8.86864500	-1.10848100	0.61909700
C	10.21353500	0.50225200	0.98877500
H	10.45667000	0.66147700	-0.06896700

H	11.13103700	0.70873100	1.55419400
C	9.11273100	1.47520500	1.40838000
H	8.87668800	1.30951100	2.46986100
H	8.20382100	1.24163600	0.84373500
C	9.44716800	2.94461700	1.18552400
H	9.63131700	3.11969800	0.12230000
H	10.37375900	3.20451500	1.71127200
C	8.30963000	3.84922000	1.65664400
H	8.14563700	3.76673000	2.73419300
H	7.36587600	3.55603200	1.17841300
C	8.46477700	5.31089900	1.35294700
O	8.02088900	6.21298300	2.02219900
C	9.67422400	-16.00629600	-0.15256300
H	9.83351800	-16.84606000	-0.83454400
H	10.23260200	-16.21188600	0.76629300
H	8.61107200	-15.98425300	0.10729100
C	9.15913900	6.84315400	-0.31771700
H	9.17992300	7.55918100	0.50714000
H	10.08351700	6.91472500	-0.89598400
C	7.96425100	7.11775200	-1.21553900
H	7.77598600	6.25604500	-1.85930500
C	8.17897200	8.37044700	-2.05119900
H	8.39266200	9.22972600	-1.40777000
H	9.02520800	8.21770700	-2.72366700
O	9.12055900	5.51200000	0.18524700
C	5.94414500	6.30407500	-0.18801200

C	4.73156600	6.77267400	0.55426400
H	5.07790700	7.30592200	1.44694600
H	4.24572800	7.50700000	-0.09955600
C	3.76901900	5.64884000	0.91313400
H	4.23995300	4.96657100	1.62964500
H	3.57132100	5.05571300	0.01398300
C	2.45687200	6.20124700	1.46416900
H	2.65474500	6.81277800	2.35428900
H	2.02088300	6.88105700	0.72024600
C	1.43720400	5.12083100	1.80739100
H	1.81222500	4.50779100	2.63672300
H	1.33804300	4.44086500	0.95095800
C	0.05746000	5.67670800	2.15398100
H	-0.32807500	6.23186100	1.28860200
H	0.14327700	6.40725100	2.96865900
C	-0.93955500	4.58847100	2.54067000
H	-0.88553800	3.78061100	1.80115800
H	-0.63750400	4.13711900	3.49512400
C	-2.38904700	5.06010600	2.62952400
H	-2.69886300	5.43722400	1.64567500
H	-2.46981600	5.90995500	3.31918300
C	-3.33332000	3.94159700	3.06473300
H	-3.08919000	3.03542500	2.49919100
H	-3.14551100	3.69273400	4.11719200
C	-4.81596900	4.23905800	2.86390600
H	-4.99272300	4.46135300	1.80223300

H	-5.09806700	5.14648800	3.41388600
C	-5.70169300	3.07018000	3.28893200
H	-5.34844600	2.14895400	2.81185300
H	-5.58976400	2.90723300	4.36905300
C	-7.18011400	3.26118100	2.96557000
H	-7.56997100	4.13094400	3.51118400
H	-7.29448700	3.50055700	1.89854500
C	-8.02108600	2.03015300	3.29633500
H	-7.71455400	1.19611700	2.65117900
H	-7.80388600	1.70988800	4.32356400
C	-9.52168300	2.27006600	3.16333200
H	-9.81500400	3.08326700	3.84031100
H	-9.74995100	2.63378400	2.15074500
C	-10.36574900	1.03392000	3.46056400
H	-10.06629600	0.61702700	4.43079800
H	-10.14893700	0.25578100	2.71841200
C	-11.86340300	1.32585600	3.49067800
H	-12.07359400	2.04474000	4.29352200
H	-12.16449300	1.83145600	2.56136000
C	-12.72687100	0.08237100	3.68516000
H	-12.38177200	-0.46019300	4.57364500
H	-12.58115600	-0.59957000	2.83882500
C	-14.20775100	0.41618600	3.83441400
H	-14.81987500	-0.48568400	3.92455600
H	-14.38451900	1.02897200	4.72388100
H	-14.57161500	0.98366900	2.97162900

O	6.79103600	7.35410300	-0.42168400
O	6.18608300	5.18508100	-0.55670600
O	7.05558900	8.64191200	-2.87900600
O	5.61273100	10.57563600	-3.28112700
O	6.11351000	9.98614600	-0.91712000
H	6.17207500	9.28855100	-0.24269600
O	4.52608700	8.43705600	-2.27922000
C	4.38008700	11.31793800	-3.32525100
H	3.53105600	10.63312600	-3.37628000
H	4.29583400	11.91813900	-2.41155400
C	4.43140700	12.20667200	-4.55188900
H	4.45166900	11.56646300	-5.43832800
H	5.37830400	12.76810700	-4.53945600
N	3.22888800	13.02845100	-4.58932900
H	3.29138400	13.78176900	-3.91171600
H	3.11965900	13.46038300	-5.49955200
P	5.70885100	9.31447100	-2.31984100
O	6.52261600	-0.75133800	1.85278200
H	7.36694500	-1.21470800	1.72693800

AmB-DSPE9

0 1

C	-1.19775900	11.21862100	-5.49020100
H	-0.21358400	11.39410400	-5.00759800
C	-2.26708200	12.23170100	-4.97607600

H	-3.24084300	12.15648900	-5.51910300
C	-2.49083700	11.97788800	-3.44514200
H	-1.52099200	11.99561100	-2.88905900
C	-2.10777400	9.62038300	-3.71736800
H	-1.21051800	9.66642900	-3.03384500
C	-1.70221800	9.80124900	-5.22531900
H	-1.03484300	8.95539800	-5.54643800
C	-3.50600100	12.93322500	-2.86089700
H	-4.49950100	12.84921500	-3.31433800
H	-3.15832200	13.96943000	-2.98248800
H	-3.64016600	12.75442100	-1.78499700
C	-3.30864200	8.10900800	-2.21327300
H	-4.16481200	8.81082300	-2.08294400
C	-3.75441900	6.66905600	-2.32817700
H	-2.95093700	5.96374100	-2.58507000
C	-5.01149300	6.27190500	-2.08277800
H	-5.78616600	6.98840600	-1.79879100
C	-5.45638300	4.87666300	-2.16640800
H	-4.67717600	4.15583400	-2.44971400
C	-6.71408700	4.45500400	-1.92502400
H	-7.51329700	5.14406000	-1.63731000
C	-7.09776200	3.04612000	-2.03949100
H	-6.28422000	2.37699700	-2.35019800
C	-8.33263100	2.56047300	-1.80408100
H	-9.15594700	3.21795400	-1.50883800
C	-8.66372400	1.14010800	-1.93296000

H	-7.84532600	0.49830300	-2.28355000
C	-9.87307700	0.61409600	-1.65539500
H	-10.70277800	1.24377900	-1.32184400
C	-10.16741900	-0.81297500	-1.78660300
H	-9.34570600	-1.42914900	-2.17183800
C	-11.35454900	-1.36623700	-1.47198700
H	-12.18546900	-0.75586200	-1.10573200
C	-11.62857900	-2.79691800	-1.60009600
H	-10.81989000	-3.39854900	-2.03244700
C	-12.78790600	-3.37522000	-1.23588200
H	-13.62123500	-2.78970300	-0.83595800
C	-2.25076000	8.33544000	-1.13288700
H	-1.99761000	9.42955700	-1.03503400
H	-2.67286300	8.09275000	-0.13320400
C	-13.01865700	-4.81791900	-1.34468200
H	-12.20828900	-5.38494300	-1.82151600
C	-14.11806400	-5.43267700	-0.89512000
H	-14.95558500	-4.89476200	-0.44818400
C	-14.32254600	-6.92497900	-0.94776500
H	-13.47757700	-7.42703900	-1.48779100
C	-14.36867900	-7.49753700	0.49452000
H	-15.33402600	-7.25174300	0.98953500
C	-13.19225600	-7.04697000	1.39767300
H	-13.23459800	-5.92373200	1.48398700
C	-11.82509000	-7.36909400	0.75397300
H	-11.81996500	-7.09438600	-0.33267400

C	-9.99418200	-5.76874800	0.66529600
C	-9.20488300	-4.82770700	1.54949000
H	-9.70513400	-4.56374200	2.49571800
H	-8.26908800	-5.35717600	1.85391000
C	-8.79925400	-3.58770900	0.73065700
H	-9.67345400	-3.03302700	0.31125600
C	-7.89471100	-2.63607300	1.53155700
H	-8.38207700	-2.34528400	2.48402400
H	-6.95237700	-3.14894600	1.83402500
C	-7.50011800	-1.35790900	0.75507800
H	-8.37834300	-0.81974300	0.32731400
C	-6.66177900	-0.38204200	1.62789000
H	-5.85157300	-0.92166900	2.16468500
H	-7.32320000	0.00235200	2.43667100
C	-6.06191100	0.84472300	0.90257900
H	-6.57614500	0.98061800	-0.07201300
H	-6.29737400	1.76049000	1.49521800
C	-4.53329200	0.74768700	0.68797900
H	-3.98633100	0.70641800	1.66179500
C	-3.97720500	1.98271000	-0.13386500
H	-4.69549400	2.84096100	-0.05053000
C	-2.54449000	2.37943100	0.23168500
H	-1.89504100	1.49878800	0.01437600
H	-2.46858600	2.53897900	1.32921300
C	-2.05727900	3.64030100	-0.52754300
H	-2.81059100	4.46576600	-0.47868500

C	-0.67514800	4.06482400	-0.02751600
H	0.06129000	3.24503900	-0.16920900
H	-0.71144900	4.27146500	1.06526300
C	-0.16803100	5.30218400	-0.77895900
C	1.12878200	5.83891900	-0.18517300
H	1.97571500	5.14251600	-0.31629100
H	1.04072300	5.94849400	0.92212000
C	1.45900000	7.19824600	-0.79733400
H	1.71268500	7.10957300	-1.88168000
C	0.28453800	8.18063600	-0.62569700
H	0.09096100	8.35817100	0.45797600
C	-0.94710500	7.54688200	-1.30294700
H	-0.72498300	7.34683100	-2.37983200
C	0.57329900	9.56830900	-1.27317400
C	-11.24602600	-8.75280900	0.95331500
H	-10.26462000	-8.84702300	0.46210400
H	-11.91089400	-9.51609300	0.51457300
H	-11.08893600	-9.01056900	2.00622900
C	-13.34867500	-7.60830300	2.80947000
H	-13.30020200	-8.70204100	2.83445700
H	-14.29994500	-7.30660700	3.26185800
H	-12.54331400	-7.23603400	3.45778600
C	-15.61888500	-7.27953700	-1.68182900
H	-16.50408200	-6.84412100	-1.20600100
H	-15.75384800	-8.37213600	-1.69293100
H	-15.59586800	-6.93329100	-2.72148800

O	-2.79818400	9.81585200	-6.19886400
H	-3.30862500	9.00709000	-5.93696600
O	-1.76849300	13.56191000	-5.07157100
H	-1.45672700	13.77862300	-5.96766800
O	-3.05599500	10.65596400	-3.36418400
O	-2.81752000	8.41497000	-3.59261500
O	0.01793900	5.02992200	-2.17854700
H	-0.57263200	4.25823100	-2.40274300
O	-1.23030700	6.26357700	-0.66839600
O	-0.08280000	10.57972200	-0.91241400
O	-1.91522900	3.43853200	-1.94863300
H	-2.46493000	2.71672400	-2.23990200
O	-3.91133700	1.72169300	-1.55357300
H	-4.18389500	0.83763800	-1.69964800
O	-4.11591800	-0.52306000	0.22921900
H	-4.98256900	-1.01219200	-0.18866300
O	-6.76478300	-1.78084400	-0.40021200
H	-6.98591000	-2.72020400	-0.66815200
O	-8.04996100	-4.18969600	-0.35082300
H	-8.63076400	-4.73685300	-0.95491200
O	-9.86329700	-5.98047600	-0.53698700
O	-10.92318100	-6.41711200	1.43076500
O	-14.32717200	-8.92502300	0.29066600
H	-14.61412700	-9.38712600	1.10125500
O	2.66529200	7.64002800	-0.12377200
H	2.89568800	8.47585000	-0.60820100

N	-1.08887300	11.35451000	-7.02146000
H	-1.95688300	10.96796800	-7.48861600
H	-1.00505700	12.34823800	-7.26198000
C	-10.02224400	3.31231600	8.58651500
H	-10.74813200	2.81855900	7.91129600
H	-9.96034400	2.64115500	9.46552400
C	-8.65092800	3.40810200	7.90336400
H	-7.91932300	3.89804900	8.57436500
H	-8.70399500	4.07505300	7.02171100
C	-8.14676200	2.02086500	7.49182000
H	-8.87444400	1.53128700	6.81619500
H	-8.09212400	1.35459400	8.37496000
C	-6.77429800	2.10928100	6.81493900
H	-6.81866200	2.76910300	5.92747900
H	-6.04197400	2.59885200	7.48599200
C	-6.28630300	0.71132400	6.42125600
H	-7.01720400	0.22427800	5.74802900
H	-6.24808200	0.05608900	7.31403500
C	-4.90926000	0.77509500	5.75474200
H	-4.93961000	1.42353500	4.85853800
H	-4.17868100	1.26608600	6.42727800
C	-4.42982300	-0.63211400	5.38457400
H	-5.15451900	-1.12124700	4.70630400
H	-4.40995300	-1.27762400	6.28481300
C	-3.04218100	-0.58300000	4.73918300
H	-3.05705700	0.05728900	3.83625800

H	-2.32012900	-0.08977300	5.41915400
C	-2.55753900	-1.99303400	4.38362300
H	-3.26988000	-2.48042600	3.68993400
H	-2.55287100	-2.63643700	5.28449700
C	-1.15941800	-1.93911600	3.75860100
H	-1.17473500	-1.30647000	2.84901200
H	-0.45389300	-1.43009400	4.44372900
C	-0.64786300	-3.34421400	3.41672200
H	-1.34134600	-3.84104100	2.71084900
H	-0.65482400	-3.98587500	4.31852600
C	0.76318600	-3.27012100	2.81969100
H	1.44160900	-2.74327500	3.51901500
H	0.75351500	-2.63722000	1.91030100
C	1.31564800	-4.66396300	2.49076500
H	0.64286100	-5.18227800	1.78098300
H	1.31351100	-5.29846300	3.39750800
C	2.73242800	-4.56069200	1.90910500
H	3.38909400	-4.02027100	2.61982500
H	2.72122800	-3.92312100	1.00323700
C	3.32563000	-5.93917600	1.58219800
H	2.67991500	-6.47749600	0.85901900
H	3.34128700	-6.57446300	2.48857400
C	4.74349300	-5.78598900	1.01478100
H	5.35947000	-5.15699600	1.69597200
H	4.71593000	-5.22906400	0.05131800
C	5.44563400	-7.10312800	0.82691100

O	6.18214800	-7.68817800	1.57735300
C	-10.53101800	4.69057100	9.00259100
H	-11.50874100	4.63094400	9.49283600
H	-9.84459900	5.18123200	9.70302900
H	-10.63686300	5.36107700	8.14153000
C	5.90185500	-8.78908100	-0.84796500
H	6.47999400	-9.21419300	0.00234200
H	5.10015300	-9.49627000	-1.15257500
C	6.81855900	-8.37975800	-2.01552700
H	6.37769900	-7.52996900	-2.59335800
C	7.14277900	-9.60679500	-2.90088300
H	7.68388300	-10.38446100	-2.33170200
H	6.20863100	-10.03424400	-3.33090600
O	5.14890700	-7.62429700	-0.42502900
C	8.34753700	-6.58280900	-1.45256600
C	9.81366100	-6.33087100	-1.23214400
H	10.36237500	-7.27420700	-1.03129600
H	10.24727100	-5.97745600	-2.19729600
C	10.02094700	-5.31066700	-0.10220300
H	9.98895300	-5.82948000	0.87640300
H	9.17029200	-4.59897700	-0.07582900
C	11.33376400	-4.53079900	-0.24729400
H	12.20057600	-5.21829800	-0.24644700
H	11.37093800	-4.02602200	-1.23333100
C	11.47780700	-3.49184500	0.87609300
H	11.45527900	-3.98746500	1.86596900

H	10.60146200	-2.81316000	0.88536300
C	12.76974600	-2.67995500	0.71949400
H	12.79833000	-2.19672500	-0.27705700
H	13.64860700	-3.35347400	0.73204100
C	12.91003600	-1.61696500	1.81782600
H	12.02959900	-0.94557400	1.82343700
H	12.90698000	-2.08855000	2.81895200
C	14.19574400	-0.80638600	1.61434900
H	14.20479500	-0.35171700	0.60429000
H	15.07727500	-1.47620400	1.62751300
C	14.34750000	0.28328300	2.68109200
H	13.46285400	0.94895400	2.68484900
H	14.36547400	-0.16272900	3.69362100
C	15.62370000	1.09439500	2.43200300
H	15.60795900	1.53047600	1.41396400
H	16.50892000	0.42941700	2.43726600
C	15.78723700	2.20135900	3.47769900
H	14.89765600	2.86042700	3.48614700
H	15.82461400	1.77043700	4.49643600
C	17.05289900	3.01706000	3.19559500
H	17.94261800	2.35797600	3.19301700
H	17.01656100	3.44073600	2.17302300
C	17.22337100	4.13601800	4.22735200
H	16.32899400	4.78842800	4.24009200
H	17.27542500	3.71452600	5.24954700
C	18.48007300	4.95734500	3.92216000

H	19.37220900	4.30173000	3.90664200
H	18.42476900	5.37597100	2.89837400
C	18.66133200	6.08116100	4.94826900
H	18.72799000	5.66051600	5.97003600
H	17.76291500	6.72785300	4.97069900
C	19.91063700	6.90790700	4.62441800
H	20.80353700	6.25401600	4.59809500
H	19.83943200	7.31922300	3.59901700
C	20.11020400	8.04096100	5.63953900
H	20.18388300	7.62530900	6.66328900
H	19.21199400	8.68815900	5.66758700
C	21.35296400	8.86359400	5.30445500
H	21.50659500	9.67703700	6.02140600
H	22.25956800	8.24717100	5.30943700
H	21.28198300	9.31533900	4.30777500
O	8.10669400	-7.95085600	-1.49064700
O	7.45310000	-5.78871200	-1.59439100
O	7.84438400	-9.17469900	-4.08939400
O	9.57049300	-10.03236500	-5.67616100
O	9.98897900	-10.43364200	-3.11551600
O	10.08377900	-7.97173300	-4.18800500
C	10.78401500	-10.74259400	-5.98084000
H	11.16276600	-11.34005300	-5.12150600
H	10.43280400	-11.43637100	-6.77827200
C	11.84068900	-9.77339500	-6.54226300
H	11.37436600	-9.13290600	-7.33092300

H	12.63704200	-10.36420000	-7.06179700
N	12.40174500	-8.87909600	-5.47482600
H	12.92330000	-9.42558500	-4.78378900
H	13.05142800	-8.21236500	-5.89213200
P	9.45719400	-9.47739600	-4.11014500
H	11.00387300	-7.92535000	-4.67513600
O	1.58385500	9.69241000	-2.27730300
H	2.24190900	10.33073500	-1.99251100

AmB-DSPE10

0 1

C	-1.08554300	-5.63978400	2.11048600
H	-0.94548900	-5.95252000	3.17105500
C	-0.17290300	-4.42588400	1.75676000
H	-0.20635400	-4.16671100	0.66899500
C	-0.63620800	-3.19652800	2.61228900
H	-0.66695400	-3.46144100	3.69723600
C	-2.92692300	-3.92760100	2.52934400
H	-2.95011000	-4.04858900	3.65050100
C	-2.54179600	-5.27038600	1.81007800
H	-3.30952500	-6.05575200	2.02686200
C	0.20533800	-1.97553100	2.33534300
H	0.15421300	-1.63787500	1.29680100
H	1.25465100	-2.18259600	2.58296100

H	-0.12251400	-1.13220500	2.95669500
C	-4.66000100	-2.19634200	2.56949200
H	-4.00136400	-1.43813500	2.08486100
C	-6.07742000	-2.14310600	2.05738300
H	-6.72088900	-2.95546100	2.41874600
C	-6.53512100	-1.15329600	1.28024100
H	-5.88343800	-0.33848400	0.95753900
C	-7.91883800	-1.07012100	0.79588600
H	-8.57688100	-1.88911500	1.11818800
C	-8.39928900	-0.08660300	0.01140000
H	-7.77708300	0.74583800	-0.32613300
C	-9.79161400	-0.06703500	-0.45110400
H	-10.39401200	-0.92316100	-0.11622800
C	-10.33430400	0.89017500	-1.22661300
H	-9.74439800	1.74093100	-1.57805400
C	-11.73466900	0.86058600	-1.66054300
H	-12.30359700	-0.02428200	-1.34750400
C	-12.32157300	1.82618200	-2.39235100
H	-11.76425800	2.70590200	-2.72453500
C	-13.72728200	1.76897700	-2.80173600
H	-14.26545000	0.85968900	-2.50762800
C	-14.34546400	2.74264800	-3.49424500
H	-13.81162400	3.64453700	-3.80614000
C	-15.75457400	2.67328000	-3.88671100
H	-16.27213400	1.74120300	-3.63285300
C	-16.40378600	3.66125400	-4.52759400

H	-15.89854800	4.58581000	-4.81926400
C	-4.54970100	-2.11735400	4.09194700
H	-3.47334400	-2.09175300	4.42095700
H	-4.93705800	-1.13617000	4.44127400
C	-17.82573900	3.57431700	-4.87648100
H	-18.30352300	2.61276500	-4.64791100
C	-18.52163400	4.57976900	-5.41836000
H	-18.06757200	5.53493100	-5.68373600
C	-19.99811500	4.51065900	-5.71332000
H	-20.40223000	3.48753900	-5.49686900
C	-20.76060200	5.51379100	-4.80567500
H	-20.60571900	6.55795400	-5.15599200
C	-20.40795000	5.41470500	-3.29985000
H	-19.30657300	5.63503400	-3.19342600
C	-20.61126800	3.98269900	-2.77028400
H	-20.18829200	3.22516100	-3.48269100
C	-18.96587200	2.85338200	-1.36884400
C	-18.13113600	3.06288900	-0.12898600
H	-18.00655900	4.11721700	0.16826100
H	-18.66843600	2.57665500	0.72434400
C	-16.78676800	2.33696300	-0.29889100
H	-16.19477900	2.69206600	-1.17939600
C	-15.92346300	2.40214100	0.97652800
H	-15.77340700	3.45572800	1.28824700
H	-16.44581500	1.91387000	1.83178600
C	-14.54291500	1.71805000	0.82749900

H	-13.97051100	2.07558600	-0.06282300
C	-13.67126300	1.88238500	2.10367500
H	-14.26008900	1.65625900	3.01718400
H	-13.41127100	2.95955200	2.19430900
C	-12.35884500	1.07485300	2.14652600
H	-12.09017500	0.74594200	1.12130000
H	-11.53271600	1.75849600	2.44357800
C	-12.40034400	-0.10358200	3.14202000
H	-12.49268600	0.23339300	4.19884100
C	-11.06688700	-0.94732800	3.04014900
H	-10.23116200	-0.30555100	2.64718200
C	-10.65213200	-1.61392600	4.36080300
H	-11.46375400	-2.32395900	4.63428500
H	-10.62327700	-0.86203300	5.17601400
C	-9.28377200	-2.33800000	4.29684300
H	-8.47945200	-1.69688700	3.85083100
C	-8.89117000	-2.88508400	5.67778600
H	-9.66293200	-3.58303700	6.06173600
H	-8.83141500	-2.04942100	6.41058200
C	-7.54882000	-3.64204000	5.62578800
C	-7.06584500	-4.02862200	7.01943900
H	-7.71665800	-4.78225600	7.49415800
H	-7.10579500	-3.15731600	7.71130900
C	-5.62920500	-4.54059000	6.94514500
H	-5.56654700	-5.50394900	6.38538500
C	-4.70753100	-3.49876900	6.27503600

H	-4.68354600	-2.56565500	6.88266100
C	-5.27506300	-3.21891700	4.86941300
H	-5.32443200	-4.16915600	4.28513100
C	-3.23342900	-4.01838000	6.14505300
C	-21.98845900	3.56879200	-2.30724900
H	-21.98806700	2.53492400	-1.93367100
H	-22.69905800	3.62072800	-3.14771600
H	-22.37619800	4.19391300	-1.49747900
C	-21.15471000	6.47709900	-2.49597500
H	-22.23846100	6.33432200	-2.52596000
H	-20.93958600	7.48595500	-2.86217600
H	-20.85188300	6.43642400	-1.44109800
C	-20.28527000	4.82568700	-7.18468500
H	-19.93845200	5.82254000	-7.47259200
H	-21.36957100	4.78343900	-7.36880400
H	-19.80105800	4.09864600	-7.84600000
O	-2.40649200	-5.23781000	0.36380600
H	-3.27655200	-4.76468300	0.09148800
O	1.16959800	-4.68862000	2.15268100
H	1.51127000	-5.51440700	1.78033300
O	-1.97256000	-2.90360800	2.15256200
O	-4.16000100	-3.50166800	2.01286400
O	-7.62445500	-4.81282800	4.78030000
H	-8.38421800	-4.63696200	4.17455200
O	-6.64477700	-2.71634700	5.00258000
O	-2.29023700	-3.20125200	5.98300000

O	-9.29551500	-3.49558200	3.41839000
H	-9.98305900	-3.41091900	2.76443700
O	-11.10433200	-2.02507800	2.07735600
H	-12.00884400	-2.06935000	1.72253600
O	-13.66624800	-0.78254800	3.12748700
H	-14.10315400	-0.63997500	2.19075400
O	-14.78798200	0.32878500	0.54879200
H	-15.69423900	0.18638100	0.13921600
O	-17.18944400	0.95192000	-0.50094300
H	-17.69798700	0.87209700	-1.42100900
O	-19.01025100	1.86373900	-2.10955100
O	-19.72084300	3.96153500	-1.56836600
O	-22.14721400	5.17492200	-5.01985800
H	-22.72016600	5.90409200	-4.71106500
O	-5.24869900	-4.82835800	8.31607500
H	-4.34772200	-5.23052000	8.21181100
N	-0.73957800	-6.81372900	1.15590200
H	-1.08242200	-6.58872400	0.17858700
H	0.27837500	-6.93283500	1.12590200
C	16.49283800	19.05592600	3.18104100
H	17.47038200	18.97466700	3.69744100
H	16.74836900	19.31326600	2.13352500
C	15.76445400	17.70505500	3.22497700
H	14.78739500	17.77827500	2.70815300
H	15.51146600	17.43863700	4.27001600
C	16.61821700	16.60241900	2.58915100

H	17.59322900	16.52227200	3.10986900
H	16.87438700	16.86902200	1.54418800
C	15.89216600	15.25245600	2.62330300
H	15.64269800	14.97262600	3.66581300
H	14.91440400	15.32506300	2.10738400
C	16.75874800	14.16883000	1.97305600
H	17.73282300	14.09276300	2.49619200
H	17.01554100	14.45724300	0.93405100
C	16.04576600	12.81322600	1.98377400
H	15.79861100	12.51516500	3.02182300
H	15.06754000	12.88782100	1.46871900
C	16.91987100	11.74615200	1.31577400
H	17.89249200	11.66419000	1.84043100
H	17.17890900	12.05513800	0.28337100
C	16.20796300	10.39006900	1.30097600
H	15.95608700	10.07790900	2.33396400
H	15.23152000	10.47559200	0.78364300
C	17.07780000	9.32660000	0.62077900
H	18.04838700	9.23119900	1.14651400
H	17.34067700	9.64681000	-0.40700900
C	16.35161200	7.97732800	0.59014700
H	16.10206400	7.65633400	1.62115300
H	15.37277200	8.08433900	0.08091500
C	17.19761800	6.90517000	-0.10865000
H	18.16709900	6.78231800	0.41318700
H	17.46461100	7.23533400	-1.13198800

C	16.44031400	5.57159900	-0.15559500
H	15.46065300	5.71332100	-0.65535100
H	16.18987900	5.24509200	0.87387700
C	17.24970300	4.48299300	-0.87486700
H	18.22329400	4.33528600	-0.36780700
H	17.50733100	4.81626600	-1.89959300
C	16.46276000	3.16588400	-0.91976600
H	15.48339100	3.33422600	-1.41197000
H	16.21039400	2.84421200	0.11059300
C	17.23571500	2.05621900	-1.64764300
H	18.21048300	1.87363900	-1.15160400
H	17.48421500	2.37565300	-2.67886200
C	16.40668500	0.76494700	-1.67683800
H	15.39069600	0.97306600	-2.08207700
H	16.24024600	0.38895800	-0.64208800
C	17.03190500	-0.31297000	-2.51296200
O	16.84414200	-0.57916400	-3.67882400
C	15.64816600	20.16205100	3.81050500
H	16.16352300	21.12853500	3.78059700
H	14.69051300	20.28326000	3.28939300
H	15.41726100	19.94457500	4.86050100
C	18.45711500	-2.26837600	-2.34780600
H	18.21870800	-2.30392400	-3.43760300
H	19.54969400	-2.14491500	-2.20115200
C	17.88257300	-3.48487300	-1.59946800
H	17.64458400	-3.22310300	-0.53453300

C	18.83562400	-4.69392100	-1.72202800
H	18.96037300	-5.00160700	-2.77989900
H	19.82685900	-4.45029700	-1.28056600
O	17.91270800	-1.05940400	-1.74251600
C	15.45384700	-3.57322700	-1.62856500
C	14.32961200	-4.37716200	-2.21020700
H	14.66904600	-4.97548000	-3.08201800
H	14.03859500	-5.14759000	-1.45779400
C	13.15117900	-3.46599500	-2.58166000
H	13.32250100	-3.02883200	-3.58640300
H	13.11325000	-2.59628600	-1.89047200
C	11.80420400	-4.20048200	-2.54738200
H	11.80634200	-5.05409400	-3.25280000
H	11.64113800	-4.65028300	-1.54715100
C	10.65020600	-3.24032200	-2.88076100
H	10.79675300	-2.79583700	-3.88554500
H	10.65844600	-2.37663100	-2.18479300
C	9.29483800	-3.95672300	-2.81236500
H	9.15724800	-4.41447700	-1.81194500
H	9.27733700	-4.80681700	-3.52348700
C	8.13150300	-3.00084600	-3.11227300
H	8.14929600	-2.13872200	-2.41605800
H	8.24567100	-2.55434800	-4.11978800
C	6.79288700	-3.74178600	-3.00703300
H	6.69277100	-4.20414700	-2.00449900
H	6.77118500	-4.59155800	-3.71833700

C	5.61004200	-2.80417600	-3.27280400
H	5.63512700	-1.94181700	-2.57734100
H	5.68893900	-2.35641900	-4.28306300
C	4.28831900	-3.56656200	-3.12914600
H	4.22023300	-4.02206400	-2.12144400
H	4.26257300	-4.42083900	-3.83471400
C	3.08997000	-2.64443300	-3.37361400
H	3.12180200	-1.77692700	-2.68466800
H	3.14342100	-2.20113000	-4.38789300
C	1.77910500	-3.41793600	-3.19804600
H	1.74747100	-4.27611300	-3.89873100
H	1.73351800	-3.86480500	-2.18605900
C	0.57063900	-2.50553500	-3.42776600
H	0.60829400	-1.63330700	-2.74508500
H	0.60715300	-2.06696300	-4.44524800
C	-0.73394400	-3.28395000	-3.22963000
H	-0.76816700	-4.15104200	-3.91894800
H	-0.77227500	-3.71725100	-2.21102100
C	-1.94950700	-2.37907100	-3.45996400
H	-1.92151400	-1.95017900	-4.48139600
H	-1.90848000	-1.50285900	-2.78208400
C	-3.24850700	-3.16316000	-3.24155400
H	-3.28359900	-4.03828800	-3.91995900
H	-3.26610100	-3.58807900	-2.21904000
C	-4.48069200	-2.27549000	-3.46346000
H	-4.46115400	-1.84744200	-4.48574000

H	-4.44141600	-1.39640200	-2.78898900
C	-5.77020300	-3.06178700	-3.23687500
H	-6.65624400	-2.43376600	-3.38986400
H	-5.84985100	-3.91488300	-3.92107200
H	-5.82924800	-3.45434100	-2.21378100
O	16.64690900	-3.91720000	-2.25226500
O	15.41776100	-2.74481900	-0.74698400
O	18.37204000	-5.76013600	-0.86353400
O	18.38201000	-8.25108100	-0.79622800
O	17.80033500	-7.12171100	-3.08683500
O	16.14673000	-6.96145900	-0.99222300
C	18.27173700	-9.55101100	-1.40884600
H	18.28625200	-9.50360200	-2.52243900
H	19.20826100	-10.04795400	-1.05877700
C	17.03436000	-10.29908000	-0.86596100
H	17.02654600	-10.21792900	0.25367200
H	17.13793400	-11.39207800	-1.10069300
N	15.75538800	-9.71348100	-1.39378500
H	15.69201600	-9.85196500	-2.41268900
H	14.95894500	-10.19440000	-0.95929100
P	17.64468800	-7.01572100	-1.62328500
H	15.68021700	-7.90221400	-0.94490000
O	-2.96121200	-5.42069600	6.21069000
H	-2.34798900	-5.59484100	6.92848600

AmB-DSPEc

AmB-DSPEc1

0 1

C	8.60280900	-2.73830400	1.10801900
H	9.33703300	-1.93782300	1.25877100
C	8.95540000	-3.42848700	-0.21053600
H	8.24009500	-4.25177800	-0.36924300
C	8.81549900	-2.45972800	-1.37819000
H	9.53328100	-1.63801600	-1.22847300
C	7.16300200	-1.20452400	-0.20831200
H	7.88136000	-0.38251200	-0.08578700
C	7.21056500	-2.11796300	1.01577000
H	7.00669800	-1.50832600	1.90674200
C	9.05293200	-3.11837500	-2.71755400
H	8.32003200	-3.91567300	-2.87542700
H	10.05149400	-3.55728000	-2.73703700
H	8.95709200	-2.39235600	-3.52771300
C	5.50325400	-0.02459200	-1.52815200
H	5.73823200	-0.66542600	-2.38427800
C	4.02318800	0.15667700	-1.42897700
H	3.67362200	0.80816700	-0.63471100
C	3.13266100	-0.49557300	-2.18300800
H	3.46924800	-1.18243100	-2.95823000
C	1.70889800	-0.38054400	-1.94553800

H	1.40950900	0.38733600	-1.23991700
C	0.74237000	-1.19018900	-2.41894200
H	0.97391800	-2.01570400	-3.08989900
C	-0.61087400	-1.01939600	-1.94273800
H	-0.75345400	-0.14458200	-1.31084600
C	-1.67188900	-1.82618100	-2.14165800
H	-1.59233300	-2.71497100	-2.76588600
C	-2.93341900	-1.54757200	-1.49683900
H	-2.96067000	-0.65837500	-0.86517300
C	-4.05636700	-2.28697900	-1.60153500
H	-4.06268700	-3.17094600	-2.23810500
C	-5.26755000	-1.97296000	-0.88489500
H	-5.23186200	-1.10968700	-0.21984500
C	-6.42071000	-2.66536900	-0.97586700
H	-6.47978100	-3.51771600	-1.65173700
C	-7.60012800	-2.34143700	-0.21030900
H	-7.50299300	-1.53542700	0.51520800
C	-8.79670900	-2.94370600	-0.33661900
H	-8.92716700	-3.73329400	-1.07571500
C	6.30340800	1.26961600	-1.68705900
H	7.36550000	1.01018600	-1.67287200
H	6.09164400	1.68069300	-2.67927400
C	-9.95334600	-2.55699700	0.44789300
H	-9.75815600	-1.85106300	1.25256300
C	-11.20985700	-2.95369200	0.21929000
H	-11.40856300	-3.64752200	-0.59921200

C	-12.41027000	-2.47345500	0.98510900
H	-12.07035600	-1.91325900	1.86352400
C	-13.26471700	-1.51173700	0.13638300
H	-13.71993500	-2.10643700	-0.67306200
C	-12.48050400	-0.37405200	-0.54456900
H	-11.73186400	-0.86470500	-1.17416700
C	-11.68047200	0.48440300	0.44090800
H	-11.14581500	-0.15566900	1.13683500
C	-9.40485000	1.12505400	0.03003600
C	-8.48828400	1.89073500	-0.89045300
H	-8.94155300	1.95746600	-1.88126100
H	-8.40713000	2.90875300	-0.49170500
C	-7.08821200	1.27690000	-0.96422300
H	-7.16243300	0.25611600	-1.36625500
C	-6.17606400	2.10746900	-1.85617500
H	-6.60332800	2.13778300	-2.86433600
H	-6.14993100	3.13559300	-1.47555700
C	-4.74090500	1.59460900	-1.94581700
H	-4.74460300	0.54210600	-2.26208700
C	-3.92445800	2.41116000	-2.94687400
H	-4.12566800	3.47599300	-2.78117700
H	-4.29846400	2.17083700	-3.94675800
C	-2.41148400	2.15115800	-2.86126700
H	-2.22620100	1.18984200	-2.37773100
H	-1.98120800	2.07827800	-3.86471000
C	-1.64093900	3.24256700	-2.11245000

H	-1.58544500	4.13617500	-2.74992200
C	-0.21479000	2.78729200	-1.76696700
H	0.21497900	2.23458200	-2.61146500
C	0.69913600	3.95538200	-1.41109000
H	0.20897300	4.59035700	-0.66548000
H	0.85262700	4.56208300	-2.31178000
C	2.04689800	3.50484400	-0.85189600
H	2.41221400	2.64291900	-1.42193500
C	3.10394300	4.60244200	-0.93718300
H	2.76331700	5.48299700	-0.38286200
H	3.23611800	4.89077600	-1.98352100
C	4.45915500	4.18283200	-0.36788000
C	5.53694500	5.23748900	-0.55609600
H	5.25705000	6.16208400	-0.04709700
H	5.65912000	5.44899600	-1.62211200
C	6.85945800	4.74746800	0.00018500
H	6.76508400	4.64102800	1.08890700
C	7.21133100	3.36319900	-0.60920700
H	7.56586000	3.57693400	-1.62517200
C	6.02603300	2.37803200	-0.67217200
H	5.86615000	1.95790200	0.32477500
C	8.35108700	2.74376400	0.15899600
C	-12.45108200	1.53658900	1.21194300
H	-11.78920300	2.03933400	1.92087700
H	-13.26360400	1.05514500	1.75789700
H	-12.87154100	2.28895800	0.54116300

C	-13.38423700	0.46057000	-1.45052400
H	-14.20040500	0.92872800	-0.89211400
H	-13.82441200	-0.16094000	-2.23569400
H	-12.81697200	1.26020600	-1.93203600
C	-13.26561100	-3.64618700	1.47265400
H	-13.60503000	-4.25407900	0.62650300
H	-14.14466200	-3.28101800	2.00527900
H	-12.68929700	-4.29282900	2.13820900
O	6.29588600	-3.18470100	0.90577600
H	5.38211200	-2.84813500	0.89438800
O	10.27903200	-3.90282700	-0.16506100
H	10.36679700	-4.26936900	0.73043800
O	7.48815500	-1.93031100	-1.38706800
O	5.85930200	-0.73699600	-0.32530400
O	4.37917500	3.91263700	1.00016700
H	3.48228500	3.54134900	1.14734800
O	4.84153800	3.02433100	-1.11270400
O	8.34486200	1.67904000	0.73199300
O	1.92143300	3.14036100	0.52809100
H	1.71542200	2.19052600	0.60303500
O	-0.28782800	1.85490100	-0.69912000
H	-0.90856300	2.24316900	-0.06401600
O	-2.26154900	3.63058300	-0.89608200
H	-2.95921900	2.98169600	-0.67544500
O	-4.09215700	1.67324400	-0.67162100
H	-4.75224900	1.45307000	0.01172800

O	-6.49594900	1.22873500	0.33149300
H	-7.16054600	0.85692500	0.93862400
O	-9.01383800	0.55007200	1.03375500
O	-10.66948400	1.17126900	-0.36221800
O	-14.28529400	-1.01789200	0.99848000
H	-14.99452600	-0.65134300	0.45973200
O	7.82653000	5.71943200	-0.33602500
H	8.68023800	5.41558900	-0.00315700
N	8.77133100	-3.71748400	2.17507300
H	7.93640700	-4.29903900	2.20267000
H	8.84128700	-3.25928500	3.07781700
C	-4.19886900	4.16053300	1.92422100
H	-4.48457600	3.63475600	1.00884000
H	-4.82056700	3.76750200	2.73565800
C	-2.71471200	3.97132100	2.22507100
H	-2.44968900	4.51637900	3.13931900
H	-2.12339500	4.40139500	1.41120200
C	-2.34332900	2.50028800	2.38734100
H	-2.58976500	1.95180400	1.47234300
H	-2.95846900	2.05240100	3.17787600
C	-0.86541400	2.29414000	2.72653500
H	-0.58831200	2.83619200	3.63340300
H	-0.21883400	2.67971200	1.92799200
C	-0.48106900	0.85646000	2.95356500
O	0.27746700	0.46307400	3.80621000
C	-0.83409100	-1.36336600	2.18349300

H	-0.62465800	-1.62401300	3.22303000
H	-1.75599900	-1.84786500	1.85336200
C	0.31496700	-1.80478700	1.29450400
H	0.29977600	-1.23573300	0.36763700
C	0.23699000	-3.29040000	0.98873300
H	0.34494200	-3.87693100	1.90608300
H	-0.72050700	-3.51229200	0.51348200
O	-1.09495400	0.03725100	2.07117400
C	2.23517400	-0.46393600	1.72764500
C	3.61870600	-0.47767700	2.29755900
H	3.66036200	-1.20608200	3.11044600
H	4.24502200	-0.86398500	1.48634500
C	4.10990800	0.90304000	2.71263700
H	3.53207500	1.25837900	3.57374800
H	3.91747400	1.60555400	1.89900500
C	5.60314700	0.91278300	3.02887300
H	5.82374200	0.16133900	3.79856600
H	6.15356700	0.60917700	2.13129500
C	6.09792700	2.28336300	3.47966400
H	5.64266600	2.57245200	4.43227000
H	5.82865300	3.04450600	2.74224800
O	1.58126100	-1.60645400	1.94379400
O	1.74864600	0.45348200	1.09300600
O	1.24047000	-3.66799700	0.04933800
O	3.05807900	-5.14688400	-0.57065700
O	2.81947200	-4.47097600	1.95662900

O	3.65464800	-2.82267700	0.15598200
C	4.42937000	-5.52415300	-0.75689700
H	5.05097700	-5.20176200	0.08488900
H	4.45802200	-6.61517200	-0.80606900
C	4.94012500	-4.92738200	-2.05622000
H	4.24006300	-5.20275100	-2.84909800
H	5.91269800	-5.37698700	-2.29724700
N	5.01081000	-3.46182800	-1.97405500
H	5.91123500	-3.15062300	-1.60345300
H	4.91539600	-3.04367600	-2.89257000
P	2.71663300	-4.04331500	0.55295300
H	4.06508000	-2.92648400	-0.78624700
O	9.44872700	3.53697300	0.18742900
H	10.10565400	3.08045400	0.73798200
H	-4.44772000	5.21701700	1.79299600
H	7.18419100	2.29034800	3.60847700

AmB-DSPEc2

0 1

C	8.74089200	-3.16060500	0.50865600
H	9.64236300	-2.70717800	0.07807600
C	8.40046100	-4.38154800	-0.34522900
H	7.51010800	-4.86717900	0.08283900
C	8.05592100	-3.95838400	-1.76834900

H	8.94108300	-3.46028000	-2.19890700
C	7.24795400	-1.86305700	-1.00670200
H	8.08171400	-1.32882700	-1.48724800
C	7.60963900	-2.14037700	0.44929900
H	7.92767900	-1.18610300	0.89419900
C	7.64388100	-5.12259100	-2.64035100
H	6.75237700	-5.59887000	-2.22389600
H	8.45077500	-5.85611600	-2.67683300
H	7.41465500	-4.78096400	-3.65214100
C	5.61595300	-0.57921100	-2.22919200
H	5.72572000	-1.35824200	-2.99073300
C	4.17020100	-0.30355100	-1.98758500
H	3.96041200	0.34302100	-1.14438500
C	3.16255300	-0.85956100	-2.66669200
H	3.36745700	-1.53215600	-3.49909200
C	1.78204300	-0.64452100	-2.28584600
H	1.61399500	0.00106700	-1.42901900
C	0.70681500	-1.21551400	-2.86126300
H	0.82350100	-1.87114100	-3.72381000
C	-0.61927000	-1.00101100	-2.33439400
H	-0.67693500	-0.38021700	-1.44190900
C	-1.75615400	-1.52079200	-2.84128700
H	-1.72104800	-2.15116200	-3.72921900
C	-3.03832200	-1.28687800	-2.23030400
H	-3.03889500	-0.65081200	-1.34516100
C	-4.21277200	-1.80410300	-2.65298400

H	-4.23844100	-2.44370500	-3.53424800
C	-5.44752300	-1.57737700	-1.94658500
H	-5.38565600	-0.91199900	-1.08743700
C	-6.64619000	-2.12500100	-2.23580100
H	-6.74808000	-2.76952100	-3.10792100
C	-7.81511500	-1.91479800	-1.41571700
H	-7.66265900	-1.35584600	-0.49371900
C	-9.05872000	-2.34726700	-1.69252700
H	-9.25201100	-2.87987300	-2.62277200
C	6.44264500	0.64669800	-2.63595400
H	7.45440700	0.30756600	-2.87410900
H	6.02130000	1.07856400	-3.54903300
C	-10.18010000	-2.09984100	-0.80611400
H	-9.92135900	-1.70421200	0.17402500
C	-11.46888600	-2.27272400	-1.11724900
H	-11.73301500	-2.64853700	-2.10687600
C	-12.61886100	-1.94149800	-0.20711500
H	-12.24178400	-1.82703900	0.81549400
C	-13.27847800	-0.60622700	-0.60491200
H	-13.72306600	-0.74850100	-1.60395900
C	-12.31378300	0.58990000	-0.72130000
H	-11.55295700	0.29139400	-1.44856800
C	-11.54810600	0.88233600	0.57524400
H	-11.14062200	-0.04029100	0.98072100
C	-9.18661700	1.32982800	0.52095200
C	-8.12494000	2.30066700	0.07192600

H	-8.52498900	2.94512400	-0.71257100
H	-7.87631300	2.93197000	0.93286400
C	-6.85207600	1.58855700	-0.39795700
H	-7.10276100	0.94514500	-1.25525200
C	-5.79304400	2.59212000	-0.82262500
H	-6.18717200	3.17419800	-1.66302800
H	-5.59964300	3.28825000	0.00200800
C	-4.46193200	1.96821600	-1.23350900
H	-4.64695100	1.13127100	-1.92196200
C	-3.57721100	2.99154800	-1.94200500
H	-3.61482300	3.93404200	-1.38277800
H	-4.02260000	3.18835100	-2.92200600
C	-2.11491000	2.54352700	-2.09151600
H	-2.03301100	1.46010500	-1.97683800
H	-1.74870500	2.77514000	-3.09629800
C	-1.17567200	3.22634900	-1.09790000
H	-1.11172500	4.29074600	-1.36517300
C	0.24454200	2.62398700	-1.14259600
H	0.46191600	2.27123200	-2.15951700
C	1.30732800	3.64728800	-0.75004700
H	1.05186100	4.09521700	0.21640800
H	1.29156700	4.44920900	-1.49875000
C	2.71443100	3.06068000	-0.65181500
H	2.84632700	2.29138500	-1.42166000
C	3.80488200	4.10965500	-0.85180900
H	3.68230800	4.91105800	-0.11582900

H	3.70418600	4.54693100	-1.84897800
C	5.21965500	3.54660300	-0.69949300
C	6.29963900	4.56568000	-1.02622400
H	6.23738800	5.41645000	-0.34428500
H	6.15995100	4.92799400	-2.04859200
C	7.67802300	3.94272400	-0.91531900
H	7.86236000	3.68502300	0.13608700
C	7.74700000	2.64019100	-1.75804100
H	7.82407700	2.95994200	-2.80398000
C	6.51746300	1.73339500	-1.56881100
H	6.58547600	1.26342300	-0.58593700
C	9.00160200	1.88846100	-1.39399100
C	-12.30070400	1.62635500	1.65973400
H	-11.66914900	1.73776500	2.54415300
H	-13.19548700	1.06205600	1.92468500
H	-12.59581200	2.62193300	1.32154200
C	-13.02600100	1.82546500	-1.27013900
H	-13.85509100	2.14048900	-0.62900500
H	-13.42785000	1.62736500	-2.26784400
H	-12.33527200	2.66808200	-1.34671500
C	-13.65904200	-3.06496700	-0.20513500
H	-14.05943800	-3.22071300	-1.21318800
H	-14.49010500	-2.81833100	0.45616900
H	-13.20875800	-4.00449500	0.12370700
O	6.50959200	-2.68039000	1.15603300
H	5.70865700	-2.26301200	0.80850600

O	9.50271900	-5.25905300	-0.37935500
H	9.83424000	-5.25748400	0.53321800
O	6.96349700	-3.04347500	-1.72652700
O	6.09119500	-1.09357700	-0.97223200
O	5.45581400	3.08804600	0.59999600
H	4.60177200	2.70209800	0.89681800
O	5.31568700	2.48347000	-1.65121600
O	9.06927200	0.80003900	-0.87176600
O	2.92184300	2.48247100	0.63852500
H	2.67720800	1.53540900	0.63116200
O	0.27704900	1.47694000	-0.30981700
H	-0.38294600	1.64534500	0.38762000
O	-1.62608800	3.15557300	0.24568600
H	-2.38207600	2.53691800	0.30325900
O	-3.73914700	1.48338400	-0.10565700
H	-4.32764800	1.02716100	0.51790600
O	-6.30831800	0.78445700	0.64413200
H	-7.06145600	0.34324400	1.07719600
O	-8.92675000	0.30742000	1.13881200
O	-10.40826500	1.71539400	0.18886800
O	-14.31042700	-0.36720700	0.34716100
H	-14.91595600	0.29036100	-0.01148000
O	8.59887100	4.91022800	-1.37319100
H	9.47954300	4.51474000	-1.35931800
N	9.07070800	-3.63490900	1.84917100
H	8.20007100	-3.83902900	2.33517600

H	9.54127400	-2.90961500	2.38102600
C	8.32679300	2.69882800	2.81983100
H	7.73633700	3.57601700	2.53871400
H	8.50386000	2.74595500	3.89924900
C	7.58818100	1.42407500	2.43046000
H	8.19804300	0.54436600	2.67315900
H	7.44344100	1.41044100	1.34693900
C	6.22904200	1.30752200	3.11361000
H	5.66726400	2.23198400	2.96172900
H	6.36910800	1.19654700	4.19618200
C	5.42273900	0.12747500	2.57166100
H	5.97147400	-0.81295200	2.65130000
H	5.21136300	0.28817000	1.50698800
C	4.09369700	-0.08680000	3.23621300
O	3.65906600	-1.15193800	3.60715500
C	2.07201100	0.99081300	3.86921700
H	2.00054600	0.16601100	4.58163500
H	1.90459100	1.94264200	4.37886600
C	1.03209000	0.81460200	2.77541200
H	1.24716500	1.47682700	1.93888800
C	-0.36929100	1.08540200	3.29763300
H	-0.57366200	0.48221700	4.18572100
H	-0.48231400	2.14568300	3.52959600
O	3.39630600	1.06919000	3.33994800
C	1.81802800	-0.91913100	1.33479100
C	1.68971400	-2.36269100	0.96205900

H	1.16744200	-2.89509200	1.75904800
H	1.05133500	-2.39008000	0.07015600
C	3.06418800	-2.95938700	0.65629000
H	3.66013200	-2.94559800	1.57710100
H	3.56723300	-2.31101700	-0.06810200
C	2.99994800	-4.37569100	0.09805800
H	2.47301700	-5.02931200	0.80442100
H	2.40135000	-4.36922100	-0.82193700
C	4.39092300	-4.93317200	-0.19282700
H	4.99443600	-4.96824500	0.71896600
H	4.93111300	-4.29908200	-0.90158700
O	1.00635700	-0.54921500	2.32125100
O	2.60158900	-0.16112900	0.79100000
O	-1.32849500	0.78431100	2.27695400
O	-3.58824000	-0.27419700	2.22626400
O	-1.69072700	-1.52894800	3.43043200
O	-1.70639400	-1.20720600	0.82393500
C	-4.52193200	-1.26159700	2.68299300
H	-4.05737800	-1.85733800	3.47381900
H	-5.37033700	-0.71713300	3.10434800
C	-5.00324200	-2.12532400	1.52958000
H	-5.48412100	-1.47285800	0.80460800
H	-5.76644500	-2.81955300	1.90744900
N	-3.89734400	-2.81431900	0.84822900
H	-3.60017500	-3.63167200	1.37462600
H	-4.19623400	-3.12763800	-0.07126900

P	-2.01229100	-0.66634600	2.27802600
H	-2.45009700	-1.85808600	0.62438400
O	10.12488100	2.59402500	-1.67301600
H	10.87170200	2.05693100	-1.36149700
H	9.29793800	2.76908600	2.32018100
H	4.33821200	-5.94495800	-0.60613700

AmB-DSPEc3

0 1

C	-10.65989400	2.37919800	1.49046500
H	-11.26441300	1.47944300	1.31517800
C	-11.05971700	3.39338700	0.41885200
H	-10.48767800	4.31922200	0.58973800
C	-10.69143100	2.87852000	-0.96733400
H	-11.22192400	1.92964900	-1.14914300
C	-8.88070000	1.64487900	-0.09351300
H	-9.40308700	0.70564100	-0.33866400
C	-9.18694300	2.00149100	1.35868000
H	-8.96944600	1.11310400	1.97244100
C	-11.01249900	3.86231600	-2.06779300
H	-10.44631000	4.78549600	-1.91753500
H	-12.07802700	4.09618900	-2.05004700
H	-10.74775700	3.44329300	-3.04064300
C	-6.92123500	1.32223400	-1.48195800

H	-7.05232100	2.25115000	-2.04599900
C	-5.46847200	1.08056700	-1.21906100
H	-5.22513400	0.18336500	-0.65507000
C	-4.49535700	1.90818600	-1.61641200
H	-4.74563400	2.80079300	-2.18958600
C	-3.09784900	1.68809800	-1.29926500
H	-2.87385400	0.82294700	-0.68210500
C	-2.07029100	2.46042100	-1.70119700
H	-2.25640400	3.31942900	-2.34537500
C	-0.70454300	2.17959800	-1.32125900
H	-0.56725300	1.36452500	-0.61431300
C	0.39126800	2.80423000	-1.80002600
H	0.28788800	3.60468800	-2.53190000
C	1.72311500	2.41625400	-1.40021400
H	1.77824000	1.65658800	-0.62376100
C	2.87579200	2.91116400	-1.89708800
H	2.84985100	3.68549900	-2.66274400
C	4.17080600	2.47697300	-1.43101800
H	4.16936300	1.68131000	-0.68582300
C	5.35830400	2.98659100	-1.81837400
H	5.37908100	3.77758700	-2.56701300
C	6.62131300	2.57172400	-1.25436600
H	6.58565600	1.79308200	-0.49387700
C	7.82587800	3.07939100	-1.57830800
H	7.89986900	3.83817400	-2.35646300
C	-7.62897800	0.19581400	-2.24293300

H	-8.63144000	0.53588000	-2.50630400
H	-7.09699400	0.01581400	-3.18114500
C	9.05222800	2.65620700	-0.92815000
H	8.92427900	1.97977700	-0.08580200
C	10.29176600	2.99792200	-1.29737300
H	10.43190100	3.65952500	-2.15369700
C	11.54224200	2.50499200	-0.62379000
H	11.26230900	1.98775100	0.30081500
C	12.30052400	1.48162400	-1.49293300
H	12.74629600	2.03306300	-2.33796700
C	11.42541800	0.37312000	-2.09891300
H	10.64987000	0.88781800	-2.67375900
C	10.66742600	-0.44450500	-1.04323200
H	10.31528200	0.21151300	-0.25107600
C	8.29772700	-0.76194100	-1.15111400
C	7.18018400	-1.05309000	-2.11648100
H	7.12085200	-0.16318300	-2.75530100
H	7.45173000	-1.89659800	-2.75472000
C	5.83728400	-1.31114400	-1.44637300
H	5.69920000	-0.57099400	-0.64739400
C	4.69211000	-1.16951400	-2.43607800
H	4.74943000	-0.17357000	-2.88687800
H	4.80436700	-1.91139900	-3.23531800
C	3.31497700	-1.33545400	-1.79994900
H	3.24694000	-0.66863600	-0.92411900
C	2.21359600	-0.96505800	-2.78069200

H	2.21693800	-1.69730600	-3.59785000
H	2.48493500	0.00199800	-3.21472200
C	0.81613300	-0.81610200	-2.18029300
H	0.88597100	-0.30995000	-1.21601000
H	0.23765600	-0.15325000	-2.83087400
C	-0.00850200	-2.08274900	-1.97026900
H	-0.03851600	-2.66435800	-2.90660100
C	-1.44082400	-1.69737600	-1.61208700
H	-1.81137100	-1.00358800	-2.37513300
C	-2.41199600	-2.87018200	-1.51266400
H	-2.08416500	-3.55583400	-0.72478600
H	-2.39802200	-3.42075000	-2.45997900
C	-3.83053600	-2.38234400	-1.22966500
H	-4.09197200	-1.60791300	-1.96314600
C	-4.88276800	-3.47805200	-1.31628000
H	-4.62894500	-4.30046000	-0.64057000
H	-4.89880400	-3.86676000	-2.33837600
C	-6.28165400	-2.97947300	-0.94516100
C	-7.38508600	-3.97720000	-1.28248900
H	-7.28165600	-4.87135000	-0.66465900
H	-7.30252300	-4.26919800	-2.33357500
C	-8.74790800	-3.35112600	-1.04804000
H	-8.83887400	-3.11186300	0.01758600
C	-8.86552400	-2.04736200	-1.84984400
H	-8.78213200	-2.27151400	-2.91799100
C	-7.69186400	-1.11341700	-1.46726000

H	-7.75735800	-0.90632600	-0.39341500
C	-10.19989900	-1.36701200	-1.66934400
C	11.40914800	-1.60853300	-0.41833600
H	10.78575900	-2.07169200	0.34960500
H	12.32995300	-1.24177500	0.03884200
H	11.65634600	-2.36796400	-1.16308400
C	12.21539300	-0.50191400	-3.07012000
H	13.04770200	-1.01522300	-2.57840400
H	12.62389800	0.09825600	-3.88843100
H	11.57143900	-1.27004900	-3.50449900
C	12.46988400	3.66620700	-0.25461800
H	12.74911300	4.23676100	-1.14732800
H	13.38252700	3.28873500	0.20895500
H	11.97649200	4.35175800	0.43840100
O	-8.40075200	3.09472600	1.78187800
H	-7.57696400	3.04389400	1.27477000
O	-12.44795600	3.62130700	0.47843300
H	-12.64002400	3.68033800	1.42820600
O	-9.28279100	2.64497400	-0.99211100
O	-7.49877800	1.50591900	-0.17022700
O	-6.37896800	-2.70222000	0.42505100
H	-5.55452100	-2.23926900	0.66138500
O	-6.47572500	-1.79879300	-1.72123400
O	-10.68773700	-0.56257100	-2.42670400
O	-3.90351400	-1.80934800	0.08090300
H	-3.07170300	-1.31408100	0.20505200

O	-1.44149600	-0.94209400	-0.39394800
H	-0.86410900	-1.38481700	0.25108800
O	0.47369200	-2.88784700	-0.90601500
H	1.44400800	-2.98521800	-1.02775200
O	3.11039100	-2.68284600	-1.37477200
H	3.93289400	-2.96318300	-0.92833600
O	5.79293900	-2.62051700	-0.89825000
H	6.19152900	-2.60303800	-0.00161500
O	8.14573600	-0.33017600	-0.02641500
O	9.48897500	-0.95648500	-1.72306400
O	13.33391300	0.95570600	-0.66459000
H	13.97480800	0.50370800	-1.22399400
O	-9.73702200	-4.28668000	-1.43901500
H	-10.56400900	-4.01099200	-1.02582500
N	-11.03950600	2.92827200	2.78777900
H	-10.33700300	3.61266500	3.05933400
H	-11.03731400	2.20595100	3.50091200
C	-5.97893400	0.35859400	2.68645900
H	-6.24719900	-0.47147600	2.02619900
H	-6.17732700	0.04633400	3.71710100
C	-4.51789600	0.75850500	2.50946900
H	-4.31216900	1.67036600	3.08394800
H	-4.33563000	1.01230400	1.45934700
C	-3.54374600	-0.33146000	2.94986800
H	-3.75085200	-1.25824000	2.40576200
H	-3.68665700	-0.54962700	4.01425200

C	-2.08336800	0.08464200	2.71990200
H	-1.86365500	0.98415400	3.30075000
H	-1.90585300	0.30164300	1.66466100
C	-1.12969600	-0.97928100	3.18429000
O	-0.72055700	-1.11022100	4.31012700
C	0.21200100	-2.81246300	2.42024200
H	0.20127100	-3.08143000	3.47807900
H	-0.05107200	-3.66557500	1.79359600
C	1.56808000	-2.26809600	2.00079300
H	1.53433400	-1.97259600	0.95325000
C	2.66927700	-3.27862400	2.21246800
H	2.84100900	-3.45184900	3.27724000
H	2.41259800	-4.22015800	1.72307700
O	-0.78591500	-1.81898800	2.16845700
C	1.64725200	0.09055800	2.31909100
C	1.83837800	1.13226700	3.38802900
H	1.07743700	0.91216500	4.14569100
H	2.80258100	0.94016600	3.86896200
C	1.72596900	2.55802200	2.86425100
H	1.59274700	3.24159300	3.70963700
H	0.82530900	2.63622200	2.24617000
C	2.93684100	2.98970200	2.04177200
H	3.83050400	2.96543500	2.67993600
H	3.09724700	2.25531800	1.24780200
C	2.77986900	4.37483700	1.42789800
H	2.65029900	5.13782300	2.20219000

H	1.90719600	4.40660600	0.77017600
O	1.88127700	-1.13409000	2.81559700
O	1.26711200	0.29895600	1.18749600
O	3.87396700	-2.83050800	1.58612800
O	4.64303600	-0.45890900	1.98456200
O	6.37162600	-2.33201000	1.72028300
O	4.84668800	-2.21140400	3.82395500
C	5.69557900	0.52399500	2.01101400
H	6.56236200	0.14482200	1.46285900
H	5.29821800	1.39515800	1.48956200
C	6.06874000	0.92424500	3.42736900
H	5.17947500	1.28690800	3.95030400
H	6.77769900	1.76029600	3.35563700
N	6.61087100	-0.21383300	4.18661000
H	7.41908800	-0.60366200	3.70375900
H	6.91846000	0.08411300	5.10715800
P	5.03427900	-1.99977000	2.27091700
H	5.46128700	-1.52959200	4.24225200
O	-10.82833000	-1.71256000	-0.51872800
H	-11.65645000	-1.20534000	-0.50423600
H	-6.64264600	1.19331100	2.45368900
H	3.65312400	4.64142800	0.82703800

AmB-DSPEc4

0 1

C	9.32201500	-4.66269900	0.59471600
H	9.80945800	-3.74619500	0.95839600
C	9.55145700	-4.70838000	-0.91776200
H	9.09424700	-5.62749400	-1.30851200
C	8.88828800	-3.50952200	-1.59945400
H	9.33932600	-2.58616600	-1.20270300
C	7.24751800	-3.38337200	0.08057000
H	7.70012100	-2.44017700	0.43230300
C	7.83124800	-4.54051000	0.88310100
H	7.68178000	-4.33524300	1.95256700
C	9.01738400	-3.52401300	-3.10642000
H	8.56120200	-4.42934900	-3.51742200
H	10.06950700	-3.48530700	-3.39884800
H	8.51238700	-2.65594300	-3.53422100
C	5.07235900	-2.55111000	-0.54992900
H	5.07426800	-2.97205800	-1.56151100
C	3.68796000	-2.59629200	0.01702500
H	3.58671000	-2.36551500	1.07503800
C	2.60144900	-2.84817000	-0.72143700
H	2.71362100	-3.06254600	-1.78537100
C	1.25169600	-2.84500000	-0.19009900
H	1.14663000	-2.72720900	0.88640500
C	0.14125700	-2.95829700	-0.94219300
H	0.23324700	-3.05716100	-2.02384600
C	-1.19315700	-2.91771400	-0.39291300

H	-1.28243200	-2.86163200	0.68767900
C	-2.32134600	-2.90911700	-1.12932000
H	-2.25038100	-2.95757900	-2.21575300
C	-3.64238000	-2.80189800	-0.56035200
H	-3.70345200	-2.71334000	0.52270500
C	-4.76674600	-2.74899600	-1.30376200
H	-4.67705000	-2.81345400	-2.38841400
C	-6.10097800	-2.60650500	-0.77768800
H	-6.21650300	-2.56074100	0.30519800
C	-7.20104400	-2.53475300	-1.55745400
H	-7.07821500	-2.56837800	-2.64013400
C	-8.55171400	-2.45383300	-1.05746600
H	-8.68061600	-2.48588700	0.02244100
C	-9.64326800	-2.34091800	-1.83664000
H	-9.52371800	-2.28089200	-2.91809300
C	5.65033700	-1.13536800	-0.63693400
H	6.60271400	-1.18072400	-1.16446600
H	4.98136300	-0.53117200	-1.25169100
C	-10.99073400	-2.25786100	-1.30838200
H	-11.09055400	-2.43712300	-0.24027200
C	-12.07355100	-1.92917300	-2.02138300
H	-11.96706500	-1.73203100	-3.08951700
C	-13.45262800	-1.75291100	-1.45281400
H	-13.46308100	-2.12657000	-0.42259400
C	-13.84535000	-0.26308400	-1.39969100
H	-13.97405800	0.07910800	-2.44017300

C	-12.79368700	0.66291200	-0.76079800
H	-11.88140400	0.52863700	-1.34984400
C	-12.41742100	0.25443700	0.66781500
H	-12.25919900	-0.81938700	0.71417700
C	-10.13318000	0.13460800	1.39390800
C	-8.87654900	0.92797400	1.63950700
H	-8.88929300	1.83277600	1.02917700
H	-8.89579500	1.23705300	2.69128200
C	-7.60083000	0.11773100	1.39029500
H	-7.56326400	-0.17708100	0.33155000
C	-6.37303200	0.95612200	1.71739900
H	-6.32432500	1.77207000	0.99080600
H	-6.48525000	1.38845400	2.71892700
C	-5.04829100	0.19881300	1.67451400
H	-4.95157300	-0.29689900	0.69908000
C	-3.87882100	1.15611100	1.87238800
H	-3.91147300	1.51420700	2.90877500
H	-4.06329200	2.02536900	1.23422300
C	-2.48283200	0.62403100	1.54070300
H	-2.48746800	0.14292600	0.55798800
H	-1.81118900	1.48275600	1.45612400
C	-1.87582200	-0.37077600	2.51646300
H	-1.90938300	0.03288200	3.54140500
C	-0.41926400	-0.68209000	2.15297900
H	-0.36014500	-0.81447900	1.06420000
C	0.58667900	0.38458300	2.56051200

H	0.50946700	0.57368300	3.63774300
H	0.35570200	1.31211300	2.02666900
C	2.02309800	-0.01733300	2.22605000
H	2.07845500	-0.26217300	1.15778700
C	3.00997300	1.10589500	2.52239000
H	2.88985000	1.43722900	3.55863600
H	2.79687200	1.95532900	1.86950600
C	4.47103900	0.69266200	2.34576100
C	5.43978000	1.86790000	2.40942700
H	5.43024800	2.29683900	3.41354800
H	5.12385700	2.63218000	1.69878800
C	6.84426900	1.40506400	2.07252900
H	7.17716000	0.70140200	2.84224300
C	6.85623700	0.69404600	0.71440000
H	6.55328700	1.39578900	-0.06590300
C	5.80836600	-0.44427400	0.70853700
H	6.07333900	-1.17576500	1.48002100
C	8.22700900	0.19952200	0.32956600
C	-13.37056800	0.67282400	1.76877800
H	-13.02990900	0.27751800	2.72850000
H	-14.36312400	0.27718700	1.54901400
H	-13.43057800	1.76049300	1.84675500
C	-13.20550200	2.13039500	-0.86385700
H	-14.15286500	2.32647900	-0.35271400
H	-13.31872100	2.42735300	-1.91053500
H	-12.45021600	2.77628000	-0.41050200

C	-14.49136800	-2.54226000	-2.25438900
H	-14.49686400	-2.22030500	-3.30172300
H	-15.48935600	-2.38443100	-1.84339000
H	-14.26344200	-3.61040300	-2.23468200
O	7.21182400	-5.75302200	0.50845600
H	6.26870800	-5.55578800	0.42069300
O	10.95887700	-4.69813200	-1.11579100
H	11.14760600	-5.05419300	-1.99003000
O	7.49649500	-3.52561700	-1.29713600
O	5.87218100	-3.40032500	0.29359600
O	4.87409900	-0.23227700	3.31066200
H	4.13360200	-0.86191900	3.40303900
O	4.54462900	0.11396200	1.03224100
O	8.58476400	-0.07481900	-0.79554500
O	2.43386100	-1.16159100	2.98294800
H	1.65594300	-1.73348900	3.10758500
O	-0.05984700	-1.91509400	2.78465000
H	-0.89975900	-2.38211100	2.93758100
O	-2.53218800	-1.63378900	2.48248800
H	-3.47921900	-1.49326000	2.70591900
O	-5.00307400	-0.78038900	2.71847800
H	-5.86744600	-1.22823500	2.73179600
O	-7.58254300	-1.05553100	2.19885700
H	-8.46993800	-1.45365000	2.15238100
O	-10.22175000	-1.06170100	1.62370700
O	-11.12531000	0.88371200	0.93439000

O	-15.09535600	-0.20443600	-0.71958800
H	-15.51004800	0.64438700	-0.90737600
O	7.69200400	2.54839100	2.04748800
H	8.59455200	2.23390800	2.17880900
N	9.87711400	-5.78796700	1.32198200
H	9.31941800	-6.61221200	1.11582500
H	10.81869600	-5.95803900	0.98181000
C	10.55471200	2.64222100	-1.51719900
H	10.34669200	1.56831100	-1.51299600
H	10.80316300	2.92787100	-2.54435500
C	9.34855200	3.42425500	-1.00725800
H	9.57642000	4.49711700	-0.98951600
H	9.12560100	3.14473600	0.02862300
C	8.09822800	3.17886700	-1.84716700
H	7.88178800	2.10721600	-1.86149200
H	8.28493800	3.47363400	-2.88718300
C	6.88607900	3.94652700	-1.30719500
H	7.02225200	5.02530900	-1.40313800
H	6.75713800	3.72111900	-0.24142800
C	5.59956900	3.59918500	-1.99957900
O	4.83390800	4.37243800	-2.52271400
C	4.16817200	1.77033700	-2.52630200
H	3.87026900	2.39098300	-3.37456700
H	4.39605900	0.76090300	-2.87476400
C	3.04986000	1.73225200	-1.49886300
H	3.45473200	1.48489300	-0.51551300

C	1.97660400	0.72506000	-1.89858800
H	1.68762800	0.85362800	-2.94637000
H	2.36536500	-0.28392600	-1.75119800
O	5.38080000	2.26062600	-1.96144400
C	2.78041100	3.89816000	-0.46785200
C	1.93249300	5.13173100	-0.50916700
H	1.93490500	5.50074200	-1.54114600
H	0.90716300	4.81119000	-0.28380400
C	2.41348800	6.20142700	0.46067700
H	3.44658400	6.47043800	0.21513000
H	2.44225000	5.78002700	1.47080600
C	1.52942100	7.44406600	0.43789300
H	1.50338100	7.85367200	-0.57964500
H	0.49787700	7.15900100	0.67857600
C	2.00730600	8.51721700	1.41035600
H	3.02590900	8.83779300	1.17066600
H	2.01404700	8.13971200	2.43756200
O	2.41119900	3.02362500	-1.44156700
O	3.66692100	3.66470700	0.31261300
O	0.82952800	0.83316500	-1.05901300
O	-1.54511500	1.05693500	-1.70475700
O	0.03934700	2.69911500	-2.64173800
H	0.83804800	3.24067200	-2.51261800
O	-0.50542400	2.81516300	-0.09007600
C	-2.85827300	1.65030600	-1.72467600
H	-3.06308500	2.12324400	-0.76290200

H	-2.89587600	2.41407400	-2.50968100
C	-3.83351200	0.51888100	-1.97855000
H	-3.58999400	-0.28367600	-1.28391700
H	-3.66188400	0.11703600	-2.98867300
N	-5.20597200	0.94419500	-1.71892700
H	-5.55179200	1.52700400	-2.47540600
H	-5.80520000	0.12442700	-1.68241800
P	-0.30305600	1.94753600	-1.26828500
O	9.06769400	0.05620600	1.37694200
H	9.90368100	-0.27895400	1.01366500
H	11.43971300	2.82424400	-0.90049000
H	1.36341500	9.40027300	1.38271300

AmB-DSPEc5

0 1

C	6.19543700	-4.45567900	0.45056800
H	6.81860200	-3.58265300	0.69283000
C	6.46781800	-4.79998200	-1.01297000
H	5.90188000	-5.70290100	-1.27482000
C	6.03467800	-3.65747600	-1.92899600
H	6.59961700	-2.75221200	-1.66594300
C	4.38117500	-2.97218100	-0.40434000
H	4.96556100	-2.06125800	-0.21615300
C	4.74345000	-4.03015700	0.63295500

H	4.61635000	-3.59727800	1.63527000
C	6.20951500	-3.95522700	-3.40249900
H	5.64051600	-4.84630700	-3.68317700
H	7.26340200	-4.10350000	-3.65270900
H	5.84571300	-3.11101200	-3.99060900
C	2.35243300	-2.01954500	-1.33283200
H	2.26169600	-2.69320300	-2.19182900
C	0.99641000	-1.69402000	-0.79052000
H	0.96280800	-1.02039900	0.06186700
C	-0.14125100	-2.17139400	-1.30394700
H	-0.10676900	-2.84323400	-2.16154500
C	-1.44760700	-1.84887300	-0.76568100
H	-1.46825800	-1.16753200	0.08160200
C	-2.61120600	-2.34136700	-1.23074300
H	-2.60370300	-3.02707200	-2.07782500
C	-3.88907500	-2.00808500	-0.64862100
H	-3.86369300	-1.30470200	0.18135400
C	-5.07873700	-2.48545800	-1.06619800
H	-5.11735700	-3.18568900	-1.90017000
C	-6.32925800	-2.11033700	-0.45441900
H	-6.28105200	-1.38768900	0.35995900
C	-7.53912500	-2.57321000	-0.82999100
H	-7.60685200	-3.28318900	-1.65363600
C	-8.77012400	-2.19095700	-0.18492300
H	-8.69301200	-1.49340800	0.64882000
C	-9.99202600	-2.63266600	-0.54505200

H	-10.08398100	-3.31357700	-1.39053600
C	-11.20638000	-2.25620800	0.13684400
H	-11.09592900	-1.62599400	1.01763200
C	-12.44751600	-2.60979200	-0.24416600
H	-12.58241800	-3.21783300	-1.13801600
C	3.14052300	-0.78741600	-1.78178000
H	4.10203800	-1.11211400	-2.17924700
H	2.59593000	-0.32005300	-2.60726200
C	-13.63856600	-2.17906300	0.46147100
H	-13.46625700	-1.66256200	1.40348100
C	-14.89111800	-2.31973400	0.01447800
H	-15.06258100	-2.82190400	-0.93910100
C	-16.11196000	-1.78573500	0.70773800
H	-15.82987300	-1.43844000	1.70832500
C	-16.69303000	-0.57475000	-0.04836300
H	-17.10198800	-0.95018500	-1.00128500
C	-15.66891600	0.51664600	-0.41204400
H	-14.90378600	0.01798100	-1.01473400
C	-14.92334500	1.07851400	0.80390400
H	-14.58994800	0.26354700	1.44064200
C	-12.54168500	1.38549800	0.77612800
C	-11.40320400	2.11351800	0.10667300
H	-11.71031300	2.42689400	-0.89286400
H	-11.20256200	3.01734000	0.69357100
C	-10.12939000	1.26663300	0.05342100
H	-10.33066900	0.34973500	-0.51887500

C	-8.98928100	2.02998800	-0.60469600
H	-9.28571700	2.28954500	-1.62667200
H	-8.82476200	2.96649300	-0.05837300
C	-7.66975400	1.26330000	-0.65266400
H	-7.83404900	0.27923800	-1.11258400
C	-6.62050900	2.01753000	-1.46286800
H	-6.61072800	3.06571000	-1.13963100
H	-6.94860700	2.01544600	-2.50681800
C	-5.20654800	1.42466600	-1.34824900
H	-5.25525500	0.38299700	-1.02227200
H	-4.72492500	1.41251600	-2.33086100
C	-4.28588900	2.20599400	-0.41288300
H	-4.11660500	3.20062000	-0.84823800
C	-2.91303500	1.50531900	-0.24335100
H	-2.72743800	0.84057300	-1.09487600
C	-1.75547800	2.48986600	-0.11564600
H	-1.92834800	3.13876000	0.75161500
H	-1.73567900	3.12777600	-1.00688900
C	-0.40440500	1.79418300	0.02195500
H	-0.26944000	1.10638100	-0.82394700
C	0.75222200	2.78619000	0.01731700
H	0.61510800	3.52006800	0.81761200
H	0.75057100	3.31803500	-0.93860900
C	2.11825100	2.12767200	0.21473200
C	3.27964700	3.07949100	-0.03717600
H	3.27651900	3.88389600	0.70211500

H	3.16997400	3.52337400	-1.03133200
C	4.60756000	2.34456600	0.02378900
H	4.76156800	1.98647300	1.04768900
C	4.59987600	1.13006400	-0.91384600
H	4.61290900	1.48462100	-1.94877200
C	3.32883100	0.27175900	-0.70263600
H	3.38942000	-0.19205100	0.28986100
C	5.80916100	0.23788300	-0.73338100
C	-15.65977600	2.10692500	1.63738300
H	-15.05611900	2.39013100	2.50270400
H	-16.60177200	1.67678800	1.98005800
H	-15.87128900	3.00702000	1.05618100
C	-16.30063000	1.61112500	-1.27035800
H	-17.12830900	2.10914200	-0.75618900
H	-16.68610700	1.19450500	-2.20541300
H	-15.56410100	2.37760000	-1.52147600
C	-17.18224500	-2.86936300	0.86579000
H	-17.47561500	-3.26731000	-0.11201700
H	-18.07063200	-2.46002100	1.34853400
H	-16.80312400	-3.70080200	1.46428700
O	3.92924600	-5.17171000	0.46715300
H	3.04176300	-4.83429800	0.27636500
O	7.87625200	-5.04815400	-1.10549100
H	8.07773700	-5.41878500	-1.97144100
O	4.64636000	-3.43526600	-1.71048600
O	3.01598400	-2.74388800	-0.27182400

O	2.26350700	1.61590700	1.50812700
H	1.41722800	1.18734900	1.72984700
O	2.17140000	1.08984700	-0.75975700
O	6.30460900	-0.42071700	-1.62525700
O	-0.34033300	1.04208000	1.23662300
H	-1.24024400	0.71858900	1.42471000
O	-2.98230300	0.66964900	0.91365200
H	-3.65074200	1.12537300	1.46699200
O	-4.82611800	2.39159700	0.88798500
H	-5.70466400	1.95769000	0.93826600
O	-7.15021300	1.06116200	0.66465800
H	-7.90706700	0.89897100	1.25678300
O	-9.70989900	0.91541700	1.36956800
H	-10.49377500	0.57692000	1.83780500
O	-12.37939800	0.59472500	1.69206900
O	-13.72045200	1.71588200	0.26943300
O	-17.75177500	-0.06896600	0.75940400
H	-18.31717000	0.48900900	0.21465600
O	5.60500000	3.27457400	-0.34709500
H	6.47538300	2.84363100	-0.36723900
N	6.51441300	-5.53622900	1.37391700
H	5.72446200	-6.17564400	1.40032600
H	7.30976800	-6.05512500	1.01365900
C	4.44689700	7.04156100	-1.66056200
H	3.94368300	6.56817700	-0.81142200
H	4.71767600	8.05911700	-1.36010000

C	5.67703200	6.23840700	-2.06838700
H	6.14616400	6.69480500	-2.94956100
H	5.37137400	5.22647700	-2.35703700
C	6.69182800	6.11527200	-0.93626800
H	6.20811700	5.62444700	-0.09109600
H	7.01383300	7.11058100	-0.60527600
C	7.90530700	5.28709000	-1.35302000
H	8.53269300	5.80210300	-2.08440200
H	7.56020200	4.35953900	-1.82649600
C	8.80730400	4.81500200	-0.24632300
O	9.98844600	4.58767500	-0.35362400
C	8.77430200	3.90485900	1.95180300
H	9.84868500	4.09855200	1.92320400
H	8.34515600	4.30031400	2.87531000
C	8.50831100	2.41261300	1.84957300
H	7.48188600	2.23847900	1.51777800
C	8.75518800	1.68081600	3.15748300
H	9.81512000	1.73106000	3.42262600
H	8.15071800	2.11177300	3.95704000
O	8.11296300	4.58994000	0.89420900
C	9.10219500	1.60620300	-0.35063800
C	10.14641200	0.77990600	-1.04517600
H	10.77257200	1.46900700	-1.62383900
H	10.77473400	0.32725200	-0.27497200
C	9.51418500	-0.28289800	-1.94545400
H	9.01892800	0.19510200	-2.79569400

H	8.71790300	-0.78603300	-1.39507200
C	10.52546000	-1.31955700	-2.42503400
H	11.25776700	-0.85229100	-3.09442900
H	11.09743900	-1.68938600	-1.56359900
C	9.85195200	-2.49729800	-3.12560400
H	9.23126600	-2.15480300	-3.95948500
H	9.19866700	-3.03804900	-2.43184400
O	9.42444300	1.80262000	0.93132300
O	8.08571000	2.02879100	-0.86372600
O	8.34234600	0.31686000	3.04397800
O	8.97513600	-2.04302500	3.36753800
O	10.73840800	-0.48008100	2.31065100
O	8.55836200	-1.16323900	1.04614700
C	9.83995100	-3.18398000	3.28721300
H	10.87255800	-2.84565700	3.14872600
H	9.76524000	-3.68970900	4.25176600
C	9.41112100	-4.14150900	2.18732600
H	8.36231000	-4.41076700	2.33520300
H	10.00523300	-5.06020600	2.28571100
N	9.54715500	-3.54561800	0.84402800
H	10.52760900	-3.42719900	0.59833700
H	9.11130900	-4.13852200	0.13433000
P	9.29980700	-0.78985600	2.41121400
H	8.88549900	-2.13188700	0.80875300
O	6.21633400	0.16550900	0.53605200
H	7.01153500	-0.41139500	0.61244800

H	3.72460700	7.11642000	-2.47847600
H	10.58589500	-3.20569000	-3.52160000

AmB-DSPEc6

0 1

C	-7.81454200	4.00076200	-0.27901000
H	-8.41722100	3.62544900	-1.12551800
C	-7.24719100	5.34391300	-0.70747700
H	-6.61312900	5.72017800	0.10336300
C	-6.40142000	5.16882200	-1.97898800
H	-7.08536400	4.82987900	-2.77600900
C	-5.87556800	2.93536600	-1.39435100
H	-6.52434500	2.53698000	-2.18866700
C	-6.65513700	3.03269800	-0.09381900
H	-7.04265200	2.03414100	0.13405100
C	-5.72099000	6.45198700	-2.40617000
H	-5.04795100	6.79861900	-1.61692100
H	-6.46255900	7.22667900	-2.61547700
H	-5.13124500	6.28512500	-3.30959000
C	-4.06505900	1.66167000	-2.29919100
H	-3.97994400	2.50298600	-2.99676200
C	-2.70366700	1.27447500	-1.81455400
H	-2.65499200	0.45075700	-1.10624900
C	-1.58359400	1.89203200	-2.20262900

H	-1.63934400	2.73182700	-2.89467400
C	-0.27337800	1.51382700	-1.71582600
H	-0.23749400	0.63122300	-1.08364500
C	0.87039300	2.18081000	-1.96146600
H	0.85044800	3.07175600	-2.58845000
C	2.13451500	1.78258300	-1.39076800
H	2.11539700	0.87996200	-0.78510400
C	3.30577900	2.43248700	-1.54257000
H	3.35351700	3.33653400	-2.14867000
C	4.52106900	1.98355000	-0.90996400
H	4.45324300	1.07215800	-0.31566200
C	5.71835200	2.59738700	-1.00207100
H	5.81293800	3.50059800	-1.60384200
C	6.89765500	2.13010000	-0.31751700
H	6.78417100	1.24255700	0.30511400
C	8.11188100	2.71119300	-0.39387300
H	8.24558800	3.58616200	-1.02898700
C	9.26335600	2.23840500	0.33552400
H	9.09866200	1.40796300	1.01997200
C	10.50969700	2.73240900	0.22089100
H	10.70455600	3.54543800	-0.47752000
C	-4.81579800	0.51204100	-2.98176100
H	-5.81417900	0.86292800	-3.24669800
H	-4.30190500	0.25070300	-3.91190300
C	11.63479100	2.19775800	0.96294700
H	11.38601600	1.46471300	1.72769100

C	12.91916700	2.49423200	0.73746600
H	13.17026500	3.21634000	-0.04126900
C	14.08015600	1.86356900	1.45217200
H	13.70230100	1.28131500	2.30040200
C	14.83617400	0.88638300	0.53069500
H	15.33389400	1.48931700	-0.24702700
C	13.94712100	-0.13212800	-0.20750400
H	13.23598600	0.46045400	-0.79134000
C	13.08757900	-0.98172900	0.73516100
H	12.62080100	-0.34491400	1.48151100
C	10.75777800	-1.38179300	0.31378500
C	9.76878800	-1.98669100	-0.65046400
H	10.21494400	-2.03515300	-1.64556300
H	9.57802100	-3.01424700	-0.31958300
C	8.44307700	-1.22224300	-0.67656300
H	8.63066500	-0.18710300	-0.99627000
C	7.45439600	-1.87258800	-1.63330500
H	7.88443000	-1.86396500	-2.64064000
H	7.31150400	-2.92009400	-1.34184500
C	6.08684900	-1.19537300	-1.67399500
H	6.21499600	-0.12097100	-1.86377200
C	5.20718100	-1.78774400	-2.77068600
H	5.25900800	-2.88195300	-2.71406100
H	5.64457900	-1.50269400	-3.73230800
C	3.74167900	-1.33052800	-2.69457300
H	3.66343600	-0.40755600	-2.11542800

H	3.36833200	-1.09390800	-3.69563200
C	2.79907200	-2.37738900	-2.10232200
H	2.73289300	-3.22241600	-2.80141800
C	1.37925000	-1.80003600	-1.88872900
H	1.20555500	-0.97161300	-2.58510600
C	0.27395300	-2.83814300	-2.05812900
H	0.44764900	-3.66703000	-1.36127300
H	0.32645000	-3.24334600	-3.07546900
C	-1.11820900	-2.25559600	-1.82255700
H	-1.26265500	-1.38913600	-2.48303300
C	-2.22281600	-3.26531600	-2.11865400
H	-2.05497900	-4.17802200	-1.53865200
H	-2.18522700	-3.52150000	-3.18199300
C	-3.62045800	-2.75035700	-1.77437600
C	-4.75356900	-3.65006500	-2.25094800
H	-4.67636900	-4.63954500	-1.79339500
H	-4.70268400	-3.76186400	-3.33732700
C	-6.07742700	-3.01882900	-1.85774500
H	-6.11509100	-2.96416400	-0.76855000
C	-6.18931500	-1.57725300	-2.39994100
H	-6.35955400	-1.64776000	-3.48338200
C	-4.91042000	-0.75206500	-2.12862000
H	-4.90152600	-0.48974900	-1.06601300
C	-7.36784200	-0.84860200	-1.77755800
C	13.77493000	-2.14511900	1.42007500
H	13.08409600	-2.63441400	2.11049900

H	14.63566900	-1.77112700	1.97605000
H	14.11638000	-2.88498400	0.69291500
C	14.76220800	-0.97999800	-1.18264800
H	15.54247200	-1.55348700	-0.67308500
H	15.24353200	-0.34827600	-1.93486700
H	14.12014400	-1.69391800	-1.70334000
C	15.04221500	2.92380500	1.99545100
H	15.42544800	3.54950800	1.18168700
H	15.89085900	2.45090600	2.49151000
H	14.53298400	3.57733500	2.70737300
O	-5.80821000	3.51007500	0.93567400
H	-4.91635800	3.17501700	0.75649600
O	-8.34475100	6.21544700	-0.96034300
H	-8.04933400	7.12103900	-0.82337900
O	-5.37731000	4.20496700	-1.77842900
O	-4.79387100	2.10397400	-1.14351900
O	-3.79284300	-2.61683100	-0.39646500
H	-3.08923200	-2.03094400	-0.06418800
O	-3.74399800	-1.50298800	-2.44436900
O	-7.38545500	0.31031400	-1.44899900
O	-1.25628000	-1.82560100	-0.46828400
H	-0.39568300	-1.46023000	-0.19322600
O	1.33792100	-1.23458400	-0.57691700
H	1.96414300	-1.79263700	-0.07210600
O	3.23596400	-2.88795600	-0.85072900
H	4.05332200	-2.41871300	-0.57849400

O	5.40264000	-1.35161900	-0.42696800
H	6.06761000	-1.28584600	0.28237300
O	7.84662200	-1.21273200	0.61764700
H	8.53962300	-0.95318000	1.25053400
O	10.42598000	-0.84157500	1.35707400
O	12.01081200	-1.52316400	-0.09295200
O	15.81836100	0.25037500	1.34332400
H	16.48682500	-0.14115900	0.77101800
O	-7.11976300	-3.86299700	-2.34730500
H	-7.93039100	-3.33701500	-2.30764200
N	-8.59537300	4.12884700	0.94348200
H	-9.16705800	3.29759500	1.06333300
H	-9.23125400	4.91304200	0.83005600
C	1.96631200	-0.08458500	2.30678100
H	2.34751300	0.10910800	3.31425200
H	1.59396200	-1.11484000	2.29106400
C	0.85701800	0.89254800	1.93277400
H	0.54649800	0.69605700	0.90433800
H	1.24400800	1.91891700	1.94077600
C	-0.35847700	0.78456300	2.84643800
H	-0.08704900	1.03939100	3.87685100
H	-0.70238200	-0.25374100	2.87755900
C	-1.51714200	1.66138400	2.38988600
H	-1.81779000	1.41966400	1.36404900
H	-1.25360500	2.72471700	2.38711800
C	-2.74557200	1.47016100	3.23506800

O	-2.85146900	0.68341500	4.14640700
C	-5.04959500	2.04183200	3.38463400
H	-4.95708000	1.68700700	4.41262700
H	-5.55436300	3.00520500	3.33970200
C	-5.80346500	1.03048500	2.53570100
H	-5.55630100	1.18728500	1.48860500
C	-7.30223200	1.12382900	2.72208700
H	-7.60264400	0.79967500	3.72220700
H	-7.62704400	2.14912600	2.53770300
O	-3.74895600	2.27532600	2.82570800
C	-4.42967300	-0.86413800	2.17311200
C	-4.10233100	-2.25933600	2.61599200
H	-4.56875100	-2.45748500	3.58187600
H	-4.57893900	-2.90377900	1.87109700
C	-2.58811200	-2.49485500	2.62375000
H	-2.17196700	-2.14454100	3.57467900
H	-2.13753300	-1.87417200	1.84545500
C	-2.18952700	-3.94243300	2.35958400
H	-2.57525400	-4.59477100	3.15296200
H	-2.66543600	-4.26073000	1.42498700
C	-0.67661200	-4.09572500	2.23490200
H	-0.16635700	-3.75387200	3.14163800
H	-0.31243400	-3.49539300	1.39534500
O	-5.41829500	-0.30720300	2.89052100
O	-3.86751600	-0.28810100	1.26335200
O	-7.97214600	0.31468000	1.73433300

O	-9.50963900	-1.45959100	0.99585100
O	-8.48983400	-1.64787400	3.37704600
O	-7.07101000	-1.98659500	1.20072900
C	-10.25692300	-2.67421400	1.18525900
H	-10.15978300	-2.99257600	2.22741900
H	-11.30553100	-2.43075300	0.99670200
C	-9.80973600	-3.76914200	0.22339600
H	-9.92389200	-3.40896400	-0.80021400
H	-10.49163000	-4.62128600	0.34072900
N	-8.40158600	-4.15035400	0.41491100
H	-8.30973900	-4.87843900	1.11667400
H	-7.99151600	-4.48584800	-0.45721000
P	-8.21959900	-1.23608000	1.98860000
H	-7.46186900	-2.87278000	0.87952100
O	-8.44985500	-1.63934200	-1.59037400
H	-9.02439300	-1.21358800	-0.92057800
H	-0.38452500	-5.13513800	2.05926500
H	2.80854900	-0.02112700	1.61078200

AmB-DSPEc7

0 1

C	10.50039000	-2.98146200	2.35016800
H	11.06849000	-2.08715700	2.06241600
C	10.93953300	-4.10386100	1.40922300

H	10.40676300	-5.02337500	1.69798700
C	10.55020900	-3.77984600	-0.02781900
H	11.04914300	-2.84589100	-0.33142800
C	8.70202700	-2.50427300	0.69376700
H	9.20345400	-1.58749200	0.33795600
C	9.01270000	-2.68664800	2.17832900
H	8.74990300	-1.74860100	2.69478700
C	10.89959200	-4.88401700	-0.99761100
H	10.35995700	-5.79698000	-0.73169700
H	11.97146100	-5.08302900	-0.95400200
H	10.62433300	-4.59726100	-2.01454700
C	6.72762100	-2.47285900	-0.72006700
H	6.80218800	-3.50674300	-1.07212100
C	5.29673500	-2.09081400	-0.50908400
H	5.12194700	-1.09160900	-0.12235100
C	4.25019500	-2.88303400	-0.75789600
H	4.40808000	-3.89036300	-1.14262100
C	2.88300100	-2.46021800	-0.52097100
H	2.75331900	-1.44198800	-0.16025700
C	1.79642800	-3.23321900	-0.70102100
H	1.91651700	-4.25536700	-1.05943000
C	0.45478200	-2.77546100	-0.42650900
H	0.35303700	-1.74265800	-0.09559600
C	-0.65448900	-3.52984400	-0.56145900
H	-0.56401900	-4.56186600	-0.89912200
C	-1.97932200	-3.04308100	-0.26869400

H	-2.05905600	-2.00602600	0.05560700
C	-3.10599500	-3.77765800	-0.36989800
H	-3.03998200	-4.81110000	-0.70845900
C	-4.41205300	-3.27642400	-0.02247300
H	-4.46501200	-2.25175600	0.34392200
C	-5.55871900	-3.97900000	-0.11925800
H	-5.53484100	-4.99441500	-0.51320600
C	-6.83794400	-3.44906800	0.28655600
H	-6.82627900	-2.46997900	0.76043600
C	-8.02723500	-4.05391500	0.11448100
H	-8.07553200	-5.02167800	-0.38330400
C	7.46181700	-1.57013500	-1.71819300
H	8.48240300	-1.93456400	-1.83611500
H	6.97451500	-1.66018800	-2.69272200
C	-9.27571100	-3.44306200	0.53009000
H	-9.17838100	-2.53937300	1.12767700
C	-10.49662400	-3.86704000	0.18787900
H	-10.59837200	-4.76062100	-0.43019700
C	-11.77551600	-3.16924200	0.55555700
H	-11.55832500	-2.40140300	1.30692500
C	-12.38853800	-2.45566900	-0.66490100
H	-12.70114800	-3.23894000	-1.37578700
C	-11.42516200	-1.52970500	-1.43167700
H	-10.58073100	-2.15897400	-1.72953900
C	-10.81819800	-0.42196000	-0.56136900
H	-10.45482400	-0.84514100	0.37183600

C	-8.46275000	0.03629000	-0.70390400
C	-7.36076800	0.57714500	-1.57202000
H	-7.67087600	0.54971600	-2.61815300
H	-7.19231600	1.62343600	-1.28815800
C	-6.05123900	-0.18760300	-1.36527900
H	-6.21610800	-1.25773300	-1.55863400
C	-4.96635100	0.32677100	-2.29654400
H	-5.27001100	0.11390300	-3.32707300
H	-4.88225100	1.41418200	-2.18915200
C	-3.59745600	-0.29224600	-2.03214900
H	-3.70352000	-1.37226200	-1.86631900
C	-2.64195500	-0.07691800	-3.20246900
H	-2.71007900	0.96568900	-3.53162000
H	-3.00256100	-0.69644900	-4.02890000
C	-1.18389100	-0.42306300	-2.86334400
H	-1.14904600	-1.05740100	-1.97507600
H	-0.72291000	-1.00274600	-3.66874100
C	-0.31801800	0.81448300	-2.62657400
H	-0.09875500	1.28780500	-3.59061000
C	1.01590900	0.46693300	-1.92805600
H	1.29101700	-0.57077400	-2.14568500
C	2.14839300	1.40039600	-2.33403700
H	1.89320900	2.42888500	-2.06331000
H	2.25985900	1.36359400	-3.42402000
C	3.49301400	1.02864100	-1.71774400
H	3.80738400	0.04574900	-2.09060900

C	4.56570700	2.05864600	-2.06453800
H	4.22567900	3.04661500	-1.74173100
H	4.69664600	2.07919500	-3.15032200
C	5.91523600	1.74982400	-1.42288500
C	7.02706600	2.69995000	-1.85377400
H	6.83440700	3.70287100	-1.46664900
H	7.04785700	2.75547400	-2.94591500
C	8.37864900	2.21069400	-1.36158300
H	8.37626000	2.22139900	-0.26454300
C	8.60874200	0.76463100	-1.82524700
H	8.59887100	0.72673800	-2.91898000
C	7.43797900	-0.10377200	-1.31163500
H	7.41240900	-0.02761100	-0.21743300
C	9.94530400	0.21034900	-1.40095900
C	-11.69986300	0.77621600	-0.27354000
H	-11.18099500	1.47600200	0.38524600
H	-12.61587500	0.43631400	0.21098500
H	-11.95895700	1.30277500	-1.19446200
C	-12.07287600	-0.98419300	-2.70367700
H	-12.97275100	-0.39993400	-2.48822000
H	-12.35345700	-1.80092600	-3.37490300
H	-11.38094400	-0.33030600	-3.23898600
C	-12.78738100	-4.14948600	1.15588900
H	-13.01698900	-4.95000700	0.44379300
H	-13.71727600	-3.63614300	1.40309200
H	-12.38451900	-4.61238700	2.05966000

O	8.28712300	-3.77108300	2.71000100
H	7.42260400	-3.75254500	2.27414400
O	12.33641100	-4.26556500	1.48654700
H	12.53252500	-4.20477200	2.43525200
O	9.13368200	-3.59584300	-0.07403800
O	7.31759200	-2.39892100	0.59742200
O	5.83680600	1.79724000	-0.01104300
H	4.98293000	1.35819500	0.20184300
O	6.23471100	0.43202700	-1.84107800
O	10.50845400	-0.72881100	-1.90927300
O	3.41551300	0.93183900	-0.28515400
H	2.47350800	0.90615800	-0.02676500
O	0.80837400	0.51920100	-0.50914100
H	0.26105400	1.31023600	-0.34414700
O	-0.99156100	1.79502600	-1.85116300
H	-1.63176400	1.33044800	-1.27484000
O	-3.01429100	0.28555300	-0.85514100
H	-3.70751100	0.32524600	-0.17623800
O	-5.59039600	-0.03956800	-0.02115800
H	-6.35873700	-0.21277500	0.55054600
O	-8.26664800	-0.36731300	0.43443200
O	-9.64883200	0.06501900	-1.29179100
O	-13.53592900	-1.75954200	-0.18800100
H	-14.08992000	-1.52557500	-0.94040700
O	9.36887900	3.08613900	-1.86759600
H	10.17269300	2.92708900	-1.35805600

N	10.90247700	-3.35267100	3.70274300
H	10.22957800	-4.02811800	4.05843300
H	10.87041200	-2.55040000	4.32368000
C	-9.41215100	1.44096100	3.11711500
H	-9.82809400	2.45355200	3.13465700
H	-9.73062000	0.95695100	2.18993900
C	-7.88994500	1.46621600	3.19594900
H	-7.51248900	0.43761700	3.14340000
H	-7.56840200	1.86432400	4.16615600
C	-7.26082400	2.28243900	2.07151800
H	-7.59871000	3.32335400	2.12503800
H	-7.60891100	1.89922300	1.11183100
C	-5.74099900	2.23871200	2.09797200
H	-5.37618700	1.21026500	2.19463400
H	-5.33239100	2.77687500	2.95907300
C	-5.10221100	2.80217500	0.85607100
O	-5.67110400	3.15290400	-0.15348700
C	-3.01824000	3.51434000	-0.02322600
H	-2.93653800	2.84819600	-0.88149500
H	-3.52601800	4.42987300	-0.33839500
C	-1.67454300	3.83034600	0.59718400
H	-1.82692400	4.42114300	1.50241200
C	-0.73821800	4.58160500	-0.32335400
H	-0.38800700	3.93789400	-1.13259500
H	-1.24687600	5.44946900	-0.75186200
O	-3.77257900	2.87802600	1.01449100

C	-0.79990700	2.40846700	2.30562400
C	-0.35210800	1.00054200	2.57301000
H	0.37292600	0.68198900	1.82218200
H	0.12804100	0.99767400	3.55344800
C	-1.56603100	0.06058100	2.57283900
H	-2.02124700	0.06742100	1.57605200
H	-2.31792100	0.45589600	3.26715600
C	-1.20637700	-1.36760700	2.97381600
H	-0.56749100	-1.81441300	2.20356500
H	-0.60827200	-1.34344700	3.89290500
C	-2.43818700	-2.23895800	3.19631500
H	-3.06521500	-2.27915300	2.30248100
H	-3.04992200	-1.84741300	4.01545700
O	-1.04991800	2.59606100	0.97909300
O	-1.00018500	3.24598700	3.14577900
O	0.34636200	5.07899700	0.46675000
O	2.60260200	5.65882900	1.11218800
O	2.28563800	4.35714900	-1.10839300
O	1.90530700	3.21619700	1.18952600
C	4.02727600	5.73126900	1.03103800
H	4.36388000	5.38976100	0.04535100
H	4.29039900	6.78599500	1.13697600
C	4.67230400	4.92558200	2.14538000
H	4.21947600	5.23111200	3.09212600
H	5.74101800	5.17797800	2.18525900
N	4.44439300	3.48684300	1.95721300

H	5.02621100	3.12981200	1.19529900
H	4.71634300	2.97582100	2.79186700
P	1.82440300	4.51884500	0.28959300
H	2.86027000	3.15891800	1.55023000
O	10.47923300	0.85192700	-0.33197700
H	11.31937500	0.40283200	-0.14260000
H	-9.84906300	0.88613100	3.95163100
H	-2.16138200	-3.26668500	3.44482700

AmB-Chol

AmB-Chol1

0 1

C	-4.60837300	2.60968700	-0.67585700
C	-3.36317100	2.86117000	-1.50973000
C	-2.24901800	3.64141600	-0.79410000
C	-4.22949000	1.89309600	0.64920100
C	-2.74465300	1.91836900	0.91580600
C	-2.08160100	0.80845500	1.23231000
C	-0.59653000	0.73037000	1.41158300
C	0.12161700	1.92025100	0.77579700
C	1.58372600	1.97165600	1.21072600
C	2.45414600	0.75846800	0.90377200
C	3.89990700	1.28845300	1.01654000

C	3.83708500	2.84531600	1.14469000
C	4.23883800	3.39286900	2.53866000
C	5.44430600	2.66139200	3.16790600
C	6.69605600	2.58590000	2.29230300
C	7.87620200	1.93149100	3.00633600
C	9.12145900	1.74613000	2.13277500
C	9.66035600	3.07279100	1.59550200
C	10.21336500	0.99789400	2.89605300
C	4.55631000	4.89413400	2.47960300
C	2.38445900	3.15974700	0.64566800
C	2.43820000	3.14778800	-0.89462500
C	1.68658100	4.43909100	1.12203700
C	0.20918600	4.46690100	0.70728900
C	-0.58759600	3.23151700	1.16009400
C	-2.07734000	3.26959100	0.71174200
C	-2.81451500	4.31467700	1.57622200
O	-5.26764300	3.85259400	-0.44704300
H	-0.60165600	3.24685200	2.26158400
H	0.07078100	1.79106800	-0.31390600
H	1.57780600	2.10426200	2.30467500
H	-5.28249500	1.96101800	-1.24177500
H	-3.64926300	3.37745600	-2.43116900
H	-2.97636600	1.87922700	-1.79795500
H	-2.43132600	4.71689000	-0.87626900
H	-1.31837800	3.44058500	-1.33408500
H	-4.57238900	0.85481000	0.61945400

H	-4.76370100	2.38970500	1.46804800
H	-2.64581400	-0.11246900	1.35282300
H	-0.22586000	-0.20293700	0.97290600
H	-0.34480700	0.68147000	2.48221000
H	2.24229900	0.40440800	-0.10644900
H	2.25873400	-0.07723500	1.58140200
H	4.48070100	1.01548900	0.13343100
H	4.40696300	0.83182300	1.86565600
H	4.54878500	3.28209600	0.43458100
H	3.39564100	3.25115000	3.22823900
H	5.70048600	3.17962100	4.10066700
H	5.16104700	1.64822100	3.47201700
H	6.97599500	3.59331800	1.97052600
H	6.48143600	2.02179600	1.37679900
H	7.56362300	0.94686800	3.37790200
H	8.14373600	2.52394900	3.89275800
H	8.81691300	1.12439200	1.28200500
H	9.86855100	3.76411000	2.41996600
H	8.95377900	3.55953300	0.91921500
H	10.59476700	2.92606900	1.04580800
H	10.56213600	1.58833700	3.75044000
H	11.08033400	0.79342600	2.25915800
H	9.84368500	0.04290200	3.28131800
H	5.37617000	5.09149400	1.78227000
H	4.86385300	5.26265200	3.46308400
H	3.70831500	5.49290500	2.15508600

H	2.97775800	2.28126700	-1.28332000
H	2.96299400	4.04168800	-1.24687500
H	1.44990700	3.14229800	-1.35618400
H	2.18187800	5.33011400	0.72119800
H	1.74395500	4.50190600	2.21440500
H	0.14713700	4.57127600	-0.37855800
H	-0.24845000	5.37125600	1.11818800
H	-2.29642100	5.27624600	1.53902500
H	-2.85492500	3.99327100	2.62227300
H	-3.82905900	4.47836400	1.21265200
H	-6.09527800	3.62942200	0.00798300
C	-9.44790800	3.39264400	0.01061200
H	-10.40566500	3.06221800	-0.41030400
C	-8.74599200	4.19764600	-1.08271900
H	-7.80673100	4.60627600	-0.68207400
C	-8.39557900	3.31554400	-2.27692000
H	-9.34037700	2.98216800	-2.73761600
C	-8.28898800	1.37419400	-0.88455700
H	-9.20636800	0.93553900	-1.31677100
C	-8.64387300	2.15220100	0.37770200
H	-9.22802600	1.48804400	1.02853100
C	-7.53026500	4.04336400	-3.28136900
H	-6.58388800	4.30414700	-2.79936100
H	-8.03348600	4.95363600	-3.61161200
H	-7.32482300	3.40685800	-4.14488000
C	-7.08374500	-0.61270600	-1.48058000

H	-7.24607700	-0.18764500	-2.47643300
C	-5.63284600	-0.91728200	-1.30082100
H	-5.33559200	-1.25292900	-0.31139900
C	-4.70509900	-0.73819500	-2.24552300
H	-4.99237900	-0.39774200	-3.23965200
C	-3.29530200	-0.90926700	-1.96951800
H	-3.03851100	-1.18619000	-0.95112200
C	-2.29168300	-0.67228300	-2.83457900
H	-2.50970300	-0.37272900	-3.85884900
C	-0.91359600	-0.74603400	-2.41821500
H	-0.75704000	-1.03539900	-1.38064500
C	0.16426300	-0.45923800	-3.17580000
H	0.04331600	-0.15239000	-4.21360300
C	1.49288200	-0.50291900	-2.62222500
H	1.55912500	-0.84323000	-1.59011300
C	2.63373600	-0.13544700	-3.24105900
H	2.60891800	0.20384700	-4.27549700
C	3.89895100	-0.11256000	-2.55041400
H	3.87788100	-0.41449200	-1.50430400
C	5.07921000	0.28455800	-3.06805200
H	5.14036500	0.58091000	-4.11417400
C	6.27974500	0.36274600	-2.27161600
H	6.16437200	0.14759200	-1.21063000
C	7.51131800	0.67653100	-2.71325900
H	7.67956500	0.87588000	-3.77058400
C	-8.02651700	-1.79721000	-1.25720600

H	-9.04591500	-1.45077100	-1.44163200
H	-7.80477300	-2.59127900	-1.97690900
C	8.64633600	0.73114900	-1.81295300
H	8.39917700	0.64089800	-0.75830000
C	9.93262200	0.83660600	-2.16176300
H	10.20262700	0.91151400	-3.21613400
C	11.06690200	0.82721600	-1.17542600
H	10.65477200	0.96685900	-0.17171500
C	11.80997900	-0.52195800	-1.17054700
H	12.31933500	-0.62187200	-2.14338400
C	10.90754000	-1.75983500	-1.01284500
H	10.15049300	-1.68121700	-1.79830200
C	10.12751400	-1.77142900	0.30892900
H	9.72162100	-0.78325700	0.51028300
C	7.76392000	-2.22888500	0.32610600
C	6.72679200	-3.30419500	0.11745200
H	7.12372100	-4.06440600	-0.55792300
H	6.55661100	-3.77905600	1.09081900
C	5.39903900	-2.74799600	-0.39909500
H	5.56508500	-2.27038000	-1.37559800
C	4.36303500	-3.85420300	-0.55182600
H	4.73004500	-4.58031900	-1.28517100
H	4.25332200	-4.37579700	0.40650800
C	2.98495800	-3.36167700	-0.98674300
H	3.08350500	-2.74927100	-1.89340500
C	2.03737100	-4.52361600	-1.28068600

H	2.14300600	-5.27831100	-0.49226000
H	2.36911500	-4.99274700	-2.21162700
C	0.56519900	-4.09182200	-1.39068200
H	0.50334400	-3.02563500	-1.62066100
H	0.07809200	-4.61154000	-2.22135800
C	-0.25631400	-4.38776800	-0.13324300
H	-0.41812900	-5.47169800	-0.06740000
C	-1.63272200	-3.68005000	-0.17422800
H	-1.94264200	-3.51624200	-1.21251600
C	-2.72455100	-4.44562300	0.56931200
H	-2.39429500	-4.62912900	1.59890800
H	-2.86118500	-5.42066300	0.08746100
C	-4.06245400	-3.70687000	0.59150600
H	-4.36452000	-3.47247300	-0.43850300
C	-5.16626200	-4.53713000	1.23871800
H	-4.83985100	-4.88173600	2.22489300
H	-5.35273600	-5.41598000	0.61465200
C	-6.47759900	-3.77196600	1.43035400
C	-7.63046600	-4.66632000	1.86298000
H	-7.42567400	-5.10760900	2.84066800
H	-7.75101700	-5.47415200	1.13580800
C	-8.93460100	-3.88856500	1.93466600
H	-8.85668400	-3.15198400	2.74704900
C	-9.17924500	-3.11981100	0.60693900
H	-9.46622700	-3.87063600	-0.13922000
C	-7.92226900	-2.36510900	0.15196000

H	-7.75048600	-1.54706700	0.85585900
C	-10.32750500	-2.15576000	0.77757900
C	10.86702000	-2.27861400	1.53048900
H	10.22505700	-2.20901000	2.41107300
H	11.75533600	-1.66777100	1.69086100
H	11.16497100	-3.32159400	1.40478800
C	11.68878700	-3.05262900	-1.24401300
H	12.52353000	-3.15942200	-0.54415300
H	12.09535100	-3.08037500	-2.25877700
H	11.04293300	-3.92444800	-1.11609000
C	12.04554200	1.97260400	-1.44579300
H	12.47054300	1.88978100	-2.45235400
H	12.86540000	1.95400700	-0.72662300
H	11.53461800	2.93591500	-1.37704500
O	-7.45842100	2.57380100	1.03967600
H	-6.89783600	1.78829500	1.12411300
O	-9.59638300	5.23154700	-1.52294200
H	-9.96500700	5.60454800	-0.70670200
O	-7.64643300	2.18456300	-1.83796000
O	-7.38620000	0.39540300	-0.49598000
O	-6.35151500	-2.76539400	2.39253700
H	-5.49535600	-2.33100200	2.22261200
O	-6.79857900	-3.23308900	0.15037500
O	-10.28818300	-0.95286200	0.66803400
O	-11.48077000	-2.78451800	1.11635100
H	-12.14768700	-2.08982900	1.24138200

O	-3.94291300	-2.48279200	1.32235200
H	-3.03940100	-2.14827100	1.16740100
O	-1.45573400	-2.38224100	0.40135400
H	-0.75348500	-2.53532400	1.06657800
O	0.38518000	-3.98574400	1.06988700
H	1.17678400	-3.45008900	0.84246900
O	2.38830600	-2.55632100	0.03485200
H	3.09248200	-2.00919900	0.43060700
O	4.86945700	-1.78769700	0.50979800
H	5.58201100	-1.16477900	0.73728400
O	7.48696500	-1.09532400	0.68703200
O	8.99145700	-2.66941000	0.09898900
O	12.77916500	-0.43589700	-0.12859800
H	13.43681900	-1.12766300	-0.25830300
O	-9.94871100	-4.83486100	2.19343600
H	-10.79778600	-4.37542500	2.17835200
N	-9.73369000	4.29903100	1.11764600
H	-8.87914200	4.43570300	1.65240300
H	-10.41602800	3.89401200	1.75040700

AmB-Chol2

0 1

C	4.05152800	-1.51997600	-1.40991100
C	2.98183200	-0.59413600	-1.96720600

C	1.91489600	-1.33196300	-2.78897200
C	3.40684000	-2.60898800	-0.51306900
C	1.90933900	-2.66199100	-0.69889100
C	1.08288400	-2.57045800	0.34188500
C	-0.40670500	-2.44311200	0.22331100
C	-0.82140000	-1.98916200	-1.17597700
C	-2.33265600	-2.03769200	-1.36133200
C	-3.15870200	-1.15236200	-0.43955900
C	-4.51370800	-1.00032500	-1.15908100
C	-4.36506600	-1.57741200	-2.60472900
C	-5.20478400	-2.85772200	-2.84777300
C	-6.63318300	-2.75524800	-2.25853200
C	-7.39624800	-1.48347800	-2.63904000
C	-8.81499100	-1.43546700	-2.07425300
C	-9.52441900	-0.09163300	-2.28062100
C	-9.83959700	0.17562800	-3.75205500
C	-10.79301500	-0.01641400	-1.43157400
C	-5.31459600	-3.19362700	-4.34146600
C	-2.80871500	-1.66415700	-2.77884800
C	-2.35134700	-0.25568700	-3.20625000
C	-2.19718900	-2.69433200	-3.73367800
C	-0.66304600	-2.69600800	-3.64742800
C	-0.10383000	-2.86107700	-2.22135800
C	1.43879800	-2.67716800	-2.14716500
C	2.10824400	-3.85748400	-2.87946700
O	4.76079100	-2.05671800	-2.52463000

H	-0.30496300	-3.90190700	-1.92337400
H	-0.48894200	-0.94775900	-1.28547400
H	-2.64592500	-3.08202400	-1.20197700
H	4.75183500	-0.92898100	-0.80554200
H	3.45500300	0.18140400	-2.57746200
H	2.51249200	-0.08991400	-1.11638700
H	2.30163000	-1.52671700	-3.79273100
H	1.06647000	-0.65188300	-2.90807100
H	3.63305500	-2.40628900	0.53687700
H	3.86972300	-3.57386900	-0.75405400
H	1.50264300	-2.53465600	1.34500100
H	-0.76700700	-1.73216300	0.97437700
H	-0.89451900	-3.40088200	0.45849800
H	-2.67035500	-0.18424800	-0.32447500
H	-3.25937100	-1.56422200	0.56839200
H	-4.82207400	0.04604500	-1.19333400
H	-5.29007500	-1.52274000	-0.60800200
H	-4.74144700	-0.83767100	-3.32086400
H	-4.71589000	-3.70158800	-2.34153800
H	-7.20368500	-3.62632200	-2.60651600
H	-6.59826500	-2.84378600	-1.16865800
H	-7.43072600	-1.38537400	-3.72910300
H	-6.85525900	-0.60515800	-2.27133000
H	-8.76891900	-1.64036100	-0.99831100
H	-9.42499800	-2.23740800	-2.51408200
H	-8.84103300	0.69312300	-1.92997800

H	-10.50972000	-0.59550500	-4.14841300
H	-8.93616700	0.18165200	-4.36616000
H	-10.33348300	1.14374700	-3.88050300
H	-11.48909200	-0.82124400	-1.69094400
H	-11.32717900	0.92802800	-1.59608500
H	-10.55292400	-0.10894800	-0.36769700
H	-5.77510100	-2.36734100	-4.89256400
H	-5.94008000	-4.07889400	-4.49240400
H	-4.35035700	-3.39155100	-4.80421800
H	-2.81437700	0.52699300	-2.60722100
H	-2.64045200	-0.08145200	-4.24761700
H	-1.27201800	-0.11052500	-3.13845900
H	-2.48408000	-2.49379400	-4.77132600
H	-2.57241000	-3.69323600	-3.48287500
H	-0.28488300	-1.77310500	-4.09421400
H	-0.28572000	-3.50477200	-4.27953900
H	1.73547600	-3.93485700	-3.90332700
H	1.89505700	-4.80185100	-2.36837100
H	3.18713600	-3.72133800	-2.94312800
H	5.40793400	-2.68744600	-2.18826500
C	-8.44172800	-2.58884800	2.12440300
H	-9.29976200	-1.91018000	2.21135100
C	-8.11567900	-3.08806500	3.53176700
H	-7.26851300	-3.78782300	3.46036400
C	-7.68050200	-1.92918200	4.42418800
H	-8.52157000	-1.21780400	4.48829800

C	-6.83242400	-0.74264600	2.56741700
H	-7.63024600	0.00889300	2.65581200
C	-7.26152500	-1.80569900	1.56564700
H	-7.53909500	-1.28360300	0.64207500
C	-7.28155700	-2.37997600	5.81090000
H	-6.42594600	-3.05741200	5.74586500
H	-8.11383700	-2.90760600	6.27930000
H	-6.99926300	-1.52292300	6.42628800
C	-5.13388000	0.96482400	2.70745800
H	-5.17902300	0.80924400	3.79096200
C	-3.71197300	1.02324300	2.24846000
H	-3.56564300	1.21071800	1.19008600
C	-2.65008300	0.77801400	3.02095900
H	-2.77582100	0.57791200	4.08432300
C	-1.31317000	0.70016200	2.46783500
H	-1.23038300	0.86940500	1.39702000
C	-0.19651500	0.38398000	3.15031200
H	-0.24722900	0.20353300	4.22346000
C	1.08069700	0.23900700	2.49500200
H	1.08047000	0.39976900	1.41847200
C	2.23932200	-0.09053600	3.10042900
H	2.26171100	-0.26624900	4.17525100
C	3.47173000	-0.24292600	2.36871600
H	3.42971000	-0.03562900	1.29978700
C	4.65233400	-0.62018500	2.90211200
H	4.71779400	-0.82529100	3.96994300

C	5.84717400	-0.79603900	2.11579800
H	5.76166900	-0.59957500	1.04803200
C	7.04457800	-1.18586000	2.59696500
H	7.16072500	-1.36781200	3.66466600
C	8.19893800	-1.38920500	1.75539300
H	8.04517500	-1.26980800	0.68395600
C	9.43449200	-1.69553800	2.19081800
H	9.62097200	-1.79117500	3.25981300
C	-5.96948900	2.19261400	2.32558100
H	-6.99176000	2.03609900	2.67732400
H	-5.57921700	3.07371800	2.84408700
C	10.55980400	-1.86600900	1.29226700
H	10.31524300	-1.88037500	0.23190600
C	11.84093400	-1.94645100	1.66717000
H	12.08766600	-1.91398200	2.72965800
C	13.00474100	-2.02996000	0.72085100
H	12.62737200	-2.21591900	-0.29119300
C	13.78440200	-0.70109900	0.67790800
H	14.28135600	-0.58165000	1.65508000
C	12.91874700	0.55618700	0.47151100
H	12.20509000	0.56676200	1.30096400
C	12.06299500	0.50129100	-0.79883100
H	11.57553600	-0.46674900	-0.87356800
C	9.74691300	1.13284300	-0.81155000
C	8.77879500	2.26479400	-0.57573300
H	9.23051500	2.99546400	0.09780700

H	8.61371800	2.75766600	-1.54095800
C	7.43387600	1.77414300	-0.03506800
H	7.59483400	1.27409000	0.93082800
C	6.46397000	2.93309600	0.15467600
H	6.88654200	3.62765000	0.88860800
H	6.36167700	3.47356400	-0.79406600
C	5.07295000	2.50778100	0.61603500
H	5.16134300	1.87979000	1.51304700
C	4.19130100	3.70915200	0.94173300
H	4.27234800	4.43966000	0.12753500
H	4.59977900	4.18844100	1.83647100
C	2.71630900	3.33371400	1.16274400
H	2.63082000	2.27441500	1.41572500
H	2.31238400	3.88356000	2.01836100
C	1.80901300	3.63916900	-0.02917600
H	1.73958800	4.72953700	-0.14396700
C	0.38429100	3.07215800	0.19085700
H	0.18126000	2.97483900	1.26317300
C	-0.70676900	3.92505300	-0.45541300
H	-0.51074800	3.99760900	-1.53207300
H	-0.65515600	4.93837600	-0.04111000
C	-2.10776800	3.36441800	-0.22488600
H	-2.29351900	3.28702700	0.85500400
C	-3.20168200	4.23433200	-0.84003200
H	-2.97110200	4.42994700	-1.89185400
H	-3.22886500	5.19196200	-0.31194500

C	-4.59126000	3.59143500	-0.78697100
C	-5.71149400	4.52916500	-1.20569500
H	-5.57782300	4.85007500	-2.24091300
H	-5.70369300	5.41370100	-0.56266900
C	-7.05932100	3.84075900	-1.07246300
H	-7.10198200	3.01858500	-1.80067600
C	-7.22576400	3.24002300	0.35130400
H	-7.43034400	4.08795700	1.01674100
C	-5.97328200	2.48044700	0.82644200
H	-5.92415900	1.53619900	0.27732200
C	-8.42153500	2.31790000	0.37888100
C	12.76080800	0.81909800	-2.10553200
H	12.06837300	0.69007900	-2.94054500
H	13.60628800	0.14136400	-2.23092200
H	13.12646800	1.84812200	-2.11719000
C	13.75821200	1.82980000	0.55848400
H	14.54033300	1.85571000	-0.20620400
H	14.23941100	1.90956200	1.53755000
H	13.13374200	2.71469300	0.41699200
C	13.94484900	-3.17923500	1.09501300
H	14.32551800	-3.05001600	2.11430600
H	14.79640800	-3.21238200	0.41414100
H	13.41932300	-4.13600800	1.05397100
O	-6.20974300	-2.72342700	1.32130300
H	-5.38329500	-2.22664000	1.40614600
O	-9.25091600	-3.70428000	4.09477900

H	-9.62836900	-4.21879300	3.36307100
O	-6.55065800	-1.28820400	3.83713900
O	-5.66086800	-0.20157300	2.05220500
O	-4.66695700	2.47109800	-1.62327200
H	-3.83503300	1.98204200	-1.48538300
O	-4.79924500	3.24151100	0.57682500
O	-8.42657200	1.15834300	0.72274900
O	-9.55185800	2.90714200	-0.07537300
H	-10.24303200	2.22380900	-0.07733600
O	-2.21274700	2.05796100	-0.79389700
H	-1.32299300	1.65353300	-0.79644100
O	0.36717200	1.74372900	-0.34448000
H	1.01706000	1.79891400	-1.07686800
O	2.28076700	3.10588800	-1.25795400
H	3.10192200	2.59231700	-1.09272700
O	4.41071300	1.74981700	-0.39960500
H	5.08111600	1.21145500	-0.85877600
O	6.83356600	0.85533800	-0.94368300
H	7.52025900	0.22216200	-1.21863600
O	9.39530600	0.02024300	-1.17145500
O	11.00476500	1.49220500	-0.60456200
O	14.76663600	-0.84829300	-0.34283900
H	15.44713100	-0.17850000	-0.21635900
O	-8.03734900	4.82013600	-1.34521500
H	-8.90346100	4.40172900	-1.26191800
N	-8.85662900	-3.73722900	1.32390200

H	-8.02295100	-4.24619500	1.03874000
H	-9.32341600	-3.43565400	0.47453800

AmB-Chol3

0 1

C	-10.46510100	-2.67603900	1.31907400
C	-9.56843900	-3.15627200	2.44952800
C	-8.28307100	-3.84816800	1.96680800
C	-9.68206600	-1.70167300	0.39658300
C	-8.19703200	-1.75265300	0.66591700
C	-7.51381300	-0.64458300	0.94930600
C	-6.07064300	-0.61386900	1.34799800
C	-5.57396900	-1.97817600	1.83087100
C	-4.04931900	-1.98981900	1.90382100
C	-3.36143500	-0.96130900	2.79542000
C	-1.92366600	-1.51318300	2.95438800
C	-1.89040200	-2.95049800	2.33806900
C	-1.15585800	-3.08187900	0.97540500
C	0.05418200	-2.15298000	0.78019500
C	1.20896800	-2.27801400	1.77273000
C	2.32325400	-1.27673500	1.46252300
C	3.49705300	-1.27671900	2.44899300
C	4.31216800	-2.56844600	2.36792800
C	4.40147900	-0.06932700	2.20328600

C	-0.71457300	-4.53154200	0.73498400
C	-3.41185400	-3.33091600	2.31026900
C	-3.80268300	-3.76200300	3.73529000
C	-3.89164900	-4.39075700	1.31054100
C	-5.42280700	-4.44324900	1.22769700
C	-6.05444800	-3.08712200	0.87405900
C	-7.60287100	-3.15008300	0.74442800
C	-7.95704000	-3.92117800	-0.54623700
O	-10.93396400	-3.83456700	0.63720200
H	-5.66982000	-2.80596700	-0.11595800
H	-5.99147600	-2.16051200	2.83164600
H	-3.70585000	-1.80756300	0.87916300
H	-11.32618000	-2.14404900	1.74987500
H	-10.13720700	-3.82892100	3.09763900
H	-9.30312100	-2.27713300	3.04623900
H	-8.50011400	-4.88883300	1.71141900
H	-7.59228300	-3.87144400	2.81428800
H	-10.03865600	-0.67801100	0.55571200
H	-9.90128700	-1.95984300	-0.64715400
H	-8.03203300	0.31047900	0.89686100
H	-5.91642600	0.15098200	2.11692300
H	-5.45899300	-0.30417200	0.49117800
H	-3.87163900	-0.88982800	3.76180300
H	-3.37747000	0.04191100	2.36067800
H	-1.63943100	-1.55283300	4.00921500
H	-1.20246600	-0.84974100	2.47931900

H	-1.36921100	-3.61732400	3.03534300
H	-1.85149600	-2.80116700	0.17488300
H	0.44359200	-2.34619600	-0.22674800
H	-0.28060700	-1.11159800	0.77155600
H	1.60768100	-3.29691100	1.75166400
H	0.84644000	-2.10870500	2.79408000
H	1.88813200	-0.26893900	1.43130800
H	2.72119900	-1.46723200	0.45509500
H	3.08254900	-1.19450100	3.46327400
H	4.74757500	-2.68385100	1.36800400
H	3.70447000	-3.45356100	2.57096800
H	5.13632200	-2.55889900	3.08752600
H	4.82226800	-0.11460800	1.19512000
H	5.23546000	-0.04068600	2.91127000
H	3.85211700	0.87265000	2.29006300
H	-0.00735300	-4.85535800	1.50492500
H	-0.21657900	-4.63082600	-0.23482800
H	-1.54974700	-5.23014100	0.75198300
H	-3.43457100	-3.06412100	4.49064400
H	-3.36695200	-4.74166000	3.95633700
H	-4.88276600	-3.84355800	3.86981600
H	-3.51463400	-5.38351000	1.58085800
H	-3.50579200	-4.15777700	0.31302300
H	-5.82451400	-4.81059400	2.17634600
H	-5.70049000	-5.18994100	0.47845600
H	-7.44192100	-4.88402800	-0.56856100

H	-7.66846000	-3.36707100	-1.44428400
H	-9.02497200	-4.13198200	-0.59462300
H	-11.49962000	-3.54964200	-0.08961600
C	-7.39270600	4.01385900	2.30623800
H	-8.28247100	4.20751100	1.69385400
C	-6.69770900	5.35697500	2.51968600
H	-5.80583100	5.19053300	3.14406100
C	-6.22418300	5.92210100	1.18558000
H	-7.10860900	6.06019300	0.54024000
C	-5.92178000	3.74321900	0.30228000
H	-6.73083100	3.87402900	-0.43420100
C	-6.48152100	3.05717700	1.54654300
H	-7.04691900	2.18201000	1.20401500
C	-5.48673300	7.23345000	1.33387000
H	-4.59162400	7.08551100	1.94379200
H	-6.13123500	7.96427200	1.82476600
H	-5.18107800	7.61363400	0.35664600
C	-4.37374700	3.20457500	-1.46434000
H	-4.37249600	4.29129900	-1.59830800
C	-2.96673500	2.70160200	-1.39467300
H	-2.85337500	1.63962100	-1.19311800
C	-1.87811800	3.47296900	-1.48167500
H	-1.97514100	4.54076400	-1.67365000
C	-0.55067600	2.93790300	-1.25731800
H	-0.50524300	1.87573300	-1.03500800
C	0.60371100	3.63144200	-1.24056800

H	0.60745800	4.69945500	-1.45445500
C	1.85016700	2.99070600	-0.89318000
H	1.77547600	1.92771200	-0.67714400
C	3.06311800	3.57160200	-0.80242100
H	3.18685200	4.63162700	-1.01968900
C	4.22286300	2.82225700	-0.38501900
H	4.05618100	1.76788700	-0.16565400
C	5.46880900	3.31391400	-0.22560700
H	5.66674200	4.36232200	-0.44499600
C	6.56478300	2.51564300	0.26505300
H	6.33742100	1.48102900	0.51972400
C	7.82670400	2.95299000	0.44883600
H	8.08269300	3.97948300	0.18929300
C	8.87162000	2.12213800	0.99691100
H	8.58073900	1.11997500	1.30932200
C	10.15981900	2.48340500	1.13988500
H	10.48277400	3.47071800	0.81191500
C	-5.24920300	2.54701200	-2.54096000
H	-6.23291100	3.01789500	-2.50964700
H	-4.81973800	2.73091300	-3.53035600
C	11.16452800	1.59175000	1.68573200
H	10.78710600	0.65797500	2.09813800
C	12.48396900	1.80920000	1.66833100
H	12.86526100	2.73774000	1.24024600
C	13.51621100	0.83486100	2.16046300
H	13.01072400	0.01975500	2.69093900

C	14.28977400	0.20085000	0.98795800
H	14.91840500	0.99109100	0.54468000
C	13.40973700	-0.34765900	-0.15031800
H	12.81720500	0.50171400	-0.50389400
C	12.38626800	-1.38593700	0.32240200
H	11.89611100	-1.03544200	1.22634900
C	10.08857100	-1.33947800	-0.36867200
C	9.16143400	-1.40201200	-1.55660600
H	9.68616000	-1.04197700	-2.44384900
H	8.91439000	-2.45711000	-1.72254100
C	7.86820800	-0.62035100	-1.32242900
H	8.11349000	0.43528800	-1.13951700
C	6.93907100	-0.71492300	-2.52483900
H	7.43881000	-0.26411900	-3.38885600
H	6.75714800	-1.77064100	-2.75962700
C	5.59079800	-0.03211100	-2.31279400
H	5.75252100	0.99579500	-1.96118200
C	4.76653700	0.01407400	-3.59421500
H	4.76327100	-0.98315100	-4.05157400
H	5.28116800	0.67855000	-4.29477000
C	3.32373300	0.49537200	-3.36741900
H	3.25781500	1.06149700	-2.43487300
H	3.02984000	1.18543200	-4.16416000
C	2.28332000	-0.62483600	-3.34381500
H	2.19787300	-1.05031000	-4.35366100
C	0.90685100	-0.08714300	-2.90908300

H	0.76182900	0.93352800	-3.28030100
C	-0.25188300	-0.97022400	-3.36684600
H	-0.07776300	-1.99522100	-3.01935500
H	-0.26892100	-0.98962000	-4.46252100
C	-1.60432300	-0.48125900	-2.85419400
H	-1.75706700	0.56172400	-3.16331700
C	-2.76533300	-1.31538800	-3.38453900
H	-2.58666100	-2.37408700	-3.17231600
H	-2.82498400	-1.18794100	-4.46914000
C	-4.10964100	-0.93868700	-2.76083300
C	-5.29587600	-1.63608700	-3.40194700
H	-5.22794600	-2.71627500	-3.25516600
H	-5.30271400	-1.43069000	-4.47613400
C	-6.59918400	-1.14088200	-2.80016000
H	-6.63274800	-1.42757200	-1.74257600
C	-6.68209700	0.40848500	-2.86491500
H	-6.84438100	0.68182900	-3.91538800
C	-5.38260900	1.04235300	-2.33240500
H	-5.34313800	0.83768900	-1.26033100
C	-7.86552400	0.90155000	-2.06196000
C	12.89602500	-2.79624900	0.53785000
H	12.09500500	-3.42753500	0.92930900
H	13.71775300	-2.77146900	1.25490700
H	13.25373800	-3.23355500	-0.39679200
C	14.25712200	-0.84758800	-1.31908200
H	14.92888100	-1.65764500	-1.01943600

H	14.86675200	-0.03707400	-1.72890400
H	13.62194600	-1.22979000	-2.12133400
C	14.49236200	1.50043800	3.13457800
H	14.99956000	2.34539800	2.65565700
H	15.25039200	0.78694600	3.46063500
H	13.96295300	1.88211600	4.01050600
O	-5.43806900	2.66429700	2.41872500
H	-4.69020000	2.42064000	1.85408600
O	-7.59116800	6.26512500	3.12106900
H	-8.05907100	5.73255000	3.78451600
O	-5.32846700	4.99008500	0.58792800
O	-4.92555600	2.89909800	-0.17088300
O	-4.13973900	-1.25627700	-1.39593000
H	-3.27780000	-0.97491600	-1.03322400
O	-4.25125200	0.46112800	-2.96749400
O	-7.85947100	1.76979500	-1.22154100
O	-9.01476200	0.24538600	-2.35668100
H	-9.68789500	0.58443500	-1.74475800
O	-1.64038200	-0.54020100	-1.42493900
H	-0.74162100	-0.32888900	-1.10682600
O	0.93112300	0.00496900	-1.48138600
H	1.49591500	-0.74646900	-1.21520700
O	2.59606800	-1.67945400	-2.44400800
H	3.45775800	-1.49644700	-2.01215600
O	4.82319000	-0.73219100	-1.32855500
H	5.44259100	-1.04265500	-0.64285400

O	7.16942900	-1.14111700	-0.19391600
H	7.80788400	-1.19539900	0.53875400
O	9.69489100	-1.23359400	0.78188200
O	11.36262800	-1.43307800	-0.72057300
O	15.12018000	-0.80697400	1.55716800
H	15.81688800	-1.02497200	0.92891500
O	-7.63024000	-1.77541900	-3.52768300
H	-8.47148400	-1.48748100	-3.15213800
N	-7.84037300	3.52859600	3.60877100
H	-7.03666000	3.12865900	4.08824500
H	-8.51687100	2.77973200	3.49879200

AmB-Chol4

0 1

C	3.29838500	2.14111300	4.58458200
C	2.93272400	3.61669900	4.61838700
C	1.57593600	3.91013700	3.96857200
C	3.20787900	1.59556100	3.15577700
C	1.83080600	1.87262300	2.61011700
C	0.98447700	0.87185400	2.37173500
C	-0.44910000	1.04676200	1.98772400
C	-0.98547000	2.42516500	2.35689100
C	-2.32145500	2.67801800	1.66539700
C	-3.45301900	1.68755900	1.89656300

C	-4.66047500	2.40927800	1.26992700
C	-4.32711500	3.93577300	1.17329500
C	-4.35864900	4.48965100	-0.27200900
C	-5.51706100	3.93662500	-1.12893800
C	-6.91133300	4.00082500	-0.49640300
C	-8.02660200	3.92421000	-1.53957600
C	-9.41447900	3.60576500	-0.97400400
C	-9.84733000	4.59645300	0.10639900
C	-10.44342500	3.55359700	-2.10221100
C	-4.43865100	6.02197800	-0.28722900
C	-2.96355600	4.05081100	1.94596900
C	-3.31601700	4.23630800	3.43382100
C	-1.94961800	5.12530700	1.52920800
C	-0.58815300	4.91320800	2.20473500
C	0.00977400	3.52268000	1.94522600
C	1.44317200	3.34616900	2.53079900
C	2.41334800	4.09398300	1.59295300
O	4.60789900	2.02605600	5.11664400
H	0.11954100	3.41867400	0.85360800
H	-1.12124900	2.45578100	3.44618000
H	-2.10895700	2.66406800	0.58226600
H	2.57711400	1.58350400	5.20466500
H	3.73200500	4.16632000	4.11001200
H	2.93628800	3.96066900	5.65676200
H	1.41873800	4.99252100	3.96294900
H	0.77941000	3.47745300	4.58570900

H	3.40963800	0.51828300	3.14709000
H	3.99744600	2.07455200	2.56616600
H	1.34734300	-0.14894500	2.45601600
H	-1.05405700	0.26389100	2.45534200
H	-0.57082400	0.88438900	0.90788200
H	-3.58941800	1.50911500	2.96856000
H	-3.27175500	0.71550000	1.43076100
H	-5.57729300	2.23869300	1.84034400
H	-4.82962700	1.99386800	0.27653600
H	-5.08461500	4.50091800	1.72812600
H	-3.42972200	4.18906900	-0.77738700
H	-5.53023800	4.50484400	-2.06786700
H	-5.31546400	2.90082700	-1.41593300
H	-7.02171000	4.92021300	0.08746500
H	-7.02518900	3.17892000	0.22081700
H	-7.78694100	3.16156300	-2.28852700
H	-8.07084800	4.87730700	-2.08496400
H	-9.36529800	2.60525600	-0.52065500
H	-9.85495600	5.61817700	-0.29042800
H	-9.17784700	4.57926100	0.97026400
H	-10.85692000	4.37223300	0.46414700
H	-10.57243500	4.54441500	-2.55182900
H	-11.42137000	3.22471200	-1.73726100
H	-10.12027200	2.87313800	-2.89330300
H	-5.39056300	6.36129800	0.13296900
H	-4.37851700	6.40175600	-1.31154300

H	-3.64663500	6.49676300	0.28806000
H	-4.09038600	3.53644800	3.75609200
H	-3.70655200	5.24637000	3.59291800
H	-2.46036300	4.10414600	4.09844600
H	-2.31149800	6.12667600	1.78323200
H	-1.80843300	5.10123900	0.44307900
H	-0.69766000	5.06562700	3.28203600
H	0.09909400	5.69176700	1.85945700
H	2.14616900	5.15162000	1.52656200
H	2.37114700	3.67601400	0.58363000
H	3.44962500	4.04663400	1.92899100
H	4.84149200	1.09063400	5.13095600
C	-8.52090200	-5.67432300	0.75055000
H	-9.37957000	-5.00547200	0.88946300
C	-8.01541600	-6.04976500	2.14292500
H	-7.16251000	-6.73653000	2.02697500
C	-7.51108000	-4.81223500	2.87545000
H	-8.35435400	-4.10733900	2.97429500
C	-6.90166000	-3.78153800	0.83403800
H	-7.68726900	-3.01913600	0.95066000
C	-7.44097900	-4.92397700	-0.02204000
H	-7.86295300	-4.47731100	-0.93481900
C	-6.94449400	-5.13003300	4.24048600
H	-6.08720300	-5.80039200	4.13649200
H	-7.70513000	-5.62235800	4.84839100
H	-6.61223100	-4.21715200	4.73979200

C	-5.18618700	-2.10393300	0.70580300
H	-5.15513000	-2.20266500	1.79571800
C	-3.79246100	-2.08528800	0.16010100
H	-3.69650100	-2.02833700	-0.92041800
C	-2.69295700	-2.16734500	0.91656700
H	-2.78544600	-2.23415600	2.00040200
C	-1.35535600	-2.19539700	0.35844700
H	-1.28200400	-2.11587400	-0.72328300
C	-0.22584400	-2.32277200	1.07946300
H	-0.28912700	-2.40955400	2.16417300
C	1.08786600	-2.33716500	0.48194700
H	1.12232500	-2.22165400	-0.59987800
C	2.24313900	-2.44809500	1.16809700
H	2.21507200	-2.56248000	2.25183500
C	3.54048400	-2.41314600	0.54071200
H	3.56341900	-2.27542900	-0.54008200
C	4.71184500	-2.51747200	1.20116200
H	4.70339200	-2.63958100	2.28404600
C	5.99765300	-2.50053500	0.54992800
H	5.99932800	-2.39790600	-0.53492900
C	7.17918300	-2.60349100	1.19101200
H	7.18975000	-2.68515100	2.27744700
C	8.45340000	-2.62124000	0.51444300
H	8.42840600	-2.60665700	-0.57368900
C	9.65159200	-2.63861700	1.12641400
H	9.69950300	-2.62615700	2.21472400

C	-6.02734300	-0.87835200	0.32786200
H	-7.02106400	-1.00142400	0.76319300
H	-5.58826500	0.01515500	0.77350800
C	10.90556600	-2.62989200	0.39851700
H	10.82405400	-2.75010500	-0.67982300
C	12.11044000	-2.43164600	0.94392800
H	12.18958800	-2.29321800	2.02349400
C	13.39377500	-2.33153200	0.16969400
H	13.20685000	-2.62835700	-0.86865100
C	13.91444900	-0.88110200	0.14066400
H	14.24002700	-0.62971700	1.16383500
C	12.86924200	0.17731200	-0.25795000
H	12.05588100	0.08075100	0.46777900
C	12.22724200	-0.09071900	-1.62369600
H	11.94994100	-1.13832100	-1.70136200
C	9.85693700	0.06283200	-1.96695900
C	8.66347800	0.98344100	-1.92467600
H	8.87159300	1.81790600	-1.25244700
H	8.53192600	1.39190000	-2.93345600
C	7.38220600	0.25247400	-1.51622600
H	7.51179800	-0.16359900	-0.50688800
C	6.18799300	1.19518600	-1.52223900
H	6.37726100	2.00589500	-0.81050100
H	6.09284800	1.64302900	-2.51873300
C	4.86186000	0.52829800	-1.16641100
H	4.96362100	-0.00275300	-0.20993700

C	3.74427600	1.55631600	-1.03838500
H	3.78040900	2.23115600	-1.90287100
H	3.96143000	2.16524500	-0.15573100
C	2.34545100	0.93615100	-0.91464900
H	2.41831100	-0.09461700	-0.55806800
H	1.77127800	1.46797300	-0.15314200
C	1.52514200	0.96889600	-2.19996100
H	1.31496800	2.01766300	-2.45236900
C	0.17688200	0.22519800	-2.01364200
H	-0.06462100	0.16956100	-0.94755600
C	-0.98727900	0.87939100	-2.75068400
H	-0.77103500	0.89388300	-3.82583400
H	-1.07780100	1.91965400	-2.41623900
C	-2.31231300	0.16730800	-2.49354300
H	-2.51200000	0.17027100	-1.41279800
C	-3.48024400	0.84748800	-3.19875500
H	-3.28139800	0.89568500	-4.27386700
H	-3.57242800	1.87026300	-2.82103400
C	-4.81461700	0.12706700	-2.99954700
C	-5.99185100	0.89661700	-3.58392200
H	-5.91795900	0.91509900	-4.67307000
H	-5.97164300	1.92627100	-3.22139300
C	-7.32174000	0.27539300	-3.18905900
H	-7.40253500	-0.71468900	-3.66057300
C	-7.38771500	0.06957800	-1.64996400
H	-7.43166300	1.05975300	-1.18257200

C	-6.13846000	-0.68627600	-1.17911100
H	-6.13507700	-1.66810400	-1.66152400
C	-8.64635800	-0.68072200	-1.30563400
C	13.02848100	0.31322800	-2.84435600
H	12.49811800	0.02072200	-3.75350600
H	13.99698500	-0.18788900	-2.81558700
H	13.18837200	1.39322000	-2.87153100
C	13.43549000	1.59148200	-0.14210700
H	14.30127100	1.73961000	-0.79454000
H	13.74692200	1.79998800	0.88550400
H	12.68410900	2.33286200	-0.42294000
C	14.46524700	-3.26285900	0.74364400
H	14.66326600	-3.02222800	1.79408400
H	15.39776300	-3.15827000	0.18746400
H	14.13731200	-4.30371900	0.69582400
O	-6.41587900	-5.84422000	-0.33431400
H	-5.60937300	-5.32251800	-0.45196300
O	-9.05855000	-6.63775100	2.88543200
H	-9.50529900	-7.21680200	2.24673600
O	-6.47174000	-4.21808800	2.10430600
O	-5.79353000	-3.28542000	0.15466600
O	-4.82208300	-1.14087100	-3.58416000
H	-3.93605800	-1.52333500	-3.44263800
O	-4.98543500	0.04409500	-1.58212800
O	-8.73108600	-1.81841200	-0.90919700
O	-9.75915000	0.06262300	-1.53587700

H	-10.52040300	-0.50067200	-1.31978400
O	-2.25733200	-1.19031600	-2.94069700
H	-1.32533500	-1.48016300	-2.90674000
O	0.35257300	-1.12187100	-2.45580900
H	1.07239500	-1.03846000	-3.11717700
O	2.18146800	0.37821400	-3.31300300
H	3.06602900	0.05971800	-3.03098100
O	4.48055400	-0.42013000	-2.16771500
H	5.29349100	-0.85003600	-2.49007600
O	7.09869300	-0.80642800	-2.42729800
H	7.92647600	-1.30354400	-2.55484700
O	9.78063200	-1.11143200	-2.29321500
O	10.98221400	0.67658200	-1.63274100
O	15.03965200	-0.87907600	-0.73273700
H	15.56294400	-0.08886600	-0.56134400
O	-8.32662400	1.14547200	-3.66920500
H	-9.17767200	0.85846000	-3.31393900
N	-9.00046700	-6.89538600	0.10923000
H	-8.19148900	-7.40879300	-0.23394900
H	-9.57259300	-6.67470800	-0.69966200

AmB-Chol5

0 1

C	10.49545700	3.22320800	0.17215000

H	11.34832000	2.75251300	0.67726600
C	9.86952600	4.19266100	1.17329400
H	9.03351000	4.71083600	0.68181300
C	9.31397100	3.43305900	2.37173900
H	10.15075600	2.90266000	2.85560000
C	8.88626100	1.52428000	1.04440200
H	9.66816700	0.95035800	1.57156000
C	9.48805900	2.14297200	-0.21630000
H	9.98036900	1.33603700	-0.77666600
C	8.63371700	4.34502000	3.36811000
H	7.77920300	4.83021700	2.89176300
H	9.33608200	5.10861900	3.70255300
H	8.27800900	3.77397900	4.22660600
C	7.35643100	-0.20914100	1.63069400
H	7.42163300	0.27600600	2.60996400
C	5.91745900	-0.46751900	1.30745200
H	5.69480100	-0.77131100	0.28613900
C	4.92540500	-0.35054000	2.19728600
H	5.14160100	-0.04581600	3.21972800
C	3.53936500	-0.57789500	1.83869400
H	3.35072100	-0.85035800	0.80110600
C	2.47453600	-0.41645400	2.64999400
H	2.61299700	-0.12235800	3.68838100
C	1.13159600	-0.55556500	2.13561900
H	1.06798800	-0.85174700	1.09073000
C	-0.01662400	-0.29435900	2.79213600

H	-0.00222200	0.00943100	3.83691300
C	-1.29394600	-0.37624200	2.12409600
H	-1.27616700	-0.63560200	1.06374800
C	-2.49179200	-0.14987200	2.70138600
H	-2.54183700	0.09742000	3.75995900
C	-3.73324700	-0.21973100	1.97129200
H	-3.66470000	-0.40798900	0.89834700
C	-4.95452100	-0.07672100	2.52576100
H	-5.03830000	0.08737400	3.59852800
C	-6.17463100	-0.10752400	1.75627200
H	-6.07370500	-0.16016400	0.67263800
C	-7.41260700	-0.03886300	2.28116800
H	-7.54027700	0.01874000	3.36086700
C	8.23087300	-1.47408300	1.62099600
H	9.22920100	-1.20435500	1.97246900
H	7.81760700	-2.20741600	2.31897000
C	-8.60413100	-0.02212700	1.45579500
H	-8.42779800	0.00752000	0.38171300
C	-9.85781500	-0.05290700	1.92401100
H	-10.02187700	-0.08288700	3.00240800
C	-11.08712500	-0.07886300	1.06218000
H	-10.79418100	0.08594700	0.01938900
C	-11.78792600	-1.44946100	1.13296900
H	-12.24502400	-1.53585800	2.13157600
C	-10.85167800	-2.65992500	0.96809200
H	-10.12562000	-2.58966800	1.78347700

C	-10.02803500	-2.59689600	-0.32217200
H	-9.68267200	-1.58079500	-0.48822100
C	-7.67659000	-2.92399900	-0.50750400
C	-6.51689900	-3.85588000	-0.24722600
H	-6.78855300	-4.56503100	0.53503700
H	-6.34811500	-4.41630700	-1.17377100
C	-5.23169100	-3.10530500	0.12037900
H	-5.39009500	-2.57016200	1.06682900
C	-4.06304600	-4.07154800	0.27424200
H	-4.29552500	-4.78006000	1.07565700
H	-3.94639500	-4.63944200	-0.65645400
C	-2.73330200	-3.38910200	0.59239700
H	-2.86277300	-2.72369000	1.45730200
C	-1.64328500	-4.40798200	0.91424400
H	-1.64392500	-5.17902000	0.13426100
H	-1.90898000	-4.90123400	1.85207200
C	-0.24657900	-3.76789000	1.01940000
H	-0.34269100	-2.68460600	1.12390900
H	0.26919800	-4.12034500	1.91611600
C	0.65600800	-4.06782900	-0.17588000
H	0.88508600	-5.14097800	-0.18411600
C	1.98691600	-3.28496600	-0.09322300
H	2.24490400	-3.10820500	0.95814100
C	3.13600400	-4.01191500	-0.78237400
H	2.88017900	-4.17876500	-1.83581900
H	3.25758500	-4.99320100	-0.31064200

C	4.46452400	-3.26390800	-0.68905700
H	4.66865800	-3.01777100	0.36261300
C	5.60799000	-4.11973900	-1.21705400
H	5.38792000	-4.43491300	-2.24185500
H	5.68422400	-5.01260600	-0.59112000
C	6.96446200	-3.41578300	-1.23050300
C	8.10058100	-4.39243700	-1.50454000
H	8.01690600	-4.78884400	-2.51798300
H	8.03773400	-5.22389600	-0.79762400
C	9.46160000	-3.74098600	-1.33411400
H	9.60146800	-2.98765500	-2.12214000
C	9.53822600	-3.01667100	0.03860900
H	9.56260500	-3.79571700	0.80957700
C	8.31940500	-2.10612200	0.23697100
H	8.34613900	-1.31677600	-0.52026900
C	10.87146000	-2.31147800	0.07247700
C	-10.70095600	-3.11004900	-1.58121600
H	-10.04501900	-2.95329000	-2.43949100
H	-11.63119700	-2.56342900	-1.74054300
H	-10.92115900	-4.17588800	-1.49969300
C	-11.61032500	-3.97685200	1.12253200
H	-12.38485700	-4.09259000	0.35943400
H	-12.08616700	-4.03332700	2.10475000
H	-10.93053900	-4.82611100	1.02934500
C	-12.06629700	1.02951400	1.46091500
H	-12.35762200	0.92483700	2.51045200

H	-12.96475100	0.98284800	0.84533200
H	-11.60163900	2.01058300	1.34120200
O	8.49517100	2.75754200	-1.00574600
H	7.71588100	2.18850500	-0.99333800
O	10.84404400	5.10280100	1.62781600
H	11.32419800	5.36746600	0.83245800
O	8.34974100	2.49006600	1.91558600
O	7.84528600	0.70258200	0.63479200
O	7.02804100	-2.40993200	-2.20190600
H	6.19565200	-1.91558000	-2.15380600
O	7.13315100	-2.87217000	0.07438500
O	11.93378600	-2.87498600	-0.00543700
O	10.80338600	-0.96723500	0.12650300
H	11.71582000	-0.64679800	0.08277900
O	4.44008900	-2.05050100	-1.44476200
H	3.56977300	-1.64372300	-1.32397400
O	1.79447900	-1.99454300	-0.67487300
H	1.16022300	-2.14789400	-1.39433200
O	0.05517400	-3.74750900	-1.42168100
H	-0.81670800	-3.34666800	-1.25947100
O	-2.27047200	-2.61076900	-0.51390000
H	-3.03930500	-2.17859300	-0.91483500
O	-4.87435900	-2.16742600	-0.88950500
H	-5.67381900	-1.69030700	-1.16012900
O	-7.55278800	-1.85738700	-1.07184500
O	-8.83626000	-3.40564600	-0.08286600

O	-12.81085800	-1.42176500	0.14362600
H	-13.43894200	-2.12335500	0.33077300
O	10.41697600	-4.77013200	-1.43718200
H	11.27200400	-4.41749800	-1.16477800
N	11.00963300	4.00994400	-0.94683900
H	10.22674700	4.24152900	-1.55379000
H	11.66104600	3.46672400	-1.50245700
C	-9.03738900	3.83489500	-1.55185100
C	-7.81973800	4.54918300	-2.11946100
C	-6.67437700	4.67549600	-1.10223200
C	-8.65255400	2.41185400	-1.05871800
C	-7.15257600	2.24512600	-0.99082700
C	-6.53829400	1.25816900	-1.64631200
C	-5.04566400	1.15114500	-1.79056200
C	-4.34853100	2.47653600	-1.48251900
C	-2.83670700	2.29499600	-1.41982000
C	-2.14250500	1.83240300	-2.70087200
C	-0.67030700	2.28287600	-2.52838900
C	-0.57792900	3.12351400	-1.21310800
C	0.09618500	2.38448700	-0.03095500
C	1.32870300	1.55406500	-0.44161200
C	2.40728900	2.28279600	-1.24490500
C	3.60989500	1.36878700	-1.48280900
C	4.78802800	2.00579900	-2.22533700
C	5.36415300	3.19227900	-1.44997300
C	5.87564500	0.95757800	-2.47203800

C	0.52430700	3.35787800	1.07672000
C	-2.06624400	3.56270600	-1.00490400
C	-2.31968800	4.73262200	-1.97383700
C	-2.55266900	3.94381200	0.39665800
C	-4.07067400	4.18981900	0.42059900
C	-4.90184600	3.02901000	-0.15628200
C	-6.41301400	3.36984500	-0.27867800
C	-6.99678600	3.54210500	1.13755500
O	-9.54895200	4.65740700	-0.50683700
H	-4.83006600	2.19954200	0.56539900
H	-4.58884500	3.17300900	-2.29761400
H	-2.63654200	1.54792100	-0.63388000
H	-9.79886500	3.74667100	-2.33623500
H	-8.11887700	5.53893700	-2.47157200
H	-7.47725200	3.97671500	-2.98705100
H	-6.89531600	5.49380200	-0.41227000
H	-5.77387800	4.95983900	-1.65348900
H	-9.06491600	1.65637100	-1.73381400
H	-9.10945100	2.25319000	-0.07449700
H	-7.14448700	0.50366900	-2.14381700
H	-4.80320000	0.82358800	-2.80741500
H	-4.64093300	0.37577600	-1.12288700
H	-2.60411900	2.31314300	-3.56961400
H	-2.22769000	0.75403800	-2.85212300
H	-0.35291700	2.88637500	-3.38161000
H	-0.00442400	1.42307000	-2.50432900

H	0.03041700	4.01583600	-1.40474400
H	-0.62308000	1.67821300	0.40458700
H	1.80088600	1.20154800	0.47840200
H	1.02840200	0.64721600	-0.97730800
H	2.72479500	3.17231100	-0.69371100
H	2.01528500	2.63179200	-2.20785200
H	3.27307700	0.48741500	-2.04506700
H	3.97767100	1.00203500	-0.51431000
H	4.42833300	2.36780900	-3.19696700
H	5.67237400	2.87590900	-0.44542700
H	4.63860000	3.99928400	-1.33595800
H	6.23977600	3.60742400	-1.95526200
H	6.24733700	0.56584000	-1.51672400
H	6.72355200	1.38071500	-3.01851800
H	5.48803100	0.11346400	-3.04895600
H	1.23404000	4.09499200	0.69011300
H	1.01761300	2.81034500	1.88519400
H	-0.31061300	3.90479600	1.50802600
H	-1.94717800	4.51296400	-2.97683400
H	-1.79188600	5.62088600	-1.61425300
H	-3.37536600	4.99007500	-2.06634400
H	-2.04699200	4.84538100	0.75660700
H	-2.31726500	3.13200700	1.09548300
H	-4.29519900	5.11895200	-0.11001800
H	-4.36536300	4.35853000	1.45880500
H	-6.46573300	4.33434600	1.66862500

H	-6.90281900	2.61511100	1.70871700
H	-8.04445800	3.83665500	1.09631800
H	-10.33772500	4.23791600	-0.15495200

AmB-Chol6

0 1

C	-9.31002400	-3.11908500	1.22658300
H	-10.03539300	-2.79499500	1.98270600
C	-8.26191100	-3.96135100	1.95273300
H	-7.53773000	-4.33658700	1.21451800
C	-7.50596300	-3.11238900	2.96825100
H	-8.23389900	-2.74961800	3.71259800
C	-7.84736700	-1.15813600	1.67818800
H	-8.54276900	-0.76346200	2.44518000
C	-8.65366000	-1.88749600	0.60604900
H	-9.41960800	-1.18832500	0.23841100
C	-6.40337800	-3.88755500	3.65451200
H	-5.67390200	-4.21456600	2.91029100
H	-6.82639300	-4.76325200	4.14704900
H	-5.89686900	-3.26120500	4.38978300
C	-6.54156800	0.80244900	1.96194600
H	-6.50964200	0.36169900	2.96360900
C	-5.14028400	0.99920100	1.48000800
H	-5.02815400	1.20646700	0.41964100

C	-4.06070800	0.91161300	2.26114600
H	-4.17144100	0.72567900	3.32804100
C	-2.71314800	0.98771900	1.73230600
H	-2.61561200	1.01719700	0.64652300
C	-1.59409400	0.95777200	2.48069900
H	-1.67592700	0.93328600	3.56594700
C	-0.27085300	0.89825800	1.90707800
H	-0.21978000	0.83447000	0.81990800
C	0.87016700	0.85597300	2.62667200
H	0.81844600	0.92729500	3.71192000
C	2.17188800	0.65889700	2.03603400
H	2.21843600	0.50112300	0.95861600
C	3.32351600	0.62579100	2.73904700
H	3.29889300	0.79361700	3.81444000
C	4.60921500	0.35126000	2.14418500
H	4.62300500	0.10892100	1.08068900
C	5.77093700	0.36000500	2.82970400
H	5.75896700	0.61891400	3.88715000
C	7.05245000	0.04167200	2.24476700
H	7.05194300	-0.31182700	1.21417800
C	8.22927200	0.16887500	2.88742400
H	8.23980300	0.54548500	3.90882900
C	-7.39770600	2.07158300	1.99081800
H	-8.38274100	1.79674400	2.37585500
H	-6.95326200	2.80119400	2.67444800
C	9.50719800	-0.13866600	2.27228100

H	9.45897500	-0.63904000	1.30794100
C	10.69704400	0.18838000	2.78988800
H	10.73332800	0.70135200	3.75263500
C	12.02107700	-0.06258500	2.12569300
H	11.86795600	-0.70868100	1.25445000
C	12.64345100	1.25539300	1.62735900
H	12.91917900	1.84395200	2.51685700
C	11.70319200	2.13937900	0.78728900
H	10.85751700	2.38329400	1.43754100
C	11.09779600	1.40073300	-0.41095800
H	10.75984000	0.41591200	-0.10131200
C	8.77514100	1.48380400	-0.96757400
C	7.62299600	2.37543800	-1.36204800
H	7.84498800	3.40297500	-1.07258700
H	7.54324100	2.33697500	-2.45426200
C	6.29723100	1.90718700	-0.75316300
H	6.37933200	1.92977600	0.34316300
C	5.15245700	2.81264200	-1.19337000
H	5.38834300	3.83956000	-0.89602100
H	5.08375900	2.78297500	-2.28753700
C	3.79314500	2.42953500	-0.60772900
H	3.87558100	2.39850000	0.49004300
C	2.71777200	3.44310600	-0.99533700
H	2.72136300	3.48051300	-2.09230300
H	2.99778100	4.43535800	-0.62477900
C	1.34121900	3.03497400	-0.47350200

H	1.16673000	1.99435000	-0.75116000
H	1.33003900	3.08643700	0.62081300
C	0.17979100	3.86221100	-1.01649800
H	0.25071200	4.90448800	-0.67592300
C	-1.17303900	3.28907000	-0.58089800
H	-1.17265100	3.13961500	0.50391600
C	-2.33887000	4.19089800	-0.98504000
H	-2.28368100	4.38679200	-2.06083000
H	-2.22705200	5.14920400	-0.46574500
C	-3.71592200	3.61169900	-0.65649500
H	-3.76687400	3.34352500	0.40772100
C	-4.81538700	4.62363700	-0.96253600
H	-4.68418800	5.01296800	-1.97696200
H	-4.72383900	5.45652600	-0.26018400
C	-6.23499900	4.05941300	-0.87299300
C	-7.29054000	5.15453700	-0.93172400
H	-7.24747400	5.67122700	-1.89235700
H	-7.10816000	5.87923600	-0.13391900
C	-8.67978500	4.57310500	-0.74188400
H	-8.92040100	3.93926900	-1.60532600
C	-8.72393400	3.69211700	0.53600400
H	-8.69383500	4.38469200	1.38650000
C	-7.53853800	2.71721000	0.61791800
H	-7.65749200	1.94098000	-0.14348500
C	-10.07973300	3.02456100	0.55754800
C	11.97454800	1.27240400	-1.64239400

H	11.45935700	0.68301600	-2.40330300
H	12.90544700	0.77255800	-1.37274500
H	12.20413800	2.25386900	-2.06119300
C	12.38343000	3.44798300	0.38712900
H	13.27246800	3.27424000	-0.22540500
H	12.68461900	4.01033500	1.27455200
H	11.70435800	4.07452500	-0.19419500
C	12.98946900	-0.76876800	3.08002900
H	13.14967300	-0.16306100	3.97758700
H	13.95239700	-0.92772200	2.59523700
H	12.58352500	-1.73216700	3.39210900
O	-7.83889600	-2.33446300	-0.44732100
H	-7.37260100	-1.58296100	-0.85392000
O	-8.89241100	-5.02131600	2.63390600
H	-9.54169500	-5.36529100	2.00657800
O	-6.90997100	-2.00415800	2.30374000
O	-7.15753400	-0.13234000	1.06160300
O	-6.51932600	3.16709300	-1.91206100
H	-5.77031800	2.55608300	-1.97818900
O	-6.32108900	3.41811500	0.39406900
O	-11.12490700	3.62125700	0.47611600
O	-10.05361100	1.68243000	0.62249300
H	-10.97550200	1.39133700	0.58290700
O	-3.97544100	2.44325100	-1.43587400
H	-3.16305600	1.91801500	-1.45589500
O	-1.31215900	1.98239200	-1.15613100

H	-0.90260700	2.02866700	-2.03224500
O	0.12558700	3.81846500	-2.44193800
H	0.98708500	4.04389500	-2.79757400
O	3.35276300	1.16689300	-1.08632800
H	4.12989100	0.60153100	-1.19497000
O	5.98293900	0.58269400	-1.17157200
H	6.77071400	0.03201800	-1.05308400
O	8.66845800	0.28194400	-0.84375700
O	9.90500600	2.15331000	-0.78765600
O	13.82062400	0.89717400	0.91282200
H	14.36952100	1.67843800	0.81383700
O	-9.57082600	5.66060600	-0.64626000
H	-10.43689800	5.30778400	-0.41108900
N	-10.00282800	-3.98956300	0.27895800
H	-9.41934200	-4.07651900	-0.54963300
H	-10.87847300	-3.57402000	-0.01863300
C	-5.24881700	-1.42747900	-2.62650200
C	-4.28283800	-0.89024400	-1.56306300
C	-4.18905700	-1.85747200	-0.38615600
C	-5.34361400	-2.96752400	-2.56898300
C	-3.96889500	-3.48582700	-2.24364700
C	-3.18743800	-4.02919200	-3.17592900
C	-1.71694500	-4.24723900	-2.95912300
C	-1.18921800	-3.14955700	-2.03367700
C	0.31134600	-3.26841700	-1.78654900
C	1.24666300	-3.21734200	-3.00190600

C	2.59193500	-2.67616400	-2.44383900
C	2.39253700	-2.40986700	-0.91989200
C	2.93973400	-3.50639300	0.03022000
C	4.24656800	-4.15841500	-0.45598700
C	5.40527900	-3.18550000	-0.70696100
C	6.55213200	-3.85441800	-1.46450500
C	7.80620400	-2.98798700	-1.62923800
C	8.55851200	-2.82548000	-0.30799300
C	8.73671200	-3.58436600	-2.68555400
C	3.16678700	-2.93756900	1.43684400
C	0.85147300	-2.17301200	-0.84849800
C	0.59681500	-0.76578500	-1.42168700
C	0.13865800	-2.31886500	0.50073700
C	-1.38445900	-2.21617300	0.33346600
C	-1.94279800	-3.21883000	-0.68830900
C	-3.51445100	-3.19254100	-0.81600300
C	-4.07089300	-4.30008400	0.10115600
O	-6.52432300	-0.81951000	-2.37637700
H	-1.69440300	-4.21709700	-0.29632600
H	-1.39126900	-2.18830700	-2.52513800
H	0.47592400	-4.23057600	-1.27806100
H	-4.90612000	-1.12464200	-3.62218600
H	-4.65057800	0.08982000	-1.25958600
H	-3.29033600	-0.74981300	-2.00775100
H	-5.20171400	-2.06628600	-0.02296500
H	-3.67725500	-1.40580900	0.46208100

H	-5.70267000	-3.36744700	-3.52112500
H	-6.06602700	-3.23631400	-1.79193800
H	-3.60309500	-4.23093300	-4.16041400
H	-1.19393300	-4.22036000	-3.91949800
H	-1.50662700	-5.23060300	-2.51561100
H	0.84192900	-2.54781700	-3.76747200
H	1.36257200	-4.19774000	-3.46845500
H	2.85586700	-1.73887500	-2.93873300
H	3.41276100	-3.36140700	-2.64640200
H	2.90407600	-1.47745300	-0.64955700
H	2.19951200	-4.31283300	0.10161500
H	4.56462400	-4.88997900	0.29605000
H	4.05962800	-4.73758000	-1.36550700
H	5.76616700	-2.79145200	0.24909900
H	5.05725500	-2.32180700	-1.28714400
H	6.18471700	-4.14573000	-2.45631100
H	6.83403800	-4.78693900	-0.95667200
H	7.49322000	-1.99390000	-1.97575100
H	8.89087000	-3.80299100	0.05661800
H	7.94037900	-2.37143800	0.47048200
H	9.43825700	-2.19180700	-0.44364300
H	9.05199600	-4.58979100	-2.38810600
H	9.63546800	-2.97476800	-2.80905300
H	8.24022100	-3.66189000	-3.65578900
H	3.93433400	-2.15971600	1.41379600
H	3.50792900	-3.71991000	2.12073300

H	2.27081800	-2.48681500	1.85989000
H	0.97210300	-0.66570400	-2.44289000
H	1.13935400	-0.03412500	-0.82207200
H	-0.45601200	-0.48498700	-1.42752600
H	0.47090900	-1.55589500	1.20915800
H	0.37964000	-3.29496800	0.93769100
H	-1.64690900	-1.19566800	0.04146700
H	-1.86124100	-2.37518800	1.30674100
H	-3.76842200	-4.10686500	1.13572500
H	-3.69222600	-5.28096700	-0.19605000
H	-5.16387600	-4.32271300	0.07225500
H	-7.13273400	-1.10748600	-3.06352000

AmB-Chol7

0 1

C	-14.29847200	-5.19284200	1.79104400
C	-13.29441600	-5.05134100	0.65846000
C	-13.31051400	-3.66205700	0.00254600
C	-14.02137200	-4.12837300	2.88621800
C	-13.08969200	-3.04767800	2.39155200
C	-11.99467900	-2.72041500	3.07516900
C	-10.94415200	-1.77210800	2.58111600
C	-11.02224900	-1.57702500	1.06776100
C	-10.15769900	-0.41335000	0.61027400

C	-8.68644200	-0.33137000	0.98967200
C	-8.28817700	0.94622900	0.24810800
C	-8.99780200	0.86934100	-1.12708400
C	-9.33228100	2.23810200	-1.75043300
C	-8.20449600	3.27649100	-1.58245100
C	-6.84802400	2.92550100	-2.19672000
C	-5.86201700	4.08996000	-2.10221300
C	-4.40333200	3.75899300	-2.44280300
C	-4.25328900	3.03634600	-3.78209100
C	-3.55704300	5.03392400	-2.43401200
C	-9.68770400	2.10450500	-3.23593500
C	-10.17811900	-0.16858900	-0.91619800
C	-9.81614000	-1.42858900	-1.72142700
C	-11.61953700	0.25322100	-1.23962900
C	-12.63511600	-0.81198600	-0.78698700
C	-12.48413700	-1.29388900	0.66962400
C	-13.42611800	-2.48038500	1.01937100
C	-14.87728500	-1.95769600	1.03868400
O	-15.59556700	-5.07167000	1.21347200
H	-12.79889100	-0.45725500	1.31302000
H	-10.68308300	-2.50538100	0.59163800
H	-10.61668600	0.48355300	1.06005600
H	-14.19829100	-6.19438600	2.23382100
H	-13.48543400	-5.82228900	-0.09314400
H	-12.30129200	-5.24401200	1.07774500
H	-14.13715200	-3.60600200	-0.71062100

H	-12.38864200	-3.56725600	-0.57843500
H	-13.57940000	-4.60656600	3.76504500
H	-14.98066400	-3.70467400	3.20743900
H	-11.82125900	-3.18866900	4.04295300
H	-9.95328200	-2.14762700	2.86046900
H	-11.04411400	-0.79686000	3.08136900
H	-8.12759900	-1.19812900	0.61739100
H	-8.51536200	-0.26586700	2.06763600
H	-7.20936700	1.07740200	0.15435700
H	-8.67892300	1.80245300	0.81702600
H	-8.29668500	0.39186500	-1.81912300
H	-10.20483100	2.65025400	-1.22671800
H	-8.55144000	4.22162500	-2.01950400
H	-8.05776900	3.49151600	-0.51638000
H	-6.97582900	2.65422100	-3.24973600
H	-6.42411600	2.05373900	-1.68988500
H	-5.88904400	4.49726700	-1.08134000
H	-6.20731400	4.90354800	-2.75535100
H	-4.02594000	3.09119800	-1.65803800
H	-4.70672700	3.62265700	-4.58978900
H	-4.71455800	2.04602200	-3.77968700
H	-3.19642600	2.89532700	-4.02541500
H	-3.76808700	5.64024300	-3.32191900
H	-2.48948700	4.80234800	-2.41292300
H	-3.77357300	5.65515400	-1.55913400
H	-8.86626800	1.64914700	-3.79735600

H	-9.89035700	3.08385800	-3.68018500
H	-10.56521200	1.47984600	-3.39672000
H	-8.84764000	-1.83958100	-1.42816600
H	-9.75064300	-1.17247200	-2.78441400
H	-10.54987000	-2.22961300	-1.62250000
H	-11.76758800	0.41756400	-2.30995500
H	-11.84297400	1.20333900	-0.74046900
H	-12.56907300	-1.66457600	-1.46682200
H	-13.63927600	-0.40291300	-0.92878300
H	-15.12453600	-1.47847700	0.08874100
H	-15.01315000	-1.21991300	1.83612400
H	-15.58743900	-2.77137900	1.17901700
H	-16.24722600	-5.16952000	1.91724500
C	-5.70442600	-0.93279800	-2.18722800
H	-6.39702800	-0.61726600	-1.39775000
C	-5.55629400	-2.44795300	-2.07200300
H	-4.87644400	-2.79357400	-2.86545000
C	-4.92949200	-2.82127600	-0.73200300
H	-5.60438300	-2.46456500	0.06607900
C	-3.77010800	-0.77103900	-0.63876500
H	-4.40564000	-0.44240400	0.19598700
C	-4.36589100	-0.25281100	-1.94165000
H	-4.50420700	0.82782000	-1.82583500
C	-4.70459100	-4.30904500	-0.58647200
H	-4.01098000	-4.65425000	-1.35768500
H	-5.65213200	-4.83770300	-0.70187500

H	-4.27595800	-4.53703200	0.39184400
C	-1.82844000	-0.32755200	0.70863600
H	-1.98260600	-1.33577600	1.10954700
C	-0.37827000	-0.11643700	0.40763600
H	-0.12118900	0.82277100	-0.07535000
C	0.58029600	-1.00810700	0.67495300
H	0.32537800	-1.95157600	1.15678400
C	1.96919300	-0.79170000	0.32282900
H	2.20018700	0.14847700	-0.17185900
C	2.96823400	-1.66827900	0.53776100
H	2.75641000	-2.61832100	1.02760000
C	4.32790000	-1.40790100	0.12915200
H	4.50137700	-0.44571100	-0.34886400
C	5.36906800	-2.24629900	0.30378000
H	5.21251200	-3.21169900	0.78397600
C	6.70748600	-1.92387000	-0.12511700
H	6.85381100	-0.94505100	-0.58162200
C	7.77812300	-2.73263400	0.01168200
H	7.65439900	-3.70857900	0.47982800
C	9.09823100	-2.38367100	-0.45047400
H	9.20945300	-1.41276600	-0.93298200
C	10.18683400	-3.16916600	-0.32446800
H	10.09298300	-4.13312500	0.17440400
C	11.49050600	-2.80693300	-0.82543900
H	11.55798000	-1.87121100	-1.37777800
C	12.61421000	-3.52520400	-0.64643800

H	12.57466200	-4.45148200	-0.07436800
C	-2.41901300	0.70399800	1.68333500
H	-3.46127700	0.44025300	1.87480700
H	-1.88715300	0.64544500	2.63760400
C	13.90629300	-3.09978900	-1.14828000
H	13.89358800	-2.22929800	-1.80108400
C	15.07915500	-3.65472800	-0.82420100
H	15.09310700	-4.51714100	-0.15565100
C	16.41914200	-3.14744000	-1.27542500
H	16.26866500	-2.37993200	-2.04326600
C	17.18277600	-2.48319900	-0.11301400
H	17.46374500	-3.28234500	0.59303600
C	16.37069000	-1.45290100	0.69364600
H	15.49752600	-1.99295700	1.07245000
C	15.80328900	-0.32045800	-0.16922000
H	15.35695100	-0.73207000	-1.07014800
C	13.53082600	0.42275900	0.03627300
C	12.51381900	1.03262500	0.96798400
H	12.80364700	0.83481100	2.00165800
H	12.54226200	2.11778500	0.81500500
C	11.09577300	0.53022500	0.68621400
H	11.06396900	-0.55879900	0.83347000
C	10.08463300	1.19298000	1.61057400
H	10.35017100	0.95841200	2.64697600
H	10.15062900	2.28077900	1.48831700
C	8.63955200	0.76713900	1.36291700

H	8.56991100	-0.32875200	1.39545400
C	7.70153700	1.35196200	2.41368100
H	7.92083900	2.42076800	2.52776600
H	7.94447400	0.87915700	3.37016600
C	6.21149800	1.15752800	2.08828000
H	6.08009000	0.33285000	1.38369600
H	5.66541600	0.87262200	2.99291000
C	5.53143300	2.40919600	1.53659600
H	5.52683500	3.17443700	2.32535400
C	4.06503100	2.12527000	1.12014700
H	3.69358800	1.24090200	1.65061800
C	3.13112500	3.29976900	1.39026200
H	3.48739300	4.17820400	0.83855600
H	3.16890100	3.54206600	2.45865000
C	1.68577700	2.99913700	1.00493000
H	1.36120600	2.08627000	1.52271500
C	0.74334100	4.12966600	1.39604800
H	1.07083100	5.06290200	0.92756600
H	0.78473100	4.25808600	2.48146400
C	-0.70828100	3.88247000	0.98548800
C	-1.66398700	4.90748800	1.57880600
H	-1.45722900	5.89876500	1.17001400
H	-1.52299800	4.94330700	2.66273700
C	-3.11299800	4.55059300	1.30063900
H	-3.28965000	4.62768800	0.22104700
C	-3.40135600	3.08445600	1.72246300

H	-3.40732000	3.05945800	2.81838900
C	-2.32914700	2.13416400	1.16224600
H	-2.42463600	2.12441900	0.07278200
C	-4.76388900	2.68910500	1.21856200
C	16.75694800	0.79644400	-0.54088700
H	16.25855300	1.50949200	-1.20161900
H	17.61925300	0.37067800	-1.05575800
H	17.10210100	1.33216000	0.34602400
C	17.16078300	-0.93691400	1.89508600
H	18.08885100	-0.44286700	1.59211600
H	17.41981300	-1.75865000	2.56899100
H	16.57340300	-0.21013900	2.46070200
C	17.26392200	-4.26944100	-1.88559900
H	17.41409800	-5.07721900	-1.16068800
H	18.24272500	-3.89044700	-2.18263700
H	16.76652600	-4.69440200	-2.76035300
O	-3.52408700	-0.53635700	-3.04148000
H	-2.61708800	-0.40241100	-2.73341800
O	-6.82347000	-3.06107400	-2.17797900
H	-7.26666200	-2.57465800	-2.89215300
O	-3.66412500	-2.17787200	-0.61715700
O	-2.49224300	-0.23102900	-0.56099900
O	-0.86718100	3.90917800	-0.40525400
H	-0.10073300	3.43705400	-0.77951100
O	-1.03797700	2.60398000	1.51924900
O	-4.99053600	1.83046700	0.39682900

O	-5.76574800	3.43291000	1.73790600
H	-6.58991200	3.12439500	1.32284300
O	1.56430900	2.79315900	-0.40502100
H	2.39553100	2.38601800	-0.71212700
O	4.05505500	1.79738400	-0.27027100
H	4.84077800	2.27636200	-0.60868800
O	6.18825600	2.95995200	0.40427400
H	6.97254400	2.41131100	0.18633200
O	8.19097900	1.20623100	0.07751600
H	8.94475800	1.13725100	-0.53677600
O	10.71589600	0.83402700	-0.65348100
H	11.45139400	0.55738800	-1.22901500
O	13.28143300	0.13085800	-1.12279500
O	14.71334100	0.26389500	0.61178400
O	18.35786300	-1.91334700	-0.68170800
H	18.99443000	-1.74980100	0.02232600
O	-3.89718000	5.47942100	2.01694400
H	-4.82616100	5.24098700	1.90614400
N	-6.32587500	-0.63084600	-3.47316000
H	-5.61445000	-0.70669300	-4.19691600
H	-6.66064300	0.32730800	-3.48780700

AmB-Chol8

0 1

C	-9.85330200	1.92793000	1.90704000
H	-10.70042200	1.23436400	1.84298400
C	-9.96406000	2.87426700	0.71161300
H	-9.15647800	3.61929600	0.77523300
C	-9.79891400	2.10819400	-0.59447800
H	-10.60963600	1.36561300	-0.66371500
C	-8.42016500	0.47431800	0.45695300
H	-9.17581800	-0.31020000	0.31525800
C	-8.55714600	1.12679400	1.83208200
H	-8.54969800	0.33475900	2.59368700
C	-9.80192500	3.01499200	-1.80515700
H	-8.93370100	3.67729600	-1.76469100
H	-10.71249100	3.61426800	-1.80638000
H	-9.74799000	2.42848900	-2.72295900
C	-6.59459400	-0.44175300	-0.85804700
H	-6.54452100	0.44486700	-1.49861300
C	-5.20611800	-0.88949000	-0.51501900
H	-5.12131500	-1.56498800	0.33286500
C	-4.10828000	-0.44314100	-1.13204200
H	-4.19748200	0.23076500	-1.98288900
C	-2.77104500	-0.75895800	-0.66828300
H	-2.69240400	-1.31276500	0.26616800
C	-1.63839100	-0.36999800	-1.28391400
H	-1.70041700	0.17479000	-2.22499900
C	-0.32227100	-0.62118400	-0.74269900
H	-0.27863700	-1.05887800	0.25456600

C	0.82438500	-0.32956600	-1.39063200
H	0.77274300	0.09132400	-2.39349800
C	2.13482200	-0.49561700	-0.81035600
H	2.19339700	-0.86502500	0.21365800
C	3.28017100	-0.18586300	-1.45527300
H	3.23433200	0.17084500	-2.48336500
C	4.58297500	-0.26357300	-0.83964000
H	4.61564700	-0.55304000	0.21097000
C	5.73818900	0.01295100	-1.47936200
H	5.71173100	0.28302300	-2.53372200
C	7.03184800	-0.02941900	-0.83869400
H	7.04256500	-0.19556800	0.23801100
C	8.20519100	0.11948300	-1.48211300
H	8.20996300	0.26731500	-2.56067000
C	-7.48345400	-1.48257900	-1.54965100
H	-8.44933800	-1.01406500	-1.76088500
H	-7.02212400	-1.73434500	-2.50917500
C	9.48792800	0.07793000	-0.80412700
H	9.45188800	0.06297700	0.28397700
C	10.67132100	0.02513100	-1.42733500
H	10.69318600	0.03534300	-2.51862300
C	12.00213400	-0.08025700	-0.73992400
H	11.85754300	0.03279100	0.34015900
C	12.64126100	-1.46182900	-0.98039000
H	12.95456700	-1.49905400	-2.03589900
C	11.70082300	-2.66034900	-0.75609200

H	10.87942300	-2.53455400	-1.46787800
C	11.05000000	-2.65131900	0.63069500
H	10.73557700	-1.64278300	0.88120400
C	8.72676800	-2.96310300	1.05940400
C	7.54360300	-3.89305500	0.92528700
H	7.71466000	-4.57955500	0.09571900
H	7.49600600	-4.48075500	1.84921500
C	6.22389600	-3.13385500	0.74836500
H	6.26375200	-2.57033300	-0.19462000
C	5.03785000	-4.09198900	0.71577100
H	5.19572000	-4.82139500	-0.08534100
H	5.00326200	-4.63407900	1.66854200
C	3.69119400	-3.39974600	0.49397400
H	3.74253400	-2.82292300	-0.44274300
C	2.55519400	-4.41385600	0.38517500
H	2.58677500	-5.00513800	1.30974700
H	2.74531600	-5.09392300	-0.45257000
C	1.19969100	-3.72742800	0.22506100
H	1.09654300	-2.97078700	1.00642700
H	1.16555800	-3.20693900	-0.73823000
C	0.00731100	-4.67333700	0.30707000
H	0.05309700	-5.41730100	-0.50050200
C	-1.32573500	-3.92521600	0.21561400
H	-1.30755300	-3.25305800	-0.64830100
C	-2.51057100	-4.88250900	0.10719900
H	-2.47870000	-5.58783100	0.94431400

H	-2.40260000	-5.45867700	-0.81836300
C	-3.87260800	-4.18827900	0.09422000
H	-3.89639600	-3.41888900	-0.68953000
C	-4.97867800	-5.20027600	-0.17822400
H	-4.88447300	-6.03963500	0.51813600
H	-4.85194300	-5.58243900	-1.19424600
C	-6.39884400	-4.65195500	-0.03410300
C	-7.42660500	-5.63874300	-0.57171000
H	-7.44077900	-6.53598100	0.04965000
H	-7.15550700	-5.92350500	-1.59162500
C	-8.81975500	-5.03839700	-0.61695800
H	-9.17422200	-4.85887300	0.40797200
C	-8.77584300	-3.67370500	-1.35662300
H	-8.54760800	-3.88854200	-2.40634100
C	-7.68468100	-2.76925600	-0.76187600
H	-7.92893100	-2.53992700	0.28135500
C	-10.17935200	-3.12961500	-1.31427900
C	11.87581500	-3.21883200	1.76952600
H	11.33872500	-3.09775200	2.71201400
H	12.82280200	-2.68135600	1.82887400
H	12.07551400	-4.28068800	1.61621700
C	12.40140000	-3.98151200	-1.06941500
H	13.26036100	-4.14863200	-0.41408000
H	12.75171400	-3.99478200	-2.10458300
H	11.71786200	-4.82203200	-0.93434600
C	12.95406000	1.02324200	-1.21329600

H	13.09616900	0.96502900	-2.29694300
H	13.92551400	0.91672300	-0.73046500
H	12.54604800	2.00840200	-0.98009300
O	-7.48720400	2.02689300	2.03733700
H	-6.67921200	1.55415100	1.79942700
O	-11.22952500	3.49084500	0.70899500
H	-11.37802200	3.75908700	1.62497100
O	-8.54259200	1.42997900	-0.57314800
O	-7.12879500	-0.04329900	0.41984100
O	-6.72961800	-4.38715400	1.29963600
H	-5.96633800	-3.93382500	1.68776100
O	-6.44320500	-3.46060700	-0.81186800
O	-11.10413300	-3.61105000	-1.91737600
O	-10.36270900	-2.09990000	-0.46648000
H	-11.30839700	-1.89334300	-0.48505900
O	-4.14750100	-3.57143300	1.35397700
H	-3.33378400	-3.13584900	1.64231400
O	-1.45214500	-3.08510300	1.36938200
H	-1.07873000	-3.59563500	2.10204300
O	-0.05597600	-5.33369800	1.57137300
H	0.80973000	-5.68662800	1.78539800
O	3.35584200	-2.53340700	1.56620700
H	4.17647600	-2.15576200	1.90947900
O	6.00742800	-2.23020000	1.82625100
H	6.83979000	-1.76672700	1.99938100
O	8.66680300	-1.89427900	1.63001100

O	9.83269800	-3.44983400	0.51495300
O	13.79023200	-1.51768000	-0.14243100
H	14.36919400	-2.21499400	-0.45877200
O	-9.63712500	-5.97310200	-1.28211400
H	-10.45736700	-5.54047100	-1.54486100
N	-10.01272700	2.72649200	3.11941100
H	-9.12770500	3.18717800	3.31681300
H	-10.22398300	2.13684800	3.91669700
C	-5.04657000	2.84943600	-0.25268000
C	-3.90362200	3.08432900	-1.25425300
C	-3.50160900	4.56061300	-1.25914000
C	-4.90423700	3.80484000	0.96138400
C	-3.43641000	4.07586500	1.15518200
C	-2.71470800	3.40459200	2.05189600
C	-1.21250300	3.38009100	2.05637000
C	-0.66084500	3.65812200	0.65568600
C	0.86072900	3.78676800	0.68648900
C	1.65935400	2.57018200	1.14227000
C	3.10300400	2.89780000	0.69873600
C	3.04272400	4.09537700	-0.31103000
C	3.70331900	5.39674400	0.20825400
C	5.04414600	5.15645900	0.93867600
C	6.05041400	4.27791600	0.18942400
C	7.42234400	4.27220300	0.86450900
C	8.41603500	3.26623900	0.27241900
C	8.62352300	3.48609500	-1.22678100

C	9.75336900	3.33032400	1.00959200
C	3.95667500	6.39373600	-0.93241800
C	1.51494500	4.16635600	-0.65778100
C	1.27430800	3.08833600	-1.73251600
C	0.89846200	5.49230700	-1.11886800
C	-0.62920100	5.37403100	-1.21431500
C	-1.27591500	4.95963800	0.11448100
C	-2.84132200	4.98780400	0.09045900
C	-3.26501600	6.44014400	0.39367400
O	-6.26268000	3.06215700	-0.95054000
H	-0.98231100	5.72705200	0.84808900
H	-0.94354500	2.81324100	0.01239600
H	1.09129400	4.61376000	1.37807200
H	-4.99639300	1.81282700	0.10359700
H	-4.26080800	2.77161800	-2.23822800
H	-3.04508500	2.45803100	-0.99083600
H	-4.41629400	5.14528900	-1.40269300
H	-2.86278200	4.79711200	-2.10876900
H	-5.35550100	3.37863300	1.85891200
H	-5.46057000	4.71396900	0.71807600
H	-3.23438600	2.77705500	2.77267500
H	-0.86990800	2.40153500	2.40638000
H	-0.80204200	4.11874700	2.75967100
H	1.28174200	1.67171700	0.64358100
H	1.59079700	2.39833500	2.21816500
H	3.57740800	2.02924500	0.23819300

H	3.70577900	3.15320000	1.56917400
H	3.59155400	3.81944300	-1.22006300
H	3.02791600	5.86987000	0.93372000
H	5.50391600	6.13608000	1.11711700
H	4.87451000	4.73074700	1.93156100
H	6.15748400	4.63355300	-0.83966700
H	5.67735100	3.24824300	0.12299300
H	7.29572000	4.05616700	1.93263900
H	7.85847900	5.27876900	0.80333200
H	7.99490900	2.26327700	0.41198100
H	8.89220500	4.52987500	-1.42477700
H	7.72210400	3.25215800	-1.79792500
H	9.43056500	2.85193600	-1.60274700
H	10.22159500	4.31041900	0.86997600
H	10.44382800	2.57114300	0.63012300
H	9.62525400	3.16992400	2.08337400
H	4.64381600	5.96828800	-1.66892000
H	4.41212400	7.30869200	-0.54469300
H	3.05059000	6.67663100	-1.46136400
H	1.84621600	2.18642700	-1.50998900
H	1.61217800	3.46326000	-2.70412800
H	0.22668300	2.79632800	-1.82721700
H	1.28998600	5.78517400	-2.09787100
H	1.15160700	6.28872700	-0.40967800
H	-0.87170500	4.64534600	-1.99200800
H	-1.04555900	6.32925100	-1.55028600

H	-2.86159800	7.11886600	-0.36389200
H	-2.89219500	6.75459800	1.37122400
H	-4.35075500	6.55207200	0.38980000
H	-6.98771000	2.62147800	-0.48897500

AmB-Chol9

0 1

C	-6.62724100	-5.10271500	-2.65398900
C	-5.84058200	-4.16981500	-1.74763900
C	-5.45729400	-2.83585500	-2.40575600
C	-7.91409400	-4.39989900	-3.16127900
C	-7.87939500	-2.90832100	-2.92331600
C	-8.90111000	-2.28157600	-2.34228300
C	-8.89509500	-0.83451100	-1.95493900
C	-7.48000100	-0.25820100	-1.89469100
C	-7.52190400	1.26330000	-1.78648300
C	-8.23336400	1.87029200	-0.58105000
C	-7.70304900	3.32151300	-0.51777500
C	-6.54901200	3.45440500	-1.56480100
C	-6.89853500	4.25861500	-2.84671000
C	-7.85699500	5.44397200	-2.62416500
C	-7.39895200	6.48921000	-1.60635300
C	-8.42769900	7.60201400	-1.41787400
C	-8.08271200	8.62668400	-0.33134200

C	-6.79118500	9.38541200	-0.63922200
C	-9.24077500	9.60502100	-0.13542700
C	-5.62300300	4.76606600	-3.53336100
C	-6.14707300	1.95829200	-1.79570600
C	-5.27229300	1.53879500	-0.59952200
C	-5.44010900	1.56431000	-3.09751200
C	-5.33502600	0.04032200	-3.23938600
C	-6.69220000	-0.67711000	-3.14901200
C	-6.57702900	-2.22113100	-3.30347800
C	-6.27609700	-2.52879000	-4.78591000
O	-5.74946800	-5.49172700	-3.70510100
H	-7.29127500	-0.33110300	-4.00620000
H	-6.98590300	-0.67497400	-1.00879200
H	-8.04415900	1.62085300	-2.68822100
H	-6.91361200	-5.99665000	-2.08054200
H	-4.93887100	-4.68610900	-1.40426600
H	-6.46370100	-3.97607900	-0.86778400
H	-4.54987900	-2.96907900	-3.00028400
H	-5.20698700	-2.14685000	-1.59784400
H	-8.78939100	-4.82182700	-2.65863100
H	-8.02885900	-4.62128700	-4.22929400
H	-9.79826500	-2.85123200	-2.10381000
H	-9.39046900	-0.71714300	-0.98321400
H	-9.49981800	-0.25165400	-2.66560400
H	-7.96691000	1.32209800	0.32650500
H	-9.32217400	1.82560800	-0.66895200

H	-7.32822500	3.54734500	0.48402800
H	-8.50512300	4.03376400	-0.70220000
H	-5.70820300	3.97691800	-1.09421200
H	-7.41223900	3.58754500	-3.54761700
H	-8.00570700	5.94063300	-3.59147600
H	-8.84744500	5.07520000	-2.33648900
H	-6.44565500	6.91879500	-1.92832700
H	-7.20720200	6.01187700	-0.63782300
H	-9.39704900	7.14742100	-1.17398100
H	-8.57333200	8.12989800	-2.37101200
H	-7.93824100	8.07750800	0.60992800
H	-6.87286000	9.90857200	-1.59885300
H	-5.92594600	8.72046800	-0.69380100
H	-6.58408200	10.13464800	0.13059600
H	-9.42082900	10.17747900	-1.05224800
H	-9.02799800	10.31971900	0.66508500
H	-10.16798500	9.08173700	0.11703300
H	-5.06788300	5.43945000	-2.87356500
H	-5.86610800	5.32139400	-4.44450100
H	-4.94660000	3.95862000	-3.80928300
H	-5.67200500	1.89817600	0.35004300
H	-4.26963700	1.96485500	-0.70694700
H	-5.17024500	0.45646900	-0.51262400
H	-4.43417900	1.99513700	-3.14366200
H	-5.99660100	1.95557300	-3.95585200
H	-4.65755600	-0.34390600	-2.47227800

H	-4.85799000	-0.18727800	-4.19673200
H	-5.40255200	-1.96642400	-5.12340700
H	-7.12370300	-2.24683900	-5.41864300
H	-6.05434200	-3.58460800	-4.93473800
H	-6.22934700	-6.08377900	-4.29572000
C	12.22934300	4.09490000	-0.66221100
H	13.15999700	3.56127800	-0.43167900
C	11.88470400	4.94407700	0.56047400
H	10.96133600	5.50322100	0.34313700
C	11.60742100	4.05199000	1.76450100
H	12.52087900	3.46940800	1.97449500
C	10.82834200	2.30826100	0.36638900
H	11.69258300	1.67663700	0.62354500
C	11.13223100	3.06789300	-0.92196300
H	11.46610200	2.32934900	-1.66570900
C	11.20418400	4.83541500	2.99297100
H	10.27945300	5.38323500	2.79322900
H	11.98899100	5.54949000	3.24736800
H	11.03359600	4.16373700	3.83724600
C	9.27856000	0.64101100	1.13703300
H	9.39317000	1.13815500	2.10575100
C	7.82970700	0.37285300	0.87692300
H	7.58347800	-0.06997800	-0.08451200
C	6.85392600	0.66342000	1.74263100
H	7.09977500	1.11001300	2.70569000
C	5.45273200	0.43357300	1.45348200

H	5.22489000	-0.00787700	0.48647600
C	4.43429800	0.74509700	2.27731200
H	4.64480200	1.19441800	3.24750200
C	3.05296200	0.51914100	1.92506300
H	2.88161300	0.05208700	0.95716100
C	1.99240900	0.82887900	2.69772500
H	2.14891700	1.29612900	3.66947600
C	0.63254100	0.57206500	2.29322700
H	0.49149100	0.07237100	1.33477200
C	-0.46066900	0.89206300	3.01585700
H	-0.33904200	1.38159900	3.98143900
C	-1.80635800	0.63685500	2.56815600
H	-1.91568900	0.15193000	1.59779000
C	-2.92180000	0.95463200	3.25666000
H	-2.83163700	1.42929100	4.23290700
C	-4.24928400	0.69872000	2.75565300
H	-4.31154900	0.26036500	1.76257000
C	-5.40008600	0.94440200	3.40946700
H	-5.37586500	1.37169500	4.41112900
C	10.16657400	-0.60924300	1.09718900
H	11.19399600	-0.30447200	1.30875000
H	9.86044000	-1.29996100	1.88858000
C	-6.69233000	0.61842100	2.83840300
H	-6.66670100	0.26013200	1.81334600
C	-7.86706200	0.66699100	3.47568800
H	-7.90160300	1.00631100	4.51218200

C	-9.17539500	0.23266100	2.87624300
H	-9.04829100	0.10839800	1.79563600
C	-9.61803600	-1.13072500	3.44018700
H	-9.83374200	-0.98815800	4.51227600
C	-8.55566300	-2.24353600	3.34981600
H	-7.67706700	-1.85987300	3.87686300
C	-8.08538000	-2.52001500	1.91591500
H	-7.88283400	-1.58274700	1.40356500
C	-5.72813700	-2.70686700	1.47147400
C	-4.49449900	-3.54131600	1.69924700
H	-4.59062300	-4.07649500	2.64615000
H	-4.46122900	-4.28741000	0.89760500
C	-3.21296200	-2.70670800	1.66340000
H	-3.25022200	-1.95690600	2.46648300
C	-1.97856400	-3.57675600	1.84594700
H	-2.05662200	-4.10225200	2.80370800
H	-1.95626300	-4.33270700	1.05192900
C	-0.66386700	-2.80064600	1.81431600
H	-0.70526900	-1.98221900	2.54654200
C	0.52127500	-3.70033800	2.14875100
H	0.44306700	-4.62191700	1.55893700
H	0.42647400	-3.99199100	3.19916300
C	1.88632600	-3.03735500	1.90162300
H	1.78463300	-1.94947400	1.89090100
H	2.56976300	-3.26946800	2.72424100
C	2.57040200	-3.49510800	0.61461100

H	2.82340800	-4.55943000	0.71848700
C	3.87638700	-2.70307300	0.35068400
H	4.25721200	-2.29046200	1.29202000
C	4.96450300	-3.54770700	-0.30303700
H	4.59219900	-3.93850600	-1.25781800
H	5.18160600	-4.40573000	0.34367600
C	6.25464200	-2.76582500	-0.53258200
H	6.58760600	-2.33852000	0.42313600
C	7.37014500	-3.65043300	-1.07392000
H	7.04519900	-4.12680800	-2.00408900
H	7.57919200	-4.43552500	-0.34151400
C	8.66441100	-2.88970100	-1.36376700
C	9.82568200	-3.81748200	-1.69056300
H	9.63339100	-4.35053800	-2.62398900
H	9.93477700	-4.55254800	-0.88816200
C	11.13478400	-3.05695700	-1.81337500
H	11.07852500	-2.39797200	-2.69206400
C	11.35650100	-2.16743000	-0.55919800
H	11.58968100	-2.84525000	0.27044900
C	10.10165600	-1.34226200	-0.23748200
H	9.95748500	-0.61591900	-1.04262600
C	12.54205600	-1.26575900	-0.78724100
C	-8.98821400	-3.38238600	1.05893900
H	-8.59679200	-3.44153700	0.04182800
H	-9.98088900	-2.93093600	1.02892200
H	-9.06875900	-4.39300200	1.46521700

C	-9.01565800	-3.51171600	4.06719900
H	-9.93803300	-3.91387900	3.63716800
H	-9.19652100	-3.31141600	5.12710800
H	-8.25578200	-4.29327500	3.99687300
C	-10.26339600	1.28544900	3.10049600
H	-10.40994400	1.46950400	4.17067600
H	-11.21185100	0.95180300	2.67822800
H	-9.97960200	2.23170000	2.63360500
O	9.98911100	3.76374300	-1.37476000
H	9.22884400	3.20512300	-1.15915400
O	12.95740500	5.80818600	0.85759000
H	13.25433100	6.12243000	-0.01183800
O	10.53543100	3.17191500	1.44263500
O	9.69163100	1.55110200	0.10332200
O	8.52082700	-2.00385100	-2.43544800
H	7.64784400	-1.58310900	-2.33146000
O	8.97190100	-2.19880000	-0.15667600
O	12.53270900	-0.06055500	-0.87030300
O	13.69595100	-1.96281000	-0.94192000
H	14.39004800	-1.30828800	-1.12387300
O	6.04778300	-1.69959800	-1.46275200
H	5.13098000	-1.38754400	-1.34885400
O	3.55377100	-1.58587100	-0.47965100
H	2.77546300	-1.90949300	-0.98019000
O	1.75700700	-3.35591900	-0.54132300
H	0.89274400	-2.96813300	-0.28436800

O	-0.43583200	-2.23269500	0.52153400
H	-1.30005300	-1.95834800	0.16322300
O	-3.08183100	-2.04278800	0.40754700
H	-3.91495000	-1.56667200	0.25061000
O	-5.72140200	-1.68105400	0.80730000
O	-6.80448700	-3.22052300	2.04271700
O	-10.81685800	-1.47955100	2.75439600
H	-11.26883100	-2.16842800	3.25326600
O	12.14470000	-4.03096200	-1.96338000
H	13.00127600	-3.58615700	-1.93778100
N	12.49555500	5.00517200	-1.77277600
H	11.60074100	5.32052100	-2.14121200
H	12.96860000	4.51958500	-2.52831200

AmB-Chol10

0 1

C	-6.11272900	1.25219300	0.89937000
H	-6.73826800	0.61707500	0.26249600
C	-5.75724200	2.49396800	0.08180900
H	-5.14451700	3.16251500	0.70234300
C	-4.93982800	2.09869600	-1.14267700
H	-5.55903700	1.42789600	-1.76103900
C	-4.04825900	0.21603700	-0.02479800
H	-4.61837900	-0.46238400	-0.68203400

C	-4.84712100	0.47693500	1.25023400
H	-5.10872500	-0.49717400	1.69095600
C	-4.50123700	3.29629700	-1.95616600
H	-3.85241000	3.92974700	-1.34743800
H	-5.37449900	3.87245000	-2.26234500
H	-3.94768600	2.97477200	-2.83916500
C	-1.91808100	-0.65337700	-0.68943400
H	-1.89962500	0.19195700	-1.38148000
C	-0.57092800	-0.78161400	-0.04528200
H	-0.46438700	-1.51195200	0.75401200
C	0.47445400	-0.04236400	-0.42843600
H	0.34652300	0.67698400	-1.23609600
C	1.79573000	-0.11311200	0.16769400
H	1.93467000	-0.78823700	1.00950100
C	2.83393800	0.62713800	-0.26501200
H	2.67990800	1.29960200	-1.10793400
C	4.15950700	0.60334700	0.31056200
H	4.31922300	-0.04561900	1.17034300
C	5.17810700	1.33682100	-0.18459700
H	4.98783700	1.97020300	-1.05025200
C	6.52249000	1.35170800	0.33980400
H	6.74457400	0.74469600	1.21649700
C	7.51241800	2.07382100	-0.22689400
H	7.28629600	2.66600400	-1.11260700
C	8.87230300	2.12188400	0.25324300
H	9.10808200	1.56269000	1.15765700

C	9.84882500	2.80856900	-0.37513000
H	9.60573100	3.34580200	-1.29048600
C	11.21966500	2.87853400	0.07530300
H	11.45461400	2.41363500	1.03139900
C	12.20662800	3.46257400	-0.63055200
H	11.97385600	3.90744200	-1.59677800
C	-2.38898200	-1.90261400	-1.44264600
H	-3.36701300	-1.70494600	-1.87744700
H	-1.69728300	-2.09912800	-2.26629100
C	13.59281400	3.50057100	-0.20224100
H	13.79463400	3.15007600	0.80775200
C	14.60798200	3.87928600	-0.98777300
H	14.39032300	4.21654300	-2.00295500
C	16.05962700	3.83847400	-0.60647400
H	16.14658400	3.57550000	0.45315400
C	16.80591200	2.75968400	-1.41534000
H	16.86685900	3.11439300	-2.45668800
C	16.11210600	1.38610700	-1.45499600
H	15.13417500	1.55515700	-1.91606800
C	15.81576300	0.83077500	-0.05797400
H	15.42677000	1.62157700	0.57672700
C	13.70764200	-0.07243800	0.60533400
C	12.68646000	-1.15726000	0.36056100
H	12.80687900	-1.54707500	-0.65042400
H	12.90419200	-1.96627700	1.06706900
C	11.25273200	-0.66919600	0.59377200

H	11.02994400	0.13571700	-0.12154100
C	10.25669100	-1.80722100	0.40630300
H	10.39488600	-2.23374200	-0.59252000
H	10.48021700	-2.58780000	1.14369100
C	8.79436600	-1.38804400	0.56126000
H	8.58314000	-0.57732300	-0.15343500
C	7.85752900	-2.55692100	0.27457100
H	8.14533100	-3.35927000	0.96725700
H	8.02989400	-2.92850600	-0.74129500
C	6.38924400	-2.17504100	0.45369800
H	6.26854600	-1.65327500	1.40639700
H	6.09624000	-1.47807900	-0.33828200
C	5.44687000	-3.37194900	0.43482500
H	5.59351600	-3.95289500	-0.48667700
C	3.97579300	-2.96171600	0.52671800
H	3.76627900	-2.18557200	-0.21668700
C	3.04032300	-4.15061100	0.32082500
H	3.26417300	-4.91453800	1.07341900
H	3.24502400	-4.58566200	-0.66346500
C	1.55779400	-3.78847900	0.38810100
H	1.33530700	-3.00097600	-0.34482100
C	0.69626100	-5.00776700	0.08072100
H	0.96203300	-5.82198100	0.76207400
H	0.90606700	-5.33124800	-0.94226100
C	-0.80683900	-4.76525100	0.21159500
C	-1.62590100	-5.93477000	-0.31398500

H	-1.45070100	-6.82243000	0.29624400
H	-1.33161500	-6.15152100	-1.34478200
C	-3.10039500	-5.58899600	-0.28239700
H	-3.40759100	-5.45529900	0.76059100
C	-3.37597000	-4.27162700	-1.03238900
H	-3.20523000	-4.44389900	-2.10359500
C	-2.41881700	-3.14837100	-0.56157200
H	-2.68100200	-2.87691100	0.46831500
C	-4.85101300	-3.94532000	-0.86539500
C	16.96153900	0.13075000	0.64901500
H	16.64982200	-0.16670400	1.65194100
H	17.80866900	0.81289500	0.72731700
H	17.26907000	-0.76213600	0.10199600
C	16.88361300	0.39925500	-2.32989400
H	17.89070600	0.21702300	-1.94526300
H	16.97125200	0.77975600	-3.35063800
H	16.37000300	-0.56319300	-2.37170000
C	16.72684200	5.20140000	-0.81877200
H	16.62924200	5.51559400	-1.86250700
H	17.78706700	5.14539500	-0.57204600
H	16.25455700	5.96004900	-0.19304500
O	-4.10465200	1.25234900	2.16259500
H	-3.19028600	0.95105700	2.10360100
O	-6.93138400	3.14224400	-0.36088800
H	-7.50621500	3.19407700	0.41387300
O	-3.77066400	1.41114400	-0.71698800

O	-2.84113200	-0.34494700	0.37353200
O	-1.19585900	-4.54620400	1.53810400
H	-0.51999200	-3.98207700	1.94206200
O	-1.07878600	-3.62625000	-0.59347900
O	-5.63700000	-4.66393700	-0.29542600
O	-5.24152800	-2.79696100	-1.42870200
H	-6.20925700	-2.70592600	-1.31912000
O	1.19582600	-3.31669200	1.68907500
H	1.93537200	-2.79489200	2.02983700
O	3.75234400	-2.35570000	1.80517200
H	4.26677300	-2.88467900	2.43173500
O	5.64635100	-4.21212500	1.57209800
H	6.58649800	-4.27860500	1.75244100
O	8.50131400	-0.94674100	1.87790100
H	9.29300000	-0.51845300	2.22866500
O	11.09013800	-0.17674000	1.91921400
H	11.85431300	0.38083800	2.12499100
O	13.59641900	0.75367300	1.48615200
O	14.73782300	-0.13875100	-0.22671100
O	18.11514700	2.68303500	-0.86329800
H	18.68974800	2.24288600	-1.49384000
O	-3.80059900	-6.66519100	-0.87367700
H	-4.72703300	-6.53640000	-0.64843900
N	-6.91941000	1.66296300	2.04583500
H	-6.29770800	2.04313800	2.75640400
H	-7.38278500	0.85951100	2.46016700

C	-10.08636500	-4.73920800	-1.52168800
C	-10.96012200	-3.88723900	-0.59431800
C	-10.15441300	-3.47644700	0.63678600
C	-8.60326300	-4.30852400	-1.42590500
C	-8.55668800	-2.83324300	-1.13379100
C	-8.25979300	-1.93860900	-2.08250800
C	-8.50007100	-0.46532400	-1.92086000
C	-9.66912900	-0.22415900	-0.96357400
C	-9.84840400	1.26148000	-0.67019200
C	-10.01093300	2.21564300	-1.85055700
C	-10.64955000	3.47965400	-1.22211700
C	-10.99016500	3.15316200	0.26852700
C	-10.03895600	3.76533300	1.33281700
C	-9.41513300	5.11988800	0.95075400
C	-10.41290600	6.23849300	0.63887000
C	-9.71715600	7.47783900	0.07792700
C	-10.65789500	8.60590600	-0.35758600
C	-11.50359100	9.12958400	0.80415100
C	-9.85804000	9.74720900	-0.98578600
C	-10.77263000	3.90536600	2.67345200
C	-11.03202500	1.58762600	0.26091300
C	-12.38130500	1.17164500	-0.34993100
C	-10.81518500	0.83605100	1.58162700
C	-10.58919500	-0.66651300	1.34851100
C	-9.43539400	-0.94793800	0.37455500
C	-9.03170600	-2.46245600	0.26851700

C	-7.85731100	-2.68044200	1.24572800
O	-10.24559400	-6.08855300	-1.10632900
H	-8.55705000	-0.44318000	0.80271500
H	-10.57246500	-0.62335500	-1.44457800
H	-8.94225800	1.57723100	-0.13219900
H	-10.42983400	-4.62351800	-2.55745900
H	-11.83600900	-4.48034500	-0.32535500
H	-11.30824400	-2.99746000	-1.12867800
H	-9.69289700	-4.38070000	1.04551400
H	-10.79570200	-3.08575600	1.42422400
H	-8.06648300	-4.54758600	-2.34839700
H	-8.13899400	-4.87830200	-0.61674400
H	-7.92186900	-2.30047200	-3.05213200
H	-8.72009300	-0.02556800	-2.89767800
H	-7.60694100	0.05995200	-1.55682400
H	-10.66325000	1.77842200	-2.61365100
H	-9.05112100	2.43698200	-2.32054300
H	-11.56184200	3.75438500	-1.75633300
H	-9.97965300	4.33212000	-1.31216000
H	-11.99387300	3.53421600	0.48979200
H	-9.20240300	3.07496200	1.49496000
H	-8.77837100	5.44113600	1.78439200
H	-8.73810600	4.99797100	0.09944000
H	-10.96291600	6.50311800	1.54619000
H	-11.15778800	5.88691900	-0.08564500
H	-9.10807400	7.17859800	-0.78398900

H	-9.01539400	7.87120900	0.82610500
H	-11.33594200	8.20095500	-1.11984300
H	-10.85749300	9.47046000	1.62024200
H	-12.17359200	8.36501000	1.20162300
H	-12.11709200	9.97700200	0.48822300
H	-9.17618200	10.18344300	-0.24858100
H	-10.51552500	10.54262700	-1.34496300
H	-9.25841800	9.39562700	-1.82899900
H	-11.61065300	4.60130800	2.57988200
H	-10.10056300	4.28952700	3.44569900
H	-11.17703600	2.95560000	3.02279600
H	-12.57722500	1.69427200	-1.28869100
H	-13.18797400	1.42672300	0.34338400
H	-12.44566100	0.10162400	-0.55255200
H	-11.67176200	0.96702700	2.25075900
H	-9.93546100	1.24028300	2.09352400
H	-11.50949100	-1.12415500	0.97191500
H	-10.38323800	-1.14191800	2.31319600
H	-8.17867800	-2.45296600	2.26779400
H	-7.02087000	-2.01750900	0.99745500
H	-7.49023100	-3.70774300	1.22102200
H	-9.61971100	-6.62924100	-1.59474000

AmB-Chol11

0 1

C	-19.65446000	-1.86274400	-0.97030200
C	-18.51415500	-2.58636600	-1.66761400
C	-17.28559100	-1.69570900	-1.90939400
C	-19.17400100	-1.30358200	0.39539000
C	-17.66759400	-1.30956800	0.50567700
C	-17.05830000	-1.86396200	1.55166800
C	-15.57418600	-2.03480500	1.66817300
C	-14.87676400	-1.93234800	0.31188400
C	-13.36360100	-1.86086000	0.48284400
C	-12.67272200	-3.03682800	1.16758700
C	-11.18623300	-2.88625600	0.76672600
C	-11.09067200	-1.75215100	-0.30687800
C	-10.46952200	-0.42370500	0.20005800
C	-9.29422300	-0.60615600	1.18109500
C	-8.14343100	-1.48582000	0.68846900
C	-6.95468600	-1.46443000	1.64591200
C	-5.77692400	-2.36157600	1.25032800
C	-5.21284900	-1.99112500	-0.12292400
C	-4.68182100	-2.28287700	2.31410700
C	-9.99993900	0.45664300	-0.96750000
C	-12.56797100	-1.66673400	-0.82152400
C	-12.76625100	-2.83244600	-1.80896400
C	-13.06163100	-0.36781100	-1.46884100
C	-14.57670800	-0.39489400	-1.70800700
C	-15.39556800	-0.69410200	-0.44108400

C	-16.92690600	-0.76384200	-0.70672800
C	-17.43069400	0.66607500	-0.99282600
O	-20.10675200	-0.84662200	-1.86054700
H	-15.24242100	0.15483800	0.24389300
H	-15.13157100	-2.83227700	-0.26443900
H	-13.16504400	-0.96411900	1.09126100
H	-20.47461900	-2.57390400	-0.79556900
H	-18.87166200	-2.99624000	-2.61627200
H	-18.23017900	-3.43273400	-1.03317800
H	-17.44660500	-1.08531500	-2.80198700
H	-16.44508700	-2.35922600	-2.13258800
H	-19.59393300	-1.90164000	1.20918400
H	-19.57208200	-0.28851600	0.51339000
H	-17.66500900	-2.24735000	2.37062100
H	-15.35310300	-3.00270300	2.13210900
H	-15.15696300	-1.27756900	2.34899100
H	-13.08971500	-3.98177900	0.80322400
H	-12.81243400	-3.02989300	2.25143600
H	-10.79878300	-3.82290400	0.35760700
H	-10.57512900	-2.67023700	1.64141000
H	-10.44907800	-2.09514800	-1.12711400
H	-11.24035500	0.13239900	0.74999200
H	-8.89609300	0.39100600	1.40886700
H	-9.65294800	-0.99326400	2.14058500
H	-7.82209700	-1.14861800	-0.30109900
H	-8.48662500	-2.51870200	0.55893000

H	-7.29943700	-1.75206100	2.64768700
H	-6.58369100	-0.43469500	1.73814200
H	-6.13759900	-3.39843400	1.20513100
H	-4.95539400	-0.92734900	-0.14917700
H	-5.92793900	-2.17875400	-0.92728800
H	-4.31022700	-2.56850800	-0.34634900
H	-4.30164000	-1.25807000	2.39296700
H	-3.83804300	-2.93555100	2.07063600
H	-5.05888000	-2.57261200	3.29936200
H	-9.21291600	-0.04125800	-1.54155600
H	-9.58649600	1.40070600	-0.59895200
H	-10.79940900	0.69875100	-1.66428100
H	-12.37343900	-3.77330200	-1.41775800
H	-12.23179600	-2.61840000	-2.73984200
H	-13.81381200	-3.00146600	-2.06262500
H	-12.55641800	-0.18616200	-2.42366900
H	-12.82939600	0.48005800	-0.81535200
H	-14.80396300	-1.12997800	-2.48381900
H	-14.87762800	0.57215400	-2.11983400
H	-16.85678800	1.12117700	-1.80329600
H	-17.32332800	1.29865500	-0.10579200
H	-18.47412900	0.66087700	-1.30450300
H	-20.84350800	-0.38658400	-1.44197600
C	15.99011000	1.64809900	-1.57950100
H	16.68860800	1.00206700	-1.03290200
C	15.86938700	2.94916800	-0.78806400

H	15.18469800	3.62024600	-1.32959200
C	15.25977300	2.68116900	0.58281900
H	15.92926700	1.99067200	1.12399100
C	14.04197700	0.83201800	-0.25701200
H	14.64613300	0.12689200	0.33454900
C	14.63877200	0.94615700	-1.65693900
H	14.75856800	-0.07621000	-2.04483600
C	15.06028200	3.94266200	1.39162800
H	14.36893100	4.60937300	0.86945700
H	16.01602800	4.45390500	1.51711900
H	14.64046300	3.70702000	2.37207700
C	12.00310200	0.07487000	0.76427500
H	12.22012000	0.84516200	1.51133400
C	10.55988900	0.14343600	0.37515700
H	10.23008300	-0.55174100	-0.39236200
C	9.68989700	1.01071300	0.90152700
H	10.02005200	1.71094400	1.66835900
C	8.30531000	1.08741600	0.48100200
H	7.99380600	0.38122200	-0.28472500
C	7.40370600	1.97035100	0.95056500
H	7.70056400	2.68947700	1.71358200
C	6.04201600	2.01574600	0.47426400
H	5.77730600	1.26988200	-0.27260700
C	5.09874300	2.88452200	0.89020600
H	5.34644700	3.63615700	1.63907900
C	3.74990200	2.86963900	0.38051300

H	3.50710100	2.08973000	-0.34116000
C	2.77848800	3.73262900	0.74223200
H	2.99948700	4.50995500	1.47288400
C	1.44546200	3.70028100	0.19446200
H	1.23554800	2.92501200	-0.54244300
C	0.45764100	4.55406100	0.53145500
H	0.64949400	5.32033000	1.28173800
C	-0.85948900	4.51765300	-0.05645500
H	-1.02035400	3.78773200	-0.84800700
C	-1.89282100	5.29711100	0.31173600
H	-1.76298000	6.01597000	1.11993100
C	12.44571200	-1.29332900	1.29804200
H	13.50677500	-1.23377500	1.55064000
H	11.90213200	-1.51939400	2.22029200
C	-3.20737000	5.19303000	-0.29147100
H	-3.27460600	4.54168800	-1.16060900
C	-4.31786400	5.77537600	0.17380200
H	-4.25383600	6.41429100	1.05620600
C	-5.69299400	5.57471500	-0.39730200
H	-5.60876200	5.03052000	-1.34495200
C	-6.56258300	4.71802000	0.54370000
H	-6.76745500	5.32465400	1.44149000
C	-5.89968700	3.41816700	1.03660100
H	-4.98367000	3.72635400	1.54997100
C	-5.43638600	2.50765100	-0.10502300
H	-4.89486300	3.09355900	-0.84185000

C	-3.28035200	1.45619900	-0.09116100
C	-2.37698800	0.49956800	0.64480000
H	-2.66095300	0.47274800	1.69857000
H	-2.55733000	-0.49917600	0.23122800
C	-0.89737300	0.85366200	0.48292900
H	-0.71650500	1.84903200	0.91310300
C	-0.00892400	-0.16580900	1.18099600
H	-0.27057900	-0.19111700	2.24428900
H	-0.21442100	-1.15910500	0.76425700
C	1.48542200	0.11679300	1.04689100
H	1.69571400	1.13930800	1.38941600
C	2.31375400	-0.85967300	1.87519700
H	1.96126000	-1.87944000	1.67709200
H	2.10539200	-0.65574700	2.92990600
C	3.82439300	-0.76742000	1.60447300
H	4.07334600	0.20245900	1.16710300
H	4.37775900	-0.82541900	2.54687100
C	4.36244300	-1.88043600	0.70654200
H	4.26586700	-2.83435700	1.24353700
C	5.85694200	-1.65804900	0.36249500
H	6.32435600	-1.02868800	1.12849700
C	6.64289500	-2.95852000	0.23442600
H	6.19628800	-3.57279100	-0.55686100
H	6.55898400	-3.51667400	1.17404000
C	8.12001700	-2.72002700	-0.06484800
H	8.53995000	-2.06910400	0.71411500

C	8.91862200	-4.01707500	-0.08330600
H	8.49432900	-4.70133500	-0.82458400
H	8.84343300	-4.48806200	0.90105700
C	10.39644900	-3.81515700	-0.42078500
C	11.21821600	-5.08136900	-0.22772500
H	10.90303700	-5.84920400	-0.93730200
H	11.06081600	-5.46075200	0.78586200
C	12.70357900	-4.81908700	-0.40716600
H	12.88952300	-4.54957400	-1.45708800
C	13.14759400	-3.62444300	0.48128700
H	13.12441000	-3.97798300	1.51888400
C	12.20506300	-2.42456400	0.30515800
H	12.32705400	-2.04461400	-0.71346400
C	14.56442900	-3.24298300	0.13820900
C	-6.50017300	1.68453200	-0.79911700
H	-6.05218100	1.09736800	-1.60433600
H	-7.25321500	2.35388100	-1.21663300
H	-6.98885800	0.99883500	-0.10666900
C	-6.78464800	2.69733400	2.05138300
H	-7.74888900	2.41065500	1.62143600
H	-6.97819300	3.33702000	2.91744400
H	-6.30334600	1.78491200	2.40936800
C	-6.38332100	6.91235600	-0.67776500
H	-6.46247700	7.50573300	0.24000000
H	-7.38947800	6.74728400	-1.06557500
H	-5.81326300	7.49583700	-1.40424300

O	13.80182100	1.71363100	-2.49745400
H	12.89321000	1.50813300	-2.23514400
O	17.14388200	3.52796200	-0.62623400
H	17.57128800	3.40066900	-1.48880700
O	13.98264500	2.07668100	0.40472800
O	12.73904700	0.37862400	-0.43333000
O	10.57888800	-3.39408200	-1.74097500
H	9.87585900	-2.74417300	-1.92418500
O	10.86110500	-2.83733800	0.50504400
O	14.93846600	-2.20396600	-0.35163900
O	15.44482400	-4.24058600	0.40489900
H	16.31583600	-3.92264700	0.11588100
O	8.28893300	-2.08153700	-1.33330800
H	7.50823700	-1.51611800	-1.48218000
O	5.92151600	-0.92164400	-0.86063100
H	5.09669100	-1.19190600	-1.31616300
O	3.66580000	-2.00497400	-0.52484400
H	2.95786200	-1.32601600	-0.56613200
O	1.90753000	0.00612100	-0.31557600
H	1.18151300	0.33492300	-0.87729200
O	-0.53274300	0.86422200	-0.89511600
H	-1.20253400	1.39420300	-1.36290000
O	-2.95394400	2.02520000	-1.12052300
O	-4.46558000	1.58702800	0.49059000
O	-7.78056000	4.46794000	-0.15131900
H	-8.45034900	4.19554200	0.48504500

O	13.36081700	-6.01813000	-0.05861800
H	14.31237100	-5.85445700	-0.06189900
N	16.59120700	1.96983500	-2.87116200
H	15.86463800	2.36293900	-3.46573600
H	16.92220700	1.12779300	-3.33139300

AmB-Chol12

0 1

C	-15.33356900	-2.27637000	3.92845100
H	-16.24316200	-1.96158300	3.40141900
C	-15.00863100	-1.18073800	4.94314100
H	-14.12662500	-1.49039200	5.52213100
C	-14.66721700	0.12176500	4.22973700
H	-15.54643500	0.42640000	3.63868200
C	-13.84478700	-1.04133000	2.34831300
H	-14.68317400	-0.67144700	1.73589500
C	-14.19633000	-2.41364100	2.92014200
H	-14.49655300	-3.05653400	2.07972600
C	-14.28354500	1.22320900	5.19226800
H	-13.38262700	0.93274000	5.73693400
H	-15.09307400	1.38258000	5.90462000
H	-14.08271600	2.14974400	4.65304100
C	-12.21159300	-0.07065100	0.88570200
H	-12.30761300	0.79970300	1.54081000

C	-10.75848500	-0.33350800	0.62505600
H	-10.51068300	-1.26751200	0.12472600
C	-9.79025000	0.51536100	0.98165600
H	-10.05464700	1.45040000	1.47326400
C	-8.37447400	0.26667300	0.77886100
H	-8.08949500	-0.69543700	0.35723700
C	-7.41538500	1.15067900	1.11292200
H	-7.71266000	2.11125100	1.53159100
C	-5.99636400	0.91564100	0.96820700
H	-5.68801700	-0.05925300	0.59309500
C	-5.06865300	1.84472900	1.27876400
H	-5.40620400	2.81453600	1.64217100
C	-3.64133900	1.65706900	1.17288500
H	-3.26988800	0.68667100	0.84624200
C	-2.75809400	2.63894800	1.45168000
H	-3.13666400	3.61321500	1.75776300
C	-1.32341700	2.49801600	1.37918000
H	-0.92503700	1.51356000	1.13677100
C	-0.47412600	3.52512900	1.58882600
H	-0.88666900	4.50935500	1.80559800
C	0.96602800	3.42102200	1.54048000
H	1.39285900	2.42699400	1.41839500
C	1.79274800	4.48097000	1.62018100
H	1.36683000	5.47837400	1.71690300
C	-13.04817600	0.14865000	-0.38091700
H	-14.08838900	0.31096900	-0.08751800

H	-12.70408900	1.05609100	-0.88498600
C	3.23886900	4.38133800	1.54479500
H	3.65557800	3.37694900	1.57618800
C	4.05451400	5.43177400	1.39487000
H	3.62363300	6.43399900	1.35536900
C	5.54535100	5.34569000	1.23257300
H	5.86664500	4.31127000	1.39081000
C	5.94380800	5.76481000	-0.19532400
H	5.73067500	6.84029900	-0.29601900
C	5.17329900	5.05199100	-1.31976100
H	4.12745600	5.34293400	-1.18149500
C	5.18210300	3.52218300	-1.20513200
H	5.01307900	3.22640300	-0.17345700
C	3.20909600	2.18940500	-1.40733800
C	2.05848800	1.80139700	-2.30509000
H	1.90809400	2.57761600	-3.05597900
H	2.34838200	0.87932000	-2.82144800
C	0.77212200	1.54329400	-1.51386000
H	0.48386000	2.46624200	-0.99024000
C	-0.35839000	1.11118500	-2.43944400
H	-0.49812000	1.88188200	-3.20440100
H	-0.06120900	0.18269800	-2.94191300
C	-1.69128600	0.88036400	-1.72588900
H	-1.96403000	1.79887000	-1.18296600
C	-2.79698000	0.54910900	-2.72313700
H	-2.45470100	-0.33750300	-3.27316400

H	-2.90063100	1.36673100	-3.44507000
C	-4.13135300	0.27954200	-2.03117600
H	-3.97708000	-0.45297000	-1.23520500
H	-4.48955900	1.20186700	-1.56124800
C	-5.21112700	-0.24242900	-2.97060900
H	-5.38525300	0.47604700	-3.78396100
C	-6.53631800	-0.49805100	-2.25011300
H	-6.81524000	0.38843400	-1.67155900
C	-7.65191400	-0.86075700	-3.22765100
H	-7.34792400	-1.74200000	-3.80253600
H	-7.77572100	-0.03233500	-3.93360100
C	-8.99906400	-1.13240800	-2.56078900
H	-9.30144200	-0.26306400	-1.96170700
C	-10.06723900	-1.40216700	-3.61475500
H	-9.73655500	-2.21439700	-4.26961800
H	-10.19328600	-0.50002900	-4.21921100
C	-11.42702500	-1.79549700	-3.04048700
C	-12.51128200	-1.83234000	-4.10771200
H	-12.29493000	-2.61337900	-4.83883800
H	-12.54819000	-0.86864500	-4.62253600
C	-13.87378500	-2.09286500	-3.49135600
H	-13.88765500	-3.10798300	-3.07143300
C	-14.13659200	-1.08777800	-2.33728200
H	-14.30305300	-0.11108200	-2.80738500
C	-12.94732600	-1.00812100	-1.36810900
H	-12.86681000	-1.95547000	-0.82616600

C	-15.43844500	-1.50056000	-1.69500500
C	6.40669800	2.80073200	-1.73299100
H	6.28663400	1.72546600	-1.58475400
H	7.28075100	3.12158500	-1.16582800
H	6.55959500	2.99332700	-2.79625400
C	5.61677500	5.54203400	-2.69776600
H	6.66974700	5.31965800	-2.89256800
H	5.47150000	6.62160900	-2.78827000
H	5.03436900	5.05685500	-3.48311400
C	6.26220400	6.22578200	2.26232400
H	5.97438800	7.27465000	2.13942200
H	7.34410700	6.14982900	2.15166200
H	5.99367900	5.91516900	3.27274800
O	-13.09240600	-2.96800400	3.59948100
H	-12.30440400	-2.71518500	3.10434800
O	-16.12110100	-0.95959200	5.77878100
H	-16.43843300	-1.84266700	6.00742600
O	-13.56228300	-0.10510300	3.36114900
O	-12.69671900	-1.22655500	1.58933600
O	-11.40201100	-3.05594000	-2.43269600
H	-10.57404100	-3.11227000	-1.93252000
O	-11.75061700	-0.77948500	-2.09916500
O	-16.48642200	-1.57877400	-2.28520700
O	-15.34564400	-1.84920700	-0.39819100
H	-16.22849400	-2.14301500	-0.13148600
O	-8.93312800	-2.27635100	-1.70480100

H	-8.06677400	-2.27211700	-1.27473700
O	-6.33760900	-1.54661800	-1.29401300
H	-5.75772400	-2.18687500	-1.73043200
O	-4.86378800	-1.51629500	-3.51417400
H	-3.95702800	-1.48928400	-3.82598800
O	-1.62722500	-0.20650700	-0.81644200
H	-0.73785900	-0.22265000	-0.43941900
O	0.96302600	0.50410700	-0.56044200
H	1.79655700	0.67559400	-0.09960200
O	3.36827300	1.72727300	-0.29822400
O	4.02516200	3.06973700	-1.97181400
O	7.35715700	5.57178300	-0.30155300
H	7.67897400	5.98752500	-1.10507600
O	-14.82220600	-1.95614900	-4.52356600
H	-15.70027300	-1.96730100	-4.12536500
N	-15.64069200	-3.49446700	4.67532300
H	-14.76051600	-3.91635800	4.96207700
H	-16.11190600	-4.17104300	4.08512200
C	9.45626400	2.93701800	1.29663000
C	9.49170100	1.42366900	1.53583300
C	9.46038700	0.69372200	0.19477200
C	10.22100200	3.30754000	0.00021300
C	11.30350700	2.28933400	-0.23026300
C	12.58285100	2.56084100	0.02038600
C	13.64215100	1.49966400	0.08721000
C	13.02349500	0.17220600	0.52944300

C	14.05974000	-0.94735100	0.52862000
C	15.29530100	-0.78103300	1.41294600
C	15.84586800	-2.22216200	1.56435200
C	14.81065600	-3.19834800	0.91501300
C	15.20645500	-3.77632200	-0.47007300
C	16.71424300	-4.02267600	-0.66056600
C	17.36619000	-4.94480800	0.37227200
C	18.87633900	-5.05519700	0.16537400
C	19.61709500	-5.85502400	1.24233100
C	19.11334600	-7.29599400	1.33479600
C	21.12184000	-5.83617200	0.97333300
C	14.44561300	-5.08242600	-0.73683800
C	13.50706000	-2.33259500	0.91840900
C	12.95951800	-2.35185200	2.35804300
C	12.37599100	-2.68386500	-0.05635700
C	11.30113500	-1.58705500	-0.07642200
C	11.87270700	-0.20436000	-0.42106300
C	10.77899200	0.90769900	-0.61053500
C	10.41388800	0.92365400	-2.10968100
O	8.09208800	3.31937200	1.20456100
H	12.35641700	-0.31260000	-1.40389800
H	12.63343000	0.31142400	1.54638300
H	14.41220800	-1.04095400	-0.51081500
H	9.92821600	3.45073500	2.14352300
H	8.63065100	1.16467100	2.15503500
H	10.39693900	1.15624800	2.08962900

H	8.62702400	1.11071700	-0.38096800
H	9.23412600	-0.36404600	0.31562800
H	10.63310700	4.31685700	0.07145800
H	9.50142500	3.30032700	-0.82579800
H	12.86276500	3.58259300	0.26583400
H	14.42229400	1.80584700	0.79004900
H	14.13829300	1.36230100	-0.88398400
H	15.01106500	-0.36031600	2.38277500
H	16.03121500	-0.10445800	0.97475800
H	15.98067200	-2.47268800	2.61865000
H	16.83014100	-2.30988700	1.10954600
H	14.66855600	-4.05619300	1.58247900
H	14.92019300	-3.05476700	-1.24520400
H	16.85870700	-4.45873300	-1.65637900
H	17.24932900	-3.06830200	-0.68087800
H	16.91419200	-5.93863700	0.30910500
H	17.16924700	-4.57667600	1.38654900
H	19.30258000	-4.04500100	0.12570700
H	19.07593400	-5.51087300	-0.81415700
H	19.43474400	-5.36526500	2.20758700
H	19.21139500	-7.79467200	0.36446200
H	18.06528300	-7.34533700	1.63592400
H	19.69575900	-7.86547900	2.06312300
H	21.34385100	-6.33128900	0.02250100
H	21.67151100	-6.35968300	1.75924600
H	21.50281600	-4.81360400	0.91556400

H	14.70725400	-5.83711800	0.00961600
H	14.69782700	-5.48427400	-1.72185200
H	13.36580800	-4.94934600	-0.69612100
H	13.74839100	-2.18554300	3.09391000
H	12.52124600	-3.33284700	2.56402200
H	12.18807900	-1.60102500	2.53292600
H	11.90932700	-3.63548400	0.21764400
H	12.77633100	-2.79651300	-1.06961000
H	10.80680300	-1.55007700	0.89815900
H	10.52782500	-1.85884400	-0.80242300
H	10.02777900	-0.05518500	-2.41200800
H	11.29058600	1.15337000	-2.71960600
H	9.64017300	1.66495600	-2.32573200
H	8.03554700	4.17543700	0.75813700

AmB-Chol13

0 1

C	4.25172700	3.33122500	3.03451400
H	4.49172200	4.30102700	2.55884900
C	4.99404100	2.24816300	2.23169400
H	4.79956500	1.26206200	2.70182800
C	4.46882200	2.19021600	0.79147300
H	4.62986000	3.18130100	0.31441500
C	2.32413100	2.94756800	1.48319700

H	2.50705200	3.90597000	0.94551500
C	2.73053000	3.12815200	2.95561100
H	2.20930400	4.03267700	3.33546700
C	5.12230900	1.10469400	-0.04278000
H	4.92562700	0.11623000	0.40068700
H	6.20869400	1.26708300	-0.07406100
H	4.72592300	1.11856400	-1.06843000
C	0.36076900	2.24962600	0.20573900
H	0.81196200	1.29901700	-0.12592800
C	-1.09083400	2.05455600	0.51177700
H	-1.61397200	2.91133400	0.94962100
C	-1.76801900	0.90149400	0.28496300
H	-1.23736800	0.04539900	-0.15289500
C	-3.16059000	0.73196000	0.60021000
H	-3.67117700	1.58911000	1.05537600
C	-3.88763700	-0.41232000	0.39333100
H	-3.39666100	-1.28203200	-0.06323700
C	-5.26597200	-0.52727600	0.75037700
H	-5.72552300	0.36194200	1.20011800
C	-6.05209300	-1.63719900	0.54755100
H	-5.61077800	-2.52495400	0.07561500
C	-7.43072000	-1.69326100	0.90700000
H	-7.85510300	-0.80473900	1.39354600
C	-8.26605600	-2.76267400	0.67586700
H	-7.85588600	-3.65084900	0.17742300
C	-9.64433300	-2.79009200	1.03863700

H	-10.04237000	-1.90781800	1.55798900
C	-10.50342900	-3.83238600	0.77723500
H	-10.11308100	-4.71138100	0.24751100
C	-11.88390400	-3.84605000	1.14331300
H	-12.26204500	-2.97794000	1.69949300
C	-12.76684700	-4.85035900	0.83904300
H	-12.40468000	-5.71970300	0.27471500
C	0.64587100	3.31193000	-0.87515500
H	1.72842200	3.32826000	-1.07082900
H	0.16767600	2.98465900	-1.81259800
C	-14.16192800	-4.81771200	1.18590500
H	-14.49334100	-3.95319100	1.77572900
C	-15.08540300	-5.73564700	0.80113600
H	-14.75714500	-6.59997500	0.20574800
C	-16.55535900	-5.65939200	1.10557700
H	-16.74039300	-4.79040400	1.76270500
C	-17.38330100	-5.44039300	-0.18810600
H	-17.33342400	-6.38299500	-0.77448100
C	-16.85137200	-4.32007200	-1.11744200
H	-15.81309200	-4.61546200	-1.35174700
C	-16.73216600	-2.95571500	-0.41466900
H	-16.28655900	-3.08887100	0.57849800
C	-14.80788700	-1.49625500	-0.53578000
C	-13.85481400	-0.76964700	-1.46324500
H	-13.83768900	-1.27227100	-2.44155500
H	-14.25570500	0.24840100	-1.61857400

C	-12.43875300	-0.66607400	-0.87100500
H	-12.04599700	-1.68972800	-0.70532100
C	-11.48648500	0.08929600	-1.79839000
H	-11.44120600	-0.44651400	-2.76208700
H	-11.89063600	1.09875700	-1.99523800
C	-10.06139500	0.22441300	-1.24518500
H	-9.68287600	-0.78090100	-0.97127000
C	-9.10623600	0.84413500	-2.26894700
H	-9.56482600	1.76284100	-2.67893000
H	-9.02270300	0.13417000	-3.10813700
C	-7.70017600	1.15949800	-1.71056400
H	-7.48394600	0.52509000	-0.83577400
H	-6.93920500	0.90180300	-2.46599500
C	-7.46721300	2.63192400	-1.34639000
H	-7.57159800	3.24041900	-2.26674300
C	-6.03636400	2.86165700	-0.77060000
H	-5.36001800	2.06957200	-1.13818800
C	-5.45157800	4.22873600	-1.14549900
H	-6.12192600	5.03274000	-0.79193400
H	-5.40513800	4.29374000	-2.24745900
C	-4.04185200	4.43775100	-0.58298800
H	-3.40568400	3.58398200	-0.89005100
C	-3.38930800	5.72391700	-1.09171900
H	-3.99903900	6.59404200	-0.79709300
H	-3.35207500	5.68662100	-2.19206600
C	-1.96027900	5.94422500	-0.56727200

C	-1.27776400	7.15501300	-1.21793600
H	-1.77016600	8.07994200	-0.88412200
H	-1.37732600	7.08420900	-2.31344800
C	0.20153600	7.20937300	-0.85707700
H	0.29782000	7.34808500	0.23410400
C	0.88361400	5.88001900	-1.24764500
H	0.78901100	5.72373200	-2.33571500
C	0.13011100	4.71025700	-0.53563700
H	0.18374500	4.88247100	0.55621300
C	2.36689000	5.85798900	-0.95161400
C	-17.99757600	-2.12150600	-0.29644400
H	-17.78973600	-1.19430100	0.25752200
H	-18.75627200	-2.69878500	0.25164900
H	-18.40080100	-1.85648900	-1.28485500
C	-17.63157500	-4.26097000	-2.43694100
H	-18.69443700	-4.01197500	-2.28284000
H	-17.58197000	-5.23002600	-2.95887900
H	-17.21214700	-3.49709200	-3.10844800
C	-17.04057900	-6.92454800	1.83894400
H	-16.84981900	-7.82469900	1.23029200
H	-18.11992900	-6.86429600	2.03764100
H	-16.50862900	-7.04470900	2.79403800
O	2.38653100	1.98994500	3.74058900
H	1.52694000	1.67917700	3.39847700
O	6.39034300	2.53765100	2.21611800
H	6.57625800	2.83853700	3.13007800

O	3.05135700	1.91224700	0.83588900
O	0.96224300	2.60982800	1.49272800
O	-1.92481000	6.13857000	0.83255400
H	-2.60879500	5.53930800	1.21173500
O	-1.24277400	4.74125300	-0.94486200
O	3.19886300	5.18946900	-1.53689500
O	2.71485600	6.67698500	0.09820000
H	3.68258400	6.57129200	0.20484400
O	-4.06478900	4.49960700	0.86290700
H	-4.74908100	3.85387400	1.15666800
O	-6.09292400	2.70498300	0.66531800
H	-7.02396100	2.97509400	0.86904300
O	-8.38836600	3.14744800	-0.37340400
H	-9.08073900	2.45884400	-0.19432400
O	-10.03860400	1.05140600	-0.05987100
H	-10.85529500	0.84872300	0.45086900
O	-12.46756600	0.03428500	0.38925100
H	-13.18157600	-0.38686200	0.91971900
O	-14.73260400	-1.44566500	0.69127300
O	-15.73932900	-2.18920700	-1.20989300
O	-18.74280500	-5.21589500	0.23398400
H	-19.32332400	-5.35684800	-0.53108500
O	0.77524000	8.32792100	-1.55125800
H	1.61506400	8.53564700	-1.10781400
N	4.81781200	3.35210900	4.39037000
H	4.39960700	2.59059200	4.93494000

H	4.58736900	4.22417400	4.87170600
C	7.49378000	-0.66522000	0.67602300
C	8.08774200	-1.99187300	0.13124800
C	9.01000800	-1.84453400	-1.11512000
C	8.31528200	0.57754500	0.30366500
C	9.73323600	0.09449800	0.23575400
C	10.44117700	0.02090800	1.38082400
C	11.57628500	-0.93450000	1.59013600
C	11.80732300	-1.93207900	0.42461800
C	13.22599800	-2.47962200	0.53453700
C	13.52187200	-3.51542800	1.61600600
C	14.94135800	-3.99316600	1.25705200
C	15.27284400	-3.40502700	-0.16427700
C	16.31170800	-2.22703500	0.00141600
C	17.68220500	-2.73398300	0.53483200
C	18.30368300	-3.77761200	-0.38892100
C	19.76701500	-4.06167600	-0.07349000
C	20.35087100	-5.10126500	-1.04585800
C	20.36070200	-4.59386500	-2.49554100
C	21.76384500	-5.48570300	-0.61947800
C	16.58238600	-1.39857700	-1.26504700
C	13.83428800	-3.12135800	-0.71952300
C	13.19219700	-4.48692100	-1.12427400
C	13.64775400	-2.18120800	-1.90942100
C	12.16445200	-1.85768700	-2.14653500
C	11.57034200	-1.16492700	-0.90479400

C	10.07339000	-0.70110800	-1.02315000
C	9.95610100	0.16442700	-2.30495700
O	6.15404000	-0.50367200	0.22202800
H	12.16515900	-0.23848800	-0.82011200
H	11.09418900	-2.76328300	0.50117900
H	13.84034700	-1.60137700	0.77093500
H	7.44909900	-0.74960900	1.77076000
H	7.28392100	-2.70163500	-0.09654200
H	8.68616100	-2.45181900	0.93072400
H	8.35054500	-1.67588600	-1.97280500
H	9.49516200	-2.81030500	-1.29981400
H	8.18279000	1.37053400	1.05246800
H	7.97624600	1.01352700	-0.63445400
H	10.09491800	0.53931100	2.27086100
H	11.39042700	-1.50690100	2.51020200
H	12.47257000	-0.32988500	1.77886400
H	12.81342500	-4.35386200	1.57793300
H	13.47308800	-3.10021900	2.62645600
H	14.98610200	-5.08867600	1.24172200
H	15.63200600	-3.66258800	2.03542400
H	15.72883600	-4.17982600	-0.78943500
H	15.94180900	-1.52565000	0.76016600
H	18.35950100	-1.87564500	0.63955300
H	17.58728600	-3.14766900	1.54431400
H	18.21808400	-3.43079600	-1.42079100
H	17.74596200	-4.71693300	-0.31178600

H	19.84634800	-4.43640400	0.95361200
H	20.34763300	-3.13197700	-0.11953600
H	19.72878300	-6.00412900	-1.01027500
H	20.83554300	-3.60908000	-2.56760600
H	19.34547300	-4.51022400	-2.89754000
H	20.90253500	-5.28429400	-3.14896300
H	22.43526100	-4.62051800	-0.64217100
H	22.17331600	-6.25292400	-1.28294800
H	21.76327600	-5.89283100	0.39629500
H	16.49392000	-1.99859400	-2.17523200
H	17.58494500	-0.95539700	-1.25521000
H	15.90652900	-0.54199500	-1.32655000
H	13.37351300	-5.29153000	-0.40606600
H	13.61552000	-4.83230500	-2.07572100
H	12.10722000	-4.42089500	-1.24369600
H	14.09644300	-2.59848500	-2.81689300
H	14.12536100	-1.22132200	-1.72394400
H	11.58924700	-2.74279800	-2.42700900
H	12.13299900	-1.17906800	-3.00693100
H	10.12205900	-0.44081000	-3.20565100
H	10.68617200	0.98103000	-2.30812500
H	8.96413200	0.60461900	-2.43332900
H	5.78251600	0.25341900	0.71086000

AmB-Chol14

C	14.58539447	3.12973687	10.67721498
H	14.52731096	2.63959752	9.68688138
C	15.65820403	2.37551098	11.48269368
H	15.76479227	2.86191442	12.47430497
C	15.23076064	0.92136036	11.71973228
H	15.09113248	0.42688470	10.73395179
C	12.93290033	1.52843307	11.66437802
H	12.82649130	0.97481030	10.70350646
C	13.20802802	3.00823602	11.34773981
H	12.41644239	3.35079726	10.64761392
C	16.22118816	0.13301890	12.55576636
H	16.32740163	0.59276081	13.55063522
H	17.20264462	0.13182645	12.06147463
H	15.87832407	-0.90451376	12.67922289
C	11.33470152	0.20689640	12.95786832
H	12.07246257	-0.08863099	13.72311832
C	10.00673964	0.47202932	13.59432869
H	9.22614303	0.87357362	12.93943902
C	9.73301604	0.25082676	14.90418414
H	10.52053092	-0.15051173	15.55607113
C	8.45275570	0.52641256	15.49778536
H	7.68331825	0.94564694	14.83839930
C	8.12822847	0.32337678	16.81468768
H	8.88303402	-0.09757986	17.49216530
C	6.84287289	0.63991803	17.35149866

H	6.11204324	1.05458204	16.64573324
C	6.45138241	0.43830472	18.65424250
H	7.16526156	0.00086135	19.36460208
C	5.14723914	0.75588387	19.13519276
H	4.44865294	1.20843518	18.41862172
C	4.69740688	0.52352268	20.41523679
H	5.38283229	0.05863129	21.13607630
C	3.38451494	0.84377228	20.86883803
H	2.71168259	1.33006892	20.14954696
C	2.90476174	0.57980201	22.13094416
H	3.56965097	0.08282711	22.84965754
C	1.58535956	0.90297284	22.57213433
H	0.93448804	1.42701416	21.85961135
C	1.07266641	0.59455143	23.80616351
H	1.70906210	0.06189976	24.52496255
C	11.31273603	-0.88874214	11.87287973
H	12.34227987	-1.05096640	11.52046688
H	10.99568976	-1.83283602	12.34483742
C	-0.27437814	0.89700188	24.20818921
H	-0.88101331	1.45619764	23.48402347
C	-0.84959892	0.50489492	25.37387841
H	-0.24581411	-0.05996438	26.09891745
C	-2.27978943	0.76162372	25.75847577
H	-2.75136456	1.39252509	24.98337908
C	-3.08656180	-0.56188334	25.82564444
H	-2.72283918	-1.12459752	26.71214375

C	-2.89797724	-1.49973216	24.60637832
H	-1.81152969	-1.69476935	24.56429913
C	-3.23741874	-0.82514376	23.26500587
H	-2.80933317	0.18420441	23.23978315
C	-1.86311827	-0.91985255	21.27757834
C	-1.15174078	-1.83367766	20.30015087
H	-0.94232295	-2.79916725	20.78387500
H	-1.84495835	-2.02476734	19.46098630
C	0.13809882	-1.20020762	19.75084896
H	0.82496839	-0.99889509	20.59776052
C	0.83971320	-2.11463981	18.74636183
H	1.08569586	-3.06388765	19.25259767
H	0.14761279	-2.34712126	17.91687528
C	2.12969061	-1.52062008	18.16475103
H	2.79333404	-1.21202134	18.99740058
C	2.88001866	-2.52814823	17.28932639
H	2.17246935	-2.97321263	16.56565317
H	3.21220814	-3.34778879	17.94743601
C	4.09536614	-1.93368941	16.54246473
H	4.46688350	-1.03824266	17.06652051
H	4.92597530	-2.65895666	16.55780191
C	3.84253181	-1.59639276	15.06691803
H	3.58694701	-2.53329349	14.53296584
C	5.10755049	-0.98180076	14.39338660
H	6.01074175	-1.30986207	14.93798242
C	5.24874496	-1.36965623	12.91654424

H	4.34803376	-1.05569481	12.35892017
H	5.31298084	-2.47084074	12.85358810
C	6.50068367	-0.76851432	12.26946676
H	7.38281177	-1.03592767	12.88449418
C	6.73641422	-1.28613336	10.84983727
H	5.87471971	-1.03070116	10.21114360
H	6.82385218	-2.38364024	10.88688560
C	8.00454025	-0.72268560	10.18644686
C	8.29751071	-1.37946545	8.83071557
H	7.52859130	-1.08248534	8.10273766
H	8.26541720	-2.47562781	8.94239378
C	9.67126715	-0.97410537	8.31112199
H	9.67916706	0.11606557	8.13604232
C	10.74879571	-1.31259826	9.36436016
H	10.74787366	-2.39920430	9.55556485
C	10.37336340	-0.59767189	10.70264187
H	10.33028078	0.49106985	10.50910787
C	12.15260190	-0.97013707	8.91684010
C	-4.70371052	-0.76555376	22.86803842
H	-4.81682397	-0.22685276	21.91578230
H	-5.26337042	-0.22820196	23.64733988
H	-5.13299041	-1.77186251	22.75471362
C	-3.60847683	-2.84387491	24.81072658
H	-4.70079706	-2.72867996	24.90553338
H	-3.23910620	-3.34159993	25.72165181
H	-3.42465195	-3.51939191	23.96205971

C	-2.37153709	1.50843292	27.10299928
H	-1.88656112	0.92686601	27.90530578
H	-3.42210847	1.67201652	27.38165423
H	-1.86416845	2.48212356	27.03883718
O	13.20870917	3.80809316	12.52683712
H	12.50263805	3.44663917	13.09545758
O	16.89347356	2.39673146	10.77039890
H	16.94222958	3.30887340	10.41537891
O	13.97074206	0.92803598	12.42737362
O	11.74560879	1.50335270	12.41177907
O	7.92597795	0.67272769	9.97386816
H	7.45030931	1.04412281	10.75267085
O	9.07568500	-1.05001412	11.10842028
O	13.17284989	-1.51372708	9.29796440
O	12.18811060	0.07088753	8.01742325
H	13.13573271	0.20998829	7.81320719
O	6.40643517	0.67416286	12.20043956
H	5.94814970	0.96126137	13.02449762
O	5.05010996	0.45488458	14.54252179
H	4.07447864	0.62328220	14.57536376
O	2.77125131	-0.66464410	14.85433232
H	2.32291948	-0.49140758	15.72301719
O	1.84909093	-0.35449720	17.35801164
H	1.11859053	0.13511260	17.80024490
O	-0.15462206	0.04210646	19.07962152
H	-0.72005855	0.55964738	19.69687732

O	-1.85257991	0.30746073	21.19111280
O	-2.50564077	-1.60650703	22.23563055
O	-4.46261341	-0.18756538	26.03343976
H	-4.94138794	-0.96688130	26.35846487
O	9.89141400	-1.67563402	7.07760449
H	10.60723041	-1.21117059	6.61179754
N	15.06631791	4.50170877	10.46358147
H	14.88757832	5.05036813	11.31115735
H	14.55714067	4.95546667	9.70191917
C	16.00809282	-3.67759620	9.57360951
C	17.03679432	-4.54702051	10.34526584
C	17.31476572	-5.95050220	9.72988967
C	15.86749971	-4.05690477	8.09227899
C	17.22450153	-4.55430121	7.69267679
C	18.15736212	-3.65103740	7.32981794
C	19.63078672	-3.88207021	7.47379082
C	20.01274121	-5.20332667	8.19168078
C	21.45769522	-5.54059538	7.84113716
C	22.57193568	-4.73685886	8.50633444
C	23.84169433	-5.51196802	8.10766040
C	23.36412835	-6.86671512	7.46535449
C	23.58773419	-6.79678916	5.90347438
C	25.09723211	-6.71107090	5.53760988
C	25.88207171	-7.90742414	6.06815780
C	27.27909123	-8.02036587	5.47057080
C	28.01788241	-9.25300903	6.02003551

C	27.32322931	-10.56512300	5.62640622
C	29.46259509	-9.26209865	5.53135486
C	22.97709503	-7.95225082	5.09380311
C	21.91629141	-6.98929261	8.05438110
C	22.03624298	-7.41649710	9.55237116
C	20.90815263	-7.93870313	7.40877310
C	19.49698634	-7.73996162	7.98339217
C	19.01813308	-6.29903089	7.72029300
C	17.55477864	-5.96241273	8.18427873
C	16.61464140	-7.02838083	7.56315627
O	14.73413502	-3.74675311	10.20555919
H	19.00600261	-6.23115179	6.61824321
H	19.92747265	-5.07284501	9.27843069
H	21.53174616	-5.33989239	6.76463478
H	16.34074502	-2.63271385	9.64524338
H	16.72469866	-4.66620459	11.38937703
H	17.99234187	-4.00377841	10.37044148
H	16.45272547	-6.57729374	9.98083903
H	18.16737707	-6.39435900	10.25737984
H	15.55184176	-3.18941596	7.49653070
H	15.08802316	-4.80214397	7.94352386
H	17.86475829	-2.63519803	7.07830899
H	20.06684216	-3.04099361	8.03160166
H	20.06128382	-3.83939971	6.46515006
H	22.46058637	-4.71818867	9.59884186
H	22.60461514	-3.69696786	8.16966687

H	24.46871609	-5.70137503	8.98706317
H	24.43315008	-4.89444009	7.42870658
H	23.96038604	-7.69557610	7.86099617
H	23.13835093	-5.87310542	5.51703906
H	25.19286419	-6.65516626	4.44482867
H	25.54461666	-5.78399612	5.91121537
H	25.32268628	-8.81997462	5.85204688
H	25.97792726	-7.83202555	7.15648346
H	27.84734688	-7.11494180	5.71335762
H	27.21560180	-8.07331220	4.37661307
H	28.03110747	-9.19551674	7.11545532
H	27.13900042	-10.60782678	4.54719095
H	26.36191623	-10.67870805	6.13852485
H	27.93268826	-11.43023085	5.90458462
H	29.51242608	-9.32543232	4.43894230
H	30.00691664	-10.11461237	5.94779380
H	29.98238475	-8.35201998	5.84619529
H	22.96666690	-8.88900258	5.65831227
H	23.52637916	-8.13161725	4.16227487
H	21.96172598	-7.71146058	4.76923508
H	22.82783556	-6.89628337	10.09904557
H	22.27715067	-8.48480455	9.61889277
H	21.11279103	-7.24495329	10.11237572
H	21.22105313	-8.98216060	7.51940042
H	20.81535967	-7.73972207	6.34308726
H	19.44447035	-7.99375837	9.04441847

H	18.84992052	-8.45293483	7.45920743
H	16.80973197	-8.02151934	7.98826763
H	16.74274105	-7.09521267	6.47734893
H	15.55794093	-6.83323030	7.76202637
H	14.17380390	-3.07227686	9.77978408

AmB-Chol15

0 1

C	7.61293000	7.96043500	-1.10472500
H	8.40739500	7.20234600	-1.24073300
C	7.76261000	8.50740900	0.32600500
H	6.99784400	9.29517700	0.48661800
C	7.51316100	7.39797500	1.35575300
H	8.25398600	6.58596400	1.18967600
C	6.04332600	6.26883700	-0.13300400
H	6.79371400	5.45088400	-0.22769700
C	6.25588000	7.26288400	-1.28732700
H	6.25629900	6.67911300	-2.23249500
C	7.59271400	7.88215000	2.79124200
H	6.82885500	8.65398100	2.97346000
H	8.58443700	8.31634200	2.97977500
H	7.42637400	7.04715900	3.48722500
C	4.23302500	4.91722400	0.79960900
H	4.14247800	5.52281900	1.71737300

C	2.88001100	4.48478500	0.32928100
H	2.84431400	3.96443900	-0.63374300
C	1.73025700	4.70407000	1.01452300
H	1.77254400	5.22781100	1.97894900
C	0.43852400	4.29170400	0.53623900
H	0.41081100	3.78806700	-0.43749800
C	-0.75075300	4.49245700	1.18899700
H	-0.74585300	4.99291800	2.16645600
C	-2.00748500	4.07959000	0.64985900
H	-1.97480100	3.57906800	-0.32613900
C	-3.22769500	4.23188700	1.26544600
H	-3.27134400	4.70872400	2.25353100
C	-4.45319200	3.78449800	0.69015500
H	-4.39731100	3.32470300	-0.30571900
C	-5.68798200	3.86988200	1.29253200
H	-5.75365000	4.31499100	2.29408400
C	-6.89820300	3.40726400	0.69810000
H	-6.82882700	2.98465500	-0.31341200
C	-8.13344700	3.44562300	1.30263000
H	-8.20585500	3.85600900	2.31848000
C	-9.33855000	2.97553500	0.69711000
H	-9.26590100	2.59410100	-0.33010600
C	-10.56655000	2.95253400	1.30726700
H	-10.65186200	3.32163500	2.33774100
C	5.20586200	3.75689100	1.09171000
H	6.14017800	4.17781800	1.49214300

H	4.77412000	3.14167600	1.89774000
C	-11.75581700	2.43513100	0.68632600
H	-11.65149100	2.10013200	-0.35387300
C	-12.95856600	2.28991000	1.29932300
H	-13.06456200	2.61979600	2.34299100
C	-14.17985500	1.68206700	0.66825200
H	-13.96207400	1.45975700	-0.39209900
C	-14.55220000	0.33958500	1.35101500
H	-14.93229000	0.58112900	2.36689400
C	-13.36648900	-0.63824900	1.55017500
H	-12.63152300	-0.07367900	2.15122800
C	-12.63595000	-0.97957000	0.23892300
H	-12.48846900	-0.06677700	-0.35076800
C	-10.23597600	-0.96361800	-0.05672400
C	-8.91479400	-1.48671700	0.46991100
H	-9.02519500	-1.76842700	1.52748700
H	-8.67290500	-2.40460500	-0.09598100
C	-7.77576500	-0.46917700	0.28571400
H	-8.02897500	0.45430000	0.84486700
C	-6.43971200	-1.01013800	0.79538800
H	-6.54694800	-1.24844000	1.86756600
H	-6.19892100	-1.94976300	0.26602600
C	-5.27028700	-0.03099500	0.62540000
H	-5.54164400	0.94005100	1.08626300
C	-3.99067800	-0.54668500	1.28958100
H	-3.81195100	-1.58865100	0.96589100

H	-4.17912300	-0.58368600	2.37511700
C	-2.74034100	0.31626200	1.00632600
H	-3.03589300	1.34536000	0.74538400
H	-2.13615500	0.39935500	1.92513700
C	-1.80629400	-0.23938400	-0.07706900
H	-1.42532100	-1.22364800	0.26100700
C	-0.58434900	0.69887600	-0.31823100
H	-0.39743200	1.30053100	0.58898200
C	0.69512100	-0.06433200	-0.68141400
H	0.52172000	-0.67873900	-1.58320800
H	0.93443900	-0.75191600	0.14964100
C	1.89037800	0.86770800	-0.90523000
H	2.00159000	1.52337100	-0.01885700
C	3.20232000	0.10660100	-1.10111000
H	3.12370400	-0.55316000	-1.98101200
H	3.37847300	-0.52242700	-0.21407400
C	4.42538600	1.01837800	-1.29726400
C	5.74273400	0.23186600	-1.33174300
H	5.78496700	-0.37773800	-2.24606600
H	5.78681700	-0.44334300	-0.46155700
C	6.94230400	1.17046600	-1.29219500
H	6.93287000	1.80004500	-2.19923900
C	6.84697600	2.08796400	-0.05362100
H	6.84783100	1.47182900	0.86161100
C	5.48382100	2.85082600	-0.10761800
H	5.45709600	3.43875300	-1.04479300

C	8.01829000	3.03555700	0.08171100
C	-13.25017500	-2.05819000	-0.63883300
H	-12.66021400	-2.17915300	-1.55921300
H	-14.27267900	-1.75840300	-0.91065900
H	-13.29200700	-3.02678600	-0.11935900
C	-13.78187600	-1.87490600	2.35728100
H	-14.55020700	-2.47261900	1.83976000
H	-14.18618500	-1.57812800	3.33830400
H	-12.92075600	-2.53586700	2.53603800
C	-15.37572600	2.65233000	0.71688700
H	-15.61290400	2.92605900	1.75897000
H	-16.26644700	2.18827100	0.27037400
H	-15.14402500	3.57788800	0.16983000
O	5.23776600	8.25911900	-1.31900800
H	4.40971400	7.80289100	-1.07672800
O	9.07720700	9.03132300	0.50222900
H	9.25629000	9.49975200	-0.33974700
O	6.18293600	6.87387500	1.14535200
O	4.73203900	5.78809000	-0.26830200
O	4.34926800	1.77131400	-2.49134700
H	3.40212500	2.01949400	-2.60114300
O	4.42575100	1.88488400	-0.13377900
O	8.43356500	3.50842700	1.12362900
O	8.59116300	3.35386100	-1.12843100
H	9.31900100	3.97494800	-0.91976000
O	1.68736400	1.69966200	-2.07210700

H	0.72769200	1.92390300	-2.08848900
O	-0.93183100	1.64064100	-1.35839500
H	-1.58709500	1.12731400	-1.89544000
O	-2.43640800	-0.42817200	-1.35312100
H	-3.40869600	-0.25184800	-1.25725300
O	-4.98262800	0.19978100	-0.77224100
H	-5.84501200	0.21938400	-1.24640100
O	-7.61054900	-0.14188900	-1.10897700
H	-8.50901000	0.07485600	-1.44715000
O	-10.33086600	-0.20827600	-1.02333600
O	-11.28017400	-1.42614400	0.64890500
O	-15.61962000	-0.23063200	0.56853200
H	-16.06126000	-0.90616300	1.10780200
O	8.12626600	0.35826400	-1.26510900
H	8.86701200	0.92890800	-1.53111100
N	7.88885900	9.06176900	-2.03754800
H	7.04911900	9.64454800	-2.11858000
H	8.09715600	8.70736800	-2.97365700
C	-1.35058400	-3.68796100	-3.49857300
C	-1.07995400	-3.65341100	-1.97045500
C	-0.75395300	-5.03150000	-1.32214200
C	-0.71768200	-4.89248200	-4.21029300
C	0.52757900	-5.18925700	-3.42940300
C	1.64412700	-4.48843300	-3.71215100
C	2.73188100	-4.23002500	-2.71460300
C	2.42562200	-4.72857900	-1.27772300

C	3.74324700	-4.85221300	-0.52068800
C	4.42735200	-3.57436400	-0.04202900
C	5.55337600	-4.10482000	0.86500400
C	5.28399600	-5.64117600	1.07239800
C	6.33574100	-6.46155400	0.22672000
C	7.78666800	-6.25378000	0.74770900
C	7.93424600	-6.67170600	2.20794000
C	9.38637200	-6.78097900	2.65618100
C	9.47969700	-7.22652400	4.12584500
C	8.90346500	-8.63410600	4.33985000
C	10.92881300	-7.17996000	4.59911000
C	6.08319600	-7.97574600	0.14314900
C	3.75484400	-5.73011800	0.73774600
C	2.95850100	-5.14058100	1.94569900
C	3.12202500	-7.08307400	0.41590200
C	1.68553700	-6.92484800	-0.10615300
C	1.68338300	-6.08733300	-1.39974000
C	0.28835500	-5.90122300	-2.09921100
C	-0.32209000	-7.31306200	-2.29922900
O	-2.75185000	-3.66898900	-3.74998000
H	2.28956600	-6.69235300	-2.09670500
H	1.78189900	-4.00333400	-0.76297900
H	4.42858900	-5.31946100	-1.23944100
H	-0.93956600	-2.76309400	-3.92665400
H	-1.92849500	-3.19936000	-1.44548500
H	-0.21903500	-2.99355200	-1.79095100

H	-1.70403800	-5.56939600	-1.23869400
H	-0.42101900	-4.85667500	-0.29219400
H	-0.50146700	-4.65444900	-5.26085600
H	-1.40109900	-5.73910100	-4.24596000
H	1.71565000	-3.92649500	-4.63946600
H	2.92611800	-3.14856900	-2.67676200
H	3.64484700	-4.69366400	-3.10961600
H	3.74007300	-2.93785500	0.53140200
H	4.81595300	-2.96711500	-0.86423500
H	5.55195300	-3.57912900	1.82730700
H	6.51518100	-3.89253300	0.39383100
H	5.41382300	-5.90157100	2.12805200
H	6.33724600	-6.08786200	-0.80518200
H	8.47393700	-6.83686600	0.11992400
H	8.10588000	-5.21289000	0.63001300
H	7.43943300	-7.63456800	2.35084000
H	7.42853700	-5.94477800	2.85226900
H	9.87259900	-5.80564600	2.53801600
H	9.92549000	-7.48629100	2.01161600
H	8.89940600	-6.52880700	4.74218800
H	9.33740800	-9.35151600	3.63452300
H	7.81628500	-8.64605500	4.20875100
H	9.10235900	-8.98896400	5.35560200
H	11.55807400	-7.86538100	4.02110700
H	11.00068800	-7.45854400	5.65443600
H	11.33663400	-6.16979900	4.49515400

H	5.60539500	-8.36314400	1.04753400
H	7.01326800	-8.53602200	-0.00747500
H	5.47112200	-8.22490700	-0.72724200
H	3.39521300	-4.22780300	2.36068500
H	2.93720100	-5.86498100	2.76943100
H	1.92462200	-4.89591400	1.68680300
H	3.13969400	-7.74505800	1.28791900
H	3.66456800	-7.58491500	-0.38265400
H	1.01726600	-6.50247900	0.64748200
H	1.32195200	-7.93806600	-0.31349800
H	-0.56479900	-7.77767600	-1.33469300
H	0.36702000	-7.97863000	-2.83026600
H	-1.26291400	-7.29636200	-2.85494600
H	-2.86204100	-3.53661300	-4.70941100

AmB-Chol16

0 1

C	-8.24584100	-3.66135400	3.27857800
H	-8.64265500	-4.47106000	2.63726300
C	-8.85851400	-2.34709600	2.76289600
H	-8.50460200	-1.51444300	3.40529600
C	-8.39232000	-2.06130400	1.32956600
H	-8.71418300	-2.90175800	0.67702700
C	-6.35106100	-3.22164100	1.70191600

H	-6.69245200	-4.01495100	0.99820700
C	-6.71601100	-3.64864800	3.13378500
H	-6.31433500	-4.67294000	3.28745500
C	-8.91815500	-0.74984600	0.77733700
H	-8.56286000	0.09034000	1.39399000
H	-10.01693600	-0.75756600	0.79163800
H	-8.57197200	-0.60271100	-0.25599700
C	-4.36411400	-2.53882700	0.45306700
H	-4.68787700	-1.48909600	0.35070700
C	-2.88782800	-2.60458200	0.68783100
H	-2.47493300	-3.59487700	0.90756400
C	-2.06240000	-1.52909900	0.64808100
H	-2.48282300	-0.53847200	0.42873400
C	-0.64775300	-1.61373600	0.89094900
H	-0.24658100	-2.60633200	1.12842900
C	0.22656400	-0.55747300	0.86538300
H	-0.15347400	0.44500600	0.62764500
C	1.62108200	-0.70181000	1.13922400
H	1.96695700	-1.71763700	1.36863300
C	2.54904600	0.31252300	1.10656100
H	2.22004500	1.32913300	0.85387100
C	3.93550000	0.11056100	1.37097700
H	4.24827300	-0.90759300	1.63899600
C	4.90510000	1.08501400	1.29906600
H	4.60582200	2.10342700	1.01833800
C	6.28723600	0.85464400	1.56103700

H	6.57625800	-0.16078800	1.86423900
C	7.27514400	1.80595900	1.45244200
H	6.99281300	2.81935200	1.13790300
C	8.65762000	1.56128200	1.71474500
H	8.93057400	0.55455900	2.05818000
C	9.66182400	2.48152900	1.55497900
H	9.40449500	3.48903300	1.20269100
C	-4.84461900	-3.30666400	-0.79499100
H	-5.92564800	-3.13920000	-0.91248100
H	-4.36480700	-2.85794300	-1.67986800
C	11.05127300	2.19402400	1.78822300
H	11.27997600	1.18995800	2.16857800
C	12.07971700	3.04190900	1.52966700
H	11.85386900	4.04652700	1.14361000
C	13.53512600	2.71106800	1.70742400
H	13.62133000	1.70800100	2.16307700
C	14.26711600	2.65810400	0.34051200
H	14.32813200	3.70003000	-0.04091900
C	13.54070200	1.83511600	-0.75344800
H	12.54655400	2.30691500	-0.84964000
C	13.25692000	0.38251000	-0.33039300
H	12.87781200	0.36422900	0.69850000
C	11.13963700	-0.75339500	-0.60310500
C	10.05343800	-1.13962900	-1.58671800
H	10.06695200	-0.44716800	-2.44131100
H	10.29701400	-2.14704700	-1.97018000

C	8.66373700	-1.17626700	-0.92786200
H	8.42945000	-0.16663700	-0.53374000
C	7.57420700	-1.59050300	-1.91719300
H	7.56527200	-0.86519600	-2.74888000
H	7.82046900	-2.58141100	-2.33975100
C	6.16938500	-1.64842600	-1.30213900
H	5.95115700	-0.68090800	-0.80691400
C	5.09236300	-1.91079800	-2.35838700
H	5.39639000	-2.77668500	-2.97500500
H	5.07591900	-1.03747200	-3.03092000
C	3.68069700	-2.14701800	-1.77559100
H	3.59529900	-1.68509900	-0.77868400
H	2.93309400	-1.64017200	-2.40832800
C	3.25448500	-3.61912100	-1.69692400
H	3.23107700	-4.03172600	-2.72523300
C	1.83134700	-3.77268400	-1.07826700
H	1.26067900	-2.83948100	-1.23135000
C	1.04088700	-4.94293900	-1.67585200
H	1.60331500	-5.88399500	-1.53842600
H	0.93864500	-4.77116600	-2.76246900
C	-0.35895700	-5.07644800	-1.06754100
H	-0.87846200	-4.10130900	-1.15202200
C	-1.21050000	-6.13157100	-1.77495600
H	-0.72001300	-7.11649900	-1.70401300
H	-1.28888300	-5.86218500	-2.84023800
C	-2.63267500	-6.26558400	-1.20472500

C	-3.50908200	-7.21424000	-2.03367800
H	-3.14079700	-8.24495700	-1.92702800
H	-3.44719500	-6.93140900	-3.09728000
C	-4.96411000	-7.14682600	-1.58663400
H	-5.03269000	-7.49501500	-0.54104800
C	-5.46428500	-5.68731700	-1.65455900
H	-5.39471100	-5.32251600	-2.69347800
C	-4.52096600	-4.80051400	-0.77889000
H	-4.55215900	-5.18693700	0.25763000
C	-6.91504200	-5.53188200	-1.25534600
C	14.39332500	-0.61742500	-0.47132000
H	14.07832800	-1.60373200	-0.10015900
H	15.24972000	-0.27159500	0.12556000
H	14.71179900	-0.72235900	-1.51886100
C	14.24734800	1.94879100	-2.11028900
H	15.26904200	1.53536500	-2.08497200
H	14.31517900	3.00295300	-2.42348500
H	13.69433900	1.40297800	-2.88914700
C	14.22779600	3.72126700	2.64208600
H	14.14247200	4.74523700	2.24043400
H	15.29479400	3.47936500	2.74747600
H	13.75982900	3.70967100	3.63731700
O	-6.17899600	-2.75359700	4.10360000
H	-5.29907100	-2.49467800	3.77012100
O	-10.28129400	-2.44042200	2.78922800
H	-10.46933700	-2.89722000	3.63562900

O	-6.94903900	-1.98791600	1.32761700
O	-4.95563700	-3.07575200	1.68175300
O	-2.63595200	-6.73976000	0.12707300
H	-1.85747100	-6.32772500	0.56857400
O	-3.18546900	-4.92660200	-1.28266500
O	-7.66660500	-4.65276600	-1.63476700
O	-7.33190200	-6.49772100	-0.36803200
H	-8.26899100	-6.28984500	-0.17388300
O	-0.28349900	-5.43945900	0.33156400
H	0.49831500	-4.96469400	0.69883500
O	1.97100000	-3.92623200	0.35230100
H	2.86144000	-4.35304700	0.43128000
O	4.13272300	-4.44214000	-0.91441000
H	4.92379100	-3.90308900	-0.65123800
O	6.07866200	-2.69299500	-0.30712600
H	6.93702100	-2.71016000	0.17437500
O	8.64541300	-2.12044000	0.16199000
H	9.43445800	-1.91640400	0.71366900
O	11.11026900	-1.04712800	0.59135800
O	12.13131600	-0.06459900	-1.18989900
O	15.59705200	2.17371400	0.61152400
H	16.15863300	2.39257800	-0.14934700
O	-5.72154500	-8.01138500	-2.44745000
H	-6.56280900	-8.19405600	-1.99578400
N	-8.75071800	-3.88832800	4.63976000
H	-8.20467000	-3.31830300	5.29417800

H	-8.62755500	-4.86388600	4.91976700
C	1.44327300	3.47019000	1.01189900
C	0.83684200	4.80804800	0.50994700
C	0.04601200	4.71816200	-0.82879400
C	0.72544100	2.22552700	0.47013200
C	-0.70243700	2.64996900	0.29822100
C	-1.51889100	2.60305500	1.37012700
C	-2.71605900	3.48871400	1.53589800
C	-2.88585300	4.56307000	0.42959600
C	-4.33465200	5.03785300	0.43882400
C	-4.78453400	5.97227100	1.55886000
C	-6.18598700	6.41404700	1.09757500
C	-6.35034100	5.92458400	-0.38882300
C	-7.33873900	4.69298900	-0.41332500
C	-8.77784100	5.09642900	0.01796400
C	-9.36113600	6.18073800	-0.88356600
C	-10.86042000	6.37512400	-0.69411800
C	-11.40148300	7.46084300	-1.64047800
C	-11.24748800	7.06806400	-3.11734600
C	-12.86637900	7.74875200	-1.32859100
C	-7.44509500	3.95475200	-1.75765900
C	-4.85290200	5.74820500	-0.81868200
C	-4.24580500	7.16791900	-1.05682700
C	-4.50629600	4.91273500	-2.05009900
C	-2.99259100	4.67381800	-2.16274800
C	-2.48466100	3.91298600	-0.92260800

C	-0.96151500	3.52567100	-0.92634400
C	-0.67881800	2.76865500	-2.25033400
O	2.82660700	3.40323200	0.68172800
H	-3.03674700	2.95765800	-0.96522900
H	-2.22661200	5.41614400	0.63692800
H	-4.92304500	4.11784500	0.54837500
H	1.37927800	3.47084700	2.10892800
H	1.62138400	5.56785700	0.41528000
H	0.14240500	5.17767400	1.27803000
H	0.79179600	4.64587500	-1.62725300
H	-0.46806000	5.67326500	-0.98913000
H	0.82644500	1.38312400	1.16816300
H	1.17397000	1.87924300	-0.45938600
H	-1.23282200	2.03247000	2.24955900
H	-2.64801100	3.99521800	2.50931000
H	-3.59418100	2.83263600	1.59038600
H	-4.11941900	6.84127900	1.65249600
H	-4.81092200	5.48208000	2.53600100
H	-6.28495300	7.50441400	1.15830100
H	-6.92973900	5.99392700	1.77762700
H	-6.78378400	6.72509300	-0.99741100
H	-7.00733800	3.95142200	0.32472800
H	-9.41730700	4.20376200	-0.00791500
H	-8.80059800	5.43390800	1.05949700
H	-9.16020000	5.91949700	-1.92459600
H	-8.86197400	7.13466000	-0.68307300

H	-11.05609800	6.66481100	0.34484500
H	-11.38579200	5.42736100	-0.86531200
H	-10.83251800	8.38450700	-1.47754200
H	-11.66241400	6.07208600	-3.30796200
H	-10.19569000	7.06046500	-3.42223000
H	-11.75927400	7.78299200	-3.76864300
H	-13.48753500	6.85945700	-1.48083800
H	-13.24954300	8.54673300	-1.97116300
H	-12.98320000	8.07508500	-0.29060800
H	-7.30123100	4.62719200	-2.60819400
H	-8.42010100	3.46876400	-1.87935200
H	-6.72359700	3.13592900	-1.81482100
H	-4.53544700	7.90539200	-0.30294700
H	-4.59381300	7.56759600	-2.01749900
H	-3.15243900	7.15888300	-1.07310200
H	-4.88737500	5.37948200	-2.96436100
H	-4.94973400	3.92135800	-1.98357500
H	-2.43927300	5.60239000	-2.31940000
H	-2.84484800	4.06636600	-3.06328600
H	-0.78906100	3.43443700	-3.11617700
H	-1.36262300	1.92366200	-2.38542900
H	0.34204100	2.38358400	-2.31211600
H	3.18813500	2.62729700	1.14798600

AmB-Ret

AmB-Ret1

0 1

C	6.55314500	3.65627800	1.14489100
C	7.96909900	4.18102800	0.90258300
C	9.00399300	3.35785800	1.71384300
C	8.76373500	1.85409300	1.56978900
C	7.37385600	1.48070400	2.09582600
C	6.29522600	2.43720900	1.65720900
H	10.02759800	3.61640400	1.38943800
H	8.85708600	1.55611500	0.50736400
H	9.54027500	1.28833800	2.11699700
H	7.12205100	0.44730700	1.77890300
H	7.38558200	1.45789900	3.20655000
H	8.94358100	3.64185400	2.78219600
C	4.90788600	1.91536500	1.87734500
H	4.14860700	2.46280100	1.30227000
H	4.81423900	0.85711900	1.59672000
H	4.62547800	1.99769400	2.93592300
C	8.28410900	4.07893700	-0.60400300
H	7.60910700	4.70218600	-1.19865100
H	9.30566700	4.40550100	-0.82043100
H	8.17846500	3.04871300	-0.95931500
C	8.07275300	5.65267900	1.35070700
H	9.09962400	6.02270600	1.27893000

H	7.44794600	6.30895300	0.73516000
H	7.74241400	5.77550700	2.38764000
C	5.48023500	4.57476900	0.72031300
H	5.49025700	4.82050900	-0.34531000
C	4.57379700	5.09939600	1.55709200
H	4.59906400	4.87459700	2.62595500
C	3.49586100	6.01144900	1.13679700
C	2.82509500	5.82002700	-0.01910200
H	3.09197800	4.97835700	-0.66399500
C	3.20575200	7.11477200	2.10501900
H	2.71271100	6.72564200	3.00760100
H	4.12897800	7.61561400	2.42960900
H	2.54857700	7.88786500	1.68400900
C	1.72823900	6.65237600	-0.49854100
H	1.43424500	7.48101400	0.15254900
C	1.10300100	6.41818800	-1.66568500
H	1.41155400	5.57987900	-2.29699400
C	-0.02170400	7.20602300	-2.19696200
C	-0.76022400	6.70771500	-3.20541200
H	-0.54475200	5.72791100	-3.63615900
C	-0.29683000	8.53775600	-1.56989200
H	-0.77445700	8.41418900	-0.58742700
H	0.62814200	9.11177900	-1.42043300
H	-0.96438400	9.16452300	-2.17648300
C	-1.94326400	7.37815700	-3.82582600
H	-1.85315600	8.48110300	-3.85916400

H	-2.88833100	7.09992300	-3.31495000
O	-2.16171100	6.90094100	-5.16521100
H	-1.36067300	7.03567400	-5.70807700
C	-10.80747300	-1.23948400	4.06600400
H	-11.75664000	-0.80460400	3.65488300
C	-10.15017100	-0.19476700	5.00423300
H	-9.32748900	-0.64890900	5.59941300
C	-9.66558700	1.04429700	4.21240900
H	-10.51743900	1.55337800	3.71132000
C	-9.26189500	-0.33208400	2.23293000
H	-9.94692100	0.20605500	1.54528600
C	-9.85892800	-1.59843300	2.88961100
H	-10.38692100	-2.21712000	2.12642500
C	-8.85199700	1.99851500	5.06724400
H	-7.89965100	1.54785600	5.37693100
H	-9.41237000	2.26680500	5.97542700
H	-8.61051700	2.91827600	4.52242600
C	-7.27071600	0.19251400	0.91392700
H	-7.19504900	1.07127700	1.59534000
C	-5.96544900	-0.53069600	0.73161100
H	-6.04118200	-1.46494900	0.16646000
C	-4.80612400	-0.06834700	1.21699300
H	-4.75581300	0.85876900	1.79388900
C	-3.54051500	-0.76817500	1.00414100
H	-3.58667400	-1.67830900	0.39198500
C	-2.37105400	-0.34643200	1.51845700

H	-2.30876800	0.54590800	2.14404700
C	-1.12255700	-1.06025700	1.26595400
H	-1.19681600	-1.91536600	0.57520900
C	0.05207600	-0.71558700	1.82419200
H	0.12748200	0.11896500	2.52393000
C	1.28684900	-1.43706300	1.53299000
H	1.22450500	-2.20237100	0.74406000
C	2.44854100	-1.19837800	2.16951600
H	2.52153600	-0.44835900	2.95924900
C	3.66835800	-1.93378300	1.85124600
H	3.58152400	-2.68205300	1.05306600
C	4.83790100	-1.72910600	2.48563700
H	4.92648600	-0.99988600	3.29402000
C	6.04896800	-2.46488900	2.13853300
H	5.93076700	-3.27845400	1.41107900
C	7.26034100	-2.18733400	2.65329000
H	7.40683900	-1.39864700	3.39262400
C	-7.91707100	0.59082300	-0.42202600
H	-8.82591700	1.19719700	-0.22510800
H	-7.22027700	1.25441900	-0.98030300
C	8.45184800	-2.92543200	2.23380100
H	8.25526500	-3.88302000	1.73239200
C	9.69935000	-2.46940300	2.40571200
H	9.90693500	-1.52421000	2.90560800
C	10.91393400	-3.22160400	1.92959000
H	10.64078000	-4.26843200	1.63094200

C	11.52576700	-2.52582500	0.68513900
H	12.05280400	-1.58940800	0.97715000
C	10.51089900	-2.21165800	-0.44290700
H	9.70630500	-1.56378400	0.00289300
C	9.81289900	-3.48433800	-0.95790300
H	9.41162000	-4.09091500	-0.11044700
C	7.39672000	-3.30837900	-1.35111200
C	6.38223800	-2.68697800	-2.26647300
H	6.77837600	-1.75288600	-2.71838700
H	6.21607600	-3.37751200	-3.12643400
C	5.05059400	-2.42976100	-1.53386400
H	5.21124200	-1.78690700	-0.63621300
C	3.99212600	-1.85460300	-2.47975600
H	4.39846200	-0.97364400	-3.01270100
H	3.75737000	-2.60630500	-3.26606200
C	2.69166800	-1.47613400	-1.75169600
H	2.89264300	-0.88667300	-0.82602900
C	1.72932600	-0.72878600	-2.69226700
H	1.70246000	-1.23480100	-3.68021000
H	2.13076800	0.28496500	-2.88425800
C	0.30802100	-0.62643200	-2.12469600
H	0.30658500	-0.89742400	-1.04368900
H	-0.04452000	0.42187400	-2.16292100
C	-0.67459600	-1.53090000	-2.87776500
H	-0.73829500	-1.25648700	-3.95480100
C	-2.08464300	-1.55315100	-2.21248300

H	-2.27222800	-0.66228500	-1.57197200
C	-3.18871300	-1.74596800	-3.26010400
H	-2.98756700	-2.67322800	-3.83941700
H	-3.16997100	-0.91752900	-3.99185700
C	-4.57597800	-1.85131800	-2.61079500
H	-4.75677700	-1.02935000	-1.87660500
C	-5.70163200	-1.91482800	-3.65347400
H	-5.59711300	-2.84105000	-4.25783100
H	-5.61559800	-1.06104900	-4.35176000
C	-7.08907900	-1.91830800	-2.99971900
C	-8.26202700	-1.81713600	-3.97842500
H	-8.33988200	-2.74435300	-4.58340900
H	-8.09590500	-0.99561600	-4.70556800
C	-9.58330700	-1.58589500	-3.24161300
H	-9.94677100	-2.51182200	-2.74276200
C	-9.48510500	-0.41738000	-2.21741600
H	-9.33293000	0.52873900	-2.80919800
C	-8.25485200	-0.62447900	-1.28791600
H	-8.37969000	-1.55687800	-0.68479100
C	-10.74094400	-0.25899200	-1.41450400
C	10.57912000	-4.34513500	-1.93697600
H	10.02694100	-5.25944400	-2.18821100
H	11.54324500	-4.64992200	-1.49566800
H	10.78394500	-3.82402700	-2.87981200
C	11.17867100	-1.41298600	-1.56273700
H	12.05191800	-1.92769300	-1.97829700

H	11.50307400	-0.42535100	-1.21689700
H	10.47363400	-1.25460800	-2.39155900
C	11.95924600	-3.31120700	3.04573600
H	12.26981400	-2.32727900	3.41039100
H	12.85866200	-3.83074900	2.68475400
H	11.57114900	-3.87379300	3.90255900
O	-8.84490200	-2.46537600	3.40287500
H	-7.95184900	-2.03618600	3.32183200
O	-11.13497600	0.33105100	5.90552600
H	-11.63020300	-0.41634000	6.32195000
O	-8.72940500	0.59769200	3.19764400
O	-8.10419200	-0.82142600	1.56762000
O	-7.33293100	-3.06166200	-2.21040800
H	-6.51614200	-3.34542900	-1.69555600
O	-7.08332200	-0.77163000	-2.11943900
O	-10.93466000	0.30916600	-0.36286900
O	-11.81913300	-0.86293100	-2.03357800
H	-12.66867200	-0.75030800	-1.52840600
O	-4.68256900	-3.09862100	-1.89198200
H	-3.88299400	-3.23028300	-1.30417300
O	-2.12442300	-2.64251700	-1.27433100
H	-1.62260700	-3.42623900	-1.66781800
O	-0.25384300	-2.90422400	-2.89011800
H	0.55176500	-3.03640700	-2.29108100
O	1.99377300	-2.65799400	-1.29780100
H	2.62322500	-3.42037100	-1.16531500

O	4.49464100	-3.67873700	-1.08561800
H	5.14229100	-4.17698500	-0.51425800
O	7.16767000	-4.03362200	-0.40008000
O	8.64949900	-2.95654300	-1.73119700
O	12.51964600	-3.46703800	0.22767400
H	13.02495700	-3.08589400	-0.51743500
O	-10.49017900	-1.20977400	-4.28584300
H	-11.42260200	-1.23108400	-3.96595800
N	-11.15643000	-2.42482300	4.89119000
H	-10.30188300	-2.91763800	5.16623600
H	-11.70596900	-3.08638700	4.34819400

AmB-Ret2

0 1

C	4.28779000	0.82998200	3.97177100
C	5.08065800	-0.14318700	3.08750700
C	6.29297400	-0.71271200	3.84388500
C	5.95585000	-1.10483300	5.27434900
C	5.49929500	0.12643500	6.04499200
C	4.48522200	0.95859400	5.29393200
H	6.69805100	-1.56352500	3.28622200
H	5.15796200	-1.85576000	5.27847000
H	6.81995200	-1.56468000	5.76465400
H	5.07465900	-0.16154400	7.01441200

H	6.36551200	0.76158300	6.28217000
H	7.07864400	0.05413800	3.87122000
C	3.72726900	1.91373500	6.17670900
H	2.98892100	2.50283300	5.63581200
H	3.21630900	1.36489900	6.97665900
H	4.41956800	2.60569700	6.67159500
C	4.16593800	-1.28965000	2.61908800
H	3.27259100	-0.91226400	2.11451900
H	4.69113300	-1.92002600	1.89644100
H	3.83798700	-1.91277700	3.45490700
C	5.59762700	0.60220500	1.84772000
H	6.22976100	-0.05592900	1.24872900
H	4.77307800	0.92874200	1.21504400
H	6.17506200	1.48703700	2.13332000
C	3.26663900	1.62291200	3.24567700
H	2.44229100	1.06563700	2.80024100
C	3.33412500	2.94037800	3.03126200
H	4.21338000	3.47289700	3.39319500
C	2.35032500	3.74898600	2.29395800
C	1.03408400	3.47813500	2.38155100
H	0.73665100	2.65025900	3.02067800
C	2.94686900	4.83827100	1.44592700
H	3.62710000	5.46022500	2.03736400
H	3.54024600	4.38944300	0.64162800
H	2.20045000	5.49097600	0.99205100
C	-0.05726100	4.18145900	1.73592900

H	0.16205500	5.13134400	1.25621600
C	-1.31539400	3.70818000	1.72787500
H	-1.50005200	2.73001700	2.16731000
C	-2.48755900	4.35645300	1.14174800
C	-3.63651200	3.66173000	1.06737100
H	-3.63062100	2.63373700	1.41207400
C	-2.33741900	5.76624200	0.63289000
H	-1.92922300	6.41651300	1.41196600
H	-1.63443800	5.80144200	-0.20665800
H	-3.28312800	6.19530000	0.29691800
C	-4.92467000	4.15053700	0.47092600
H	-5.17468600	5.15154600	0.84905000
H	-5.73957300	3.47854400	0.75018700
O	-4.92093200	4.16231400	-0.96139600
H	-4.08210900	4.54457000	-1.24389000
C	9.79447500	-2.76227500	0.82693800
H	10.42664900	-1.99833800	0.35743100
C	9.86623900	-4.00650900	-0.05690400
H	9.25210600	-4.79491100	0.40601100
C	9.27639900	-3.71292700	-1.43090700
H	9.86296600	-2.90084600	-1.89132100
C	7.80915000	-2.09250100	-0.52702700
H	8.33339700	-1.28309200	-1.05124400
C	8.36342800	-2.23747200	0.88881600
H	8.34347500	-1.24537000	1.36553200
C	9.26732800	-4.92316300	-2.33614500

H	8.65021800	-5.71006000	-1.89426000
H	10.28445400	-5.29943200	-2.45586100
H	8.85490100	-4.66717500	-3.31465200
C	5.60854900	-1.87832100	-1.53676500
H	5.63423600	-2.90708500	-1.91029100
C	4.23988400	-1.56865500	-1.01581100
H	4.11853500	-0.60635000	-0.52743900
C	3.21207700	-2.42041700	-1.08843600
H	3.34031800	-3.37947900	-1.59024000
C	1.90546800	-2.13306100	-0.52877000
H	1.81798600	-1.22342200	0.06238700
C	0.80857100	-2.89489400	-0.70324700
H	0.87305400	-3.80316800	-1.30193500
C	-0.48637900	-2.53067000	-0.17672900
H	-0.52510900	-1.64119300	0.44664700
C	-1.64391500	-3.17070100	-0.43931600
H	-1.63727400	-4.06524500	-1.06148000
C	-2.91944600	-2.69950300	0.04411600
H	-2.90197100	-1.77940800	0.62350100
C	-4.10509900	-3.29190700	-0.20340100
H	-4.13147400	-4.20702200	-0.79437000
C	-5.36709500	-2.78297500	0.27636200
H	-5.33316200	-1.86094100	0.85555700
C	-6.55839300	-3.36751700	0.03920900
H	-6.58626300	-4.27995200	-0.55580500
C	-7.82438400	-2.86845400	0.52194900

H	-7.79924600	-1.97717600	1.14819000
C	-9.01439900	-3.42596900	0.23064900
H	-9.04141100	-4.30692100	-0.41013000
C	6.11029600	-0.97496200	-2.66984000
H	7.10409100	-1.33018000	-2.95344700
H	5.45476600	-1.12555000	-3.53358500
C	-10.28773400	-2.90750500	0.69058000
H	-10.24410200	-2.06576800	1.37899000
C	-11.47843800	-3.36515300	0.28652800
H	-11.51220900	-4.19923100	-0.41672800
C	-12.80821800	-2.79998000	0.69538800
H	-12.65144400	-2.04852100	1.47729500
C	-13.49922400	-2.09295400	-0.48677100
H	-13.79328200	-2.87337300	-1.20830700
C	-12.60956000	-1.09727300	-1.25308100
H	-11.74460400	-1.67354600	-1.59542700
C	-12.03455400	0.00185800	-0.35354400
H	-11.68793600	-0.43175000	0.58040700
C	-9.71734000	0.59497100	-0.36368100
C	-8.56888500	1.10291500	-1.19901000
H	-8.75819800	0.87985100	-2.25063400
H	-8.53341200	2.19295300	-1.08944100
C	-7.23442900	0.51559600	-0.73685300
H	-7.27485600	-0.57856500	-0.83296600
C	-6.06619600	1.05560600	-1.54642900
H	-6.25327900	0.85216300	-2.60624200

H	-6.00842900	2.14204000	-1.42171300
C	-4.71375400	0.44305600	-1.17488600
H	-4.72661900	-0.63506600	-1.39154000
C	-3.59947300	1.10740000	-1.97552700
H	-3.64587500	2.18269000	-1.77490100
H	-3.84918300	0.97045400	-3.03366900
C	-2.17663800	0.58669800	-1.74190500
H	-2.16284900	-0.50755400	-1.68834800
H	-1.57417900	0.86115000	-2.61571100
C	-1.45855800	1.13625500	-0.51007400
H	-1.61718600	2.22156800	-0.45033400
C	0.05231300	0.85404400	-0.58928600
H	0.19836100	-0.09350400	-1.12358100
C	0.83879300	1.94071400	-1.30849000
H	0.69348700	2.89576800	-0.79174400
H	0.45459800	2.04297900	-2.32951200
C	2.33048100	1.62127100	-1.36283300
H	2.46737500	0.60261200	-1.74842700
C	3.10405100	2.57635500	-2.25973400
H	2.90139100	3.61128800	-1.96609100
H	2.76615200	2.44100500	-3.29118600
C	4.61924700	2.36443300	-2.20122700
C	5.35256700	3.15867400	-3.26970700
H	5.23353700	4.22928700	-3.09072900
H	4.93050000	2.92212800	-4.25036100
C	6.83058200	2.81875800	-3.29745800

H	7.29023700	3.15138400	-2.35576700
C	7.01403000	1.28048500	-3.40975800
H	6.70072800	1.00535100	-4.42367300
C	6.15687100	0.52105700	-2.37937600
H	6.55877700	0.70580300	-1.37763500
C	8.47445700	0.94525100	-3.26094300
C	-12.93704100	1.18166900	-0.05644300
H	-12.44211600	1.86473000	0.63766000
H	-13.85980500	0.81650600	0.39668400
H	-13.18063100	1.73241800	-0.96744500
C	-13.32162500	-0.54194600	-2.48482300
H	-14.23796700	-0.00620800	-2.21905100
H	-13.58889100	-1.34842000	-3.17377800
H	-12.67754900	0.15878300	-3.02113400
C	-13.72354600	-3.88910300	1.26281100
H	-13.88053000	-4.68464600	0.52592600
H	-14.69563000	-3.46901900	1.52484700
H	-13.27957500	-4.34124400	2.15252300
O	7.59236400	-3.15663400	1.63739800
H	6.68633600	-3.07997700	1.30376000
O	11.20725400	-4.41155900	-0.20280500
H	11.58624300	-4.30114900	0.68428400
O	7.92507800	-3.29030600	-1.26182000
O	6.45049300	-1.80423300	-0.37205500
O	5.15640600	2.74333200	-0.96542700
H	4.56281900	2.36792700	-0.28913000

O	4.81295200	0.97487100	-2.44887900
O	8.99701600	0.35685700	-2.34464400
O	9.21892200	1.43049400	-4.28550400
H	10.14220300	1.20475600	-4.08470900
O	2.90681100	1.70570800	-0.05965800
H	2.24445900	1.41350900	0.58954700
O	0.58586700	0.70388900	0.72793000
H	-0.18215200	0.55972200	1.30749500
O	-1.89707000	0.53577300	0.69976600
H	-2.88024400	0.55469600	0.70708100
O	-4.45129600	0.60921400	0.22280900
H	-5.30401700	0.65117100	0.69915000
O	-7.00345200	0.85961300	0.63229000
H	-7.81052300	0.61532200	1.12211500
O	-9.60646200	0.30876500	0.81888600
O	-10.84928400	0.50525300	-1.04619200
O	-14.66430700	-1.47010600	0.04590100
H	-15.26523400	-1.26741100	-0.67905600
O	7.37117000	3.50331500	-4.40671000
H	8.28105700	3.20777600	-4.53665100
N	10.38081700	-3.10093600	2.12022000
H	9.68899200	-3.62051700	2.65591700
H	10.59381700	-2.26173400	2.64993100

AmB-Ret3

0 1

C	-3.27957700	7.45177900	-0.28796400
C	-1.93559500	7.07854900	-0.91563800
C	-1.25538200	8.32958900	-1.53095300
C	-1.27760600	9.51288100	-0.56189700
C	-2.72052300	9.90096700	-0.22247000
C	-3.60936600	8.71376200	0.04818000
H	-0.21675600	8.09012200	-1.81978500
H	-0.72877900	9.25399500	0.36491800
H	-0.74609600	10.37750900	-1.00036800
H	-2.72395600	10.58801500	0.64727500
H	-3.16171300	10.47931800	-1.06218900
H	-1.77626800	8.60809800	-2.46719600
C	-4.90517600	9.08245200	0.70457900
H	-5.49057200	8.20943600	1.02520100
H	-4.74205800	9.69578900	1.60071700
H	-5.54260600	9.66014900	0.02171400
C	-1.03329800	6.47541400	0.18037000
H	-1.46381900	5.55980400	0.59733100
H	-0.04265900	6.22583300	-0.20938800
H	-0.89934900	7.17918200	1.00945900
C	-2.13969800	6.04449100	-2.04134300
H	-1.20402000	5.83584700	-2.56757800
H	-2.51485000	5.09158600	-1.65233100
H	-2.86909500	6.39944000	-2.77712400

C	-4.16977400	6.30490800	-0.02526700
H	-3.77284300	5.57786500	0.68830600
C	-5.35657900	6.13760500	-0.62624000
H	-5.71720200	6.86626600	-1.35718600
C	-6.25814700	4.99719400	-0.39025500
C	-6.37347500	4.41957000	0.82447300
H	-5.77999800	4.79704100	1.66171400
C	-7.04052900	4.57422000	-1.59518200
H	-7.78702000	5.33502700	-1.86494100
H	-6.38603300	4.43542800	-2.46690700
H	-7.58283000	3.63122600	-1.44067000
C	-7.26478200	3.30908600	1.14103600
H	-7.84163100	2.90013100	0.30539100
C	-7.39608800	2.81436200	2.38308200
H	-6.82877500	3.22907500	3.21963800
C	-8.31473500	1.71740200	2.74155300
C	-8.99319200	1.77031700	3.90014900
H	-8.88255600	2.61466200	4.58434400
C	-8.43998300	0.59130700	1.76398500
H	-9.18919700	0.81917000	0.99363200
H	-7.48801900	0.38955800	1.25178400
H	-8.74195100	-0.35429700	2.24272700
C	-9.98788500	0.75529500	4.36505500
H	-9.67818500	-0.28759300	4.16058300
H	-10.99115000	0.93825000	3.92730400
O	-10.23712700	0.89271300	5.77460200

H	-9.42483800	0.70182600	6.28231500
C	-7.71444200	-4.74590500	2.76451900
H	-8.73949200	-4.52948500	2.36295600
C	-7.41244000	-3.74775400	3.91128800
H	-6.51731900	-4.06153500	4.49227400
C	-7.27221900	-2.29926200	3.38626900
H	-8.22436100	-1.94501500	2.92453300
C	-6.45269000	-3.12223200	1.23122800
H	-7.23895800	-2.67677200	0.58752200
C	-6.67951200	-4.60299100	1.61592700
H	-6.99493200	-5.18959600	0.72109700
C	-6.76460800	-1.34134900	4.44870100
H	-5.70136400	-1.51501900	4.66506100
H	-7.32826700	-1.47218700	5.38364800
H	-6.86094100	-0.29527500	4.12834500
C	-4.58744700	-1.83590600	0.30019000
H	-4.74371400	-1.18269600	1.19090100
C	-3.14340000	-2.18481800	0.07086700
H	-2.96468500	-2.87360000	-0.76026300
C	-2.15694700	-1.68336800	0.82475300
H	-2.36433000	-1.01251200	1.66342600
C	-0.74916200	-1.98621100	0.57376000
H	-0.53528400	-2.64752200	-0.27520200
C	0.24574600	-1.48685400	1.32885100
H	0.04029600	-0.84684500	2.18977400
C	1.65024400	-1.76271500	1.04134500

H	1.84453400	-2.37324500	0.14623900
C	2.65455200	-1.29801800	1.80636900
H	2.45651000	-0.70885500	2.70476000
C	4.05771000	-1.54863200	1.49309100
H	4.26175700	-2.05674300	0.53957600
C	5.06161900	-1.17473100	2.30793000
H	4.86409100	-0.67384800	3.25807100
C	6.46251100	-1.42097300	1.98202600
H	6.64990700	-1.95865500	1.04556800
C	7.47612900	-1.02299900	2.77351600
H	7.28884800	-0.49788300	3.71296000
C	8.87361600	-1.26235300	2.43095400
H	9.05816400	-1.86255400	1.53101100
C	9.90536600	-0.78381200	3.14982700
H	9.75401500	-0.19799000	4.05752700
C	-5.25806400	-1.22644100	-0.93961100
H	-6.29991300	-0.93699100	-0.68830100
H	-4.73341000	-0.28296500	-1.20861800
C	11.28874400	-1.00925900	2.73232300
H	11.42186700	-1.75736000	1.93828800
C	12.33095200	-0.33493000	3.23575900
H	12.21692600	0.40060800	4.03098000
C	13.73959800	-0.53073300	2.74040500
H	13.81276500	-1.45353400	2.10553900
C	14.16813600	0.67510500	1.86259500
H	14.38727500	1.56400400	2.49661000

C	13.14911700	1.06966600	0.76350000
H	12.17230900	1.29056300	1.27669700
C	12.87859900	-0.09172300	-0.21123900
H	12.65366600	-1.03526100	0.34242800
C	10.54709100	-0.48374400	-0.87343300
C	9.42349000	0.11249500	-1.67070000
H	9.49144200	1.22101500	-1.67729800
H	9.54975500	-0.19295100	-2.73583600
C	8.05111200	-0.35644300	-1.14798600
H	7.93198600	-0.10229000	-0.06787300
C	6.90663200	0.18991300	-2.00672400
H	7.02507000	1.28087900	-2.14908600
H	6.96947600	-0.25984400	-3.02283600
C	5.52189100	-0.11609900	-1.41163800
H	5.46926000	0.14797500	-0.32880600
C	4.41370400	0.58780600	-2.21536600
H	4.58205600	0.42631900	-3.30099600
H	4.49496300	1.68029000	-2.05691700
C	3.00775000	0.10994800	-1.83159800
H	3.04796800	-0.48215700	-0.88821300
H	2.35890900	0.97689200	-1.60412600
C	2.36961500	-0.73686400	-2.93912100
H	2.27716500	-0.16240600	-3.88827400
C	0.99848600	-1.34016800	-2.50625200
H	0.53921400	-0.79073400	-1.65373000
C	0.03716800	-1.44855600	-3.69781500

H	0.50628500	-2.06724800	-4.49342900
H	-0.13114000	-0.45193800	-4.14483100
C	-1.30064200	-2.08186900	-3.29008800
H	-1.72784400	-1.60008600	-2.37715700
C	-2.32007800	-2.08167000	-4.43949200
H	-1.95348400	-2.73406800	-5.26092500
H	-2.41870900	-1.06153100	-4.85506300
C	-3.69239200	-2.59787200	-3.98824200
C	-4.80387900	-2.47910800	-5.03502600
H	-4.61389600	-3.17409900	-5.87932600
H	-4.81760700	-1.46251700	-5.47888900
C	-6.17394900	-2.78935900	-4.42687800
H	-6.30873100	-3.87922600	-4.24648800
C	-6.42715200	-1.98422500	-3.11895400
H	-6.48722900	-0.89710100	-3.40639500
C	-5.23356200	-2.17402400	-2.13979800
H	-5.14876900	-3.24593700	-1.83592800
C	-7.72276000	-2.35399000	-2.46146200
C	13.89613800	-0.32386200	-1.30578900
H	13.64963700	-1.20949100	-1.90471400
H	14.89304700	-0.48908200	-0.86297700
H	13.96514100	0.52397700	-1.99762700
C	13.59229100	2.34707600	0.04976900
H	14.58598800	2.25009100	-0.40075600
H	13.61389300	3.20406300	0.73215700
H	12.89249200	2.59263400	-0.76195400

C	14.70782000	-0.69434200	3.91592000
H	14.69046800	0.16246500	4.59655900
H	15.73832600	-0.80586700	3.54825000
H	14.46527200	-1.58789500	4.50280200
O	-5.47181800	-5.23311400	2.05022000
H	-4.71651100	-4.58484800	2.02224000
O	-8.54012300	-3.68026700	4.79619300
H	-8.80272600	-4.59506600	5.06385600
O	-6.23608100	-2.27139100	2.37250400
O	-5.18644500	-3.14466100	0.58086600
O	-3.67595600	-3.94868200	-3.58288200
H	-2.83314600	-4.18328800	-3.08539200
O	-4.02265600	-1.79706400	-2.83084500
O	-8.11273800	-2.15735100	-1.33182600
O	-8.57306800	-2.99709600	-3.34014600
H	-9.44784300	-3.23487200	-2.92990800
O	-1.10751100	-3.47881800	-2.98414300
H	-0.31056500	-3.59920100	-2.38964200
O	1.22785200	-2.65083700	-1.96239200
H	1.95673200	-3.10127000	-2.49801500
O	3.17495300	-1.87233900	-3.29177800
H	3.96651200	-1.95712300	-2.66587400
O	5.23691300	-1.53181500	-1.46096900
H	6.07746500	-2.06738900	-1.50922300
O	7.94514600	-1.78533500	-1.28108100
H	8.67985100	-2.24525000	-0.78883500

O	10.52973700	-1.54191300	-0.27142600
O	11.63147400	0.32957400	-0.91598100
O	15.40950900	0.23708300	1.27080100
H	15.81372400	0.96695100	0.76159700
O	-7.09826600	-2.34364700	-5.42748700
H	-8.00497600	-2.68506400	-5.24370900
N	-7.72206400	-6.10969200	3.35404200
H	-6.76647800	-6.38729800	3.59641900
H	-8.05399600	-6.78982600	2.67457800

AmB-Ret4

0 1

C	3.65994700	7.44094100	1.19494400
C	4.18992000	8.49640600	2.16669600
C	4.42696200	9.84387900	1.43579100
C	3.24632300	10.21069600	0.53499400
C	3.04713500	9.14261100	-0.54605100
C	3.12941300	7.73844300	-0.00607400
H	4.60824800	10.64487700	2.17465400
H	2.32408100	10.31160100	1.14038200
H	3.41399300	11.19833900	0.06688800
H	2.07321800	9.30236200	-1.05093800
H	3.81799600	9.25990100	-1.33729100

H	5.34970600	9.77740800	0.82782700
C	2.56399900	6.70544000	-0.93391400
H	2.84941800	5.68061900	-0.66255400
H	1.46550400	6.74438700	-0.93107600
H	2.89509800	6.86404900	-1.96849900
C	3.15703100	8.68862200	3.29576700
H	3.01619200	7.77171000	3.87610500
H	3.47103500	9.47096100	3.99349800
H	2.17953400	8.97526700	2.89251600
C	5.53238800	8.03778700	2.77092800
H	5.97299400	8.81688400	3.40019400
H	5.41152900	7.14600600	3.39541400
H	6.25531400	7.78858300	1.98731300
C	3.70646900	6.05755200	1.70723100
H	2.96181900	5.84032600	2.47891400
C	4.59194100	5.13975400	1.29421800
H	5.34985100	5.38814100	0.54801400
C	4.64033000	3.75964700	1.80575800
C	3.51117100	3.07515500	2.08682700
H	2.53988000	3.55806300	1.94311900
C	6.02093600	3.20150900	1.95726000
H	6.50856000	3.07417800	0.98160800
H	6.65417100	3.87976100	2.54908100
H	6.03404300	2.22748200	2.46496700
C	3.45932900	1.70647500	2.58392900
H	4.42263000	1.21156600	2.75011900

C	2.30003100	1.07300100	2.83270800
H	1.34660800	1.58935000	2.68309400
C	2.17710900	-0.31238100	3.31411600
C	0.99985500	-0.73783500	3.80900000
H	0.13521400	-0.07529500	3.87766900
C	3.36478300	-1.21617700	3.19898300
H	3.27794800	-1.85486300	2.30458200
H	4.31160500	-0.66724900	3.11497900
H	3.46140900	-1.88321100	4.06650900
C	0.72312700	-2.13236300	4.26876700
H	1.54907400	-2.57292900	4.85748800
H	0.48077900	-2.79912100	3.40256700
O	-0.48112800	-2.19571400	5.04879300
H	-0.41372800	-1.61445100	5.83135300
C	-11.10587100	-3.64240000	0.92961200
H	-11.98224800	-2.94571600	1.00514800
C	-10.60341100	-3.95804300	2.36211000
H	-9.88033000	-4.80294400	2.36073400
C	-10.00657300	-2.69828500	3.03580800
H	-10.77062900	-1.89523300	3.12252000
C	-9.29640400	-1.82627000	0.86968100
H	-9.86807100	-0.87594300	0.91870000
C	-9.99184600	-2.96362900	0.08612800
H	-10.40352700	-2.57386500	-0.87453400
C	-9.34440500	-3.00510800	4.36616500
H	-8.42990100	-3.59852600	4.23077200

H	-10.02889300	-3.57865000	5.00931900
H	-9.05892800	-2.08759700	4.89313900
C	-7.11600200	-0.72386700	0.81123600
H	-7.11779200	-0.90183600	1.91196900
C	-5.81081100	-1.08075900	0.15617700
H	-5.80922800	-0.98930700	-0.93428400
C	-4.73470500	-1.47196500	0.85126400
H	-4.76265500	-1.57844400	1.93938300
C	-3.45708300	-1.77091900	0.20574600
H	-3.42234600	-1.64286600	-0.88351500
C	-2.36902500	-2.17812900	0.88429000
H	-2.39000700	-2.32818100	1.96715400
C	-1.09657300	-2.43292900	0.21323700
H	-1.07488100	-2.21769500	-0.86665000
C	-0.00642600	-2.88912000	0.85527100
H	-0.03174700	-3.11499500	1.93168300
C	1.27209500	-3.09098700	0.18181200
H	1.35082800	-2.69300500	-0.84140500
C	2.31930500	-3.70291600	0.76563300
H	2.24418500	-4.12169900	1.77110100
C	3.60662100	-3.85193100	0.09410700
H	3.64208800	-3.52598700	-0.95205100
C	4.69604500	-4.35184400	0.70671700
H	4.65766500	-4.68693600	1.74545500
C	5.98339900	-4.48167300	0.03287100
H	6.00029800	-4.23718500	-1.03642000

C	7.10836800	-4.87513700	0.65767600
H	7.11683200	-5.13891600	1.71625400
C	-7.56862000	0.71002700	0.49306000
H	-8.49723000	0.93674300	1.05816100
H	-6.80134200	1.42395700	0.86566800
C	8.38538600	-4.95752700	-0.05027300
H	8.32134400	-4.87173100	-1.14436400
C	9.56509000	-5.09540000	0.56960500
H	9.64315400	-5.18941300	1.65207000
C	10.87522000	-5.13713400	-0.17190500
H	10.70513300	-5.30457600	-1.26878500
C	11.62980400	-3.78995000	-0.01827000
H	12.08372300	-3.70718600	0.99528600
C	10.77365900	-2.52934500	-0.29938300
H	9.89704700	-2.55677500	0.40554500
C	10.18980200	-2.54027900	-1.72462500
H	9.70245600	-3.52023100	-1.94944500
C	7.84964400	-1.85466400	-1.99857900
C	6.93836000	-0.66753400	-1.88400200
H	7.31745000	0.04838600	-1.12532100
H	6.96445800	-0.11022100	-2.84986300
C	5.49238800	-1.10052100	-1.56824500
H	5.45840300	-1.71521000	-0.63782100
C	4.54635800	0.10146000	-1.51001300
H	4.96519800	0.88639900	-0.85082700
H	4.47589800	0.55745800	-2.52273600

C	3.13344500	-0.27774600	-1.03501900
H	3.16043200	-0.89022500	-0.10274000
C	2.26228600	0.97665800	-0.85389200
H	2.38922600	1.65222000	-1.72485000
H	2.63359200	1.54438900	0.02492000
C	0.77774300	0.64215500	-0.66495000
H	0.65159300	-0.44644000	-0.46198700
H	0.38401500	1.15028400	0.23550000
C	-0.05327400	1.03389700	-1.89157800
H	0.01641100	2.12527100	-2.09881800
C	-1.53741300	0.57250200	-1.77305400
H	-1.84876900	0.39115300	-0.71968000
C	-2.47816600	1.55211600	-2.48750400
H	-2.16940800	1.64668400	-3.55115300
H	-2.38216800	2.56226300	-2.05006800
C	-3.93796300	1.08262200	-2.42719500
H	-4.24631700	0.81733600	-1.38663500
C	-4.90405100	2.10499000	-3.04455300
H	-4.70229700	2.19483500	-4.13333800
H	-4.73347800	3.10268200	-2.59922700
C	-6.36877700	1.68992200	-2.85651200
C	-7.39736000	2.72953400	-3.31021800
H	-7.38174400	2.82693000	-4.41575300
H	-7.14145100	3.73397900	-2.91515900
C	-8.80932800	2.34929200	-2.85865100
H	-9.22774600	1.51607200	-3.46639800

C	-8.86200100	1.99978500	-1.34283000
H	-8.62599200	2.94404500	-0.77630200
C	-7.77676200	0.93702200	-1.00527800
H	-7.97986000	-0.01269400	-1.55743500
C	-10.22153500	1.53935700	-0.91146400
C	11.10500300	-2.11165500	-2.84939600
H	10.62063400	-2.22258100	-3.82783600
H	12.01186300	-2.74011200	-2.85543500
H	11.41508400	-1.06395300	-2.76006800
C	11.56809600	-1.25839100	0.00318000
H	12.49431700	-1.19720500	-0.57855100
H	11.83173600	-1.18969700	1.06429000
H	10.97241800	-0.36824200	-0.24432300
C	11.74810000	-6.28502900	0.34584900
H	11.95024800	-6.20799100	1.41871200
H	12.71655100	-6.29333500	-0.17504400
H	11.26744200	-7.25413700	0.16869700
O	-9.07147200	-3.98134800	-0.31298500
H	-8.17261500	-3.80169100	0.07237800
O	-11.71766900	-4.30517600	3.19683400
H	-12.26386200	-4.99412800	2.74515900
O	-8.91797800	-2.21917000	2.20398900
O	-8.05208900	-1.67210400	0.19816800
O	-6.70032000	0.49138700	-3.52173200
H	-5.95203700	-0.18132900	-3.48381200
O	-6.49911700	1.45411200	-1.43677900

O	-10.57038800	0.95543600	0.09008700
O	-11.19201200	1.88397700	-1.83345800
H	-12.10599300	1.60616200	-1.55517100
O	-4.10608400	-0.09922300	-3.23796900
H	-3.38055400	-0.76246000	-3.04287800
O	-1.65944500	-0.72703000	-2.37607500
H	-1.07026000	-0.75958500	-3.19653300
O	0.42664100	0.41550400	-3.09639400
H	1.16640000	-0.24469000	-2.88691600
O	2.46626700	-1.11594000	-2.00700400
H	3.12280200	-1.57738100	-2.59879700
O	4.98598300	-1.90500300	-2.64862800
H	5.56067300	-2.70538100	-2.79722000
O	7.53831900	-2.98036500	-2.34158400
O	9.11699200	-1.50401700	-1.66449200
O	12.70402900	-3.89801000	-0.97643100
H	13.30089900	-3.12791000	-0.90034600
O	-9.57007200	3.54339100	-3.08295200
H	-10.53725700	3.35851200	-3.02637100
N	-11.57288800	-4.91812800	0.32779900
H	-10.76874900	-5.51963400	0.12694200
H	-12.02951900	-4.74404400	-0.56433500

AmB-Ret5

0 1

C	-1.83502200	0.02201500	3.93183800
C	-1.16653100	-1.14587600	3.18968600
C	-0.28196800	-1.97440400	4.13673400
C	-0.93512100	-2.21499200	5.48768700
C	-1.17152400	-0.87639400	6.17244300
C	-1.82808600	0.14556900	5.27182600
H	-0.02287000	-2.91826500	3.64466300
H	-1.89140900	-2.73401400	5.35795800
H	-0.30899400	-2.85944700	6.11242700
H	-1.78864900	-1.00765400	7.06880400
H	-0.21596000	-0.46721400	6.53146500
H	0.66132200	-1.43775800	4.29862300
C	-2.47526200	1.27320600	6.03084800
H	-3.04836600	1.94683900	5.39706100
H	-3.14069400	0.87214700	6.80371600
H	-1.71569400	1.86534500	6.55548500
C	-2.23793400	-2.04960000	2.55171600
H	-2.89898000	-1.48473600	1.88962500
H	-1.76619800	-2.83093300	1.94764200
H	-2.86110600	-2.53103500	3.30961400
C	-0.26339100	-0.59338100	2.07694700
H	0.28978600	-1.40963400	1.60281400
H	-0.83564700	-0.07756200	1.30402700
H	0.46838400	0.10766700	2.49068500
C	-2.53722100	0.98005600	3.05201000

H	-3.27889000	0.54703900	2.38268000
C	-2.32729000	2.30018500	2.97212600
H	-1.52018400	2.73690100	3.55829300
C	-3.08913700	3.22200900	2.11573700
C	-4.39125700	2.98332300	1.85517500
H	-4.85645300	2.13467700	2.34942700
C	-2.31737700	4.40404700	1.59558300
H	-1.83554400	4.92597200	2.42974000
H	-1.52094300	4.07430200	0.92160100
H	-2.94416500	5.12777700	1.07385000
C	-5.25685500	3.71875200	0.95928100
H	-4.82747500	4.53811100	0.39066600
C	-6.55041100	3.39438400	0.78213100
H	-6.95293500	2.56791300	1.36691600
C	-7.46895200	3.98295900	-0.19222100
C	-8.71321900	3.48074500	-0.28239000
H	-9.00335900	2.70702400	0.42764900
C	-6.93353600	5.07351700	-1.08077500
H	-6.61596100	5.93040500	-0.47892600
H	-6.05261000	4.72625700	-1.63046200
H	-7.66775900	5.43002200	-1.80164500
C	-9.77703900	3.78829700	-1.28943100
H	-9.38914300	4.40068400	-2.11288300
H	-10.59837500	4.35116200	-0.83051400
O	-10.35646100	2.58914800	-1.79305600
H	-9.66877800	1.90984100	-1.82029400

C	11.23683500	-2.93923500	0.94586100
H	12.02512600	-2.40380700	0.40190500
C	10.94913900	-4.22354700	0.16962400
H	10.17118200	-4.78615900	0.70886600
C	10.40092000	-3.89534500	-1.21413000
H	11.16560400	-3.31010200	-1.75320600
C	9.43793700	-1.88521600	-0.41870900
H	10.14197700	-1.28083800	-1.01107700
C	9.99408000	-2.05701800	0.99193400
H	10.24869500	-1.05477300	1.36682600
C	10.04363100	-5.13000100	-2.00996300
H	9.25995300	-5.68850100	-1.49126200
H	10.92282600	-5.76807200	-2.11174000
H	9.67518400	-4.85461400	-3.00064500
C	7.54854700	-0.84538600	-1.47957200
H	7.66812400	-1.63781200	-2.22578300
C	6.10305100	-0.70892300	-1.11792600
H	5.86467900	0.00957500	-0.33804300
C	5.12612200	-1.42762300	-1.67928900
H	5.36514400	-2.15091200	-2.45840300
C	3.73836800	-1.31483700	-1.27814100
H	3.51850500	-0.59328100	-0.49510800
C	2.72700500	-2.04904800	-1.77938200
H	2.92814800	-2.78340600	-2.55886900
C	1.36908600	-1.91756400	-1.30833300
H	1.20739300	-1.15881400	-0.54596400

C	0.31796900	-2.64391500	-1.73995600
H	0.46299200	-3.40377900	-2.50719100
C	-1.01499100	-2.46585700	-1.21933400
H	-1.14257700	-1.69833200	-0.45763300
C	-2.10082400	-3.16802300	-1.60452600
H	-2.00111300	-3.93343100	-2.37327100
C	-3.40805400	-2.96552300	-1.03157600
H	-3.48104500	-2.21989500	-0.24094900
C	-4.52723900	-3.62138700	-1.39935800
H	-4.47914600	-4.35411700	-2.20402500
C	-5.80883500	-3.40456800	-0.77115800
H	-5.82115600	-2.73019900	0.08402900
C	-6.97216600	-3.95300400	-1.16697600
H	-6.99048400	-4.60724300	-2.03778200
C	8.19293900	0.44581700	-1.99971300
H	9.23377700	0.22950900	-2.25096300
H	7.69222500	0.75850600	-2.92090700
C	-8.23081600	-3.68872400	-0.49635300
H	-8.15664400	-3.12947100	0.43450900
C	-9.44018100	-4.03768000	-0.94889200
H	-9.51952300	-4.58680300	-1.88855700
C	-10.73441200	-3.71004100	-0.26002900
H	-10.51237100	-3.26258500	0.71554900
C	-11.56152100	-2.67641000	-1.04866200
H	-11.94687000	-3.18035800	-1.95082800
C	-10.77862400	-1.44370500	-1.54082600

H	-9.99214100	-1.83516300	-2.19436200
C	-10.04339000	-0.71563700	-0.41158900
H	-9.55534900	-1.43986400	0.23265800
C	-7.73284700	-0.06128200	-0.55607800
C	-6.71020000	0.66064800	-1.39095200
H	-6.98683000	0.57049200	-2.44419400
H	-6.78297300	1.71846300	-1.12508800
C	-5.27836300	0.16864400	-1.17400400
H	-5.18219200	-0.84353600	-1.58839600
C	-4.28746900	1.09510400	-1.86469100
H	-4.53842600	1.14849500	-2.92959100
H	-4.39686600	2.10028500	-1.43995200
C	-2.83509700	0.65625000	-1.72004100
H	-2.73644000	-0.38834600	-2.04533500
C	-1.89103200	1.51761700	-2.54904000
H	-2.10004000	2.57394900	-2.33911600
H	-2.13051500	1.35282100	-3.60398600
C	-0.40732300	1.20938500	-2.28806000
H	-0.29911000	0.20842700	-1.86306900
H	0.14350900	1.19734100	-3.23359600
C	0.29575000	2.21854300	-1.38264700
H	0.33092000	3.18563400	-1.90313800
C	1.74398100	1.77661500	-1.06351000
H	2.11480500	1.12278900	-1.86113200
C	2.70563700	2.94674700	-0.88976100
H	2.35293000	3.58723900	-0.07228900

H	2.70111900	3.54709200	-1.80686200
C	4.13374900	2.48781900	-0.60855500
H	4.44882800	1.79922700	-1.40442900
C	5.11422300	3.65235900	-0.57212400
H	4.79958100	4.37730000	0.18491200
H	5.10292800	4.14784300	-1.54722700
C	6.54790200	3.22991700	-0.25003000
C	7.54637000	4.36227300	-0.44276400
H	7.35332300	5.16349000	0.27363500
H	7.43878200	4.76753000	-1.45282000
C	8.97839900	3.88326700	-0.27851800
H	9.13249800	3.58406500	0.76852000
C	9.23286300	2.64084600	-1.17572400
H	9.25450000	2.99940500	-2.21162400
C	8.12497200	1.59221600	-0.99754900
H	8.19622800	1.19103800	0.01755200
C	10.58034000	2.05295100	-0.84523000
C	-10.85852000	0.24137000	0.43215900
H	-10.25114700	0.61175800	1.26230800
H	-11.71668800	-0.29910200	0.83511100
H	-11.20153500	1.09257800	-0.15722500
C	-11.67317600	-0.52135300	-2.36852100
H	-12.51739200	-0.14563000	-1.78316800
H	-12.07141300	-1.05338900	-3.23765800
H	-11.13257400	0.35562900	-2.72775700
C	-11.56837000	-4.97253000	-0.02056700

H	-11.78362600	-5.47872500	-0.96810100
H	-12.51672300	-4.71554200	0.45351700
H	-11.02986900	-5.67568000	0.61881800
O	9.04827500	-2.68793000	1.83080100
H	8.18001100	-2.36373000	1.55254500
O	12.13413200	-4.97419100	0.03553400
H	12.56301200	-4.89478700	0.90304900
O	9.21577100	-3.12036600	-1.06328100
O	8.20842700	-1.25236100	-0.26893200
O	6.67524400	2.77798200	1.06674500
H	5.89110500	2.22822800	1.24713200
O	6.85493400	2.19947400	-1.18427200
O	10.80016500	0.97124300	-0.35417600
O	11.59632000	2.90778700	-1.12423200
H	12.41357300	2.46511900	-0.84235000
O	4.21474900	1.80648600	0.64597800
H	3.37378000	1.32957500	0.77183300
O	1.71291500	0.98264100	0.12384700
H	0.95439300	1.34999800	0.62115800
O	-0.35764600	2.41690600	-0.13662900
H	-1.17455200	1.86934300	-0.09976800
O	-2.41336200	0.74323100	-0.35594700
H	-3.18402400	0.56525100	0.21237100
O	-4.93662400	0.13881400	0.21143600
H	-5.67921400	-0.28101000	0.68159400
O	-7.47813200	-0.63814600	0.48826500

O	-8.95784900	0.03004600	-1.06806000
O	-12.64736500	-2.31920000	-0.19958200
H	-13.32200900	-1.88063100	-0.72914000
O	9.80220400	4.97366500	-0.62943700
H	10.71879900	4.67031000	-0.63993900
N	11.76958100	-3.32022500	2.25106900
H	10.98767600	-3.59936900	2.83975100
H	12.20837000	-2.52511700	2.70454300

AmB-Ret6

0 1

C	-4.67506400	3.75755700	-1.21679300
C	-5.14080700	4.58564800	-0.00909700
C	-6.65939100	4.80903400	-0.08054700
C	-7.42612400	3.52359000	-0.35075000
C	-6.99991800	2.94256700	-1.69396300
C	-5.51202100	3.00527000	-1.95580900
H	-6.99495200	5.28090200	0.84940100
H	-7.23384400	2.80099300	0.44700000
H	-8.50493700	3.71094300	-0.34761900
H	-7.33358100	1.90160400	-1.78210800
H	-7.50450400	3.47712900	-2.51238600
H	-6.86945600	5.52475800	-0.88583700
C	-5.08164100	2.17898700	-3.14047900

H	-4.00498600	2.02099600	-3.17939100
H	-5.57483300	1.20244900	-3.12116700
H	-5.38441700	2.65860900	-4.07925200
C	-4.75782200	3.87161000	1.30053100
H	-3.68525000	3.65811100	1.33572800
H	-4.99079300	4.50991700	2.15907900
H	-5.29012500	2.92798100	1.43736100
C	-4.46653900	5.96755500	-0.02468300
H	-4.88403000	6.59188700	0.77152200
H	-3.38792700	5.90067300	0.13517400
H	-4.63052700	6.47608100	-0.97927200
C	-3.23404800	3.87716600	-1.52397200
H	-2.53701900	3.73836800	-0.69834100
C	-2.74349600	4.26481700	-2.70708900
H	-3.45353700	4.52417000	-3.49196500
C	-1.32544300	4.46077600	-3.03584000
C	-0.37881000	3.59623100	-2.61740800
H	-0.70636700	2.70594900	-2.08473800
C	-1.05377200	5.68177600	-3.87613900
H	-1.71362100	5.68848400	-4.75049800
H	-1.27344500	6.59475100	-3.31209400
H	-0.02774900	5.74253600	-4.23759700
C	1.04790800	3.71923200	-2.84490300
H	1.39982200	4.64290000	-3.29242500
C	1.94382900	2.76260700	-2.54708800
H	1.59073500	1.83357200	-2.10321400

C	3.38484100	2.85054800	-2.79301800
C	4.14825200	1.77201000	-2.55421300
H	3.66344600	0.87896700	-2.16652800
C	3.91933600	4.15024700	-3.33523400
H	3.51958700	4.34422900	-4.33576800
H	3.61229100	4.98656200	-2.70002900
H	5.00615300	4.16182500	-3.40754600
C	5.62030400	1.60985800	-2.78477200
H	6.12646200	2.57848500	-2.88408100
H	5.79356100	1.06854700	-3.72293200
O	6.23959800	0.82016200	-1.78047500
H	6.07144300	1.22753500	-0.91809700
C	-9.35024100	-0.15696600	3.61354300
H	-10.14173700	-0.03275000	2.86383000
C	-8.95167900	1.23749600	4.08699900
H	-8.16562200	1.13070100	4.85043000
C	-8.36833000	2.04904800	2.93616400
H	-9.14941600	2.16090000	2.16530300
C	-7.56289400	0.07022200	1.88667000
H	-8.27374300	0.16455800	1.05017300
C	-8.16116000	-0.84290500	2.95533800
H	-8.48126300	-1.76457100	2.45010300
C	-7.89098000	3.41021100	3.38950300
H	-7.06982000	3.29141500	4.10143300
H	-8.71101200	3.93728400	3.87967600
H	-7.53244000	3.99946700	2.54567400

C	-5.79336000	-0.10586200	0.25324900
H	-5.90980900	0.97736500	0.15131300
C	-4.34093200	-0.43947100	0.37463500
H	-4.10781900	-1.46897600	0.63445200
C	-3.35603200	0.45022900	0.21853200
H	-3.59184800	1.47747000	-0.05022800
C	-1.96736900	0.10332300	0.43691500
H	-1.76577800	-0.93077000	0.70441300
C	-0.93014800	0.96060400	0.38317100
H	-1.10207700	2.00578100	0.13076300
C	0.42048200	0.55059300	0.68317100
H	0.54841700	-0.49937700	0.94035100
C	1.50187800	1.35634900	0.67044800
H	1.39353400	2.40664100	0.40439100
C	2.82299400	0.88396300	0.99909000
H	2.92026600	-0.17230600	1.25176300
C	3.93274600	1.65064800	1.00606400
H	3.85847600	2.70177600	0.73181700
C	5.23079800	1.14438200	1.37237200
H	5.28592100	0.09163500	1.65115200
C	6.37080900	1.86983700	1.39408700
H	6.34241900	2.92120100	1.10772600
C	7.64084700	1.32301700	1.80965600
H	7.62981700	0.29830800	2.17665800
C	8.81860700	1.97076600	1.76561800
H	8.86317000	2.98599200	1.37319100

C	-6.51256500	-0.80002500	-0.91043900
H	-7.54352100	-0.44047100	-0.93653000
H	-6.03699600	-0.51383400	-1.85247200
C	10.06123600	1.35428700	2.18890900
H	9.96064200	0.39169700	2.68594500
C	11.28326400	1.84916000	1.96686400
H	11.38595600	2.80435900	1.44941000
C	12.56437700	1.15923600	2.34130300
H	12.33386000	0.31839500	3.00543200
C	13.26917400	0.58014700	1.09912400
H	13.60009600	1.43342000	0.48360700
C	12.37993200	-0.29290700	0.19405100
H	11.54147100	0.34376900	-0.10473200
C	11.74503800	-1.48158000	0.92561700
H	11.31594600	-1.14871300	1.86699200
C	9.42475800	-2.02356500	0.61815000
C	8.38807200	-2.46584600	-0.38185600
H	8.72603400	-2.21807700	-1.38957900
H	8.30634200	-3.55680800	-0.31194700
C	7.02021100	-1.84350500	-0.09670800
H	7.11740000	-0.75223100	-0.13071100
C	5.99387000	-2.27437600	-1.13126000
H	6.31390100	-1.88671800	-2.10188300
H	5.96568900	-3.36965900	-1.18609100
C	4.58377400	-1.76776200	-0.84926400
H	4.62238700	-0.68895700	-0.67301400

C	3.65182200	-2.03721100	-2.02442200
H	3.77568600	-3.07794800	-2.34816800
H	3.98959500	-1.40967900	-2.85438300
C	2.17211500	-1.76045300	-1.71274200
H	2.08424000	-1.08280900	-0.85960200
H	1.69774300	-1.25034900	-2.55747700
C	1.35780700	-3.02420700	-1.44123200
H	1.30588000	-3.60528000	-2.37240400
C	-0.08787300	-2.69396400	-0.98815400
H	-0.36615100	-1.68968700	-1.32833900
C	-1.11080900	-3.70107100	-1.50230600
H	-0.82420700	-4.70632600	-1.17011600
H	-1.08712200	-3.69580600	-2.59828500
C	-2.53147300	-3.39692400	-1.03503400
H	-2.78805400	-2.36351400	-1.30598900
C	-3.55023000	-4.32782400	-1.68036600
H	-3.27223800	-5.36919500	-1.49014900
H	-3.53494400	-4.16217800	-2.76146800
C	-4.97653100	-4.12659300	-1.16827800
C	-6.00376600	-4.89458400	-1.98794100
H	-5.84848600	-5.96983400	-1.87805600
H	-5.88706700	-4.63445100	-3.04374900
C	-7.42600400	-4.55407900	-1.57548800
H	-7.59454400	-4.92070300	-0.55232100
C	-7.62682200	-3.01358600	-1.57170900
H	-7.62640800	-2.69271600	-2.62023900

C	-6.49516500	-2.31806900	-0.80097000
H	-6.57382100	-2.60492600	0.25125900
C	-8.96791600	-2.67607200	-0.97273700
C	12.63363600	-2.68448300	1.16978800
H	12.09049100	-3.44038300	1.74163600
H	13.51385300	-2.37016700	1.73201500
H	12.95594900	-3.13312400	0.22763700
C	13.11989000	-0.71383900	-1.07489900
H	14.01497400	-1.30294000	-0.85270500
H	13.42779000	0.16352900	-1.65104800
H	12.47653200	-1.32562300	-1.71096300
C	13.50586200	2.10870400	3.08762500
H	13.74931600	2.97779900	2.46626200
H	14.43663900	1.60094200	3.34299200
H	13.03671200	2.47435300	4.00392600
O	-7.21247500	-1.11903900	3.96585000
H	-6.35920500	-1.21811300	3.52094800
O	-10.08108300	1.90954400	4.59619300
H	-10.55227200	1.22746800	5.10175900
O	-7.24774300	1.35464800	2.38827400
O	-6.37688100	-0.53435200	1.49453900
O	-5.11321600	-4.53219600	0.16257300
H	-4.32168700	-4.20748900	0.62964800
O	-5.23925600	-2.73430600	-1.31535200
O	-9.17519600	-2.05161600	0.04115600
O	-9.99708400	-3.18963500	-1.69136700

H	-10.81000200	-2.95513900	-1.21441000
O	-2.63865400	-3.53932100	0.38370900
H	-1.79009700	-3.24744400	0.76536500
O	-0.10182200	-2.65729500	0.44036800
H	0.63233100	-3.26456700	0.67435600
O	1.92496400	-3.86064100	-0.44317600
H	2.74231200	-3.44272500	-0.09233400
O	4.03012600	-2.39913600	0.31257400
H	4.74851000	-2.47778200	0.96546700
O	6.54651600	-2.24130500	1.19076300
H	7.27736500	-2.10024500	1.81725400
O	9.16675800	-1.80290500	1.79129400
O	10.63346600	-1.91692600	0.08353500
O	14.40649700	-0.12992100	1.57991900
H	15.01138900	-0.27741200	0.84503500
O	-8.26993700	-5.20317500	-2.50117300
H	-9.17882200	-4.92708800	-2.32863100
N	-9.91869900	-0.86845200	4.75531000
H	-9.14993900	-1.19290200	5.33805200
H	-10.42221800	-1.69437300	4.44772500

AmB-Ret7

0 1

C	-2.48965800	4.88238700	1.98145300

C	-2.24568900	5.84580700	3.15192900
C	-1.61940200	7.15759200	2.65284600
C	-2.32355500	7.70824200	1.42250400
C	-2.22769300	6.70363300	0.28171300
C	-2.51094800	5.27673500	0.69397300
H	-1.62221300	7.88898900	3.46908900
H	-3.37677800	7.90415900	1.65067400
H	-1.88652400	8.66608700	1.12314200
H	-2.90916500	6.98529900	-0.52956600
H	-1.22093500	6.73496400	-0.16025600
H	-0.56773700	6.97077800	2.40133500
C	-2.79046900	4.36467400	-0.47376200
H	-3.24766200	3.42056400	-0.17851600
H	-3.45465100	4.86585200	-1.18732600
H	-1.86448300	4.12206700	-1.00590700
C	-3.57762400	6.13444600	3.86840200
H	-4.04033600	5.21473500	4.23662000
H	-3.41186200	6.78633600	4.73249900
H	-4.29505400	6.62174100	3.20411200
C	-1.27495300	5.21353100	4.16149500
H	-1.04129000	5.92907900	4.95650100
H	-1.69504200	4.31870300	4.62675500
H	-0.33863800	4.92573800	3.67491500
C	-2.73705200	3.48155400	2.37241600
H	-3.49024000	3.31071800	3.14110300
C	-2.07303800	2.42471200	1.88808700

H	-1.24107200	2.61258900	1.21230000
C	-2.37485300	1.01460500	2.16919300
C	-3.65209800	0.63171600	2.37539400
H	-4.41261700	1.40518300	2.29805400
C	-1.19673200	0.08097300	2.11363200
H	-0.66242900	0.21901600	1.16784000
H	-0.48641700	0.30113800	2.91736100
H	-1.47407100	-0.97100300	2.18547200
C	-4.16790600	-0.70211600	2.60093200
H	-3.46387900	-1.52566400	2.67467300
C	-5.48929000	-0.94677400	2.68217600
H	-6.17385300	-0.10256200	2.60247400
C	-6.12450300	-2.25217200	2.83632200
C	-7.46333100	-2.32757000	2.82142200
H	-8.03791700	-1.41173800	2.69117800
C	-5.24120200	-3.46556100	2.94194600
H	-4.65703600	-3.59055500	2.02419200
H	-4.53232800	-3.36176600	3.76915200
H	-5.81078800	-4.38168200	3.10069400
C	-8.29048300	-3.58230200	2.89369200
H	-8.08933100	-4.13507500	3.82430100
H	-8.03841600	-4.25100600	2.06536700
O	-9.67751200	-3.31498600	2.75456000
H	-9.95964900	-2.79407100	3.51539100
C	10.97721100	-3.13296400	2.16567100
H	11.82062700	-2.80691700	1.54412900

C	10.64758100	-4.56796400	1.75709900
H	9.81356000	-4.92184300	2.38296800
C	10.18308900	-4.61301100	0.30626700
H	11.00502700	-4.23602500	-0.32624000
C	9.30791200	-2.41962400	0.45748100
H	10.07238300	-2.03584600	-0.23539700
C	9.78918200	-2.21682300	1.89152100
H	10.08709500	-1.16241500	1.99019400
C	9.78787500	-6.00272900	-0.13862300
H	8.94894700	-6.35791700	0.46568200
H	10.63017200	-6.68373000	-0.00721900
H	9.48202100	-5.99746100	-1.18717400
C	7.51879600	-1.64898300	-0.95275900
H	7.60371300	-2.63276700	-1.42578300
C	6.07749100	-1.32393000	-0.71680400
H	5.87203700	-0.40063000	-0.18163800
C	5.06472300	-2.10276300	-1.10823900
H	5.26961900	-3.02908900	-1.64421500
C	3.67768200	-1.78726000	-0.83153800
H	3.49166000	-0.86558400	-0.28540400
C	2.62834000	-2.55549100	-1.17974700
H	2.79862400	-3.48365000	-1.72472800
C	1.26651500	-2.20841900	-0.85113400
H	1.13213000	-1.27274000	-0.31227600
C	0.17994300	-2.94049300	-1.16813900
H	0.29913800	-3.87771300	-1.71095500

C	-1.15958900	-2.54037200	-0.81589100
H	-1.26853100	-1.59100800	-0.29379900
C	-2.27442600	-3.24434500	-1.09762900
H	-2.18967900	-4.18769900	-1.63614400
C	-3.59590700	-2.82073200	-0.70863100
H	-3.67058600	-1.89257500	-0.14145100
C	-4.73026000	-3.49061700	-0.99977300
H	-4.67307800	-4.40957700	-1.58234500
C	-6.03390400	-3.05251200	-0.56770700
H	-6.06283200	-2.17015600	0.06756200
C	-7.20165900	-3.64527700	-0.87996900
H	-7.21238000	-4.52417200	-1.52374400
C	8.26587800	-0.61030900	-1.79920600
H	9.30006100	-0.94308300	-1.91295300
H	7.82369700	-0.56838600	-2.79909400
C	-8.46744200	-3.13653700	-0.39183600
H	-8.38915800	-2.31949200	0.31962900
C	-9.68361100	-3.56383200	-0.74534100
H	-9.78144700	-4.37862200	-1.46487200
C	-10.96098300	-2.96464400	-0.22797900
H	-10.71661200	-2.31374000	0.61654900
C	-11.65176000	-2.10902500	-1.30535500
H	-12.00431700	-2.79171700	-2.09661400
C	-10.73755300	-1.07521400	-1.99149600
H	-9.91344100	-1.64894300	-2.42593500
C	-10.07854400	-0.10452900	-1.00484900

H	-9.69225800	-0.65438800	-0.15130500
C	-7.74416500	0.44722100	-1.10071900
C	-6.65507300	1.03846900	-1.96401900
H	-6.93327200	0.93801600	-3.01504100
H	-6.59840600	2.10845700	-1.73283700
C	-5.29299900	0.40250600	-1.68242900
H	-5.35200300	-0.67639800	-1.88645900
C	-4.18745600	1.01122700	-2.53405600
H	-4.42535600	0.85507600	-3.59176800
H	-4.15239700	2.09253000	-2.35476500
C	-2.80517900	0.42540900	-2.24494300
H	-2.84812800	-0.66868600	-2.33775200
C	-1.75204600	0.95911400	-3.21168900
H	-1.85916700	2.04838300	-3.28594200
H	-1.97630100	0.55433200	-4.20313700
C	-0.31178900	0.60273000	-2.80422000
H	-0.30819600	-0.26947300	-2.14611800
H	0.26857400	0.31886000	-3.68767800
C	0.44326300	1.74928900	-2.13286900
H	0.58650500	2.54689800	-2.87426600
C	1.83627400	1.30275500	-1.61774100
H	2.15795900	0.39931100	-2.14749400
C	2.89584000	2.38737200	-1.77748700
H	2.58153700	3.28006300	-1.22270300
H	2.95933000	2.66312800	-2.83647000
C	4.27693100	1.94716700	-1.30190000

H	4.56465100	1.03002700	-1.83364400
C	5.33150900	3.01196300	-1.57517000
H	5.03751700	3.95238300	-1.09872600
H	5.38719500	3.17777200	-2.65490100
C	6.72314300	2.64314400	-1.06188500
C	7.79335600	3.61783300	-1.53166300
H	7.61972900	4.60690600	-1.10269800
H	7.75023100	3.70046700	-2.62134400
C	9.18642200	3.14857000	-1.15019600
H	9.27980600	3.17491100	-0.05467600
C	9.40357900	1.68234300	-1.61575800
H	9.48901300	1.70820900	-2.70851000
C	8.22859200	0.78765200	-1.19369500
H	8.23521400	0.71058600	-0.10258300
C	10.69874200	1.16323900	-1.04690500
C	-10.92649200	1.05239300	-0.51765700
H	-10.37154400	1.63772100	0.21936100
H	-11.83292500	0.65777600	-0.05666600
H	-11.20559900	1.71280400	-1.34157900
C	-11.46077000	-0.35684600	-3.12909400
H	-12.34558500	0.18075700	-2.77497300
H	-11.78301400	-1.07067500	-3.89273200
H	-10.80321900	0.37346700	-3.60622500
C	-11.90435800	-4.04886000	0.29611900
H	-12.11486800	-4.79542800	-0.47839200
H	-12.85108800	-3.60704100	0.61115500

H	-11.44486000	-4.55007100	1.15005400
O	8.77006300	-2.54506100	2.81320400
H	7.93680900	-2.27185400	2.40379500
O	11.78976700	-5.37973200	1.90605600
H	12.18285200	-5.08239700	2.74261100
O	9.04125500	-3.77383600	0.16661100
O	8.11267200	-1.71538200	0.35461900
O	6.77201100	2.60706600	0.33473600
H	5.94636800	2.18179800	0.62996700
O	7.00514000	1.36592500	-1.62492800
O	10.83319300	0.27169100	-0.24281800
O	11.77446200	1.84866000	-1.50958200
H	12.55147400	1.47606700	-1.06153900
O	4.27351000	1.67911200	0.10291300
H	3.38939700	1.34015800	0.33395200
O	1.70922400	0.93672800	-0.24114700
H	0.97775600	1.50822100	0.06921900
O	-0.25188600	2.31354800	-1.02842900
H	-1.09532900	1.82094600	-0.89481200
O	-2.37552100	0.74360900	-0.91993300
H	-3.14943700	0.70239100	-0.32350000
O	-4.92569900	0.60964500	-0.32568900
H	-5.63679700	0.26065200	0.23727800
O	-7.55275600	0.02885300	0.02975700
O	-8.92291100	0.45147500	-1.70817900
O	-12.77179800	-1.49195400	-0.67507400

H	-13.38343300	-1.19524100	-1.35729000
O	10.08681500	4.04370900	-1.76583800
H	10.98371800	3.71251700	-1.63117200
N	11.42549700	-3.15998000	3.55517700
H	10.60199700	-3.22915400	4.14926400
H	11.89025700	-2.29101500	3.79864300

AmB-Ret8

0 1

C	1.20443000	4.76520900	1.96368400
C	2.46978100	4.17078200	1.33312800
C	3.68502600	5.08332000	1.57070600
C	3.74443300	5.62055500	2.99181300
C	2.50769600	6.46812800	3.25714900
C	1.22320800	5.80423200	2.81498900
H	4.59719300	4.53475100	1.31155400
H	3.78222000	4.79123000	3.70667700
H	4.65321100	6.21051400	3.14648400
H	2.43079900	6.71281100	4.32340300
H	2.59901400	7.43492500	2.74084200
H	3.62633500	5.93501300	0.88120600
C	-0.00284800	6.41064600	3.44382000
H	-0.93000000	5.94872700	3.11022400
H	0.05299900	6.32944300	4.53585700

H	-0.05366800	7.48234600	3.21686100
C	2.73810000	2.77573300	1.92776900
H	1.91672300	2.08476400	1.72057800
H	3.64497300	2.34612000	1.49275400
H	2.86880200	2.82155700	3.01173600
C	2.27470100	4.04182500	-0.18537500
H	3.18347000	3.64417400	-0.64383000
H	1.44944300	3.37231100	-0.43205100
H	2.05256900	5.01759000	-0.62859900
C	-0.06334900	4.08328200	1.59930400
H	-0.20835300	3.07133000	1.97915000
C	-1.01058200	4.60680800	0.81700200
H	-0.81956100	5.57817100	0.36050000
C	-2.31578800	4.00081000	0.50892300
C	-3.01497700	3.37447300	1.47644500
H	-2.57410700	3.34018300	2.47041700
C	-2.79397500	4.20062600	-0.90333900
H	-2.74596300	5.26116000	-1.17361300
H	-2.13464700	3.65722200	-1.58598900
H	-3.81597100	3.85717700	-1.06523800
C	-4.35572300	2.83402100	1.36710000
H	-4.84059600	2.83937800	0.39554200
C	-5.05209900	2.35287800	2.40881700
H	-4.55884800	2.25564500	3.37628500
C	-6.46414000	1.93913400	2.34807200
C	-7.34417100	2.66574700	1.64515800

H	-6.97617600	3.57676700	1.17459000
C	-6.80792900	0.70331700	3.13990900
H	-7.82988200	0.36197000	2.97888400
H	-6.13106800	-0.11681500	2.87829200
H	-6.67289900	0.88988300	4.21085400
C	-8.80379800	2.41223000	1.43184200
H	-9.40498300	3.14334800	1.99209000
H	-9.09827200	1.41674800	1.75837300
O	-9.14440300	2.47066100	0.04263900
H	-9.03060600	3.38044200	-0.25573600
C	10.52885000	-2.46670600	2.68624400
H	11.31585200	-2.51902900	1.92342200
C	10.13497000	-3.90404100	3.02304800
H	9.35659000	-3.87566900	3.80135200
C	9.53656300	-4.58982600	1.80074900
H	10.30336300	-4.60075800	1.00721800
C	8.72191000	-2.52852800	0.97095500
H	9.42633400	-2.55729900	0.12576600
C	9.33809400	-1.71271800	2.10417600
H	9.66410000	-0.75306300	1.67589500
C	9.07436500	-6.00010900	2.08865400
H	8.28957400	-5.98208200	2.84950900
H	9.91193800	-6.59258100	2.46012100
H	8.67090900	-6.46345300	1.18558300
C	6.82697500	-2.39025600	-0.50365500
H	6.84362000	-3.48448700	-0.46708500

C	5.42183600	-1.90091300	-0.34813100
H	5.29376300	-0.82767600	-0.23533800
C	4.35241100	-2.70187000	-0.33476800
H	4.48102500	-3.77750500	-0.45411400
C	3.00108200	-2.21245300	-0.14691300
H	2.88814500	-1.14389300	0.01579800
C	1.90065900	-2.98745900	-0.14163500
H	2.00143200	-4.06063400	-0.30320900
C	0.57015800	-2.47100000	0.07823100
H	0.49270800	-1.39879300	0.24830200
C	-0.54727900	-3.22446900	0.07986500
H	-0.46507900	-4.29760700	-0.09157800
C	-1.87096100	-2.69870600	0.30544000
H	-1.96321800	-1.62372900	0.46066300
C	-2.98617500	-3.45673300	0.32720500
H	-2.89666200	-4.52979800	0.15948300
C	-4.30817000	-2.94492100	0.58464300
H	-4.40324100	-1.87472000	0.76598900
C	-5.41944900	-3.70821500	0.61250300
H	-5.33626600	-4.77547200	0.40896900
C	-6.73302000	-3.18988200	0.90411200
H	-6.79419300	-2.13638700	1.16693400
C	-7.87420700	-3.90078100	0.84850800
H	-7.84309400	-4.94953700	0.55488500
C	7.52073700	-1.91602600	-1.78790600
H	8.52676600	-2.34131900	-1.80994400

H	6.98180800	-2.30498400	-2.65702100
C	-9.17259400	-3.31041300	1.10959600
H	-9.15448900	-2.29691900	1.50206000
C	-10.35137900	-3.87872200	0.83650500
H	-10.37622500	-4.88609900	0.41759700
C	-11.68041600	-3.19903500	1.00658400
H	-11.53559300	-2.27437900	1.57703500
C	-12.27710500	-2.80401900	-0.35846600
H	-12.52241500	-3.74033700	-0.88799600
C	-11.32911700	-2.01599700	-1.28211100
H	-10.43833600	-2.64095800	-1.39694100
C	-10.82714700	-0.70053100	-0.67221000
H	-10.48631500	-0.87578200	0.34549200
C	-8.49527700	-0.15322300	-0.83724900
C	-7.40453000	0.30973300	-1.76501900
H	-7.66053900	0.05226000	-2.79449300
H	-7.38182900	1.40109300	-1.67544600
C	-6.03988400	-0.25667600	-1.37089000
H	-6.09594300	-1.35541400	-1.34897100
C	-4.95171300	0.16700300	-2.34497500
H	-5.18239100	-0.24951300	-3.33123100
H	-4.94995500	1.25987900	-2.43663100
C	-3.55305700	-0.28104700	-1.92580400
H	-3.57568600	-1.34927700	-1.66678900
C	-2.54078600	-0.07259600	-3.04491600
H	-2.61298500	0.96412500	-3.39642900

H	-2.84326800	-0.70924300	-3.88176600
C	-1.09401500	-0.39276900	-2.63738500
H	-1.08062000	-1.06058500	-1.77135900
H	-0.59062000	-0.93649500	-3.44327800
C	-0.24223200	0.83716900	-2.34279000
H	-0.15792700	1.42749000	-3.26508100
C	1.19401000	0.44228500	-1.88169300
H	1.35062300	-0.63114300	-2.04049800
C	2.27385800	1.21617500	-2.63023000
H	2.10083700	2.29088600	-2.50673500
H	2.18162000	0.98945500	-3.69917000
C	3.69150800	0.87490400	-2.17991500
H	3.86249900	-0.20380400	-2.29253400
C	4.73218100	1.62025200	-3.00694700
H	4.52092400	2.69414300	-2.98731000
H	4.66111700	1.27820200	-4.04357400
C	6.16606600	1.42073600	-2.51664200
C	7.19796300	1.97917600	-3.48560700
H	7.10183700	3.06454200	-3.55794700
H	7.02901000	1.54946000	-4.47695100
C	8.61297200	1.63269600	-3.05569800
H	8.83362400	2.15747100	-2.11483800
C	8.73428000	0.10612500	-2.79452200
H	8.69666900	-0.38450900	-3.77428700
C	7.59206500	-0.39852400	-1.90024000
H	7.72507900	0.03185800	-0.90356200

C	10.07043600	-0.18779600	-2.16306400
C	-11.78062400	0.47710500	-0.69833700
H	-11.29136700	1.34880400	-0.26012800
H	-12.68034600	0.22233100	-0.13667300
H	-12.06388200	0.72770000	-1.72332100
C	-11.94141400	-1.82009800	-2.66867100
H	-12.88591700	-1.26861000	-2.62773000
H	-12.13538200	-2.78539000	-3.14508500
H	-11.26386700	-1.25493700	-3.31239400
C	-12.66458700	-4.08453400	1.77610200
H	-12.81997100	-5.03378800	1.25127000
H	-13.63051200	-3.58774000	1.87434200
H	-12.27835700	-4.31353700	2.77206500
O	8.40315900	-1.51302800	3.14410000
H	7.53963800	-1.41893300	2.71735300
O	11.27052000	-4.62085500	3.45030300
H	11.74618200	-3.99240500	4.01741800
O	8.40315200	-3.84405900	1.36789100
O	7.53631000	-1.88153100	0.63839500
O	6.38253200	2.03425000	-1.27832900
H	5.60292900	1.82627300	-0.73253000
O	6.34303300	0.01105600	-2.43706900
O	10.26973200	-0.60658700	-1.04749600
O	11.10565400	0.11327100	-2.98674500
H	11.91759800	-0.06675200	-2.48508000
O	3.88609900	1.22115000	-0.80499800

H	3.02799800	1.12340500	-0.35094900
O	1.29701900	0.66567000	-0.47610700
H	0.53722700	1.25663000	-0.28239200
O	-0.79722500	1.68728900	-1.34894400
H	-1.64669500	1.31606400	-1.01971700
O	-3.10026200	0.45330500	-0.78626800
H	-3.84115800	0.52947100	-0.15811000
O	-5.65921800	0.21350600	-0.08337500
H	-6.44220900	0.15444500	0.48999600
O	-8.30380000	-0.34951800	0.35338400
O	-9.65391300	-0.31774200	-1.45343700
O	-13.47484600	-2.08714300	-0.07745200
H	-13.99082100	-2.01978000	-0.88807000
O	9.46035500	2.06474400	-4.09806000
H	10.35709500	1.76333400	-3.90483400
N	11.10619500	-1.87195400	3.88858200
H	10.34188300	-1.60715400	4.50631600
H	11.60754400	-1.01923800	3.66077700

AmB-Ret9

0 1

C	12.12633200	5.44967400	-0.55266600
C	13.20188400	6.09506000	0.33561600
C	13.52150900	7.51944300	-0.14601000

C	12.26975600	8.33246600	-0.43432300
C	11.46588800	7.65887400	-1.53836900
C	11.33154900	6.16138400	-1.37443100
H	14.15089300	8.01595900	0.60135300
H	11.65744700	8.41108900	0.47018500
H	12.53041700	9.35534900	-0.72390900
H	10.46570800	8.10253900	-1.60509500
H	11.93623600	7.85460400	-2.51343500
H	14.11744400	7.45395500	-1.06536000
C	10.23200700	5.57318500	-2.21978400
H	9.99690600	4.54252600	-1.96041600
H	9.32066500	6.17212700	-2.11684200
H	10.50111200	5.59996600	-3.28291000
C	12.71284900	6.12973700	1.79523200
H	12.49030400	5.12632800	2.16770400
H	13.48432800	6.55694000	2.44444800
H	11.80516000	6.72787200	1.90113600
C	14.50116800	5.27659400	0.27332200
H	15.28946600	5.78169800	0.84094800
H	14.37552600	4.27520500	0.69151000
H	14.84285200	5.16450500	-0.75971600
C	12.01303200	3.98632000	-0.40259300
H	11.98745200	3.60598400	0.61755400
C	12.01602400	3.09446000	-1.40151700
H	12.15872400	3.45898800	-2.41789500
C	11.87856500	1.63941000	-1.25304100

C	11.12399900	1.11457400	-0.26673500
H	10.59431000	1.80682600	0.38385700
C	12.60647500	0.82695100	-2.29035500
H	12.30547300	1.14363300	-3.29513900
H	13.68732500	0.98989900	-2.22025900
H	12.41935500	-0.24345500	-2.20988500
C	10.89916900	-0.28826000	0.01835600
H	11.44033600	-1.03186100	-0.56069700
C	10.04828000	-0.69865600	0.97228400
H	9.53464300	0.05940100	1.55983100
C	9.70747000	-2.09211300	1.28734300
C	9.68733200	-3.04964800	0.34674900
H	9.91343200	-2.77463200	-0.67992600
C	9.33907000	-2.34052200	2.72727800
H	10.18750800	-2.11040000	3.38006400
H	8.52695900	-1.66645700	3.02235200
H	9.00446500	-3.35994700	2.90894400
C	9.30106900	-4.48299100	0.59303300
H	9.94806500	-4.95316400	1.33992700
H	9.39704700	-5.06174400	-0.33389200
O	7.97348700	-4.59136600	1.11168700
H	7.37399700	-4.03497300	0.57640100
C	6.85136100	0.00081900	-0.52970500
H	7.49576400	-0.74943900	-0.05687800
C	6.69562400	1.16086300	0.45310000
H	6.02705700	1.90997800	0.00314400

C	6.03040700	0.66842000	1.73143000
H	6.65301300	-0.13810200	2.15808400
C	4.77893400	-0.94017300	0.51007800
H	5.28354800	-1.79134200	0.99505000
C	5.48887300	-0.62633300	-0.80777900
H	5.60534300	-1.57922600	-1.34524900
C	5.83622900	1.76293900	2.75559900
H	5.19172800	2.54327900	2.34261200
H	6.80166000	2.20311200	3.01052300
H	5.36588400	1.36546500	3.65770900
C	2.59942600	-1.70394900	1.21148200
H	2.78161000	-1.10148500	2.10729400
C	1.20836500	-1.46769600	0.71367300
H	0.94342100	-1.95163600	-0.22270100
C	0.30678700	-0.71183400	1.34732300
H	0.57100500	-0.22482900	2.28562300
C	-1.02841500	-0.48595300	0.83122500
H	-1.27217500	-0.97786200	-0.10742800
C	-1.96133900	0.29008900	1.41453700
H	-1.73244200	0.79469400	2.35284200
C	-3.26899300	0.49580500	0.83922000
H	-3.46643200	-0.03733200	-0.08861200
C	-4.23946400	1.27011500	1.36459900
H	-4.05943300	1.80853300	2.29455000
C	-5.53100200	1.42710300	0.74270700
H	-5.70828500	0.85917500	-0.17048200

C	-6.52518600	2.20629700	1.21542500
H	-6.37056700	2.77056300	2.13447100
C	-7.79806900	2.35524100	0.55546800
H	-7.94143600	1.79657100	-0.36927500
C	-8.80664800	3.12899100	1.00491400
H	-8.68191300	3.67633300	1.93860000
C	-10.06154500	3.28389300	0.31008200
H	-10.15245600	2.78159300	-0.65146500
C	-11.11676600	3.98084200	0.76977300
H	-11.05547400	4.46958800	1.74149300
C	2.90309300	-3.17823900	1.51058200
H	3.91963500	-3.24433200	1.90775900
H	2.22582500	-3.54262200	2.28859300
C	-12.36932500	4.07956100	0.04634000
H	-12.36435000	3.66872700	-0.96127600
C	-13.50805900	4.57268200	0.54503400
H	-13.51608900	4.96800000	1.56220900
C	-14.82538300	4.58229100	-0.17646200
H	-14.66049500	4.29927800	-1.22237600
C	-15.79582700	3.54792700	0.42663600
H	-16.07492900	3.90812200	1.43083200
C	-15.20706600	2.13738100	0.61635600
H	-14.33466400	2.26419400	1.26468400
C	-14.66304700	1.52842400	-0.68075300
H	-14.06470600	2.26424900	-1.21097200
C	-12.52607000	0.43817300	-0.76951100

C	-11.70917500	-0.71427100	-0.24109000
H	-12.10946700	-1.03031100	0.72405600
H	-11.83148200	-1.54799400	-0.94236200
C	-10.22136200	-0.37034200	-0.13585100
H	-10.09427500	0.46763800	0.56435100
C	-9.41515000	-1.56386200	0.35573100
H	-9.78879800	-1.85754500	1.34258500
H	-9.57756500	-2.40735400	-0.32599400
C	-7.91354900	-1.30538300	0.45371500
H	-7.73506300	-0.41105200	1.06627100
C	-7.18733500	-2.48813600	1.08590000
H	-7.52934900	-3.41196500	0.60317300
H	-7.50305800	-2.54640400	2.13201100
C	-5.65545100	-2.39053800	0.99802600
H	-5.34779400	-1.35326800	0.84527700
H	-5.20607900	-2.70289100	1.94576900
C	-5.04148600	-3.26630600	-0.09290400
H	-5.21270000	-4.31899300	0.17224000
C	-3.51499200	-3.03231000	-0.21640200
H	-3.12247500	-2.63882600	0.72842600
C	-2.74307900	-4.29279500	-0.59263400
H	-3.10936900	-4.66617700	-1.55664100
H	-2.94095400	-5.06573500	0.15901600
C	-1.23827100	-4.05170000	-0.66566800
H	-0.89926500	-3.63560300	0.29273500
C	-0.45832400	-5.33420800	-0.92868400

H	-0.79320500	-5.78372400	-1.86852700
H	-0.66172600	-6.03964500	-0.11770700
C	1.05189400	-5.11095600	-1.02064600
C	1.84506300	-6.41667500	-1.08865700
H	1.63915900	-6.92675200	-2.03185000
H	1.53253000	-7.06773200	-0.26426000
C	3.34099000	-6.14615500	-0.97104600
H	3.65871600	-5.54183000	-1.82541500
C	3.60904500	-5.34185400	0.31488400
H	3.30577700	-5.94950400	1.17654900
C	2.76767000	-4.06187400	0.27811700
H	3.06514700	-3.48881900	-0.60416500
C	5.07903800	-5.05147800	0.43245000
C	-15.67923600	0.92390600	-1.62787800
H	-15.18380700	0.57967400	-2.53859000
H	-16.41935500	1.68276100	-1.88510800
H	-16.18792700	0.07287000	-1.16998100
C	-16.19235100	1.21384900	1.33053700
H	-17.12400500	1.09829800	0.76855200
H	-16.44129500	1.60584800	2.32090300
H	-15.76371300	0.21764400	1.46076100
C	-15.46252400	5.97453700	-0.15675100
H	-15.61879000	6.31277400	0.87365100
H	-16.42949400	5.95801200	-0.66133700
H	-14.81517200	6.70175200	-0.65202900
O	4.74167300	0.29864300	-1.57062100

H	3.81021100	0.08534900	-1.41682700
O	7.96026500	1.71622800	0.74179300
H	8.42300600	1.68463100	-0.11441400
O	4.74459300	0.15832900	1.39502500
O	3.46590400	-1.24306200	0.16163400
O	1.40243300	-4.36732400	-2.15063000
H	0.73109300	-3.66703000	-2.24400700
O	1.39576800	-4.42228100	0.17900300
O	5.60295800	-4.05510800	-0.05051700
O	5.74243400	-5.97644700	1.10641900
H	6.70250100	-5.71779600	1.14575400
O	-0.91879900	-3.12382800	-1.70575300
H	-1.66595400	-2.50184700	-1.78268400
O	-3.30397500	-2.01406000	-1.19627000
H	-4.07711500	-2.12332000	-1.78860600
O	-5.60168200	-3.04718000	-1.37949300
H	-6.29152500	-2.35168500	-1.31792000
O	-7.34769700	-1.08169500	-0.84091800
H	-8.00351300	-0.58948800	-1.36808700
O	-9.70357700	0.00077400	-1.41081700
H	-10.32436500	0.64557600	-1.79492900
O	-12.09802000	1.23904700	-1.58569200
O	-13.75078600	0.46315700	-0.26519500
O	-16.94538900	3.54093900	-0.41454000
H	-17.67885400	3.14355400	0.06690500
O	4.11167100	-7.32901000	-1.03288700

H	4.03995200	-7.78346300	-0.18424400
N	7.54855400	0.50765000	-1.70673900
H	6.86977200	0.95851200	-2.31534000
H	7.97604500	-0.24818500	-2.23027500

AmB-Ret10

0 1

C	11.93393700	-4.98030400	-0.38820500
C	11.96225500	-5.32280500	1.10739900
C	12.99526000	-6.43153100	1.39061500
C	14.31951300	-6.18406000	0.68003100
C	14.10166800	-6.15613500	-0.82939900
C	12.90213900	-5.34110300	-1.24613800
H	13.14781000	-6.51339800	2.47478600
H	14.74862400	-5.22923500	1.01161100
H	15.05120900	-6.95815500	0.94406300
H	14.99983600	-5.77005700	-1.33256200
H	13.96840300	-7.18298200	-1.20708700
H	12.58614100	-7.39773100	1.06189700
C	12.91031200	-4.96686000	-2.70314800
H	11.92674800	-4.67670200	-3.07633400
H	13.58871000	-4.12188700	-2.87725100
H	13.26834200	-5.79852000	-3.31988300
C	12.31643600	-4.05492100	1.90742400

H	11.55297300	-3.28097800	1.78617800
H	12.38853400	-4.27786700	2.97671600
H	13.27088600	-3.63158000	1.58357200
C	10.58540200	-5.82838500	1.56514100
H	10.62673800	-6.15753600	2.60817800
H	9.82270800	-5.04850100	1.49260500
H	10.25236600	-6.67171300	0.95248600
C	10.78079000	-4.14734000	-0.80931300
H	10.78560000	-3.11678800	-0.45184800
C	9.75022700	-4.58435400	-1.53790100
H	9.71958500	-5.62808500	-1.85027400
C	8.59701900	-3.75453400	-1.93242800
C	8.76683900	-2.47373500	-2.31065700
H	9.78156700	-2.07952800	-2.34783300
C	7.27062400	-4.45637300	-1.85847200
H	7.25288300	-5.32544300	-2.52730500
H	7.09237100	-4.83032100	-0.84359200
H	6.42615700	-3.81786400	-2.12525400
C	7.71639500	-1.52644200	-2.65234000
H	6.71995400	-1.92628400	-2.82530400
C	7.92603800	-0.20522400	-2.73595100
H	8.92841300	0.18892200	-2.56909800
C	6.88022600	0.79692600	-3.01375100
C	7.18366300	1.90946200	-3.70068100
H	8.19391900	2.03506400	-4.08547000
C	5.51733900	0.51208800	-2.45321000

H	5.07319700	-0.37345100	-2.92189300
H	5.57790800	0.30861000	-1.37684200
H	4.82993300	1.34841100	-2.58250000
C	6.22929700	3.03658400	-3.97929200
H	5.36363700	2.70733100	-4.56895900
H	6.73290300	3.84337500	-4.53011400
O	5.66705700	3.57969600	-2.77529300
H	6.32464500	3.52502200	-2.06429300
C	7.12105600	0.53704800	2.91719500
H	7.95044800	1.02081600	2.37594400
C	6.94224100	-0.85757500	2.32477800
H	6.23272100	-1.44204000	2.93261500
C	6.46010700	-0.78215300	0.87940700
H	7.21277600	-0.25764700	0.26912400
C	5.26671700	1.29122500	1.33700800
H	5.80920200	1.93297200	0.62944100
C	5.84099300	1.37646400	2.75715400
H	6.06131100	2.43203400	2.98972500
C	6.14132800	-2.14473400	0.30263000
H	5.22079800	-2.53010600	0.75004600
H	6.95858200	-2.84076600	0.51467900
H	6.00735500	-2.09496400	-0.78083600
C	3.17493900	1.66384800	0.20937600
H	3.44492100	0.73412700	-0.30646500
C	1.73091800	1.68619600	0.61447100
H	1.43380000	2.54632500	1.21192800

C	0.83966900	0.75961000	0.25610000
H	1.15420100	-0.10636100	-0.32629300
C	-0.57403100	0.84465000	0.58925800
H	-0.90550500	1.75234100	1.09571400
C	-1.46504200	-0.11870500	0.30047400
H	-1.13355500	-1.03575800	-0.18541100
C	-2.87908700	-0.01429400	0.60406200
H	-3.21257200	0.93202900	1.03610600
C	-3.76222300	-1.00295300	0.37351000
H	-3.41832200	-1.95001000	-0.04021000
C	-5.17909300	-0.89706700	0.65700900
H	-5.54149700	0.07593500	0.99471800
C	-6.04193600	-1.92144800	0.52052000
H	-5.67832700	-2.89452200	0.19319600
C	-7.45901500	-1.82616700	0.80684100
H	-7.81981800	-0.86127400	1.16701600
C	-8.31612700	-2.85358400	0.66101600
H	-7.94880100	-3.81881800	0.31434700
C	-9.73347400	-2.76685600	0.95011100
H	-10.08800100	-1.82932100	1.38085400
C	-10.61254500	-3.75667300	0.71758200
H	-10.27617400	-4.70471100	0.29977800
C	3.50891800	2.87800300	-0.64885700
H	4.53091200	2.83963300	-1.03024600
H	2.86523200	2.85772600	-1.53828900
C	-12.03721300	-3.61920000	0.98186900

H	-12.32857300	-2.73380800	1.54897100
C	-12.97277900	-4.46681100	0.54303800
H	-12.69243100	-5.35799100	-0.01909100
C	-14.44548100	-4.26730400	0.75890600
H	-14.61127500	-3.47165000	1.50235900
C	-15.10858100	-3.80633600	-0.55023500
H	-15.17947700	-4.65737900	-1.24996900
C	-14.33212200	-2.67316700	-1.24280600
H	-13.30451400	-3.04572900	-1.37870500
C	-14.19723200	-1.42593000	-0.35836800
H	-13.88038000	-1.70942800	0.65073000
C	-12.01301300	-0.42074000	-0.19154300
C	-10.95217100	0.31978800	-0.96000000
H	-11.01459500	0.05450400	-2.02212100
H	-11.17697100	1.39507500	-0.89693100
C	-9.54561800	0.07288400	-0.40345900
H	-9.31794000	-1.00642600	-0.40805000
C	-8.49681600	0.86133700	-1.17682700
H	-8.63171800	0.69918000	-2.25468800
H	-8.68172500	1.92919900	-0.99072800
C	-7.04294900	0.56051600	-0.80275500
H	-6.84139000	-0.52422100	-0.82172200
C	-6.09413800	1.30024400	-1.74228400
H	-6.45923600	2.33196000	-1.84586600
H	-6.16668100	0.84677300	-2.73929000
C	-4.62824100	1.32135200	-1.27581000

H	-4.45733200	0.58074900	-0.48113700
H	-3.95751300	1.03553200	-2.09573900
C	-4.23602200	2.69357600	-0.74178100
H	-4.34978000	3.46016200	-1.52387300
C	-2.79037300	2.74412900	-0.18164200
H	-2.14983100	1.96789800	-0.62460100
C	-2.18643700	4.13580100	-0.37831100
H	-2.83517100	4.87359000	0.11687100
H	-2.18193400	4.38478900	-1.44832500
C	-0.77662600	4.26825200	0.18798100
H	-0.13845000	3.44147500	-0.16310000
C	-0.12355100	5.60597400	-0.15151700
H	-0.66132600	6.41160800	0.36547700
H	-0.18820500	5.80083300	-1.22884800
C	1.33006900	5.63832000	0.30098900
C	2.12097200	6.86344800	-0.13207800
H	1.73496200	7.75400400	0.37751800
H	2.00826600	7.03569600	-1.20978700
C	3.59907400	6.70712400	0.19501200
H	3.74899000	6.76617800	1.28216600
C	4.16322100	5.36777500	-0.33861000
H	4.16346700	5.41747400	-1.44197200
C	3.24753700	4.18775800	0.07623200
H	3.27464700	4.06235700	1.16780300
C	5.59306900	5.12981700	0.08594500
C	-15.36351700	-0.46244500	-0.30294100

H	-15.16126600	0.34065000	0.41147400
H	-16.26500000	-0.98844800	0.02114700
H	-15.55089400	-0.00455000	-1.27730100
C	-14.90194300	-2.36918300	-2.62983800
H	-15.95822500	-2.08416900	-2.59612800
H	-14.80878100	-3.23640600	-3.28990200
H	-14.36071100	-1.53923800	-3.09357500
C	-15.11589500	-5.54491700	1.27544400
H	-14.96172100	-6.38238900	0.58694800
H	-16.19286300	-5.39318000	1.38765500
H	-14.70799700	-5.83189500	2.24876300
O	4.89407400	0.93181000	3.71813100
H	4.09384100	0.66331900	3.23290100
O	8.20143400	-1.51808700	2.28979600
H	8.59370400	-1.38534200	3.17007800
O	5.22557800	-0.04169300	0.83106200
O	3.92488500	1.69711600	1.45200400
O	1.43123500	5.56035700	1.69899000
H	0.72760800	4.95239000	2.01353000
O	1.90132100	4.47884800	-0.29680600
O	6.26698200	4.13061800	-0.05724900
O	6.15381800	6.20516700	0.70538200
H	7.07122900	5.96916100	0.93617900
O	-0.81667300	4.21517700	1.62438200
H	-1.37992800	3.45032600	1.86331600
O	-2.83220000	2.39790300	1.20725800

H	-3.66932000	2.80029700	1.53088400
O	-5.10032500	3.10322000	0.32220000
H	-5.70474000	2.35629000	0.55485900
O	-6.75378900	1.00183500	0.53654900
H	-7.58888200	0.99719600	1.04930000
O	-9.47426500	0.54992000	0.94503700
H	-10.16708800	0.09117300	1.45905400
O	-11.92636100	-0.73240200	0.98183300
O	-13.07452100	-0.68623500	-0.96068100
O	-16.44157900	-3.42361300	-0.18084200
H	-16.94281800	-3.22240400	-0.98242100
O	4.24148500	7.80483900	-0.44956500
H	5.13471000	7.87713700	-0.08290800
N	7.53261100	0.37358500	4.31424600
H	6.71802200	0.06129300	4.84046200
H	7.80381400	1.26859800	4.71134700

AmB-Ret11

0 1

C	3.94517900	-3.35679100	1.88188600
C	3.12127000	-4.51781900	1.30036900
C	2.00082000	-4.91171800	2.27575800
C	1.18286800	-3.71847400	2.74229100
C	2.08261400	-2.69890500	3.43326700

C	3.45425900	-2.52373100	2.82167500
H	1.35688500	-5.65699000	1.79502200
H	0.67408000	-3.25760400	1.89281000
H	0.39613300	-4.04359000	3.43050100
H	1.58619500	-1.72121800	3.46729200
H	2.23236200	-2.97948100	4.48643400
H	2.45638700	-5.40216000	3.14561400
C	4.20121400	-1.35542300	3.41164000
H	5.05047200	-1.03504500	2.81214800
H	3.52791800	-0.50071500	3.52742400
H	4.56996900	-1.60198900	4.41475100
C	2.52578400	-4.09736900	-0.05550800
H	3.31096800	-3.79007500	-0.75440200
H	1.99152900	-4.93634200	-0.51406900
H	1.82362700	-3.26850400	0.05335300
C	4.00712100	-5.75683600	1.09087200
H	3.38984700	-6.60517300	0.77908900
H	4.76686300	-5.60226800	0.32091200
H	4.52149900	-6.03270700	2.01577900
C	5.29494600	-3.21961500	1.30667100
H	5.36458700	-3.37380300	0.23018800
C	6.42878500	-3.00897000	1.99388600
H	6.35480700	-2.96199600	3.07763400
C	7.79058000	-2.84613700	1.46634600
C	8.04384000	-2.62828200	0.15825500
H	7.19890700	-2.57320800	-0.52355800

C	8.86146600	-2.90595900	2.52455400
H	8.67380400	-2.14883800	3.29386100
H	8.84519800	-3.87777900	3.02905600
H	9.86647400	-2.74289800	2.13849100
C	9.33199700	-2.41667400	-0.46469700
H	10.21882600	-2.52465400	0.15036400
C	9.46703200	-2.06360300	-1.75497200
H	8.56501800	-1.95047800	-2.35500200
C	10.72455900	-1.77458000	-2.44309900
C	10.67608800	-1.35192500	-3.71777900
H	9.70044800	-1.25968900	-4.19297700
C	11.99732100	-1.93319800	-1.65470500
H	12.02577000	-1.21556500	-0.82804600
H	12.05691900	-2.93314700	-1.21492600
H	12.88979300	-1.77543700	-2.25853200
C	11.80580000	-0.97004000	-4.62249200
H	12.78324100	-1.21765800	-4.18871300
H	11.79886800	0.11354400	-4.78763100
O	11.65479600	-1.54329200	-5.91243800
H	11.61077900	-2.50020900	-5.79859700
C	-12.31761100	2.70335700	1.17336600
H	-13.04171600	2.15609100	0.55677800
C	-12.06676300	4.04481600	0.48607600
H	-11.35505700	4.61907400	1.09942500
C	-11.42507400	3.82924700	-0.87927000
H	-12.12213200	3.23310800	-1.49296100

C	-10.38589900	1.83517800	-0.14108800
H	-11.01776300	1.22596100	-0.80556500
C	-11.02779400	1.89258000	1.24236100
H	-11.24104600	0.85723700	1.54676200
C	-11.10012000	5.12625200	-1.58472400
H	-10.38220500	5.69850200	-0.99120800
H	-12.00940300	5.71743200	-1.70407500
H	-10.66067100	4.92980600	-2.56520300
C	-8.37279700	0.98952700	-1.14547400
H	-8.49876300	1.82489800	-1.84187800
C	-6.94562200	0.91545700	-0.70182000
H	-6.70571000	0.15243300	0.03404500
C	-5.98806300	1.73775700	-1.14136800
H	-6.22911800	2.50496200	-1.87663400
C	-4.62135900	1.68008900	-0.66303600
H	-4.40017300	0.91283600	0.07491400
C	-3.63287200	2.51072800	-1.04541000
H	-3.83726900	3.28945500	-1.77968700
C	-2.29530300	2.42202100	-0.51011900
H	-2.12724700	1.62252800	0.20882400
C	-1.26465300	3.22643600	-0.83996000
H	-1.41209400	4.02978900	-1.56096900
C	0.05154400	3.06903100	-0.27209000
H	0.18481300	2.24193300	0.42522000
C	1.11941800	3.84486100	-0.55064100
H	1.01634500	4.66731700	-1.25759400

C	2.41070200	3.64602800	0.05925500
H	2.49193700	2.83016000	0.77641500
C	3.51190000	4.38041100	-0.19789700
H	3.46173500	5.18582800	-0.92978000
C	4.77612200	4.15651600	0.46209900
H	4.78699700	3.39036200	1.23534600
C	5.92402100	4.80290200	0.18816000
H	5.94424800	5.55626700	-0.59844400
C	-8.90010400	-0.29731200	-1.79287400
H	-9.93861000	-0.12947100	-2.08719600
H	-8.33183100	-0.50720400	-2.70398800
C	7.16643400	4.51841500	0.88014500
H	7.08776200	3.83303800	1.72246300
C	8.36920900	4.99088300	0.53464500
H	8.44990900	5.66468900	-0.31993200
C	9.65841100	4.63524500	1.21968800
H	9.43136800	4.08131300	2.13760700
C	10.52961700	3.71560700	0.34171400
H	10.89773800	4.32013600	-0.50399200
C	9.79214000	2.51035000	-0.26750600
H	8.97533100	2.93044400	-0.86238700
C	9.11191500	1.62854700	0.78484600
H	8.60730700	2.25071700	1.51862900
C	6.87042100	0.81155700	0.61212100
C	5.90079900	0.03327700	-0.23148800
H	6.29002700	-0.06825400	-1.24570200

H	5.84087800	-0.96175400	0.21762000
C	4.49448400	0.63365200	-0.23911600
H	4.51476200	1.62772100	-0.70706100
C	3.54592700	-0.27635500	-1.00686800
H	3.87304500	-0.32236100	-2.05119300
H	3.61070300	-1.28798200	-0.58926800
C	2.08587000	0.15817700	-0.95761100
H	2.00114700	1.21607100	-1.23823000
C	1.22800000	-0.67300300	-1.90649700
H	1.48948800	-1.73180300	-1.79220200
H	1.50887300	-0.39148900	-2.92591100
C	-0.28268400	-0.48152400	-1.70374000
H	-0.48323700	0.47969000	-1.22428500
H	-0.78915400	-0.44768600	-2.67333100
C	-0.94618900	-1.59741800	-0.89921900
H	-0.89514200	-2.52572900	-1.48360800
C	-2.43206500	-1.27796800	-0.60353800
H	-2.81435700	-0.57525100	-1.35252700
C	-3.32013200	-2.51693700	-0.58070300
H	-2.96168500	-3.20499200	0.19464800
H	-3.23383000	-3.03085500	-1.54502400
C	-4.78709300	-2.17619100	-0.33435600
H	-5.11254300	-1.44441000	-1.08627100
C	-5.68699800	-3.39990700	-0.44101800
H	-5.36032000	-4.16253500	0.27267900
H	-5.59649000	-3.81274200	-1.44988300

C	-7.15979900	-3.09598200	-0.16355100
C	-8.06962300	-4.27126500	-0.49012100
H	-7.85739400	-5.11073800	0.17521100
H	-7.88755600	-4.59106400	-1.51999500
C	-9.53651500	-3.89716100	-0.36580900
H	-9.75854200	-3.68724700	0.69067700
C	-9.83140000	-2.60981800	-1.18378000
H	-9.77984700	-2.89062700	-2.24236400
C	-8.80589400	-1.50926700	-0.87337100
H	-8.95303300	-1.19030600	0.16259100
C	-11.22926600	-2.13424800	-0.88310700
C	9.98763400	0.62084500	1.50047500
H	9.40242700	0.08989600	2.25457700
H	10.81241300	1.14627200	1.98420400
H	10.38722800	-0.11361200	0.79941400
C	10.69613700	1.71566400	-1.20826000
H	11.57900900	1.32614400	-0.69124900
H	11.04104900	2.34369100	-2.03516300
H	10.16630200	0.86071300	-1.63263900
C	10.44618300	5.88991500	1.60759200
H	10.66788100	6.49581800	0.72199800
H	11.39084700	5.61454000	2.07867000
H	9.87071800	6.50878000	2.29978900
O	-10.16872300	2.52721300	2.16717100
H	-9.26855400	2.27215200	1.91983800
O	-13.28604300	4.73245300	0.32460600

H	-13.75536500	4.57941600	1.16073300
O	-10.20507600	3.11652900	-0.70255200
O	-9.12966200	1.26765900	0.04508700
O	-7.37997300	-2.75422700	1.17390000
H	-6.64253000	-2.17381400	1.43649800
O	-7.49054100	-2.01959700	-1.03560000
O	-11.54379800	-1.10654400	-0.33145500
O	-12.17162000	-3.02984300	-1.27164300
H	-13.02890900	-2.66106600	-1.00265400
O	-4.96961300	-1.60934700	0.96566100
H	-4.16621300	-1.09931900	1.17651000
O	-2.49914100	-0.59963000	0.65289400
H	-1.73521000	-0.96701100	1.14146100
O	-0.31134400	-1.84453100	0.34948600
H	0.41982400	-1.19594000	0.47603000
O	1.56719400	0.00782700	0.36985800
H	2.28404500	0.24192900	0.98832600
O	3.99373900	0.76186300	1.09001700
H	4.73322300	1.04553900	1.65957700
O	6.56708300	1.28785900	1.69767800
O	8.07145000	0.89945100	0.06656700
O	11.62805200	3.31476000	1.15637400
H	12.32699000	2.97851700	0.58534900
O	-10.26833300	-5.00841600	-0.83538400
H	-11.20182900	-4.76423900	-0.87034000
N	-12.94205800	2.97731300	2.46477600

H	-12.21124500	3.26599000	3.11165200
H	-13.35637300	2.13377600	2.84842100

AmB-Ret12

0 1

C	7.27275800	7.83226700	0.90265600
C	7.67641600	8.56244300	2.19418600
C	8.18879300	9.97767900	1.88225200
C	7.28928700	10.72188600	0.90844500
C	7.23408700	9.96770700	-0.41311000
C	7.05166600	8.47416600	-0.26047600
H	8.29908200	10.53283200	2.82081700
H	6.27959400	10.80923700	1.32348800
H	7.65068700	11.74278700	0.74933100
H	6.42922300	10.36393100	-1.04329500
H	8.16016500	10.14314200	-0.98050900
H	9.19189900	9.89946200	1.44380600
C	6.61009000	7.80253200	-1.53470600
H	6.27918700	6.77662300	-1.38347600
H	5.79109200	8.36858700	-1.99203100
H	7.42365000	7.78745600	-2.27041000
C	6.46702500	8.63800300	3.14416600
H	6.08841400	7.64233200	3.38985400
H	6.75138900	9.12162100	4.08463900

H	5.64312800	9.20376400	2.70320100
C	8.80807600	7.80022600	2.90179200
H	9.15060400	8.36513500	3.77492400
H	8.48706600	6.81398300	3.24428100
H	9.66046100	7.65739500	2.23141000
C	7.12275400	6.37188800	1.05412800
H	6.56349400	6.03084200	1.92416500
C	7.68207400	5.44092800	0.26984000
H	8.34202600	5.77337700	-0.53065900
C	7.52904600	3.98756800	0.41603600
C	6.37873000	3.45186600	0.87299000
H	5.55249400	4.13296600	1.06755700
C	8.73281500	3.18109300	0.00390600
H	9.06060900	3.47923500	-0.99808100
H	9.57617300	3.36589800	0.67862300
H	8.54330100	2.10819200	-0.01750500
C	6.09992500	2.05215600	1.11487600
H	6.90262600	1.33893600	0.96155800
C	4.89617000	1.60281100	1.50601800
H	4.09296600	2.32526400	1.64511800
C	4.53923200	0.20125200	1.72174400
C	3.25709400	-0.09630600	1.98010600
H	2.53574800	0.71579300	2.04771400
C	5.62642600	-0.82916200	1.58908400
H	5.28948000	-1.82755000	1.85894200
H	5.99136500	-0.84049300	0.55773100

H	6.46961000	-0.57827600	2.24081100
C	2.64334500	-1.44743700	2.15494000
H	2.50461900	-1.66532300	3.22558000
H	3.26435100	-2.24339900	1.73066300
O	1.36447200	-1.40050600	1.51141300
H	0.95994900	-2.27473400	1.55297000
C	7.19173600	-5.48986300	2.30843400
H	7.84406500	-5.76990400	1.47188900
C	6.45674700	-6.75397600	2.75349100
H	5.83426700	-6.49682500	3.62780200
C	5.52028200	-7.24881700	1.65723700
H	6.13332900	-7.51701900	0.78144500
C	5.29949600	-5.06315600	0.75946400
H	5.91426000	-5.38363400	-0.09447100
C	6.19170000	-4.43846000	1.82155600
H	6.71489700	-3.58377800	1.38414500
C	4.68707800	-8.43411400	2.08721800
H	4.04964300	-8.15335900	2.93014000
H	5.34262000	-9.24865000	2.39936700
H	4.04877400	-8.77237000	1.26834200
C	3.57371800	-4.47076400	-0.77792300
H	3.36088500	-5.54575900	-0.76403200
C	2.29510400	-3.70875600	-0.64691600
H	2.39149000	-2.62923100	-0.57962100
C	1.08893200	-4.28309900	-0.58021400
H	1.00287300	-5.36823800	-0.63303200

C	-0.12931000	-3.51922000	-0.39798600
H	-0.02673200	-2.43786100	-0.36827400
C	-1.36516300	-4.02865100	-0.23295400
H	-1.52630200	-5.10594500	-0.26694900
C	-2.50042200	-3.17120500	0.01558100
H	-2.27210900	-2.10873400	0.07502900
C	-3.77629700	-3.57341200	0.17594400
H	-4.02848300	-4.63132000	0.10950300
C	-4.84493000	-2.64390200	0.44923800
H	-4.56967200	-1.59156800	0.52198300
C	-6.14004300	-2.98043000	0.61551700
H	-6.43814700	-4.02462600	0.52697100
C	-7.16966700	-2.02274400	0.93360900
H	-6.85449000	-0.98740600	1.06287600
C	-8.47523700	-2.32272400	1.08532200
H	-8.80707700	-3.34983400	0.93731000
C	-9.47607800	-1.34860700	1.44733900
H	-9.12061200	-0.34355500	1.66735000
C	-10.79862700	-1.58635900	1.51638800
H	-11.18137900	-2.57646900	1.27148200
C	4.38613100	-4.12875100	-2.03357500
H	5.23992700	-4.80907300	-2.08253600
H	3.77474800	-4.30989800	-2.92272100
C	-11.76129200	-0.55891600	1.86380500
H	-11.33847300	0.37805800	2.22051900
C	-13.08706600	-0.66431500	1.72403100

H	-13.51033800	-1.59750000	1.34872300
C	-14.06648600	0.44100600	2.00018300
H	-13.54683400	1.25582200	2.51718100
C	-14.63324700	1.02197000	0.68998600
H	-15.26569400	0.24247000	0.23325000
C	-13.57714600	1.40119800	-0.36552700
H	-13.01838300	0.48314200	-0.57163300
C	-12.53623000	2.40178700	0.14841200
H	-12.18037300	2.09276400	1.12735700
C	-10.18927400	2.10566900	-0.26488900
C	-9.13052900	2.01855300	-1.33472800
H	-9.59088100	1.71896900	-2.27802400
H	-8.72005200	3.02619800	-1.46853700
C	-7.99195700	1.06963500	-0.95139400
H	-8.39856800	0.05770400	-0.80979000
C	-6.92333100	1.03641900	-2.03434500
H	-7.37997500	0.68539400	-2.96597700
H	-6.55976000	2.05664400	-2.20643600
C	-5.72311800	0.14998500	-1.70965000
H	-6.07366000	-0.85679900	-1.44157600
C	-4.78240400	0.04532800	-2.90566000
H	-4.61472500	1.05175100	-3.30853200
H	-5.30408200	-0.51999300	-3.68418400
C	-3.43063300	-0.61256300	-2.58315300
H	-3.50053000	-1.19946900	-1.66420100
H	-3.16169700	-1.31739900	-3.37581300

C	-2.27924700	0.38428200	-2.46054700
H	-2.13711000	0.86132900	-3.44153700
C	-0.96050000	-0.31020400	-2.05876100
H	-0.95536800	-1.33615900	-2.44787200
C	0.27297100	0.41644000	-2.58171000
H	0.26945800	1.45452800	-2.23137100
H	0.22097400	0.43615200	-3.67740100
C	1.57574200	-0.25102300	-2.14380200
H	1.46279100	-1.34032500	-2.22368600
C	2.75647100	0.15418600	-3.01956400
H	2.89196000	1.23947500	-2.97620600
H	2.54108500	-0.12172500	-4.05565400
C	4.07324400	-0.49871900	-2.59755400
C	5.19866500	-0.25732200	-3.59247600
H	5.42258700	0.80939200	-3.65887800
H	4.89211900	-0.61126400	-4.58080400
C	6.45710100	-1.00169200	-3.18366600
H	6.83087700	-0.56569300	-2.24605000
C	6.13510000	-2.50117500	-2.93130100
H	5.98509700	-2.95252200	-3.91942100
C	4.87894900	-2.68601300	-2.06370300
H	5.11902700	-2.36695300	-1.04681600
C	7.31986000	-3.16702200	-2.28000400
C	-12.95557000	3.85681100	0.19669900
H	-12.15761400	4.46190200	0.63318300
H	-13.85333700	3.94796400	0.80945500

H	-13.16873400	4.23914400	-0.80388500
C	-14.23148500	1.85416100	-1.66985000
H	-14.86441700	2.73520700	-1.52608600
H	-14.85239100	1.05662400	-2.08809400
H	-13.47339600	2.11508300	-2.41163800
C	-15.20855800	-0.03961100	2.89981700
H	-15.74055500	-0.87640900	2.43358000
H	-15.92362000	0.76579200	3.07249300
H	-14.82197000	-0.38455400	3.86151800
O	5.41166600	-3.96262600	2.90863700
H	4.59920600	-4.48819500	2.91554100
O	7.38068800	-7.76560000	3.07243400
H	8.08003500	-7.30343100	3.56234800
O	4.62700500	-6.18949100	1.30914500
O	4.33922800	-4.13851500	0.39424600
O	4.51263000	-0.03987000	-1.35802100
H	3.71812300	0.03329000	-0.79016900
O	3.80937200	-1.90522000	-2.56869300
O	7.35860300	-3.66644900	-1.17892500
O	8.42724900	-3.12996500	-3.06083400
H	9.14168600	-3.54059700	-2.54695700
O	1.91939400	0.09979400	-0.80712700
H	1.35406100	-0.36298400	-0.16322900
O	-0.91978400	-0.42577300	-0.64451800
H	-1.31444200	0.40926000	-0.33459000
O	-2.51826100	1.40826000	-1.50697800

H	-3.39439600	1.26576000	-1.09628600
O	-4.98026600	0.67613200	-0.60749000
H	-5.61841100	1.05636500	0.02219000
O	-7.36824200	1.49755800	0.25612100
H	-8.07833800	1.69238100	0.89318700
O	-9.93961100	2.02705800	0.92762200
O	-11.40081000	2.29583100	-0.76688600
O	-15.44115100	2.13423800	1.06283500
H	-16.02337300	2.35298900	0.32740300
O	7.38449500	-0.83204800	-4.23415900
H	8.17031900	-1.35400500	-4.02938800
N	8.03447300	-5.06002300	3.41904500
H	7.44302500	-4.56472200	4.08372900
H	8.73715400	-4.39958200	3.10221800

AmB-Ret13

0 1

C	-13.05597700	0.50583100	-0.52232800
C	-13.47295000	0.86191300	-1.95925200
C	-14.33303600	2.13663700	-1.98017100
C	-13.77714400	3.22458700	-1.07460600
C	-13.80304100	2.72993600	0.36443600
C	-13.23388800	1.33772700	0.52619800
H	-14.42264300	2.48617700	-3.01419500

H	-12.74527600	3.46284700	-1.35311400
H	-14.35791900	4.14675300	-1.17061300
H	-13.24478300	3.41444900	1.01413800
H	-14.83313000	2.73331700	0.74860800
H	-15.34786500	1.88739300	-1.64454900
C	-12.89542500	1.00625000	1.95742300
H	-12.39582800	0.04757900	2.07697100
H	-12.25875700	1.78719400	2.38905400
H	-13.81006900	0.98883900	2.56144400
C	-12.23617800	1.05572200	-2.85667000
H	-11.62711100	0.14904700	-2.90175900
H	-12.55430700	1.27829800	-3.87997500
H	-11.59916300	1.86978800	-2.50695700
C	-14.31281900	-0.29135200	-2.53215600
H	-14.68722000	-0.02229600	-3.52481500
H	-13.72833800	-1.20946300	-2.62719200
H	-15.17276700	-0.50689700	-1.89102700
C	-12.39806300	-0.81165100	-0.37912400
H	-11.51872300	-0.96415100	-1.00231200
C	-12.77653000	-1.82740700	0.40587900
H	-13.71824500	-1.75700700	0.94997100
C	-11.98017200	-3.04959700	0.60619400
C	-10.64398300	-2.93030900	0.73218600
H	-10.24881400	-1.92088800	0.72320900
C	-12.74104000	-4.34186300	0.70191900
H	-13.53130500	-4.26225000	1.45655300

H	-13.23541900	-4.57286500	-0.24762000
H	-12.10758200	-5.18798900	0.97035700
C	-9.63904400	-3.95237700	0.94351400
H	-9.93581000	-4.99717600	0.94473000
C	-8.35762100	-3.60917000	1.16193000
H	-8.10885300	-2.54858700	1.15155400
C	-7.23337700	-4.50053000	1.43974500
C	-6.07290100	-3.93629900	1.81160100
H	-6.04142600	-2.85114600	1.88857000
C	-7.46455900	-5.98219500	1.32418000
H	-8.17955300	-6.31683300	2.08269100
H	-7.89403900	-6.22802700	0.34845400
H	-6.55158100	-6.56316100	1.45275000
C	-4.77515300	-4.58476800	2.17316100
H	-4.74897000	-5.64614300	1.89696000
H	-4.61522900	-4.53314400	3.25577000
O	-3.67501000	-3.88015300	1.60529300
H	-3.76239100	-3.92432100	0.64570600
C	13.53455400	3.12444100	-0.68683600
H	14.29510100	2.46186200	-0.25478200
C	13.21893500	4.18894900	0.36297200
H	12.46948800	4.87739000	-0.05760400
C	12.60656700	3.54684600	1.60198200
H	13.34250800	2.83654400	2.01648500
C	11.67104900	1.80144800	0.30702500
H	12.34511000	1.06267700	0.76675200

C	12.29107100	2.29778200	-0.99618600
H	12.55801000	1.41064800	-1.58950200
C	12.21996300	4.55939800	2.65586200
H	11.46551000	5.24054900	2.25335100
H	13.09721300	5.13853700	2.94848100
H	11.80289500	4.05968600	3.53301100
C	9.71903000	0.58609500	1.00963200
H	9.79456000	1.19423000	1.91692200
C	8.29714400	0.54648000	0.54559300
H	8.10460600	0.01250100	-0.38114800
C	7.28775900	1.13758500	1.19199600
H	7.47973300	1.67598500	2.11971900
C	5.92418900	1.12010600	0.70168900
H	5.75204700	0.58085500	-0.22647600
C	4.88244700	1.73347600	1.29454800
H	5.03806600	2.28507800	2.22138700
C	3.54890400	1.70390400	0.74291000
H	3.42865500	1.12556900	-0.17112700
C	2.47165900	2.31785100	1.27231000
H	2.57517300	2.90141000	2.18660900
C	1.16313100	2.24492300	0.67080000
H	1.06653900	1.62329100	-0.21916200
C	0.06335300	2.87428600	1.13305000
H	0.13592400	3.49076800	2.02841300
C	-1.22165300	2.79764100	0.48433000
H	-1.28303800	2.17887800	-0.41081600

C	-2.33327700	3.43526300	0.90411900
H	-2.28972800	4.04392700	1.80663100
C	-3.59702500	3.36601100	0.21146500
H	-3.61345900	2.79286900	-0.71402100
C	-4.74233000	3.93662200	0.62858500
H	-4.75593100	4.49443800	1.56421700
C	10.33394700	-0.79536400	1.26997900
H	11.36176200	-0.65246900	1.61123800
H	9.78717100	-1.29163600	2.07733100
C	-5.99210300	3.80306600	-0.09436400
H	-5.92131200	3.31914700	-1.06661700
C	-7.19561400	4.16219300	0.36600300
H	-7.27263700	4.63108200	1.34836700
C	-8.49029700	3.90873100	-0.35275500
H	-8.26826400	3.56254400	-1.36879200
C	-9.30447000	2.79061900	0.33030200
H	-9.68698800	3.19484200	1.28254300
C	-8.49525500	1.52858500	0.68879600
H	-7.66714000	1.87446600	1.31454600
C	-7.83496300	0.87401400	-0.52791500
H	-7.37061200	1.63995900	-1.14220700
C	-5.56109300	0.12662000	-0.59018500
C	-4.56153200	-0.85815300	-0.03937300
H	-4.85034700	-1.16361900	0.96625900
H	-4.61083500	-1.74749800	-0.67933700
C	-3.13185400	-0.31156300	-0.04665200

H	-3.08408400	0.56029900	0.62170600
C	-2.13689900	-1.36201100	0.42111500
H	-2.44127400	-1.74529600	1.39855000
H	-2.17043600	-2.20591600	-0.27984000
C	-0.69824800	-0.86174200	0.48911000
H	-0.65455300	0.04760400	1.10460200
C	0.22531300	-1.91188800	1.09607800
H	0.03403900	-2.87552000	0.60829600
H	-0.06261400	-2.03352500	2.14442600
C	1.71699700	-1.55661600	0.98968700
H	1.84279000	-0.47979800	0.84992600
H	2.22820800	-1.80160600	1.92595000
C	2.45269100	-2.30136700	-0.12275400
H	2.46610900	-3.37096000	0.12876900
C	3.91756100	-1.81046900	-0.26326400
H	4.23882200	-1.33293100	0.66961400
C	4.89336000	-2.92844100	-0.61373900
H	4.58982900	-3.38763500	-1.56241500
H	4.83790800	-3.70153600	0.16137800
C	6.33398800	-2.43601100	-0.71607100
H	6.60685500	-1.93533500	0.22291500
C	7.31421600	-3.57811200	-0.94963900
H	7.03461400	-4.12556500	-1.85515700
H	7.25681300	-4.26647500	-0.10148300
C	8.76238300	-3.11475800	-1.11097200
C	9.74766600	-4.27447700	-1.12484800

H	9.58418100	-4.89913900	-2.00543400
H	9.59407500	-4.88710000	-0.23203800
C	11.18634800	-3.78819900	-1.12075800
H	11.38541700	-3.26813200	-2.06907000
C	11.40505000	-2.77596600	0.03679200
H	11.37685900	-3.35198000	0.96923500
C	10.31009100	-1.69824600	0.04233800
H	10.42942500	-1.08798400	-0.85769900
C	12.76886200	-2.14975800	-0.09712500
C	-8.70607400	-0.00928500	-1.39687600
H	-8.13875800	-0.34944200	-2.26647500
H	-9.56977800	0.56201900	-1.73609900
H	-9.04922700	-0.88877200	-0.84882500
C	-9.33266900	0.55653200	1.51366600
H	-10.22055500	0.22305100	0.97108100
H	-9.67044700	1.02825300	2.44063000
H	-8.75326400	-0.32915400	1.78390400
C	-9.33460200	5.18156800	-0.45809000
H	-9.55600100	5.58096400	0.53791600
H	-10.28061500	4.97088300	-0.95929900
H	-8.80168300	5.95431300	-1.01680300
O	11.38651400	3.12517200	-1.69829700
H	10.50516500	2.75773100	-1.54066700
O	14.40085900	4.86588400	0.72435100
H	14.86759700	4.99038100	-0.11796400
O	11.42546000	2.85037600	1.21804100

O	10.44710500	1.24132700	-0.04318700
O	8.95059800	-2.39055300	-2.29155600
H	8.17396200	-1.80895600	-2.38541500
O	9.03091800	-2.31510700	0.03713300
O	13.01635100	-0.99099400	-0.33270000
O	13.76744800	-3.05875300	0.03694600
H	14.59844600	-2.57656300	-0.10529400
O	6.48028200	-1.50361900	-1.79035600
H	5.63660400	-1.02142000	-1.87465600
O	3.94911400	-0.79679800	-1.26958900
H	3.18591700	-1.03618800	-1.83721600
O	1.84043100	-2.16589500	-1.39727300
H	1.04684000	-1.59288400	-1.31491300
O	-0.20585400	-0.53651300	-0.81670300
H	-0.95418900	-0.19011900	-1.33662500
O	-2.74176300	0.08178900	-1.36078200
H	-3.47690700	0.59639500	-1.73964500
O	-5.29858000	0.90108400	-1.49721300
O	-6.75047100	0.03584600	-0.00989100
O	-10.39250700	2.51252900	-0.54176700
H	-11.06634000	1.97578400	-0.10027000
O	11.99178500	-4.93899600	-0.98481200
H	12.90765300	-4.65669500	-0.86761800
N	14.12691500	3.79991800	-1.83825800
H	13.37300900	4.22433600	-2.37432400
H	14.58082000	3.13016600	-2.45132100

AmB-Ret14

0 1

C	-11.19029900	-0.96178300	-1.10011200
C	-9.90529800	-1.73880600	-0.76599100
C	-10.05786200	-3.23500700	-1.08652900
C	-10.73516100	-3.48540800	-2.42423000
C	-12.13239900	-2.88130800	-2.40741800
C	-12.17843300	-1.47180300	-1.86023600
H	-9.06526900	-3.70438600	-1.07347200
H	-10.15021600	-3.03051300	-3.23035900
H	-10.78430000	-4.55759800	-2.63843900
H	-12.55694100	-2.88686300	-3.41775600
H	-12.80377200	-3.51184500	-1.80576000
H	-10.65187700	-3.71355200	-0.29761400
C	-13.42347100	-0.71948700	-2.24992900
H	-13.32848200	0.35589200	-2.10539400
H	-13.66425100	-0.90875700	-3.30140000
H	-14.28861700	-1.05937900	-1.66731000
C	-8.73297200	-1.15801900	-1.57998800
H	-8.64290100	-0.07884700	-1.44349700
H	-7.78963800	-1.61347900	-1.26134800
H	-8.86439700	-1.33932900	-2.64947700
C	-9.59050900	-1.60952800	0.73484300

H	-8.64767500	-2.11433500	0.97055600
H	-9.49193400	-0.56707400	1.04376400
H	-10.39033700	-2.05097900	1.33699300
C	-11.20034800	0.40483900	-0.54827800
H	-10.24985700	0.93353300	-0.58913900
C	-12.21307300	1.02175500	0.07820800
H	-13.14130900	0.47747700	0.24264300
C	-12.14226600	2.37330300	0.65248800
C	-11.28908900	3.29240100	0.15546400
H	-10.72177700	3.02223000	-0.73142400
C	-13.04687100	2.60903300	1.83263200
H	-13.05756300	3.64711500	2.16434700
H	-14.07584600	2.32765000	1.58324200
H	-12.74682800	1.98546900	2.68209000
C	-10.98465100	4.60199900	0.69521500
H	-11.57993200	4.96788500	1.52610100
C	-9.96718700	5.34701500	0.22644300
H	-9.36365100	4.93598300	-0.58025600
C	-9.55237100	6.65528900	0.73132000
C	-8.50378900	7.25970600	0.14759500
H	-8.01044700	6.74645100	-0.67656800
C	-10.33632500	7.23938300	1.87666300
H	-11.38016500	7.39410500	1.58572000
H	-10.34063700	6.55151000	2.72805300
H	-9.94148100	8.19595400	2.21617800
C	-7.87355800	8.57491600	0.48287300

H	-8.29264900	9.01123600	1.39897800
H	-8.05524200	9.29503700	-0.32320100
O	-6.45924700	8.46391400	0.56940900
H	-6.26731400	7.76236800	1.20326700
C	-7.01095300	1.12713300	1.00621200
H	-7.58835600	0.32200200	0.54455900
C	-6.94695100	2.26869900	-0.00695000
H	-6.42569900	3.11967800	0.45617700
C	-6.16590400	1.86351400	-1.25393300
H	-6.72058600	1.05241300	-1.75278700
C	-4.95283600	0.25755700	-0.03384300
H	-5.53402800	-0.52755900	-0.53764300
C	-5.62032100	0.58225300	1.29494100
H	-5.69257800	-0.35328400	1.86778500
C	-5.97078600	3.01482300	-2.21457700
H	-5.39302500	3.80711000	-1.73122500
H	-6.94163400	3.41603500	-2.51089300
H	-5.42899100	2.68437700	-3.10334400
C	-2.92978300	-0.73591300	-0.83992000
H	-3.06512500	-0.09899100	-1.72111300
C	-1.49636300	-0.71060800	-0.41262000
H	-1.25162600	-1.26305100	0.49076400
C	-0.53870100	-0.03926200	-1.05917900
H	-0.78282900	0.51774100	-1.96332400
C	0.83321700	0.01008700	-0.59529500
H	1.05561000	-0.54464300	0.31282800

C	1.82508700	0.70194700	-1.18721000
H	1.62011900	1.26864200	-2.09508700
C	3.16771200	0.73712100	-0.65848900
H	3.33662300	0.14896900	0.24137400
C	4.19984200	1.41791900	-1.19568300
H	4.04806600	2.00863100	-2.09851600
C	5.52288400	1.40401000	-0.62238600
H	5.66711000	0.78657800	0.26416400
C	6.58294100	2.07952300	-1.11116600
H	6.46147100	2.68978200	-2.00545800
C	7.88804300	2.05567500	-0.49956800
H	7.99762700	1.45388800	0.40255900
C	8.96507500	2.71806700	-0.96762700
H	8.87295700	3.30631100	-1.87998800
C	10.25402000	2.69923400	-0.31964200
H	10.31826000	2.15908800	0.62343800
C	11.36864800	3.27877100	-0.80166800
H	11.33176600	3.80227600	-1.75635300
C	-3.46942100	-2.14170300	-1.14681000
H	-4.50771500	-2.05029100	-1.47378300
H	-2.90455800	-2.56980900	-1.98017700
C	12.64909100	3.20398600	-0.12569500
H	12.63083000	2.76374200	0.86934600
C	13.82034300	3.57466500	-0.65424700
H	13.83939300	3.99948400	-1.65929500
C	15.15425600	3.40577900	0.01525100

H	14.99461800	3.10834400	1.05796800
C	15.97444900	2.28683100	-0.65638500
H	16.26086600	2.64792300	-1.65817100
C	15.21645900	0.96314600	-0.86859800
H	14.34491600	1.21387200	-1.48116100
C	14.64586400	0.37879600	0.42826700
H	14.15771100	1.16224200	1.00129900
C	12.39989100	-0.45583600	0.56754800
C	11.43315600	-1.48327100	0.03460400
H	11.75875700	-1.80914400	-0.95502400
H	11.47869100	-2.35063600	0.70351000
C	9.99478200	-0.96146600	-0.00146000
H	9.94526200	-0.08731100	-0.66635900
C	9.03571100	-2.03114000	-0.50385000
H	9.33754900	-2.32616900	-1.51453200
H	9.12024100	-2.91499000	0.13976300
C	7.57295100	-1.59466000	-0.53332100
H	7.48099500	-0.65966000	-1.10269600
C	6.69068300	-2.65589200	-1.18247700
H	6.93519600	-3.63321100	-0.74827700
H	6.96327600	-2.70684800	-2.24101700
C	5.18503000	-2.38166600	-1.03448800
H	5.00954000	-1.32252400	-0.83179200
H	4.67103500	-2.59889100	-1.97593100
C	4.50668700	-3.22332800	0.04483600
H	4.54781500	-4.27732600	-0.26362300

C	3.02001700	-2.82363300	0.22899600
H	2.65255600	-2.33313500	-0.67965000
C	2.11968000	-4.00953000	0.55676500
H	2.46954600	-4.48219300	1.48254400
H	2.20519900	-4.75062400	-0.24636900
C	0.65425400	-3.60962300	0.70265000
H	0.33384700	-3.09109400	-0.21137800
C	-0.24895900	-4.81848400	0.90694200
H	0.08080200	-5.38157400	1.78558800
H	-0.16380200	-5.46894100	0.03162100
C	-1.72026900	-4.45546600	1.10918900
C	-2.62737500	-5.67674700	1.08592000
H	-2.41122400	-6.32490500	1.93766300
H	-2.44597200	-6.24090900	0.16680700
C	-4.09538300	-5.28917200	1.11728100
H	-4.31796300	-4.82699200	2.09020700
C	-4.39870600	-4.24210200	0.00892000
H	-4.34756700	-4.77286700	-0.94888900
C	-3.37531000	-3.09445200	0.03955800
H	-3.51959100	-2.53375000	0.96788400
C	-5.80082900	-3.72233600	0.19384500
C	15.61281100	-0.37361200	1.31936900
H	15.10953300	-0.68767200	2.23668200
H	16.44647700	0.28292600	1.57225400
H	16.00089900	-1.26270700	0.81771100
C	16.06148100	-0.04482500	-1.64564900

H	16.98919100	-0.29116900	-1.12037200
H	16.32540900	0.35006400	-2.63099200
H	15.51212700	-0.97738200	-1.79280500
C	15.94995800	4.71412800	0.01247800
H	16.10856500	5.06738800	-1.01251100
H	16.92545200	4.56710300	0.47800800
H	15.41090200	5.49430900	0.55462600
O	-4.89531700	1.56462500	2.00592100
H	-3.95817200	1.37410200	1.86218200
O	-8.26312300	2.62388400	-0.38791700
H	-8.75694000	2.67446900	0.44825200
O	-4.87885900	1.39326600	-0.86957900
O	-3.66138500	-0.16134800	0.25601600
O	-1.93410000	-3.79514500	2.32231700
H	-1.19610900	-3.16751400	2.42892000
O	-2.05955800	-3.62745000	0.00123500
O	-6.11736600	-2.59501900	0.49879300
O	-6.73691700	-4.68697000	0.02760800
H	-7.60172800	-4.28233400	0.21140600
O	0.47132900	-2.73039700	1.81535800
H	1.28335000	-2.19749700	1.90163200
O	2.94986600	-1.84963600	1.27200200
H	3.72866200	-2.07200400	1.82441300
O	5.12780900	-3.12111000	1.31802700
H	5.89136800	-2.50722500	1.25723800
O	7.08258000	-1.36273100	0.79025000

H	7.80900500	-0.97112700	1.30900600
O	9.56882100	-0.58226900	1.30467800
H	10.27436900	-0.03011500	1.68664400
O	12.09994700	0.35873100	1.42623600
O	13.59961700	-0.55557600	0.01465100
O	17.14223100	2.11388000	0.14094800
H	17.80741700	1.65233100	-0.38074000
O	-4.82140500	-6.48527900	0.93627200
H	-5.75681600	-6.26423800	0.84534700
N	-7.75687200	1.60101500	2.16607800
H	-7.13419300	2.16397400	2.74105800
H	-8.06157000	0.81893500	2.73698900

AmB-Dod

AmB-Dod1

0 1

C	4.66938100	0.24751100	-3.61237800
H	4.89758000	-0.74195800	-3.19970900
H	5.36887300	0.95979300	-3.16501400
H	4.86736500	0.20668900	-4.68812100
C	3.22281400	0.63249400	-3.32173400
H	3.01795500	1.63484800	-3.71882300
H	3.08351000	0.70579800	-2.23975900

C	2.20502700	-0.35631100	-3.88095700
H	2.46792600	-1.36521300	-3.53299800
H	2.27297700	-0.39205400	-4.97618600
C	0.77133300	-0.05295300	-3.45017300
H	0.45713300	0.91937900	-3.85017000
H	0.74968100	0.06012000	-2.36121300
C	-0.22521500	-1.13545400	-3.86683700
H	0.25712200	-2.11920700	-3.78159600
H	-0.48282600	-1.02402700	-4.92763000
C	-1.49420200	-1.15991800	-3.01864900
H	-1.95925300	-0.16709800	-2.99241700
H	-1.21113500	-1.37497800	-1.98065500
C	-2.50782100	-2.20139200	-3.48645400
H	-1.97620700	-3.11859300	-3.77459100
H	-3.00568600	-1.84652100	-4.39799800
C	-3.55048300	-2.55723500	-2.43087600
H	-4.02583800	-1.64803100	-2.04257700
H	-3.03895400	-3.00946100	-1.57002100
C	-4.61457400	-3.52267600	-2.94599900
H	-4.12388400	-4.36873400	-3.44661900
H	-5.21316300	-3.02293600	-3.71896900
C	-5.53588400	-4.04809000	-1.85081100
H	-5.99098600	-3.20810900	-1.31865700
H	-4.93238900	-4.58502500	-1.10519500
C	-6.62569700	-4.98232400	-2.36587600
H	-6.17306100	-5.86286500	-2.83878300

H	-7.21620700	-4.47543000	-3.13836200
C	-7.57645300	-5.43829000	-1.27126300
H	-8.25123500	-6.21651100	-1.65773800
H	-6.99858100	-5.88932400	-0.44987700
O	-8.31590500	-4.32065200	-0.82036700
H	-8.53461300	-4.40527700	0.12390700
C	-9.09290500	4.62270000	0.92127000
H	-9.95814600	4.07391900	0.52856700
C	-8.58056800	5.51698800	-0.20678700
H	-7.72096800	6.09184400	0.17162100
C	-8.08608800	4.66838400	-1.37195800
H	-8.93467900	4.06760800	-1.74153700
C	-7.48632900	2.90467100	0.09026300
H	-8.27276700	2.25261100	-0.32048800
C	-8.02223300	3.61590500	1.33004000
H	-8.45022200	2.84584500	1.98897400
C	-7.51443200	5.49734000	-2.49951300
H	-6.65233700	6.06491500	-2.13917200
H	-8.27005900	6.19615300	-2.86181000
H	-7.18789100	4.85476200	-3.32020600
C	-5.75489800	1.33112100	-0.46368900
H	-5.73050600	1.86384900	-1.41961000
C	-4.35995600	1.12282500	0.03662200
H	-4.25877800	0.61488600	0.99159900
C	-3.26420400	1.55901700	-0.59199700
H	-3.35407700	2.07947400	-1.54503200

C	-1.93569000	1.40549700	-0.03414000
H	-1.86960200	0.86752100	0.90869300
C	-0.80850700	1.90081800	-0.57786400
H	-0.85984300	2.45025100	-1.51735700
C	0.48758200	1.73947000	0.03610700
H	0.51405700	1.14795300	0.94931600
C	1.63863800	2.23966100	-0.45582000
H	1.62010800	2.82854500	-1.37215000
C	2.92459400	2.00889100	0.15378700
H	2.94820400	1.37410200	1.03891700
C	4.08886700	2.48265000	-0.33549700
H	4.07296900	3.09921400	-1.23365100
C	5.37579500	2.21557600	0.25385300
H	5.39170900	1.59002900	1.14574500
C	6.54472600	2.67648100	-0.23599600
H	6.53818500	3.28686300	-1.13852500
C	7.82436800	2.41193400	0.37462400
H	7.81451700	1.84140100	1.30145600
C	9.01224600	2.79937400	-0.12489200
H	9.04662200	3.34933000	-1.06466800
C	-6.57313000	0.04440600	-0.61141400
H	-7.58224700	0.31498300	-0.92748000
H	-6.14200300	-0.57943800	-1.40007900
C	10.27406200	2.47574700	0.51131100
H	10.20042500	2.00980500	1.49171100
C	11.48068100	2.64980700	-0.03837900

H	11.55469500	3.10115000	-1.02918800
C	12.77450400	2.21405900	0.58781200
H	12.58160200	1.90043600	1.62022400
C	13.36604300	0.99888700	-0.15299400
H	13.69577400	1.34939100	-1.14527100
C	12.37648400	-0.15389300	-0.40420300
H	11.55202700	0.28173000	-0.97681300
C	11.73619100	-0.69344200	0.87963800
H	11.41332300	0.13316300	1.50668000
C	9.37616100	-1.10154800	1.04098000
C	8.22735500	-1.91185000	0.49516400
H	8.46281200	-2.23671400	-0.52008100
H	8.13934100	-2.80870600	1.11958000
C	6.90154300	-1.14776800	0.53558800
H	6.98169100	-0.25302500	-0.09800000
C	5.75618600	-2.01808200	0.03924200
H	5.97014900	-2.32586900	-0.99003600
H	5.70917100	-2.92448700	0.65481900
C	4.38910400	-1.33979600	0.07170100
H	4.43645500	-0.39819600	-0.49283400
C	3.31919000	-2.23392400	-0.54559800
H	3.40485700	-3.23843300	-0.11327400
H	3.54839400	-2.33003300	-1.61141300
C	1.88883500	-1.70533300	-0.36281400
H	1.90440600	-0.62684000	-0.18305600
H	1.32158500	-1.85194600	-1.28625700

C	1.09549100	-2.39038000	0.74841000
H	0.92966700	-3.43737100	0.45897500
C	-0.28498500	-1.70920800	0.94925800
H	-0.55092800	-1.14622800	0.04863000
C	-1.40500900	-2.69388000	1.26864900
H	-1.16406300	-3.22457900	2.19788400
H	-1.45968200	-3.43927700	0.46656600
C	-2.76573200	-2.01335800	1.39291700
H	-2.98584300	-1.48903800	0.45389400
C	-3.88405300	-3.01501300	1.65479800
H	-3.66313300	-3.58458000	2.56289200
H	-3.93258400	-3.71243300	0.81439100
C	-5.25590500	-2.36389900	1.83160500
C	-6.38592300	-3.38101200	1.89597400
H	-6.27567600	-4.01758300	2.77640600
H	-6.35041500	-4.00441600	1.00012900
C	-7.73649700	-2.69020600	1.94495500
H	-7.82953700	-2.16471700	2.90349600
C	-7.87302100	-1.66292500	0.79122100
H	-8.00195500	-2.24432700	-0.12988900
C	-6.62618500	-0.77021200	0.67577700
H	-6.60413700	-0.09457300	1.53612800
C	-9.10558400	-0.81805500	0.98865200
C	12.56652300	-1.65864300	1.70054900
H	12.03064500	-1.93084800	2.61276600
H	13.50940600	-1.17897700	1.96675900

H	12.77861700	-2.57187200	1.14048900
C	13.00596600	-1.25487900	-1.25571300
H	13.88673700	-1.69185200	-0.77551800
H	13.31248900	-0.86193900	-2.22940600
H	12.29303600	-2.06416800	-1.42867500
C	13.79335200	3.35657300	0.62082700
H	13.99372900	3.72443800	-0.39161600
H	14.73481200	3.01463900	1.05292400
H	13.41442900	4.19393400	1.21111500
O	-6.99053300	4.32612100	1.98364200
H	-6.18718700	3.79771700	1.87282600
O	-9.61610100	6.36286900	-0.65097700
H	-10.05874100	6.64233100	0.16684300
O	-7.05276600	3.80905700	-0.90159600
O	-6.38048400	2.17765800	0.51804600
O	-5.32315800	-1.59153500	2.99459100
H	-4.46820600	-1.12850200	3.07095100
O	-5.44985100	-1.56848400	0.66682400
O	-9.16896500	0.38743700	1.02037900
O	-10.22505200	-1.57006000	1.15746600
H	-10.95809500	-0.94873300	1.29594100
O	-2.76904800	-1.05795900	2.45832300
H	-1.85537700	-0.73448000	2.57131400
O	-0.15655100	-0.74904200	1.99883300
H	0.57808800	-1.11379500	2.53636300
O	1.75318300	-2.38916300	2.00663600

H	2.61939700	-1.93536100	1.91855800
O	3.99353400	-1.03927400	1.41309800
H	4.79659300	-0.80481800	1.91353200
O	6.59253900	-0.75360300	1.87001500
H	7.39791400	-0.35291200	2.24354700
O	9.24876500	-0.28446600	1.93932800
O	10.52648300	-1.39679000	0.45367100
O	14.49665100	0.58036000	0.60560200
H	15.05649000	0.02930400	0.04829800
O	-8.71684700	-3.72298900	1.86082200
H	-9.58599400	-3.29708300	1.84931900
N	-9.56186100	5.49177900	1.99719800
H	-8.74795300	5.81722400	2.51439000
H	-10.13697900	4.97216900	2.65264400

AmB-Dod2

0 1

C	13.42888900	1.29187500	2.64365800
H	13.90765600	0.43509200	3.12925300
H	14.03654100	2.17615000	2.85649900
H	13.44535000	1.11886000	1.56336200
C	11.99476000	1.46584900	3.13352000
H	11.54819100	2.34241000	2.64858000
H	11.98491200	1.67903300	4.20943700

C	11.12851400	0.24093900	2.85339800
H	11.52901400	-0.62102400	3.40316200
H	11.21387500	-0.01780300	1.79027000
C	9.65702400	0.43344000	3.20676200
H	9.25479100	1.27567800	2.62659300
H	9.56541600	0.72489900	4.26116100
C	8.80988500	-0.80703700	2.94006100
H	9.17874700	-1.64070500	3.55205000
H	8.93923900	-1.10827800	1.89424000
C	7.32148300	-0.59867900	3.19771400
H	6.98089600	0.26582700	2.61417200
H	7.15701700	-0.34350900	4.25271200
C	6.47703000	-1.81176100	2.82038800
H	6.80078600	-2.68543500	3.40072000
H	6.66963500	-2.05615200	1.76861600
C	4.97829000	-1.59849200	3.01426000
H	4.67685400	-0.66562700	2.51891900
H	4.75972700	-1.45605300	4.08049300
C	4.13704600	-2.74111200	2.45503400
H	4.42853800	-3.68593800	2.93145700
H	4.35904300	-2.84419400	1.38708400
C	2.63622200	-2.52982400	2.62714200
H	2.37448200	-1.53635200	2.23811700
H	2.38604200	-2.52115500	3.69756900
C	1.80216500	-3.58155700	1.90071400
H	2.12690900	-4.58566500	2.20046100

H	1.97750600	-3.49865100	0.82402300
C	0.31025400	-3.46906000	2.17864000
H	-0.24601700	-4.14090800	1.52420300
H	0.09353100	-3.73783700	3.22194100
O	-0.20818500	-2.16900300	1.90350600
H	0.18479600	-1.52874800	2.50590200
C	-10.98699700	3.34238300	1.79677800
H	-11.85535200	2.88017200	1.31054300
C	-10.66738700	4.62044700	1.02308500
H	-9.80804600	5.11076900	1.50640900
C	-10.25913700	4.28616700	-0.40672200
H	-11.10624700	3.77105800	-0.89145200
C	-9.38713100	2.19189000	0.26977600
H	-10.17794400	1.65384100	-0.27516600
C	-9.81494000	2.36968700	1.72400400
H	-10.11344700	1.38006000	2.10001600
C	-9.87639700	5.50997700	-1.20750700
H	-9.01447100	5.99811600	-0.74494200
H	-10.71121700	6.21228300	-1.22621300
H	-9.60987000	5.23164100	-2.22953700
C	-7.65345300	1.06924200	-0.96052000
H	-7.77220300	1.89739600	-1.66665700
C	-6.19872800	0.82499200	-0.70955500
H	-5.95236800	0.01028600	-0.03393700
C	-5.21762800	1.56249900	-1.23916700
H	-5.45828600	2.38630700	-1.91043500

C	-3.82067700	1.33012500	-0.93282500
H	-3.60819800	0.48861200	-0.27861000
C	-2.78798000	2.07611500	-1.36894700
H	-2.97046500	2.93003200	-2.02062000
C	-1.42703900	1.78643000	-0.98517400
H	-1.29745000	0.90607900	-0.35970900
C	-0.33419400	2.48724400	-1.34695400
H	-0.43268200	3.36861600	-1.97966000
C	0.99109000	2.11343700	-0.91819400
H	1.07042300	1.20775000	-0.31594800
C	2.12183100	2.78600000	-1.21491400
H	2.06360300	3.68442800	-1.82823200
C	3.42541100	2.38983700	-0.74474200
H	3.47329300	1.49721400	-0.12076000
C	4.57247100	3.04079200	-1.02547600
H	4.54133800	3.92138800	-1.66608500
C	5.86114700	2.63415400	-0.52044500
H	5.87085400	1.79269900	0.16975800
C	7.03599300	3.19936300	-0.85355600
H	7.05177900	4.02726300	-1.56153500
C	-8.41731200	-0.15695900	-1.47584200
H	-9.45980600	0.13108600	-1.62941200
H	-8.01463100	-0.45692500	-2.44794400
C	8.30894600	2.72074800	-0.35122200
H	8.25475000	1.95571200	0.42009200
C	9.50804800	3.09754400	-0.80773300

H	9.56743800	3.85030200	-1.59559000
C	10.81283200	2.51626100	-0.34432000
H	10.62413300	1.87816400	0.52547100
C	11.44637300	1.62638300	-1.43121700
H	11.78228000	2.29062200	-2.24433600
C	10.49530200	0.59786600	-2.07128700
H	9.65084400	1.17562100	-2.45886700
C	9.88933200	-0.37894300	-1.05655500
H	9.56228700	0.16390500	-0.17299600
C	7.52996600	-0.83610900	-1.05705800
C	6.40216500	-1.49343700	-1.81043400
H	6.62720700	-1.48771800	-2.87874400
H	6.36877400	-2.54043200	-1.48597400
C	5.04546600	-0.84262900	-1.52941000
H	5.07391100	0.20591200	-1.85831500
C	3.92652900	-1.57131400	-2.25826600
H	4.12502900	-1.52529000	-3.33450900
H	3.93454900	-2.62777800	-1.96416400
C	2.53212400	-1.01030500	-1.98709500
H	2.53892600	0.07616600	-2.15312100
C	1.49485800	-1.64018000	-2.91076400
H	1.61521400	-2.72876200	-2.87856100
H	1.72679500	-1.32034600	-3.93124100
C	0.04980300	-1.26812000	-2.54382000
H	0.04234700	-0.40507200	-1.87443800
H	-0.50894200	-0.96633300	-3.43480100

C	-0.71465500	-2.41418300	-1.87908500
H	-0.93185800	-3.17738900	-2.63886400
C	-2.04839800	-1.93156200	-1.29123200
H	-2.49298900	-1.19708500	-1.97434500
C	-3.05255600	-3.05896700	-1.06782000
H	-2.64475900	-3.76776900	-0.33771200
H	-3.19634100	-3.60045500	-2.00975800
C	-4.40418300	-2.53332900	-0.59261600
H	-4.76209800	-1.78336700	-1.31121500
C	-5.45570800	-3.62962700	-0.49252500
H	-5.11608400	-4.40650300	0.19939800
H	-5.58488700	-4.08189400	-1.48000700
C	-6.81234300	-3.12035200	-0.00209800
C	-7.90702000	-4.17155800	-0.11658600
H	-7.70209200	-5.00585300	0.55748500
H	-7.93232600	-4.55289700	-1.14127200
C	-9.27692900	-3.59831900	0.20468000
H	-9.30289100	-3.32068400	1.26868900
C	-9.52902600	-2.31343100	-0.63159000
H	-9.67162000	-2.63469500	-1.67004900
C	-8.33693100	-1.35075500	-0.53014900
H	-8.27776900	-0.99212100	0.50171600
C	-10.79571400	-1.64939000	-0.15798400
C	10.75389000	-1.55515900	-0.64940300
H	10.25636100	-2.13753500	0.12945400
H	11.70438200	-1.18541600	-0.26387500

H	10.94584900	-2.21228400	-1.50016700
C	11.15719000	-0.11542300	-3.24920700
H	12.05979800	-0.65440900	-2.94562900
H	11.43741600	0.60132600	-4.02631300
H	10.47518800	-0.84404000	-3.69329600
C	11.79316600	3.61530700	0.07469600
H	11.98996500	4.29574600	-0.76119400
H	12.74182500	3.18414100	0.39673100
H	11.37852900	4.20641800	0.89454500
O	-8.75922100	2.90785400	2.49349300
H	-7.94379300	2.52933100	2.13488100
O	-11.79941100	5.45937700	1.00135900
H	-12.16094300	5.39009600	1.89988800
O	-9.12684200	3.42390700	-0.36737300
O	-8.19931400	1.46992200	0.30903200
O	-6.76358400	-2.70620800	1.33202900
H	-5.92005500	-2.22978900	1.44268200
O	-7.14128100	-2.03782800	-0.86870700
O	-10.89364400	-0.57600600	0.38757100
O	-11.89105800	-2.42223200	-0.36986200
H	-12.64577000	-1.93639100	0.00116200
O	-4.28963400	-1.91772700	0.69303200
H	-3.41160200	-1.49117200	0.72442900
O	-1.81791200	-1.22373400	-0.07346000
H	-1.18539000	-1.72571900	0.48578200
O	0.03872600	-3.05404200	-0.86491500

H	0.70217200	-2.42001800	-0.53513300
O	2.13474200	-1.25932300	-0.63514700
H	2.91311900	-1.10388200	-0.07409600
O	4.74874500	-0.88267400	-0.13506200
H	5.53694400	-0.55766700	0.33443600
O	7.38027600	-0.30798800	0.03387700
O	8.68893400	-0.92756700	-1.69032000
O	12.57872500	1.00363600	-0.82778200
H	13.17091700	0.69093300	-1.51997700
O	-10.20864200	-4.61774700	-0.08489500
H	-11.09743400	-4.24958800	-0.00279000
N	-11.38174300	3.73001600	3.14848700
H	-10.53552900	3.93560500	3.67530800
H	-11.84122600	2.95868600	3.62212700

AmB-Dod3

0 1

C	9.20164400	1.32075800	-2.53807500
H	9.31053900	1.64732600	-3.57703400
H	10.04061500	0.65837300	-2.30965400
H	9.28927500	2.20515800	-1.89912800
C	7.86305500	0.62340500	-2.31980400
H	7.75899900	0.36828900	-1.25847800
H	7.84724600	-0.32403600	-2.87430500

C	6.67200800	1.47528900	-2.75225400
H	6.81010600	1.78183600	-3.79753000
H	6.65826700	2.40035400	-2.16163600
C	5.33056200	0.76405100	-2.60702000
H	5.17988700	0.50097800	-1.55487200
H	5.36028600	-0.18157500	-3.16653400
C	4.14007100	1.59185200	-3.08378600
H	4.30163600	1.89907500	-4.12544600
H	4.08888400	2.51836500	-2.49728300
C	2.81107100	0.85003900	-2.97220800
H	2.64402700	0.56440500	-1.92822100
H	2.87168200	-0.08511600	-3.54598000
C	1.61131000	1.65569300	-3.46019200
H	1.76982200	1.95249300	-4.50545600
H	1.54467700	2.58970000	-2.88650300
C	0.29298700	0.89568500	-3.33988200
H	0.11276900	0.65130000	-2.28699600
H	0.38008600	-0.06265800	-3.87068900
C	-0.90159300	1.66401700	-3.89498900
H	-0.71517400	1.91034300	-4.94877100
H	-0.99330200	2.62561100	-3.37243000
C	-2.22040100	0.90349200	-3.78227500
H	-2.44883600	0.74810000	-2.72023800
H	-2.10198300	-0.09568300	-4.22399700
C	-3.37720800	1.62057600	-4.47408300
H	-3.13150600	1.75854200	-5.53472500

H	-3.50690300	2.62134700	-4.04551800
C	-4.70645300	0.88492900	-4.37663200
H	-5.45717200	1.38770200	-4.99257200
H	-4.59783200	-0.13886800	-4.76748600
O	-5.23754300	0.87399200	-3.06356300
H	-4.66832700	0.35279400	-2.48406500
C	-10.18474200	2.90538300	2.30165900
H	-10.98843300	2.53928100	1.65044800
C	-9.72508900	4.24764600	1.73475700
H	-8.92935600	4.64255300	2.38543700
C	-9.12901800	4.05941900	0.34477400
H	-9.91339100	3.64053700	-0.30872500
C	-8.41791300	1.86658200	0.88283100
H	-9.13961200	1.42706900	0.17678600
C	-9.04106300	1.89697100	2.27565600
H	-9.41645900	0.88496600	2.48758200
C	-8.60247000	5.34743200	-0.24630900
H	-7.80152000	5.74158400	0.38481800
H	-9.40601200	6.08384400	-0.29737600
H	-8.20110800	5.17298300	-1.24697700
C	-6.58100500	0.79433600	-0.23434700
H	-6.57108800	1.68401100	-0.86914100
C	-5.18251100	0.45601200	0.18456900
H	-5.06975600	-0.39374800	0.85284300
C	-4.10161600	1.15688900	-0.18000900
H	-4.21300500	2.00761900	-0.85042300

C	-2.76845500	0.84226600	0.29396900
H	-2.68112700	-0.00668400	0.96750400
C	-1.65609600	1.53268500	-0.02305300
H	-1.72667100	2.38778600	-0.69522100
C	-0.35526800	1.19499300	0.50319700
H	-0.31854200	0.32958500	1.16182000
C	0.78624800	1.85446300	0.22126300
H	0.75948700	2.71697900	-0.44396500
C	2.06914400	1.47068800	0.75503300
H	2.09592100	0.59063100	1.39778500
C	3.22472000	2.11405200	0.49132100
H	3.20849300	2.98540000	-0.16235900
C	4.50121900	1.72359500	1.03250400
H	4.51514700	0.85978400	1.69742700
C	5.66250800	2.35375600	0.76276800
H	5.65792300	3.20630200	0.08462000
C	6.92886500	1.96651600	1.33233300
H	6.91264700	1.14784900	2.04972700
C	8.11442600	2.53049600	1.03567600
H	8.16005900	3.33609900	0.30357500
C	-7.31720900	-0.33543000	-0.96212400
H	-8.32415400	0.01826600	-1.19424700
H	-6.82152900	-0.53188600	-1.91483400
C	9.35946600	2.08354300	1.62819400
H	9.26169700	1.34363900	2.41973100
C	10.58087300	2.46067600	1.23631900

H	10.68126500	3.18758700	0.42860600
C	11.86163400	1.90100000	1.78651900
H	11.63565200	1.31174400	2.68273000
C	12.52356200	0.94929600	0.77029500
H	12.86732000	1.56604400	-0.07693400
C	11.58486200	-0.11738000	0.18013200
H	10.75097000	0.43559200	-0.25877200
C	10.94955300	-1.02775800	1.23674300
H	10.56816000	-0.42912000	2.06017200
C	8.60376100	-1.55333700	1.16288100
C	7.52784300	-2.23650000	0.35784500
H	7.81479800	-2.23910000	-0.69547000
H	7.47793800	-3.27867300	0.69389200
C	6.15887700	-1.58313900	0.54868300
H	6.21863000	-0.53541700	0.22547100
C	5.09308100	-2.29618700	-0.26793100
H	5.37666600	-2.24707900	-1.32438700
H	5.06301400	-3.35290500	0.02305500
C	3.69446800	-1.70813000	-0.10973400
H	3.73211500	-0.62853500	-0.30113500
C	2.71332000	-2.34237500	-1.08649300
H	2.79437500	-3.43366500	-1.01048800
H	3.03681800	-2.07122800	-2.09529100
C	1.25718400	-1.90527100	-0.86663400
H	1.22249300	-0.96455000	-0.31103800
H	0.77977800	-1.70285000	-1.82984300

C	0.39737500	-2.94257900	-0.15052800
H	0.31101900	-3.82670500	-0.79755700
C	-1.02654000	-2.39884500	0.13186600
H	-1.25723600	-1.58669700	-0.56751300
C	-2.10563500	-3.47073300	0.02837700
H	-1.88731000	-4.27125100	0.74557000
H	-2.07264600	-3.90874200	-0.97592600
C	-3.50696300	-2.92213400	0.28041300
H	-3.69577500	-2.09256100	-0.41514800
C	-4.58111700	-3.98050100	0.06356400
H	-4.37758000	-4.84799500	0.69898900
H	-4.54456100	-4.30507600	-0.98045000
C	-5.99504800	-3.49158000	0.38018700
C	-7.06542200	-4.48516300	-0.04733300
H	-6.97433500	-5.41081400	0.52486100
H	-6.93550600	-4.71879800	-1.10783600
C	-8.46135400	-3.91789200	0.14407800
H	-8.64328800	-3.78747100	1.22071100
C	-8.56977700	-2.52601300	-0.53521500
H	-8.57375800	-2.70317400	-1.61727200
C	-7.39083400	-1.62034700	-0.15030600
H	-7.48111100	-1.37810500	0.91260400
C	-9.87734800	-1.88033300	-0.15369800
C	11.81289800	-2.14860800	1.77834400
H	11.27202200	-2.69854700	2.55197300
H	12.72072300	-1.72271000	2.20752200

H	12.08882900	-2.84974800	0.98775700
C	12.26353700	-0.90236200	-0.94070100
H	13.16296700	-1.42037700	-0.59392500
H	12.55310500	-0.23476300	-1.75760000
H	11.58728000	-1.65772600	-1.34747800
C	12.83679100	3.01339900	2.18022600
H	13.06871200	3.64653700	1.31655700
H	13.76987100	2.58785200	2.55171700
H	12.40333200	3.65055300	2.95444700
O	-8.09006300	2.29777700	3.24122400
H	-7.24197300	1.93229300	2.95183800
O	-10.81990200	5.13189500	1.65846800
H	-11.30575400	4.97644400	2.48464500
O	-8.03818100	3.14962100	0.43677700
O	-7.26705400	1.09535900	0.99560100
O	-6.16834200	-3.25458100	1.74730800
H	-5.35727800	-2.81194900	2.05638800
O	-6.16276800	-2.30019600	-0.38101700
O	-10.02928800	-0.87245800	0.49449400
O	-10.94775700	-2.58992900	-0.59354100
H	-11.73658900	-2.12548300	-0.26919900
O	-3.62946800	-2.43149700	1.61739300
H	-2.75283300	-2.10245400	1.88995400
O	-1.02618000	-1.82144200	1.43869700
H	-0.32130300	-2.32802700	1.89528500
O	0.93471600	-3.36440500	1.09456400

H	1.79514600	-2.91845300	1.24840200
O	3.19479400	-1.90829600	1.21506700
H	3.94215300	-1.80085400	1.83050800
O	5.76088100	-1.62758800	1.91656200
H	6.50965200	-1.29101900	2.43922600
O	8.39182800	-1.00498300	2.23278900
O	9.79222300	-1.63729800	0.58287700
O	13.64728500	0.37509700	1.43006000
H	14.23807100	0.00287000	0.76651600
O	-9.35101500	-4.85813100	-0.41843800
H	-10.23833100	-4.47741600	-0.40864500
N	-10.75445700	3.15417100	3.62348200
H	-9.98451600	3.26162300	4.28044000
H	-11.29534600	2.35388700	3.93565800

AmB-Dod4

0 1

C	-0.14024600	-0.60891000	-2.79545000
H	0.14960000	0.32365000	-3.28902700
H	0.60393800	-1.36621800	-3.05893700
H	-0.09232600	-0.44156900	-1.71578000
C	-1.54168900	-1.04201900	-3.20908400
H	-1.81050700	-1.96209700	-2.67526500
H	-1.55458300	-1.29456600	-4.27619300

C	-2.59261600	0.02962000	-2.93487600
H	-2.32756300	0.94641500	-3.47720500
H	-2.56550200	0.29653900	-1.86974900
C	-4.00885100	-0.39570100	-3.30914800
H	-4.25794900	-1.31926000	-2.77252200
H	-4.04894300	-0.64148800	-4.37805800
C	-5.05401400	0.66952000	-2.99089200
H	-4.85034500	1.57370800	-3.57863500
H	-4.94482000	0.97450800	-1.94037700
C	-6.49027000	0.21244200	-3.22890500
H	-6.68272100	-0.69268200	-2.64165600
H	-6.61756400	-0.07142600	-4.28148300
C	-7.52151800	1.27301600	-2.85456200
H	-7.44149300	2.12231000	-3.54558000
H	-7.28194300	1.66943200	-1.85957200
C	-8.95471300	0.75026600	-2.84251700
H	-9.03596500	-0.01936800	-2.06496800
H	-9.17479400	0.24836300	-3.79387500
C	-9.98993600	1.84539200	-2.60152200
H	-9.95693600	2.56074700	-3.43338900
H	-9.71340700	2.42058400	-1.70710700
C	-11.41596200	1.32429500	-2.44282500
H	-11.47407000	0.69699500	-1.54393900
H	-11.66100400	0.66377400	-3.28468600
C	-12.45317500	2.44433800	-2.36909500
H	-12.47245600	2.97010500	-3.33165200

H	-12.15817200	3.18283500	-1.61411300
C	-13.86245900	1.96486600	-2.03642200
H	-14.58384800	2.75836900	-2.25303700
H	-14.12086800	1.11135900	-2.68529900
O	-14.04662000	1.64238500	-0.67307000
H	-13.35749100	1.03112000	-0.37164800
C	11.21311100	3.65894400	-1.01941900
H	12.07157300	3.14832300	-0.56529400
C	10.79647000	4.77434900	-0.06164100
H	9.94519400	5.31166000	-0.50774300
C	10.32475100	4.18688700	1.26326300
H	11.16636400	3.62733200	1.70639500
C	9.58610300	2.21061200	0.18992400
H	10.37240000	1.61631900	0.68046400
C	10.08048700	2.65134200	-1.18461300
H	10.43965000	1.75198100	-1.70625500
C	9.84198800	5.24216100	2.23220900
H	8.98549000	5.76999000	1.80453000
H	10.64082600	5.96105400	2.42095700
H	9.53200800	4.78468100	3.17437600
C	7.85533900	0.82061900	1.10925500
H	7.90157200	1.51300000	1.95613700
C	6.42605000	0.56836400	0.74421800
H	6.25062800	-0.06220200	-0.12348500
C	5.38914000	1.08892900	1.40757800
H	5.56643200	1.72184500	2.27673000

C	4.01149900	0.87593900	1.01130200
H	3.84934300	0.25500100	0.13386900
C	2.94207800	1.40508900	1.63532100
H	3.08884400	2.03000200	2.51594600
C	1.58818600	1.19130200	1.18262900
H	1.47636400	0.56205200	0.30202300
C	0.48588500	1.69256000	1.77551900
H	0.58796700	2.31507400	2.66392400
C	-0.84908900	1.44366700	1.29077800
H	-0.94162600	0.81295200	0.40671700
C	-1.97462000	1.91873000	1.86281800
H	-1.89724500	2.53364100	2.75885500
C	-3.29760400	1.67013100	1.34712700
H	-3.36729600	1.08461100	0.43041700
C	-4.43718500	2.10469000	1.92272400
H	-4.38174700	2.66592900	2.85481800
C	-5.74831100	1.87157200	1.36673600
H	-5.78635800	1.39385900	0.38930200
C	-6.91194900	2.18540900	1.96566600
H	-6.90380000	2.63715900	2.95687600
C	8.65544900	-0.44499500	1.44164700
H	9.67341200	-0.14562900	1.70099000
H	8.22277200	-0.93028000	2.32165800
C	-8.20034700	1.91943900	1.35607100
H	-8.16763800	1.58271000	0.32199100
C	-9.38729400	2.01543400	1.96519600

H	-9.43195800	2.34013600	3.00575800
C	-10.69898900	1.69445700	1.30522300
H	-10.51095600	1.51987700	0.24135700
C	-11.33145400	0.40442200	1.86178400
H	-11.75584500	0.63907600	2.84935100
C	-10.36784900	-0.77686100	2.05785700
H	-9.58156700	-0.41010400	2.72509000
C	-9.64660800	-1.18049800	0.76728400
H	-9.31612000	-0.29030100	0.24005900
C	-7.28997900	-1.57274300	0.62356000
C	-6.14275900	-2.41209500	1.12551000
H	-6.35825300	-2.74844600	2.14172100
H	-6.09081200	-3.30159500	0.48658900
C	-4.80579900	-1.67154200	1.05861300
H	-4.85799100	-0.77833500	1.69655600
C	-3.65691500	-2.55413900	1.52226000
H	-3.83856300	-2.84980500	2.56110300
H	-3.63743000	-3.46718900	0.91512400
C	-2.28761800	-1.88473700	1.42891900
H	-2.32291100	-0.90907000	1.93223300
C	-1.20488900	-2.73582400	2.08168300
H	-1.29611100	-3.76560600	1.71464600
H	-1.41282400	-2.76667200	3.15552700
C	0.21900000	-2.21384800	1.83019100
H	0.19196500	-1.16041600	1.54112900
H	0.80331200	-2.25986900	2.75427700

C	0.99058000	-3.00510200	0.77643500
H	1.15458500	-4.02225900	1.15905000
C	2.36856200	-2.36259200	0.48310800
H	2.69332800	-1.77083700	1.34656200
C	3.44686300	-3.38514500	0.14250300
H	3.13443800	-3.96052700	-0.73742800
H	3.54518100	-4.08673000	0.97893900
C	4.80306100	-2.73625600	-0.11811000
H	5.07397200	-2.11819800	0.74864800
C	5.90273200	-3.76861800	-0.32874500
H	5.63991300	-4.42311400	-1.16549200
H	5.98409000	-4.38106500	0.57384500
C	7.26755200	-3.14709200	-0.62719200
C	8.39223900	-4.17225500	-0.61317500
H	8.26237000	-4.88852700	-1.42717200
H	8.36874700	-4.71763300	0.33452300
C	9.75514000	-3.51394100	-0.74354600
H	9.83535300	-3.06464000	-1.74417800
C	9.90467700	-2.37868500	0.30663100
H	10.00567700	-2.86537000	1.28395300
C	8.68067900	-1.45121200	0.29731500
H	8.66739100	-0.91573600	-0.65644700
C	11.16461800	-1.60006300	0.02872100
C	-10.39757300	-2.08524800	-0.18876000
H	-9.80421900	-2.24531000	-1.09210900
H	-11.34353300	-1.61815700	-0.46398900

H	-10.60198900	-3.05634600	0.26683800
C	-11.06079400	-1.95444200	2.73997700
H	-11.90570100	-2.32109800	2.15016500
H	-11.43511700	-1.66561600	3.72633200
H	-10.36630600	-2.78672100	2.87496100
C	-11.67716300	2.86765700	1.43175500
H	-11.87514400	3.08943600	2.48639000
H	-12.62428100	2.65221700	0.93521800
H	-11.24825700	3.76580000	0.98032400
O	9.04576500	3.28124500	-1.91170600
H	8.22859700	2.81965700	-1.67580800
O	11.88815700	5.63395400	0.17229000
H	12.30122900	5.73279200	-0.70089500
O	9.23666800	3.30380600	1.01030200
O	8.43611300	1.46256300	-0.03892700
O	7.29231400	-2.51312300	-1.87269100
H	6.44284900	-2.04334500	-1.96079300
O	7.49533400	-2.22126100	0.43087700
O	11.24386900	-0.44190500	-0.30579000
O	12.27935600	-2.36336100	0.15503200
H	13.03030200	-1.79434100	-0.08075100
O	4.75668200	-1.90247900	-1.27898600
H	3.86139200	-1.51897300	-1.32471200
O	2.20370700	-1.44203700	-0.59687500
H	1.47265700	-1.83845800	-1.11258300
O	0.30737900	-3.11335000	-0.46430600

H	-0.55064900	-2.64301800	-0.40546300
O	-1.91319600	-1.67349200	0.06409600
H	-2.71463500	-1.41358100	-0.42432200
O	-4.52182600	-1.27396000	-0.27972400
H	-5.30859000	-0.81155200	-0.61659000
O	-7.16418800	-0.71159700	-0.23250000
O	-8.44237000	-1.89195700	1.19334100
O	-12.39687900	0.05039200	0.96900100
H	-13.08100200	-0.42219900	1.45501900
O	10.70729000	-4.53922600	-0.56138600
H	11.58460000	-4.13691500	-0.53499300
N	11.66539400	4.28539600	-2.25875400
H	10.84187800	4.55102600	-2.79458900
H	12.18330400	3.62250000	-2.82684700

AmB-Dod5

0 1

C	-9.29036600	-2.24237200	3.28776000
H	-9.94216500	-1.39205400	3.51283700
H	-9.64880500	-3.10204200	3.86094500
H	-9.41981800	-2.47668700	2.22546800
C	-7.83459800	-1.91454000	3.60906100
H	-7.19914500	-2.78251700	3.39573500
H	-7.73448500	-1.71631500	4.68339400

C	-7.30597900	-0.72214300	2.81860800
H	-7.94826300	0.15246800	2.98965300
H	-7.38646800	-0.95612800	1.75067300
C	-5.85725200	-0.36570800	3.13785300
H	-5.24285700	-1.26516500	3.02631700
H	-5.77424400	-0.06900400	4.19202100
C	-5.31574400	0.74834500	2.24689500
H	-5.86309200	1.67677100	2.44961200
H	-5.53324400	0.52119800	1.19756200
C	-3.81492500	0.98915600	2.38829400
H	-3.27382200	0.10188000	2.03082700
H	-3.54876600	1.10455200	3.44803600
C	-3.36446600	2.21754900	1.60425000
H	-3.88156500	3.09737500	2.01014300
H	-3.71011200	2.12677900	0.56870300
C	-1.86041500	2.47434800	1.61907500
H	-1.33189800	1.58953100	1.24134700
H	-1.51511800	2.62294800	2.65120900
C	-1.48453600	3.68255600	0.76634600
H	-2.04559200	4.56040900	1.11257200
H	-1.81777100	3.49705600	-0.26228400
C	0.00339300	4.01669400	0.74943500
H	0.55946800	3.12717600	0.43411000
H	0.34214100	4.25971100	1.76590200
C	0.32230500	5.17903800	-0.18876800
H	-0.21869200	6.07503500	0.14172200

H	-0.04187000	4.94318900	-1.19598200
C	1.80490500	5.51051100	-0.28749400
H	1.94982500	6.39288300	-0.91788200
H	2.20193100	5.75642600	0.71000300
O	2.56324800	4.47626500	-0.89229700
H	2.57251700	3.71359700	-0.30225200
C	-8.37858900	3.96987200	-0.40320300
H	-8.62924900	4.34788100	-1.40238900
C	-7.11123400	4.70359700	0.03074900
H	-6.82445400	4.35235100	1.03465200
C	-5.96753700	4.36428800	-0.91678600
H	-6.27186600	4.65410800	-1.93787800
C	-6.84094100	2.19749600	-1.29648200
H	-7.03388800	2.42088900	-2.36554700
C	-8.12549200	2.46770200	-0.50088500
H	-8.94826200	1.97997800	-1.04710300
C	-4.67624000	5.05987700	-0.55506600
H	-4.37326600	4.77872900	0.45498700
H	-4.81905700	6.14090000	-0.59348300
H	-3.87961600	4.76948100	-1.24324900
C	-5.71988400	0.19504400	-2.06928000
H	-5.70127100	0.77764600	-3.00011800
C	-4.32964100	0.06432600	-1.52481300
H	-4.21639900	-0.56345200	-0.64525100
C	-3.25095400	0.66890500	-2.03180500
H	-3.34399300	1.31721400	-2.90247100

C	-1.93408000	0.50396800	-1.44968300
H	-1.87406100	-0.17590000	-0.60251900
C	-0.80777900	1.11833700	-1.86004200
H	-0.84226900	1.81815700	-2.69427500
C	0.46502400	0.88075100	-1.22105200
H	0.45911100	0.13720300	-0.42663200
C	1.63039000	1.48481100	-1.53014100
H	1.66608700	2.25700000	-2.29596000
C	2.85976200	1.17351400	-0.83824300
H	2.81701800	0.38116700	-0.09061000
C	4.04493400	1.78192100	-1.06090700
H	4.10119300	2.58157200	-1.79739600
C	5.25227500	1.45562800	-0.34264000
H	5.18656200	0.66795200	0.40767100
C	6.43944800	2.06043700	-0.54824800
H	6.51189000	2.83742100	-1.30822900
C	7.63895200	1.74271000	0.18800100
H	7.54491700	1.00815000	0.98605800
C	8.85119900	2.27247200	-0.05824500
H	8.96659400	2.98955700	-0.87021700
C	-6.41852600	-1.14408800	-2.31549400
H	-7.43519500	-0.91553400	-2.64562900
H	-5.91502800	-1.70542900	-3.10761700
C	10.04188000	1.89916100	0.67990500
H	9.88006500	1.26129300	1.54654000
C	11.29165300	2.22930800	0.33644200

H	11.45349900	2.85504400	-0.54287200
C	12.52813300	1.75728800	1.04674600
H	12.23479200	1.25199000	1.97414000
C	13.30006700	0.73039600	0.19478700
H	13.72666800	1.27288100	-0.66528100
C	12.44137100	-0.40743400	-0.38811400
H	11.67003900	0.08076100	-0.99182300
C	11.67944100	-1.19375700	0.68357500
H	11.22127700	-0.50503900	1.38748500
C	9.35921300	-1.75550800	0.47115800
C	8.33965300	-2.51042800	-0.34493500
H	8.70842500	-2.63191900	-1.36514200
H	8.24803100	-3.50917900	0.09791400
C	6.96676800	-1.83319300	-0.33123200
H	7.05166700	-0.83859200	-0.79178400
C	5.93786400	-2.65660400	-1.09364200
H	6.28292200	-2.78679700	-2.12495500
H	5.87413300	-3.65092500	-0.63550000
C	4.54038400	-2.04159400	-1.11728800
H	4.59737500	-1.02307400	-1.52526700
C	3.58461800	-2.86378600	-1.97640800
H	3.68154200	-3.92126600	-1.70097200
H	3.91930900	-2.77874500	-3.01474600
C	2.11621600	-2.42463500	-1.85496300
H	2.05739500	-1.39398300	-1.49782300
H	1.64183400	-2.43039500	-2.84115300

C	1.26874100	-3.32067400	-0.95164300
H	1.16738700	-4.30362600	-1.43253800
C	-0.14146300	-2.72525600	-0.73843700
H	-0.42379500	-2.11444800	-1.60363000
C	-1.21345300	-3.78141400	-0.49117600
H	-0.95179500	-4.35106500	0.40855100
H	-1.22625400	-4.48159000	-1.33416800
C	-2.60426200	-3.17246900	-0.34144200
H	-2.84149100	-2.60305400	-1.25011000
C	-3.68291800	-4.23312000	-0.14769700
H	-3.45407000	-4.83456200	0.73732400
H	-3.68997800	-4.89268300	-1.02032600
C	-5.07865800	-3.63704300	0.03134300
C	-6.19001200	-4.67456700	0.04131300
H	-6.07002600	-5.35803400	0.88462400
H	-6.15210000	-5.25377600	-0.88546900
C	-7.54893300	-4.00259200	0.14117100
H	-7.62470400	-3.52199500	1.12527100
C	-7.69997800	-2.90341800	-0.94918700
H	-7.88465700	-3.42947300	-1.89321400
C	-6.45225500	-2.01417200	-1.06009500
H	-6.42424400	-1.37728400	-0.17360400
C	-8.90578700	-2.05590600	-0.62354800
C	12.46008400	-2.24705400	1.44264400
H	11.82968900	-2.69476100	2.21440300
H	13.32552300	-1.77818400	1.91306600

H	12.80483200	-3.04080300	0.77638300
C	13.25923900	-1.30925000	-1.31076900
H	14.09405300	-1.78223600	-0.78494900
H	13.66781500	-0.73658100	-2.14838200
H	12.63686400	-2.10789700	-1.72064500
C	13.44363900	2.92863800	1.41349600
H	13.73644800	3.48548300	0.51646600
H	14.34919200	2.56503200	1.90110300
H	12.93224000	3.62226100	2.08467300
O	-8.04184000	1.95999900	0.80895700
H	-7.89440000	1.00653700	0.73380700
O	-7.34234700	6.09275100	0.01170800
H	-8.23495500	6.18807000	0.38230400
O	-5.74014100	2.95898900	-0.86475600
O	-6.55709900	0.85866800	-1.11646500
O	-5.18240000	-2.91091300	1.22197200
H	-4.34337100	-2.42458600	1.33003000
O	-5.27422100	-2.80254500	-1.10669000
O	-8.91614300	-0.87952900	-0.34948200
O	-10.05325200	-2.78033100	-0.60574600
H	-10.75587700	-2.17217600	-0.32308200
O	-2.65691900	-2.28130000	0.77741700
H	-1.76267000	-1.91456400	0.91326900
O	-0.06727700	-1.83412900	0.37805200
H	0.62069300	-2.25022600	0.93748400
O	1.82600900	-3.52171000	0.33900700

H	2.66970500	-3.02391800	0.40707600
O	3.99114700	-1.96302600	0.20158600
H	4.72660100	-1.79436100	0.82010600
O	6.49231200	-1.69365900	1.00564600
H	7.22605900	-1.33156000	1.53498200
O	9.07403500	-1.13316000	1.48249300
O	10.58533100	-1.86653200	-0.01726600
O	14.35202400	0.23878100	1.01970600
H	15.02129500	-0.16726900	0.45867300
O	-8.51455800	-5.02075000	-0.00679200
H	-9.38754100	-4.60956800	0.02714900
N	-9.45624400	4.35129900	0.50421800
H	-9.36599100	3.78779000	1.34689300
H	-10.36056300	4.12938900	0.10029300

AmB-Dod6

0 1

C	9.44588900	3.54302300	-0.77888300
H	10.21494500	2.87363500	-1.18340500
C	9.89075500	3.93057500	0.63073900
H	9.16222700	4.64263100	1.04526900
C	9.91822800	2.70745900	1.53805000
H	10.63826300	1.98647400	1.11673800
C	8.21560500	1.65585200	0.29226500

H	8.92795100	0.89738600	-0.06169200
C	8.11933000	2.78843800	-0.72947000
H	7.89772800	2.33977200	-1.70809000
C	10.29867100	3.04970800	2.96087100
H	9.55835900	3.73368800	3.38143000
H	11.27447200	3.53536300	2.96966400
H	10.33221300	2.14880400	3.57484300
C	6.67829600	0.20191400	1.46750000
H	6.82513800	0.71765100	2.42068500
C	5.22919700	-0.12515700	1.28637400
H	4.94442700	-0.47678300	0.29702000
C	4.28624400	0.08380100	2.21070300
H	4.54917000	0.45448700	3.19930100
C	2.88550700	-0.08055800	1.88230700
H	2.68080000	-0.42181900	0.86936100
C	1.83030800	0.26319700	2.64483700
H	1.97063500	0.63215100	3.65841100
C	0.50054300	0.23011500	2.08444400
H	0.44985000	-0.09612200	1.04678700
C	-0.64252900	0.61321700	2.68812500
H	-0.63581200	0.95555400	3.72081900
C	-1.89878400	0.60534400	1.97918200
H	-1.86684100	0.28569000	0.93655300
C	-3.08978600	0.97330500	2.49525200
H	-3.15008600	1.29969900	3.53179600
C	-4.31008800	0.96506800	1.72628400

H	-4.23415000	0.66424000	0.68014000
C	-5.51905000	1.30238500	2.22172600
H	-5.60029800	1.59439700	3.26733700
C	-6.73276400	1.30036200	1.43974900
H	-6.63496300	1.07356000	0.37862400
C	-7.95696900	1.54911500	1.94437100
H	-8.06167700	1.76276500	3.00685300
C	7.64138100	-0.99257000	1.43109200
H	8.64955000	-0.61483200	1.62786800
H	7.37456800	-1.66246400	2.25394200
C	-9.16770700	1.51532700	1.14557200
H	-9.02363100	1.39989700	0.07364200
C	-10.40733200	1.57328100	1.64866700
H	-10.53209700	1.68049700	2.72801600
C	-11.67510000	1.47194200	0.84778300
H	-11.43300300	1.50862300	-0.21929100
C	-12.38879900	0.13275200	1.11124300
H	-12.76562100	0.15560600	2.14634200
C	-11.48770600	-1.11086000	0.99434200
H	-10.72074300	-0.99805900	1.76679900
C	-10.72479800	-1.16967100	-0.33216000
H	-10.31955000	-0.18910100	-0.56201000
C	-8.39988600	-1.65415500	-0.55112900
C	-7.29877700	-2.64109400	-0.24911400
H	-7.60848600	-3.28846100	0.57174600
H	-7.16775600	-3.26126600	-1.14306000

C	-5.97026800	-1.95018600	0.07383400
H	-6.09977200	-1.32031200	0.96527200
C	-4.88011000	-2.98146600	0.33609600
H	-5.17961800	-3.59508300	1.19178900
H	-4.79395800	-3.63911500	-0.53713000
C	-3.50784400	-2.37710400	0.62423300
H	-3.60052500	-1.62480100	1.41912000
C	-2.51305300	-3.44499400	1.06855300
H	-2.56679600	-4.28589100	0.36654900
H	-2.83776700	-3.82076600	2.04156900
C	-1.06958200	-2.91777300	1.14950000
H	-1.07172600	-1.82558600	1.13299700
H	-0.60558000	-3.21238500	2.09399700
C	-0.17201200	-3.41715500	0.01956000
H	-0.02885700	-4.49973400	0.12796700
C	1.21202900	-2.73111300	0.05993900
H	1.46457000	-2.47940500	1.09666500
C	2.31939900	-3.59445900	-0.53571700
H	2.05794200	-3.85867200	-1.56765200
H	2.38179600	-4.52392600	0.04058100
C	3.68747000	-2.91244400	-0.51544100
H	3.90295300	-2.55962300	0.50317400
C	4.78718900	-3.87762000	-0.93596400
H	4.53125100	-4.32961400	-1.89949200
H	4.84917100	-4.67338900	-0.18912200
C	6.17002700	-3.24114600	-1.08717000

C	7.24973800	-4.30401900	-1.24129100
H	7.11408600	-4.83477400	-2.18524200
H	7.17084700	-5.02222500	-0.42111200
C	8.64446200	-3.70761700	-1.19756900
H	8.80265300	-3.07935500	-2.08558500
C	8.78706700	-2.80862600	0.05989900
H	8.75524100	-3.47210500	0.93080000
C	7.63311000	-1.79524100	0.13702500
H	7.67861000	-1.12315800	-0.72699000
C	10.17453300	-2.22706100	0.00093100
C	-11.48742000	-1.69451300	-1.53306600
H	-10.86454000	-1.62366000	-2.42688500
H	-12.38777000	-1.09599200	-1.67662000
H	-11.77204800	-2.73834100	-1.38912800
C	-12.26919800	-2.39172300	1.28276800
H	-13.07737000	-2.54364700	0.56239600
H	-12.70515300	-2.35727100	2.28452700
H	-11.61495200	-3.26401200	1.23001500
C	-12.61638200	2.63886100	1.16237500
H	-12.85850200	2.65744800	2.22966900
H	-13.54404900	2.54053900	0.59903400
H	-12.14356900	3.58888100	0.90758400
O	7.10252700	3.70482500	-0.37443900
H	6.43336200	3.22130500	0.13074800
O	11.18424400	4.48592000	0.58689900
H	11.17059500	5.08770300	-0.16861200

O	8.62033700	2.12094100	1.55797800
O	6.93186400	1.13487200	0.40338600
O	6.24603400	-2.40169700	-2.20386400
H	5.43845700	-1.86643400	-2.20431500
O	6.40246200	-2.50744700	0.11195700
O	11.17903900	-2.86622100	0.18152500
O	10.22804600	-0.93418400	-0.37120000
H	11.16572000	-0.70297900	-0.43882300
O	3.72000100	-1.79361500	-1.40419100
H	2.85816700	-1.35618400	-1.34440800
O	1.11881500	-1.48760700	-0.63969900
H	0.47465200	-1.65810100	-1.34683500
O	-0.72190600	-3.18198000	-1.26884100
H	-1.55996400	-2.69745100	-1.17369700
O	-2.96356900	-1.74587900	-0.53800700
H	-3.68476600	-1.28449200	-0.99134500
O	-5.53639600	-1.13521800	-1.01123400
H	-6.29316900	-0.61326800	-1.31626900
O	-8.21304300	-0.62152300	-1.16004900
O	-9.58115200	-2.04632500	-0.09821500
O	-13.48765400	0.08481000	0.20792600
H	-14.10258700	-0.58907500	0.50678400
O	9.54452000	-4.79133500	-1.17397700
H	10.41113500	-4.47326000	-0.89689100
N	9.43455600	4.75866700	-1.58867900
H	8.58737700	5.27844300	-1.37289200

H	9.40407900	4.53578800	-2.57739500
C	-9.83194400	2.71883600	-2.28771200
H	-9.86985600	1.62760700	-2.19279300
H	-10.64075400	3.02882100	-2.95236600
H	-10.02212400	3.14970000	-1.29972100
C	-8.46422000	3.15383600	-2.80966000
H	-8.43303000	4.24416900	-2.90900300
H	-8.30738900	2.74543200	-3.81378800
C	-7.32906200	2.69129200	-1.89920300
H	-7.39101000	1.60018700	-1.79600700
H	-7.47686500	3.10445800	-0.89284400
C	-5.94216900	3.07622100	-2.40565900
H	-5.86288400	4.16875000	-2.45958900
H	-5.81578700	2.70713900	-3.43092300
C	-4.81303900	2.52864100	-1.53466800
H	-4.87876700	1.43184200	-1.50744300
H	-4.94774800	2.86883200	-0.50012100
C	-3.42788000	2.93540000	-2.03042800
H	-3.34331800	4.02855100	-2.00340200
H	-3.32343300	2.64628300	-3.08360000
C	-2.28035500	2.32331500	-1.22955200
H	-2.34543200	1.22717800	-1.28184800
H	-2.38645000	2.58693100	-0.16975000
C	-0.91136300	2.77445600	-1.73613700
H	-0.83760100	3.86521000	-1.64687100
H	-0.83535500	2.55033100	-2.80761600

C	0.26728700	2.13176000	-1.00873600
H	0.20507100	1.03898100	-1.10510400
H	0.20055100	2.34549800	0.06541700
C	1.61733800	2.61097600	-1.54267200
H	1.72702400	3.68597900	-1.36962200
H	1.64709400	2.46343500	-2.63002000
C	2.80312800	1.88722300	-0.91130500
H	2.73318200	0.81343700	-1.11916900
H	2.77539300	2.00259700	0.17743100
C	4.14550300	2.39249000	-1.40652600
H	4.95012900	1.75378900	-1.02006300
H	4.17268700	2.35262600	-2.50514500
O	4.30490400	3.72882500	-0.94861800
H	5.16342600	4.04757700	-1.24298400

AmB-Dod7

0 1

C	5.01864500	3.26781400	-0.59956400
H	5.02243200	2.20035600	-0.84280100
H	5.88629000	3.45085900	0.03745200
H	5.15376700	3.82993800	-1.52970800
C	3.70968700	3.64415500	0.08419900
H	3.70862800	4.71691400	0.31640900
H	3.63674300	3.12252100	1.04627400

C	2.47892900	3.30641700	-0.75195800
H	2.44871500	2.22196500	-0.91887900
H	2.57467400	3.76674600	-1.74468200
C	1.16906000	3.75456800	-0.11208600
H	1.18778800	4.84421900	0.02514600
H	1.08951500	3.32025100	0.89252900
C	-0.06582600	3.36671400	-0.91922900
H	-0.11740200	2.27285800	-0.99143700
H	0.03842500	3.73795100	-1.94759500
C	-1.36703200	3.89365900	-0.32276500
H	-1.31928900	4.98920500	-0.26120500
H	-1.46460500	3.53169300	0.70842400
C	-2.60655800	3.48540400	-1.11236600
H	-2.67876000	2.39030700	-1.12553200
H	-2.49292100	3.79855100	-2.15882000
C	-3.89960500	4.06592600	-0.54875400
H	-3.82866300	5.16176000	-0.53194700
H	-4.01186300	3.74791000	0.49531600
C	-5.14114400	3.65095500	-1.33128200
H	-5.22064400	2.55653500	-1.32338300
H	-5.02721200	3.94331100	-2.38332500
C	-6.43170000	4.24710100	-0.77834600
H	-6.34582800	5.34206500	-0.78605900
H	-6.54605100	3.94464100	0.27077300
C	-7.67431900	3.82663800	-1.55789800
H	-7.74009600	2.73250500	-1.56830100

H	-7.58667400	4.15486700	-2.60000500
C	-8.96733300	4.38497800	-0.97755100
H	-9.82436700	4.02448900	-1.55462200
H	-9.09916500	4.01948400	0.05177800
O	-9.03272000	5.80072000	-1.03211600
H	-8.36288800	6.14931800	-0.43389100
C	10.08020400	3.13537500	-0.77035100
H	10.93185100	2.65884800	-0.26886300
C	9.57726800	4.24232800	0.15523200
H	8.73020100	4.74603800	-0.33591700
C	9.06557900	3.64962800	1.46262800
H	9.90563300	3.12738900	1.95136600
C	8.44840600	1.63696600	0.36900600
H	9.23256300	1.07308600	0.89936100
C	8.99382900	2.08655600	-0.98359200
H	9.41235500	1.19629400	-1.47527700
C	8.49629000	4.69423500	2.39520600
H	7.64477700	5.18858200	1.91981000
H	9.25883500	5.44245100	2.61693300
H	8.15487400	4.23346400	3.32473500
C	6.76534600	0.15218200	1.21856400
H	6.80360200	0.80738100	2.09509800
C	5.33995900	-0.09873700	0.84100300
H	5.17712900	-0.64945000	-0.08176000
C	4.29136700	0.33695100	1.54557800
H	4.44818900	0.89436300	2.46840300

C	2.92570100	0.13744100	1.10718600
H	2.79523200	-0.40248300	0.17270600
C	1.82964900	0.59666900	1.74044200
H	1.93383600	1.14867800	2.67392400
C	0.50233800	0.40460200	1.20931900
H	0.44477700	-0.15581400	0.27810000
C	-0.63985100	0.85452200	1.76693700
H	-0.60648800	1.41658000	2.69947600
C	-1.92906100	0.62750000	1.16456100
H	-1.94095500	0.05005700	0.24027500
C	-3.10633300	1.06368500	1.65783800
H	-3.12284100	1.63121300	2.58744400
C	-4.36468800	0.83331700	0.99523900
H	-4.32861100	0.28619000	0.05334900
C	-5.56477600	1.24618100	1.45084000
H	-5.62351900	1.77924400	2.39899400
C	-6.79480700	1.02963700	0.72981100
H	-6.70678500	0.55247200	-0.24482300
C	-8.02295300	1.36553500	1.16528800
H	-8.14114500	1.82498800	2.14591200
C	7.57896300	-1.11945500	1.49052000
H	8.58759800	-0.82068700	1.78405400
H	7.13864500	-1.66487900	2.33065400
C	-9.21989000	1.11669400	0.38666700
H	-9.05280900	0.74668100	-0.62286800
C	-10.47459900	1.26609400	0.82457000

H	-10.64618700	1.62271400	1.84144800
C	-11.69980200	0.93351700	0.02232200
H	-11.40258800	0.74180500	-1.01509500
C	-12.37736100	-0.34924700	0.54326400
H	-12.81103200	-0.11419600	1.52944300
C	-11.42833600	-1.54412900	0.75543300
H	-10.66730300	-1.19879700	1.46178200
C	-10.65904300	-1.93249600	-0.51194100
H	-10.28305600	-1.03935900	-1.00246700
C	-8.29772000	-2.34817100	-0.52450600
C	-7.19125400	-3.21673700	0.02007300
H	-7.50591100	-3.65609600	0.96846500
H	-7.04218400	-4.03507300	-0.69408600
C	-5.87521100	-2.44986800	0.17722500
H	-6.01877400	-1.63619400	0.90236300
C	-4.75878200	-3.36417900	0.66209500
H	-5.04332800	-3.78276000	1.63344100
H	-4.65173800	-4.19847100	-0.04147400
C	-3.40352300	-2.67377600	0.79944400
H	-3.50897900	-1.78330300	1.43393000
C	-2.36567900	-3.60220100	1.42263400
H	-2.41572800	-4.57637300	0.92096100
H	-2.65711700	-3.77060200	2.46379100
C	-0.93038800	-3.05408500	1.35203100
H	-0.94401600	-1.97037300	1.21481800
H	-0.41264500	-3.23336000	2.29938200

C	-0.08158100	-3.69327700	0.25413000
H	0.07458000	-4.75025400	0.51054500
C	1.30359100	-3.00836700	0.12895000
H	1.55922300	-2.50987700	1.07112700
C	2.41597300	-3.98277000	-0.24254400
H	2.16505700	-4.46969700	-1.19278100
H	2.47112900	-4.76377500	0.52465800
C	3.77964100	-3.30712400	-0.35676100
H	3.99791600	-2.77785200	0.58086400
C	4.89399800	-4.31569700	-0.60623900
H	4.66747900	-4.90312100	-1.50144200
H	4.94260400	-4.99799900	0.24734800
C	6.26695300	-3.67295500	-0.80468600
C	7.39384400	-4.69578600	-0.82431900
H	7.29355800	-5.35432900	-1.68960200
H	7.34114300	-5.30496700	0.08243900
C	8.75789900	-4.02784400	-0.86442000
H	8.86794300	-3.50967300	-1.82819100
C	8.86929100	-2.96864400	0.26640500
H	8.94483000	-3.52232800	1.20987800
C	7.64095500	-2.04724300	0.28386000
H	7.65488900	-1.44782300	-0.63063700
C	10.13148300	-2.16495600	0.08305200
C	-11.39508100	-2.78922200	-1.52121000
H	-10.77081100	-2.94706400	-2.40365600
H	-12.31225900	-2.27803100	-1.81688500

H	-11.65098800	-3.76402700	-1.10064500
C	-12.15503100	-2.72825700	1.39019100
H	-12.97830800	-3.08491700	0.76399400
H	-12.56696700	-2.45115800	2.36483800
H	-11.47122300	-3.56685500	1.54028000
C	-12.69529900	2.09669900	0.02131000
H	-12.99906200	2.34643200	1.04393900
H	-13.58859500	1.83248100	-0.54621500
H	-12.24455500	2.98900900	-0.41954000
O	7.98325900	2.67609300	-1.77488000
H	7.17671400	2.16084800	-1.63735100
O	10.62535700	5.14198300	0.43350100
H	11.07675600	5.24902000	-0.41956300
O	8.02588400	2.72029800	1.17108700
O	7.33770600	0.84803500	0.09885500
O	6.33560500	-2.95099700	-1.99973600
H	5.49212700	-2.46928000	-2.07934600
O	6.45432500	-2.82610900	0.32533400
O	10.21227400	-0.98244800	-0.15181500
O	11.24703400	-2.93171600	0.17278300
H	12.00040000	-2.34261600	0.00345100
O	3.79142700	-2.36054500	-1.42869500
H	2.89435700	-1.98396800	-1.49409200
O	1.19773200	-1.97972200	-0.85734300
H	0.48780200	-2.31754600	-1.44301000
O	-0.68724200	-3.64764600	-1.02990000

H	-1.54514200	-3.17462800	-0.96544600
O	-2.91084900	-2.25670700	-0.47751700
H	-3.67674700	-1.97133300	-1.00928900
O	-5.46456700	-1.89830900	-1.07109800
H	-6.24678900	-1.47798300	-1.47265300
O	-8.10150200	-1.44978000	-1.32863100
O	-9.48904300	-2.68400000	-0.05450700
O	-13.41997100	-0.65145600	-0.37853500
H	-14.04906800	-1.24305300	0.04792500
O	9.70768600	-5.06165500	-0.72379000
H	10.58263000	-4.66038200	-0.64842200
N	10.57264400	3.76796600	-1.99067900
H	9.76900400	3.99802400	-2.57123900
H	11.14408300	3.12065500	-2.52429900

AmB-Dod8

0 1

C	0.68543200	2.90778700	3.03757800
H	0.94719900	3.23624500	2.02781100
H	1.42552900	2.16872300	3.35841200
H	0.77381800	3.77477600	3.69941100
C	-0.73190200	2.34322900	3.06311900
H	-0.99224900	2.03718700	4.08405900
H	-0.78047600	1.43087000	2.45418700

C	-1.76952800	3.33410500	2.54148500
H	-1.51138000	3.59559300	1.50909600
H	-1.70971900	4.26549700	3.12001700
C	-3.19690900	2.79660900	2.59014400
H	-3.51343700	2.67596800	3.63446600
H	-3.22604200	1.79493700	2.14608700
C	-4.19597600	3.67520600	1.84398600
H	-3.89902300	3.71676300	0.78664100
H	-4.13814100	4.70741900	2.21352800
C	-5.63298000	3.17063100	1.94185000
H	-5.98332100	3.25068300	2.97881000
H	-5.64555800	2.10157300	1.69629100
C	-6.59330600	3.90922800	1.01391300
H	-6.21900300	3.82690600	-0.01691100
H	-6.58976100	4.98228800	1.24381000
C	-8.02064000	3.37219900	1.06457400
H	-8.44682000	3.53809200	2.06146800
H	-7.99477600	2.28245200	0.94246100
C	-8.94735800	3.96209800	-0.00006000
H	-8.39521000	4.07723500	-0.94243000
H	-9.25905100	4.97301600	0.29114100
C	-10.16405300	3.07742900	-0.25016300
H	-10.65287100	2.84591200	0.70187800
H	-9.81840900	2.11275700	-0.64137600
C	-11.18928200	3.67348900	-1.20965800
H	-10.68740200	4.09534500	-2.08969200

H	-11.70841200	4.51270800	-0.72674200
C	-12.20286400	2.64439100	-1.69476100
H	-12.96927400	3.12388800	-2.32036100
H	-11.69984400	1.89533200	-2.31287700
O	-12.80280200	1.90552200	-0.63918000
H	-13.23573800	2.52726800	-0.04190500
C	11.41658800	-2.78366200	1.56466800
H	12.19422200	-2.27687800	0.97948900
C	11.15679300	-4.13155900	0.89346200
H	10.38987400	-4.66514500	1.47620600
C	10.60349900	-3.92583200	-0.51155000
H	11.35620800	-3.36919700	-1.09583400
C	9.59859400	-1.87892700	0.12030300
H	10.29212800	-1.31074700	-0.51851900
C	10.15548500	-1.92685800	1.54037000
H	10.38839000	-0.89294600	1.83500100
C	10.27297000	-5.22680100	-1.20695800
H	9.50277400	-5.76046400	-0.64378600
H	11.16592000	-5.85147900	-1.26163600
H	9.89698400	-5.03787500	-2.21491600
C	7.68338300	-0.98335400	-1.02194300
H	7.81220600	-1.84248500	-1.68830600
C	6.23774600	-0.83592100	-0.66489600
H	5.99008400	-0.04504900	0.03827900
C	5.27065300	-1.62622200	-1.14072400
H	5.51792500	-2.42167900	-1.84311900

C	3.88397900	-1.49760800	-0.74015200
H	3.65764100	-0.70216300	-0.03428200
C	2.88244700	-2.29740000	-1.15321400
H	3.09103400	-3.10296800	-1.85669100
C	1.52643600	-2.14498200	-0.68298400
H	1.35673100	-1.32182900	0.00792300
C	0.48244500	-2.92125000	-1.03677700
H	0.62974600	-3.74656600	-1.73253700
C	-0.84654700	-2.71226100	-0.51775100
H	-0.97646900	-1.86881000	0.16043200
C	-1.92458400	-3.46657200	-0.81507200
H	-1.81979500	-4.30214400	-1.50614800
C	-3.22744800	-3.24064900	-0.24057200
H	-3.31032600	-2.42312400	0.47589500
C	-4.33195200	-3.96198500	-0.52044000
H	-4.27244200	-4.76846400	-1.25036800
C	-5.60809000	-3.72892300	0.11220500
H	-5.63071700	-2.97170700	0.89440300
C	-6.75786900	-4.35685800	-0.19572000
H	-6.77232100	-5.09704500	-0.99482100
C	8.30323700	0.26141000	-1.66934700
H	9.34960300	0.04290900	-1.89420000
H	7.80054600	0.46823000	-2.61886000
C	-8.01003400	-4.05899000	0.47329700
H	-7.92878700	-3.41167900	1.34454000
C	-9.22134900	-4.46488600	0.07706000

H	-9.30811900	-5.10188500	-0.80468200
C	-10.51161200	-4.06912000	0.73868800
H	-10.28378900	-3.54234600	1.67259800
C	-11.32125100	-3.09502500	-0.14063900
H	-11.68871600	-3.66452100	-1.01166200
C	-10.51423500	-1.91231300	-0.70502500
H	-9.71298900	-2.36418800	-1.29918400
C	-9.80317600	-1.09819800	0.37944100
H	-9.26134400	-1.76730500	1.04004000
C	-7.54126600	-0.34596800	0.12452900
C	-6.57722400	0.39421400	-0.77080000
H	-6.92437400	0.32146300	-1.80375800
H	-6.60941600	1.45181600	-0.49006600
C	-5.14288800	-0.12106200	-0.64060800
H	-5.10342300	-1.16245100	-0.98917200
C	-4.17646100	0.72128500	-1.46001300
H	-4.48708400	0.69187800	-2.51001700
H	-4.23842900	1.76334800	-1.12279200
C	-2.72368200	0.26421300	-1.35973100
H	-2.66013000	-0.80615600	-1.59802600
C	-1.82158500	1.03396000	-2.31844800
H	-2.03805900	2.10526400	-2.22599900
H	-2.09873300	0.74408700	-3.33635500
C	-0.32426700	0.77961000	-2.07734000
H	-0.18430100	-0.14944800	-1.51988800
H	0.19614500	0.64478000	-3.03037700

C	0.37777100	1.91853700	-1.33996700
H	0.41686900	2.79273700	-2.00393300
C	1.82149100	1.53759800	-0.94063800
H	2.21702900	0.79273700	-1.64053300
C	2.76310100	2.73607000	-0.89487600
H	2.38122900	3.46985800	-0.17487100
H	2.77138600	3.21431900	-1.88109300
C	4.19101600	2.34511000	-0.52736700
H	4.53910500	1.57308100	-1.22701100
C	5.14742100	3.52752900	-0.60883000
H	4.80630400	4.32447900	0.05913500
H	5.14180200	3.91154100	-1.63297400
C	6.58428900	3.17216200	-0.22512700
C	7.56307200	4.30015100	-0.51799000
H	7.34574000	5.16342300	0.11441100
H	7.46002700	4.60254700	-1.56396900
C	9.00222000	3.86910100	-0.29280600
H	9.14906900	3.67669000	0.78003900
C	9.29258600	2.55049700	-1.06116400
H	9.31760900	2.80570000	-2.12713600
C	8.20424400	1.50159400	-0.78902700
H	8.27265000	1.20580100	0.26201500
C	10.64827300	2.02695000	-0.66307100
C	-10.65831400	-0.16608900	1.21135500
H	-10.02814300	0.37971100	1.91818200
H	-11.37939300	-0.76854600	1.76650700

H	-11.20892400	0.54964000	0.60011300
C	-11.35310200	-1.04355800	-1.64263000
H	-12.16782000	-0.52281600	-1.13150700
H	-11.77831500	-1.64884800	-2.44900900
H	-10.72560000	-0.27398700	-2.09793500
C	-11.36168200	-5.29624400	1.08124100
H	-11.58976400	-5.87167700	0.17727400
H	-12.30416900	-4.99015500	1.53735800
H	-10.82921100	-5.95464100	1.77147200
O	9.22145700	-2.50926800	2.42605500
H	8.34706400	-2.22702800	2.12227200
O	12.35700400	-4.86591800	0.81905500
H	12.78259200	-4.70899700	1.67762700
O	9.40218600	-3.16630200	-0.42255000
O	8.35679600	-1.26003400	0.21800400
O	6.70113600	2.85595300	1.13124300
H	5.91960900	2.32030600	1.35970200
O	6.92506200	2.06076600	-1.04832800
O	10.88513400	1.00349100	-0.06644800
O	11.64957000	2.87189800	-1.01588500
H	12.47247400	2.47637500	-0.68466300
O	4.25449400	1.82186600	0.80208700
H	3.41590600	1.35488600	0.97146100
O	1.77296600	0.89896100	0.33811900
H	1.01400200	1.33127100	0.77628300
O	-0.29264100	2.31321700	-0.15135800

H	-1.05519000	1.71597700	0.00787700
O	-2.21983900	0.45682300	-0.03325100
H	-2.93455200	0.22318200	0.58698300
O	-4.70589900	-0.06972500	0.71715900
H	-5.40499500	-0.48706000	1.25408800
O	-7.20474100	-0.89553900	1.16217000
O	-8.78413000	-0.31808600	-0.33261700
O	-12.42280500	-2.67315800	0.65360600
H	-13.01136300	-2.14210300	0.10519900
O	9.80845900	4.93730300	-0.73993600
H	10.73102200	4.65390900	-0.71124700
N	11.95553800	-3.04819400	2.89600800
H	11.17864900	-3.29483700	3.50546700
H	12.37772200	-2.21063800	3.28415300

AmB-Dod9

0 1

C	10.01736600	4.14794700	-0.98696100
H	10.82818100	3.67200200	-0.42109700
C	9.38114100	5.18452200	-0.06161400
H	8.59529200	5.71487100	-0.61855100
C	8.72828100	4.50108200	1.13306300
H	9.51159800	3.95447700	1.68341200
C	8.30018500	2.54943700	-0.12292800
H	9.02545900	1.98315200	0.48458700

C	8.99064400	3.08585600	-1.37619400
H	9.48095900	2.23796200	-1.87681800
C	8.03692700	5.48159200	2.05366300
H	7.23922700	5.99225500	1.50986900
H	8.75629400	6.22101000	2.40588000
H	7.60037900	4.96123700	2.90709400
C	6.49269600	1.09021300	0.45721600
H	6.35070100	1.79346900	1.28156000
C	5.16190900	0.77848800	-0.15734600
H	5.16678300	0.19246800	-1.07414300
C	4.00602100	1.19981600	0.36469100
H	4.01390200	1.78017900	1.28574300
C	2.71333200	0.96672500	-0.25097600
H	2.70001600	0.40374200	-1.18271700
C	1.55744600	1.45112100	0.24165200
H	1.57648900	2.02070000	1.16976700
C	0.27549900	1.28808800	-0.40450600
H	0.25816000	0.71258900	-1.32893200
C	-0.86857700	1.81800300	0.07643300
H	-0.83487500	2.39226100	1.00167900
C	-2.15653800	1.67981800	-0.55975300
H	-2.21625800	1.08902700	-1.47297600
C	-3.28531500	2.23032300	-0.06347600
H	-3.22912500	2.80993100	0.85751900
C	-4.58936600	2.09237700	-0.66717500
H	-4.65547200	1.51978400	-1.59131400

C	-5.71163800	2.60931500	-0.12371500
H	-5.63173800	3.16590500	0.80940000
C	-7.03644900	2.45937100	-0.68264100
H	-7.11552200	1.98003800	-1.65684500
C	-8.16153400	2.84921000	-0.05153300
H	-8.08372300	3.30526200	0.93480300
C	7.25104100	-0.14095900	0.96414700
H	8.21404300	0.18323200	1.36648700
H	6.67936800	-0.58344500	1.78592700
C	-9.49667100	2.65746400	-0.58834200
H	-9.55569600	2.30224300	-1.61473200
C	-10.62144800	2.84178300	0.11397400
H	-10.54667300	3.18769400	1.14692700
C	-12.01039500	2.58592400	-0.39754500
H	-11.95916000	2.33530500	-1.46232600
C	-12.66105100	1.39162900	0.32678600
H	-12.88995000	1.71474900	1.35504800
C	-11.76970900	0.14375900	0.44447400
H	-10.88947700	0.45996900	1.00926800
C	-11.23759000	-0.33752700	-0.90918100
H	-10.92662800	0.51548800	-1.50515200
C	-8.94352800	-0.84786500	-1.33487000
C	-7.77078000	-1.72138000	-0.95095200
H	-7.90001500	-2.06593800	0.07673900
H	-7.80558200	-2.59928300	-1.60670300
C	-6.41881700	-1.01926300	-1.12861600

H	-6.35564800	-0.18020300	-0.41902800
C	-5.26907900	-1.99294400	-0.88078700
H	-5.38953600	-2.43996500	0.11196300
H	-5.34165800	-2.79609700	-1.62447600
C	-3.87671000	-1.36373000	-0.96652700
H	-3.79980000	-0.57357000	-0.20075500
C	-2.78364700	-2.40346400	-0.71840100
H	-2.93510100	-3.18628400	-1.47351200
H	-2.92051000	-2.86823700	0.26392000
C	-1.38782800	-1.79113100	-0.83545400
H	-1.35065100	-1.17917700	-1.73982000
H	-1.20913500	-1.12468800	0.01442300
C	-0.25673500	-2.81123600	-0.90417500
H	-0.27259200	-3.46376800	-0.01938500
C	1.11472100	-2.13747100	-0.99948600
H	1.21933500	-1.41130500	-0.18658700
C	2.26604400	-3.14132900	-0.95164000
H	2.13799000	-3.86946300	-1.75971300
H	2.20983800	-3.68487900	-0.00099200
C	3.64987700	-2.49767900	-1.05151700
H	3.77712600	-1.77691200	-0.23274100
C	4.74709300	-3.55168000	-0.95353000
H	4.60437200	-4.30104100	-1.73821000
H	4.66709400	-4.05009500	0.01742500
C	6.16306900	-2.98812600	-1.09544400
C	7.22656900	-4.02105900	-0.75409900

H	7.20689800	-4.83439400	-1.48152300
H	7.02837100	-4.43386900	0.23939300
C	8.61069600	-3.39875300	-0.73875200
H	8.86890700	-3.07309500	-1.75560400
C	8.61953200	-2.15513600	0.18883900
H	8.53685200	-2.53670700	1.21446000
C	7.45298600	-1.20333900	-0.11316900
H	7.62291400	-0.72617000	-1.08366200
C	9.98376400	-1.51985700	0.05897700
C	-12.16611100	-1.21449800	-1.72742300
H	-11.71229200	-1.43287000	-2.69589200
H	-13.10786900	-0.68767600	-1.88619300
H	-12.36508800	-2.15744900	-1.21521200
C	-12.45954200	-0.96503800	1.23716500
H	-13.38305700	-1.29702300	0.75474100
H	-12.70449900	-0.62004400	2.24487900
H	-11.80594600	-1.83501000	1.32916400
C	-12.89255100	3.82928300	-0.24227800
H	-12.93615000	4.13776700	0.80686900
H	-13.90589800	3.61737400	-0.58356500
H	-12.48812000	4.66091700	-0.82109900
O	8.05563000	3.69625200	-2.23653200
H	7.24364700	3.17926400	-2.17782600
O	10.36794400	6.07266900	0.40887800
H	10.89985800	6.28537600	-0.36897000
O	7.75020000	3.58317100	0.65696500

O	7.26277000	1.73998000	-0.56919900
O	6.43283000	-2.52205000	-2.38517700
H	5.63772100	-2.06019600	-2.69092700
O	6.23320200	-1.92878200	-0.14953200
O	11.01953100	-2.09742100	0.27299000
O	9.97505500	-0.24863500	-0.38079800
H	10.90146100	0.01623800	-0.47242500
O	3.83114100	-1.81052000	-2.29218700
H	2.98321900	-1.41415600	-2.53755200
O	1.15839100	-1.38140200	-2.21728200
H	0.72400300	-1.93737000	-2.87994800
O	-0.33812400	-3.59993700	-2.09207700
H	-1.25130100	-3.85661700	-2.23627600
O	-3.61474000	-0.80931500	-2.24675500
H	-4.45723600	-0.53776200	-2.63426000
O	-6.26964900	-0.52495200	-2.45543600
H	-7.10172900	-0.10192000	-2.71209400
O	-8.89735700	-0.02075200	-2.22093100
O	-10.02957000	-1.10588200	-0.61929300
O	-13.87503100	1.12177100	-0.36629300
H	-14.44379000	0.59808500	0.20287400
O	9.49739800	-4.39186900	-0.28010000
H	10.35486100	-3.97880600	-0.12439200
N	10.61130100	4.86497600	-2.11307400
H	9.86657100	5.09727800	-2.76592600
H	11.26718200	4.27495200	-2.61279400

C	-8.60558800	0.40695100	2.34470000
H	-8.62696400	0.11476300	1.28986800
H	-9.28915700	1.25234800	2.46029200
H	-8.99506500	-0.42952500	2.93339600
C	-7.18793100	0.77525000	2.76391000
H	-7.18350000	1.16999300	3.78524600
H	-6.82496900	1.57886500	2.11663600
C	-6.21119300	-0.39578800	2.67583400
H	-6.38152800	-0.94124300	1.73834600
H	-6.40959100	-1.10880100	3.48516100
C	-4.75839300	0.06600000	2.72174800
H	-4.61375100	0.72875400	3.58433200
H	-4.55625400	0.67175600	1.82956000
C	-3.74874100	-1.07383200	2.79139100
H	-3.95482400	-1.79206900	1.98692000
H	-3.87672000	-1.62259600	3.73223800
C	-2.30881700	-0.58264100	2.67239500
H	-2.11942500	0.16960500	3.44872000
H	-2.18442600	-0.06545000	1.71262800
C	-1.27874000	-1.69981300	2.79580000
H	-1.46537200	-2.45767800	2.02283300
H	-1.41145300	-2.21060500	3.75735400
C	0.16399800	-1.21445200	2.68187500
H	0.36536500	-0.48130400	3.47242600
H	0.30231800	-0.67962500	1.73349300
C	1.16364400	-2.36151400	2.79042800

H	0.95289000	-3.10055200	2.00449900
H	1.00155300	-2.88345800	3.74173000
C	2.62531500	-1.93489200	2.70127100
H	2.84100600	-1.17194700	3.45514400
H	2.82126400	-1.46675900	1.72730400
C	3.56574200	-3.11894000	2.91366400
H	3.35351600	-3.90095400	2.17326300
H	3.37873600	-3.55570200	3.90080100
C	5.03260500	-2.75242100	2.80985200
H	5.64864900	-3.64427200	2.98745900
H	5.25360200	-2.37637100	1.80544000
O	5.30974900	-1.75187600	3.78634500
H	6.25969900	-1.62992300	3.83192700

AmB-Dod10

0 1

C	13.16893000	0.09147600	1.17749900
H	13.15554300	0.27019500	0.09740000
H	13.73608200	-0.82602800	1.35382000
H	13.71499600	0.91914200	1.64096700
C	11.75213600	-0.01178200	1.73289000
H	11.79184700	-0.21326200	2.81052300
H	11.25532000	-0.88306000	1.28815700
C	10.92811000	1.25272100	1.48947500

H	10.84218800	1.43046800	0.40808100
H	11.47199800	2.12024300	1.88495700
C	9.53231100	1.20950800	2.10516600
H	9.61865400	1.10078500	3.19490900
H	9.00779800	0.31630100	1.74793000
C	8.66732100	2.42315200	1.77388600
H	8.63627000	2.51903000	0.68177100
H	9.13793400	3.33673900	2.15845200
C	7.24944000	2.29146300	2.32197700
H	7.29293600	2.14699600	3.40996600
H	6.79730800	1.37789500	1.91138100
C	6.32988100	3.46522900	1.99753100
H	6.27056600	3.57727900	0.90934500
H	6.75818800	4.39811200	2.38631800
C	4.91697900	3.26154600	2.54202800
H	4.92031500	3.34292100	3.63695700
H	4.61197400	2.23435900	2.31343300
C	3.88283700	4.21274300	1.94406600
H	4.00911200	4.20763700	0.85647500
H	4.06561500	5.24083100	2.28152500
C	2.44621500	3.80296300	2.26028400
H	2.27348600	3.87539200	3.34210000
H	2.31785300	2.74874700	1.98525500
C	1.39704900	4.62199200	1.51207000
H	1.64352100	4.62768700	0.44224700
H	1.41607300	5.66394400	1.85128500

C	-0.01287300	4.06678000	1.67012200
H	-0.73049400	4.67586100	1.11298200
H	-0.04920300	3.05443800	1.24085000
O	-0.46415300	4.06768500	3.01426900
H	0.08447100	3.44734500	3.50684500
C	8.75149600	-2.48380400	1.66647300
H	9.17373900	-1.62034800	1.14157400
C	9.18745600	-3.71333600	0.87344400
H	8.80961200	-4.61401100	1.38073900
C	8.60002400	-3.68019300	-0.53423100
H	9.00477000	-2.79954600	-1.05771900
C	6.77792900	-2.38159900	0.20235200
H	7.22673400	-1.52083000	-0.31615300
C	7.23811100	-2.33154100	1.65339900
H	6.95519700	-1.34670500	2.06093400
C	8.90680300	-4.92779700	-1.32880000
H	8.46045600	-5.79873700	-0.84139600
H	9.98749700	-5.06882600	-1.38394800
H	8.50015500	-4.84476700	-2.33867300
C	4.70629500	-2.36336200	-1.05334300
H	4.70232000	-3.40346000	-1.39707700
C	3.31518800	-1.90724700	-0.74249200
H	3.23081200	-0.91373200	-0.30984500
C	2.21073700	-2.62965300	-0.95222500
H	2.28585800	-3.62795500	-1.38236200
C	0.88917000	-2.14594500	-0.60353000

H	0.84447600	-1.15169500	-0.16512800
C	-0.25964300	-2.83212300	-0.75125900
H	-0.24057000	-3.83201400	-1.18386700
C	-1.53510400	-2.29866600	-0.33441300
H	-1.51270800	-1.29558500	0.08716100
C	-2.71903100	-2.93610100	-0.42928900
H	-2.76273500	-3.93942600	-0.85187200
C	-3.95624200	-2.34751500	0.02201400
H	-3.90116400	-1.33577400	0.42359300
C	-5.16076300	-2.95213700	-0.02466200
H	-5.23830600	-3.95800900	-0.43599800
C	-6.37164300	-2.34471100	0.46895000
H	-6.28290900	-1.34551300	0.89477600
C	-7.58695100	-2.92760300	0.43921600
H	-7.69153900	-3.91820400	-0.00207200
C	-8.77746800	-2.31235100	0.97420900
H	-8.64972000	-1.35099400	1.46879200
C	-10.01676200	-2.82996900	0.89197500
H	-10.16934700	-3.77875200	0.37873800
C	5.40275000	-1.50898500	-2.12203700
H	6.37003400	-1.95694100	-2.35473900
H	4.80593800	-1.54569600	-3.03721700
C	-11.18644400	-2.15844300	1.42414100
H	-10.98887400	-1.26793200	2.01740600
C	-12.45280900	-2.51992700	1.19142700
H	-12.65020300	-3.40322600	0.58168900

C	-13.66014600	-1.76576600	1.67093200
H	-13.33960600	-0.99408700	2.38004800
C	-14.36512200	-1.04649700	0.50464500
H	-14.81347700	-1.82413700	-0.13585900
C	-13.43871000	-0.21430400	-0.40133100
H	-12.68929900	-0.91380400	-0.78433200
C	-12.64487900	0.85254400	0.36110100
H	-12.22738300	0.42459500	1.26831700
C	-10.29320400	1.17876000	0.01215300
C	-9.23644400	1.55011900	-0.99762000
H	-9.61149100	1.35745100	-2.00445200
H	-9.06339800	2.62869100	-0.90564100
C	-7.91719900	0.81447400	-0.74951900
H	-8.08832500	-0.26822400	-0.83456200
C	-6.85487700	1.23586500	-1.75461800
H	-7.21155500	0.99903200	-2.76272900
H	-6.72018500	2.32259400	-1.69571700
C	-5.49775800	0.56980500	-1.54158200
H	-5.62664200	-0.52065400	-1.50746800
C	-4.52601700	0.91531600	-2.66493900
H	-4.55729300	1.99776000	-2.84009700
H	-4.89588800	0.43950900	-3.57819500
C	-3.08051200	0.47424600	-2.38358100
H	-3.06439600	-0.32123000	-1.63458400
H	-2.63618000	0.04753800	-3.28818700
C	-2.16317800	1.61080600	-1.93530100

H	-2.04598700	2.31126000	-2.77380800
C	-0.76162900	1.08219700	-1.53653100
H	-0.57817300	0.11325700	-2.01519200
C	0.36387200	2.04289300	-1.90553200
H	0.18713900	3.01091700	-1.42116100
H	0.34504500	2.20641700	-2.98909700
C	1.73739500	1.51067300	-1.51156200
H	1.88759700	0.52460400	-1.97151400
C	2.86455500	2.42751300	-1.96912800
H	2.71999800	3.42764500	-1.54834600
H	2.83064300	2.50684200	-3.05929200
C	4.25208000	1.93896100	-1.55819600
C	5.37969700	2.78986700	-2.13478300
H	5.34419000	3.79425700	-1.70735100
H	5.26092900	2.86976100	-3.21933800
C	6.72307800	2.15408300	-1.82684600
H	6.84936900	2.12305500	-0.73950900
C	6.74360300	0.71096300	-2.34922100
H	6.59869200	0.70883700	-3.43371200
C	5.55907900	-0.05214900	-1.70744100
H	5.66905000	0.00911900	-0.61921400
C	8.06172200	0.02170700	-2.10345200
C	-13.37672800	2.13586300	0.69685600
H	-12.73279600	2.78984600	1.28933500
H	-14.27146100	1.89461800	1.27242600
H	-13.67069700	2.67085700	-0.20871000

C	-14.19732000	0.36082200	-1.59626800
H	-15.00959800	1.02388900	-1.28374300
H	-14.62925600	-0.44084200	-2.20229700
H	-13.52867300	0.94244200	-2.23486600
C	-14.64558000	-2.69053400	2.39092100
H	-14.97142300	-3.49951500	1.72762100
H	-15.52765000	-2.13297700	2.70864000
H	-14.17766000	-3.14544200	3.26688000
O	6.67403700	-3.37472100	2.41411600
H	5.75748600	-3.46450800	2.11805900
O	10.59586800	-3.71953100	0.77927300
H	10.89395500	-3.48605000	1.67367700
O	7.17761100	-3.56389100	-0.44180500
O	5.39286600	-2.30635400	0.21465300
O	4.41019000	1.94225900	-0.16668000
H	3.56836900	1.62939800	0.21361400
O	4.35826800	0.61403900	-2.07177600
O	8.49771900	-0.90497200	-2.74168900
O	8.73511300	0.51937700	-1.03493700
H	9.54701500	-0.00650700	-0.94428800
O	1.83315900	1.36782400	-0.08966100
H	0.95205800	1.10115500	0.23673900
O	-0.76855200	0.84186000	-0.12717600
H	-1.44959900	1.47083000	0.19594300
O	-2.66034800	2.34455900	-0.82625200
H	-3.52109500	1.96462900	-0.54405300

O	-4.91013800	0.98987400	-0.30687700
H	-5.63036400	1.09279700	0.34215700
O	-7.41753400	1.10993200	0.55188100
H	-8.15983500	1.00760200	1.17432100
O	-10.03616900	0.91260700	1.17577700
O	-11.51417200	1.19591900	-0.50121300
O	-15.39566600	-0.25504800	1.08805000
H	-16.03342600	-0.02467400	0.40411200
O	7.73456600	2.94491700	-2.42302100
H	8.55890800	2.74955500	-1.96254500
N	9.36923000	-2.54225500	2.98463800
H	8.81455600	-3.15768400	3.57491200
H	9.36871100	-1.62469100	3.41904000

AmB-Dod11

0 1

C	-7.00133500	4.08803400	-3.08984900
H	-7.39099200	4.92851500	-2.50733300
H	-7.78668700	3.32792400	-3.13772900
H	-6.82284100	4.44950700	-4.10761700
C	-5.72367900	3.53188300	-2.46893300
H	-5.36510000	2.68728100	-3.07279100
H	-5.94697700	3.13277600	-1.47318700
C	-4.60726100	4.56732000	-2.34917300

H	-4.92478700	5.36598800	-1.66586800
H	-4.44280800	5.04919100	-3.32220300
C	-3.29574500	3.96475300	-1.85464900
H	-2.94400800	3.23052700	-2.59335900
H	-3.47868200	3.40056500	-0.93156700
C	-2.19177100	4.98767600	-1.60002500
H	-2.49675500	5.65083200	-0.78027000
H	-2.06971500	5.62998000	-2.48213300
C	-0.85615700	4.33284400	-1.25907500
H	-0.51172800	3.75526300	-2.12857900
H	-1.00461300	3.60125300	-0.45493200
C	0.23695500	5.31163300	-0.84125600
H	-0.07947600	5.83572500	0.07002400
H	0.35919100	6.08570600	-1.61033700
C	1.57455700	4.62086000	-0.59133600
H	1.93324400	4.18097800	-1.53230400
H	1.42308700	3.77646800	0.09243700
C	2.65460500	5.53334000	-0.01917900
H	2.30033200	5.95481900	0.93111600
H	2.81481500	6.38797800	-0.68953700
C	3.97450100	4.80330800	0.20921600
H	4.37627000	4.45197600	-0.74721700
H	3.78960300	3.90210000	0.80781900
C	5.03339600	5.65291800	0.90268300
H	4.65503600	6.00272000	1.87122900
H	5.25119800	6.54647600	0.30568400

C	6.33035500	4.90090100	1.13512400
H	7.01587800	5.51214200	1.74138600
H	6.12164200	3.97842600	1.69819400
O	6.91019000	4.59736700	-0.12385400
H	7.59224100	3.93240100	0.02602100
C	-9.79427800	2.25057600	2.00791800
H	-10.50221200	2.25237800	1.16949400
C	-9.03027900	3.57046100	1.95676900
H	-8.32266100	3.58983200	2.79994300
C	-8.21591000	3.67296100	0.67197000
H	-8.91635400	3.66321200	-0.18027500
C	-7.97471500	1.30217400	0.58760200
H	-8.61005100	1.20857400	-0.31150700
C	-8.83166500	1.08312000	1.83272000
H	-9.38330900	0.14461700	1.68491200
C	-7.37399800	4.92935000	0.63999500
H	-6.65220200	4.90539200	1.46068400
H	-8.01753100	5.80214700	0.76149900
H	-6.82555600	5.01014600	-0.29882500
C	-6.30377300	0.06479800	-0.61522900
H	-6.30803800	0.97336000	-1.22528600
C	-4.89517100	-0.26777500	-0.24021700
H	-4.76629000	-1.03825400	0.51543600
C	-3.82773100	0.34056700	-0.76494000
H	-3.96154500	1.10586300	-1.52724400
C	-2.47392700	0.06055900	-0.33412100

H	-2.35687900	-0.67593100	0.45674700
C	-1.37461500	0.67059900	-0.81612500
H	-1.47421400	1.41352700	-1.60518800
C	-0.05083700	0.40289700	-0.30825700
H	0.01494400	-0.34962000	0.47562100
C	1.07607800	1.00798300	-0.73545800
H	1.01831500	1.76147500	-1.51869300
C	2.37770400	0.71313200	-0.19084400
H	2.42761700	-0.06276000	0.57289700
C	3.52281400	1.31458500	-0.57473900
H	3.48996400	2.08296100	-1.34495000
C	4.81000300	1.01128200	-0.00200900
H	4.83491300	0.25806600	0.78573300
C	5.96721400	1.58833700	-0.38572100
H	5.95885800	2.34102200	-1.17042300
C	7.23902800	1.27844700	0.22138300
H	7.21974000	0.63325300	1.09839900
C	8.43856100	1.70458000	-0.22404200
H	8.49240200	2.29967200	-1.13631500
C	-7.05450000	-1.04421600	-1.36043500
H	-8.05249800	-0.66794800	-1.59515300
H	-6.54618200	-1.26496800	-2.30408200
C	9.68773500	1.34741200	0.42137100
H	9.59206500	0.81933300	1.36796900
C	10.90686100	1.57026200	-0.08045300
H	11.00316900	2.08571700	-1.03747900

C	12.18829100	1.11916000	0.55994700
H	11.96871700	0.73658600	1.56332400
C	12.83187300	-0.03399100	-0.23415900
H	13.18991700	0.38575900	-1.18891300
C	11.87686400	-1.18716300	-0.59463200
H	11.07091100	-0.73392100	-1.18014100
C	11.18851100	-1.81090700	0.62451300
H	10.81188400	-1.02705000	1.27574600
C	8.83329000	-2.28368400	0.63289500
C	7.73809900	-3.07204100	-0.04021800
H	8.03867900	-3.30745900	-1.06300700
H	7.63552800	-4.01905400	0.50236900
C	6.39590300	-2.33843500	-0.01309200
H	6.49845700	-1.37748800	-0.53771200
C	5.30690000	-3.16283500	-0.68379600
H	5.59456900	-3.33944000	-1.72573500
H	5.24070600	-4.13844600	-0.18747600
C	3.92565200	-2.51367300	-0.65500800
H	3.99185700	-1.49244000	-1.05482500
C	2.92325700	-3.30496500	-1.48855500
H	3.00259100	-4.36631800	-1.22283200
H	3.22907600	-3.22046000	-2.53578400
C	1.47070000	-2.83076900	-1.31919200
H	1.44791700	-1.80708800	-0.93789600
H	0.96984300	-2.80193600	-2.29190300
C	0.62862700	-3.72801100	-0.41468500

H	0.52805200	-4.70859900	-0.90007000
C	-0.79490300	-3.14269500	-0.19289900
H	-1.00709700	-2.38868000	-0.95961200
C	-1.88518800	-4.20744100	-0.22996000
H	-1.68134900	-4.96276400	0.53898800
H	-1.85102500	-4.70974700	-1.20390400
C	-3.28277800	-3.63161200	-0.01817400
H	-3.45433500	-2.82458300	-0.74338400
C	-4.36437900	-4.68668200	-0.21166600
H	-4.17729600	-5.53406000	0.45532000
H	-4.31643200	-5.04683400	-1.24336700
C	-5.77889500	-4.17858400	0.06838500
C	-6.84679300	-5.18081200	-0.34596900
H	-6.77760400	-6.08240700	0.26617600
H	-6.68912900	-5.46006500	-1.39157900
C	-8.24616400	-4.60297000	-0.22164800
H	-8.46295500	-4.42904300	0.84258800
C	-8.32782400	-3.23739700	-0.95749700
H	-8.28282300	-3.45436900	-2.03161100
C	-7.16065700	-2.32704900	-0.54780100
H	-7.28592300	-2.07343800	0.50863600
C	-9.64975700	-2.57375300	-0.66449000
C	12.00381200	-2.79750800	1.43460100
H	11.43414500	-3.12799100	2.30611700
H	12.92181600	-2.31081600	1.76683800
H	12.26400200	-3.67572200	0.83982500

C	12.56668000	-2.22714700	-1.47601000
H	13.43271800	-2.67459800	-0.97910000
H	12.90996300	-1.77463600	-2.41082600
H	11.87908700	-3.03782300	-1.72704400
C	13.17956200	2.27794500	0.70023700
H	13.40661500	2.71238800	-0.27961300
H	14.11277400	1.92804700	1.14364100
H	12.76247700	3.06899600	1.32751800
O	-8.02363800	1.01931000	2.99096600
H	-7.22307400	0.53719700	2.74161900
O	-9.93560100	4.64897400	2.01437400
H	-10.58969500	4.37205400	2.67636300
O	-7.33005800	2.55854400	0.58446500
O	-6.98880600	0.32721700	0.62035100
O	-5.97012300	-3.89527600	1.42421200
H	-5.15892500	-3.44881400	1.72837700
O	-5.92934200	-3.01012200	-0.73180800
O	-9.82567900	-1.51737300	-0.10574000
O	-10.70403200	-3.31968500	-1.08195700
H	-11.50384200	-2.83436500	-0.82144100
O	-3.41519300	-3.09859800	1.30217500
H	-2.54925600	-2.72611400	1.55079400
O	-0.80984800	-2.46064700	1.06123600
H	-0.08299900	-2.89745100	1.55418700
O	1.20719600	-3.94391600	0.86448100
H	2.04574500	-3.43860800	0.93058200

O	3.42662700	-2.43943700	0.68353500
H	4.18018700	-2.23804100	1.26734500
O	5.98860400	-2.09686900	1.33153000
H	6.74891700	-1.69777600	1.79005200
O	8.63327700	-1.53146800	1.57344200
O	10.02240900	-2.51808900	0.09753900
O	13.94251600	-0.47801600	0.53916100
H	14.53652000	-0.97804200	-0.03070900
O	-9.11802800	-5.56454200	-0.77461000
H	-10.00669000	-5.18795300	-0.79849900
N	-10.56541800	2.23148300	3.24809000
H	-9.93162300	1.99336400	4.00797400
H	-11.27350800	1.50484000	3.21823600

AmB-Dod12

0 1

C	10.91969000	-3.89000600	0.91734500
H	11.63828500	-3.43745700	0.22254100
C	10.39414100	-5.15816100	0.24662600
H	9.70334600	-5.65915500	0.94010400
C	9.61921700	-4.80753300	-1.01721900
H	10.30821700	-4.29232500	-1.70660300
C	8.98029700	-2.71258400	-0.13032700
H	9.60552100	-2.18777200	-0.87116900

C	9.77411600	-2.91132600	1.16019200
H	10.16164200	-1.92923700	1.46887400
C	9.02958700	-6.02762900	-1.68903000
H	8.31312800	-6.50183900	-1.01487000
H	9.82486900	-6.73670500	-1.91921300
H	8.51267300	-5.74640600	-2.60731000
C	6.98782400	-1.60737900	-0.86800300
H	6.91240900	-2.45971400	-1.54860600
C	5.64559200	-1.35270900	-0.25065700
H	5.60596000	-0.63540000	0.56669800
C	4.53305600	-1.96848200	-0.66196600
H	4.59091200	-2.67547600	-1.48828100
C	3.22550400	-1.77951100	-0.06085800
H	3.16242800	-1.12923500	0.80977700
C	2.11231900	-2.38879700	-0.51159400
H	2.18602200	-3.03500800	-1.38527500
C	0.80481900	-2.25886200	0.09125400
H	0.73047700	-1.64810400	0.99006300
C	-0.29454000	-2.85516500	-0.41547300
H	-0.19240500	-3.44955300	-1.32261200
C	-1.61905200	-2.77052000	0.15264900
H	-1.74969200	-2.21191200	1.07882400
C	-2.69626200	-3.34078100	-0.42815700
H	-2.56363400	-3.87727800	-1.36661600
C	-4.03744100	-3.29196700	0.10429900
H	-4.17728600	-2.81081000	1.07175400

C	-5.10591500	-3.79728400	-0.54707500
H	-4.95623600	-4.24969700	-1.52629300
C	-6.46029700	-3.77528500	-0.04233700
H	-6.60699800	-3.42597600	0.97825600
C	-7.53412600	-4.14017200	-0.76870900
H	-7.39047000	-4.46297700	-1.79855300
C	7.57580100	-0.40800700	-1.62129600
H	8.55352900	-0.69340500	-2.01740100
H	6.93317500	-0.16797000	-2.47305100
C	-8.89863700	-4.09322800	-0.27341300
H	-9.01802200	-3.89827800	0.79029300
C	-9.98456800	-4.22725200	-1.04467700
H	-9.85305700	-4.41432000	-2.11203800
C	-11.40357900	-4.11482800	-0.56458300
H	-11.40617400	-4.01031000	0.52564100
C	-12.09232700	-2.86708900	-1.15046300
H	-12.25153100	-3.05326400	-2.22438700
C	-11.27535100	-1.56671700	-1.04221600
H	-10.35094500	-1.74527700	-1.60025200
C	-10.83662300	-1.26067800	0.39328100
H	-10.47440300	-2.16772300	0.86829200
C	-8.57971300	-0.67644400	0.97044400
C	-7.46584700	0.31248400	0.74917800
H	-7.60185800	0.80072800	-0.21647500
H	-7.56614400	1.08870200	1.51593300
C	-6.08482000	-0.33632600	0.83905600

H	-5.99315500	-1.09458600	0.04862200
C	-4.99475700	0.71196100	0.66378000
H	-5.15627000	1.22725100	-0.28883100
H	-5.09212200	1.45468600	1.46537900
C	-3.57682000	0.14443500	0.68200700
H	-3.49592200	-0.63357100	-0.09322500
C	-2.55165300	1.23518500	0.39304500
H	-2.71937900	2.02311600	1.13983400
H	-2.75011700	1.67386500	-0.59138600
C	-1.12027800	0.70919800	0.45797400
H	-0.99034000	0.14275800	1.38355100
H	-0.94791100	0.01863500	-0.37445500
C	-0.06847400	1.80947700	0.41344800
H	-0.18346500	2.41019000	-0.50052700
C	1.35689100	1.25954500	0.45747500
H	1.47099100	0.48372400	-0.30656800
C	2.39599400	2.35840800	0.24515800
H	2.26357600	3.13108600	1.01064000
H	2.20843300	2.82609100	-0.72811500
C	3.83779900	1.85608300	0.26871000
H	3.96596500	1.06445700	-0.48191300
C	4.80728300	2.99129600	-0.04044100
H	4.64179200	3.81471400	0.66165400
H	4.60640000	3.35545500	-1.05186700
C	6.27953800	2.59412800	0.04795000
C	7.19923600	3.67904000	-0.49506000

H	7.13490500	4.57367500	0.12731900
H	6.89656900	3.93607500	-1.51362400
C	8.64132600	3.20569900	-0.53609300
H	8.99946600	3.06041200	0.49247100
C	8.73471800	1.84412600	-1.27739700
H	8.55087600	2.05349800	-2.33802000
C	7.69791400	0.83965200	-0.75437000
H	7.96004200	0.55714200	0.26998100
C	10.16714600	1.38492200	-1.15513300
C	-11.86018900	-0.58757400	1.28773000
H	-11.45512800	-0.47453100	2.29495600
H	-12.76021500	-1.20179800	1.33082000
H	-12.12590800	0.40184300	0.90845900
C	-12.00741200	-0.39474000	-1.69453600
H	-12.96354100	-0.19156400	-1.20496300
H	-12.20042500	-0.60173900	-2.75010400
H	-11.41109700	0.51930300	-1.63896300
C	-12.20834000	-5.36778200	-0.92704600
H	-12.20145900	-5.52501900	-2.01005400
H	-13.24228800	-5.26108200	-0.59893600
H	-11.77411300	-6.25064200	-0.45596900
O	8.95523100	-3.46062200	2.16783800
H	8.08265700	-3.06298300	2.06451600
O	11.47170900	-5.99585200	-0.10179500
H	12.05546500	-5.98029700	0.66778800
O	8.54624800	-3.93674800	-0.67437700

O	7.86093200	-1.96734700	0.21647300
O	6.68036700	2.33185100	1.36328000
H	5.96507100	1.83008300	1.78220600
O	6.40914600	1.43670700	-0.76730600
O	11.11414200	2.01714900	-1.55043200
O	10.33474000	0.22129500	-0.50025500
H	11.28985300	0.07485700	-0.44379100
O	4.18404900	1.32822100	1.55213800
H	3.40640800	0.87068700	1.90072600
O	1.55718700	0.60442500	1.71625400
H	1.10275400	1.15214700	2.37167400
O	-0.15125400	2.65357900	1.56098700
H	-1.05814600	2.94963000	1.66818200
O	-3.24187900	-0.40995100	1.94478300
H	-4.04549300	-0.79259100	2.32010900
O	-5.88611800	-0.95543300	2.10618500
H	-6.67001700	-1.48560400	2.30306700
O	-8.49851400	-1.63357300	1.70432600
O	-9.68435300	-0.36865100	0.28038700
O	-13.35047800	-2.76241400	-0.49447100
H	-13.92943200	-2.20640200	-1.02114000
O	9.38068200	4.20666200	-1.19557200
H	10.26289400	3.85965700	-1.37216000
N	11.64483200	-4.29320600	2.12060300
H	10.96098500	-4.48963600	2.84783300
H	12.22673300	-3.53630600	2.46224100

C	3.88351900	7.12727700	-0.08800400
H	3.82447600	7.59819600	-1.07265500
H	4.87760700	6.68615500	0.01390500
H	3.78673700	7.91447300	0.66401400
C	2.78747300	6.07900600	0.07912100
H	2.87820300	5.60329400	1.06258100
H	2.92700500	5.28162400	-0.65947600
C	1.37896600	6.64922000	-0.06997900
H	1.27895200	7.11576100	-1.05784700
H	1.22883800	7.44839800	0.66590600
C	0.29966400	5.58444800	0.10571900
H	0.38596900	5.13482300	1.10081200
H	0.48504100	4.76814300	-0.60524700
C	-1.11785500	6.10952300	-0.10730300
H	-1.20148500	6.51799000	-1.12167600
H	-1.31048900	6.94405400	0.57720400
C	-2.18951600	5.03764000	0.08562200
H	-2.19435400	4.71592600	1.13595800
H	-1.92863800	4.15543900	-0.51457300
C	-3.59315100	5.50207400	-0.29668900
H	-3.57266600	5.89301000	-1.32095200
H	-3.89007100	6.33876200	0.34656200
C	-4.63549300	4.39000300	-0.20959800
H	-4.65388300	3.97935300	0.80821700
H	-4.32927200	3.56511200	-0.86569500
C	-6.04069400	4.84266700	-0.59976700

H	-6.00892500	5.29624200	-1.59807000
H	-6.37572400	5.62895700	0.08668500
C	-7.05244500	3.69978100	-0.59419900
H	-7.09419800	3.24928100	0.40315200
H	-6.70771500	2.91541100	-1.28222800
C	-8.45862100	4.13117500	-1.00133200
H	-8.43938200	4.56054900	-2.00894000
H	-8.81832000	4.91238700	-0.32353000
C	-9.45335800	2.98359500	-0.97430900
H	-10.41931400	3.31473100	-1.37625000
H	-9.08786100	2.16580600	-1.61365100
O	-9.59783300	2.54471200	0.36602700
H	-9.92366400	1.63959700	0.35382300

AmB-Dod13

0 1

C	9.95267500	-4.71177600	1.18332700
H	10.71426200	-4.35528400	0.47851200
C	9.34521800	-5.97577000	0.57657500
H	8.60717700	-6.38231400	1.28276700
C	8.61962300	-5.64523300	-0.72164900
H	9.35525000	-5.22152000	-1.42473100
C	8.12251100	-3.46150300	0.02834900
H	8.79615300	-3.02939100	-0.73010800

C	8.88039300	-3.63753600	1.34377400
H	9.33585800	-2.66892400	1.59766300
C	7.95528700	-6.85749600	-1.33527600
H	7.19651400	-7.24153600	-0.65007900
H	8.70219700	-7.63231400	-1.50887100
H	7.47459200	-6.59424700	-2.27820600
C	6.23176400	-2.25098000	-0.80953700
H	6.10030700	-3.13062500	-1.44582700
C	4.90519800	-1.85903200	-0.23133800
H	4.91466000	-1.10888400	0.55698000
C	3.74927900	-2.38768400	-0.64277800
H	3.75595300	-3.12954000	-1.44009200
C	2.45484500	-2.05639600	-0.07563500
H	2.43814400	-1.36490500	0.76480700
C	1.29908700	-2.58534200	-0.51919200
H	1.32833100	-3.27408500	-1.36235600
C	0.00106700	-2.31836600	0.05773500
H	-0.03335100	-1.65853300	0.92344600
C	-1.13730100	-2.85387400	-0.42888400
H	-1.07615000	-3.50423800	-1.30056500
C	-2.45293800	-2.64123400	0.12452500
H	-2.54652500	-2.01492800	1.01099500
C	-3.56426800	-3.18011200	-0.41956400
H	-3.46702000	-3.78836000	-1.31783400
C	-4.89762400	-3.01577000	0.10676100
H	-5.00886100	-2.45251600	1.03250100

C	-5.99145900	-3.52006500	-0.50095200
H	-5.86684700	-4.06011300	-1.43826200
C	-7.34061000	-3.39016600	-0.00185300
H	-7.47065500	-2.93036600	0.97622300
C	-8.42778900	-3.78925600	-0.68888800
H	-8.29662500	-4.22832300	-1.67666500
C	6.92133300	-1.13992500	-1.61077600
H	7.88647900	-1.51299200	-1.96202300
H	6.31750700	-0.90361900	-2.49143100
C	-9.78865800	-3.62948600	-0.21094200
H	-9.90139400	-3.29273700	0.81738700
C	-10.87422700	-3.81684500	-0.97122000
H	-10.74265300	-4.14426300	-2.00414600
C	-12.28922300	-3.56685900	-0.53574500
H	-12.30054300	-3.33735000	0.53505300
C	-12.88153500	-2.35291100	-1.27672300
H	-13.02524000	-2.64852400	-2.32838200
C	-11.98586900	-1.10044400	-1.28996400
H	-11.06693600	-1.39468700	-1.80658100
C	-11.54506800	-0.66388300	0.11070200
H	-11.24325300	-1.53320100	0.68712500
C	-9.29889800	-0.10475900	0.69396700
C	-8.13452300	0.81585700	0.41896500
H	-8.24406200	1.25007800	-0.57544000
H	-8.18853700	1.62915600	1.15197600
C	-6.78438200	0.10639600	0.56581100

H	-6.72618400	-0.70861900	-0.16999500
C	-5.63113500	1.07771400	0.33871300
H	-5.74502200	1.52976300	-0.65283100
H	-5.69847200	1.87893500	1.08567600
C	-4.24714100	0.43229900	0.42747400
H	-4.19826400	-0.39636300	-0.29655900
C	-3.14558500	1.43794900	0.10330500
H	-3.26665000	2.26342500	0.81883800
H	-3.30026100	1.84967000	-0.90097700
C	-1.75615800	0.81160000	0.21312800
H	-1.67480600	0.29235300	1.17142500
H	-1.63329200	0.06032300	-0.57441200
C	-0.61585800	1.81703200	0.10974300
H	-0.67145600	2.35708200	-0.84708900
C	0.75740000	1.15128700	0.22057600
H	0.81475200	0.31687200	-0.48600100
C	1.89707500	2.13523400	-0.04176200
H	1.82103800	2.97304700	0.66199600
H	1.77468000	2.54185900	-1.05225400
C	3.28819600	1.50985800	0.06039300
H	3.36514500	0.66264600	-0.63497800
C	4.36177900	2.53328800	-0.29104100
H	4.25303100	3.41959700	0.34492800
H	4.21943100	2.83690600	-1.33242200
C	5.79331300	2.02296000	-0.13075100
C	6.80950800	3.01007500	-0.68256100

H	6.77828200	3.93288300	-0.10144600
H	6.56656800	3.23436900	-1.72587300
C	8.21075300	2.43413800	-0.64532600
H	8.51626500	2.29855400	0.40143100
C	8.23014000	1.04383400	-1.33633300
H	8.09459600	1.22338500	-2.40962200
C	7.10484000	0.14116000	-0.80627400
H	7.31408200	-0.10292900	0.24000200
C	9.61981400	0.48737300	-1.14864000
C	-12.54262900	0.15336800	0.90945200
H	-12.14784800	0.34381900	1.90915500
H	-13.47749600	-0.40164700	0.99412200
H	-12.73829900	1.11115000	0.42403700
C	-12.63320300	0.03366600	-2.08317600
H	-13.58443900	0.34342600	-1.64216600
H	-12.81882200	-0.27623300	-3.11475200
H	-11.98255100	0.90993500	-2.10652900
C	-13.16847000	-4.79906300	-0.77193100
H	-13.15027300	-5.08342800	-1.82875900
H	-14.19946500	-4.58682200	-0.48884100
H	-12.80558500	-5.64665800	-0.18879100
O	8.00832500	-4.05910300	2.36833000
H	7.16976500	-3.60532500	2.22396200
O	10.36378600	-6.90903300	0.30126700
H	10.93143100	-6.89159600	1.08285000
O	7.60743000	-4.68178200	-0.45012100

O	7.05666300	-2.61762000	0.30961000
O	6.12931500	1.78653600	1.20687900
H	5.35520700	1.38488300	1.62828600
O	5.86111600	0.82178100	-0.89225600
O	10.62194700	1.03468200	-1.53578200
O	9.68376100	-0.65791600	-0.44478100
H	10.62379400	-0.86795900	-0.35024500
O	3.55278300	1.03917100	1.38423900
H	2.72896000	0.67618700	1.73810100
O	0.87589400	0.57100000	1.52545800
H	0.46923400	1.20738500	2.13066700
O	-0.63666600	2.74905400	1.19069400
H	-1.53180700	3.07801300	1.30919100
O	-3.97086700	-0.05996600	1.72905500
H	-4.80289800	-0.36976500	2.11012800
O	-6.63592100	-0.43337100	1.87466900
H	-7.46442600	-0.87552700	2.10872100
O	-9.27510600	-0.96618500	1.54715900
O	-10.34720200	0.14741900	-0.07832600
O	-14.14844300	-2.10070200	-0.67912900
H	-14.67140900	-1.56582900	-1.28069100
O	9.04957900	3.36027600	-1.30112900
H	9.90522400	2.93880100	-1.44546700
N	10.62562800	-5.09396300	2.42322300
H	9.91841100	-5.18356400	3.14908400
H	11.26604000	-4.36814900	2.72521400

C	-7.36309100	4.50765400	0.48025700
H	-7.62126400	5.32106900	-0.20239900
H	-8.18818100	4.38783500	1.18580500
H	-7.28812800	3.59254100	-0.11400600
C	-6.04390000	4.79772000	1.19129800
H	-5.84321700	4.02477500	1.94209700
H	-6.12102100	5.74233200	1.73993400
C	-4.87152900	4.87329000	0.21716200
H	-5.09976800	5.60573900	-0.56672100
H	-4.76181400	3.90656100	-0.29135500
C	-3.55025300	5.25282300	0.87942900
H	-3.34177000	4.55815000	1.70586000
H	-3.64776500	6.24212400	1.34191400
C	-2.37878400	5.26439600	-0.09957400
H	-2.64677400	5.87161500	-0.97239300
H	-2.21675200	4.24864100	-0.48618400
C	-1.07936900	5.79807800	0.50019700
H	-0.86356000	5.28720800	1.44491200
H	-1.20473900	6.86018800	0.74166500
C	0.10942300	5.61925600	-0.43820100
H	-0.09187000	6.13455700	-1.38573400
H	0.21118300	4.55290400	-0.67426800
C	1.42587900	6.12241100	0.14524200
H	1.64206000	5.57432400	1.07080700
H	1.32468800	7.17764200	0.42688800
C	2.59761400	5.96414500	-0.81963000

H	2.37682400	6.49939500	-1.75144600
H	2.69486300	4.90476000	-1.09072100
C	3.92366100	6.45739700	-0.24745000
H	4.12222400	5.96466100	0.70887800
H	3.85737500	7.53146400	-0.03461000
C	5.09334200	6.19043200	-1.19098000
H	4.93255200	6.70384100	-2.14543300
H	5.13548900	5.11903300	-1.40967200
C	6.43451200	6.62811700	-0.62690900
H	7.24225900	6.28737500	-1.28617000
H	6.47403700	7.72404400	-0.56610000
O	6.57485500	6.06196400	0.66981000
H	7.48842900	6.15530700	0.94736600

AmB-Dod14

0 1

C	-6.31899500	5.77983600	1.59150000
H	-7.10874200	5.07644400	1.26606300
C	-6.21531400	6.86373900	0.50385300
H	-5.45087000	7.60580100	0.81403400
C	-5.75791100	6.25057400	-0.82590700
H	-6.49931400	5.48283300	-1.13680900
C	-4.56246600	4.53815400	0.31000400
H	-5.30005300	3.79424200	-0.06901600

C	-5.00401300	4.99370300	1.71117600
H	-5.16540500	4.07991400	2.32197500
C	-5.58310900	7.27512000	-1.93095500
H	-4.81525900	8.01136500	-1.64677700
H	-6.53239200	7.80273600	-2.09848300
H	-5.27438200	6.78301400	-2.86465300
C	-2.58372500	3.57804900	-0.75611000
H	-2.33923900	4.49575300	-1.31761000
C	-1.33106500	2.91859100	-0.27152900
H	-1.46157400	2.05357000	0.38737500
C	-0.08004900	3.33098600	-0.59474300
H	0.04416800	4.20003800	-1.25468700
C	1.11049900	2.69006600	-0.10567100
H	0.96982600	1.83644400	0.56821500
C	2.39479400	3.07026100	-0.40031100
H	2.55863800	3.92121400	-1.07480800
C	3.53979500	2.40633400	0.13697500
H	3.33928300	1.55799700	0.80352000
C	4.84869900	2.72557700	-0.13854000
H	5.06270500	3.55652800	-0.82360500
C	5.95710200	2.01728700	0.41166600
H	5.72928700	1.19945200	1.10847700
C	7.27932600	2.26926200	0.12342000
H	7.51811200	3.07542000	-0.58271900
C	8.36952800	1.54020100	0.68192600
H	8.12479600	0.75080000	1.40547300

C	9.69358500	1.75004900	0.37274900
H	9.94235100	2.52985800	-0.35911400
C	10.77688100	1.00998300	0.93726900
H	10.52518400	0.25206700	1.69103000
C	12.09614700	1.16642800	0.59687600
H	12.36114800	1.91368300	-0.16258000
C	-3.46178900	2.68928600	-1.66010700
H	-4.31551600	3.28687700	-2.01296100
H	-2.87668100	2.42757400	-2.55661800
C	13.16205100	0.37779900	1.15324400
H	12.87586000	-0.34115400	1.93191100
C	14.45960000	0.42510800	0.75627300
H	14.74817700	1.14069300	-0.02728600
C	15.55702800	-0.45201400	1.29067000
H	15.15325700	-1.06981500	2.11315700
C	16.07651300	-1.42458900	0.19920500
H	16.63124500	-0.81611000	-0.54701800
C	14.96833800	-2.17219100	-0.58486900
H	14.34428800	-1.37366000	-1.02428600
C	14.01712700	-2.97128300	0.32409700
H	13.74478100	-2.36635300	1.19734600
C	11.60383900	-2.94260500	0.17322900
C	10.41296500	-3.13538400	-0.74391500
H	10.72133500	-2.97329800	-1.78730000
H	10.09085200	-4.18844500	-0.65134600
C	9.23824700	-2.21656500	-0.36640900

H	9.57013300	-1.16173800	-0.44867300
C	8.03002500	-2.43166800	-1.27820600
H	8.33704400	-2.22386400	-2.31763400
H	7.71614600	-3.48994900	-1.22702700
C	6.82884800	-1.54053000	-0.93437400
H	7.15954400	-0.48279800	-0.90598800
C	5.70389600	-1.67348800	-1.96455800
H	5.49089000	-2.74595400	-2.12843700
H	6.08892900	-1.28087200	-2.92000100
C	4.40488300	-0.92962300	-1.58052200
H	4.62578200	-0.11068000	-0.87694500
H	3.97779600	-0.45041700	-2.47724700
C	3.29996500	-1.82157000	-0.99869700
H	3.00830900	-2.56376500	-1.76830300
C	2.03498400	-0.99352100	-0.61686200
H	2.00492000	-0.06827800	-1.21934300
C	0.72701800	-1.76612400	-0.82600700
H	0.74506000	-2.70008900	-0.23593900
H	0.65871100	-2.04668100	-1.89245900
C	-0.50842200	-0.93825800	-0.45755500
H	-0.46904200	0.02375000	-1.00610600
C	-1.81767000	-1.63980900	-0.82161700
H	-1.88781300	-2.60115000	-0.28626900
H	-1.81486800	-1.84783300	-1.90340100
C	-3.07494000	-0.81778300	-0.49108900
C	-4.35937100	-1.47729300	-1.01117000

H	-4.55565700	-2.39995300	-0.44587600
H	-4.22888200	-1.74162000	-2.07332700
C	-5.55089700	-0.53720300	-0.87702300
H	-5.72123400	-0.32748200	0.19358000
C	-5.24947500	0.79435100	-1.59887200
H	-5.06949000	0.59973500	-2.66985500
C	-3.93574900	1.39410200	-1.00097000
H	-4.09373700	1.55267700	0.08280300
C	-6.39585900	1.77964100	-1.54201100
C	14.48262800	-4.34419700	0.78187700
H	13.73659700	-4.79242200	1.45442200
H	15.43139300	-4.23636100	1.32730600
H	14.63944300	-5.02251800	-0.06963400
C	15.55069100	-3.00041300	-1.73734500
H	16.22355000	-3.79746300	-1.38042100
H	16.12166700	-2.35683500	-2.42563500
H	14.75109900	-3.48468000	-2.31742200
C	16.72078400	0.38783400	1.85121700
H	17.13918400	1.04419100	1.06939000
H	17.52412700	-0.26529800	2.22022600
H	16.37321300	1.02625500	2.67658100
O	-4.03023600	5.83481500	2.32300100
H	-3.16235200	5.46952100	2.06638300
O	-7.48607400	7.48629100	0.32692600
H	-7.82635500	7.58539800	1.24060300
O	-4.47805300	5.61323200	-0.61565100

O	-3.28836500	3.97170100	0.46715500
O	-3.23497600	-0.61456200	0.89874200
H	-2.32947800	-0.48449400	1.26464300
O	-2.88025900	0.44226200	-1.18312600
O	-6.62293800	2.65408000	-2.35775700
O	-7.18750100	1.61523800	-0.42836000
H	-7.87768600	2.30710300	-0.49166800
O	-0.54042000	-0.65892000	0.96227300
H	0.39502500	-0.51382800	1.23688700
O	2.16588700	-0.57085800	0.75930200
H	2.72225900	-1.29238600	1.14806800
O	3.68926200	-2.54256700	0.18011600
H	4.65870100	-2.39678300	0.33683500
O	6.28498700	-1.87618700	0.36214300
H	7.04519900	-2.09692200	0.94718300
O	8.81322900	-2.46963600	0.98817200
H	9.62966900	-2.45750400	1.53757900
O	11.50391300	-2.64668000	1.36332500
O	12.76924800	-3.13916500	-0.46346600
O	16.99415800	-2.31973300	0.85757900
H	17.54135400	-2.74446600	0.17759600
O	-6.69240300	-1.20280600	-1.43926000
H	-7.48124400	-0.74735000	-1.09957700
N	-6.78447900	6.42607700	2.82638100
H	-5.98633000	6.87942000	3.28332500
H	-7.15335900	5.73705200	3.48551600

C	-20.43556800	-7.76509500	0.98941700
H	-20.61214200	-7.75212000	-0.06583500
H	-21.37119400	-7.80326000	1.50715400
H	-19.85230400	-8.62558800	1.24290300
C	-19.67431100	-6.49035100	1.39821500
H	-19.49943100	-6.50240000	2.45375700
H	-20.25672300	-5.62979900	1.14297200
C	-18.32648400	-6.43681600	0.65517000
H	-18.50134400	-6.42514200	-0.40038000
H	-17.74391800	-7.29718100	0.91069600
C	-17.56545600	-5.16181300	1.06355800
H	-17.39066900	-5.17344500	2.11912600
H	-18.14797900	-4.30144100	0.80795800
C	-16.21757700	-5.10834000	0.32060300
H	-16.39237300	-5.09671400	-0.73496300
H	-15.63505100	-5.96869700	0.57620900
C	-15.45655900	-3.83332400	0.72897700
H	-15.28177700	-3.84494700	1.78454300
H	-16.03909000	-2.97295900	0.47336900
C	-14.10868800	-3.77985600	-0.01397600
H	-14.28347600	-3.76822200	-1.06953400
H	-13.52614900	-4.64021200	0.24164100
C	-13.34765700	-2.50484000	0.39440900
H	-13.17287500	-2.51646300	1.44997400
H	-13.93018800	-1.64447500	0.13880000
C	-11.99979200	-2.45136400	-0.34854700

H	-12.17457900	-2.43973200	-1.40411500
H	-11.41726000	-3.31172800	-0.09293900
C	-11.23876000	-1.17634700	0.05983800
H	-11.06397300	-1.18798100	1.11539600
H	-11.82129200	-0.31598300	-0.19577100
C	-9.89089500	-1.12287400	-0.68312800
H	-10.06568200	-1.11124000	-1.73868600
H	-9.30836300	-1.98323800	-0.42751900
C	-9.12985800	0.15213400	-0.27474000
H	-8.19335300	0.18929200	-0.79095100
H	-9.71239800	1.01249600	-0.53033800
O	-8.89626500	0.13658800	1.13596300
H	-8.16487900	0.72182800	1.34614600

AmB-Dod15

0 1

C	7.07527400	4.37143800	-1.19800600
H	7.76812100	3.61634400	-0.78067300
C	7.03161800	5.53619300	-0.19301200
H	6.36722200	6.32816700	-0.59624600
C	6.44407600	5.07101900	1.14559000
H	7.08282900	4.25597900	1.54990400
C	5.13340600	3.41217600	0.05832400
H	5.76740500	2.62610300	0.52867400

C	5.69212900	3.71742700	-1.34182700
H	5.78669400	2.75008300	-1.87981800
C	6.32260000	6.18542500	2.16770700
H	5.65286900	6.97386000	1.79056300
H	7.31269200	6.62385400	2.35500600
H	5.91587000	5.79644400	3.11244900
C	3.01182600	2.73940900	1.06832400
H	2.83769100	3.71557100	1.55208100
C	1.72274000	2.17826000	0.55624200
H	1.79428400	1.25897400	-0.03472700
C	0.50766500	2.73885500	0.77738100
H	0.44274300	3.66157300	1.36948400
C	-0.71754500	2.18941500	0.26322800
H	-0.63392800	1.27931300	-0.34282700
C	-1.96762000	2.71940200	0.45610300
H	-2.07456000	3.62927300	1.06158700
C	-3.14757000	2.13898200	-0.10187000
H	-3.00434600	1.22853100	-0.69746100
C	-4.42757300	2.60963100	0.07457800
H	-4.58699200	3.50555700	0.68898400
C	-5.57522800	1.98051700	-0.49116200
H	-5.40010100	1.09533900	-1.11733300
C	-6.87625500	2.38671500	-0.29832600
H	-7.06399500	3.26210600	0.33727200
C	-8.00761600	1.73452800	-0.86988200
H	-7.81130000	0.87389100	-1.52358800

C	-9.31601100	2.10063500	-0.65349700
H	-9.51777900	2.95277100	0.00889700
C	-10.44137400	1.43638800	-1.22990300
H	-10.23324800	0.60408700	-1.91539600
C	-11.75228400	1.75128500	-0.97858300
H	-11.97504500	2.57478400	-0.28748500
C	3.74300700	1.83328200	2.07946900
H	4.63613300	2.36434100	2.44109700
H	3.08819900	1.69847000	2.95561300
C	-12.86595100	1.03766900	-1.54261100
H	-12.61815600	0.23868800	-2.25349000
C	-14.16978700	1.24616700	-1.22638600
H	-14.42056000	2.04240600	-0.51055100
C	-15.32528200	0.44957500	-1.76447700
H	-14.94773900	-0.26426100	-2.51885900
C	-16.00041500	-0.38308200	-0.64289600
H	-16.52494300	0.33143200	0.02730000
C	-15.01958500	-1.18140100	0.25286800
H	-14.33763700	-0.42121000	0.67385900
C	-14.11323600	-2.13739300	-0.54348700
H	-13.73375400	-1.62837500	-1.43767500
C	-11.72148400	-2.34501100	-0.25373300
C	-10.60644300	-2.59171000	0.74242000
H	-10.94897600	-2.32375300	1.75280900
H	-10.39365300	-3.67613300	0.73931800
C	-9.32298600	-1.82772400	0.37395500

H	-9.54476500	-0.74129500	0.36610300
C	-8.19281400	-2.09873400	1.36728700
H	-8.52894900	-1.78584500	2.37076500
H	-7.99088000	-3.18449600	1.40527700
C	-6.88775100	-1.36248000	1.03575900
H	-7.10236100	-0.28130700	0.91757000
C	-5.83761800	-1.53489500	2.13665400
H	-5.74839100	-2.60882400	2.38388900
H	-6.22728800	-1.03675100	3.03958200
C	-4.44897800	-0.95767700	1.78063500
H	-4.54527600	-0.17356000	1.01227800
H	-4.01998700	-0.46100000	2.66688500
C	-3.41663800	-1.99782200	1.32532800
H	-3.24531300	-2.70825500	2.15848100
C	-2.05296500	-1.33344200	0.96389300
H	-1.95576700	-0.37493200	1.50405300
C	-0.84689900	-2.21866500	1.30037100
H	-0.93372100	-3.18615900	0.77386100
H	-0.86357200	-2.42673800	2.38532500
C	0.48673300	-1.55047300	0.95054700
H	0.52164300	-0.55228800	1.43047000
C	1.69367000	-2.35411300	1.43666700
H	1.68870300	-3.35382900	0.97165400
H	1.61330000	-2.48183700	2.52788900
C	3.04641500	-1.69120300	1.12628700
C	4.22516700	-2.43929000	1.76355100

H	4.35110700	-3.41571100	1.27352200
H	4.01303000	-2.61122600	2.83149800
C	5.51508900	-1.63829100	1.63705100
H	5.76143100	-1.52522100	0.56666900
C	5.32013600	-0.23410200	2.24958900
H	5.06579700	-0.33117100	3.31877700
C	4.10993900	0.45206000	1.53707900
H	4.33926300	0.51469800	0.45626600
C	6.56606500	0.62196000	2.19441000
C	-14.69779400	-3.48499000	-0.93517400
H	-13.97006300	-4.05475700	-1.53146200
H	-15.60050600	-3.32041400	-1.54123000
H	-14.96923000	-4.08021900	-0.05097600
C	-15.74494700	-1.85999000	1.42190800
H	-16.47943900	-2.60773900	1.08012500
H	-16.27890800	-1.11318800	2.03110700
H	-15.03211900	-2.38024800	2.07883800
C	-16.36297700	1.36129500	-2.44700400
H	-16.74886700	2.11193500	-1.73655000
H	-17.21113900	0.76880700	-2.81806700
H	-15.90769400	1.89930900	-3.29132100
O	4.84585300	4.60724200	-2.06438300
H	3.93207000	4.35225000	-1.83526600
O	8.35054300	6.03658600	0.01615200
H	8.74584700	6.03389100	-0.88059100
O	5.11625700	4.55446800	0.90368200

O	3.81610600	2.96921300	-0.13508000
O	3.29821400	-1.60665300	-0.26223300
H	2.43161800	-1.41144200	-0.68841800
O	2.95139400	-0.37104200	1.72015300
O	6.84256700	1.52535300	2.96192400
O	7.39185700	0.29715000	1.14250200
H	8.14744800	0.91750100	1.19985800
O	0.62101100	-1.37947600	-0.48043000
H	-0.27841100	-1.15968100	-0.81844300
O	-2.06747100	-1.00041500	-0.44269500
H	-2.67663600	-1.68746800	-0.81440600
O	-3.81930600	-2.75868400	0.17644300
H	-4.75845900	-2.52618300	-0.04617900
O	-6.31681400	-1.84507900	-0.20138900
H	-7.06514800	-2.02864900	-0.81394000
O	-8.85831500	-2.22043300	-0.93349900
H	-9.63958500	-2.16463600	-1.52938200
O	-11.52977700	-2.14786700	-1.45301100
O	-12.93229600	-2.37461300	0.32527600
O	-16.97293500	-1.22493600	-1.29273900
H	-17.59630000	-1.54104900	-0.61917500
O	6.54910400	-2.37474100	2.30833800
H	7.39823200	-2.02822700	1.98584000
N	7.66948600	4.87547300	-2.44383700
H	6.94848900	5.37382400	-2.97597400
H	7.99643800	4.10638000	-3.03280600

C	19.02599300	-7.04475100	-1.16104000
H	19.14857700	-6.97350200	-0.10047300
H	19.97759800	-7.21588300	-1.61936900
H	18.36842800	-7.85711900	-1.39031200
C	18.42638600	-5.73177000	-1.69783900
H	18.30559800	-5.80229500	-2.75865600
H	19.08302200	-4.91912900	-1.46686900
C	17.05553100	-5.48687800	-1.04026500
H	17.17625800	-5.41672100	0.02058400
H	16.39877700	-6.29933800	-1.27153800
C	16.45615700	-4.17363300	-1.57666000
H	16.33550700	-4.24375500	-2.63752500
H	17.11286600	-3.36115600	-1.34531700
C	15.08524800	-3.92880300	-0.91917700
H	15.20590600	-3.85868700	0.14168800
H	14.42853900	-4.74126500	-1.15052700
C	14.48588600	-2.61554600	-1.45555700
H	14.36524100	-2.68566000	-2.51642000
H	15.14260100	-1.80307600	-1.22420500
C	13.11498300	-2.37072100	-0.79807400
H	13.23563500	-2.30059700	0.26278100
H	12.45826100	-3.18318300	-1.02943600
C	12.51560800	-1.05746300	-1.33446600
H	12.39496400	-1.12757800	-2.39532900
H	13.17232400	-0.24499400	-1.10311400
C	11.14471300	-0.81263200	-0.67698100

H	11.26536300	-0.74251000	0.38388400
H	10.48799700	-1.62510100	-0.90833300
C	10.54533700	0.50062600	-1.21337300
H	10.42468600	0.43050200	-2.27422900
H	11.20205300	1.31309600	-0.98202100
C	9.17444100	0.74545600	-0.55587800
H	9.29509200	0.81558000	0.50497700
H	8.51772500	-0.06701300	-0.78723000
C	8.57505900	2.05870700	-1.09227200
H	7.62255300	2.22882000	-0.63544800
H	9.23178300	2.87117200	-0.86093100
O	8.41381700	1.96499100	-2.51005600
H	7.76046500	2.60524200	-2.80129100

AmB-Dod16

0 1

C	5.76982000	6.10236400	-1.69094600
H	6.48688000	6.21893700	-0.86771500
C	4.83884600	7.31357100	-1.64133200
H	4.12335100	7.23251500	-2.47428700
C	4.03643600	7.31431100	-0.34578300
H	4.74753500	7.37042100	0.49636100
C	4.10307300	4.95111500	-0.23020600
H	4.74670200	4.98611000	0.66506000

C	4.97906500	4.81544200	-1.47449500
H	5.66286000	3.96793200	-1.30487000
C	3.05409600	8.46032400	-0.26563800
H	2.32883300	8.38161800	-1.07974000
H	3.58711600	9.40775200	-0.35860100
H	2.51413100	8.43659000	0.68337700
C	2.40640900	3.68161600	0.91067800
H	2.01039800	4.66518600	1.18245600
C	1.28800600	2.80325500	0.44476700
H	1.56984900	1.85728500	-0.00966700
C	-0.00103500	3.13219100	0.56449800
H	-0.27017900	4.07813900	1.03447100
C	-1.09509300	2.30980300	0.08551300
H	-0.83403900	1.39801800	-0.44588500
C	-2.39246500	2.62890800	0.24549400
H	-2.64506200	3.54248100	0.78400600
C	-3.49727000	1.84758800	-0.26013000
H	-3.24953500	0.95348900	-0.82921000
C	-4.78757900	2.18168300	-0.05673200
H	-5.00604800	3.07781800	0.52462600
C	-5.92477900	1.44748600	-0.55462700
H	-5.74266100	0.55760000	-1.15514200
C	-7.19855300	1.80996000	-0.29724200
H	-7.38024500	2.69048500	0.31922000
C	-8.36152700	1.11073500	-0.78422200
H	-8.19183700	0.25228200	-1.43123700

C	-9.62530800	1.45911400	-0.46905500
H	-9.78745000	2.30646500	0.19680800
C	-10.80037200	0.77197900	-0.94793000
H	-10.64226000	-0.03229000	-1.66364500
C	-12.05490400	1.04546700	-0.54497300
H	-12.21749100	1.83138200	0.19204900
C	3.21046900	3.11836000	2.08873100
H	3.98490500	3.84316800	2.35389600
H	2.55432100	3.01167100	2.95761200
C	-13.21855800	0.30970100	-0.99980700
H	-13.04760500	-0.39950300	-1.80679900
C	-14.43726800	0.40414000	-0.45669600
H	-14.59817600	1.10326000	0.36582200
C	-15.62422300	-0.42613500	-0.85358300
H	-15.38442500	-0.97656700	-1.77039400
C	-15.95677900	-1.46816600	0.23264900
H	-16.32174700	-0.91507500	1.11415700
C	-14.76100700	-2.31376400	0.70995400
H	-14.01670100	-1.59905400	1.07403600
C	-14.07099300	-3.07506800	-0.42719800
H	-13.93313400	-2.41557000	-1.27963900
C	-11.68651000	-3.07819300	-0.67270900
C	-10.37779400	-3.49461000	-0.04973100
H	-10.51418000	-3.64115200	1.02316600
H	-10.10466600	-4.46105100	-0.48933200
C	-9.25584000	-2.48942600	-0.33213500

H	-9.51989100	-1.52350400	0.12344200
C	-7.92905200	-2.97036500	0.24008200
H	-8.05237700	-3.14723200	1.31429600
H	-7.67552600	-3.92786000	-0.23109100
C	-6.76861700	-1.99678600	0.02835700
H	-7.03405700	-1.02842200	0.48222800
C	-5.49342900	-2.51157100	0.68737300
H	-5.30225500	-3.50756400	0.26297100
H	-5.66299400	-2.65127700	1.76160900
C	-4.30181300	-1.58628000	0.45693300
H	-4.23230000	-1.35013500	-0.60711000
H	-4.46513100	-0.63962200	0.98152000
C	-2.97257500	-2.18083900	0.90136500
H	-3.02746800	-2.47866000	1.95959000
C	-1.80465700	-1.20913100	0.72453200
H	-2.05950900	-0.24045400	1.16536700
C	-0.51364500	-1.74173100	1.34100400
H	-0.28503800	-2.71671100	0.89769900
H	-0.68000200	-1.89974600	2.41277700
C	0.68193100	-0.80965000	1.16217800
H	0.44661700	0.17713000	1.58247200
C	1.91504000	-1.35838400	1.87021400
H	2.11786100	-2.37509100	1.51941600
H	1.70812800	-1.40275000	2.94329700
C	3.17905800	-0.52926900	1.64568800
C	4.33473600	-0.97706500	2.53204600

H	4.65327500	-1.98396900	2.25395000
H	4.00675900	-0.99259100	3.57518500
C	5.51549300	-0.02898900	2.41740300
H	5.90931800	-0.07835200	1.39271200
C	5.04668900	1.42874300	2.67328300
H	4.76423100	1.49365100	3.72977000
C	3.84195100	1.76655200	1.78742000
H	4.15722300	1.73637900	0.74028700
C	6.25300300	2.30791300	2.48712900
C	-14.72821800	-4.36189400	-0.88277200
H	-14.18189900	-4.78102600	-1.73089100
H	-15.75418000	-4.14890900	-1.18579300
H	-14.74085400	-5.10386100	-0.08148500
C	-15.14908200	-3.22167800	1.87566300
H	-15.94438100	-3.92153000	1.60176500
H	-15.49808200	-2.62990300	2.72680500
H	-14.29265500	-3.81383900	2.20600800
C	-16.84807400	0.45088300	-1.13299000
H	-17.10768400	1.04389900	-0.24894900
H	-17.70814900	-0.16694300	-1.39431900
H	-16.64582100	1.14446100	-1.95229700
O	4.18715800	4.59948500	-2.62099100
H	3.43782300	4.05683400	-2.33613400
O	5.59610900	8.49833900	-1.71851000
H	6.26556700	8.31008500	-2.39590600
O	3.29260700	6.10179200	-0.26992400

O	3.27451000	3.83604300	-0.22749800
O	3.61943000	-0.59664800	0.32047000
H	2.82315600	-0.53346700	-0.23721300
O	2.82115700	0.80250100	2.00441400
O	7.21677200	2.28852800	3.22449300
O	6.23933000	3.07322900	1.37998100
H	7.10652700	3.50978500	1.34456100
O	1.00465900	-0.65376900	-0.22249700
H	0.16639700	-0.65089500	-0.71740500
O	-1.63533000	-0.96967100	-0.68086000
H	-1.79247900	-1.83580300	-1.09428000
O	-2.60942500	-3.30632200	0.09756600
H	-3.40159700	-3.81253900	-0.11181800
O	-6.47518400	-1.81419200	-1.34724400
H	-7.31252800	-1.88031500	-1.83545200
O	-9.07880200	-2.31793100	-1.73381900
H	-9.96460600	-2.22474700	-2.12825600
O	-11.75728100	-2.51334800	-1.75315700
O	-12.73518800	-3.41073800	0.06586800
O	-17.00664200	-2.27447100	-0.29342700
H	-17.43606800	-2.73633200	0.43452800
O	6.48717600	-0.44540800	3.35081700
H	7.08214400	0.30002800	3.52419000
N	6.51240200	6.16475900	-2.94564900
H	5.90300700	5.83242600	-3.68984400
H	7.31519100	5.54408600	-2.92359200

C	8.75175300	0.64363800	0.87855300
H	8.30990300	-0.09369600	1.55547500
H	7.93225700	1.17384200	0.38325100
H	9.29078700	1.36697700	1.49897500
C	9.67730700	-0.02372600	-0.13209100
H	10.08884600	0.72985100	-0.81555300
H	9.10033400	-0.71703300	-0.75623600
C	10.82493000	-0.78091000	0.53029400
H	10.41214300	-1.53425300	1.21400000
H	11.40178600	-0.08795400	1.15736300
C	11.75991800	-1.45892000	-0.46652700
H	12.17086900	-0.70465600	-1.15084700
H	11.18229100	-2.15202200	-1.09238400
C	12.90675700	-2.21515100	0.19757900
H	12.49578900	-2.96952100	0.88140900
H	13.48385600	-1.52213500	0.82404900
C	13.84275300	-2.89325900	-0.79852700
H	14.25348400	-2.13886600	-1.48262500
H	13.26585500	-3.58670400	-1.42471800
C	14.98970400	-3.64873100	-0.13377000
H	14.57894000	-4.40334200	0.55009700
H	15.56613500	-2.95522300	0.49274000
C	15.92648800	-4.32626700	-1.12947700
H	16.33850300	-3.57203500	-1.81267700
H	15.35002000	-5.01930100	-1.75658500
C	17.07313600	-5.08209000	-0.46466100

H	16.66176800	-5.83828200	0.21764100
H	17.64788400	-4.38885200	0.16362300
C	18.01136500	-5.75254500	-1.46305800
H	18.43565000	-5.00256100	-2.13833800
H	17.43779000	-6.44612300	-2.09305300
C	19.15403100	-6.51053500	-0.79404700
H	18.75433600	-7.30017300	-0.14591600
H	19.72338400	-5.82921200	-0.15097200
C	20.11311000	-7.13798000	-1.78799500
H	20.87355900	-7.72677900	-1.25290400
H	19.56186800	-7.83044400	-2.44413000
O	20.71136200	-6.09267200	-2.53707700
H	21.27052500	-6.48427100	-3.21633300

structures with protonation and deprotonation in basic and acidic groups

AmB-DSPE

0 1

C	-10.52914200	2.22885100	0.78693000
H	-10.84464100	2.28423000	-0.25740600
C	-10.63655000	3.58145900	1.46713500
H	-10.30551400	3.48208300	2.50980900
C	-9.68976000	4.56582500	0.76569800
H	-10.01967500	4.65629000	-0.28300700

C	-8.25530500	2.80309900	0.15616500
H	-8.63638100	2.90732100	-0.86210500
C	-9.10317200	1.73344500	0.83864200
H	-9.00299100	0.79906700	0.28186100
C	-9.68382600	5.92999800	1.42257600
H	-9.37995700	5.84352000	2.46952600
H	-10.67084700	6.39781200	1.36697200
H	-8.97078000	6.58108900	0.91446000
C	-6.12430200	2.87013100	-0.90188500
H	-6.12620200	3.96827400	-0.88561500
C	-4.74396200	2.36527700	-0.62568400
H	-4.62503600	1.28520600	-0.59832100
C	-3.68439600	3.16291900	-0.46584500
H	-3.81596900	4.24437900	-0.49508500
C	-2.33849100	2.66796400	-0.26430800
H	-2.20953000	1.58818900	-0.27654200
C	-1.25780500	3.44894900	-0.07634900
H	-1.37455800	4.53224000	-0.05817100
C	0.06809700	2.91045600	0.09645300
H	0.14957900	1.82807700	0.04765500
C	1.18844700	3.63521000	0.29120200
H	1.13193100	4.72194100	0.34322000
C	2.48708300	3.02353100	0.41996400
H	2.52180500	1.93781800	0.35143100
C	3.64745800	3.68609100	0.60198000
H	3.63909600	4.77322300	0.67267000

C	4.92290200	3.02276600	0.70668600
H	4.91445100	1.93623000	0.64101700
C	6.10250700	3.65467700	0.86962600
H	6.11813700	4.74252500	0.92924300
C	7.36929400	2.97105300	0.96623000
H	7.33918500	1.88347000	0.94116400
C	8.56186400	3.58619800	1.07129700
H	8.60066300	4.67500800	1.08927100
C	-6.65916800	2.38762100	-2.26134800
H	-7.62090300	2.85738600	-2.47094600
H	-5.96684300	2.71751400	-3.04129700
C	9.82201500	2.87365500	1.13040100
H	9.75386100	1.78867500	1.16109000
C	11.02904700	3.45139000	1.11078900
H	11.09799500	4.53990700	1.07094900
C	12.32878200	2.70011900	1.10537500
H	12.11750700	1.64054000	1.27005000
C	13.04240100	2.81521500	-0.25524400
H	13.39963800	3.85323900	-0.35635900
C	12.14867500	2.53068700	-1.47618000
H	11.29954300	3.21522700	-1.39284000
C	11.54641200	1.12355500	-1.43761600
H	11.22316100	0.89238500	-0.42649700
C	9.23642100	0.61764300	-1.78683400
C	8.07227500	0.69121800	-2.74008300
H	8.27451700	1.43540800	-3.51212600

H	7.99463500	-0.28955000	-3.22412500
C	6.74991000	0.99150500	-2.02521700
H	6.81572300	1.98098200	-1.55038400
C	5.58657300	0.97340100	-3.00393500
H	5.76675000	1.72436500	-3.78053000
H	5.55287100	-0.00840200	-3.49121900
C	4.22390400	1.24522200	-2.37117000
H	4.26281100	2.18835500	-1.80955700
C	3.14502700	1.35157000	-3.44286600
H	3.23783300	0.49350200	-4.12034000
H	3.36475900	2.24323600	-4.03753500
C	1.71271000	1.42629100	-2.89204300
H	1.71756600	1.76378100	-1.85346600
H	1.13815600	2.17224300	-3.44973800
C	0.94974700	0.10941000	-2.99179100
H	0.85756100	-0.15566500	-4.05455200
C	-0.47209100	0.22220600	-2.39269600
H	-0.80455100	1.26592200	-2.42958100
C	-1.49468400	-0.65154600	-3.10905300
H	-1.19480600	-1.70335500	-3.02699500
H	-1.49433500	-0.39063800	-4.17355900
C	-2.90620000	-0.46123600	-2.56352900
H	-3.15601000	0.60719900	-2.60413700
C	-3.94459200	-1.21681500	-3.38264700
H	-3.72630400	-2.28887300	-3.36022800
H	-3.88105900	-0.87421600	-4.41974900

C	-5.37624300	-1.00722200	-2.88581400
C	-6.43229800	-1.59220400	-3.82457400
H	-6.36737700	-2.68297600	-3.81037800
H	-6.25208300	-1.24535900	-4.84620000
C	-7.81447100	-1.13612500	-3.38560000
H	-7.98471700	-1.50056800	-2.36218800
C	-7.86627200	0.40408800	-3.34695600
H	-7.59851000	0.77205500	-4.34570000
C	-6.80651700	0.87367600	-2.35516300
H	-7.02490200	0.45669700	-1.36596200
C	-9.32516400	0.81710900	-3.08938800
C	12.41060300	-0.00443300	-1.96046100
H	11.88686100	-0.95328400	-1.84264700
H	13.34012700	-0.03866500	-1.39072400
H	12.64408100	0.13246200	-3.01834500
C	12.87154400	2.83449400	-2.78662200
H	13.77958600	2.23486200	-2.90193200
H	13.15562600	3.88960000	-2.83457400
H	12.22816200	2.62081100	-3.64356800
C	13.25642100	3.16983800	2.22869900
H	13.47260400	4.23984700	2.13411400
H	14.20070100	2.62408900	2.19272700
H	12.79109300	3.00759700	3.20374700
O	-8.77557900	1.57213200	2.20611900
H	-7.86854400	1.22872100	2.27366300
O	-12.00793100	3.94969300	1.39415200

H	-12.16060700	4.70223500	1.97625300
O	-8.37239700	4.05460000	0.82961800
O	-6.93993300	2.39478100	0.18302400
O	-5.58039200	-1.61910600	-1.64192500
H	-4.86267500	-1.32660800	-1.05632800
O	-5.52472900	0.39798200	-2.79342500
O	-9.65622400	1.33799300	-1.99981700
O	-2.99649600	-0.89770900	-1.20181400
H	-2.13114800	-0.72838400	-0.78784700
O	-0.40456200	-0.13020400	-1.00869000
H	0.34125100	-0.76353500	-0.97866200
O	1.59789000	-0.96311800	-2.32511700
H	2.45730800	-0.65840000	-1.96263500
O	3.85283500	0.19965700	-1.46645100
H	4.65984400	-0.11608000	-1.02201500
O	6.47762700	0.01332000	-1.02958500
H	7.30699800	-0.16794100	-0.54800500
O	9.15602400	0.07728600	-0.69325700
O	10.33869500	1.17147600	-2.26391000
O	14.15843000	1.92880000	-0.19507700
H	14.79034800	2.17622600	-0.87846100
O	-8.82753700	-1.67627800	-4.23786900
H	-9.35728800	-0.87556500	-4.52304100
N	-11.43367500	1.26461900	1.47889900
H	-11.72259500	0.46465400	0.86568400
H	-12.26850900	1.76789600	1.79078800

C	14.47347500	-1.53173800	2.39427400
H	14.60439500	-1.85578300	3.43409000
H	14.38864900	-2.44820000	1.79785900
C	13.18286700	-0.72976000	2.26681900
H	13.06826900	-0.42022300	1.21958300
H	13.28080300	0.20223400	2.83925300
C	11.92559000	-1.46814300	2.71670200
H	12.00964100	-1.72844400	3.77967500
H	11.84388400	-2.41807300	2.17374600
C	10.66273900	-0.64524900	2.48283000
H	10.75248700	0.31528800	3.00843800
H	10.59066500	-0.40235700	1.41532600
C	9.36476100	-1.32838800	2.90377500
H	9.38923700	-1.55129200	3.97816300
H	9.28174200	-2.29483400	2.38980900
C	8.14228600	-0.48015000	2.57482300
H	8.22499700	0.49627500	3.07478400
H	8.15531400	-0.26074100	1.49934500
C	6.80552500	-1.12037000	2.93450000
H	6.75310200	-1.27864600	4.01933600
H	6.74599800	-2.11738100	2.47754400
C	5.61008400	-0.28896700	2.47691100
H	5.69289600	0.72900900	2.88018200
H	5.65157300	-0.18214600	1.38544200
C	4.26570000	-0.88486700	2.87943000
H	4.21528600	-0.94705400	3.97434400

H	4.20103500	-1.91778100	2.51390200
C	3.06285700	-0.09755500	2.36444300
H	3.16388200	0.95858200	2.64578800
H	3.06126300	-0.11847000	1.26605800
C	1.74170800	-0.64246200	2.89890400
H	1.76761200	-0.60794300	3.99611100
H	1.65634200	-1.70438600	2.63375400
C	0.49632200	0.09326900	2.40855200
H	0.40367300	-0.02006400	1.32224200
H	0.59987500	1.17005200	2.59277100
C	-0.76736700	-0.42909900	3.08610800
H	-0.65768400	-0.31133700	4.17256200
H	-0.84426400	-1.51007600	2.91025800
C	-2.07719400	0.22841600	2.65171800
H	-2.23993600	0.06359000	1.57894500
H	-2.01738400	1.31573400	2.78555700
C	-3.24663200	-0.35287900	3.44097800
H	-3.06174800	-0.22250800	4.51374200
H	-3.27871300	-1.43356700	3.28047200
C	-4.61950500	0.22099800	3.10739200
H	-4.82775000	0.20346600	2.03058900
H	-4.72252900	1.26721000	3.41182700
C	-5.71095100	-0.59081700	3.75490400
O	-5.56702000	-1.67604800	4.26173600
C	15.69127500	-0.73141700	1.94103400
H	16.60674800	-1.32843000	1.97605400

H	15.55713000	-0.36669200	0.91772300
H	15.83928300	0.14740400	2.57686800
C	-8.06989600	-0.73399800	4.07941200
H	-7.78476100	-1.45877900	4.84560600
H	-8.77062400	-0.00592900	4.48231500
C	-8.69418200	-1.42463600	2.87718700
H	-8.79172100	-0.72227100	2.05744400
C	-10.06281000	-2.02643700	3.19227600
H	-10.01345900	-3.11448700	3.13373200
H	-10.39896400	-1.75676600	4.19985300
O	-6.91696600	0.02886200	3.68675700
C	-7.01722700	-2.11036400	1.38477700
C	-6.18600000	-3.24927900	0.87404600
H	-6.53749300	-4.19753000	1.28308300
H	-6.29750900	-3.25014700	-0.21205800
C	-4.72181000	-2.97483900	1.24135700
H	-4.57643300	-3.16902600	2.30955600
H	-4.53335800	-1.90581200	1.09804000
C	-3.69327800	-3.73085800	0.41101000
H	-3.85104900	-4.81429400	0.48243800
H	-3.82401500	-3.45356000	-0.64033400
C	-2.28149200	-3.36853700	0.86323100
H	-2.08994900	-3.80266200	1.85369600
H	-2.23238700	-2.28131800	1.00274600
C	-1.17195700	-3.77298500	-0.09898900
H	-1.36646600	-3.30543600	-1.07248500

H	-1.18632900	-4.85771300	-0.26461700
C	0.20055900	-3.34183400	0.40722600
H	0.17119700	-2.26294900	0.60662600
H	0.39384200	-3.81188300	1.38092900
C	1.35537000	-3.64591300	-0.54141100
H	1.16571000	-3.18103200	-1.51432100
H	1.41541200	-4.72863900	-0.71223800
C	2.68814600	-3.14088600	0.00242500
H	2.60151200	-2.06992300	0.22576700
H	2.89145000	-3.63083900	0.96457700
C	3.87501800	-3.35760900	-0.93208900
H	3.77144300	-2.70821300	-1.81054900
H	3.86165000	-4.38852900	-1.30931200
C	5.21156100	-3.09590600	-0.24395000
H	5.22021500	-2.08084100	0.17157400
H	5.30223800	-3.76983700	0.61859900
C	6.42591300	-3.27447700	-1.14787500
H	6.37040700	-4.25110900	-1.64676300
H	6.39967300	-2.51499100	-1.93825000
C	7.74588000	-3.17160500	-0.39048100
H	7.81765400	-2.19397200	0.10004400
H	7.75335500	-3.91188500	0.42059100
C	8.97629200	-3.38110900	-1.26539200
H	8.91904300	-4.36834500	-1.74247800
H	8.97134600	-2.64743000	-2.08347300
C	10.28493500	-3.25918200	-0.49156900

H	10.24216300	-3.90127400	0.39819200
H	10.38026100	-2.23140600	-0.12093300
C	11.51858100	-3.62474300	-1.31136000
H	11.45177000	-4.67199300	-1.63290300
H	11.53116200	-3.03305500	-2.23703000
C	12.82419900	-3.40805700	-0.55151100
H	12.83519200	-4.05632700	0.33348200
H	12.85137700	-2.38159000	-0.17011300
C	14.06646400	-3.66444200	-1.39654300
H	14.98230700	-3.50753600	-0.81980000
H	14.08227500	-4.68979500	-1.77877100
H	14.09644400	-2.99079200	-2.25958900
O	-7.83738300	-2.46482200	2.39048500
O	-6.89874800	-0.96577500	1.00252300
O	-11.07500900	-1.51810000	2.31034500
O	-12.15941100	-1.02321600	0.20620400
O	-11.71004600	-3.51051500	0.72432900
O	-9.76213600	-1.87681500	0.14231600
C	-12.77916000	-1.33483200	-1.04615900
H	-13.13032900	-2.37139700	-1.00522300
H	-13.64871600	-0.67965400	-1.13725800
C	-11.86820100	-1.08104400	-2.23670300
H	-11.43303000	-0.08138100	-2.19796500
H	-12.43579900	-1.16262000	-3.16535700
N	-10.73014600	-2.04439500	-2.29352000
H	-11.06044100	-3.01036500	-2.30461100

H	-10.10116800	-1.90091600	-3.12659400
P	-11.08992000	-2.16846300	0.80844400
O	-10.11760100	0.48784100	-4.01976200
H	-10.15180400	-1.94820200	-1.39377200
H	-10.95013400	0.84526900	2.28026100

AmB-Cyc

0 1

C	-9.69960800	4.77016900	-2.03789000
C	-11.01741000	4.27929700	-1.45191400
C	-10.91315100	2.84195900	-0.97858600
C	-10.30857400	1.96727100	-2.06871800
C	-8.97236400	2.56147400	-2.50421600
C	-8.23906400	1.79642100	-3.58638700
O	-8.77151400	4.90733500	-1.00520400
O	-11.38876900	5.14036400	-0.39236200
O	-12.21339900	2.45239200	-0.61877000
O	-9.23518200	3.87314500	-3.01396600
O	-6.87269700	2.17184500	-3.63922600
H	-10.99068400	1.93428900	-2.92808500
H	-9.84901100	5.72509000	-2.55192300
H	-11.76853200	4.30939700	-2.25328300
H	-10.22930100	2.80946600	-0.11607800
H	-8.31198700	2.62694800	-1.63127300

H	-8.33955000	0.72176800	-3.40291000
H	-8.69807200	2.02352900	-4.55375000
H	-12.14045200	4.71671300	0.04552300
H	-12.23192100	1.48928100	-0.46006100
H	-6.34916700	1.51514900	-3.13145600
C	-10.71851200	-0.41672300	-2.18387200
C	-11.58472900	-1.19995400	-1.20002500
C	-10.72287500	-1.84165700	-0.13417600
C	-9.65145600	-2.67087700	-0.81437500
C	-8.82701800	-1.81566600	-1.77287900
C	-7.76344200	-2.60398500	-2.53776700
O	-10.14502100	0.67053900	-1.51573700
O	-12.54855000	-0.33774600	-0.62616700
O	-11.57728000	-2.62070000	0.66586500
O	-9.72847600	-1.25080200	-2.73510800
O	-6.47112900	-2.26529500	-2.05536200
H	-10.14233700	-3.45606400	-1.40444500
H	-11.32229100	-0.07487100	-3.03000900
H	-12.08524800	-2.00052800	-1.76142200
H	-10.23384400	-1.04828800	0.45037500
H	-8.32564800	-1.01148200	-1.22164300
H	-7.92172600	-3.68019200	-2.42264900
H	-7.85366900	-2.34903800	-3.59936500
H	-12.91890900	-0.81939000	0.12793700
H	-11.04660800	-3.11642700	1.31827800
H	-5.90312000	-3.07314200	-1.93455200

C	-8.82148000	-4.65047500	0.25145500
C	-8.95935700	-5.09783400	1.70495400
C	-7.71856700	-4.75289200	2.50133400
C	-6.49477700	-5.28871200	1.77828900
C	-6.45191400	-4.72200300	0.36279100
C	-5.24870800	-5.15577600	-0.45277600
O	-8.85086800	-3.25121600	0.19589700
O	-10.11624200	-4.50044600	2.26022900
O	-7.87554100	-5.30789400	3.78259700
O	-7.64004500	-5.14668900	-0.31889500
O	-4.75929700	-4.06313500	-1.22042500
H	-6.55208300	-6.38360700	1.72442300
H	-9.63951300	-5.07225900	-0.34029500
H	-9.06201500	-6.19175700	1.70145600
H	-7.62853400	-3.65639200	2.54843800
H	-6.45223000	-3.62748700	0.42925100
H	-4.45950000	-5.52516900	0.20677100
H	-5.55540200	-5.97836200	-1.10922700
H	-10.06128700	-4.63766700	3.21634600
H	-7.04526700	-5.18813000	4.28271000
H	-3.90131300	-4.45511600	-1.78061400
C	-4.49304900	-5.88380700	2.98525500
C	-4.12812500	-5.57613200	4.43130400
C	-3.25247800	-4.34125200	4.52666000
C	-2.08323000	-4.47011000	3.56055400
C	-2.60534800	-4.72338500	2.14979800

C	-1.55836800	-4.83035900	1.05003500
O	-5.36809300	-4.88551800	2.52823400
O	-5.32115300	-5.42867600	5.18221000
O	-2.84934300	-4.23871500	5.86911700
O	-3.34093800	-5.95558900	2.19151800
O	-1.36461600	-3.54772800	0.50089100
H	-1.46846400	-5.32939000	3.86057800
H	-4.97037800	-6.86638700	2.91791600
H	-3.54395300	-6.42527800	4.81058400
H	-3.84653300	-3.46587700	4.22034300
H	-3.27864200	-3.90809200	1.86195000
H	-0.61658700	-5.24885200	1.42006200
H	-1.95258300	-5.51117200	0.28696700
H	-5.05841000	-5.06349300	6.03847600
H	-2.06904900	-3.65414500	5.92824400
H	-0.41841200	-3.31902200	0.45313400
C	0.05265900	-3.37252000	3.82892200
C	0.44606800	-2.55305500	5.05565100
C	0.19585300	-1.08225000	4.80614400
C	0.88550500	-0.65695800	3.52731700
C	0.49500500	-1.55099600	2.35542800
C	1.32392100	-1.26249200	1.12242200
O	-1.33567800	-3.27484600	3.66018300
O	-0.29073000	-3.01034800	6.17781900
O	0.67628800	-0.37299000	5.92547800
O	0.74825600	-2.91832500	2.70404400

O	0.85834500	-2.01811500	0.02607600
H	1.97170100	-0.71007700	3.68627800
H	0.34893300	-4.41762200	3.96325400
H	1.52111900	-2.69815900	5.22523800
H	-0.88564300	-0.93268500	4.67344500
H	-0.56468000	-1.41562600	2.11383100
H	1.27762300	-0.19492300	0.89676700
H	2.36545900	-1.54454600	1.32083100
H	-0.15062900	-2.35491400	6.87583500
H	0.82943400	0.55249300	5.66151900
H	0.46998600	-1.41776000	-0.63716600
C	1.41886300	1.58667200	2.79810300
C	1.51721500	2.77134500	3.75763600
C	0.24372400	3.58832300	3.72170500
C	-0.10243200	3.91486800	2.27905500
C	-0.25820600	2.62553600	1.47671600
C	-0.68013400	2.80061900	0.01970900
O	0.48681300	0.68661900	3.31874400
O	1.77773500	2.27749300	5.05977600
O	0.47260000	4.74501400	4.49262100
O	1.03835600	2.00773200	1.51137500
O	-2.05957000	2.45260000	-0.08450600
H	0.72685600	4.47460800	1.82781800
H	2.39722400	1.11057800	2.68062600
H	2.34089400	3.41559800	3.42073300
H	-0.57328500	2.98326200	4.14185700

H	-0.99142100	1.96326200	1.94790000
H	-0.52337200	3.83289100	-0.31007600
H	-0.07233500	2.13201300	-0.60116700
H	1.67662200	3.03512600	5.65314200
H	-0.32935500	5.29872600	4.47924700
H	-2.51457300	3.01542900	-0.74230900
C	-1.10839500	6.02819200	1.85455800
C	-1.96226400	6.94822400	2.72331700
C	-3.43756300	6.71060300	2.47218700
C	-3.69608200	6.76978400	0.97596000
C	-2.82490200	5.73925800	0.26668500
C	-3.04592900	5.60178500	-1.23488400
O	-1.26725800	4.71494200	2.30229000
O	-1.61468000	6.74054000	4.07883900
O	-4.15046400	7.69935300	3.17718100
O	-1.46593300	6.13891200	0.49970100
O	-3.57628300	4.30989100	-1.49383200
H	-3.42250800	7.76536800	0.60291200
H	-0.05967400	6.33365300	1.91751700
H	-1.73710200	7.98200600	2.42586300
H	-3.69629400	5.70137400	2.82541400
H	-2.99137800	4.75167300	0.70681800
H	-3.73200300	6.36988400	-1.60334600
H	-2.07873000	5.73203100	-1.73483000
H	-2.29095700	7.18315300	4.60916100
H	-5.09507900	7.62303000	2.95087000

H	-4.29556000	4.37238400	-2.16435800
C	-5.79382300	7.53554000	0.11036500
C	-7.15334300	7.69512100	0.78121000
C	-8.00330900	6.46086700	0.55954000
C	-8.03471200	6.11373100	-0.91954100
C	-6.61875500	5.96535100	-1.46796300
C	-6.52043000	5.64441800	-2.96133500
O	-5.08007300	6.54502900	0.78612800
O	-6.94691600	7.95201400	2.15716200
O	-9.28903400	6.73676300	1.06178800
O	-5.94783900	7.21550100	-1.24986300
O	-5.62686500	4.57342000	-3.22325000
H	-8.55400100	6.91623700	-1.46055000
H	-5.25297600	8.48680700	0.13400500
H	-7.66103900	8.54443800	0.30358300
H	-7.53655200	5.61900200	1.09266800
H	-6.10566700	5.17090800	-0.91518300
H	-7.50068300	5.40013100	-3.37407200
H	-6.14482700	6.53849000	-3.46732700
H	-7.80911900	7.87010400	2.58670900
H	-9.90562200	6.06136100	0.72556500
H	-6.14315800	3.74868500	-3.36426700
C	-3.62730300	-1.09557300	-1.54188400
H	-3.77951700	-1.85710200	-2.31297300
C	-4.09618000	0.26039100	-2.05195300
H	-3.96013400	1.03470300	-1.28799400

C	-3.19868500	0.61976200	-3.25666400
H	-3.30955600	-0.18523700	-4.00132300
C	-1.40946500	-0.63081800	-2.42085900
H	-1.66424700	-1.33950700	-3.20904600
C	-2.14794000	-1.07721700	-1.17555300
H	-1.84658000	-2.09242100	-0.91654200
C	-3.51087200	1.95116100	-3.89771900
H	-3.34504600	2.76314900	-3.18998900
H	-4.53848000	1.98454200	-4.26141200
H	-2.84122600	2.10180500	-4.74670100
C	0.81275800	-1.04775600	-3.27432800
H	0.71865100	-0.29846700	-4.06981000
C	2.19189000	-1.01339800	-2.69568800
H	2.38696300	-1.71752700	-1.89145400
C	3.15038000	-0.16867100	-3.08686600
H	2.96383000	0.53936100	-3.89406100
C	4.45078700	-0.13613500	-2.44866200
H	4.59707800	-0.82928500	-1.62377000
C	5.46019100	0.69159900	-2.77730000
H	5.33671600	1.39670800	-3.59888200
C	6.71756900	0.69137500	-2.06855200
H	6.79891900	-0.02755400	-1.25530800
C	7.76940100	1.48821000	-2.34433500
H	7.70375900	2.20822200	-3.15948800
C	9.00251200	1.43634600	-1.59900000
H	9.06218700	0.69890400	-0.79863500

C	10.07761600	2.21681200	-1.83202800
H	10.04225500	2.94771700	-2.63921500
C	11.28783700	2.15334500	-1.05170900
H	11.30826700	1.43442100	-0.23281700
C	12.37962800	2.91393900	-1.26983900
H	12.37894800	3.62046600	-2.09915100
C	13.56841900	2.85208700	-0.45473900
H	13.53338600	2.19168300	0.40998500
C	14.70580000	3.52957200	-0.69566600
H	14.77169200	4.17472700	-1.57103400
C	0.40983100	-2.42404200	-3.83581400
H	-0.54965000	-2.36034200	-4.34680100
H	1.15560600	-2.69882500	-4.58780500
C	15.88447600	3.40872500	0.13995900
H	15.75243900	2.84357200	1.06051400
C	17.09717500	3.88164900	-0.16717300
H	17.23302300	4.43276800	-1.09922700
C	18.33268500	3.67021100	0.66059400
H	18.04439800	3.24049200	1.62677200
C	19.28963800	2.66847300	-0.01475200
H	19.68974700	3.15678600	-0.91901600
C	18.63305600	1.35578900	-0.48171200
H	17.84138300	1.65009000	-1.17745700
C	17.92016800	0.59989400	0.64473600
H	17.31805900	1.29125400	1.22775400
C	15.72108200	-0.32028700	0.36107600

C	14.89141100	-1.30655300	-0.42167800
H	15.36953900	-1.50048400	-1.38364000
H	14.88440200	-2.24616600	0.14303500
C	13.44835400	-0.83145600	-0.60881400
H	13.45040100	0.11399400	-1.17001800
C	12.62610500	-1.86501100	-1.36477100
H	13.08415900	-2.02769100	-2.34628100
H	12.66283900	-2.81564900	-0.81929000
C	11.16179700	-1.48003900	-1.56221100
H	11.10363900	-0.48857100	-2.03203900
C	10.44186500	-2.48989200	-2.44984300
H	10.67623800	-3.50115800	-2.09505400
H	10.86745800	-2.40468100	-3.45441900
C	8.91716700	-2.29793300	-2.50095400
H	8.65071200	-1.27918200	-2.20954600
H	8.56037200	-2.42212300	-3.52800400
C	8.14180100	-3.29311300	-1.63983800
H	8.30593000	-4.30005700	-2.04837700
C	6.61909300	-3.00204200	-1.65449700
H	6.36094600	-2.43119000	-2.55417100
C	5.77313700	-4.26901400	-1.60797700
H	6.01496700	-4.83284100	-0.69871800
H	6.03926700	-4.89707100	-2.46626200
C	4.27308000	-3.98439400	-1.65030600
H	4.05860900	-3.35067200	-2.52249700
C	3.46319200	-5.26616100	-1.79084400

H	3.70430100	-5.94975500	-0.97124400
H	3.74696100	-5.74774300	-2.73183100
C	1.94849000	-5.05461000	-1.78168700
C	1.17335900	-6.27957300	-2.24632700
H	1.34093200	-7.11966900	-1.56771600
H	1.51854500	-6.56498700	-3.24446200
C	-0.30424700	-5.94885100	-2.28474700
H	-0.61279300	-5.70931200	-1.26514600
C	-0.60103400	-4.71311800	-3.14988800
H	-0.46248000	-4.99401500	-4.20178600
C	0.35840400	-3.55173000	-2.80369800
H	0.05121000	-3.16661800	-1.82983600
C	-2.06826600	-4.28287900	-2.95692800
C	18.79240300	-0.22090000	1.57237400
H	18.18461400	-0.66059800	2.36646500
H	19.54891600	0.42833500	2.01516600
H	19.29064200	-1.02906900	1.03262100
C	19.62300100	0.47716000	-1.24482000
H	20.48271500	0.19744500	-0.62831400
H	19.99777300	0.99766800	-2.13088300
H	19.14367800	-0.44715600	-1.57494600
C	19.05826100	4.99205500	0.92696400
H	19.34094700	5.47355400	-0.01579600
H	19.96536600	4.81859200	1.50740500
H	18.41241000	5.68276900	1.47381600
O	-1.93095800	-0.28632300	-0.02722800

H	-2.01159000	0.67331200	-0.23184900
O	-5.48384200	0.22570700	-2.36242800
H	-5.78231900	-0.69603400	-2.52850700
O	-1.84500900	0.66594000	-2.80331500
O	-0.04418600	-0.62769100	-2.17389600
O	1.48456000	-4.73151900	-0.49894700
H	1.96640900	-3.93660800	-0.21208500
O	1.70809400	-4.00148700	-2.70402300
O	-2.48359100	-3.22143100	-3.44824900
O	-2.80017600	-5.06566300	-2.24815600
O	3.84954200	-3.30436900	-0.46837500
H	4.57861700	-2.71564300	-0.20837700
O	6.31785900	-2.15837500	-0.54137000
H	7.01183200	-2.40272700	0.10558000
O	8.55284900	-3.30569400	-0.27994200
H	9.27999500	-2.65965400	-0.15539900
O	10.47485900	-1.42279700	-0.30894700
H	11.09859800	-1.07418100	0.35364400
O	12.82267300	-0.62549600	0.65501800
H	13.43915300	-0.10254200	1.19802300
O	15.26341200	0.37372000	1.25525400
O	16.99026400	-0.31295800	-0.01916800
O	20.34697300	2.43957500	0.91194000
H	21.09542700	2.05999400	0.43934300
O	-1.00839800	-7.07896000	-2.76051100
H	-1.93881600	-6.84167600	-2.62622800

N	-4.36198900	-1.55415700	-0.32468800
H	-5.33302300	-1.23403400	-0.34324000
H	-3.85108800	-1.20017800	0.49378500
H	-4.39002900	-2.61176800	-0.37769600

AmB-DSPEc

0 1

C	8.44721900	-1.52852600	1.20672000
H	8.93057300	-0.54741500	1.12901400
C	9.12175600	-2.60233000	0.37527400
H	8.58935700	-3.55312900	0.51971100
C	9.00017800	-2.22620000	-1.11156700
H	9.51065900	-1.26440600	-1.26201000
C	6.99550800	-1.03714400	-0.66709700
H	7.57931000	-0.12067200	-0.75449900
C	6.99301500	-1.40582100	0.80646400
H	6.53679200	-0.56854900	1.33727000
C	9.58810900	-3.27947300	-2.02428200
H	9.10178000	-4.24617100	-1.85850600
H	10.66221700	-3.38664300	-1.85303400
H	9.44206500	-2.99245800	-3.06684600
C	5.36344300	0.04075100	-2.13581100
H	5.60627100	-0.44397800	-3.09352900
C	3.88350100	0.17977800	-1.98334600

H	3.56363500	0.79977100	-1.15237500
C	2.96708500	-0.49167400	-2.68825100
H	3.26331500	-1.13773700	-3.51736000
C	1.56473900	-0.41890000	-2.33020700
H	1.31815300	0.32078700	-1.57536100
C	0.56653400	-1.21905600	-2.74841600
H	0.73724400	-2.01000900	-3.47759000
C	-0.74496600	-1.06823400	-2.16016800
H	-0.83039300	-0.23408600	-1.46602100
C	-1.83195300	-1.84021800	-2.35472900
H	-1.80313900	-2.68528900	-3.04137600
C	-3.05902200	-1.57614400	-1.64256300
H	-3.04607400	-0.71658000	-0.97119000
C	-4.19801500	-2.29091100	-1.74205700
H	-4.24266900	-3.14381100	-2.41835100
C	-5.37738300	-1.99005300	-0.96974500
H	-5.30638600	-1.15136100	-0.27689300
C	-6.54204800	-2.66517900	-1.04128600
H	-6.63821100	-3.49324600	-1.74262600
C	-7.68724700	-2.35005000	-0.22242800
H	-7.55256400	-1.56489700	0.51959900
C	-8.89605200	-2.93281000	-0.31935900
H	-9.06571300	-3.70120600	-1.07272000
C	6.13694500	1.35561000	-2.04776000
H	7.20578600	1.15421100	-2.15222200
H	5.85612800	1.95225700	-2.92107900

C	-10.01799700	-2.54490400	0.51322900
H	-9.78385900	-1.85757600	1.32363300
C	-11.28853100	-2.91326400	0.31775600
H	-11.52692200	-3.58739700	-0.50661500
C	-12.45499000	-2.41833800	1.12513800
H	-12.07759000	-1.88224600	2.00335800
C	-13.30561900	-1.42090700	0.31439200
H	-13.79177600	-1.98966000	-0.49585700
C	-12.51057700	-0.28839400	-0.36310500
H	-11.78429300	-0.78390200	-1.01454300
C	-11.67457100	0.53899100	0.61931000
H	-11.13560700	-0.12262200	1.29165400
C	-9.39592500	1.13951700	0.15679800
C	-8.49362800	1.91774900	-0.76691300
H	-8.97692700	2.02674800	-1.73960600
H	-8.38115800	2.92011800	-0.33717400
C	-7.10723500	1.28432400	-0.90864200
H	-7.21125800	0.28071600	-1.34592700
C	-6.21364200	2.13730500	-1.79822100
H	-6.67654200	2.21598400	-2.78791900
H	-6.16044500	3.14814100	-1.37635200
C	-4.78814000	1.61569700	-1.96509800
H	-4.81370400	0.58104900	-2.33515400
C	-4.00547400	2.48123100	-2.95343400
H	-4.20634300	3.53585100	-2.73217200
H	-4.41166900	2.28457800	-3.95040300

C	-2.48836100	2.23298300	-2.93092100
H	-2.27506500	1.25597200	-2.49123900
H	-2.09275300	2.20628200	-3.95084200
C	-1.70564900	3.30214600	-2.16170600
H	-1.68374600	4.22511900	-2.75837200
C	-0.26476600	2.85069400	-1.88799800
H	0.15907100	2.38992500	-2.78873900
C	0.63454300	4.00214000	-1.43836600
H	0.12719400	4.58067200	-0.65938200
H	0.79761000	4.66975300	-2.29285000
C	1.97132300	3.51383300	-0.88770300
H	2.34808900	2.70258900	-1.51935600
C	3.04875100	4.59581400	-0.83921200
H	2.71636600	5.42365800	-0.20451300
H	3.21081000	4.98576400	-1.84789000
C	4.38332700	4.07480600	-0.28992500
C	5.52115800	5.08062500	-0.35012400
H	5.26978200	5.96889900	0.23497400
H	5.69122100	5.38233700	-1.38817200
C	6.78587800	4.44375000	0.19951500
H	6.61268800	4.19987600	1.25787400
C	7.11086200	3.13766200	-0.55394300
H	7.51734300	3.46853100	-1.51894200
C	5.89516600	2.23894400	-0.81477100
H	5.71249000	1.62673100	0.07311100
C	8.25696800	2.42891300	0.21292300

C	-12.41131400	1.59002400	1.42441000
H	-11.72624800	2.07151900	2.12603000
H	-13.21902400	1.11236200	1.98059700
H	-12.83470900	2.35934600	0.77501000
C	-13.41380500	0.57789100	-1.23955200
H	-14.21319500	1.05018000	-0.66063200
H	-13.87702300	-0.02193800	-2.02829900
H	-12.84010100	1.37602300	-1.71581100
C	-13.32728400	-3.57751000	1.61479500
H	-13.70473400	-4.16051200	0.76723700
H	-14.18219300	-3.20027300	2.17741800
H	-12.75033600	-4.25100300	2.25262600
O	6.40833700	-2.64612300	1.13500500
H	5.43299000	-2.68404900	0.92709000
O	10.45630100	-2.68465900	0.84565100
H	10.87538000	-3.47428200	0.48524800
O	7.61940600	-2.08453900	-1.44153600
O	5.69204500	-0.89473100	-1.07200700
O	4.24792400	3.68900200	1.05227200
H	3.32545000	3.37704000	1.14365600
O	4.70470400	2.96791200	-1.12927500
O	8.06018700	1.23822100	0.61068000
O	1.78709100	3.01842900	0.44798900
H	1.68274400	2.05050400	0.41635900
O	-0.30145300	1.81164000	-0.91978800
H	-0.83492900	2.15682500	-0.18936700

O	-2.29272100	3.62469400	-0.90995900
H	-2.95445300	2.94144600	-0.68679400
O	-4.09336600	1.62097700	-0.71319200
H	-4.73222700	1.37886700	-0.01777400
O	-6.47371800	1.17828300	0.36375400
H	-7.12557500	0.80069800	0.98072500
O	-8.98621500	0.52482200	1.12882900
O	-10.67120800	1.22481300	-0.19369400
O	-14.29617300	-0.92151400	1.20769700
H	-15.00531000	-0.52361000	0.69150100
O	7.85022600	5.35427300	0.06312500
H	8.64702700	4.80201400	0.21905800
N	8.45404900	-1.97057200	2.64240000
H	7.67744000	-2.65154600	2.71669200
H	8.26445300	-1.17753900	3.26041000
C	-4.14439400	3.88462700	2.02244800
H	-4.45498300	3.42631700	1.08001000
H	-4.73078100	3.42015200	2.82246000
C	-2.64769000	3.70178400	2.25711300
H	-2.35919500	4.19064300	3.19570700
H	-2.09247600	4.19615100	1.45430400
C	-2.24173600	2.23123100	2.31125000
H	-2.49063200	1.73944500	1.36498500
H	-2.83186100	1.71624400	3.07974600
C	-0.75318800	2.04767900	2.61412400
H	-0.47632600	2.55263000	3.54245800

H	-0.13076600	2.49586400	1.82943300
C	-0.31988000	0.61522000	2.77086100
O	0.47519500	0.21589800	3.58802800
C	-0.65404400	-1.59492100	1.97043900
H	-0.45390100	-1.86672300	3.00917500
H	-1.56576800	-2.08655400	1.62252200
C	0.50706700	-2.02274700	1.09323900
H	0.45595700	-1.51660800	0.12946500
C	0.52752400	-3.52794200	0.88934900
H	0.63637500	-4.04326500	1.84891400
H	-0.39775100	-3.84484700	0.40217500
O	-0.93149000	-0.19527800	1.87825700
C	2.36769100	-0.57648800	1.40436100
C	3.75191700	-0.46202300	1.96344800
H	3.97120800	-1.34212200	2.57067000
H	4.39974900	-0.48828400	1.08164500
C	3.94154100	0.84820200	2.72530300
H	3.26535400	0.86528700	3.58749400
H	3.64458900	1.66430800	2.06904500
C	5.37966200	1.09738000	3.17256700
H	5.72318400	0.25171900	3.79046400
H	6.04309700	1.15135500	2.30088000
C	5.51425900	2.40393800	3.94862800
H	4.89382700	2.39583000	4.85087500
H	5.19506800	3.23764600	3.31794200
O	1.76637800	-1.72132200	1.71456700

O	1.83384500	0.27579400	0.71724600
O	1.59210800	-3.88738100	0.01998600
O	3.42310900	-5.36513400	-0.48925900
O	3.07268900	-4.66560300	2.01309500
O	4.00145000	-3.03049700	0.21575400
C	4.77461600	-5.59985000	-0.82832800
H	5.46417700	-5.24115000	-0.05367300
H	4.91372700	-6.67942600	-0.93204100
C	5.08647300	-4.92702300	-2.15736100
H	4.28874800	-5.17074700	-2.86025200
H	6.03737700	-5.27878500	-2.56735100
N	5.13814500	-3.45321400	-1.97976100
H	6.09249900	-3.11748300	-1.78845200
H	4.79053900	-2.93303500	-2.78151500
P	3.06297800	-4.19786400	0.61292900
O	9.28259500	3.10628000	0.40785700
H	-4.41361500	4.94351900	1.98154400
H	6.54986400	2.58614300	4.24907500
H	4.53866400	-3.15282200	-1.05887600
H	9.35968400	-2.38954100	2.87177000

AmB-Ret

0 1

C	5.72554000	-2.31256900	-1.83424800

C	6.06105100	-3.80888700	-1.71207100
C	7.51679500	-4.07533500	-2.12742800
C	8.49359000	-3.08995500	-1.50623400
C	8.15122900	-1.67451200	-1.94994800
C	6.67277000	-1.35709200	-1.92214400
H	7.77872600	-5.10707100	-1.86747300
H	8.45159800	-3.15185300	-0.41291200
H	9.52178700	-3.33756900	-1.78819400
H	8.67789700	-0.95017100	-1.32036000
H	8.51888100	-1.50202200	-2.97213300
H	7.58880900	-3.99821200	-3.21954000
C	6.39094200	0.11941800	-2.02028900
H	5.38673900	0.39074900	-1.69715500
H	7.10633300	0.68865300	-1.41899600
H	6.51038600	0.47171000	-3.05245800
C	5.83420300	-4.27961000	-0.26348900
H	4.80590000	-4.10186100	0.06317300
H	6.02077800	-5.35502100	-0.17956300
H	6.49592700	-3.76586100	0.43810400
C	5.15307300	-4.63052900	-2.64156200
H	5.45665100	-5.68209600	-2.62674300
H	4.10355300	-4.58249000	-2.34194000
H	5.22077000	-4.26998100	-3.67166000
C	4.27915800	-2.01973600	-1.80946200
H	3.70279200	-2.56802200	-1.06658700
C	3.60513200	-1.20337300	-2.62936600

H	4.16183300	-0.65354400	-3.38561100
C	2.15967900	-0.93568800	-2.58580700
C	1.28150000	-1.85915500	-2.14512000
H	1.67085800	-2.84179500	-1.88405600
C	1.77263100	0.43368300	-3.07486900
H	2.22031500	1.20156300	-2.43852900
H	2.15335500	0.59103700	-4.09032900
H	0.69617500	0.59789800	-3.09174000
C	-0.14806200	-1.69835500	-1.97908800
H	-0.57974100	-0.72445200	-2.18500600
C	-0.94576200	-2.69393300	-1.55613000
H	-0.49192500	-3.66369600	-1.35277600
C	-2.38963600	-2.61381700	-1.33896500
C	-3.03895900	-3.72728600	-0.96322400
H	-2.45171200	-4.63793000	-0.83696700
C	-3.06950700	-1.29336200	-1.57930500
H	-2.53513500	-0.47773200	-1.08561200
H	-3.08699500	-1.05785700	-2.64917500
H	-4.09362300	-1.27793700	-1.20996700
C	-4.50567500	-3.90877000	-0.71912100
H	-4.98753000	-4.33826800	-1.61201500
H	-5.00875700	-2.96554400	-0.50049200
O	-4.74292100	-4.73373900	0.41679500
H	-4.45687000	-5.63158200	0.21411600
C	-11.26818800	1.70177700	-1.66916000
H	-11.24407500	1.95023600	-2.73805600

C	-11.71994700	2.94748200	-0.92137100
H	-11.81340900	2.70207200	0.14515600
C	-10.65223900	4.04467700	-1.07359800
H	-10.54891000	4.25869000	-2.15077900
C	-8.87581300	2.48917800	-1.21108300
H	-8.57691300	2.74928400	-2.24354500
C	-9.87254400	1.34079500	-1.20948100
H	-9.50233000	0.56215300	-1.87281700
C	-11.00857800	5.31676400	-0.33487500
H	-11.12359200	5.11076300	0.73310800
H	-11.93830600	5.73771200	-0.72474600
H	-10.21669700	6.05775000	-0.45561400
C	-6.76946900	1.27464300	-1.07598800
H	-6.63982900	1.61888300	-2.11236300
C	-5.50002900	1.52395700	-0.31959800
H	-5.51029900	1.29218200	0.74266400
C	-4.38509000	1.97417900	-0.90303800
H	-4.38797700	2.18195000	-1.97356400
C	-3.13011300	2.17595400	-0.20574100
H	-3.13163700	2.02114000	0.87120700
C	-1.97667100	2.50481200	-0.81936200
H	-1.97411000	2.65959100	-1.89872600
C	-0.70897000	2.60654700	-0.13580800
H	-0.71132300	2.43910600	0.93672200
C	0.47066500	2.84260400	-0.74413900
H	0.49080200	3.03010500	-1.81741500

C	1.73655200	2.82363000	-0.05160900
H	1.71010400	2.54015400	0.99804400
C	2.92051200	3.08691200	-0.64135500
H	2.93241700	3.36804200	-1.69455300
C	4.20034600	3.02433100	0.02059000
H	4.20907700	2.74158700	1.07299100
C	5.36593800	3.28053300	-0.60662400
H	5.34036100	3.54358900	-1.66395700
C	6.66814000	3.22148700	0.01044400
H	6.70740100	2.99423800	1.07399100
C	7.81989600	3.39548900	-0.66467800
H	7.77841600	3.59422900	-1.73537000
C	-7.14752000	-0.22337900	-1.13178100
H	-7.98626900	-0.37549800	-1.80437400
H	-6.30099900	-0.74708400	-1.58638000
C	9.13452700	3.27328000	-0.06669900
H	9.16028900	3.16200300	1.01476300
C	10.27639200	3.21835100	-0.76131100
H	10.23976500	3.31117900	-1.84825100
C	11.63451700	2.97485000	-0.16937300
H	11.56067000	3.01643400	0.92322500
C	12.15124700	1.57009800	-0.53883700
H	12.36441700	1.57483400	-1.62082000
C	11.14962200	0.42592900	-0.29500300
H	10.26217400	0.68076800	-0.88220600
C	10.66836100	0.34798000	1.15768400

H	10.42741900	1.34403300	1.51835900
C	8.34698800	0.12064700	1.72051800
C	7.14696700	-0.78658400	1.65605800
H	7.22549800	-1.43989800	0.78587900
H	7.17358300	-1.41636400	2.55338600
C	5.82511400	-0.01767100	1.62048800
H	5.78223400	0.56906800	0.69338700
C	4.64563400	-0.97755400	1.66156200
H	4.74412200	-1.67013500	0.82304500
H	4.69625000	-1.55826800	2.59039700
C	3.28200200	-0.29466600	1.58820800
H	3.23730400	0.32365100	0.67873600
C	2.15651400	-1.32193600	1.54080600
H	2.18494000	-1.90413500	2.47034400
H	2.38876900	-2.01191300	0.72339100
C	0.74346100	-0.78380200	1.29807700
H	0.75093800	-0.08531900	0.45363900
H	0.11466300	-1.62382900	0.98576900
C	0.05695300	-0.09228100	2.47151300
H	0.15442700	-0.70844500	3.38016400
C	-1.43615400	0.12703700	2.16843600
H	-1.53372100	0.31961700	1.09170100
C	-2.33890600	-1.04351800	2.53197300
H	-2.28228500	-1.22140400	3.61199200
H	-1.98911700	-1.94534100	2.01806900
C	-3.79194700	-0.79555100	2.12778600

H	-3.82282400	-0.58854600	1.05166600
C	-4.68543700	-1.99502800	2.41488500
H	-4.64035100	-2.23535000	3.48196600
H	-4.32478900	-2.86359700	1.85885400
C	-6.15518300	-1.74975700	2.06760300
C	-7.00560800	-3.00892300	2.13594500
H	-7.06824000	-3.35336400	3.17065700
H	-6.54044100	-3.79474600	1.53659200
C	-8.40370500	-2.73237300	1.61704400
H	-8.88947700	-2.00076900	2.27408000
C	-8.36651700	-2.12058000	0.20315100
H	-7.94185700	-2.85666200	-0.49081500
C	-7.42854100	-0.88221300	0.21638500
H	-7.84460600	-0.15492600	0.91622000
C	-9.80465800	-1.86570600	-0.23227700
C	11.59222100	-0.33902800	2.14244600
H	11.17374700	-0.28297900	3.14999600
H	12.56193100	0.16042100	2.12816000
H	11.72987400	-1.39150700	1.88544100
C	11.68704700	-0.90790900	-0.81018300
H	12.62490900	-1.18576400	-0.31997700
H	11.86876300	-0.86099000	-1.88788700
H	10.97168700	-1.71219300	-0.62543200
C	12.63947400	4.03915000	-0.61909400
H	12.71932000	4.05729800	-1.71173100
H	13.62729900	3.82737700	-0.20759800

H	12.32271100	5.03173200	-0.29109000
O	-9.98598500	0.84684300	0.11285500
H	-9.24425700	1.21661400	0.61387500
O	-12.95998300	3.34925100	-1.48001100
H	-13.41162400	3.92123700	-0.85065100
O	-9.40037100	3.60691700	-0.54876100
O	-7.76009400	2.11242200	-0.44673000
O	-6.73570400	-0.79674400	2.91001800
H	-6.06153300	-0.10623300	3.04805200
O	-6.15294200	-1.28227300	0.70856100
O	-9.94329600	-1.56486400	-1.53052800
O	-10.76395700	-1.97726600	0.50292700
O	-4.34294100	0.33253600	2.81742900
H	-3.62297900	0.95271000	3.02647600
O	-1.89346500	1.28007000	2.87353600
H	-1.09057700	1.76705800	3.13162000
O	0.58097200	1.20344200	2.71907900
H	1.54549700	1.11173000	2.88731900
O	3.09396700	0.53737800	2.73681700
H	3.95269700	0.94086800	2.96238500
O	5.71158200	0.86098700	2.73830400
H	6.55782400	1.33561800	2.81879200
O	8.33756300	1.20623600	2.27899300
O	9.41418700	-0.40390300	1.13023400
O	13.36304900	1.38710300	0.18631000
H	13.86173800	0.67047100	-0.22040500

O	-9.11663600	-3.95368800	1.62436400
H	-10.04404800	-3.70909900	1.50197300
N	-12.09676000	0.50765000	-1.50063500
H	-12.12527600	0.26960100	-0.51018300
H	-10.84906500	-1.19590400	-1.66279400
H	-13.04668000	0.70969800	-1.79663000

AmB-Ret

0 1

C	4.36568000	2.56554300	1.22024000
C	3.06968400	2.78318500	1.98502700
C	2.01837500	3.63382200	1.25790900
C	4.08117400	1.97075100	-0.18365700
C	2.61646400	2.02799400	-0.53750400
C	1.97156900	0.94548600	-0.96683000
C	0.49676300	0.87995100	-1.21656000
C	-0.24730800	2.02571000	-0.53233800
C	-1.69507400	2.09669400	-1.00683100
C	-2.56668700	0.86570800	-0.78413800
C	-4.01236400	1.39563000	-0.89859400
C	-3.95313500	2.95701900	-0.95380800
C	-4.32017100	3.56634900	-2.33157500
C	-5.50785000	2.86229800	-3.02299800
C	-6.77912200	2.74271300	-2.18113800

C	-7.94354800	2.12914000	-2.95465600
C	-9.20322300	1.88905200	-2.11584600
C	-9.75064200	3.17887200	-1.50271500
C	-10.28278800	1.19314600	-2.94342700
C	-4.64249400	5.06315300	-2.21387000
C	-2.51516700	3.25167100	-0.40350900
C	-2.60699700	3.16276700	1.13259300
C	-1.80994700	4.55459300	-0.79842400
C	-0.34604700	4.57047700	-0.33620300
C	0.47405100	3.35780700	-0.80996400
C	1.93647000	3.36404800	-0.27679900
C	2.72672300	4.46346600	-1.01719500
O	5.08461700	3.79310600	1.14644900
H	0.54780900	3.43105100	-1.90666300
H	-0.23102300	1.82839400	0.54825300
H	-1.65975300	2.28512200	-2.09189200
H	4.97083000	1.84272300	1.77672500
H	3.30102100	3.22539100	2.95893900
H	2.64813400	1.79108600	2.17220600
H	2.21507700	4.69729100	1.42323800
H	1.05271100	3.42018200	1.72650700
H	4.42507200	0.93560000	-0.22834400
H	4.68263800	2.53207200	-0.90633800
H	2.54454500	0.03659900	-1.12524700
H	0.11045200	-0.08023300	-0.85571500
H	0.28901800	0.90245000	-2.29741200

H	-2.37369800	0.45870200	0.21024600
H	-2.35344700	0.06835100	-1.50135300
H	-4.60714900	1.08116500	-0.03879800
H	-4.50317300	0.97709900	-1.77637500
H	-4.68500200	3.35885300	-0.24352100
H	-3.45941700	3.45671000	-3.00509200
H	-5.74393100	3.42315100	-3.93618300
H	-5.21400100	1.86519200	-3.36751600
H	-7.06585900	3.73206500	-1.81260300
H	-6.58524300	2.13047100	-1.29234600
H	-7.62108700	1.16913700	-3.37850500
H	-8.19713400	2.77285900	-3.80881300
H	-8.91224300	1.21354700	-1.30194900
H	-9.94307700	3.92299000	-2.28390900
H	-9.05598800	3.61882700	-0.78338400
H	-10.69511600	2.99856900	-0.98096600
H	-10.61994100	1.83974600	-3.76101500
H	-11.15844300	0.94576400	-2.33426200
H	-9.90635400	0.26652500	-3.38694200
H	-5.48001600	5.22852000	-1.52931200
H	-4.92626100	5.47437200	-3.18750200
H	-3.80427100	5.64816000	-1.84224500
H	-3.14976800	2.27461900	1.46424900
H	-3.14642200	4.03505200	1.51562000
H	-1.63028300	3.14006900	1.61765900
H	-2.32211500	5.42435800	-0.37282700

H	-1.83439100	4.66710200	-1.88804100
H	-0.32044800	4.63446400	0.75418000
H	0.11998100	5.49172400	-0.69749100
H	2.21562100	5.42602400	-0.93341500
H	2.82398500	4.22273700	-2.08089900
H	3.72225700	4.58429100	-0.58795300
H	5.98475300	3.54155100	0.89057400
C	10.56602800	2.02518200	0.63084200
H	10.90142000	2.21083800	1.65933200
C	10.05587500	3.34755500	0.07240600
H	9.77186100	3.19930200	-0.97810600
C	8.80573500	3.78413500	0.85435800
H	9.09134300	3.89273000	1.91283200
C	8.14474700	1.54124400	1.29037200
H	8.24980300	1.61932300	2.38687300
C	9.42473700	1.03153600	0.65101000
H	9.75709400	0.15779100	1.20771800
C	8.20718800	5.08018800	0.35134600
H	7.91198600	4.97723200	-0.69685100
H	8.93318600	5.89090400	0.44375000
H	7.32157900	5.34626300	0.93192900
C	6.92529700	-0.53773200	1.70952400
H	7.09728500	-0.29817800	2.76825200
C	5.48762100	-0.88773200	1.49963700
H	5.20597000	-1.13951800	0.48155500
C	4.55143200	-0.83760600	2.45146700

H	4.81982100	-0.58904000	3.47793500
C	3.14901700	-1.01162000	2.13985300
H	2.91679000	-1.20687600	1.09719000
C	2.12517000	-0.84807500	2.99804500
H	2.31669500	-0.62740800	4.04740800
C	0.75845600	-0.88672700	2.53967000
H	0.63134800	-1.09965700	1.48003000
C	-0.34093200	-0.64650900	3.28190800
H	-0.25270000	-0.41433700	4.34220900
C	-1.65155900	-0.64337600	2.68405900
H	-1.68647000	-0.91209000	1.62929900
C	-2.80951800	-0.31377600	3.29212800
H	-2.81438000	-0.04585800	4.34764000
C	-4.05544700	-0.24332900	2.57009200
H	-4.00701100	-0.47730300	1.50762800
C	-5.24923000	0.11836300	3.08263400
H	-5.33699300	0.34592100	4.14401400
C	-6.43074700	0.24488900	2.26417800
H	-6.29003100	0.09707100	1.19487400
C	-7.67341200	0.52684200	2.69602500
H	-7.86679200	0.65819800	3.75968400
C	7.88801100	-1.67076700	1.30731300
H	8.91019500	-1.41633800	1.57423700
H	7.62057000	-2.54422200	1.91036500
C	-8.78799900	0.63465800	1.77512700
H	-8.51670300	0.61304900	0.72271500

C	-10.08232300	0.71154800	2.10096500
H	-10.37591200	0.71726400	3.15168500
C	-11.19447900	0.76010200	1.09090600
H	-10.76103100	0.96492400	0.10766100
C	-11.92989600	-0.58900100	0.98305900
H	-12.46349300	-0.75133400	1.93424600
C	-11.01735200	-1.81136400	0.77191100
H	-10.28168600	-1.77981500	1.58065400
C	-10.20251000	-1.73694700	-0.52657400
H	-9.79638700	-0.73669300	-0.65409800
C	-7.83714100	-2.18651300	-0.51450300
C	-6.80223500	-3.27122400	-0.34842900
H	-7.21291100	-4.07238200	0.26888500
H	-6.60836200	-3.68462100	-1.34514100
C	-5.48799100	-2.74767200	0.23269500
H	-5.67751700	-2.33177800	1.23288900
C	-4.45501400	-3.86208000	0.34038300
H	-4.83844900	-4.63158700	1.01903000
H	-4.32361100	-4.32392200	-0.64548300
C	-3.08709500	-3.39935000	0.83555300
H	-3.20588000	-2.84237700	1.77499900
C	-2.14688700	-4.57891200	1.08035400
H	-2.23351900	-5.28389900	0.24507700
H	-2.50093700	-5.10338900	1.97273400
C	-0.67821100	-4.15577900	1.25245100
H	-0.62480900	-3.10573800	1.54905400

H	-0.21071000	-4.72556200	2.06121800
C	0.17612400	-4.37030100	-0.00052800
H	0.36222900	-5.44529300	-0.12175200
C	1.53326600	-3.63575700	0.11159500
H	1.82895700	-3.54598000	1.16288200
C	2.65304900	-4.30658800	-0.68115100
H	2.33527400	-4.40847600	-1.72585700
H	2.81099100	-5.31622600	-0.28491900
C	3.97402200	-3.53742700	-0.62707900
H	4.27849400	-3.41017600	0.42099400
C	5.09132000	-4.26558100	-1.36957400
H	4.75180100	-4.53618500	-2.37430300
H	5.32528100	-5.18804400	-0.83019400
C	6.37080400	-3.44110800	-1.53176100
C	7.54026500	-4.24556700	-2.08218700
H	7.31998500	-4.59274200	-3.09390400
H	7.71352700	-5.11737300	-1.44420500
C	8.78694800	-3.38195200	-2.10728200
H	8.61513600	-2.54447100	-2.79221200
C	9.08630900	-2.78942100	-0.71825700
H	9.35655100	-3.60605200	-0.03780900
C	7.81352400	-2.09819300	-0.15684000
H	7.58534600	-1.24538100	-0.79931200
C	10.30473200	-1.88371800	-0.85700700
C	-10.90666600	-2.16854100	-1.79709200
H	-10.24175600	-2.04117100	-2.65388200

H	-11.79366900	-1.55216700	-1.94272600
H	-11.20206200	-3.21849700	-1.74541200
C	-11.79774700	-3.11884700	0.90059500
H	-12.61299100	-3.18465900	0.17324300
H	-12.23103300	-3.21162900	1.90033000
H	-11.14414500	-3.97861500	0.73582300
C	-12.18532900	1.88182700	1.41135700
H	-12.63194400	1.73354400	2.40089300
H	-12.98896600	1.90527300	0.67422200
H	-11.67866200	2.84982200	1.41508200
O	9.15515400	0.68269300	-0.69476300
H	8.19297300	0.62729800	-0.78618700
O	11.10212100	4.29325900	0.20844000
H	10.94516100	5.02097000	-0.40244000
O	7.78204100	2.78693100	0.74122100
O	7.09612300	0.68109000	0.94254000
O	6.17406000	-2.35539600	-2.39090000
H	5.30720600	-1.97575100	-2.15782200
O	6.71708600	-3.00440000	-0.21738100
O	10.84802000	-1.50877400	0.30932200
O	10.78612500	-1.55865000	-1.92146400
O	3.81752600	-2.24125300	-1.21276000
H	2.89328500	-1.96747200	-1.05922700
O	1.32094400	-2.30094200	-0.36098700
H	0.64195600	-2.42328500	-1.05547600
O	-0.44698200	-3.91530000	-1.19478300

H	-1.24636700	-3.39665600	-0.95637700
O	-2.46712800	-2.53490500	-0.12219500
H	-3.16222000	-1.96324300	-0.49879500
O	-4.93867800	-1.73231500	-0.60174700
H	-5.64716700	-1.09745700	-0.80785500
O	-7.55549100	-1.03251300	-0.79885800
O	-9.06812000	-2.64257500	-0.34321400
O	-12.87239700	-0.44119800	-0.07625300
H	-13.52913000	-1.14257100	-0.00793600
O	9.86471900	-4.17866000	-2.55833000
H	10.56538600	-3.55666000	-2.79591200
N	11.66649100	1.39639400	-0.09774400
H	11.36378300	1.22271800	-1.05470400
H	11.47146700	-0.76643500	0.13433700
H	12.45637500	2.03319400	-0.13503200

AmB-Dod

0 1

C	-4.82395700	-0.23466700	3.60154400
H	-5.05853900	-1.13553800	3.02289000
H	-5.50708000	0.55380700	3.27232900
H	-5.03972100	-0.45511400	4.65177000
C	-3.36820400	0.17186800	3.40012500
H	-3.15513200	1.08613500	3.96864200

H	-3.21260300	0.42998700	2.34936100
C	-2.37264200	-0.91577500	3.78890500
H	-2.64072800	-1.84097900	3.25957500
H	-2.46204700	-1.14813500	4.85822600
C	-0.92640900	-0.56240200	3.44605500
H	-0.60700900	0.31362200	4.02454500
H	-0.88009600	-0.25056100	2.39700000
C	0.04303400	-1.72270800	3.67576700
H	-0.45387700	-2.66384700	3.40222500
H	0.27653200	-1.81269300	4.74416400
C	1.33254200	-1.62150300	2.86529200
H	1.81729600	-0.65153300	3.03145600
H	1.07326400	-1.63911700	1.79908500
C	2.31227800	-2.75530100	3.15954300
H	1.75616100	-3.69890000	3.24392500
H	2.77717600	-2.59414300	4.14072300
C	3.39166400	-2.92122600	2.09487200
H	3.90394500	-1.96724600	1.91857200
H	2.90832900	-3.17229700	1.14091100
C	4.41563600	-4.00053600	2.43680900
H	3.89144600	-4.93021200	2.69757400
H	4.96830600	-3.70305900	3.33762200
C	5.39955600	-4.26781700	1.30371400
H	5.88090700	-3.32953300	1.01425400
H	4.84168300	-4.60733600	0.41970600
C	6.46576200	-5.30578800	1.63855300

H	5.99574800	-6.27720000	1.83733200
H	6.99444200	-5.01408100	2.55382900
C	7.49900600	-5.46371200	0.53306400
H	8.15379000	-6.32065000	0.75289700
H	6.98777800	-5.68500300	-0.41679900
O	8.25004200	-4.27202400	0.44632600
H	8.52270300	-4.09444100	-0.47240700
C	9.84403300	3.23351200	0.51074300
H	9.96457300	3.54657700	1.55574900
C	9.78866200	4.49371900	-0.33954500
H	9.73090400	4.20189500	-1.39706200
C	8.51792100	5.28582000	0.01301600
H	8.57439300	5.54344900	1.08412900
C	7.28571500	3.33645100	0.54323100
H	7.12910900	3.58296700	1.60972000
C	8.53517400	2.48898300	0.36213700
H	8.52295900	1.69861000	1.10819400
C	8.36783300	6.55008200	-0.80561300
H	8.32445200	6.30487800	-1.87063600
H	9.20669200	7.22584600	-0.62335900
H	7.44453000	7.06578700	-0.53683600
C	5.57532700	1.61947900	0.85658400
H	5.50282800	2.01683300	1.87910900
C	4.19956600	1.45591000	0.28804600
H	4.14152200	1.08586200	-0.73171600
C	3.07818000	1.74960500	0.95202100

H	3.13358200	2.12069500	1.97546000
C	1.76122700	1.63183800	0.35933900
H	1.72201300	1.24535000	-0.65588800
C	0.61469100	1.99501100	0.96348900
H	0.64340300	2.39030100	1.97863900
C	-0.67360900	1.89526400	0.32066700
H	-0.67871200	1.46126500	-0.67734900
C	-1.83911200	2.27876300	0.87863500
H	-1.83856400	2.70832100	1.87985300
C	-3.11699900	2.12482400	0.22908000
H	-3.12241200	1.65322300	-0.75292700
C	-4.29335300	2.47848900	0.78620200
H	-4.29386500	2.92693800	1.77923600
C	-5.57275800	2.29375400	0.15064900
H	-5.57320000	1.84244200	-0.84099500
C	-6.75243000	2.63022200	0.71096300
H	-6.76068200	3.06182900	1.71142400
C	-8.02520900	2.45803700	0.05451200
H	-8.00152400	2.08011700	-0.96594200
C	-9.22208700	2.71173400	0.61460200
H	-9.26971600	3.06507900	1.64402700
C	6.35320000	0.28761000	0.91902200
H	7.30838500	0.42374100	1.41761200
H	5.77485900	-0.39181000	1.55259000
C	-10.47580300	2.49202100	-0.07943600
H	-10.39078900	2.23612500	-1.13360200

C	-11.68613900	2.52264900	0.48856900
H	-11.77020700	2.76242900	1.54986300
C	-12.97008300	2.19480500	-0.21850600
H	-12.77086900	2.10175600	-1.29224900
C	-13.53628100	0.84214500	0.25626500
H	-13.88174000	0.97813300	1.29466000
C	-12.52042400	-0.31499500	0.28198000
H	-11.71419200	0.01152400	0.94608900
C	-11.85169900	-0.56357900	-1.07445200
H	-11.54085400	0.38155300	-1.51073400
C	-9.48401400	-0.89079000	-1.29121300
C	-8.32253400	-1.76874600	-0.89900400
H	-8.55997400	-2.29088200	0.02976200
H	-8.20752400	-2.52369900	-1.68559900
C	-7.01440900	-0.98439900	-0.77112400
H	-7.12225000	-0.22890500	0.02016000
C	-5.85608700	-1.91068400	-0.43124700
H	-6.07730700	-2.41586100	0.51517700
H	-5.77916700	-2.67990900	-1.20905100
C	-4.50463100	-1.21195900	-0.30737200
H	-4.58161700	-0.39700900	0.42601500
C	-3.42479700	-2.18745700	0.14776400
H	-3.47941300	-3.09035600	-0.47271000
H	-3.67050800	-2.49295400	1.16962300
C	-2.00401100	-1.60626500	0.10229800
H	-2.04053300	-0.51360600	0.13542400

H	-1.45011400	-1.91957800	0.99161500
C	-1.17510100	-2.04666200	-1.10261000
H	-0.99435500	-3.12728000	-1.02044200
C	0.19758500	-1.31785000	-1.13173200
H	0.41463300	-0.90596900	-0.14043100
C	1.35103700	-2.21974900	-1.55706100
H	1.16034200	-2.59332000	-2.57065600
H	1.38739500	-3.08683900	-0.88725200
C	2.70261300	-1.51159800	-1.50890900
H	2.87516300	-1.14587700	-0.48801800
C	3.84574700	-2.44641700	-1.88394100
H	3.66887300	-2.85999900	-2.88178400
H	3.86911700	-3.27305200	-1.16971800
C	5.21800500	-1.77294700	-1.89648400
C	6.35620200	-2.76505900	-2.09324800
H	6.30545900	-3.19951000	-3.09383100
H	6.26396500	-3.56519800	-1.35594000
C	7.69353100	-2.07881300	-1.90372500
H	7.83354200	-1.33748200	-2.69743000
C	7.77337400	-1.34905600	-0.55074200
H	7.71678300	-2.08980700	0.25405400
C	6.54695400	-0.40602000	-0.42730200
H	6.59816400	0.32932000	-1.23376000
C	9.14685600	-0.70125100	-0.45587400
C	-12.64895100	-1.35361900	-2.09198800
H	-12.09829800	-1.41407000	-3.03353600

H	-13.60201500	-0.85152500	-2.26395200
H	-12.84145600	-2.36933500	-1.73989200
C	-13.13315400	-1.58196600	0.87630200
H	-13.99453600	-1.92936000	0.29791500
H	-13.46473900	-1.40562900	1.90365800
H	-12.40220900	-2.39346700	0.89321300
C	-14.01375300	3.29818500	-0.02342500
H	-14.22035200	3.45013800	1.04182200
H	-14.94815200	3.02925100	-0.51799600
H	-13.65485200	4.24544600	-0.43217100
O	8.51808500	1.93261900	-0.94121200
H	7.61828500	2.05212000	-1.27959700
O	10.96129900	5.24132700	-0.06267700
H	11.10723900	5.86551400	-0.78124300
O	7.35217600	4.50194200	-0.23234500
O	6.19079700	2.62723300	0.02559400
O	5.31615300	-0.81208500	-2.90871300
H	4.45405600	-0.35921100	-2.94820100
O	5.36099700	-1.17754400	-0.60834200
O	9.44960200	-0.22421800	0.75351300
O	9.94538700	-0.68083400	-1.37348500
O	2.72965300	-0.39303900	-2.40182900
H	1.81350400	-0.07711100	-2.51295300
O	0.09088100	-0.20175700	-2.01620400
H	-0.62946100	-0.47452300	-2.62354400
O	-1.80937900	-1.81064700	-2.35053800

H	-2.68383800	-1.39246500	-2.19528700
O	-4.09706600	-0.65253200	-1.55908600
H	-4.89800300	-0.34410500	-2.02110500
O	-6.69823700	-0.33627200	-2.00072500
H	-7.50749800	0.11308900	-2.30359400
O	-9.36791100	0.08511600	-2.01568500
O	-10.63129300	-1.31405100	-0.78147000
O	-14.65063300	0.55976100	-0.58475300
H	-15.20147500	-0.10394000	-0.15612400
O	8.70600900	-3.07882200	-2.01277200
H	9.54148600	-2.58762600	-2.05138600
N	10.91499700	2.28659100	0.19670100
H	10.81821400	1.99963100	-0.77613300
H	10.23636500	0.36741600	0.66842300
H	11.81438900	2.74756500	0.29349800
